# UMGAP: the Unipept MetaGenomics Analysis Pipeline

**DOI:** 10.1186/s12864-022-08542-4

**Published:** 2022-06-10

**Authors:** Felix Van der Jeugt, Rien Maertens, Aranka Steyaert, Pieter Verschaffelt, Caroline De Tender, Peter Dawyndt, Bart Mesuere

**Affiliations:** 1 Department of Applied Mathematics, Computer Science and Statistics, Ghent University, Ghent, Belgium; 2 Department of Information Technology, IDLab, imec, Ghent, Belgium; 3 VIB-UGent Center for Medical Biotechnology, Ghent, Belgium; 4 Plant Sciences Unit, Flanders Research Institute for Agriculture, Fisheries and Food, Ghent, Belgium

**Keywords:** Shotgun metagenomics, Biodiversity analysis, Taxonomic profiling

## Abstract

**Background:**

Shotgun metagenomics yields ever richer and larger data volumes on the complex communities living in diverse environments. Extracting deep insights from the raw reads heavily depends on the availability of fast, accurate and user-friendly biodiversity analysis tools.

**Results:**

Because environmental samples may contain strains and species that are not covered in reference databases and because protein sequences are more conserved than the genes encoding them, we explore the alternative route of taxonomic profiling based on protein coding regions translated from the shotgun metagenomics reads, instead of directly processing the DNA reads. We therefore developed the Unipept MetaGenomics Analysis Pipeline (UMGAP), a highly versatile suite of open source tools that are implemented in Rust and support parallelization to achieve optimal performance. Six preconfigured pipelines with different performance trade-offs were carefully selected, and benchmarked against a selection of state-of-the-art shotgun metagenomics taxonomic profiling tools.

**Conclusions:**

UMGAP’s protein space detour for taxonomic profiling makes it competitive with state-of-the-art shotgun metagenomics tools. Despite our design choices of an extra protein translation step, a broad spectrum index that can identify both archaea, bacteria, eukaryotes and viruses, and a highly configurable non-monolithic design, UMGAP achieves low runtime, manageable memory footprint and high accuracy. Its interactive visualizations allow for easy exploration and comparison of complex communities.

**Supplementary Information:**

The online version contains supplementary material available at (10.1186/s12864-022-08542-4).

## Background

Biodiversity, in many environments, is formed by complex communities of archaea, bacteria, eukaryotes, and viruses. Most of these organisms are hard to isolate and culture in lab conditions, so getting insight into which species are present in these environments and estimating their abundances nowadays routinely relies on metagenomics [1]: a combination of high-throughput DNA sequencing and computational methods that bypass the cultivation step to enable genomic analysis. In particular, shotgun metagenomics, the non-targeted sequencing of all genomes in an environmental sample, is applied more often [2], as it allows the profiling of both taxonomic composition and functional potential of the sample.

In general, computational approaches for taxonomic profiling of metagenomics data from high-complexity environments directly process the reads by either assembling them into larger contigs before profiling [3–8] or by individually mapping them to DNA sequence databases [9–11], e.g., compiled from publicly available reference genomes. As the latter approach is carried out without assembly, it can mitigate assembly problems, speed up computations, and enable profiling of low-abundance organisms that cannot be assembled de novo [2]. Mapping a read onto a reference database usually either applies inexact string matching algorithms on the entire read or breaks it into shorter fragments before applying exact string matching algorithms.

### Aims

In this paper we explore the alternative route of first translating the protein coding regions in the reads of a shotgun metagenomics data set. We then map the resulting protein fragments onto a reference protein sequence database. Because protein sequences are more conserved than the genes encoding them [12], this might alleviate the limitation that environmental samples contain strains that are not covered in reference databases or even belong to yet uncharacterized species. In addition, it allows us to adapt the high-performance mapping algorithms based on periodic builds of a UniProtKB-based index [13] for mapping tryptic peptides that we developed for shotgun metaproteomics analysis in Unipept [14–17]. Mapping against the complete UniProt Knowledgebase has the advantage that it covers all domains of life in a single general-purpose analysis, compared to using one or more reference databases of selected genomes.

### Methods

**Fig. 1:**
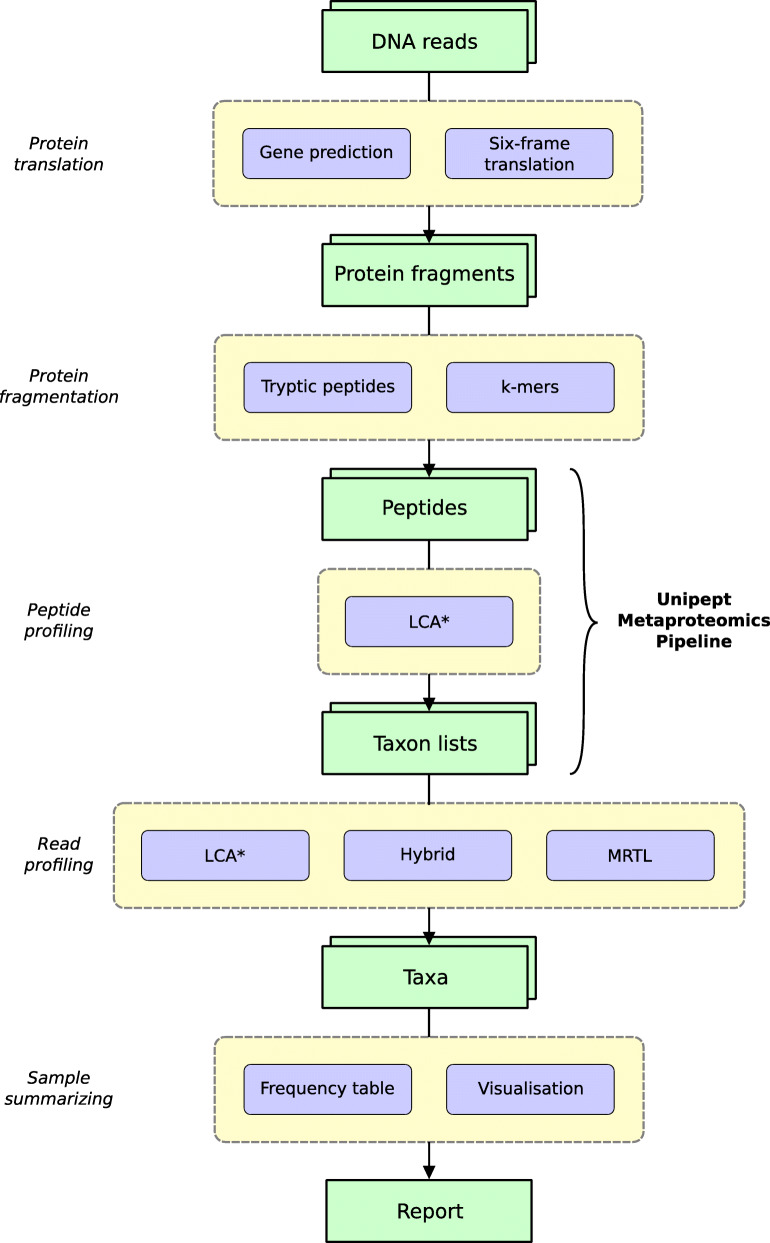
Outline of the Unipept Metagenomics Analysis Pipeline (UMGAP). Green blocks represent data types, purple blocks represent tools. Horizontally aligned purple blocks grouped in yellow boxes are alternative approaches for the same general step of the pipeline

The general steps involved in our approach for taxonomic profiling of a DNA read are outlined in Fig. 1. After identifying and translating the (partial) protein coding genes in the read, the protein fragments are split into short peptides. Each individual peptide is then mapped onto a precomputed consensus taxon derived from all proteins containing the peptide in the reference database. As a final step we derive a consensus taxon for the read from the consensus taxa of its individual peptides. For paired-end sequencing, the information content in the final step increases after merging the individual peptides from a read pair, as it is guaranteed that both reads originate from the same organism.

Each individual step in the above process can be tackled using a multitude of strategies. To explore which strategy performs best and how the combination of alternative strategies leads to different trade-offs with respect to runtime, memory footprint and predictive accuracy, we have implemented the Unipept Metagenomics Analysis Pipeline (UMGAP) according to the Unix philosophy [18]. The result is a modular suite of 20 versatile filters (commands that read from standard input and write to standard output) that each implement a single operation and that can be seamlessly combined into a single data processing pipeline. All filters are implemented in Rust (https://www.rust-lang.org) and support parallelisation to achieve optimal performance. As some filters implement alternative strategies of the same operation, we have performed a parameter sweep to collect performance metrics of all relevant combinations of alternative strategies. Based on our observations from this parameter sweep, we have selected six preconfigured pipelines with different performance trade-offs whose results have been compared in an established benchmark [19] to a selection of state-of-the-art shotgun metagenomics taxonomic profiling tools.

UMGAP has been open sourced on GitHub (https://github.com/unipept/umgap) under the MIT License. Documentation (https://unipept.ugent.be/umgap) and case studies (https://unipept.ugent.be/umgap/casestudies) are available on the Unipept website.

## Implementation

UMGAP performs taxonomic profiling of individual reads or read pairs in a shotgun metagenomics data set. Results can be summarized for the entire data set, either as a hierarchical frequency table containing each identified taxon or as an interactive taxonomic visualization. The pipeline executes a multi-step process and provides fast implementations of alternative strategies for every step of the analysis. In this section, we chronologically discuss the successive steps of the generic pipeline, together with their alternative strategies.

### Protein translation

**Fig. 2:**
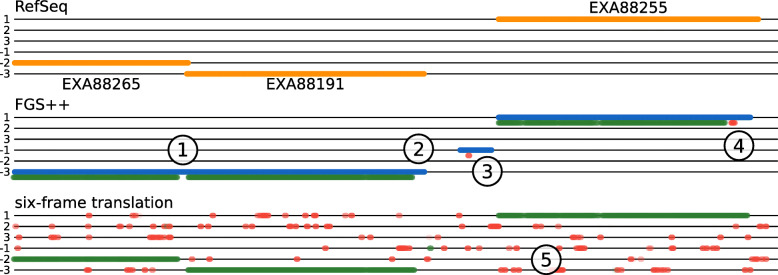
Sample DNA fragment extracted from the *Acinetobacter baumannii* 118362 genome (NCBI Assembly ASM58051v1, positions 37.700-39.530) containing three RefSeq annotated coding regions of a major Facilitator Superfamily protein (EXA88265), a tetR family protein (EXA88191) and a translocator family protein (EXA88255), marked with yellow lines (top). Blue lines indicate coding regions predicted by FGS. Green dots indicate starting positions of 9-mers with an LCA* on the *A. baumannii* lineage (true positive identifications). Red dots indicate starting positions of 9-mers with an LCA* outside the *A. baumannii* lineage (false positive identifications). Opacity of colored dots indicates depth in the taxonomic tree: opaque colors indicate highly specific LCA* (species level) and translucent colors indicate nonspecific LCA*. This example illustrates the following general observations: (1) the frameshift-correcting topology of the FGS hidden Markov model often incorrectly interprets coding regions of genes that are very close or overlapping as frameshifts and glues them together; (2) missing dots at the end of coding regions is merely an artefact of the visualization: the last 8 codons (24 bases) are never starting positions of *k*-mers; (3) FGS may identify false coding regions or (4) frame shifts, but the extracted peptides from those and (5) translations from non-coding regions in a six-frame translation are mostly filtered automatically as they have no exact match with any UniProt protein or can be filtered with additional heuristics

UMGAP does not profile a read directly at the DNA level. Instead, protein fragments translated from the coding regions in the read are matched. Non-coding regions are ignored a priori (which might impact the sensitivity compared to identification strategies that use the entire read, especially for organisms with a lower coding density) and extra steps are required to find coding regions and protein translations (which might negatively impact both performance and accuracy). However, the more conserved nature of proteins compared to DNA might lead to better generalizations when it comes to profiling environmental strains that have no perfect match in the reference database [12]. Two approaches are supported: one based on gene prediction in short reads and one based on a full six-frame translation (Fig. 2).

**Gene prediction** In theory, UMGAP may use any gene predictor capable of identifying coding regions and their translations in short reads. Not all gene predictors can do this task accurately, as reads might contain only partial coding regions with start and/or stop sections missing. The translation table to be used is also a priori unknown.

We used both FragGeneScan (FGS) [20] and our own faster and more robust implementation of FGS in Rust called FragGeneScanRs (FGSrs) [21] for testing and benchmarking purposes. FGS has a custom Hidden Markov Model whose topology especially addresses the problem of finding genes in short and error-prone reads, correcting frameshifts resulting from read errors. FragGeneScan-Plus (FGS+) [22] is a faster implementation of FGS. As FGS+ is no longer actively supported, our own implementation with improved multithreading support and several critical bug fixes has been released as FGSrs.

FGS and its derivatives are functionally equivalent and can thus be plugged into UMGAP interchangeably. They perform gene prediction relatively fast and accurate. However, due to their predictive nature, false negatives and, to a lesser extent, false positives might have a negative impact on downstream steps of the pipeline. Especially missed coding regions may lead to information loss and reduced precision of the pipeline.

Other gene prediction tools such as Prodigal [23], MetaGeneMark [24] and MetaGeneAnnotator [25] can also be plugged into UMGAP. However, this would also require an additional gene translator, as some of these tools merely predict the loci of genes but do not translate them into protein sequences.

**Six-frame translation** Translation of all coding regions is guaranteed when applied on all six reading frames of an error-free read, but at least 83.33% false positives (5 out of 6 reading frames) need to be filtered away downstream. UMGAP implements this strategy without attempting to correct for read errors at this stage, as they only result in local information loss in downstream steps. The translation table is user-specified, without an attempt to derive it from the data or using multiple tables. While this approach might lead to increased sensitivity compared to gene prediction, it yields at least a sixfold increase in the data volume that needs to be processed in downstream analysis.

### Protein fragmentation

All (partial) proteins that are putatively translated from the read are matched against the complete UniProt knowledgebase [22, 26]. Direct full-length exact matching is not feasible due to natural variation and read errors. Even though fast heuristics exist for full-length inexact matching or alignment [27], it remains a relatively slow approach. Instead, UMGAP achieves high-performance inexact matching of protein fragments by breaking them down into short peptides. Two approaches are supported: non-overlapping variable-length peptides and overlapping fixed-length peptides.

**Tryptic peptides** UMGAP breaks protein fragments into non-overlapping variable-length peptides by splitting after each lysine (K) or arginine (R) residue that is not followed by proline (P). This is the classic in silico emulation of a trypsin digest, the most widespread protein digestion used for mass spectrometry [28]. It is a random fragmentation strategy in the context of metagenomics, but finds its roots in the Unipept metaproteomics analysis pipeline as the initial starting point for UMGAP, and is merely an attempt to reuse part of the metaproteomics processing pipeline for metagenomics analysis. Applying this fragmentation strategy to all proteins in the UniProt Knowledgebase (2020/04 release) yields a collection of tryptic peptides with an average length of 17.671 amino acids (with peptides shorter than 5 or longer than 50 discarded).

Note that UMGAP also supports user-specified regular expressions for variable-length protein fragmentation, other than the default regular expression that mimics an in silico tryptic digest. However, the regular expression used for protein fragmentation must match the regular expression used when fragmenting proteins to build a peptide profiling index from a reference database. We currently only host a pre-built index for tryptic peptides extracted from UniProt, so for peptide profiling with a non-tryptic fragmentation strategy, a custom peptide profiling index needs to be built.

***K*-mers** With overlapping fixed-length peptides or *k*-mers, the only parameter is the length *k* of the peptides. Choosing smaller *k* leads to more spurious hits when matching the *k*-mers of a protein fragment against the *k*-mers inferred from a reference protein database. Choosing larger *k* increases the impact of natural variation and read errors. Because protein fragments are reduced to all their overlapping *k*-mers, the number of resulting peptides increases *k*-fold compared to using tryptic peptides. So, choosing larger *k* also increases the total length of all peptides and thus the memory footprint for indexing them. It also increases the number of lookups that need to be done during peptide profiling. Finding a well-balanced peptide length *k* is therefore crucial.

**Fig. 3:**
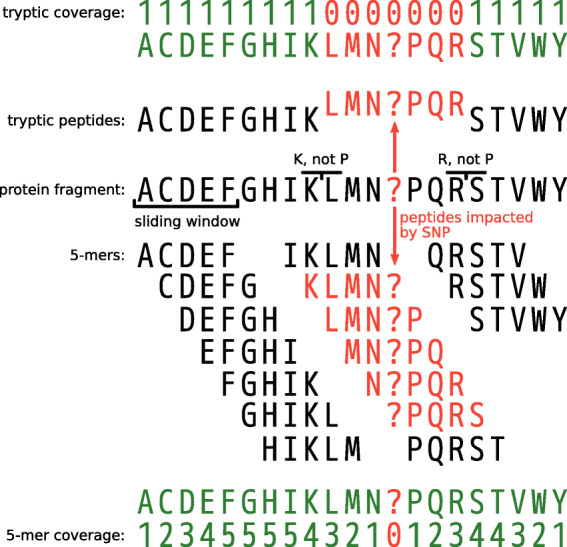
Impact of a single nucleotide polymorphism (SNP) on the peptide coverage of a protein fragment. Because tryptic peptides are non-overlapping, each read position is covered by exactly one tryptic peptide. A SNP modifies a single tryptic peptide, but all positions around the SNP covered by the modified peptide are no longer covered (correctly) after matching (53 read positions on average). Because *k*-mers are overlapping, each read position is covered by k peptides, except at the ends of the protein fragments. A SNP modifies k peptides, but apart from reduced peptide coverage around the SNP, only a single codon is no longer covered (correctly) after matching. The redundancy of the overlapping *k*-mers therefore makes up for a reduced impact of SNPs compared to non-overlapping tryptic peptides, at the cost of larger data volumes that need to be processed

Because UMGAP uses exact matching for mapping peptides derived from a read onto peptides derived from the proteins in a reference database, read errors and natural variation usually have a lower impact on *k*-mers than on tryptic peptides. This is illustrated in Fig. 3 for a single nucleotide polymorphism (either as a read error or a natural variation).

### Peptide profiling

Fragmentation of all partial proteins found in a read yields a list of peptides (tryptic peptides or *k*-mers). Each peptide may have an associated consensus taxon that is looked up in an index structure. Overall, these lookups are the most time-consuming step in the pipeline, so performance is of utmost importance.

Upon each UniProt release, the Unipept team builds and publishes new indexes from tryptic peptides and 9-mers extracted from all UniProt proteins in the knowledgebase (available online at https://unipept.ugent.be/system/umgap/recent/tryptic.fstand https://unipept.ugent.be/system/umgap/recent/ninemer.fst). Each of these peptides is associated with the modified lowest common ancestor (LCA*) consensus taxon computed from the set of taxonomic annotations on all UniProt proteins that exactly match the peptide [14]. LCA* is the most specific taxon that does not contradict any taxon in the set, i.e., all taxa in the set must either be descendants or ancestors of the LCA* in the NCBI Taxonomy [29]. See the read profiling step for a detailed discussion on the LCA* algorithm introduced by UMGAP as a variation on the lowest common ancestor (LCA) algorithm.

UMGAP uses a finite state transducer [30] (FST) as its index structure to lookup the LCA* consensus taxon for each peptide extracted from a read. This index structure supports high-performance and parallel lookups, supports both fixed and variable length peptides, and has a relatively small memory footprint. The latter is important, given the large number of peptides extracted from UniProt. The index should be loaded in process memory, but UMGAP can also operate with an on-disk index and very little memory at the cost of performance.

**Fig. 4:**
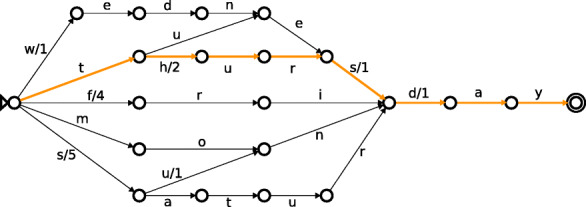
Finite state transducer mapping all weekdays to their index number (monday =1, tuesday =2,...). Integer labels are not shown on edges with zero weight. Adding weights along the path spelled by the letters of the word Thursday, from the initial state on the left (indicated by a triangle) to the final state on the right (indicated by a double circle), yields 2+1+1=4. So, Thursday is the fourth day in the week

The FST maps each peptide extracted from a UniProt protein to the NCBI Taxonomy Identifier (an integer) of the LCA* associated with the peptide. It is a flow graph whose edges are labeled with amino acids and integers. Peptides are matched by following the path of their amino acid sequence. The sum of the integers along this path corresponds to the identifier of the LCA*. Where tries are ordered tree data structures that take advantage of common prefixes to reduce the memory footprint, FSTs are even more compressed by taking both common prefixes and suffixes into account (Fig. 4).

**Fig. 5:**
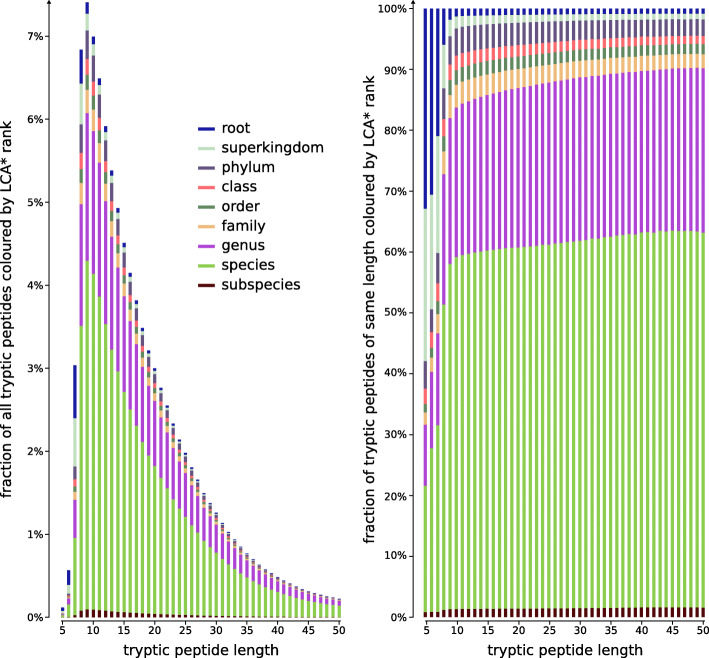
All tryptic peptides found in the UniprotKB (release 2020-04-22), classified by length and associated LCA* rank. Fractions of peptides (y-axis) across all peptide lengths (left) or per peptide length (right). Short tryptic peptides are more frequently associated with less specific ranks in the NCBI Taxonomy and therefore have a lower information content. Relative taxonomic information content (depth of LCA* rank in tree of life) is low for short peptides (length 8 and below). Because tryptic peptides of length *k* are a random sample of all *k*-mers, similar ratios and conclusions are expected should this analysis be repeated for all *k*-mers in UniprotKB across all lengths *k*

For UniProt release 2020-04, a 19.3 GiB FST-index maps all 1.2 billion tryptic peptides to their LCA* and a 132.9 GiB FST-index maps all 17 billion 9-mers to their LCA*. We also experimented with other k-mer lengths, but precision dropped significantly for *k*≤7 (Fig. 5) and the index size became too large for *k*≥10. The only viable options were *k*=8 and *k*=9, with the latter giving the best balance between index size and accuracy of read profiling.

### Peptide filtering

Protein fragmentation may yield false positives: peptides that do not occur in proteins encoded in the read. Most false positives are automatically filtered as they have no exact match with any UniProt protein. As a result, they cannot be associated with a taxon during peptide profiling. This is the case for most peptides from translations of wrong gene predictions or outside coding regions in a six-frame translation (Fig. 2). But peptide profiling itself may also yield false positive identifications: peptides associated with an inconsistent taxon, i.e., a taxon that is not the correct taxon or one of its ancestors in the NCBI Taxonomy tree. This could be the case for both true and false positive peptides from protein fragmentation. Peptide filtering aims at strongly reducing the number of false positive identifications, while keeping most true positives. UMGAP supports three kinds of filters.

**Short tryptic peptides** Analysis on UniProt proteins shows that short tryptic peptides are typically associated with highly unspecific LCA* consensus taxa, i.e., taxa at or close to the root of the NCBI Taxonomy tree [14] (Fig. 5). Because these peptides match proteins occurring across all domains of life, they do not provide a strong taxonomic signal that could be useful in downstream steps of the pipeline. In addition, by being short they often cause spurious matches during peptide profiling. UMGAP can skip very short tryptic peptides, e.g., having less than 6 amino acids.

**Low-frequency identifications** In the context of peptide profiling, true positive identifications should come from the same lineage in the NCBI Taxonomy tree, whereas false positives are randomly scattered across the tree. Since one read typically yields many peptides that may each have an associated taxon, identifications along the correct lineage are expected to occur with high frequency and false positives are expected to occur with low frequency. Therefore UMGAP can skip peptides associated with low-frequency identifications.

**Fig. 6:**
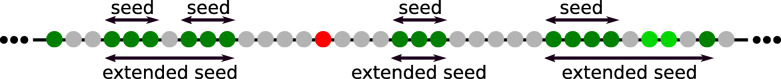
Seed-and-extend strategy for filtering false positive identifications after peptide profiling, with minimum seed size *s*=3 and maximum gap size *g*=1. Successive peptides fragmented from (partial) protein are shown as a sequence of dots. Green dots indicate correct identifications (true positives). Red dots indicate wrong identifications (false positives). Brightness of colored dots indicates depth in the taxonomic tree: dark colors indicate highly specific LCA* (species level) and light colors indicate nonspecific LCA*. Grey dots indicate peptides without an associated LCA*

**Seed-and-extend** The (partial) proteins in the read are typically fragmented into multiple peptides and it is expected that neighboring peptides have similar identifications (Fig. 2). It therefore seems natural to use a seed-and-extend approach to exploit this expected local conservation of identifications. Peptides are first scanned to find seeds: *s* or more successive peptides that are associated to the same LCA*. With increased minimum seed size *s*, the precision of the pipeline will increase, and its sensitivity will decrease. Each seed is then extended in both directions to neighboring seeds and individual peptides that are bridged by gaps (successive peptides with no associated LCA*) of at most *g* peptides. With increased maximum gap size *g*, the precision of the pipeline will decrease, and its sensitivity will increase. UMGAP can skip peptides that are excluded from extended seeds (Fig. 6).

### Read profiling

**Fig. 7:**
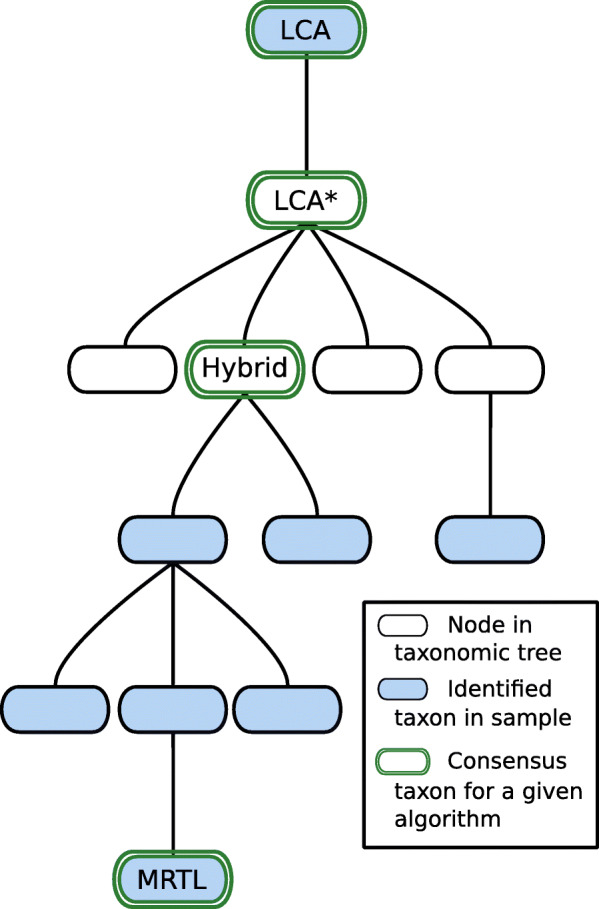
Lowest common ancestor (LCA), LCA*, hybrid _*f*_ and maximum root-to-leaf (MRTL) are four heuristics that determine a consensus taxon for a given list of taxa. All heuristics map the list of taxa onto the NCBI Taxonomy tree, that guides the heuristic towards a consensus taxon. MRTL is the most progressive heuristic and LCA* is the most conservative heuristic, so the MRTL consensus will be deeper in the tree than the LCA* consensus. The hybrid _*f*_ heuristic has a parameter *f* that can be tweaked to yield a result that is either more towards the LCA* consensus or the MRTL consensus

Previous steps of the pipeline result in a list of taxonomic identifications, derived from a (filtered) list of peptides extracted from the read. As the read comes from a single organism, it is natural to aggregate these individual identifications that rely on partial data into one global consensus identification. UMGAP supports three heuristics that infer a consensus taxon after mapping a frequency table of the individual identifications onto the NCBI Taxonomy tree (Fig. 7). They balance between providing a consensus taxon that is as far away from the root as possible and that allows for a good generalization. The first goal is progressive and leads to a very specific consensus but has to avoid overfitting. The second goal is conservative and takes into account the possibility of false positives among the individual identifications but has to avoid underfitting. All heuristics are implemented with two different data structures: a tree and a range minimum query [31]. Both implementations are functionally equivalent, but the latter gives a faster implementation of the MRTL heuristic because querying ancestry is supported in constant time.

**MRTL** Maximum root-to-leaf [11] is the most progressive heuristic. It identifies the consensus among a list of taxa as a taxon having the maximal number of ancestors in the list. Ties are broken arbitrarily. By definition, the consensus taxon is always included in the original list of taxa. This property does not hold for the other two heuristics, and shows that this heuristic might not necessarily be good at generalizing.

LCA* This the most conservative heuristic, though less conservative than a standard lowest common ancestor (LCA). For a given list of taxa, it identifies the consensus taxon as the most specific taxon in the tree that is either an ancestor or a descendant of each taxon in the list. This is the LCA of all taxa in the list, after we have first discarded all ancestors of at least one other taxon in the list. The latter is a measure against underfitting. Because the individual identifications are only based on partial data, it is expected that some identifications might be more specific than others. The LCA* heuristic is also used to compute the consensus taxon during peptide profiling.

**Hybrid**_***f***_ This heuristic has a parameter *f*∈[0,1] that allows to balance between being conservative or progressive: with *f*=1 this heuristic is the same as LCA* and with *f*=0 this heuristic is very close to MRTL (the same for most lists of taxa). LCA* can be implemented by starting at the root of the tree and repeatedly descending to the child node whose subtree contains all taxa in the list, until such a child no longer exists (i.e., the taxa in the list are distributed over multiple subtrees). In the latter case, the hybrid heuristic continues descending to the child node whose subtree contains most taxa from the list (with ties broken arbitrarily) if the fraction of the number of descendants in the child node over the number of descendants in the current node is larger than or equal to *f*.

### Summary and visualization

Previous steps assign a consensus taxon to each read (pair). The final step of the pipeline computes a frequency table of all identifications across the entire data set, with the option to filter low-frequency identifications. Another option is to report the frequency table at a user-specified taxonomic rank. Frequency tables are exported in CSV-format, enabling easy postprocessing.

**Fig. 8:**
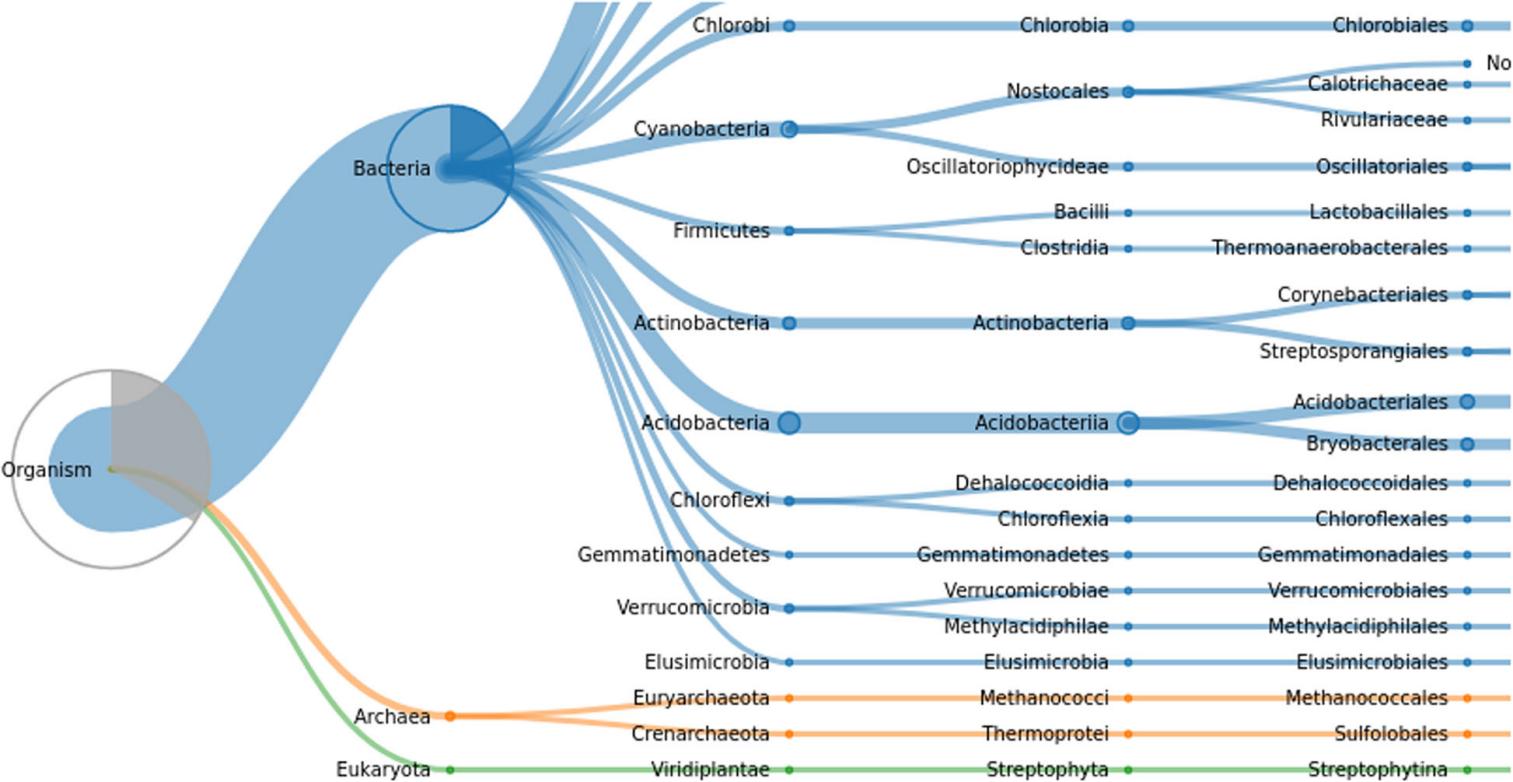
Taxonomic profiling by UMGAP as visualized by the Unipept Web API

To gain insight into environmental samples with a complex biodiversity, UMGAP also supports rendering taxonomic frequency tables as interactive visualizations (Fig. 8) that are automatically made available on a dedicated website. The online service hosting the visualizations also support shareable links (e.g. https://bl.ocks.org/5960ffd859fb17439d7975896f513bc3).

## Results

UMGAP implements multiple strategies for each step in the pipeline (Fig. 1), with some strategies also driven by user-specified parameters. Runtime, memory footprint and accuracy of UMGAP were benchmarked as a two-step process. Using some smaller data sets, we first measured and analysed performance metrics for a large number of relevant combinations of strategies and parameter settings. This broad exploration allowed us to investigate how different strategies/parameter settings led to different performance trade-offs. As a result, we defined six preconfigured pipelines with different performance trade-offs. Performance of these configurations has then been compared to a selection of state-of-the shotgun metagenomics taxonomic profiling tools in an established benchmark [19] that uses larger data sets.

Both the parameter sweep and the benchmark were executed on a 2.60GHz 16 core Intel *Ⓡ* Xeon *Ⓡ* CPU E5-2650 v2 CPU with 195GB RAM running Debian 9.8 (stretch).

### Parameter tuning

For protein translation we either used gene prediction or six-frame translation. FGSrs was used for gene prediction. In the protein fragmentation step we either used non-overlapping tryptic peptides or overlapping 9-mers. Tryptic peptides were filtered by length, with minimum length ranging from 5 to 10 amino acids and maximum length ranging from 45 to 50 amino acids. Peptide profiling was invariably done using LCA*. Low-frequency identifications were filtered with a minimum number of taxon hits per read that varied between 1 and 5, with a minimum of 1 hit effectively corresponding to no low-frequency identification filtering. For 9-mers, identifications were optionally also filtered using the seed-and-extend strategy with seed size *s* between 2 and 4, and gap size *g* between 0 to 4. Read profiling was done using either MRTL, LCA* or hybrid _*f*_, with parameter *f* either set to 0.25, 0.5 or 0.75.

All variation included in this parameter sweep resulted in 3900 different UMGAP configurations whose performance was evaluated for taxonomic profiling of two metagenome data sets simulated by Wood and Salzberg [11]. These data sets are referenced as the HiSeq metagenome and the MiSeq metagenome after the Illumina sequencing platforms whose read error models have been used for simulation. For each data set 1000 reads were simulated from 10 bacterial genomes, for a total of 10.000 reads per data set.

Accuracy of each UMGAP configuration was evaluated at the genus level by computing precision and sensitivity of the taxonomic profiling for each data set. Using the UMGAP snaptaxon tool and guided by the NCBI Taxonomy tree, more specific UMGAP predictions were mapped to the genus level because expected predictions were only known at the genus level for these data sets. True positives (TP) are reads assigned to the expected genus. False positives (FP) are reads assigned to a genus other than the expected genus. False negatives (FN) are reads that UMGAP could not assign at or below the genus level. As these data sets contain no invalid reads, there are no true negatives (TN).

**Fig. 9:**
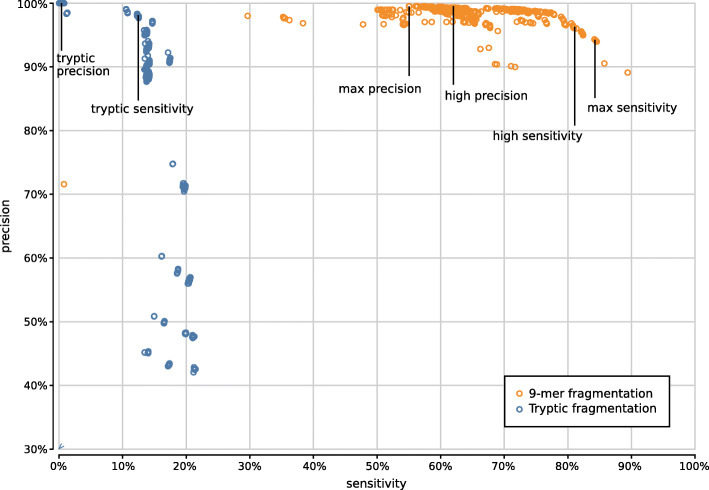
Precision and sensitivity of 3900 UMGAP configurations, with tryptic peptide configurations marked in blue and 9-mer configurations marked in orange. Six configurations are selected for further benchmarking

Figure 9 shows the precision and sensitivity of all 3900 UMGAP configurations tested. As expected, the protein fragmentation method has a major influence on sensitivity. The difference in precision is less pronounced at first glance. In general, 9-mer configurations (orange) have a higher sensitivity than tryptic configurations (blue), but they also have a higher runtime and memory footprint. To simplify further discussion, we will treat tryptic and 9-mer configurations separately in what follows.

### Tryptic configurations

If we look at the impact of protein translation on the accuracy of 1800 tryptic configurations (Supplementary Fig. 10), six-frame translation clearly improves the sensitivity of the tryptic configurations, at the cost of a steep drop in precision if spurious identifications resulting from incorrect translations are not properly filtered after peptide profiling. As it also yields much more work during the peptide profiling step, increasing execution time, combining six-frame translation with tryptic peptides proves less favorable.

**Fig. 10:**
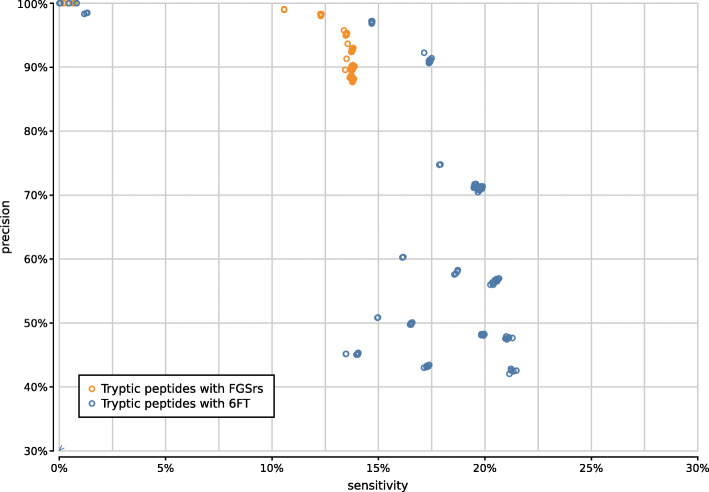
Precision and sensitivity of 1800 tryptic UMGAP configurations, classified per protein translation method: configurations using FGSrs gene predictions marked in orange and configurations using six-frame translations (6FT) marked in blue

Shorter peptides have a higher probability of random hits in a protein database. With tryptic peptides, it is therefore recommended to discard very short peptides. In general, we advise to only retain tryptic peptides with a length of at least 9 amino acids (Supplementary Fig. 11). We have also investigated the impact of a maximum peptide length cutoff on the accuracy of the predictions, but the effect is negligible except for a marginal gain in the speed of the pipeline.

**Fig. 11:**
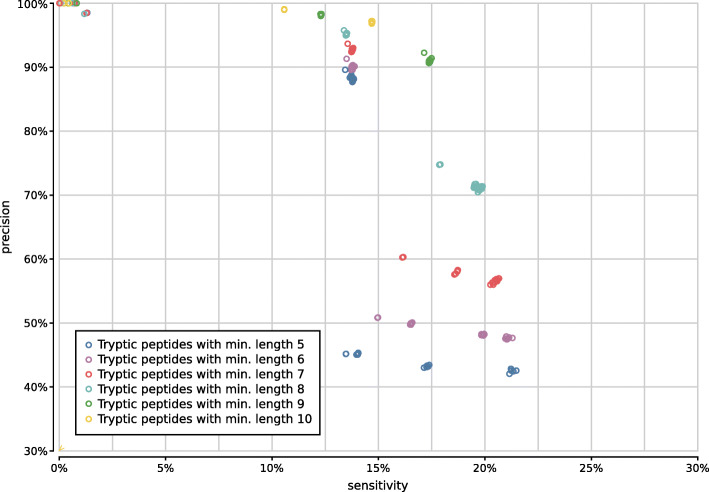
Precision and sensitivity of 1800 tryptic UMGAP configurations, classified per minimal peptide length

Tryptic configurations effectively profile only a limited number of peptides per read, such that filtering taxa after peptide profiling must be done carefully to avoid losing valuable information. Discarding taxa that have only been assigned to a single peptide guarantees high precision at the cost of a steep drop in sensitivity (Supplementary Fig. 12). This shows that tryptic configurations often profile reads based on a single peptide, increasing the risk of spurious predictions.

**Fig. 12:**
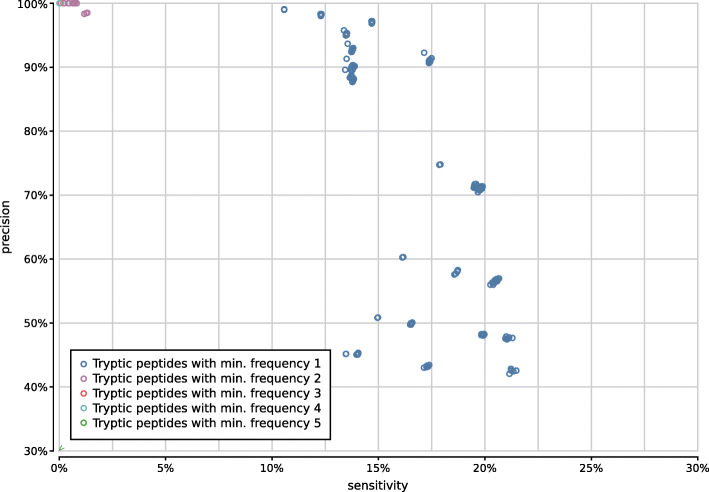
Precision and sensitivity of 1800 tryptic UMGAP configurations, classified according to filtering of low-frequency identifications

The choice of read profiling method has no significant impact on the performance of the pipelines, again because of the limited number of (reliable) peptides per read whose predictions need to be aggregated.

Based on the above observations we have selected two tryptic configurations with good accuracy trade-offs, either favoring higher precision or higher sensitivity (Fig. 9):

- *tryptic precision:* FGSrs, minimum peptide length 5, maximum peptide length 45, minimum 2 taxon hits, MRTL
- *tryptic sensitivity:* FGSrs, minimum peptide length 9, maximum peptide length 45, no rare taxon filtering, MRTL

### 9-mer configurations

When evaluating 800 UMGAP configurations that use 9-mer peptide fragmentation, the first observation is that seed-and-extend filtering has a positive effect on both precision and sensitivity (Supplementary Fig. 13). This filtering technique is not useful when working with tryptic peptides, but proves to be highly effective for discarding unreliable identifications after peptide profiling when working with 9-mers. As a result, we recommend to always apply seed-and-extend filtering in 9-mer configurations, and we will only focus on these configurations in any further analysis.

**Fig. 13:**
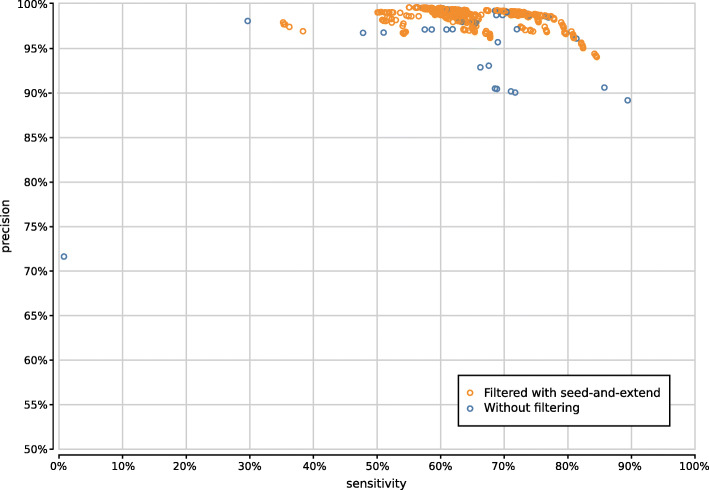
Precision and sensitivity of 800 UMGAP configurations using 9-mer peptide fragmentation, with configurations that don’t use seed-and-extend filtering marked in blue and configurations that do use seed-and-extend filtering marked in orange

With respect to protein translation method, the same observations concerning accuracy hold as with the tryptic configurations (Supplementary Fig. 14). The sensitivity gain that can be obtained with six-frame translation is more pronounced than with the tryptic configurations, which may make up for the extra work during the peptide profiling step. However, effective filtering of spurious identifications after peptide profiling is still needed in order to avoid poor precision.

**Fig. 14:**
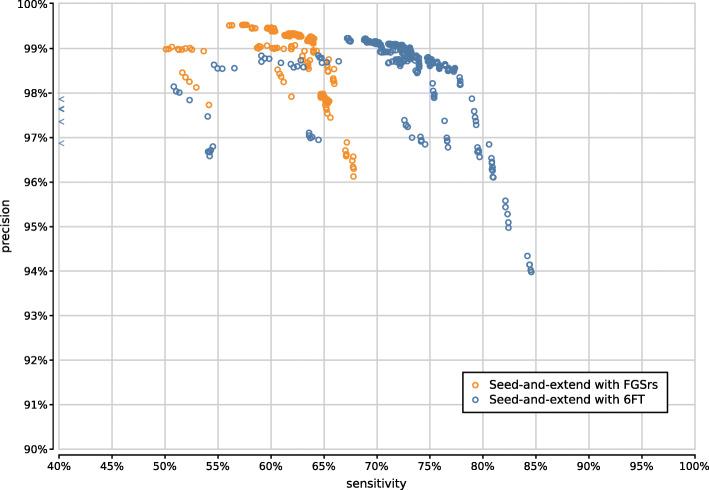
Precision and sensitivity of 750 UMGAP configurations using 9-mer peptide fragmentation in combination with seed-and-extend filtering, classified per protein translation method: configurations using FGSrs gene predictions marked in orange and configurations using six-frame translations (6FT) marked in blue

Gene prediction is best combined with minimum seed size *s*=2 for optimal sensitivity and with minimum seed size *s*=3 for the best trade-off between precision and sensitivity (Supplementary Fig. 15). In combination with six-frame translation, better trade-offs between precision and sensitivity are achieved with higher minimum seed size *s*. With gene prediction the low-frequency identifications filter has a higher impact than the chosen read profiling method, whereas the opposite is true for six-frame translation (Supplementary Figs. 16–19). In both cases, the maximum gap size *g* has no significant impact on the accuracy (data not shown).

**Fig. 15:**
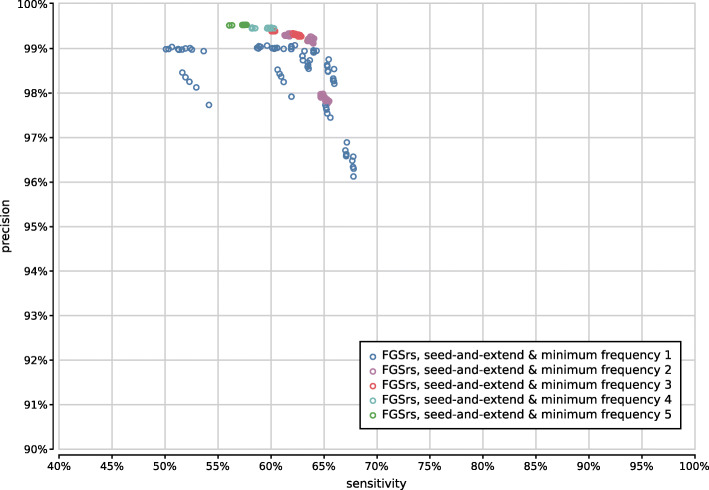
Precision and sensitivity of 750 UMGAP configurations using 9-mer peptide fragmentation in combination with seed-and-extend filtering, classified per minimal seed size and protein translation method. Gene prediction with FGS has been excluded

**Fig. 16:**
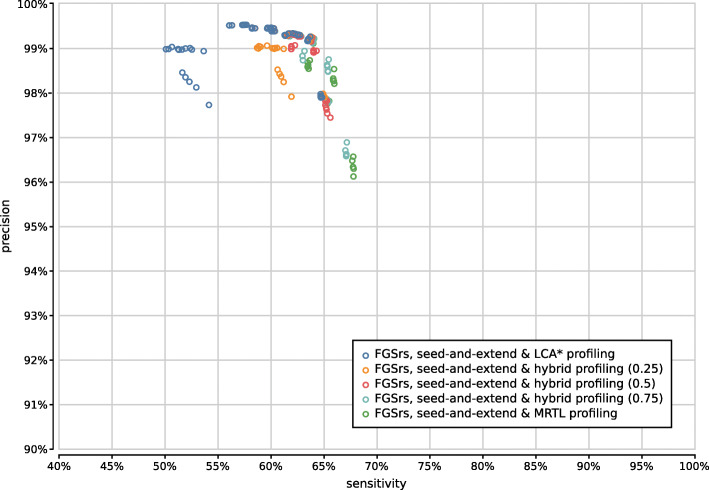
Precision and sensitivity of 375 UMGAP configurations using 9-mer peptide fragmentation, seed-and-extend filtering and gene prediction, classified per read profiling method

**Fig. 17:**
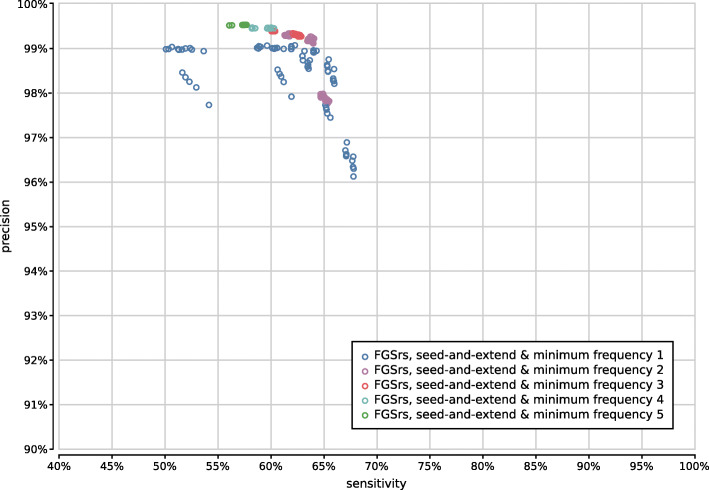
Precision and sensitivity of 375 UMGAP configurations using 9-mer peptide fragmentation, seed-and-extend filtering and gene prediction, classified per filtering of low-frequency identifications

**Fig. 18:**
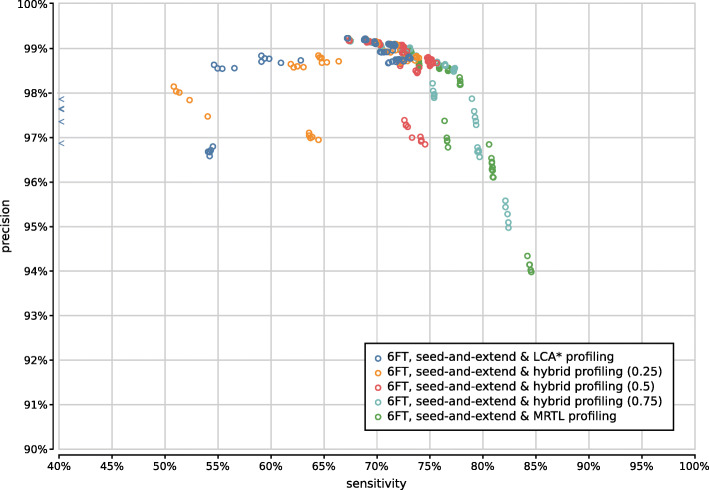
Precision and sensitivity of 375 UMGAP configurations using 9-mer peptide fragmentation, seed-and-extend filtering and six-frame translation, classified per read profiling method

**Fig. 19:**
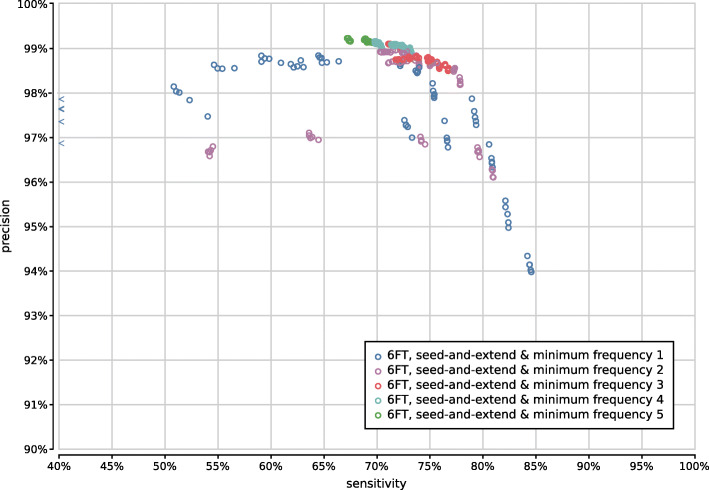
Precision and sensitivity of 375 UMGAP configurations using 9-mer peptide fragmentation, seed-and-extend filtering and six-frame translation, classified per filtering of low-frequency identifications

Based on the above observations we have selected four 9-mer configurations that represent different accuracy trade-offs. Ranging in optimization from precision to sensitivity they use the following UMGAP configurations (Fig. 9):

- *max precision* FGSrs, minimum 5 taxon hits, seed-and-extend with *s*=2 and *g*=2, hybrid _*f*_ with *f*=0.75
- *high precision* six-frame translation, minimum 4 taxon hits, seed-and-extend with *s*=3 and *g*=4, hybrid _*f*_ with *f*=0.5
- *high sensitivity* six-frame translation, no filtering on low-frequency identifications, seed-and-extend with *s*=3 and *g*=0, MRTL
- *max sensitivity* six-frame translation, no filtering on low-frequency identifications, seed-and-extend with *s*=2 and *g*=0, MRTL

### Benchmark

The six preconfigured UMGAP pipelines selected from the parameter sweep analysis were compared with the two best-performing shotgun metagenomics analysis tools found in the MetaBenchmark study [19] and with the popular Kaiju tool [32] that was published shortly after the initial benchmark. Kraken [11] and the newer Kraken 2 [33] were run with their default (preloaded) indexes and 16 threads. CLARK [34] was run with 20-mer indexes in full-mode. Because CLARK only supports identifications for a predefined taxonomic rank, we used indexes built from bacterial databases for the taxonomic ranks of phylum, genus, and species. Kaiju was run with its default index.

Our benchmark uses the same experimental setup as the MetaBenchmark study, including its use of two simulated metagenomes that differ in relative abundance of the individual phyla and that have three replicates each. The six data sets contain between 27 and 37 million read pairs simulated from both real, simulated, and shuffled genomes, with read length 100 and mean insert size 500 (standard deviation 25). All data sets contain 20% reads simulated from shuffled genomes that serve as a negative control and also contain reads simulated from genomes that were artificially diverged from a *Leptospira interrogans* reference genome to test the robustness of the tools against natural variation.

In addition to evaluating the accuracy of taxonomic profiling tools at the phylum and genus levels, we also evaluated their accuracy at the species level (Table 1, genus and phylum level are included in Supplementary Tables 2 and 3). Using the UMGAP snaptaxon tool and guided by the NCBI Taxonomy tree, predictions more specific than the taxonomic rank under evaluation were mapped to the taxonomic rank under evaluation. Predictions less specific than the taxonomic rank under evaluation were considered as no assignment to any taxon. Reads whose expected identification is less specific than the taxonomic rank under evaluation are ignored. True positives (TP) are non-shuffled reads assigned to the expected taxon. False positives (FP) are non-shuffled reads assigned to a taxon that differs from the expected taxon or shuffled reads assigned to a taxon. True negatives (TN) are shuffled reads not assigned to any taxon. False negatives (FN) are non-shuffled reads not assigned to any taxon.

**Table 1:** Table: MetaBenchmark performance metrics for ten metagenomics analysis tools sorted by precision. Average numbers for the six simulated data sets are given. Accuracy evaluated at the species level and reported as sensitivity, specificity, precision (positive predictive value), negative predictive value (NPV) and Matthew’s Correlation Coefficient (MCC). Index size reported for CLARK is the sum of the phylum (46.6GiB), genus (149.5GiB) and species (146.3GiB) indexes. Performance metrics at genus and phylum ranks can be found in Supplementary Tables 2 and 3

Tool	Precision	Sensitivity	Specificity	NPV	MCC	Run time	Index size
UMGAP tryptic prec.	99.70%	3.50%	99.96%	21.83%	8.62%	6.96 m	19.3 GB
Kraken	99.38%	81.34%	98.15%	59.21%	68.24%	210.86 m	198.7 GB
UMGAP max prec.	98.94%	45.87%	98.22%	33.35%	37.73%	16.47 m	132.9 GB
Kraken 2	98.15%	82.27%	94.64%	60.68%	67.27%	2.10 m	43.3 GB
UMGAP high prec.	98.11%	55.68%	96.21%	38.07%	43.33%	30.42 m	132.9 GB
Kaiju	98.02%	68.31%	95.21%	46.44%	53.15%	304.10 m	74.4 GB
UMGAP tryptic sens.	96.07%	18.03%	97.36%	24.88%	17.95%	6.10 m	19.3 GB
UMGAP high sens.	92.55%	66.70%	84.12%	46.04%	44.28%	30.32 m	132.9 GB
UMGAP max sens.	80.78%	77.73%	63.34%	58.94%	40.39%	31.12 m	132.9 GB
CLARK	71.41%	100.00%	27.87%	100.0%	44.61%	20.54 m	342.3 GB

**Table 2:** The MetaBenchmark performance metrics for ten metagenomics analysis tools at genus level

Tool	Precision	Sensitivity	Specificity	NPV	MCC	Run time
UMGAP tryptic precision	99.87%	5.19%	99.97%	20.97%	10.37%	7.29 min
Kraken	99.59%	87.78%	98.60%	67.39%	76.07%	227.99 min
UMGAP max precision	99.56%	63.49%	98.89%	40.86%	50.22%	17.28 min
Kraken 2	98.64%	89.10%	95.40%	70.02%	76.17%	2.15 min
UMGAP high precision	99.08%	73.24%	97.40%	48.63%	58.05%	31.59 min
Kaiju	99.04%	80.49%	97.02%	56.51%	65.62%	314.55 min
UMGAP tryptic sensitivity	98.14%	25.62%	98.12%	25.36%	23.62%	6.37 min
UMGAP high sensitivity	93.78%	85.81%	82.38%	65.20%	63.42%	31.83 min
UMGAP max sensitivity	81.58%	100.00%	54.58%	100.00%	66.72%	32.41 min
CLARK	70.76%	100.00%	8.97%	100.00%	25.19%	22.27 min

**Table 3:** The MetaBenchmark performance metrics for ten metagenomics analysis tools at phylum level

Tool	Precision	Sensitivity	Specificity	NPV	MCC	Run time
UMGAP tryptic precision	100.00%	6.10%	100.00%	21.03%	11.32%	7.27 min
Kraken	99.98%	88.95%	99.92%	69.35%	78.49%	223.16 min
UMGAP max precision	99.99%	73.50%	99.96%	48.55%	59.71%	17.08 min
Kraken 2	99.99%	91.08%	99.97%	73.70%	81.91%	2.14 min
UMGAP high precision	99.96%	83.02%	99.88%	59.56%	70.25%	31.74 min
Kaiju	99.96%	84.32%	99.88%	61.47%	71.92%	317.04 min
UMGAP tryptic sensitivity	99.69%	30.77%	99.62%	26.55%	28.24%	6.40 min
UMGAP high sensitivity	94.72%	93.35%	83.48%	79.83%	75.68%	31.85 min
UMGAP max sensitivity	81.50%	100.00%	37.88%	100.00%	55.56%	32.52 min
CLARK	74.28%	100.00%	7.87%	100.00%	24.17%	22.39 min

**Fig. 20:**
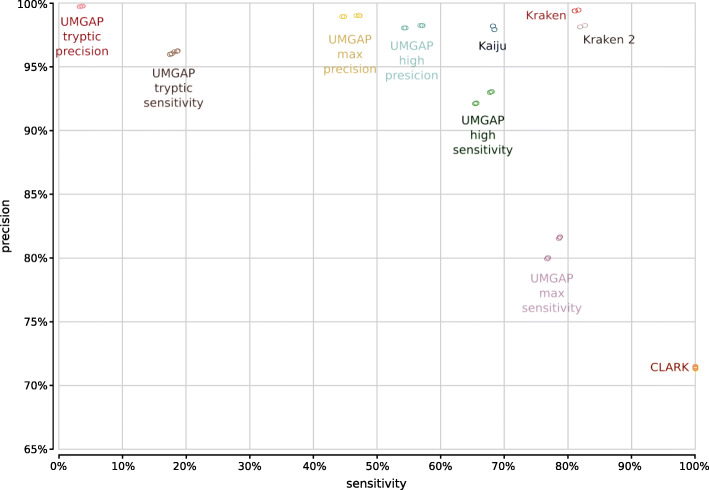
Precision and sensitivity evaluated at the species level for ten metagenomics analysis tools and the two simulated metagenomes of the MetaBenchmark. Dots indicating the accuracy metrics for the three replicates of each simulated metagenome are on top of each other, since each replicate was generated for identical proportions of phyla

In terms of precision the UMGAP tryptic precision configuration marginally surpasses Kraken, with the UMGAP max/high precision configurations, Kraken 2, and Kaiju also showing very high precision rates (Fig. 20, Table 1). As expected, the UMGAP pipelines have a lower sensitivity than the other metagenomics pipelines because a priori no taxa are assigned to reads that have no or only short overlap with protein coding regions. This benchmark again underscores the difference in sensitivity between the tryptic and 9-mer configurations of UMGAP. Also take into account that precision is a more important accuracy metric than sensitivity for most biological applications, especially with deeply sequenced samples. In terms of speed Kraken 2 is the best-performing tool, with UMGAP’s tryptic configurations following in close range. Clark and the UMGAP 9-mer configurations are still considerably faster than Kraken and Kaiju.

### In-depth analysis

We would like to stress that UMGAP does not require setting a specific target taxonomic rank prior to processing a dataset. Instead, UMGAP automatically decides for each read at which taxonomic rank a reliable identification can be made, taking into account that deeper ranks are more informative. As a result, UMGAP automatically balances between optimal information content (specificity) and reliability (sensitivity), with different settings of the pipeline resulting in different trade-offs. Mapping UMGAP identifications to a specific rank is only a post-processing step we have done (using UMGAP’s snaprank tool) to comply with the experimental setup of the MetaBenchmark.

Taking advantage of the dynamic taxonomic rank assignment and the fact that UMGAP reports taxonomic profiles for each individual read, we performed a more in-depth analysis to investigate two questions not elucidated by the MetaBenchmark: *i*) how specific are read profilings that are correctly identified but above the species level and *ii*) can we observe any trends that explain wrong identifications? The analysis still uses species as the target rank, but in a less stringent way compared to the MetaBenchmark.

We performed the analysis using the UMGAP high precision pipeline. Accuracy metrics are reported per operational taxonomic unit (OTU), i.e. all (paired-end) reads are grouped per OTU from which they were extracted/generated. Results are reported in separate tables for one of the small datasets we used for parameter tuning (10 OTUs) and one of the large MetaBenchmark datasets (1105 OTUs), split into real (963), simulated (32) and shuffled (110) OTUs (Additional file 1). In what follows, we discuss some general observations from the in-depth analysis and illustrate them with specific use cases.

In addition to correct identifications at the species level (the typical rank of the expected identification derived from the benchmark data), UMGAP also identifies (paired-end) reads correctly but at less specific taxonomic ranks (genus level and above) as can be seen from the second column in the reported tables. For some OTUs, UMGAP yields highly specific identifications, i.e. most of the OTU reads are correctly identified at the species level (species entry marked in bold in the second column). For other OTUs, UMGAP yields less specific identifications, i.e. most of the OTU reads are correctly identified at the genus level or above (species entry not marked in bold in the second column). One particular reason for the latter are misidentifications in the reference databases, especially because UMGAP uses broad spectrum indexes built from the entire UniProt Knowledgebase. Using the LCA* algorithm to compute the taxonomic profiling of a single peptide might correct for some misidentifications in UniProt, but definitely not all. For example, misidentifying UniProt proteins from one strain to another species of the same genus might cause that the taxonomic profiles of most peptides of the two species (the correct and wrong identification) resolve at the genus level and no longer at the species level. For some species groups it is also well known that they are extremely hard to differentiate or that there’s even debate whether it is natural to keep them taxonomically separate (as the *Bacillus cereus* vs. *Bacillus anthracis* case, with multiple OTUs included in the MetaBenchmark). Again, problematic identification in these species groups also increases the possibility of misidentifications in UniProt.

Wrong identifications exceeding 2% of the total number of (paired-end) reads (marked in bold in the third column) are rare and might indicate issues with the expected identification in the benchmark dataset. For example, in the smaller dataset used for parameter tuning of UMGAP, none of the reads for the OTU identified as *Aeromonas hydrophila* SSU are identified by UMGAP as the species *A. hydrophila*, whereas 10% of the reads are identified as the species *A. dhakensis*. If we look into the history of the classification of these species, *Aeromonas hydrophila* subsp. *dhakensis* was established in 2002 as a new subspecies of *A. hydrophila* [35], whereas in 2015 it was reclassified as a separate species *A. dhakensis* by Beaz-Hidalgo et al. [36]. Grim et al. [37] reclassified the virulent *A. hydrophila* SSU strain isolated from a patient with diarrhea in the Philippines as *A. dhakensis* SSU, showing that in this case UMGAP actually comes up with a correct identification and instead the identification in the benchmark should have been updated. Where Chen et al. [38] mention that *A. dhakensis* is often misidentified as *A. hydrophila*, *A. veronii*, or *A. caviae* by commercial phenotypic tests in the clinical laboratory, our analysis shows that UMGAP is indeed able to correctly identify reads in a metagenomics dataset to *A. dhakensis*. Apart from the power of the identification pipeline used by UMGAP, this case study also reminds us that taxonomy is not a constant and underscores the importance of using broad spectrum indexes that are constantly updated.

Some OTUs are only identified to the genus rank (or above) in the MetaBenchmark, whereas UMGAP consistently identifies many of the corresponding (paired-end) reads to one particular species of the same genus. An example is *Methylovorus* sp. MP688 in the large dataset, where UMGAP assigns 3087 of the 5556 reads (55%) to the species *Methylovorus glucosotrophus*. The correctness of this observation is confirmed by Doronina et al. [39] based on phylogenetic analysis using 16S rRNA gene sequences and mxaF amino acid sequences, five years after the complete genome sequence of the strain MP688 has been deposited [40] as *Methylovorus* sp., a name that has never been updated in the public sequence databases. An important factor in this case, is the fact that the complete genome sequence *Methylovorus glucosetrophus* strain SIP3-4 has been deposited in the public sequence database [41], whose proteome is also available in UniProt.

Almost all shuffled reads in the large dataset are mapped to the root of the NCBI Taxonomy, which corresponds to no identification at all. This reflects the robustness of UMGAP against spurious identifications. The large dataset contains reads simulated from genomes that were artificially diverged from a *Leptospira interrogans* reference genome (AE016823). In total, reads for 32 OTUs were generated from 8 simulated genomes with either little, medium, mixed or high divergence. Since these genomes are not random but simulated using an evolutionary model, it is expected that the derived reads could be assigned to the correct clade. Of the OTUs generated from simulated genomes with little divergence, we consistently observe that 35% of the reads are correctly identified to the species level and 40% to the genus level. Of the OTUs generated from simulated genomes with medium divergence, only 1-2% of the reads are correctly identified at the species level and 5% at the genus level. Of the OTUs generated from simulated genomes with high divergence, almost no reads could be identified. OTUs generated from simulated genomes with mixed divergence either follow the pattern of genomes with little divergence or the genomes with medium divergence.

## Discussion

The six predefined pipelines come bundled with UMGAP a separate POSIX shell script, which makes them the primary way to run UMGAP on metagenomics data sets. This section details the setup of the reference database and optional external tools, followed by a short case study using a preconfigured pipeline. Instructions on the configuration of your own pipeline and the details of all UMGAP tools are available on the Unipept website (https://unipept.ugent.be/umgap).

After downloading and installing UMGAP as described in the README (https://github.com/unipept/umgap), run the umgap-setup.sh script to interactively download the relevant databases. Pass -f /opt/FragGeneScan to link the installation directory of FragGeneScan or put the FGSrs executable in your PATH.

As an example, we will profile 100 paired-end reads sampled from the benchmark dataset [19] (supplementary files 2 and 3) using a tryptic peptide index. If these files are saved as A1.fq and A2.fq, the following command will profile the reads:


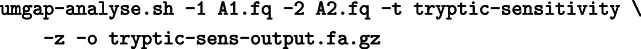


The two paired-end files are passed using the options -1 and -2. Only use the option -1 when processing single-end reads. Both files can be GZIP-compressed and will be automatically decompressed by UMGAP. The option -t is used to select one of the preconfigured pipelines. The flag -z demands UMGAP to compress the output, which is written to the file indicated with the option -o. The output file contains a single taxonomic profile for each read. For the sample files it should start with:


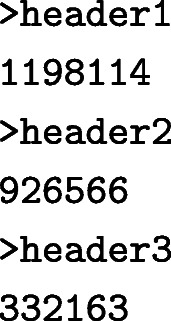


The umgap-visualize.sh script can be used to summarize and visualize the results. This script can create importable CSV frequency tables and interactive visualizations that are either stored locally or hosted online. This is illustrated in the following shell session. Figure 8 contains a screenshot of one of the interactive vizualizations.


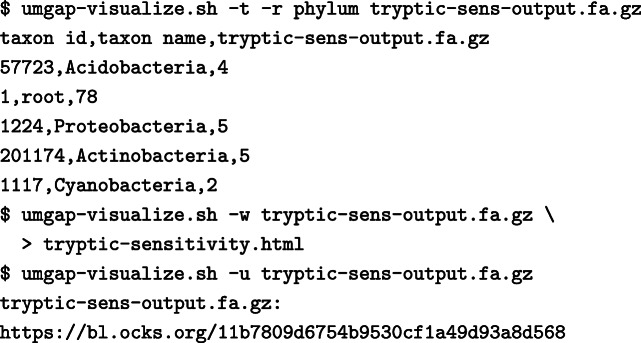


The flags -t, -w and -u select the output mode. The option -r allows setting the taxonomic rank for the output.

## Conclusions

UMGAP’s protein space detour for taxonomic profiling makes it competitive with state-of-the-art shotgun metagenomics tools. Despite our design choices of an extra protein translation step, a broad spectrum index that can identify both archaea, bacteria, eukaryotes and viruses, and a highly configurable non-monolithic design, UMGAP achieves low runtime, manageable memory footprint and high accuracy. Integrating the command line tool with the interactive Unipept visualizations [17] also allows exploration and comparison of complex communities. As such the pipeline has already been used to study the biodiversity in the rhizosphere [42].

As a next step, we want to further explore how the protein translation detour can be used to infer the functional capacity of an environmental sample from its metagenome, which is more challenging than inferring biodiversity. Again, Unipept’s function analysis pipeline for metaproteomes could be used as a potential starting point. In addition, both the biodiversity and the functional capacity of a sample could also be derived from its metatranscriptome, which could be analysed using pipelines similar to UMGAP but with an adjusted protein translation step.

## Availability and requirements

**Project name:** UMGAP

**Project home page:** https://github.com/unipept/umgap

**Archived release:** https://github.com/unipept/umgap/releases/tag/v1.0.0

**Operating system(s):** GNU/Linux, MacOS

**Programming language:** Rust

**Other requirements:** Rust edition 2018 or higher

**License:** MIT

**Any restrictions to use by non-academics:** None

## Supplementary Information

**Additional file 1** Table of misidentifications. This PDF file contains several multi-page tables showing the misidentifications in the benchmark data sets.

**Additional file 2** A1.fq.

**Additional file 3** A2.fq.

## Data Availability

The parameter tuning dataset can be found as the accuracy dataset at the Kraken website https://ccb.jhu.edu/software/kraken/. The benchmark dataset can be found at UC Bioinformatics http://web.archive.org/web/20170602055550/http://www.ucbioinformatics.org/metabenchmark.html. The latter are also rehosted at Unipept (https://unipept.ugent.be/system/umgap/metabenchmark/setA1_1.fq.gz, setA1_2.fq.gz, setA2_1.fq.gz, setA2_2.fq.gz, setA3_1.fq.gz, setA3_2.fq.gz, setB1_1.fq.gz, setB1_2.fq.gz, setB2_1.fq.gz, setB2_2.fq.gz, setB3_1.fq.gz, and setB3_2.fq.gz) to ensure availability. The created index files are dated by UniprotKB release day and are found at https://unipept.ugent.be/system/umgap/recent/ninemer.fst, tryptic.fst and taxons.tsv.

## Abbreviations

FGS: FragGeneScan
FGS+: FragGeneScan+
FGSrs: FragGeneScanRs
LCA: Lowest common ancestor
FST: Finite state transducer
MRTL: Maximum root-to-leaf path
OTU: Operational taxonomic unit.
