# Supplementary material for: UMGAP: the Unipept MetaGenomics Analysis Pipeline
Source: BMC Genomics. 2022 Jun 10;23:433. doi: 10.1186/s12864-022-08542-4 (PMC9188040; doi:10.1186/s12864-022-08542-4)
Supplement: Supplementary file 1 — Additional file 1 Table of misidentifications. This PDF file contains several multi-page tables showing the misidentifications in the benchmark data sets. [file 12864_2022_8542_MOESM1_ESM.pdf]

Analysis of misclassifications per Operational Taxonomic Unit

This file contains an in-depth analysis in which we investigate the accuracy of the taxonomic profilings of UMGAP. This analysis uses a single setting of UMGAP (high-precision) and reports accuracy metrics per operational taxonomic unit (OTU), i.e. all (paired-end) reads are grouped per OTU from which they were extracted/generated. We still use species as the target rank, but in a less stringent way compared to the MetaBenchmark.

We performed the analysis on both datasets, used in the manuscript for parameter tuning (smaller dataset; 10 OTUs) and benchmarking (larger dataset; 1105 OTUs). The results are reported in four tables:

- Parameter Tuning dataset: results for the 10 OTUs in the small dataset. (page 3)
- Benchmark dataset - real reads: results for the 963 OTUs in the large dataset, excluding the evolutionary simulated and randomized OTUs in the large dataset. (page 6)
- Benchmark dataset - shuffled reads: results for the 110 randomized OTUs in the large dataset. (page 237)
- Benchmark dataset - simulated divergence reads: results for the 32 evolutionary simulated OTUs in the large dataset. (page 265)

For each OTU, the tables report:

- (first column) OTU ID (a unique identifier) as specified in the benchmark dataset; this may include an accession number of the sequence record where the read was extracted from.
- (first column) name and taxon ID of the OTU as included in the benchmark and the name, taxon ID and rank of the expected identification; these taxa might differ for two reasons:
  - The OTU from the benchmark does not necessarily have a rank in the NCBI Taxonomy. It is typically an ID for a specific strain or clone. This taxon is mapped to its most specific ancestor with a rank in the NCBI Taxonomy.
  - After the previous step, some OTUs are mapped to a rank that is more specific (e.g. subspecies) than the target rank of the benchmark (in casu the species rank). These OTUs are mapped to the target rank of the benchmark.

  All taxon IDs (taxid) included in the report are identifiers assigned by the NCBI Taxonomy [@federhen].
- (first column) total number of (paired-end) reads in the dataset for the OTU.
- (first column) absolute and relative number of (paired-end) reads for the OTU that could be identified; UMGAP yields not identification if no peptides could be extracted from the read (e.g. because the read contains no coding regions or no coding regions could be predicted on the read).
- (second column) absolute and relative number of (paired-end) reads with a correct taxonomic identification (according to the expected identification) per taxonomic rank at or above the rank of the expected identification (in casu never below the species rank); UMGAP profiles a read to the root of the NCBI Taxonomy if not enough taxonomic profilings could be made for individual peptides extracted from the read based on the filtering options; the taxonomic rank with the highest number of correct identifications is marked in bold.
- (third column) absolute and relative number of (paired-end) reads with a wrong taxonomic identification (according to the expected identification) per taxon as identified by UMGAP; UMGAP identifications for ranks that are more specific than the rank of the expected identification are also reported as wrong identifications while technically they are not necessarily wrong (that status cannot be derived from the benchmark data), as this might indicate where UMGAP consistently identifies reads of the OTU to a more specific taxon than what

is known about the OTU in the benchmark; identifications are sorted by decreasing number of reads and truncated after 7 OTUs; wrong identifications exceeding 2% of the total number of reads are marked in bold and indicate identifications that consistently differ from the expected identification.

The most striking observations from this more in-depth analysis:

- In addition to correct identifications at the species level (the typical rank of the expected identification derived from the benchmark data), UMGAP also identifies (paired-end) reads correctly but at less specific taxonomic ranks (genus level and above) as can be seen from the second column in the reports. For some OTUs, UMGAP yields highly specific identifications, i.e. most of the OTU reads are correctly identified at the species level (species entry marked in bold in the second column). For other OTUs, UMGAP yields less specific identifications, i.e. most of the OTU reads are correctly identified at the genus level or above (species entry not marked in bold in the second column). One particular reason for the latter are misidentifications in the reference databases, especially because UMGAP uses broad spectrum indexes built from the entire UniProt Knowledgebase. Using the LCA* algorithm to compute the taxonomic profiling of a single peptide might correct for some misidentifications in UniProt, but definitely not all. For example, misidentifying UniProt proteins from one strain to another species of the same genus, might cause that the taxonomic profiles of most peptides of the two species (the correct and wrong identification) resolve at the genus level and no longer at the species level. For some species groups it is also well known that they are extremely hard to differentiate or that there's even debate whether it is natural to keep them taxonomically separate (as the *Bacillus cereus* vs. *Bacillus anthracis* case, with multiple OTUs included in the MetaBenchmark). Again, problematic identification in these species groups also increases the possibility of misidentifications in UniProt.
- Wrong identifications exceeding 2% of the total number of (paired-end) reads (marked in bold in the third column) are rare and might indicate issues with the expected identification in the benchmark dataset. For example, in the smaller dataset used for parameter tuning of UMGAP, none of the reads for the OTU identified as *Aeromonas hydrophila* SSU are identified by UMGAP as the species *A. hydrophila*, whereas 10% of the reads are identified as the species *A. dhakensis*. If we look into the history of the classification of these species, *Aeromonas hydrophila* subsp. *dhakensis* was established in 2002 as a new subspecies of *A. hydrophila* [@huys], whereas in 2015 it was reclassified as a separate species *A. dhakensis* by @beaz. @grim reclassified the virulent *A. hydrophila* SSU strain isolated from a patient with diarrhea in the Philippines as *A. dhakensis* SSU, showing that in this case UMGAP actually comes up with a correct identification and instead the identification in the benchmark should have been updated. Where @polin mention that A. dhakensis is often misidentified as *A. hydrophila*, *A. veronii*, or *A. caviae* by commercial phenotypic tests in the clinical laboratory, our analysis shows that UMGAP is indeed able to correctly identify reads in a metagenomics dataset to *A. dhakensis*. Apart from the power of the identification pipeline used by UMGAP, this case study also reminds us that taxonomy is not a constant and underscores the importance of using broad spectrum indexes that are constantly updated.
- Some OTUs are only identified to the genus rank (or above) in the MetaBenchmark, whereas UMGAP consistently identifies many of the corresponding (paired-end) reads to one particular species of the same genus. An example is *Methylovorus* sp. MP688 in the benchmark dataset, where UMGAP assigns 3087 of the 5556 reads (55%) to the species *Methylovorus glucosotrophus*. The correctness of this observation is confirmed by @doronina based on phylogenetic analysis using 16S rRNA gene sequences and mxaF amino acid sequences, five years after the complete genome sequence of the strain MP688 has been deposited [@xiong] as *Methylovorus* sp., a name that has never been updated in the public sequence databases. An important factor in this case, is the fact that the complete genome sequence *Methylovorus glucosetrophus* strain SIP3-4 has been deposited in the public sequence database [@lapidus], whose proteome is also available in Uniprot.
- Almost all shuffled reads in the large dataset are mapped to the root of the NCBI Taxonomy, which corresponds to no identification at all. This reflects the robustness of UMGAP against spurious identifications.
- The large dataset contains reads simulated from genomes that were artificially diverged from a *Leptospira interrogans* reference genome (AE016823). In total, reads for 32 OTUs were generated from 8 simulated genomes with either little, medium, mixed or high divergence. Since these genomes are not random but simulated using an evolutionary model, it is expected that the derived reads could be assigned to the correct clade. Of the OTUs generated from simulated genomes with little divergence, we consistently observe that 35% of the reads are correctly identified to the species level and 40% to the genus level. Of the OTUs generated from simulated genomes with medium divergence, only 1-2% of the reads are correctly identified at the species level and 5% at the genus level. Of the OTUs generated from simulated genomes with high divergence, almost no reads could be identified. OTUs generated from simulated genomes with mixed divergence either follow the pattern of genomes with little divergence or the genomes with medium divergence.

References

Parameter Tuning dataset

Operational Taxonomic Unit (OTU)	Correct identifications	Wrong or overspecific identifications at species rank
Benchmark OTU ID: `A_hydrophila_HiSeq`<br>OTU taxon: Aeromonas hydrophila SSU [taxid 1073377]<br>Expected: Aeromonas hydrophila [taxid 644] (species)<br>Number of reads: 1000<br>Number of identified reads: 876 (87.6%)	• species: 0 (0.0%)<br>• **genus: 658 (65.8%)**<br>• family: 3 (0.3%)<br>• order: 0 (0.0%)<br>• class: 69 (6.9%)<br>• phylum: 29 (2.9%)<br>• superkingdom: 4 (0.4%)<br>• root: 113 (11.3%)	• **Aeromonas dhakensis [taxid 196024]: 103 (10.3%)**<br>• Aeromonas salmonicida [taxid 645]: 3 (0.3%)<br>• Aeromonas lusitana [taxid 931529]: 1 (0.1%)<br>• Azospirillum brasilense [taxid 192]: 1 (0.1%)<br>• Aeromonas molluscorum [taxid 271417]: 1 (0.1%)<br>• Aeromonas caviae [taxid 648]: 1 (0.1%)
Benchmark OTU ID: `B_cereus_HiSeq`<br>OTU taxon: Bacillus cereus VD118 [taxid 1053231]<br>Expected: Bacillus cereus [taxid 1396] (species)<br>Number of reads: 1000<br>Number of identified reads: 877 (87.7%)	• species: 47 (4.7%)<br>• **genus: 702 (70.2%)**<br>• family: 13 (1.3%)<br>• order: 11 (1.1%)<br>• class: 2 (0.2%)<br>• phylum: 4 (0.4%)<br>• superkingdom: 14 (1.4%)<br>• root: 84 (8.4%)	• Bacillus mycoides [taxid 1405]: 5 (0.5%)<br>• Bacillus cytotoxicus [taxid 580165]: 2 (0.2%)<br>• Bacillus anthracis [taxid 1392]: 1 (0.1%)<br>• Persephonella marina [taxid 309805]: 1 (0.1%)<br>• Bacillus thuringiensis [taxid 1428]: 1 (0.1%)<br>• Bacillus pseudomycoides [taxid 64104]: 1 (0.1%)<br>• Methylobacterium platani [taxid 427683]: 1 (0.1%)<br>• Bacillus wiedmannii [taxid 1890302]: 1 (0.1%)
Benchmark OTU ID: `B_fragilis_HiSeq`<br>OTU taxon: Bacteroides fragilis HMW 615 [taxid 1073387]<br>Expected: Bacteroides fragilis [taxid 817] (species)<br>Number of reads: 1000<br>Number of identified reads: 916 (91.6%)	• **species: 405 (40.5%)**<br>• genus: 249 (24.9%)<br>• family: 0 (0.0%)<br>• order: 100 (10.0%)<br>• class: 1 (0.1%)<br>• phylum: 7 (0.7%)<br>• superkingdom: 21 (2.1%)<br>• root: 133 (13.3%)	• Prevotella copri [taxid 165179]: 1 (0.1%)<br>• Bacteroides caccae [taxid 47678]: 1 (0.1%)<br>• Enterococcus faecalis [taxid 1351]: 1 (0.1%)<br>• Bacteroides uniformis [taxid 820]: 1 (0.1%)
Benchmark OTU ID: `M_abscessus_HiSeq`<br>OTU taxon: Mycobacteroides abscessus 6G-0125-R [taxid 1001740]<br>Expected: Mycobacteroides abscessus [taxid 36809] (species)<br>Number of reads: 1000<br>Number of identified reads: 864 (86.4%)	• **species: 296 (29.6%)**<br>• genus: 286 (28.6%)<br>• family: 71 (7.1%)<br>• order: 35 (3.5%)<br>• class: 31 (3.1%)<br>• phylum: 1 (0.1%)<br>• superkingdom: 23 (2.3%)<br>• root: 121 (12.1%)	• Mycobacterium tuberculosis [taxid 1773]: 1 (0.1%)<br>• Mycolicibacterium rhodesiae [taxid 36814]: 1 (0.1%)<br>• Actinoplanes xinjiangensis [taxid 512350]: 1 (0.1%)
Benchmark OTU ID: `P_fermentans_HiSeq`<br>OTU taxon: Pelosinus fermentans A11 [taxid 1149860]<br>Expected: Pelosinus fermentans [taxid 365349] (species)<br>Number of reads: 1000<br>Number of identified reads: 813 (81.3%)	• **species: 442 (44.2%)**<br>• genus: 141 (14.1%)<br>• family: 14 (1.4%)<br>• order: 2 (0.2%)<br>• class: 5 (0.5%)<br>• phylum: 35 (3.5%)<br>• superkingdom: 28 (2.8%)<br>• root: 145 (14.5%)	• Lacrimispora celerecrescens [taxid 29354]: 5 (0.5%)<br>• Pelosinus propionicus [taxid 380084]: 2 (0.2%)<br>• Eumeta japonica [taxid 151549]: 1 (0.1%)<br>• Sporomusa acidovorans [taxid 112900]: 1 (0.1%)

Operational Taxonomic Unit (OTU)	Correct identifications	Wrong or overspecific identifications at species rank
Benchmark OTU ID: `R_sphaeroides_HiSeq`<br>OTU taxon: Rhodobacter sphaeroides 2.4.1 [taxid 272943]<br>Expected: Rhodobacter sphaeroides [taxid 1063] (species)<br>Number of reads: 1000<br>Number of identified reads: 638 (63.8%)	• species: 84 (8.4%)<br>• **genus: 279 (27.9%)**<br>• family: 98 (9.8%)<br>• order: 1 (0.1%)<br>• class: 39 (3.9%)<br>• phylum: 9 (0.9%)<br>• superkingdom: 17 (1.7%)<br>• root: 109 (10.9%)	• Lupinus albus [taxid 3870]: 1 (0.1%)<br>• Rhodobacter johrii [taxid 445629]: 1 (0.1%)<br>• Pararhodospirillum photometricum [taxid 1084]: 1 (0.1%)
Benchmark OTU ID: `S_aureus_HiSeq`<br>OTU taxon: Staphylococcus aureus M0927 [taxid 1213734]<br>Expected: Staphylococcus aureus [taxid 1280] (species)<br>Number of reads: 1000<br>Number of identified reads: 878 (87.8%)	• **species: 503 (50.3%)**<br>• genus: 217 (21.7%)<br>• family: 19 (1.9%)<br>• order: 23 (2.3%)<br>• class: 12 (1.2%)<br>• phylum: 1 (0.1%)<br>• superkingdom: 16 (1.6%)<br>• root: 86 (8.6%)	• Staphylococcus arlettae [taxid 29378]: 1 (0.1%)<br>• Lupinus albus [taxid 3870]: 1 (0.1%)
Benchmark OTU ID: `S_pneumoniae_HiSeq`<br>OTU taxon: Streptococcus pneumoniae TIGR4 [taxid 170187]<br>Expected: Streptococcus pneumoniae [taxid 1313] (species)<br>Number of reads: 1000<br>Number of identified reads: 867 (86.7%)	• species: 216 (21.6%)<br>• **genus: 524 (52.4%)**<br>• family: 6 (0.6%)<br>• order: 7 (0.7%)<br>• class: 5 (0.5%)<br>• phylum: 15 (1.5%)<br>• superkingdom: 15 (1.5%)<br>• root: 79 (7.9%)	• Streptococcus mitis [taxid 28037]: 4 (0.4%)<br>• Streptococcus equi [taxid 1336]: 1 (0.1%)<br>• Ligilactobacillus animalis [taxid 1605]: 1 (0.1%)<br>• Streptococcus oralis [taxid 1303]: 1 (0.1%)<br>• Streptococcus infantis [taxid 68892]: 1 (0.1%)
Benchmark OTU ID: `V_cholerae_HiSeq`<br>OTU taxon: Vibrio cholerae CP1032(5) [taxid 991923]<br>Expected: Vibrio cholerae [taxid 666] (species)<br>Number of reads: 1000<br>Number of identified reads: 881 (88.1%)	• species: 171 (17.1%)<br>• **genus: 497 (49.7%)**<br>• family: 30 (3.0%)<br>• order: 0 (0.0%)<br>• class: 47 (4.7%)<br>• phylum: 11 (1.1%)<br>• superkingdom: 8 (0.8%)<br>• root: 117 (11.7%)	• Pantoea cypripedii [taxid 55209]: 1 (0.1%)<br>• Providencia stuartii [taxid 588]: 1 (0.1%)<br>• Zobellella denitrificans [taxid 347534]: 1 (0.1%)<br>• Erwinia tracheiphila [taxid 65700]: 1 (0.1%)<br>• Klebsiella pneumoniae [taxid 573]: 1 (0.1%)
Benchmark OTU ID: `X_axonopodis_HiSeq`<br>OTU taxon: Xanthomonas axonopodis pv. manihotis str. UA323 [taxid 1185664]<br>Expected: Xanthomonas phaseoli [taxid 1985254] (species)<br>Number of reads: 1000<br>Number of identified reads: 891 (89.1%)	• species: 68 (6.8%)<br>• **genus: 580 (58.0%)**<br>• family: 58 (5.8%)<br>• order: 4 (0.4%)<br>• class: 17 (1.7%)<br>• phylum: 21 (2.1%)<br>• superkingdom: 16 (1.6%)<br>• root: 127 (12.7%)	• Xanthomonas arboricola [taxid 56448]: 5 (0.5%)<br>• Xanthomonas citri [taxid 346]: 4 (0.4%)<br>• Xanthomonas oryzae [taxid 347]: 2 (0.2%)<br>• Moorea sp. SIOASIH [taxid 2607817]: 1 (0.1%)<br>• Xanthomonas campestris [taxid 339]: 1 (0.1%)<br>• Salmonella enterica [taxid 28901]: 1 (0.1%)

Benchmark dataset, real reads

Operational Taxonomic Unit (OTU)	Correct identifications	Wrong or overspecific identifications at species rank
Benchmark OTU ID: `CP002059-_Cyanobacteria`<br>OTU taxon: ‘Nostoc azollae’ 0708 [taxid 551115]<br>Expected: Trichormus azollae [taxid 1164] (species)<br>Number of reads: 31481<br>Number of identified reads: 30733 (97.623%)	• **species: 12098 (38.429%)**<br>• genus: 177 (0.562%)<br>• family: 1198 (3.805%)<br>• order: 3833 (12.175%)<br>• phylum: 2487 (7.9%)<br>• superkingdom: 1582 (5.025%)<br>• root: 9275 (29.462%)	• Anabaena cylindrica [taxid 1165]: 74 (0.235%)<br>• Trichormus variabilis [taxid 264691]: 67 (0.212%)<br>• Nostoc cycadae [taxid 246795]: 32 (0.101%)<br>• Nodularia spumigena [taxid 70799]: 27 (0.085%)<br>• Cylindrospermum stagnale [taxid 142864]: 23 (0.073%)<br>• Microcystis aeruginosa [taxid 1126]: 14 (0.044%)<br>• Nostoc flagelliforme [taxid 1306274]: 14 (0.044%)<br>• other: 266 (0.844%)
Benchmark OTU ID: `CP000828-_Cyanobacteria`<br>OTU taxon: Acaryochloris marina MBIC11017 [taxid 329726]<br>Expected: Acaryochloris marina [taxid 155978] (species)<br>Number of reads: 39064<br>Number of identified reads: 38745 (99.183%)	• **species: 27922 (71.477%)**<br>• genus: 146 (0.373%)<br>• family: 0 (0.0%)<br>• order: 382 (0.977%)<br>• phylum: 1676 (4.29%)<br>• superkingdom: 2283 (5.844%)<br>• root: 6276 (16.065%)	• Crocosphaera watsonii [taxid 263511]: 4 (0.01%)<br>• Leptolyngbya sp. SIOISBB [taxid 2607771]: 3 (0.007%)<br>• Prochlorococcus marinus [taxid 1219]: 2 (0.005%)<br>• Nostoc flagelliforme [taxid 1306274]: 2 (0.005%)<br>• Oscillatoria acuminata [taxid 118323]: 2 (0.005%)<br>• Gloeobacter violaceus [taxid 33072]: 2 (0.005%)<br>• Lupinus albus [taxid 3870]: 2 (0.005%)<br>• other: 64 (0.163%)
Benchmark OTU ID: `AP011163-_Proteobacteria`<br>OTU taxon: Acetobacter pasteurianus IFO 3283-01-42C [taxid 634458]<br>Expected: Acetobacter pasteurianus [taxid 438] (species)<br>Number of reads: 5450<br>Number of identified reads: 5441 (99.834%)	• species: 1082 (19.853%)<br>• **genus: 2298 (42.165%)**<br>• family: 516 (9.467%)<br>• order: 24 (0.44%)<br>• class: 192 (3.522%)<br>• phylum: 165 (3.027%)<br>• superkingdom: 296 (5.431%)<br>• root: 861 (15.798%)	• Acetobacter pomorum [taxid 65959]: 31 (0.568%)<br>• Acetobacter okinawensis [taxid 1076594]: 5 (0.091%)<br>• Acetobacter cibinongensis [taxid 146475]: 5 (0.091%)<br>• Acetobacter orientalis [taxid 146474]: 5 (0.091%)<br>• Acetobacter oryzoeni [taxid 2500548]: 4 (0.073%)<br>• Acetobacter cerevisiae [taxid 178900]: 3 (0.055%)<br>• Acetobacter tropicalis [taxid 104102]: 3 (0.055%)<br>• Acetobacter malorum [taxid 178901]: 3 (0.055%)<br>• other: 36 (0.66%)
Benchmark OTU ID: `AP011135-_Proteobacteria`<br>OTU taxon: Acetobacter pasteurianus IFO 3283-07 [taxid 634454]<br>Expected: Acetobacter pasteurianus [taxid 438] (species)<br>Number of reads: 5655<br>Number of identified reads: 5643 (99.787%)	• species: 1154 (20.406%)<br>• **genus: 2423 (42.847%)**<br>• family: 548 (9.69%)<br>• order: 13 (0.229%)<br>• class: 207 (3.66%)<br>• phylum: 182 (3.218%)<br>• superkingdom: 307 (5.428%)<br>• root: 799 (14.129%)	• Acetobacter pomorum [taxid 65959]: 34 (0.601%)<br>• Komagataeibacter saccharivorans [taxid 265959]: 7 (0.123%)<br>• Acetobacter syzygii [taxid 146476]: 4 (0.07%)<br>• Acetobacter oryzoeni [taxid 2500548]: 3 (0.053%)<br>• Acetobacter aceti [taxid 435]: 3 (0.053%)<br>• Gluconobacter oxydans [taxid 442]: 3 (0.053%)<br>• Acetobacter cibinongensis [taxid 146475]: 3 (0.053%)<br>• Acetobacter tropicalis [taxid 104102]: 3 (0.053%)<br>• other: 37 (0.654%)

Operational Taxonomic Unit (OTU)	Correct identifications	Wrong or overspecific identifications at species rank
Benchmark OTU ID: `AP011142-_Proteobacteria`<br>OTU taxon: Acetobacter pasteurianus IFO 3283-22 [taxid 634455]<br>Expected: Acetobacter pasteurianus [taxid 438] (species)<br>Number of reads: 5657<br>Number of identified reads: 5640 (99.699%)	• species: 1139 (20.134%)<br>• **genus: 2400 (42.425%)**<br>• family: 536 (9.474%)<br>• order: 22 (0.388%)<br>• class: 196 (3.464%)<br>• phylum: 190 (3.358%)<br>• superkingdom: 303 (5.356%)<br>• root: 843 (14.901%)	• Acetobacter pomorum [taxid 65959]: 23 (0.406%)<br>• Acetobacter malorum [taxid 178901]: 4 (0.07%)<br>• Gluconobacter oxydans [taxid 442]: 3 (0.053%)<br>• Komagataeibacter saccharivorans [taxid 265959]: 3 (0.053%)<br>• Acetobacter oryzoeni [taxid 2500548]: 3 (0.053%)<br>• Acetobacter ascendens [taxid 481146]: 3 (0.053%)<br>• Acetobacter cibinongensis [taxid 146475]: 2 (0.035%)<br>• Magnetospirillum gryphiswaldense [taxid 55518]: 2 (0.035%)<br>• other: 39 (0.689%)
Benchmark OTU ID: `AP011149-_Proteobacteria`<br>OTU taxon: Acetobacter pasteurianus IFO 3283-26 [taxid 634456]<br>Expected: Acetobacter pasteurianus [taxid 438] (species)<br>Number of reads: 5657<br>Number of identified reads: 5640 (99.699%)	• species: 1114 (19.692%)<br>• **genus: 2345 (41.453%)**<br>• family: 526 (9.298%)<br>• order: 23 (0.406%)<br>• class: 224 (3.959%)<br>• phylum: 197 (3.482%)<br>• superkingdom: 258 (4.56%)<br>• root: 935 (16.528%)	• Acetobacter pomorum [taxid 65959]: 31 (0.547%)<br>• Acetobacter indonesiensis [taxid 104101]: 4 (0.07%)<br>• Komagataeibacter saccharivorans [taxid 265959]: 3 (0.053%)<br>• Acetobacter aceti [taxid 435]: 3 (0.053%)<br>• Acetobacter malorum [taxid 178901]: 3 (0.053%)<br>• Acetobacter orientalis [taxid 146474]: 3 (0.053%)<br>• Acetobacter tropicalis [taxid 104102]: 3 (0.053%)<br>• Acetobacter oryzoeni [taxid 2500548]: 3 (0.053%)<br>• other: 39 (0.689%)
Benchmark OTU ID: `AP011156-_Proteobacteria`<br>OTU taxon: Acetobacter pasteurianus IFO 3283-32 [taxid 634457]<br>Expected: Acetobacter pasteurianus [taxid 438] (species)<br>Number of reads: 5651<br>Number of identified reads: 5638 (99.769%)	• species: 1079 (19.093%)<br>• **genus: 2428 (42.965%)**<br>• family: 541 (9.573%)<br>• order: 18 (0.318%)<br>• class: 224 (3.963%)<br>• phylum: 177 (3.132%)<br>• superkingdom: 287 (5.078%)<br>• root: 868 (15.36%)	• Acetobacter pomorum [taxid 65959]: 34 (0.601%)<br>• Acetobacter malorum [taxid 178901]: 7 (0.123%)<br>• Acetobacter ascendens [taxid 481146]: 7 (0.123%)<br>• Komagataeibacter saccharivorans [taxid 265959]: 5 (0.088%)<br>• Acetobacter tropicalis [taxid 104102]: 4 (0.07%)<br>• Acetobacter oryzoeni [taxid 2500548]: 4 (0.07%)<br>• Acetobacter indonesiensis [taxid 104101]: 3 (0.053%)<br>• Acetobacter peroxydans [taxid 104098]: 3 (0.053%)<br>• other: 53 (0.937%)
Benchmark OTU ID: `CP002987-_Firmicutes`<br>OTU taxon: Acetobacterium woodii DSM 1030 [taxid 931626]<br>Expected: Acetobacterium woodii [taxid 33952] (species)<br>Number of reads: 5703<br>Number of identified reads: 5642 (98.93%)	• **species: 3900 (68.385%)**<br>• genus: 211 (3.699%)<br>• family: 15 (0.263%)<br>• order: 111 (1.946%)<br>• class: 5 (0.087%)<br>• phylum: 417 (7.311%)<br>• superkingdom: 305 (5.348%)<br>• root: 672 (11.783%)	• Acetobacterium bakii [taxid 52689]: 4 (0.07%)<br>• Acetobacterium wieringae [taxid 52694]: 2 (0.035%)<br>• Clostridium botulinum [taxid 1491]: 1 (0.017%)<br>• Nicotiana tabacum [taxid 4097]: 1 (0.017%)<br>• Paenibacillus lautus [taxid 1401]: 1 (0.017%)<br>• Clostridium tepidum [taxid 1962263]: 1 (0.017%)<br>• Methylocella silvestris [taxid 199596]: 1 (0.017%)<br>• Pseudomonas palleroniana [taxid 191390]: 1 (0.017%)<br>• other: 10 (0.175%)

Operational Taxonomic Unit (OTU)	Correct identifications	Wrong or overspecific identifications at species rank
Benchmark OTU ID: `CP002535-_Crenarchaeota`<br>OTU taxon: Acidianus hospitalis W1 [taxid 933801]<br>Expected: Acidianus hospitalis [taxid 563177] (species)<br>Number of reads: 4469<br>Number of identified reads: 4352 (97.381%)	• species: 1259 (28.171%)<br>• **genus: 1798 (40.232%)**<br>• family: 240 (5.37%)<br>• order: 12 (0.268%)<br>• class: 29 (0.648%)<br>• phylum: 0 (0.0%)<br>• superkingdom: 31 (0.693%)<br>• root: 951 (21.279%)	• Acidianus infernus [taxid 12915]: 14 (0.313%)<br>• Acidianus ambivalens [taxid 2283]: 13 (0.29%)<br>• Acidianus brierleyi [taxid 41673]: 7 (0.156%)<br>• Candidatus Acidianus copahuensis [taxid 1160895]: 6 (0.134%)<br>• Sulfolobus islandicus [taxid 43080]: 5 (0.111%)<br>• Metallosphaera yellowstonensis [taxid 1111107]: 2 (0.044%)<br>• Acidianus sulfidivorans [taxid 312539]: 2 (0.044%)<br>• Acidianus manzaensis [taxid 282676]: 2 (0.044%)<br>• other: 11 (0.246%)
Benchmark OTU ID: `CP001742-_Crenarchaeota`<br>OTU taxon: Acidilobus saccharovorans 345-15 [taxid 666510]<br>Expected: Acidilobus saccharovorans [taxid 242703] (species)<br>Number of reads: 2866<br>Number of identified reads: 2808 (97.976%)	• **species: 1602 (55.896%)**<br>• genus: 112 (3.907%)<br>• family: 0 (0.0%)<br>• order: 17 (0.593%)<br>• class: 27 (0.942%)<br>• phylum: 0 (0.0%)<br>• superkingdom: 22 (0.767%)<br>• root: 951 (33.182%)	• Phaeobacter gallaeciensis [taxid 60890]: 1 (0.034%)<br>• Acidianus brierleyi [taxid 41673]: 1 (0.034%)<br>• Candidatus Methanoperedens nitroreducens [taxid 1392998]: 1 (0.034%)<br>• Colletotrichum salicis [taxid 1209931]: 1 (0.034%)<br>• Eimeria tenella [taxid 5802]: 1 (0.034%)<br>• Aphanomyces euteiches [taxid 100861]: 1 (0.034%)<br>• Thermoprotei archaeon [taxid 2250277]: 1 (0.034%)
Benchmark OTU ID: `CP002985-_Proteobacteria`<br>OTU taxon: Acidithiobacillus ferrivorans SS3 [taxid 743299]<br>Expected: Acidithiobacillus ferrivorans [taxid 160808] (species)<br>Number of reads: 6333<br>Number of identified reads: 6307 (99.589%)	• **species: 2899 (45.776%)**<br>• genus: 1242 (19.611%)<br>• family: 0 (0.0%)<br>• order: 0 (0.0%)<br>• class: 0 (0.0%)<br>• phylum: 740 (11.684%)<br>• superkingdom: 416 (6.568%)<br>• root: 1004 (15.853%)	• Acidithiobacillus caldus [taxid 33059]: 9 (0.142%)<br>• Acidithiobacillus ferrooxidans [taxid 920]: 7 (0.11%)<br>• Acidithiobacillus sp. SH [taxid 187490]: 5 (0.078%)<br>• Acidithiobacillus thiooxidans [taxid 930]: 5 (0.078%)<br>• Salmonella enterica [taxid 28901]: 2 (0.031%)<br>• Acidithiobacillus ferridurans [taxid 1232575]: 2 (0.031%)<br>• Acidithiobacillus sulfuriphilus [taxid 1867749]: 2 (0.031%)<br>• Photobacterium aquae [taxid 1195763]: 1 (0.015%)<br>• other: 15 (0.236%)
Benchmark OTU ID: `CP001132-_Proteobacteria`<br>OTU taxon: Acidithiobacillus ferrooxidans ATCC 53993 [taxid 380394]<br>Expected: Acidithiobacillus ferrooxidans [taxid 920] (species)<br>Number of reads: 5607<br>Number of identified reads: 5590 (99.696%)	• species: 430 (7.668%)<br>• **genus: 1693 (30.194%)**<br>• family: 0 (0.0%)<br>• order: 0 (0.0%)<br>• class: 0 (0.0%)<br>• phylum: 990 (17.656%)<br>• superkingdom: 800 (14.267%)<br>• root: 1659 (29.588%)	• Acidithiobacillus ferrivorans [taxid 160808]: 63 (1.123%)<br>• Acidithiobacillus sp. 'AMD consortium' [taxid 2614801]: 19 (0.338%)<br>• Acidithiobacillus ferridurans [taxid 1232575]: 15 (0.267%)<br>• Acidithiobacillus thiooxidans [taxid 930]: 14 (0.249%)<br>• Acidithiobacillus sulfuriphilus [taxid 1867749]: 8 (0.142%)<br>• Acidithiobacillus sp. SH [taxid 187490]: 7 (0.124%)<br>• Acidithiobacillus caldus [taxid 33059]: 5 (0.089%)<br>• Labilithrix luteola [taxid 1391654]: 1 (0.017%)<br>• other: 39 (0.695%)

Operational Taxonomic Unit (OTU)	Correct identifications	Wrong or overspecific identifications at species rank
Benchmark OTU ID: `CP001472-_Acidobacteria`<br>OTU taxon: Acidobacterium capsulatum ATCC 51196 [taxid 240015]<br>Expected: Acidobacterium capsulatum [taxid 33075] (species)<br>Number of reads: 314711<br>Number of identified reads: 313677 (99.671%)	• **species: 220712 (70.131%)**<br>• genus: 1 (0.0%)<br>• family: 8562 (2.72%)<br>• order: 708 (0.224%)<br>• class: 414 (0.131%)<br>• phylum: 5281 (1.678%)<br>• superkingdom: 35595 (11.31%)<br>• root: 42077 (13.37%)	• Acidipila rosea [taxid 768535]: 92 (0.029%)<br>• Acidipila dinghuensis [taxid 1560006]: 73 (0.023%)<br>• Acidisarcina polymorpha [taxid 2211140]: 53 (0.016%)<br>• Terriglobus roseus [taxid 392734]: 24 (0.007%)<br>• Granulicella mallensis [taxid 940614]: 19 (0.006%)<br>• bacterium [taxid 1869227]: 17 (0.005%)<br>• Edaphobacter modestus [taxid 388466]: 16 (0.005%)<br>• Candidatus Koribacter versatilis [taxid 658062]: 15 (0.004%)<br>• other: 798 (0.253%)
Benchmark OTU ID: `CP000481-_Actinobacteria`<br>OTU taxon: Acidothermus cellulolyticus 11B [taxid 351607]<br>Expected: Acidothermus cellulolyticus [taxid 28049] (species)<br>Number of reads: 9570<br>Number of identified reads: 9491 (99.174%)	• **species: 5744 (60.02%)**<br>• genus: 0 (0.0%)<br>• family: 0 (0.0%)<br>• order: 0 (0.0%)<br>• class: 1181 (12.34%)<br>• phylum: 21 (0.219%)<br>• superkingdom: 838 (8.756%)<br>• root: 1688 (17.638%)	• Mycobacterium tuberculosis [taxid 1773]: 3 (0.031%)<br>• Nocardia cyriacigeorgica [taxid 135487]: 1 (0.01%)<br>• Pyramimonas parkeae [taxid 36894]: 1 (0.01%)<br>• Haloechinothrix alba [taxid 664784]: 1 (0.01%)<br>• Blastococcus colisei [taxid 1564162]: 1 (0.01%)<br>• Corethrella appendiculata [taxid 1370023]: 1 (0.01%)<br>• Haloplanus rubicundus [taxid 1547898]: 1 (0.01%)<br>• Massilia plicata [taxid 321984]: 1 (0.01%)<br>• other: 31 (0.323%)
Benchmark OTU ID: `CP002521-_Proteobacteria`<br>OTU taxon: Acidovorax avenae subsp. avenae ATCC 19860 [taxid 643561]<br>Expected: Acidovorax avenae [taxid 80867] (species)<br>Number of reads: 11451<br>Number of identified reads: 11418 (99.711%)	• species: 3393 (29.63%)<br>• **genus: 3426 (29.918%)**<br>• family: 1075 (9.387%)<br>• order: 1246 (10.881%)<br>• class: 132 (1.152%)<br>• phylum: 731 (6.383%)<br>• superkingdom: 553 (4.829%)<br>• root: 856 (7.475%)	• Acidovorax citrulli [taxid 80869]: 34 (0.296%)<br>• Acidovorax cattleyae [taxid 80868]: 21 (0.183%)<br>• Acidovorax konjaci [taxid 32040]: 11 (0.096%)<br>• Acidovorax anthurii [taxid 78229]: 7 (0.061%)<br>• Salmonella enterica [taxid 28901]: 4 (0.034%)<br>• Acidovorax delafieldii [taxid 47920]: 4 (0.034%)<br>• Acidovorax wautersii [taxid 1177982]: 3 (0.026%)<br>• Cupriavidus pauculus [taxid 82633]: 2 (0.017%)<br>• other: 60 (0.523%)
Benchmark OTU ID: `CP000512-_Proteobacteria`<br>OTU taxon: Acidovorax citrulli AAC00-1 [taxid 397945]<br>Expected: Acidovorax citrulli [taxid 80869] (species)<br>Number of reads: 11160<br>Number of identified reads: 11117 (99.614%)	• **species: 3373 (30.224%)**<br>• genus: 3181 (28.503%)<br>• family: 965 (8.646%)<br>• order: 1212 (10.86%)<br>• class: 126 (1.129%)<br>• phylum: 849 (7.607%)<br>• superkingdom: 513 (4.596%)<br>• root: 885 (7.93%)	• Acidovorax avenae [taxid 80867]: 52 (0.465%)<br>• Acidovorax cattleyae [taxid 80868]: 21 (0.188%)<br>• Acidovorax valerianellae [taxid 187868]: 5 (0.044%)<br>• Acidovorax wautersii [taxid 1177982]: 4 (0.035%)<br>• Acidovorax anthurii [taxid 78229]: 4 (0.035%)<br>• Acidovorax konjaci [taxid 32040]: 3 (0.026%)<br>• Clostridium botulinum [taxid 1491]: 2 (0.017%)<br>• Giesbergeria anulus [taxid 180197]: 2 (0.017%)<br>• other: 72 (0.645%)

Operational Taxonomic Unit (OTU)	Correct identifications	Wrong or overspecific identifications at species rank
Benchmark OTU ID: `CP000539-_Proteobacteria`<br>OTU taxon: Acidovorax sp. JS42 [taxid 232721]<br>Expected: Acidovorax [taxid 12916] (genus)<br>Number of reads: 9126<br>Number of identified reads: 9086 (99.561%)	• genus: 857 (9.39%)<br>• **family: 4554 (49.901%)**<br>• order: 1261 (13.817%)<br>• class: 144 (1.577%)<br>• phylum: 1244 (13.631%)<br>• superkingdom: 391 (4.284%)<br>• root: 622 (6.815%)	• Diaphorobacter nitroreducens [taxid 164759]: 12 (0.131%)<br>• Alicycliphilus denitrificans [taxid 179636]: 9 (0.098%)<br>• Acidovorax ebreus [taxid 721785]: 5 (0.054%)<br>• Acidovorax delafieldii [taxid 47920]: 5 (0.054%)<br>• Comamonas testosteroni [taxid 285]: 4 (0.043%)<br>• Comamonas sp. JNW [taxid 2170731]: 3 (0.032%)<br>• Acidovorax citrulli [taxid 80869]: 3 (0.032%)<br>• other: 122 (1.336%)
Benchmark OTU ID: `CP003872-_Proteobacteria`<br>OTU taxon: Acidovorax sp. KKS102 [taxid 358220]<br>Expected: Acidovorax [taxid 12916] (genus)<br>Number of reads: 10809<br>Number of identified reads: 10777 (99.703%)	• **genus: 4794 (44.351%)**<br>• family: 1031 (9.538%)<br>• order: 2848 (26.348%)<br>• class: 122 (1.128%)<br>• phylum: 737 (6.818%)<br>• superkingdom: 360 (3.33%)<br>• root: 875 (8.095%)	• Acidovorax delafieldii [taxid 47920]: 15 (0.138%)<br>• Acidovorax temperans [taxid 80878]: 6 (0.055%)<br>• Curvibacter putative symbiont of Hydra magnipapillata [taxid 667019]: 5 (0.046%)<br>• Comamonas testosteroni [taxid 285]: 4 (0.037%)<br>• Acidovorax carolinensis [taxid 553814]: 4 (0.037%)<br>• Acidovorax anthurii [taxid 78229]: 3 (0.027%)<br>• Pseudomonas aeruginosa [taxid 287]: 3 (0.027%)<br>• other: 75 (0.693%)
Benchmark OTU ID: `CP001941-_Euryarchaeota`<br>OTU taxon: Aciduliprofundum boonei T469 [taxid 439481]<br>Expected: Aciduliprofundum boonei [taxid 379547] (species)<br>Number of reads: 1164<br>Number of identified reads: 1153 (99.054%)	• **species: 802 (68.9%)**<br>• genus: 27 (2.319%)<br>• phylum: 3 (0.257%)<br>• superkingdom: 45 (3.865%)<br>• root: 266 (22.852%)	• Acidilobales archaeon [taxid 2268176]: 1 (0.085%)
Benchmark OTU ID: `CP001921-_Pathogens`<br>OTU taxon: Acinetobacter baumannii 1656-2 [taxid 696749]<br>Expected: Acinetobacter baumannii [taxid 470] (species)<br>Number of reads: 8638<br>Number of identified reads: 8550 (98.981%)	• species: 730 (8.451%)<br>• **genus: 6504 (75.295%)**<br>• family: 39 (0.451%)<br>• order: 48 (0.555%)<br>• class: 195 (2.257%)<br>• phylum: 222 (2.57%)<br>• superkingdom: 214 (2.477%)<br>• root: 588 (6.807%)	• Acinetobacter pittii [taxid 48296]: 28 (0.324%)<br>• Acinetobacter calcoaceticus [taxid 471]: 13 (0.15%)<br>• Acinetobacter nosocomialis [taxid 106654]: 11 (0.127%)<br>• Acinetobacter oleivorans [taxid 1148157]: 8 (0.092%)<br>• Acinetobacter lactucae [taxid 1785128]: 6 (0.069%)<br>• Acinetobacter seifertii [taxid 1530123]: 6 (0.069%)<br>• Acinetobacter towneri [taxid 202956]: 5 (0.057%)<br>• Acinetobacter qingfengensis [taxid 1262585]: 3 (0.034%)<br>• other: 71 (0.821%)
Benchmark OTU ID: `CP001172-_Pathogens`<br>OTU taxon: Acinetobacter baumannii AB307-0294 [taxid 557600]<br>Expected: Acinetobacter baumannii [taxid 470] (species)<br>Number of reads: 8188<br>Number of identified reads: 8098 (98.9%)	• species: 1114 (13.605%)<br>• **genus: 5849 (71.433%)**<br>• family: 34 (0.415%)<br>• order: 42 (0.512%)<br>• class: 170 (2.076%)<br>• phylum: 179 (2.186%)<br>• superkingdom: 175 (2.137%)<br>• root: 531 (6.485%)	• Acinetobacter pittii [taxid 48296]: 28 (0.341%)<br>• Acinetobacter nosocomialis [taxid 106654]: 18 (0.219%)<br>• Acinetobacter calcoaceticus [taxid 471]: 12 (0.146%)<br>• Acinetobacter lactucae [taxid 1785128]: 6 (0.073%)<br>• Acinetobacter towneri [taxid 202956]: 5 (0.061%)<br>• Acinetobacter seifertii [taxid 1530123]: 5 (0.061%)<br>• Acinetobacter lwoffii [taxid 28090]: 5 (0.061%)<br>• Acinetobacter qingfengensis [taxid 1262585]: 4 (0.048%)<br>• other: 63 (0.769%)

Operational Taxonomic Unit (OTU)	Correct identifications	Wrong or overspecific identifications at species rank
Benchmark OTU ID: `CP000863-_Pathogens`<br>OTU taxon: Acinetobacter baumannii ACICU [taxid 405416]<br>Expected: Acinetobacter baumannii [taxid 470] (species)<br>Number of reads: 8545<br>Number of identified reads: 8460 (99.005%)	• species: 797 (9.327%)<br>• **genus: 6417 (75.096%)**<br>• family: 27 (0.315%)<br>• order: 45 (0.526%)<br>• class: 204 (2.387%)<br>• phylum: 181 (2.118%)<br>• superkingdom: 233 (2.726%)<br>• root: 554 (6.483%)	• Acinetobacter pittii [taxid 48296]: 34 (0.397%)<br>• Acinetobacter calcoaceticus [taxid 471]: 24 (0.28%)<br>• Acinetobacter haemolyticus [taxid 29430]: 6 (0.07%)<br>• Acinetobacter oleivorans [taxid 1148157]: 6 (0.07%)<br>• Acinetobacter nosocomialis [taxid 106654]: 6 (0.07%)<br>• Acinetobacter lactucae [taxid 1785128]: 5 (0.058%)<br>• Acinetobacter seifertii [taxid 1530123]: 4 (0.046%)<br>• Acinetobacter marinus [taxid 281375]: 4 (0.046%)<br>• other: 53 (0.62%)
Benchmark OTU ID: `CU459141-_Pathogens`<br>OTU taxon: Acinetobacter baumannii AYE [taxid 509173]<br>Expected: Acinetobacter baumannii [taxid 470] (species)<br>Number of reads: 8627<br>Number of identified reads: 8536 (98.945%)	• species: 1207 (13.99%)<br>• **genus: 5945 (68.911%)**<br>• family: 36 (0.417%)<br>• order: 40 (0.463%)<br>• class: 277 (3.21%)<br>• phylum: 219 (2.538%)<br>• superkingdom: 249 (2.886%)<br>• root: 558 (6.468%)	• Acinetobacter pittii [taxid 48296]: 27 (0.312%)<br>• Acinetobacter seifertii [taxid 1530123]: 10 (0.115%)<br>• Acinetobacter calcoaceticus [taxid 471]: 9 (0.104%)<br>• Acinetobacter lactucae [taxid 1785128]: 8 (0.092%)<br>• Acinetobacter nosocomialis [taxid 106654]: 6 (0.069%)<br>• Acinetobacter oleivorans [taxid 1148157]: 5 (0.057%)<br>• Escherichia coli [taxid 562]: 5 (0.057%)<br>• Acinetobacter haemolyticus [taxid 29430]: 5 (0.057%)<br>• other: 70 (0.811%)
Benchmark OTU ID: `CP003856-_Pathogens`<br>OTU taxon: Acinetobacter baumannii TYTH-1 [taxid 1100841]<br>Expected: Acinetobacter baumannii [taxid 470] (species)<br>Number of reads: 8676<br>Number of identified reads: 8596 (99.077%)	• species: 752 (8.667%)<br>• **genus: 6484 (74.734%)**<br>• family: 28 (0.322%)<br>• order: 41 (0.472%)<br>• class: 230 (2.65%)<br>• phylum: 188 (2.166%)<br>• superkingdom: 227 (2.616%)<br>• root: 642 (7.399%)	• Acinetobacter pittii [taxid 48296]: 27 (0.311%)<br>• Acinetobacter calcoaceticus [taxid 471]: 15 (0.172%)<br>• Acinetobacter seifertii [taxid 1530123]: 11 (0.126%)<br>• Acinetobacter lactucae [taxid 1785128]: 10 (0.115%)<br>• Acinetobacter beijerinckii [taxid 262668]: 5 (0.057%)<br>• Acinetobacter qingfengensis [taxid 1262585]: 5 (0.057%)<br>• Acinetobacter lanii [taxid 2715163]: 5 (0.057%)<br>• Acinetobacter nosocomialis [taxid 106654]: 5 (0.057%)<br>• other: 79 (0.91%)
Benchmark OTU ID: `CR543861-_Proteobacteria`<br>OTU taxon: Acinetobacter baylyi ADP1 [taxid 62977]<br>Expected: Acinetobacter baylyi [taxid 202950] (species)<br>Number of reads: 7213<br>Number of identified reads: 7171 (99.417%)	• **species: 3658 (50.713%)**<br>• genus: 2210 (30.639%)<br>• family: 32 (0.443%)<br>• order: 64 (0.887%)<br>• class: 208 (2.883%)<br>• phylum: 197 (2.731%)<br>• superkingdom: 208 (2.883%)<br>• root: 588 (8.151%)	• Acinetobacter baumannii [taxid 470]: 9 (0.124%)<br>• Acinetobacter towneri [taxid 202956]: 6 (0.083%)<br>• Acinetobacter soli [taxid 487316]: 4 (0.055%)<br>• Acinetobacter boissieri [taxid 1219383]: 3 (0.041%)<br>• Acinetobacter calcoaceticus [taxid 471]: 3 (0.041%)<br>• Acinetobacter nectaris [taxid 1219382]: 2 (0.027%)<br>• Acinetobacter marinus [taxid 281375]: 2 (0.027%)<br>• Acinetobacter pittii [taxid 48296]: 2 (0.027%)<br>• other: 25 (0.346%)

Operational Taxonomic Unit (OTU)	Correct identifications	Wrong or overspecific identifications at species rank
Benchmark OTU ID: `CP002080-_Proteobacteria`<br>OTU taxon: Acinetobacter oleivorans DR1 [taxid 436717]<br>Expected: Acinetobacter oleivorans [taxid 1148157] (species)<br>Number of reads: 8459<br>Number of identified reads: 8365 (98.888%)	• species: 657 (7.766%)<br>• **genus: 6516 (77.03%)**<br>• family: 28 (0.331%)<br>• order: 49 (0.579%)<br>• class: 151 (1.785%)<br>• phylum: 192 (2.269%)<br>• superkingdom: 204 (2.411%)<br>• root: 565 (6.679%)	• Acinetobacter baumannii [taxid 470]: 70 (0.827%)<br>• Acinetobacter pittii [taxid 48296]: 39 (0.461%)<br>• Acinetobacter calcoaceticus [taxid 471]: 28 (0.331%)<br>• Acinetobacter lactucae [taxid 1785128]: 18 (0.212%)<br>• Acinetobacter seifertii [taxid 1530123]: 11 (0.13%)<br>• Acinetobacter johnsonii [taxid 40214]: 6 (0.07%)<br>• Acinetobacter sp. JW [taxid 2302364]: 5 (0.059%)<br>• Acinetobacter baylyi [taxid 202950]: 4 (0.047%)<br>• other: 63 (0.744%)
Benchmark OTU ID: `CP002177-_Proteobacteria`<br>OTU taxon: Acinetobacter pittii PHEA-2 [taxid 871585]<br>Expected: Acinetobacter pittii [taxid 48296] (species)<br>Number of reads: 7807<br>Number of identified reads: 7742 (99.167%)	• species: 647 (8.287%)<br>• **genus: 6032 (77.263%)**<br>• family: 38 (0.486%)<br>• order: 18 (0.23%)<br>• class: 137 (1.754%)<br>• phylum: 169 (2.164%)<br>• superkingdom: 183 (2.344%)<br>• root: 511 (6.545%)	• Acinetobacter baumannii [taxid 470]: 80 (1.024%)<br>• Acinetobacter calcoaceticus [taxid 471]: 29 (0.371%)<br>• Acinetobacter lactucae [taxid 1785128]: 23 (0.294%)<br>• Acinetobacter seifertii [taxid 1530123]: 12 (0.153%)<br>• Acinetobacter larvae [taxid 1789224]: 6 (0.076%)<br>• Acinetobacter haemolyticus [taxid 29430]: 6 (0.076%)<br>• Acinetobacter oleivorans [taxid 1148157]: 6 (0.076%)<br>• Acinetobacter nosocomialis [taxid 106654]: 4 (0.051%)<br>• other: 59 (0.755%)
Benchmark OTU ID: `CP005929-_Actinobacteria`<br>OTU taxon: Actinoplanes sp. N902-109 [taxid 649831]<br>Expected: Actinoplanes [taxid 1865] (genus)<br>Number of reads: 42814<br>Number of identified reads: 42651 (99.619%)	• **genus: 28548 (66.679%)**<br>• family: 3279 (7.658%)<br>• order: 0 (0.0%)<br>• class: 4108 (9.594%)<br>• phylum: 38 (0.088%)<br>• superkingdom: 2745 (6.411%)<br>• root: 3895 (9.097%)	• Actinoplanes friuliensis [taxid 196914]: 40 (0.093%)<br>• Pseudosporangium ferrugineum [taxid 439699]: 24 (0.056%)<br>• Actinoplanes atraurantiacus [taxid 1036182]: 18 (0.042%)<br>• Couchioplanes caeruleus [taxid 56438]: 13 (0.03%)<br>• Actinoplanes teichomyceticus [taxid 1867]: 12 (0.028%)<br>• Actinoplanes awajinensis [taxid 135946]: 11 (0.025%)<br>• Actinoplanes brasiliensis [taxid 52695]: 10 (0.023%)<br>• other: 210 (0.49%)
Benchmark OTU ID: `CP003555-_Proteobacteria`<br>OTU taxon: Advenella kashmirensis WT001 [taxid 1036672]<br>Expected: Advenella kashmirensis [taxid 310575] (species)<br>Number of reads: 8939<br>Number of identified reads: 8929 (99.888%)	• species: 2759 (30.864%)<br>• **genus: 3084 (34.5%)**<br>• family: 279 (3.121%)<br>• order: 296 (3.311%)<br>• class: 97 (1.085%)<br>• phylum: 730 (8.166%)<br>• superkingdom: 432 (4.832%)<br>• root: 1244 (13.916%)	• Advenella incenata [taxid 267800]: 138 (1.543%)<br>• Advenella mimigardefordensis [taxid 302406]: 48 (0.536%)<br>• Bordetella pertussis [taxid 520]: 1 (0.011%)<br>• Pseudoalteromonas phenolica [taxid 161398]: 1 (0.011%)<br>• Deinococcus proteolyticus [taxid 55148]: 1 (0.011%)<br>• Pseudomonas stutzeri [taxid 316]: 1 (0.011%)<br>• Pseudolabrys taiwanensis [taxid 331696]: 1 (0.011%)<br>• Phyllobacterium salinisoli [taxid 1899321]: 1 (0.011%)<br>• other: 33 (0.369%)

Operational Taxonomic Unit (OTU)	Correct identifications	Wrong or overspecific identifications at species rank
Benchmark OTU ID: `CP003280-_Bacteroidetes`<br>OTU taxon: Aequorivita sublithincola DSM 14238 [taxid 746697]<br>Expected: Aequorivita sublithincola [taxid 101385] (species)<br>Number of reads: 22946<br>Number of identified reads: 22735 (99.08%)	• **species: 12876 (56.114%)**<br>• genus: 2445 (10.655%)<br>• family: 2949 (12.851%)<br>• order: 328 (1.429%)<br>• class: 9 (0.039%)<br>• phylum: 799 (3.482%)<br>• superkingdom: 952 (4.148%)<br>• root: 2369 (10.324%)	• Aequorivita antarctica [taxid 153266]: 15 (0.065%)<br>• Gelidibacter algens [taxid 49280]: 10 (0.043%)<br>• Aequorivita lipolytica [taxid 153267]: 8 (0.034%)<br>• Aequorivita soesokkakensis [taxid 1385699]: 7 (0.03%)<br>• Vitellibacter aquimaris [taxid 1548749]: 5 (0.021%)<br>• Cochleicola gelatinilyticus [taxid 1763537]: 5 (0.021%)<br>• Pricia antarctica [taxid 641691]: 3 (0.013%)<br>• Aequorivita viscosa [taxid 797419]: 3 (0.013%)<br>• other: 83 (0.361%)
Benchmark OTU ID: `CP002512-_Firmicutes`<br>OTU taxon: Aerococcus urinae ACS-120-V-Col10a [taxid 866775]<br>Expected: Aerococcus urinae [taxid 1376] (species)<br>Number of reads: 2560<br>Number of identified reads: 2534 (98.984%)	• species: 946 (36.953%)<br>• **genus: 1014 (39.609%)**<br>• family: 8 (0.312%)<br>• order: 67 (2.617%)<br>• class: 39 (1.523%)<br>• phylum: 75 (2.929%)<br>• superkingdom: 131 (5.117%)<br>• root: 252 (9.843%)	• Aerococcus sanguinicola [taxid 119206]: 3 (0.117%)<br>• Aerococcus christensenii [taxid 87541]: 2 (0.078%)<br>• Neisseria gonorrhoeae [taxid 485]: 2 (0.078%)<br>• Thermoanaerobacterium sp. RBIITD [taxid 1550240]: 1 (0.039%)<br>• Peptoniphilus lacrimalis [taxid 33031]: 1 (0.039%)<br>• Fructobacillus ficulneus [taxid 157463]: 1 (0.039%)<br>• Lacticaseibacillus paracasei [taxid 1597]: 1 (0.039%)<br>• Oryza sativa [taxid 4530]: 1 (0.039%)<br>• other: 8 (0.312%)
Benchmark OTU ID: `CP000644-_Proteobacteria`<br>OTU taxon: Aeromonas salmonicida subsp. salmonicida A449 [taxid 382245]<br>Expected: Aeromonas salmonicida [taxid 645] (species)<br>Number of reads: 9696<br>Number of identified reads: 9663 (99.659%)	• species: 1407 (14.511%)<br>• **genus: 5265 (54.3%)**<br>• family: 19 (0.195%)<br>• order: 1 (0.01%)<br>• class: 1504 (15.511%)<br>• phylum: 250 (2.578%)<br>• superkingdom: 300 (3.094%)<br>• root: 913 (9.416%)	• Aeromonas veronii [taxid 654]: 25 (0.257%)<br>• Aeromonas hydrophila [taxid 644]: 15 (0.154%)<br>• Aeromonas media [taxid 651]: 11 (0.113%)<br>• Aeromonas sobria [taxid 646]: 9 (0.092%)<br>• Aeromonas molluscorum [taxid 271417]: 9 (0.092%)<br>• Aeromonas simiae [taxid 218936]: 8 (0.082%)<br>• Aeromonas caviae [taxid 648]: 7 (0.072%)<br>• Aeromonas cavernicola [taxid 1006623]: 7 (0.072%)<br>• other: 73 (0.752%)
Benchmark OTU ID: `CP002607-_Proteobacteria`<br>OTU taxon: Aeromonas veronii B565 [taxid 998088]<br>Expected: Aeromonas veronii [taxid 654] (species)<br>Number of reads: 9357<br>Number of identified reads: 9328 (99.69%)	• species: 357 (3.815%)<br>• **genus: 6573 (70.246%)**<br>• family: 33 (0.352%)<br>• order: 1 (0.01%)<br>• class: 980 (10.473%)<br>• phylum: 219 (2.34%)<br>• superkingdom: 302 (3.227%)<br>• root: 858 (9.169%)	• Aeromonas hydrophila [taxid 644]: 37 (0.395%)<br>• Aeromonas sobria [taxid 646]: 26 (0.277%)<br>• Aeromonas salmonicida [taxid 645]: 23 (0.245%)<br>• Aeromonas enteropelogenes [taxid 29489]: 20 (0.213%)<br>• Aeromonas allosaccharophila [taxid 656]: 13 (0.138%)<br>• Aeromonas media [taxid 651]: 12 (0.128%)<br>• Aeromonas cavernicola [taxid 1006623]: 11 (0.117%)<br>• Escherichia coli [taxid 562]: 10 (0.106%)<br>• other: 78 (0.833%)

Operational Taxonomic Unit (OTU)	Correct identifications	Wrong or overspecific identifications at species rank
Benchmark OTU ID: `BA000002-_Crenarchaeota`<br>OTU taxon: Aeropyrum pernix K1 [taxid 272557]<br>Expected: Aeropyrum pernix [taxid 56636] (species)<br>Number of reads: 3299<br>Number of identified reads: 3110 (94.27%)	• **species: 1384 (41.952%)**<br>• genus: 350 (10.609%)<br>• family: 0 (0.0%)<br>• order: 1 (0.03%)<br>• class: 29 (0.879%)<br>• phylum: 0 (0.0%)<br>• superkingdom: 23 (0.697%)<br>• root: 1242 (37.647%)	• Aeropyrum camini [taxid 229980]: 4 (0.121%)<br>• Cytospora leucostoma [taxid 1230097]: 1 (0.03%)<br>• Pyrenophora teres [taxid 53485]: 1 (0.03%)<br>• Apiotrichum porosum [taxid 105984]: 1 (0.03%)<br>• Mycobacterium montefiorense [taxid 154654]: 1 (0.03%)<br>• Candida parapsilosis [taxid 5480]: 1 (0.03%)<br>• Paraperlucidibaca baekdonensis [taxid 748120]: 1 (0.03%)<br>• Pyrobaculum ferrireducens [taxid 1104324]: 1 (0.03%)<br>• other: 4 (0.121%)
Benchmark OTU ID: `CP003099-_Proteobacteria`<br>OTU taxon: Aggregatibacter actinomycetemcomitans ANH9381 [taxid 754507]<br>Expected: Aggregatibacter actinomycetemcomitans [taxid 714] (species)<br>Number of reads: 3922<br>Number of identified reads: 3890 (99.184%)	• **species: 1911 (48.725%)**<br>• genus: 307 (7.827%)<br>• family: 734 (18.714%)<br>• order: 0 (0.0%)<br>• class: 325 (8.286%)<br>• phylum: 103 (2.626%)<br>• superkingdom: 125 (3.187%)<br>• root: 384 (9.79%)	• Pasteurella multocida [taxid 747]: 5 (0.127%)<br>• Haemophilus haemolyticus [taxid 726]: 3 (0.076%)<br>• Haemophilus parainfluenzae [taxid 729]: 3 (0.076%)<br>• Escherichia coli [taxid 562]: 2 (0.05%)<br>• Rodentibacter myodis [taxid 1907939]: 2 (0.05%)<br>• Histophilus somni [taxid 731]: 2 (0.05%)<br>• Gallibacterium anatis [taxid 750]: 2 (0.05%)<br>• Conservatibacter flavescens [taxid 28161]: 1 (0.025%)<br>• other: 28 (0.713%)
Benchmark OTU ID: `CP001607-_Proteobacteria`<br>OTU taxon: Aggregatibacter aphrophilus NJ8700 [taxid 634176]<br>Expected: Aggregatibacter aphrophilus [taxid 732] (species)<br>Number of reads: 4320<br>Number of identified reads: 4293 (99.375%)	• **species: 1578 (36.527%)**<br>• genus: 687 (15.902%)<br>• family: 884 (20.462%)<br>• order: 0 (0.0%)<br>• class: 468 (10.833%)<br>• phylum: 128 (2.962%)<br>• superkingdom: 155 (3.587%)<br>• root: 390 (9.027%)	• Aggregatibacter actinomycetemcomitans [taxid 714]: 36 (0.833%)<br>• Aggregatibacter segnis [taxid 739]: 11 (0.254%)<br>• Haemophilus parainfluenzae [taxid 729]: 4 (0.092%)<br>• Pasteurella multocida [taxid 747]: 3 (0.069%)<br>• Haemophilus influenzae [taxid 727]: 3 (0.069%)<br>• Escherichia coli [taxid 562]: 3 (0.069%)<br>• Haemophilus haemolyticus [taxid 726]: 3 (0.069%)<br>• Rodentibacter rarus [taxid 1908260]: 2 (0.046%)<br>• other: 36 (0.833%)
Benchmark OTU ID: `AE007870-_Proteobacteria`<br>OTU taxon: Agrobacterium fabrum str. C58 [taxid 176299]<br>Expected: Agrobacterium fabrum [taxid 1176649] (species)<br>Number of reads: 3786<br>Number of identified reads: 3786 (100.0%)	• species: 36 (0.95%)<br>• genus: 1307 (34.521%)<br>• **family: 1548 (40.887%)**<br>• order: 164 (4.331%)<br>• class: 109 (2.879%)<br>• phylum: 88 (2.324%)<br>• superkingdom: 142 (3.75%)<br>• root: 382 (10.089%)	• Agrobacterium tumefaciens [taxid 358]: 63 (1.664%)<br>• Lupinus albus [taxid 3870]: 6 (0.158%)<br>• Agrobacterium deltaense [taxid 1183412]: 5 (0.132%)<br>• Rhizobium pusense [taxid 648995]: 3 (0.079%)<br>• Agrobacterium salinitolerans [taxid 1183413]: 2 (0.052%)<br>• Rhizobium nepotum [taxid 1035271]: 2 (0.052%)<br>• Rhizobium mesoamericanum [taxid 1079800]: 1 (0.026%)<br>• Phyllobacterium phragmitis [taxid 2670329]: 1 (0.026%)<br>• other: 14 (0.369%)

Operational Taxonomic Unit (OTU)	Correct identifications	Wrong or overspecific identifications at species rank
Benchmark OTU ID: `CP000629-_Proteobacteria`<br>OTU taxon: Agrobacterium radiobacter K84 [taxid 311403]<br>Expected: Agrobacterium tumefaciens [taxid 358] (species)<br>Number of reads: 5081<br>Number of identified reads: 5071 (99.803%)	• species: 592 (11.651%)<br>• **genus: 1799 (35.406%)**<br>• family: 1158 (22.79%)<br>• order: 331 (6.514%)<br>• class: 135 (2.656%)<br>• phylum: 198 (3.896%)<br>• superkingdom: 221 (4.349%)<br>• root: 630 (12.399%)	• Agrobacterium rhizogenes [taxid 359]: 11 (0.216%)<br>• Rhizobium leguminosarum [taxid 384]: 5 (0.098%)<br>• Ensifer adhaerens [taxid 106592]: 2 (0.039%)<br>• Rhizobium favelukesii [taxid 348824]: 2 (0.039%)<br>• Parvibaculum sedimenti [taxid 2608632]: 1 (0.019%)<br>• Methanosarcina acetivorans [taxid 2214]: 1 (0.019%)<br>• Parasedimentitalea marina [taxid 2483033]: 1 (0.019%)<br>• Rhizobium tumorigenes [taxid 2041385]: 1 (0.019%)<br>• other: 23 (0.452%)
Benchmark OTU ID: `CP000633-_Proteobacteria`<br>OTU taxon: Agrobacterium vitis S4 [taxid 311402]<br>Expected: Agrobacterium vitis [taxid 373] (species)<br>Number of reads: 7500<br>Number of identified reads: 7486 (99.813%)	• **species: 3734 (49.786%)**<br>• genus: 17 (0.226%)<br>• family: 1614 (21.52%)<br>• order: 453 (6.04%)<br>• class: 267 (3.56%)<br>• phylum: 169 (2.253%)<br>• superkingdom: 232 (3.093%)<br>• root: 990 (13.2%)	• Rhizobium taibaishanense [taxid 887144]: 7 (0.093%)<br>• Lupinus albus [taxid 3870]: 4 (0.053%)<br>• Rhizobium leguminosarum [taxid 384]: 4 (0.053%)<br>• Agrobacterium larrymoorei [taxid 160699]: 2 (0.026%)<br>• Rhizobium rhizosphaerae [taxid 1672749]: 1 (0.013%)<br>• Sinorhizobium meliloti [taxid 382]: 1 (0.013%)<br>• Rhizobium deserti [taxid 2547961]: 1 (0.013%)<br>• Sphingomonas jatrophae [taxid 1166337]: 1 (0.013%)<br>• other: 17 (0.226%)
Benchmark OTU ID: `CP001071-_Verrucomicrobia`<br>OTU taxon: Akkermansia muciniphila ATCC BAA-835 [taxid 349741]<br>Expected: Akkermansia muciniphila [taxid 239935] (species)<br>Number of reads: 177429<br>Number of identified reads: 176958 (99.734%)	• **species: 73965 (41.687%)**<br>• genus: 54424 (30.673%)<br>• family: 0 (0.0%)<br>• order: 743 (0.418%)<br>• class: 142 (0.08%)<br>• phylum: 1060 (0.597%)<br>• superkingdom: 24664 (13.9%)<br>• root: 21803 (12.288%)	• Akkermansia glycaniphila [taxid 1679444]: 91 (0.051%)<br>• Bacteroides ovatus [taxid 28116]: 71 (0.04%)<br>• Gardnerella vaginalis [taxid 2702]: 16 (0.009%)<br>• Beta vulgaris [taxid 161934]: 14 (0.007%)<br>• Bacteroides caccae [taxid 47678]: 6 (0.003%)<br>• Oikopleura dioica [taxid 34765]: 6 (0.003%)<br>• bacterium [taxid 1869227]: 6 (0.003%)<br>• Helicobacter pylori [taxid 210]: 5 (0.002%)<br>• other: 371 (0.209%)
Benchmark OTU ID: `CP003466-_Proteobacteria`<br>OTU taxon: Alcanivorax dieselolei B5 [taxid 930169]<br>Expected: Alcanivorax dieselolei [taxid 285091] (species)<br>Number of reads: 10204<br>Number of identified reads: 10174 (99.705%)	• **species: 3719 (36.446%)**<br>• genus: 3605 (35.329%)<br>• family: 28 (0.274%)<br>• order: 73 (0.715%)<br>• class: 749 (7.34%)<br>• phylum: 588 (5.762%)<br>• superkingdom: 458 (4.488%)<br>• root: 946 (9.27%)	• Salmonella enterica [taxid 28901]: 3 (0.029%)<br>• Alcanivorax pacificus [taxid 1306787]: 2 (0.019%)<br>• Acidisphaera rubrifaciens [taxid 50715]: 2 (0.019%)<br>• Pelagibacterium luteolum [taxid 440168]: 1 (0.009%)<br>• Geothermobacter sp. EPR-M [taxid 1969733]: 1 (0.009%)<br>• Kushneria sinocarnis [taxid 595502]: 1 (0.009%)<br>• Lysobacter capsici [taxid 435897]: 1 (0.009%)<br>• Rhizobium naphthalenivorans [taxid 1118451]: 1 (0.009%)<br>• other: 33 (0.323%)

Operational Taxonomic Unit (OTU)	Correct identifications	Wrong or overspecific identifications at species rank
Benchmark OTU ID: `FP929032-_Bacteroidetes`<br>OTU taxon: Alistipes shahii WAL 8301 [taxid 717959]<br>Expected: Alistipes shahii [taxid 328814] (species)<br>Number of reads: 23181<br>Number of identified reads: 23144 (99.84%)	• species: 2113 (9.115%)<br>• **genus: 14790 (63.802%)**<br>• family: 16 (0.069%)<br>• order: 1922 (8.291%)<br>• class: 37 (0.159%)<br>• phylum: 508 (2.191%)<br>• superkingdom: 1391 (6.0%)<br>• root: 2356 (10.163%)	• Alistipes timonensis [taxid 1465754]: 77 (0.332%)<br>• Alistipes finegoldii [taxid 214856]: 28 (0.12%)<br>• Alistipes putredinis [taxid 28117]: 25 (0.107%)<br>• Alistipes onderdonkii [taxid 328813]: 25 (0.107%)<br>• Bacteroides ovatus [taxid 28116]: 16 (0.069%)<br>• Alistipes dispar [taxid 2585119]: 16 (0.069%)<br>• Alistipes communis [taxid 2585118]: 11 (0.047%)<br>• Bacteroides uniformis [taxid 820]: 7 (0.03%)<br>• other: 96 (0.414%)
Benchmark OTU ID: `CP001896-_Proteobacteria`<br>OTU taxon: Allochromatium vinosum DSM 180 [taxid 572477]<br>Expected: Allochromatium vinosum [taxid 1049] (species)<br>Number of reads: 7052<br>Number of identified reads: 7036 (99.773%)	• **species: 3720 (52.75%)**<br>• genus: 557 (7.898%)<br>• family: 807 (11.443%)<br>• order: 33 (0.467%)<br>• class: 430 (6.097%)<br>• phylum: 424 (6.012%)<br>• superkingdom: 386 (5.473%)<br>• root: 674 (9.557%)	• Allochromatium warmingii [taxid 61595]: 5 (0.07%)<br>• Allochromatium palmeri [taxid 231048]: 5 (0.07%)<br>• Imhoffiella purpurea [taxid 1249627]: 4 (0.056%)<br>• Salmonella enterica [taxid 28901]: 3 (0.042%)<br>• Thiorhodococcus drewsii [taxid 210408]: 3 (0.042%)<br>• Thermochromatium tepidum [taxid 1050]: 3 (0.042%)<br>• Thiocystis violascens [taxid 73141]: 3 (0.042%)<br>• Nitrosomonas oligotropha [taxid 42354]: 1 (0.014%)<br>• other: 23 (0.326%)
Benchmark OTU ID: `CP002339-_Proteobacteria`<br>OTU taxon: Alteromonas naphthalenivorans [taxid 715451]<br>Expected: Alteromonas naphthalenivorans [taxid 715451] (species)<br>Number of reads: 10303<br>Number of identified reads: 10244 (99.427%)	• species: 1240 (12.035%)<br>• **genus: 3334 (32.359%)**<br>• family: 563 (5.464%)<br>• order: 298 (2.892%)<br>• class: 1083 (10.511%)<br>• phylum: 2233 (21.673%)<br>• superkingdom: 462 (4.484%)<br>• root: 1027 (9.967%)	• Alteromonas stellipolaris [taxid 233316]: 17 (0.165%)<br>• Alteromonas australica [taxid 589873]: 7 (0.067%)<br>• Alteromonas macleodii [taxid 28108]: 5 (0.048%)<br>• Alteromonas mediterranea [taxid 314275]: 5 (0.048%)<br>• Alteromonas lipolytica [taxid 1856405]: 3 (0.029%)<br>• Alteromonas confluentis [taxid 1656094]: 3 (0.029%)<br>• Alteromonas sediminis [taxid 2259342]: 3 (0.029%)<br>• Alteromonas marina [taxid 203795]: 3 (0.029%)<br>• other: 61 (0.592%)
Benchmark OTU ID: `CP002000-_Actinobacteria`<br>OTU taxon: Amycolatopsis mediterranei U32 [taxid 749927]<br>Expected: Amycolatopsis mediterranei [taxid 33910] (species)<br>Number of reads: 47757<br>Number of identified reads: 47618 (99.708%)	• **species: 18868 (39.508%)**<br>• genus: 14638 (30.651%)<br>• family: 2036 (4.263%)<br>• order: 18 (0.037%)<br>• class: 5254 (11.001%)<br>• phylum: 40 (0.083%)<br>• superkingdom: 2762 (5.783%)<br>• root: 3968 (8.308%)	• Amycolatopsis tolypomycina [taxid 208445]: 54 (0.113%)<br>• Amycolatopsis australiensis [taxid 546364]: 30 (0.062%)<br>• Amycolatopsis rifamycinica [taxid 287986]: 27 (0.056%)<br>• Amycolatopsis vastitatis [taxid 1905142]: 23 (0.048%)<br>• Amycolatopsis balhimycina [taxid 208443]: 22 (0.046%)<br>• Amycolatopsis vancoresmycina [taxid 208444]: 22 (0.046%)<br>• Amycolatopsis pretoriensis [taxid 218821]: 18 (0.037%)<br>• Amycolatopsis saalfeldensis [taxid 394193]: 15 (0.031%)<br>• other: 275 (0.575%)

Operational Taxonomic Unit (OTU)	Correct identifications	Wrong or overspecific identifications at species rank
Benchmark OTU ID: `CP003659-_Cyanobacteria`<br>OTU taxon: Anabaena cylindrica PCC 7122 [taxid 272123]<br>Expected: Anabaena cylindrica [taxid 1165] (species)<br>Number of reads: 38352<br>Number of identified reads: 37848 (98.685%)	• **species: 20052 (52.284%)**<br>• genus: 152 (0.396%)<br>• family: 2066 (5.386%)<br>• order: 5540 (14.445%)<br>• phylum: 2684 (6.998%)<br>• superkingdom: 1933 (5.04%)<br>• root: 5343 (13.931%)	• Trichormus variabilis [taxid 264691]: 33 (0.086%)<br>• Trichormus azollae [taxid 1164]: 30 (0.078%)<br>• Cylindrospermum stagnale [taxid 142864]: 22 (0.057%)<br>• Dolichospermum flos-aquae [taxid 1166]: 16 (0.041%)<br>• Sphaerospermopsis reniformis [taxid 531300]: 15 (0.039%)<br>• Nostoc sphaeroides [taxid 446679]: 14 (0.036%)<br>• Aphanizomenon flos-aquae [taxid 1176]: 10 (0.026%)<br>• other: 203 (0.529%)
Benchmark OTU ID: `AP012029-_Chloroflexi`<br>OTU taxon: Anaerolinea thermophila UNI-1 [taxid 926569]<br>Expected: Anaerolinea thermophila [taxid 167964] (species)<br>Number of reads: 85944<br>Number of identified reads: 85732 (99.753%)	• **species: 64734 (75.321%)**<br>• genus: 579 (0.673%)<br>• family: 861 (1.001%)<br>• order: 60 (0.069%)<br>• class: 378 (0.439%)<br>• phylum: 2520 (2.932%)<br>• superkingdom: 7722 (8.984%)<br>• root: 8829 (10.272%)	• Anaerolinea thermolimosa [taxid 229919]: 16 (0.018%)<br>• Bellilinea caldifistulae [taxid 360411]: 10 (0.011%)<br>• Thermanaerothrix daxensis [taxid 869279]: 6 (0.006%)<br>• Levilinea saccharolytica [taxid 229921]: 4 (0.004%)<br>• Helicobacter pylori [taxid 210]: 3 (0.003%)<br>• Ornatilinea apprima [taxid 1134406]: 3 (0.003%)<br>• Leptolinea tardivitalis [taxid 229920]: 3 (0.003%)<br>• bacterium [taxid 1869227]: 3 (0.003%)<br>• other: 115 (0.133%)
Benchmark OTU ID: `CP001359-_Proteobacteria`<br>OTU taxon: Anaeromyxobacter dehalogenans 2CP-1 [taxid 455488]<br>Expected: Anaeromyxobacter dehalogenans [taxid 161493] (species)<br>Number of reads: 10432<br>Number of identified reads: 10380 (99.501%)	• species: 2282 (21.875%)<br>• **genus: 5619 (53.863%)**<br>• family: 0 (0.0%)<br>• order: 57 (0.546%)<br>• class: 92 (0.881%)<br>• phylum: 449 (4.304%)<br>• superkingdom: 1072 (10.276%)<br>• root: 796 (7.63%)	• Anaeromyxobacter sp. K [taxid 447217]: 40 (0.383%)<br>• Mycobacterium ahvazicum [taxid 1964395]: 1 (0.009%)<br>• bacterium [taxid 1869227]: 1 (0.009%)<br>• Aliarcobacter faecis [taxid 1564138]: 1 (0.009%)<br>• Desulfatirhabdium butyrativorans [taxid 340467]: 1 (0.009%)<br>• Durinskia baltica [taxid 400756]: 1 (0.009%)<br>• Pseudomonas fuscovaginae [taxid 50340]: 1 (0.009%)<br>• Mycolicibacterium confluentis [taxid 28047]: 1 (0.009%)<br>• other: 12 (0.115%)
Benchmark OTU ID: `FP929061-_Firmicutes`<br>OTU taxon: Anaerostipes hadrus [taxid 649756]<br>Expected: Anaerostipes hadrus [taxid 649756] (species)<br>Number of reads: 4012<br>Number of identified reads: 3978 (99.152%)	• species: 111 (2.766%)<br>• genus: 6 (0.149%)<br>• family: 170 (4.237%)<br>• **order: 2633 (65.628%)**<br>• class: 8 (0.199%)<br>• phylum: 571 (14.232%)<br>• superkingdom: 161 (4.012%)<br>• root: 318 (7.926%)	• Anaerostipes rhamnosivorans [taxid 1229621]: 2 (0.049%)<br>• [Eubacterium] rectale [taxid 39491]: 2 (0.049%)<br>• Clostridioides difficile [taxid 1496]: 2 (0.049%)<br>• Eubacterium ventriosum [taxid 39496]: 2 (0.049%)<br>• [Clostridium] aminophilum [taxid 1526]: 1 (0.024%)<br>• Hungatella hathewayi [taxid 154046]: 1 (0.024%)<br>• Clostridium beijerinckii [taxid 1520]: 1 (0.024%)<br>• Butyrivibrio proteoclasticus [taxid 43305]: 1 (0.024%)<br>• other: 15 (0.373%)

Operational Taxonomic Unit (OTU)	Correct identifications	Wrong or overspecific identifications at species rank
Benchmark OTU ID: `CP001079-_Proteobacteria`<br>OTU taxon: Anaplasma marginale str. Florida [taxid 320483]<br>Expected: Anaplasma marginale [taxid 770] (species)<br>Number of reads: 1821<br>Number of identified reads: 1810 (99.395%)	• **species: 771 (42.339%)**<br>• genus: 357 (19.604%)<br>• family: 25 (1.372%)<br>• order: 3 (0.164%)<br>• class: 23 (1.263%)<br>• phylum: 20 (1.098%)<br>• superkingdom: 112 (6.15%)<br>• root: 493 (27.073%)	• Anaplasma phagocytophilum [taxid 948]: 2 (0.109%)<br>• Anaplasma centrale [taxid 769]: 2 (0.109%)<br>• Pararhodospirillum photometricum [taxid 1084]: 1 (0.054%)<br>• Rubripirellula reticaptiva [taxid 2528013]: 1 (0.054%)<br>• Anaplasma ovis [taxid 142058]: 1 (0.054%)<br>• Daphnia dolichocephala [taxid 2282166]: 1 (0.054%)<br>• Anthracocystis flocculosa [taxid 84751]: 1 (0.054%)<br>• Nitrosococcus wardiae [taxid 1814290]: 1 (0.054%)<br>• other: 4 (0.219%)
Benchmark OTU ID: `CP000030-_Proteobacteria`<br>OTU taxon: Anaplasma marginale str. St. Maries [taxid 234826]<br>Expected: Anaplasma marginale [taxid 770] (species)<br>Number of reads: 1811<br>Number of identified reads: 1803 (99.558%)	• **species: 728 (40.198%)**<br>• genus: 396 (21.866%)<br>• family: 24 (1.325%)<br>• order: 4 (0.22%)<br>• class: 24 (1.325%)<br>• phylum: 21 (1.159%)<br>• superkingdom: 85 (4.693%)<br>• root: 513 (28.326%)	• Anaplasma centrale [taxid 769]: 13 (0.717%)<br>• Anaplasma ovis [taxid 142058]: 2 (0.11%)<br>• Hevea brasiliensis [taxid 3981]: 1 (0.055%)<br>• Phytophthora parasitica [taxid 4792]: 1 (0.055%)<br>• Wolbachia endosymbiont of Folsomia candida [taxid 169402]: 1 (0.055%)<br>• Mycoplasma dispar [taxid 86660]: 1 (0.055%)
Benchmark OTU ID: `CP000235-_Proteobacteria`<br>OTU taxon: Anaplasma phagocytophilum str. HZ [taxid 212042]<br>Expected: Anaplasma phagocytophilum [taxid 948] (species)<br>Number of reads: 2426<br>Number of identified reads: 2388 (98.433%)	• **species: 1728 (71.228%)**<br>• genus: 13 (0.535%)<br>• family: 31 (1.277%)<br>• order: 0 (0.0%)<br>• class: 17 (0.7%)<br>• phylum: 29 (1.195%)<br>• superkingdom: 48 (1.978%)<br>• root: 519 (21.393%)	• Treponema socranskii [taxid 53419]: 2 (0.082%)<br>• Candidatus Neoehrlichia lotoris [taxid 467750]: 1 (0.041%)<br>• Hevea brasiliensis [taxid 3981]: 1 (0.041%)<br>• Tritrichomonas foetus [taxid 1144522]: 1 (0.041%)<br>• Trachymyrmex cornetzi [taxid 471704]: 1 (0.041%)
Benchmark OTU ID: `chr2-_Eukaryotes`<br>OTU taxon: Anolis carolinensis [taxid 28377]<br>Expected: Anolis carolinensis [taxid 28377] (species)<br>Number of reads: 194345<br>Number of identified reads: 181567 (93.425%)	• species: 11508 (5.921%)<br>• genus: 0 (0.0%)<br>• family: 0 (0.0%)<br>• order: 1138 (0.585%)<br>• class: 0 (0.0%)<br>• phylum: 2236 (1.15%)<br>• superkingdom: 3530 (1.816%)<br>• **root: 161244 (82.967%)**	• Podarcis muralis [taxid 64176]: 276 (0.142%)<br>• Limosa lapponica [taxid 161683]: 35 (0.018%)<br>• Paroedura picta [taxid 143630]: 18 (0.009%)<br>• Hirundo rustica [taxid 43150]: 13 (0.006%)<br>• Bos indicus x Bos taurus [taxid 30522]: 12 (0.006%)<br>• Pelodiscus sinensis [taxid 13735]: 11 (0.005%)<br>• Muntiacus muntjak [taxid 9888]: 11 (0.005%)<br>• Sipha flava [taxid 143950]: 10 (0.005%)<br>• other: 781 (0.401%)

Operational Taxonomic Unit (OTU)	Correct identifications	Wrong or overspecific identifications at species rank
Benchmark OTU ID: `CP000922-_Firmicutes`<br>OTU taxon: Anoxybacillus flavithermus WK1 [taxid 491915]<br>Expected: Anoxybacillus flavithermus [taxid 33934] (species)<br>Number of reads: 3786<br>Number of identified reads: 3755 (99.181%)	• species: 952 (25.145%)<br>• genus: 672 (17.749%)<br>• **family: 1368 (36.133%)**<br>• order: 168 (4.437%)<br>• class: 31 (0.818%)<br>• phylum: 44 (1.162%)<br>• superkingdom: 177 (4.675%)<br>• root: 341 (9.006%)	• Anoxybacillus suryakundensis [taxid 1325335]: 6 (0.158%)<br>• Anoxybacillus pushchinoensis [taxid 150248]: 4 (0.105%)<br>• Anoxybacillus amylolyticus [taxid 294699]: 3 (0.079%)<br>• Anoxybacillus vitaminiphilus [taxid 581036]: 3 (0.079%)<br>• Anoxybacillus gonensis [taxid 198467]: 3 (0.079%)<br>• Geobacillus subterraneus [taxid 129338]: 2 (0.052%)<br>• Anoxybacillus ayderensis [taxid 265546]: 2 (0.052%)<br>• Lysinibacillus mangiferihumi [taxid 1130819]: 1 (0.026%)<br>• other: 25 (0.66%)
Benchmark OTU ID: `Arabidopsis_chr4-_Eukaryotes`<br>OTU taxon: Arabidopsis thaliana [taxid 3702]<br>Expected: Arabidopsis thaliana [taxid 3702] (species)<br>Number of reads: 242004<br>Number of identified reads: 219918 (90.873%)	• species: 49903 (20.62%)<br>• genus: 2667 (1.102%)<br>• family: 10367 (4.283%)<br>• order: 2 (0.0%)<br>• class: 6939 (2.867%)<br>• phylum: 88 (0.036%)<br>• superkingdom: 2710 (1.119%)<br>• **root: 145483 (60.115%)**	• Microthlaspi erraticum [taxid 1685480]: 449 (0.185%)<br>• Arabidopsis lyrata [taxid 59689]: 254 (0.104%)<br>• Tanacetum cinerariifolium [taxid 118510]: 141 (0.058%)<br>• Raphanus sativus [taxid 3726]: 135 (0.055%)<br>• Noccaea caerulescens [taxid 107243]: 106 (0.043%)<br>• Capsella rubella [taxid 81985]: 103 (0.042%)<br>• Brassica oleracea [taxid 3712]: 87 (0.035%)<br>• Fagus sylvatica [taxid 28930]: 58 (0.023%)<br>• other: 1238 (0.511%)
Benchmark OTU ID: `CP005290-_Euryarchaeota`<br>OTU taxon: Archaeoglobus sulfaticallidus PM70-1 [taxid 387631]<br>Expected: Archaeoglobus sulfaticallidus [taxid 1316941] (species)<br>Number of reads: 1784<br>Number of identified reads: 1773 (99.383%)	• **species: 1107 (62.051%)**<br>• genus: 10 (0.56%)<br>• family: 10 (0.56%)<br>• order: 29 (1.625%)<br>• class: 3 (0.168%)<br>• phylum: 16 (0.896%)<br>• superkingdom: 15 (0.84%)<br>• root: 561 (31.446%)	• Astyanax mexicanus [taxid 7994]: 1 (0.056%)<br>• Archaeoglobales archaeon [taxid 2250258]: 1 (0.056%)<br>• Candidatus Thiomargarita nelsonii [taxid 1003181]: 1 (0.056%)
Benchmark OTU ID: `AP012047-_Proteobacteria`<br>OTU taxon: Arcobacter butzleri ED-1 [taxid 944546]<br>Expected: Arcobacter butzleri [taxid 28197] (species)<br>Number of reads: 4193<br>Number of identified reads: 4149 (98.95%)	• species: 803 (19.15%)<br>• genus: 68 (1.621%)<br>• **family: 1617 (38.564%)**<br>• order: 146 (3.481%)<br>• class: 13 (0.31%)<br>• phylum: 196 (4.674%)<br>• superkingdom: 342 (8.156%)<br>• root: 956 (22.799%)	• Pseudoarcobacter acticola [taxid 1849015]: 5 (0.119%)<br>• Helicobacter pylori [taxid 210]: 4 (0.095%)<br>• Arcobacter cryaerophilus [taxid 28198]: 3 (0.071%)<br>• Arcobacter ellisii [taxid 913109]: 2 (0.047%)<br>• Malaciobacter molluscorum [taxid 1032072]: 2 (0.047%)<br>• Pseudoarcobacter caeni [taxid 1912877]: 2 (0.047%)<br>• Campylobacter jejuni [taxid 197]: 2 (0.047%)<br>• Acinetobacter bouvetii [taxid 202951]: 1 (0.023%)<br>• other: 42 (1.001%)

Operational Taxonomic Unit (OTU)	Correct identifications	Wrong or overspecific identifications at species rank
Benchmark OTU ID: `CP001999-_Proteobacteria`<br>OTU taxon: Arcobacter nitrofigilis DSM 7299 [taxid 572480]<br>Expected: Arcobacter nitrofigilis [taxid 28199] (species)<br>Number of reads: 6298<br>Number of identified reads: 6236 (99.015%)	• **species: 3408 (54.112%)**<br>• genus: 147 (2.334%)<br>• family: 1701 (27.008%)<br>• order: 110 (1.746%)<br>• class: 21 (0.333%)<br>• phylum: 198 (3.143%)<br>• superkingdom: 211 (3.35%)<br>• root: 435 (6.906%)	• Sulfurospirillum halorespirans [taxid 194424]: 2 (0.031%)<br>• Pseudoarcobacter caeni [taxid 1912877]: 2 (0.031%)<br>• Helicobacter pylori [taxid 210]: 1 (0.015%)<br>• Helicobacter mustelae [taxid 217]: 1 (0.015%)<br>• Arcobacter cibarius [taxid 255507]: 1 (0.015%)<br>• Malaciobacter molluscorum [taxid 1032072]: 1 (0.015%)<br>• Wolinella succinogenes [taxid 844]: 1 (0.015%)<br>• Candidatus Adiutrix intracellularis [taxid 1705730]: 1 (0.015%)<br>• other: 13 (0.206%)
Benchmark OTU ID: `CR555306-_Proteobacteria`<br>OTU taxon: Aromatoleum aromaticum EbN1 [taxid 76114]<br>Expected: Aromatoleum aromaticum [taxid 551760] (species)<br>Number of reads: 8782<br>Number of identified reads: 8756 (99.703%)	• **species: 3983 (45.354%)**<br>• genus: 6 (0.068%)<br>• family: 15 (0.17%)<br>• order: 1759 (20.029%)<br>• class: 752 (8.562%)<br>• phylum: 887 (10.1%)<br>• superkingdom: 448 (5.101%)<br>• root: 900 (10.248%)	• Azoarcus sp. CIB [taxid 198107]: 7 (0.079%)<br>• Candidatus Accumulibacter phosphatis [taxid 327160]: 2 (0.022%)<br>• Thauera chlorobenzoica [taxid 96773]: 2 (0.022%)<br>• Thauera aromatica [taxid 59405]: 2 (0.022%)<br>• Taylorella asinigenitalis [taxid 84590]: 2 (0.022%)<br>• Rhodanobacter glycinis [taxid 582702]: 1 (0.011%)<br>• Conchiformibius steedae [taxid 153493]: 1 (0.011%)<br>• Chromobacterium vaccinii [taxid 1108595]: 1 (0.011%)<br>• other: 43 (0.489%)
Benchmark OTU ID: `CP003203-_Actinobacteria`<br>OTU taxon: Arthrobacter sp. Rue61a [taxid 1118963]<br>Expected: Arthrobacter [taxid 1663] (genus)<br>Number of reads: 20805<br>Number of identified reads: 20669 (99.346%)	• genus: 2802 (13.467%)<br>• **family: 12637 (60.74%)**<br>• order: 497 (2.388%)<br>• class: 956 (4.595%)<br>• phylum: 7 (0.033%)<br>• superkingdom: 1085 (5.215%)<br>• root: 2654 (12.756%)	• Paenarthrobacter aurescens [taxid 43663]: 124 (0.596%)<br>• Arthrobacter sp. YN [taxid 2020486]: 90 (0.432%)<br>• Paenarthrobacter nitroguajacolicus [taxid 211146]: 17 (0.081%)<br>• Arthrobacter cupressi [taxid 1045773]: 11 (0.052%)<br>• Paenarthrobacter nicotinovorans [taxid 29320]: 8 (0.038%)<br>• Arthrobacter sp. UCD-GKA [taxid 1913576]: 5 (0.024%)<br>• Renibacterium salmoninarum [taxid 1646]: 5 (0.024%)<br>• other: 141 (0.677%)
Benchmark OTU ID: `AP011615-_Cyanobacteria`<br>OTU taxon: Arthrospira platensis NIES-39 [taxid 696747]<br>Expected: Arthrospira platensis [taxid 118562] (species)<br>Number of reads: 40276<br>Number of identified reads: 39704 (98.579%)	• **species: 10241 (25.427%)**<br>• genus: 9083 (22.551%)<br>• family: 9631 (23.912%)<br>• order: 663 (1.646%)<br>• phylum: 2134 (5.298%)<br>• superkingdom: 1969 (4.888%)<br>• root: 5925 (14.71%)	• Limnospira indica [taxid 147322]: 137 (0.34%)<br>• Limnospira maxima [taxid 129910]: 103 (0.255%)<br>• Limnospira fusiformis [taxid 54297]: 38 (0.094%)<br>• Planktothrix agardhii [taxid 1160]: 5 (0.012%)<br>• Microcystis aeruginosa [taxid 1126]: 4 (0.009%)<br>• Planktothrix rubescens [taxid 59512]: 3 (0.007%)<br>• Tychonema bourrellyi [taxid 54313]: 3 (0.007%)<br>• other: 70 (0.173%)
Benchmark OTU ID: `CP002395-_Proteobacteria`<br>OTU taxon: Asticcacaulis excentricus CB 48 [taxid 573065]<br>Expected: Asticcacaulis excentricus [taxid 78587] (species)<br>Number of reads: 4939<br>Number of identified reads: 4928 (99.777%)	• **species: 3108 (62.927%)**<br>• genus: 608 (12.31%)<br>• family: 52 (1.052%)<br>• order: 9 (0.182%)<br>• class: 222 (4.494%)<br>• phylum: 150 (3.037%)<br>• superkingdom: 217 (4.393%)<br>• root: 557 (11.277%)	• Asticcacaulis benevestitus [taxid 347481]: 3 (0.06%)<br>• Sinorhizobium fredii [taxid 380]: 1 (0.02%)<br>• Paraburkholderia unamae [taxid 219649]: 1 (0.02%)<br>• Pedobacter lusitanus [taxid 1503925]: 1 (0.02%)<br>• Fusarium coffeatum [taxid 231269]: 1 (0.02%)<br>• Brevundimonas abyssalis [taxid 1125965]: 1 (0.02%)<br>• Legionella quinlivanii [taxid 45073]: 1 (0.02%)<br>• Thermoflexus hugenholtzii [taxid 1495650]: 1 (0.02%)

Operational Taxonomic Unit (OTU)	Correct identifications	Wrong or overspecific identifications at species rank
Benchmark OTU ID: `CP002396-_Proteobacteria`<br>OTU taxon: Asticcacaulis excentricus CB 48 [taxid 573065]<br>Expected: Asticcacaulis excentricus [taxid 78587] (species)<br>Number of reads: 2077<br>Number of identified reads: 2069 (99.614%)	• **species: 1307 (62.927%)**<br>• genus: 245 (11.795%)<br>• family: 27 (1.299%)<br>• order: 2 (0.096%)<br>• class: 98 (4.718%)<br>• phylum: 68 (3.273%)<br>• superkingdom: 77 (3.707%)<br>• root: 236 (11.362%)	• Tanacetum cinerariifolium [taxid 118510]: 1 (0.048%)<br>• Coprococcus catus [taxid 116085]: 1 (0.048%)<br>• Cupriavidus oxalaticus [taxid 96344]: 1 (0.048%)<br>• Mikania micrantha [taxid 192012]: 1 (0.048%)<br>• Rhipicephalus microplus [taxid 6941]: 1 (0.048%)<br>• Pseudarcicella hirudinis [taxid 1079859]: 1 (0.048%)<br>• Asticcacaulis benevestitus [taxid 347481]: 1 (0.048%)
Benchmark OTU ID: `CP001721-_Actinobacteria`<br>OTU taxon: Atopobium parvulum DSM 20469 [taxid 521095]<br>Expected: Lancefieldella parvula [taxid 1382] (species)<br>Number of reads: 5162<br>Number of identified reads: 5148 (99.728%)	• **species: 2362 (45.757%)**<br>• genus: 50 (0.968%)<br>• family: 1410 (27.314%)<br>• order: 135 (2.615%)<br>• class: 38 (0.736%)<br>• phylum: 94 (1.82%)<br>• superkingdom: 475 (9.201%)<br>• root: 579 (11.216%)	• Lancefieldella rimae [taxid 1383]: 3 (0.058%)<br>• Collinsella aerofaciens [taxid 74426]: 1 (0.019%)<br>• Mesocestoides corti [taxid 53468]: 1 (0.019%)<br>• Candidatus Nitrosotalea okcheonensis [taxid 1903276]: 1 (0.019%)<br>• Chondromyces crocatus [taxid 52]: 1 (0.019%)<br>• Ulvibacter antarcticus [taxid 442714]: 1 (0.019%)<br>• Helicobacter pylori [taxid 210]: 1 (0.019%)<br>• Burkholderia vietnamiensis [taxid 60552]: 1 (0.019%)<br>• other: 4 (0.077%)
Benchmark OTU ID: `AP009384-_Proteobacteria`<br>OTU taxon: Azorhizobium caulinodans ORS 571 [taxid 438753]<br>Expected: Azorhizobium caulinodans [taxid 7] (species)<br>Number of reads: 11198<br>Number of identified reads: 11144 (99.517%)	• **species: 7193 (64.234%)**<br>• genus: 25 (0.223%)<br>• family: 105 (0.937%)<br>• order: 1074 (9.59%)<br>• class: 604 (5.393%)<br>• phylum: 592 (5.286%)<br>• superkingdom: 591 (5.277%)<br>• root: 954 (8.519%)	• Xanthobacter autotrophicus [taxid 280]: 1 (0.008%)<br>• Aureimonas fodinaquatilis [taxid 2565783]: 1 (0.008%)<br>• Aquabacter spiritensis [taxid 933073]: 1 (0.008%)<br>• Thalassocella blandensis [taxid 2584524]: 1 (0.008%)<br>• Achromobacter aloeverae [taxid 1750518]: 1 (0.008%)<br>• Nocardia brasiliensis [taxid 37326]: 1 (0.008%)<br>• Nelumbo nucifera [taxid 4432]: 1 (0.008%)<br>• Notoacmeibacter ruber [taxid 2670375]: 1 (0.008%)<br>• other: 38 (0.339%)
Benchmark OTU ID: `HE577327-_Proteobacteria`<br>OTU taxon: Azospirillum baldaniorum [taxid 1064539]<br>Expected: Azospirillum baldaniorum [taxid 1064539] (species)<br>Number of reads: 5904<br>Number of identified reads: 5879 (99.576%)	• species: 95 (1.609%)<br>• **genus: 4402 (74.559%)**<br>• family: 89 (1.507%)<br>• order: 23 (0.389%)<br>• class: 321 (5.436%)<br>• phylum: 227 (3.844%)<br>• superkingdom: 284 (4.81%)<br>• root: 432 (7.317%)	• **Azospirillum brasilense [taxid 192]: 218 (3.692%)**<br>• Azospirillum lipoferum [taxid 193]: 3 (0.05%)<br>• Azospirillum doebereinerae [taxid 92933]: 2 (0.033%)<br>• Azospirillum thermophilum [taxid 2202148]: 2 (0.033%)<br>• Azospirillum thiophilum [taxid 528244]: 2 (0.033%)<br>• Ferruginivarius sediminum [taxid 2661937]: 1 (0.016%)<br>• Arsenophonus endosymbiont of Aleurodicus floccissimus [taxid 2152761]: 1 (0.016%)<br>• Moraxella atlantae [taxid 34059]: 1 (0.016%)<br>• other: 10 (0.169%)

Operational Taxonomic Unit (OTU)	Correct identifications	Wrong or overspecific identifications at species rank
Benchmark OTU ID: `FQ311868-_Proteobacteria`<br>OTU taxon: Azospirillum lipoferum 4B [taxid 862719]<br>Expected: Azospirillum lipoferum [taxid 193] (species)<br>Number of reads: 5840<br>Number of identified reads: 5808 (99.452%)	• species: 1363 (23.339%)<br>• **genus: 2963 (50.736%)**<br>• family: 81 (1.386%)<br>• order: 13 (0.222%)<br>• class: 335 (5.736%)<br>• phylum: 206 (3.527%)<br>• superkingdom: 263 (4.503%)<br>• root: 574 (9.828%)	• Azospirillum brasilense [taxid 192]: 10 (0.171%)<br>• Azospirillum humicireducens [taxid 1226968]: 7 (0.119%)<br>• Lupinus albus [taxid 3870]: 5 (0.085%)<br>• Azospirillum thiophilum [taxid 528244]: 4 (0.068%)<br>• Azospirillum thermophilum [taxid 2202148]: 4 (0.068%)<br>• Azospirillum doebereinerae [taxid 92933]: 4 (0.068%)<br>• Azospirillum ramasamyi [taxid 682998]: 4 (0.068%)<br>• Azospirillum oryzae [taxid 286727]: 4 (0.068%)<br>• other: 19 (0.325%)
Benchmark OTU ID: `AP010946-_Proteobacteria`<br>OTU taxon: Azospirillum sp. B510 [taxid 137722]<br>Expected: Azospirillum lipoferum [taxid 193] (species)<br>Number of reads: 6566<br>Number of identified reads: 6539 (99.588%)	• **species: 2816 (42.887%)**<br>• genus: 1971 (30.018%)<br>• family: 91 (1.385%)<br>• order: 37 (0.563%)<br>• class: 388 (5.909%)<br>• phylum: 241 (3.67%)<br>• superkingdom: 318 (4.843%)<br>• root: 673 (10.249%)	• Azospirillum thiophilum [taxid 528244]: 4 (0.06%)<br>• Azospirillum brasilense [taxid 192]: 4 (0.06%)<br>• Lupinus albus [taxid 3870]: 3 (0.045%)<br>• Azospirillum palustre [taxid 2044885]: 3 (0.045%)<br>• Azospirillum doebereinerae [taxid 92933]: 2 (0.03%)<br>• Pseudobythopirellula maris [taxid 2527991]: 1 (0.015%)<br>• Azospirillum oryzae [taxid 286727]: 1 (0.015%)<br>• Acidisarcina polymorpha [taxid 2211140]: 1 (0.015%)<br>• other: 23 (0.35%)
Benchmark OTU ID: `FN597644-_Firmicutes`<br>OTU taxon: Bacillus amyloliquefaciens DSM 7 = ATCC 23350 [taxid 692420]<br>Expected: Bacillus amyloliquefaciens [taxid 1390] (species)<br>Number of reads: 5599<br>Number of identified reads: 5577 (99.607%)	• species: 1479 (26.415%)<br>• **genus: 2866 (51.187%)**<br>• family: 189 (3.375%)<br>• order: 156 (2.786%)<br>• class: 52 (0.928%)<br>• phylum: 51 (0.91%)<br>• superkingdom: 194 (3.464%)<br>• root: 585 (10.448%)	• Bacillus velezensis [taxid 492670]: 24 (0.428%)<br>• Bacillus nakamurai [taxid 1793963]: 14 (0.25%)<br>• Bacillus subtilis [taxid 1423]: 11 (0.196%)<br>• Bacillus atrophaeus [taxid 1452]: 6 (0.107%)<br>• Bacillus pumilus [taxid 1408]: 2 (0.035%)<br>• Bacillus siamensis [taxid 659243]: 2 (0.035%)<br>• Bacillus gobiensis [taxid 1441095]: 2 (0.035%)<br>• Bacillus lacisalsi [taxid 2045244]: 2 (0.035%)<br>• other: 20 (0.357%)
Benchmark OTU ID: `CP002634-_Firmicutes`<br>OTU taxon: Bacillus amyloliquefaciens LL3 [taxid 1001582]<br>Expected: Bacillus amyloliquefaciens [taxid 1390] (species)<br>Number of reads: 5623<br>Number of identified reads: 5590 (99.413%)	• species: 1460 (25.964%)<br>• **genus: 2877 (51.164%)**<br>• family: 174 (3.094%)<br>• order: 159 (2.827%)<br>• class: 37 (0.658%)<br>• phylum: 37 (0.658%)<br>• superkingdom: 211 (3.752%)<br>• root: 628 (11.168%)	• Bacillus velezensis [taxid 492670]: 54 (0.96%)<br>• Bacillus subtilis [taxid 1423]: 7 (0.124%)<br>• Bacillus atrophaeus [taxid 1452]: 5 (0.088%)<br>• Bacillus siamensis [taxid 659243]: 5 (0.088%)<br>• Bacillus nakamurai [taxid 1793963]: 4 (0.071%)<br>• Bacillus stratosphericus [taxid 293386]: 2 (0.035%)<br>• Thalassobacillus cyri [taxid 571932]: 2 (0.035%)<br>• Bacillus vallismortis [taxid 72361]: 2 (0.035%)<br>• other: 32 (0.569%)

Operational Taxonomic Unit (OTU)	Correct identifications	Wrong or overspecific identifications at species rank
Benchmark OTU ID: `CP002627-_Firmicutes`<br>OTU taxon: Bacillus amyloliquefaciens TA208 [taxid 999891]<br>Expected: Bacillus amyloliquefaciens [taxid 1390] (species)<br>Number of reads: 5531<br>Number of identified reads: 5517 (99.746%)	• species: 1376 (24.877%)<br>• **genus: 2901 (52.449%)**<br>• family: 167 (3.019%)<br>• order: 152 (2.748%)<br>• class: 34 (0.614%)<br>• phylum: 30 (0.542%)<br>• superkingdom: 200 (3.615%)<br>• root: 652 (11.788%)	• Bacillus velezensis [taxid 492670]: 39 (0.705%)<br>• Bacillus subtilis [taxid 1423]: 17 (0.307%)<br>• Bacillus nakamurai [taxid 1793963]: 13 (0.235%)<br>• Bacillus siamensis [taxid 659243]: 10 (0.18%)<br>• Bacillus atrophaeus [taxid 1452]: 4 (0.072%)<br>• Bacillus sp. JS [taxid 1127744]: 3 (0.054%)<br>• Bacillus daliensis [taxid 745820]: 2 (0.036%)<br>• Bacillus oleivorans [taxid 1448271]: 2 (0.036%)<br>• other: 26 (0.47%)
Benchmark OTU ID: `AE016879-_Firmicutes`<br>OTU taxon: Bacillus anthracis str. Ames [taxid 198094]<br>Expected: Bacillus anthracis [taxid 1392] (species)<br>Number of reads: 7595<br>Number of identified reads: 7490 (98.617%)	• species: 342 (4.502%)<br>• **genus: 6106 (80.394%)**<br>• family: 94 (1.237%)<br>• order: 98 (1.29%)<br>• class: 37 (0.487%)<br>• phylum: 53 (0.697%)<br>• superkingdom: 224 (2.949%)<br>• root: 528 (6.951%)	• **Bacillus cereus [taxid 1396]: 164 (2.159%)**<br>• Bacillus thuringiensis [taxid 1428]: 57 (0.75%)<br>• Bacillus wiedmannii [taxid 1890302]: 27 (0.355%)<br>• Bacillus mycoides [taxid 1405]: 18 (0.236%)<br>• Bacillus toyonensis [taxid 155322]: 9 (0.118%)<br>• Bacillus cytotoxicus [taxid 580165]: 9 (0.118%)<br>• Bacillus manliponensis [taxid 574376]: 8 (0.105%)<br>• Bacillus pacificus [taxid 2026187]: 5 (0.065%)<br>• other: 47 (0.618%)
Benchmark OTU ID: `CP002091-_Firmicutes`<br>OTU taxon: Bacillus anthracis str. H9401 [taxid 768494]<br>Expected: Bacillus anthracis [taxid 1392] (species)<br>Number of reads: 7581<br>Number of identified reads: 7469 (98.522%)	• species: 300 (3.957%)<br>• **genus: 6123 (80.767%)**<br>• family: 96 (1.266%)<br>• order: 81 (1.068%)<br>• class: 32 (0.422%)<br>• phylum: 59 (0.778%)<br>• superkingdom: 221 (2.915%)<br>• root: 548 (7.228%)	• Bacillus cereus [taxid 1396]: 140 (1.846%)<br>• Bacillus thuringiensis [taxid 1428]: 50 (0.659%)<br>• Bacillus wiedmannii [taxid 1890302]: 28 (0.369%)<br>• Bacillus toyonensis [taxid 155322]: 12 (0.158%)<br>• Bacillus mycoides [taxid 1405]: 12 (0.158%)<br>• Bacillus cytotoxicus [taxid 580165]: 9 (0.118%)<br>• Bacillus pseudomycoides [taxid 64104]: 6 (0.079%)<br>• Bacillus gaemokensis [taxid 574375]: 6 (0.079%)<br>• other: 50 (0.659%)
Benchmark OTU ID: `AE017225-_Firmicutes`<br>OTU taxon: Bacillus anthracis str. Sterne [taxid 260799]<br>Expected: Bacillus anthracis [taxid 1392] (species)<br>Number of reads: 7597<br>Number of identified reads: 7486 (98.538%)	• species: 319 (4.199%)<br>• **genus: 6088 (80.136%)**<br>• family: 98 (1.289%)<br>• order: 106 (1.395%)<br>• class: 41 (0.539%)<br>• phylum: 41 (0.539%)<br>• superkingdom: 229 (3.014%)<br>• root: 560 (7.371%)	• **Bacillus cereus [taxid 1396]: 164 (2.158%)**<br>• Bacillus thuringiensis [taxid 1428]: 41 (0.539%)<br>• Bacillus wiedmannii [taxid 1890302]: 19 (0.25%)<br>• Bacillus mycoides [taxid 1405]: 14 (0.184%)<br>• Bacillus cytotoxicus [taxid 580165]: 12 (0.157%)<br>• Bacillus toyonensis [taxid 155322]: 8 (0.105%)<br>• Bacillus pseudomycoides [taxid 64104]: 4 (0.052%)<br>• Bacillus manliponensis [taxid 574376]: 4 (0.052%)<br>• other: 41 (0.539%)

Operational Taxonomic Unit (OTU)	Correct identifications	Wrong or overspecific identifications at species rank
Benchmark OTU ID: `CP002207-_Firmicutes`<br>OTU taxon: Bacillus atrophaeus 1942 [taxid 720555]<br>Expected: Bacillus atrophaeus [taxid 1452] (species)<br>Number of reads: 5900<br>Number of identified reads: 5864 (99.389%)	• **species: 3226 (54.677%)**<br>• genus: 1237 (20.966%)<br>• family: 201 (3.406%)<br>• order: 161 (2.728%)<br>• class: 53 (0.898%)<br>• phylum: 57 (0.966%)<br>• superkingdom: 267 (4.525%)<br>• root: 658 (11.152%)	• Bacillus subtilis [taxid 1423]: 8 (0.135%)<br>• Bacillus amyloliquefaciens [taxid 1390]: 4 (0.067%)<br>• Bacillus fortis [taxid 254758]: 2 (0.033%)<br>• Bacillus swezeyi [taxid 1925020]: 2 (0.033%)<br>• Bacillus spizizenii [taxid 96241]: 2 (0.033%)<br>• Bacillus pumilus [taxid 1408]: 2 (0.033%)<br>• Bacillus cereus [taxid 1396]: 2 (0.033%)<br>• Frankia canadensis [taxid 1836972]: 1 (0.016%)<br>• other: 31 (0.525%)
Benchmark OTU ID: `CP003056-_Firmicutes`<br>OTU taxon: Bacillus coagulans 36D1 [taxid 345219]<br>Expected: Bacillus coagulans [taxid 1398] (species)<br>Number of reads: 4915<br>Number of identified reads: 4894 (99.572%)	• **species: 3089 (62.848%)**<br>• genus: 287 (5.839%)<br>• family: 178 (3.621%)<br>• order: 196 (3.987%)<br>• class: 93 (1.892%)<br>• phylum: 88 (1.79%)<br>• superkingdom: 271 (5.513%)<br>• root: 687 (13.977%)	• Bacillus thermoamylovorans [taxid 35841]: 2 (0.04%)<br>• Bacillus fortis [taxid 254758]: 1 (0.02%)<br>• Bacillus ginsengihumi [taxid 363870]: 1 (0.02%)<br>• Bacillus sp. B-jedd [taxid 1476857]: 1 (0.02%)<br>• Macrococcus carouselicus [taxid 69969]: 1 (0.02%)<br>• Jeotgalibacillus campisalis [taxid 220754]: 1 (0.02%)<br>• Caldalkalibacillus thermarum [taxid 296745]: 1 (0.02%)<br>• Tetragenococcus koreensis [taxid 290335]: 1 (0.02%)<br>• other: 13 (0.264%)
Benchmark OTU ID: `BA000004-_Firmicutes`<br>OTU taxon: Bacillus halodurans C-125 [taxid 272558]<br>Expected: Alkalihalobacillus halodurans [taxid 86665] (species)<br>Number of reads: 5955<br>Number of identified reads: 5918 (99.378%)	• species: 1094 (18.371%)<br>• **genus: 2896 (48.631%)**<br>• family: 456 (7.657%)<br>• order: 239 (4.013%)<br>• class: 59 (0.99%)<br>• phylum: 67 (1.125%)<br>• superkingdom: 333 (5.591%)<br>• root: 771 (12.947%)	• Alkalihalobacillus okuhidensis [taxid 136160]: 15 (0.251%)<br>• Alkalihalobacillus hemicellulosilyticus [taxid 127886]: 4 (0.067%)<br>• Alkalihalobacillus pseudofirmus [taxid 79885]: 3 (0.05%)<br>• Bacillus stratosphericus [taxid 293386]: 2 (0.033%)<br>• Alkalihalobacillus wakoensis [taxid 127891]: 2 (0.033%)<br>• Staphylococcus aureus [taxid 1280]: 2 (0.033%)<br>• Hydrogenovibrio marinus [taxid 28885]: 1 (0.016%)<br>• Anoxybacillus ayderensis [taxid 265546]: 1 (0.016%)<br>• other: 22 (0.369%)
Benchmark OTU ID: `CP000002-_Firmicutes`<br>OTU taxon: Bacillus licheniformis DSM 13 = ATCC 14580 [taxid 279010]<br>Expected: Bacillus licheniformis [taxid 1402] (species)<br>Number of reads: 5987<br>Number of identified reads: 5968 (99.682%)	• species: 1330 (22.214%)<br>• **genus: 3158 (52.747%)**<br>• family: 206 (3.44%)<br>• order: 183 (3.056%)<br>• class: 38 (0.634%)<br>• phylum: 47 (0.785%)<br>• superkingdom: 290 (4.843%)<br>• root: 707 (11.808%)	• Bacillus paralicheniformis [taxid 1648923]: 23 (0.384%)<br>• Bacillus swezeyi [taxid 1925020]: 8 (0.133%)<br>• Bacillus haynesii [taxid 1925021]: 7 (0.116%)<br>• Bacillus glycinifermentans [taxid 1664069]: 5 (0.083%)<br>• Bacillus gobiensis [taxid 1441095]: 4 (0.066%)<br>• Bacillus subtilis [taxid 1423]: 3 (0.05%)<br>• Bacillus obstructivus [taxid 1914540]: 2 (0.033%)<br>• Bacillus atrophaeus [taxid 1452]: 2 (0.033%)<br>• other: 42 (0.701%)

Operational Taxonomic Unit (OTU)	Correct identifications	Wrong or overspecific identifications at species rank
Benchmark OTU ID: `CP001982-_Firmicutes`<br>OTU taxon: Bacillus megaterium DSM 319 [taxid 592022]<br>Expected: Priestia megaterium [taxid 1404] (species)<br>Number of reads: 7387<br>Number of identified reads: 7279 (98.537%)	• species: 979 (13.253%)<br>• genus: 0 (0.0%)<br>• **family: 4922 (66.63%)**<br>• order: 214 (2.896%)<br>• class: 43 (0.582%)<br>• phylum: 74 (1.001%)<br>• superkingdom: 320 (4.331%)<br>• root: 724 (9.801%)	• Bacillus aryabhattai [taxid 412384]: 21 (0.284%)<br>• Bacillus sp. ALD [taxid 2293318]: 3 (0.04%)<br>• Paraliobacillus ryukyuensis [taxid 200904]: 2 (0.027%)<br>• Mesobacillus persicus [taxid 930146]: 2 (0.027%)<br>• Staphylococcus aureus [taxid 1280]: 2 (0.027%)<br>• Bacillus aquiflavi [taxid 2672567]: 2 (0.027%)<br>• Bacillus thuringiensis [taxid 1428]: 2 (0.027%)<br>• Bacillus oleivorans [taxid 1448271]: 2 (0.027%)<br>• other: 49 (0.663%)
Benchmark OTU ID: `CP003017-_Firmicutes`<br>OTU taxon: Bacillus megaterium WSH-002 [taxid 1006007]<br>Expected: Priestia megaterium [taxid 1404] (species)<br>Number of reads: 7203<br>Number of identified reads: 7110 (98.708%)	• species: 751 (10.426%)<br>• genus: 0 (0.0%)<br>• **family: 4923 (68.346%)**<br>• order: 228 (3.165%)<br>• class: 46 (0.638%)<br>• phylum: 59 (0.819%)<br>• superkingdom: 303 (4.206%)<br>• root: 793 (11.009%)	• Bacillus aryabhattai [taxid 412384]: 68 (0.944%)<br>• Bacillus sp. ALD [taxid 2293318]: 6 (0.083%)<br>• Bacillus koreensis [taxid 284581]: 4 (0.055%)<br>• Staphylococcus aureus [taxid 1280]: 3 (0.041%)<br>• Bacillus wiedmannii [taxid 1890302]: 2 (0.027%)<br>• Schizaphis graminum [taxid 13262]: 2 (0.027%)<br>• Oceanobacillus sojae [taxid 582851]: 2 (0.027%)<br>• Bacillus dafuensis [taxid 1742359]: 2 (0.027%)<br>• other: 62 (0.86%)
Benchmark OTU ID: `CP000813-_Firmicutes`<br>OTU taxon: Bacillus pumilus SAFR-032 [taxid 315750]<br>Expected: Bacillus pumilus [taxid 1408] (species)<br>Number of reads: 5158<br>Number of identified reads: 5133 (99.515%)	• species: 1349 (26.153%)<br>• **genus: 2733 (52.985%)**<br>• family: 170 (3.295%)<br>• order: 140 (2.714%)<br>• class: 34 (0.659%)<br>• phylum: 49 (0.949%)<br>• superkingdom: 232 (4.497%)<br>• root: 422 (8.181%)	• Bacillus stratosphericus [taxid 293386]: 37 (0.717%)<br>• Bacillus safensis [taxid 561879]: 16 (0.31%)<br>• Bacillus australimaris [taxid 1326968]: 6 (0.116%)<br>• Bacillus xiamenensis [taxid 1178537]: 4 (0.077%)<br>• Bacillus zhangzhouensis [taxid 1178540]: 3 (0.058%)<br>• Bacillus sp. SJS [taxid 1423321]: 2 (0.038%)<br>• Bacillus subtilis [taxid 1423]: 2 (0.038%)<br>• Alteribacillus iranensis [taxid 930128]: 1 (0.019%)<br>• other: 20 (0.387%)
Benchmark OTU ID: `CP005586-_Firmicutes`<br>OTU taxon: Bacillus sp. 1NLA3E [taxid 666686]<br>Expected: Bacillus [taxid 1386] (genus)<br>Number of reads: 6936<br>Number of identified reads: 6821 (98.341%)	• **genus: 4584 (66.089%)**<br>• family: 472 (6.805%)<br>• order: 301 (4.339%)<br>• class: 65 (0.937%)<br>• phylum: 101 (1.456%)<br>• superkingdom: 356 (5.132%)<br>• root: 929 (13.393%)	• Bacillus sp. B-jedd [taxid 1476857]: 10 (0.144%)<br>• Bacillus sp. (in: Bacteria) [taxid 1409]: 4 (0.057%)<br>• Bacillus sp. SJS [taxid 1423321]: 4 (0.057%)<br>• Bacillus methanolicus [taxid 1471]: 4 (0.057%)<br>• Anaerobacillus alkalilacustris [taxid 393763]: 3 (0.043%)<br>• Beta vulgaris [taxid 161934]: 3 (0.043%)<br>• Listeria monocytogenes [taxid 1639]: 2 (0.028%)<br>• other: 69 (0.994%)

Operational Taxonomic Unit (OTU)	Correct identifications	Wrong or overspecific identifications at species rank
Benchmark OTU ID: `CP003492-_Firmicutes`<br>OTU taxon: Bacillus sp. JS [taxid 1127744]<br>Expected: Bacillus sp. JS [taxid 1127744] (species)<br>Number of reads: 5824<br>Number of identified reads: 5812 (99.793%)	• species: 1154 (19.814%)<br>• **genus: 3400 (58.379%)**<br>• family: 157 (2.695%)<br>• order: 150 (2.575%)<br>• class: 34 (0.583%)<br>• phylum: 55 (0.944%)<br>• superkingdom: 271 (4.653%)<br>• root: 586 (10.061%)	• Bacillus subtilis [taxid 1423]: 73 (1.253%)<br>• Bacillus atrophaeus [taxid 1452]: 8 (0.137%)<br>• Bacillus intestinalis [taxid 1963032]: 7 (0.12%)<br>• Bacillus spizizenii [taxid 96241]: 4 (0.068%)<br>• Bacillus nakamurai [taxid 1793963]: 4 (0.068%)<br>• Bacillus vallismortis [taxid 72361]: 3 (0.051%)<br>• Bacillus velezensis [taxid 492670]: 3 (0.051%)<br>• Bacillus mojavensis [taxid 72360]: 3 (0.051%)<br>• other: 36 (0.618%)
Benchmark OTU ID: `AP012495-_Cyanobacteria`<br>OTU taxon: Bacillus subtilis BEST7613 [taxid 1204343]<br>Expected: Bacillus subtilis [taxid 1423] (species)<br>Number of reads: 46204<br>Number of identified reads: 45923 (99.391%)	• species: 2168 (4.692%)<br>• genus: 17138 (37.092%)<br>• family: 683 (1.478%)<br>• order: 635 (1.374%)<br>• class: 181 (0.391%)<br>• phylum: 208 (0.45%)<br>• **superkingdom: 19991 (43.266%)**<br>• root: 4857 (10.512%)	• Bacillus sp. JS [taxid 1127744]: 43 (0.093%)<br>• Bacillus spizizenii [taxid 96241]: 42 (0.09%)<br>• Bacillus atrophaeus [taxid 1452]: 34 (0.073%)<br>• Bacillus tequilensis [taxid 227866]: 33 (0.071%)<br>• Bacillus vallismortis [taxid 72361]: 28 (0.06%)<br>• Bacillus nakamurai [taxid 1793963]: 19 (0.041%)<br>• Bacillus mojavensis [taxid 72360]: 17 (0.036%)<br>• Bacillus amyloliquefaciens [taxid 1390]: 17 (0.036%)<br>• other: 308 (0.666%)
Benchmark OTU ID: `CP003329-_Firmicutes`<br>OTU taxon: Bacillus subtilis subsp. subtilis 6051-HGW [taxid 1147161]<br>Expected: Bacillus subtilis [taxid 1423] (species)<br>Number of reads: 5976<br>Number of identified reads: 5948 (99.531%)	• species: 536 (8.969%)<br>• **genus: 4119 (68.925%)**<br>• family: 172 (2.878%)<br>• order: 147 (2.459%)<br>• class: 57 (0.953%)<br>• phylum: 45 (0.753%)<br>• superkingdom: 236 (3.949%)<br>• root: 629 (10.525%)	• Bacillus sp. JS [taxid 1127744]: 16 (0.267%)<br>• Bacillus tequilensis [taxid 227866]: 11 (0.184%)<br>• Bacillus atrophaeus [taxid 1452]: 8 (0.133%)<br>• Bacillus spizizenii [taxid 96241]: 8 (0.133%)<br>• Bacillus vallismortis [taxid 72361]: 7 (0.117%)<br>• Bacillus amyloliquefaciens [taxid 1390]: 4 (0.066%)<br>• Bacillus gobiensis [taxid 1441095]: 3 (0.05%)<br>• Bacillus velezensis [taxid 492670]: 2 (0.033%)<br>• other: 39 (0.652%)
Benchmark OTU ID: `CP004405-_Firmicutes`<br>OTU taxon: Bacillus subtilis subsp. subtilis str. BAB-1 [taxid 1302650]<br>Expected: Bacillus subtilis [taxid 1423] (species)<br>Number of reads: 5666<br>Number of identified reads: 5640 (99.541%)	• species: 687 (12.124%)<br>• **genus: 3843 (67.825%)**<br>• family: 148 (2.612%)<br>• order: 139 (2.453%)<br>• class: 34 (0.6%)<br>• phylum: 45 (0.794%)<br>• superkingdom: 216 (3.812%)<br>• root: 524 (9.248%)	• Bacillus sp. JS [taxid 1127744]: 12 (0.211%)<br>• Bacillus spizizenii [taxid 96241]: 11 (0.194%)<br>• Bacillus atrophaeus [taxid 1452]: 7 (0.123%)<br>• Bacillus tequilensis [taxid 227866]: 6 (0.105%)<br>• Bacillus gobiensis [taxid 1441095]: 4 (0.07%)<br>• Bacillus vallismortis [taxid 72361]: 4 (0.07%)<br>• Bacillus stratosphericus [taxid 293386]: 4 (0.07%)<br>• Bacillus glycinifermentans [taxid 1664069]: 3 (0.052%)<br>• other: 46 (0.811%)

Operational Taxonomic Unit (OTU)	Correct identifications	Wrong or overspecific identifications at species rank
Benchmark OTU ID: `ENA|CM000748|CM000748.1-_Firmicutes`<br>OTU taxon: Bacillus thuringiensis serovar thuringiensis str. T01001 [taxid 527025]<br>Expected: Bacillus thuringiensis [taxid 1428] (species)<br>Number of reads: 9175<br>Number of identified reads: 9020 (98.31%)	• species: 310 (3.378%)<br>• **genus: 7399 (80.643%)**<br>• family: 93 (1.013%)<br>• order: 114 (1.242%)<br>• class: 40 (0.435%)<br>• phylum: 58 (0.632%)<br>• superkingdom: 276 (3.008%)<br>• root: 726 (7.912%)	• **Bacillus cereus [taxid 1396]: 220 (2.397%)**<br>• Bacillus wiedmannii [taxid 1890302]: 27 (0.294%)<br>• Bacillus mycoides [taxid 1405]: 22 (0.239%)<br>• Bacillus anthracis [taxid 1392]: 11 (0.119%)<br>• Bacillus toyonensis [taxid 155322]: 10 (0.108%)<br>• Bacillus cytotoxicus [taxid 580165]: 7 (0.076%)<br>• Bacillus pseudomycoides [taxid 64104]: 7 (0.076%)<br>• Bacillus pacificus [taxid 2026187]: 4 (0.043%)<br>• other: 43 (0.468%)
Benchmark OTU ID: `CP003838-_Firmicutes`<br>OTU taxon: Bacillus velezensis AS43.3 [taxid 1225788]<br>Expected: Bacillus velezensis [taxid 492670] (species)<br>Number of reads: 5569<br>Number of identified reads: 5547 (99.604%)	• species: 157 (2.819%)<br>• **genus: 4190 (75.237%)**<br>• family: 145 (2.603%)<br>• order: 139 (2.495%)<br>• class: 52 (0.933%)<br>• phylum: 39 (0.7%)<br>• superkingdom: 214 (3.842%)<br>• root: 600 (10.773%)	• Bacillus amyloliquefaciens [taxid 1390]: 73 (1.31%)<br>• Bacillus nakamurai [taxid 1793963]: 9 (0.161%)<br>• Bacillus subtilis [taxid 1423]: 8 (0.143%)<br>• Bacillus siamensis [taxid 659243]: 7 (0.125%)<br>• Bacillus sp. RUPDJ [taxid 1907305]: 5 (0.089%)<br>• Bacillus stratosphericus [taxid 293386]: 3 (0.053%)<br>• Bacillus atrophaeus [taxid 1452]: 3 (0.053%)<br>• Bacillus vallismortis [taxid 72361]: 3 (0.053%)<br>• other: 40 (0.718%)
Benchmark OTU ID: `HE617159-_Firmicutes`<br>OTU taxon: Bacillus velezensis CAU B946 [taxid 1114958]<br>Expected: Bacillus velezensis [taxid 492670] (species)<br>Number of reads: 5663<br>Number of identified reads: 5626 (99.346%)	• species: 96 (1.695%)<br>• **genus: 4325 (76.372%)**<br>• family: 161 (2.843%)<br>• order: 135 (2.383%)<br>• class: 42 (0.741%)<br>• phylum: 47 (0.829%)<br>• superkingdom: 192 (3.39%)<br>• root: 619 (10.93%)	• Bacillus amyloliquefaciens [taxid 1390]: 65 (1.147%)<br>• Bacillus intestinalis [taxid 1963032]: 13 (0.229%)<br>• Bacillus nakamurai [taxid 1793963]: 10 (0.176%)<br>• Bacillus subtilis [taxid 1423]: 9 (0.158%)<br>• Bacillus atrophaeus [taxid 1452]: 8 (0.141%)<br>• Bacillus siamensis [taxid 659243]: 6 (0.105%)<br>• Lupinus albus [taxid 3870]: 3 (0.052%)<br>• Bacillus swezeyi [taxid 1925020]: 3 (0.052%)<br>• other: 41 (0.723%)
Benchmark OTU ID: `FQ312004-_Bacteroidetes`<br>OTU taxon: Bacteroides fragilis 638R [taxid 862962]<br>Expected: Bacteroides fragilis [taxid 817] (species)<br>Number of reads: 37025<br>Number of identified reads: 36811 (99.422%)	• **species: 18468 (49.879%)**<br>• genus: 9466 (25.566%)<br>• family: 15 (0.04%)<br>• order: 3588 (9.69%)<br>• class: 49 (0.132%)<br>• phylum: 528 (1.426%)<br>• superkingdom: 1226 (3.311%)<br>• root: 3457 (9.336%)	• Bacteroides ovatus [taxid 28116]: 19 (0.051%)<br>• Bacteroides thetaiotaomicron [taxid 818]: 16 (0.043%)<br>• Phocaeicola dorei [taxid 357276]: 14 (0.037%)<br>• Bacteroides stercoris [taxid 46506]: 12 (0.032%)<br>• Bacteroides uniformis [taxid 820]: 10 (0.027%)<br>• Parabacteroides distasonis [taxid 823]: 8 (0.021%)<br>• Bacteroides reticulotermitis [taxid 1133319]: 8 (0.021%)<br>• Bacteroides pyogenes [taxid 310300]: 8 (0.021%)<br>• other: 151 (0.407%)

Operational Taxonomic Unit (OTU)	Correct identifications	Wrong or overspecific identifications at species rank
Benchmark OTU ID: `CR626927-_Bacteroidetes`<br>OTU taxon: Bacteroides fragilis NCTC 9343 [taxid 272559]<br>Expected: Bacteroides fragilis [taxid 817] (species)<br>Number of reads: 35748<br>Number of identified reads: 35504 (99.317%)	• **species: 18155 (50.786%)**<br>• genus: 9298 (26.009%)<br>• family: 11 (0.03%)<br>• order: 2920 (8.168%)<br>• class: 41 (0.114%)<br>• phylum: 494 (1.381%)<br>• superkingdom: 1243 (3.477%)<br>• root: 3322 (9.292%)	• Bacteroides pyogenes [taxid 310300]: 11 (0.03%)<br>• Bacteroides caccae [taxid 47678]: 11 (0.03%)<br>• Bacteroides intestinalis [taxid 329854]: 11 (0.03%)<br>• Bacteroides uniformis [taxid 820]: 11 (0.03%)<br>• Phocaeicola dorei [taxid 357276]: 11 (0.03%)<br>• Bacteroides reticulotermitis [taxid 1133319]: 8 (0.022%)<br>• Bacteroides thetaiotaomicron [taxid 818]: 8 (0.022%)<br>• Bacteroides clarus [taxid 626929]: 7 (0.019%)<br>• other: 129 (0.36%)
Benchmark OTU ID: `AP006841-_Bacteroidetes`<br>OTU taxon: Bacteroides fragilis YCH46 [taxid 295405]<br>Expected: Bacteroides fragilis [taxid 817] (species)<br>Number of reads: 36268<br>Number of identified reads: 36045 (99.385%)	• **species: 17959 (49.517%)**<br>• genus: 9351 (25.783%)<br>• family: 18 (0.049%)<br>• order: 3683 (10.154%)<br>• class: 41 (0.113%)<br>• phylum: 469 (1.293%)<br>• superkingdom: 1227 (3.383%)<br>• root: 3287 (9.063%)	• Bacteroides caccae [taxid 47678]: 21 (0.057%)<br>• Bacteroides xylanisolvens [taxid 371601]: 17 (0.046%)<br>• Bacteroides pyogenes [taxid 310300]: 14 (0.038%)<br>• Bacteroides stercoris [taxid 46506]: 13 (0.035%)<br>• Bacteroides intestinalis [taxid 329854]: 12 (0.033%)<br>• Phocaeicola vulgatus [taxid 821]: 11 (0.03%)<br>• Bacteroides salyersiae [taxid 291644]: 11 (0.03%)<br>• Bacteroides cellulosilyticus [taxid 246787]: 10 (0.027%)<br>• other: 180 (0.496%)
Benchmark OTU ID: `CP002352-_Bacteroidetes`<br>OTU taxon: Bacteroides helcogenes P 36-108 [taxid 693979]<br>Expected: Bacteroides helcogenes [taxid 290053] (species)<br>Number of reads: 26581<br>Number of identified reads: 26479 (99.616%)	• **species: 15420 (58.011%)**<br>• genus: 4511 (16.97%)<br>• family: 42 (0.158%)<br>• order: 2140 (8.05%)<br>• class: 45 (0.169%)<br>• phylum: 406 (1.527%)<br>• superkingdom: 967 (3.637%)<br>• root: 2932 (11.03%)	• Bacteroides fragilis [taxid 817]: 26 (0.097%)<br>• Bacteroides stercoris [taxid 46506]: 25 (0.094%)<br>• Bacteroides fluxus [taxid 626930]: 12 (0.045%)<br>• Bacteroides heparinolyticus [taxid 28113]: 12 (0.045%)<br>• Bacteroides uniformis [taxid 820]: 11 (0.041%)<br>• Bacteroides cellulosilyticus [taxid 246787]: 11 (0.041%)<br>• Phocaeicola vulgatus [taxid 821]: 7 (0.026%)<br>• Bacteroides pyogenes [taxid 310300]: 7 (0.026%)<br>• other: 109 (0.41%)
Benchmark OTU ID: `CP003123-_Proteobacteria`<br>OTU taxon: Bartonella australis Aust/NH1 [taxid 1094489]<br>Expected: Bartonella australis [taxid 388640] (species)<br>Number of reads: 2708<br>Number of identified reads: 2684 (99.113%)	• **species: 1600 (59.084%)**<br>• genus: 274 (10.118%)<br>• family: 0 (0.0%)<br>• order: 129 (4.763%)<br>• class: 64 (2.363%)<br>• phylum: 56 (2.067%)<br>• superkingdom: 85 (3.138%)<br>• root: 474 (17.503%)	• Bartonella quintana [taxid 803]: 2 (0.073%)<br>• Bartonella koehlerae [taxid 92181]: 2 (0.073%)<br>• Klebsiella pneumoniae [taxid 573]: 1 (0.036%)<br>• Peptoniphilus coxii [taxid 755172]: 1 (0.036%)<br>• Brucella canis [taxid 36855]: 1 (0.036%)<br>• Bartonella rattimassiliensis [taxid 270250]: 1 (0.036%)<br>• Bartonella krasnovii [taxid 2267275]: 1 (0.036%)<br>• Sporolactobacillus laevolacticus [taxid 33018]: 1 (0.036%)<br>• other: 12 (0.443%)

Operational Taxonomic Unit (OTU)	Correct identifications	Wrong or overspecific identifications at species rank
Benchmark OTU ID: `CP000524-_Proteobacteria`<br>OTU taxon: Bartonella bacilliformis KC583 [taxid 360095]<br>Expected: Bartonella bacilliformis [taxid 774] (species)<br>Number of reads: 2367<br>Number of identified reads: 2341 (98.901%)	• **species: 1456 (61.512%)**<br>• genus: 236 (9.97%)<br>• family: 0 (0.0%)<br>• order: 105 (4.435%)<br>• class: 67 (2.83%)<br>• phylum: 45 (1.901%)<br>• superkingdom: 89 (3.76%)<br>• root: 341 (14.406%)	• Bartonella ancashensis [taxid 1318743]: 2 (0.084%)<br>• Sediminispirochaeta smaragdinae [taxid 55206]: 1 (0.042%)<br>• Bartonella rochalimae [taxid 395923]: 1 (0.042%)<br>• Bartonella schoenbuchensis [taxid 165694]: 1 (0.042%)<br>• Variibacter gotjawalensis [taxid 1333996]: 1 (0.042%)<br>• Bartonella birtlesii [taxid 111504]: 1 (0.042%)<br>• Rhizobium etli [taxid 29449]: 1 (0.042%)<br>• Bartonella alsatica [taxid 52764]: 1 (0.042%)<br>• other: 4 (0.168%)
Benchmark OTU ID: `FN645454-_Proteobacteria`<br>OTU taxon: Bartonella clarridgeiae 73 [taxid 696125]<br>Expected: Bartonella clarridgeiae [taxid 56426] (species)<br>Number of reads: 2542<br>Number of identified reads: 2483 (97.678%)	• **species: 1165 (45.83%)**<br>• genus: 704 (27.694%)<br>• family: 0 (0.0%)<br>• order: 84 (3.304%)<br>• class: 58 (2.281%)<br>• phylum: 47 (1.848%)<br>• superkingdom: 74 (2.911%)<br>• root: 350 (13.768%)	• Bartonella birtlesii [taxid 111504]: 3 (0.118%)<br>• Bartonella rochalimae [taxid 395923]: 3 (0.118%)<br>• Lupinus albus [taxid 3870]: 1 (0.039%)<br>• Bartonella vinsonii [taxid 33047]: 1 (0.039%)<br>• Bartonella schoenbuchensis [taxid 165694]: 1 (0.039%)<br>• Bartonella doshiae [taxid 33044]: 1 (0.039%)<br>• Yersinia pestis [taxid 632]: 1 (0.039%)<br>• Bartonella apis [taxid 1686310]: 1 (0.039%)<br>• other: 9 (0.354%)
Benchmark OTU ID: `BX897699-_Proteobacteria`<br>OTU taxon: Bartonella henselae str. Houston-1 [taxid 283166]<br>Expected: Bartonella henselae [taxid 38323] (species)<br>Number of reads: 3461<br>Number of identified reads: 3388 (97.89%)	• **species: 1282 (37.041%)**<br>• genus: 1063 (30.713%)<br>• family: 0 (0.0%)<br>• order: 127 (3.669%)<br>• class: 77 (2.224%)<br>• phylum: 63 (1.82%)<br>• superkingdom: 83 (2.398%)<br>• root: 689 (19.907%)	• Bartonella koehlerae [taxid 92181]: 5 (0.144%)<br>• Bartonella vinsonii [taxid 33047]: 5 (0.144%)<br>• Bartonella ancashensis [taxid 1318743]: 4 (0.115%)<br>• Bartonella tribocorum [taxid 85701]: 4 (0.115%)<br>• Bartonella doshiae [taxid 33044]: 3 (0.086%)<br>• Bartonella quintana [taxid 803]: 3 (0.086%)<br>• Bartonella taylorii [taxid 33046]: 3 (0.086%)<br>• Bartonella kosoyi [taxid 2133959]: 2 (0.057%)<br>• other: 18 (0.52%)
Benchmark OTU ID: `CP003784-_Proteobacteria`<br>OTU taxon: Bartonella quintana RM-11 [taxid 1225179]<br>Expected: Bartonella quintana [taxid 803] (species)<br>Number of reads: 2688<br>Number of identified reads: 2659 (98.921%)	• **species: 1210 (45.014%)**<br>• genus: 622 (23.139%)<br>• family: 0 (0.0%)<br>• order: 89 (3.311%)<br>• class: 61 (2.269%)<br>• phylum: 59 (2.194%)<br>• superkingdom: 70 (2.604%)<br>• root: 544 (20.238%)	• Bartonella vinsonii [taxid 33047]: 5 (0.186%)<br>• Bartonella washoeensis [taxid 186739]: 4 (0.148%)<br>• Bartonella rattimassiliensis [taxid 270250]: 3 (0.111%)<br>• Bartonella henselae [taxid 38323]: 2 (0.074%)<br>• Bartonella doshiae [taxid 33044]: 2 (0.074%)<br>• Bartonella krasnovii [taxid 2267275]: 1 (0.037%)<br>• Pararhodospirillum photometricum [taxid 1084]: 1 (0.037%)<br>• Bartonella tamiae [taxid 373638]: 1 (0.037%)<br>• other: 13 (0.483%)

Operational Taxonomic Unit (OTU)	Correct identifications	Wrong or overspecific identifications at species rank
Benchmark OTU ID: `BX897700-_Proteobacteria`<br>OTU taxon: Bartonella quintana str. Toulouse [taxid 283165]<br>Expected: Bartonella quintana [taxid 803] (species)<br>Number of reads: 2674<br>Number of identified reads: 2633 (98.466%)	• **species: 1261 (47.157%)**<br>• genus: 603 (22.55%)<br>• family: 0 (0.0%)<br>• order: 100 (3.739%)<br>• class: 60 (2.243%)<br>• phylum: 48 (1.795%)<br>• superkingdom: 73 (2.729%)<br>• root: 485 (18.137%)	• Bartonella bacilliformis [taxid 774]: 3 (0.112%)<br>• Bartonella vinsonii [taxid 33047]: 2 (0.074%)<br>• Bartonella australis [taxid 388640]: 2 (0.074%)<br>• Bartonella grahamii [taxid 33045]: 2 (0.074%)<br>• Bartonella washoeensis [taxid 186739]: 2 (0.074%)<br>• Bartonella rochalimae [taxid 395923]: 1 (0.037%)<br>• Parastrongyloides trichosuri [taxid 131310]: 1 (0.037%)<br>• Bartonella ancashensis [taxid 1318743]: 1 (0.037%)<br>• other: 16 (0.598%)
Benchmark OTU ID: `CP003124-_Proteobacteria`<br>OTU taxon: Bartonella vinsonii subsp. berkhoffii str. Winnie [taxid 1094497]<br>Expected: Bartonella vinsonii [taxid 33047] (species)<br>Number of reads: 3172<br>Number of identified reads: 3123 (98.455%)	• **species: 1287 (40.573%)**<br>• genus: 968 (30.517%)<br>• family: 0 (0.0%)<br>• order: 131 (4.129%)<br>• class: 70 (2.206%)<br>• phylum: 55 (1.733%)<br>• superkingdom: 113 (3.562%)<br>• root: 493 (15.542%)	• Bartonella krasnovii [taxid 2267275]: 11 (0.346%)<br>• Bartonella tribocorum [taxid 85701]: 9 (0.283%)<br>• Bartonella washoeensis [taxid 186739]: 9 (0.283%)<br>• Bartonella grahamii [taxid 33045]: 8 (0.252%)<br>• Bartonella quintana [taxid 803]: 7 (0.22%)<br>• Lupinus albus [taxid 3870]: 5 (0.157%)<br>• Bartonella taylorii [taxid 33046]: 5 (0.157%)<br>• Bartonella henselae [taxid 38323]: 5 (0.157%)<br>• other: 33 (1.04%)
Benchmark OTU ID: `CP003537-_Proteobacteria`<br>OTU taxon: Bdellovibrio exovorus JSS [taxid 1184267]<br>Expected: Bdellovibrio exovorus [taxid 453816] (species)<br>Number of reads: 5096<br>Number of identified reads: 5066 (99.411%)	• **species: 4144 (81.318%)**<br>• genus: 56 (1.098%)<br>• family: 1 (0.019%)<br>• order: 42 (0.824%)<br>• class: 6 (0.117%)<br>• phylum: 152 (2.982%)<br>• superkingdom: 205 (4.022%)<br>• root: 457 (8.967%)	• Bdellovibrio bacteriovorus [taxid 959]: 1 (0.019%)<br>• Ruminobacter amylophilus [taxid 867]: 1 (0.019%)<br>• Amnibacterium flavum [taxid 2173173]: 1 (0.019%)<br>• Rhizopus stolonifer [taxid 4846]: 1 (0.019%)<br>• Sphaeroforma arctica [taxid 72019]: 1 (0.019%)<br>• Bacteriovorax stolpii [taxid 960]: 1 (0.019%)
Benchmark OTU ID: `CP001016-_Proteobacteria`<br>OTU taxon: Beijerinckia indica subsp. indica ATCC 9039 [taxid 395963]<br>Expected: Beijerinckia indica [taxid 533] (species)<br>Number of reads: 8499<br>Number of identified reads: 8477 (99.741%)	• **species: 5235 (61.595%)**<br>• genus: 0 (0.0%)<br>• family: 90 (1.058%)<br>• order: 457 (5.377%)<br>• class: 367 (4.318%)<br>• phylum: 358 (4.212%)<br>• superkingdom: 387 (4.553%)<br>• root: 1571 (18.484%)	• Beta vulgaris [taxid 161934]: 2 (0.023%)<br>• Rhodomicrobium vannielii [taxid 1069]: 2 (0.023%)<br>• Massilia plicata [taxid 321984]: 1 (0.011%)<br>• Hypericibacter terrae [taxid 2602015]: 1 (0.011%)<br>• Novosphingobium malaysiense [taxid 1348853]: 1 (0.011%)<br>• Hyphomicrobium facile [taxid 51670]: 1 (0.011%)<br>• Methyloceanibacter marginalis [taxid 1774971]: 1 (0.011%)<br>• Nocardiopsis dassonvillei [taxid 2014]: 1 (0.011%)<br>• other: 35 (0.411%)

Operational Taxonomic Unit (OTU)	Correct identifications	Wrong or overspecific identifications at species rank
Benchmark OTU ID: `CP001618-_Actinobacteria`<br>OTU taxon: Beutenbergia cavernae DSM 12333 [taxid 471853]<br>Expected: Beutenbergia cavernae [taxid 84757] (species)<br>Number of reads: 20476<br>Number of identified reads: 20415 (99.702%)	• **species: 14074 (68.734%)**<br>• genus: 0 (0.0%)<br>• family: 42 (0.205%)<br>• order: 1028 (5.02%)<br>• class: 1942 (9.484%)<br>• phylum: 23 (0.112%)<br>• superkingdom: 1562 (7.628%)<br>• root: 1731 (8.453%)	• Georgenia subflava [taxid 1622177]: 3 (0.014%)<br>• Humibacillus xanthopallidus [taxid 412689]: 2 (0.009%)<br>• Luteimicrobium subarcticum [taxid 620910]: 2 (0.009%)<br>• Nocardioides immobilis [taxid 2049295]: 2 (0.009%)<br>• Micrococcus luteus [taxid 1270]: 2 (0.009%)<br>• Phytohabitans suffuscus [taxid 624315]: 1 (0.004%)<br>• Pseudooceanicola algae [taxid 1537215]: 1 (0.004%)<br>• Microcella alkaliphila [taxid 279828]: 1 (0.004%)<br>• other: 88 (0.429%)
Benchmark OTU ID: `CP003745-_Proteobacteria`<br>OTU taxon: Bibersteinia trehalosi USDA-ARS-USMARC-192 [taxid 1171377]<br>Expected: Bibersteinia trehalosi [taxid 47735] (species)<br>Number of reads: 4534<br>Number of identified reads: 4513 (99.536%)	• **species: 2744 (60.52%)**<br>• genus: 0 (0.0%)<br>• family: 755 (16.651%)<br>• order: 0 (0.0%)<br>• class: 394 (8.689%)<br>• phylum: 104 (2.293%)<br>• superkingdom: 164 (3.617%)<br>• root: 350 (7.719%)	• Haemophilus influenzae [taxid 727]: 4 (0.088%)<br>• Salmonella enterica [taxid 28901]: 3 (0.066%)<br>• Mannheimia haemolytica [taxid 75985]: 3 (0.066%)<br>• Mannheimia granulomatis [taxid 85402]: 3 (0.066%)<br>• [Haemophilus] ducreyi [taxid 730]: 2 (0.044%)<br>• Klebsiella pneumoniae [taxid 573]: 2 (0.044%)<br>• Pararhodospirillum photometricum [taxid 1084]: 2 (0.044%)<br>• Escherichia coli [taxid 562]: 2 (0.044%)<br>• other: 31 (0.683%)
Benchmark OTU ID: `AP009256-_Actinobacteria`<br>OTU taxon: Bifidobacterium adolescentis ATCC 15703 [taxid 367928]<br>Expected: Bifidobacterium adolescentis [taxid 1680] (species)<br>Number of reads: 7835<br>Number of identified reads: 7818 (99.783%)	• species: 1704 (21.748%)<br>• **genus: 4636 (59.17%)**<br>• family: 200 (2.552%)<br>• order: 18 (0.229%)<br>• class: 168 (2.144%)<br>• phylum: 21 (0.268%)<br>• superkingdom: 396 (5.054%)<br>• root: 669 (8.538%)	• Bifidobacterium pseudocatenulatum [taxid 28026]: 13 (0.165%)<br>• Bifidobacterium ruminantium [taxid 78346]: 10 (0.127%)<br>• Bifidobacterium longum [taxid 216816]: 10 (0.127%)<br>• Bifidobacterium pullorum [taxid 78448]: 5 (0.063%)<br>• Bifidobacterium dentium [taxid 1689]: 5 (0.063%)<br>• Bifidobacterium bifidum [taxid 1681]: 5 (0.063%)<br>• Bifidobacterium choloepi [taxid 2614131]: 5 (0.063%)<br>• Bifidobacterium breve [taxid 1685]: 4 (0.051%)<br>• other: 82 (1.046%)
Benchmark OTU ID: `CP002567-_Actinobacteria`<br>OTU taxon: Bifidobacterium animalis subsp. animalis ATCC 25527 [taxid 703613]<br>Expected: Bifidobacterium animalis [taxid 28025] (species)<br>Number of reads: 7067<br>Number of identified reads: 7056 (99.844%)	• **species: 4540 (64.242%)**<br>• genus: 1144 (16.187%)<br>• family: 164 (2.32%)<br>• order: 0 (0.0%)<br>• class: 154 (2.179%)<br>• phylum: 7 (0.099%)<br>• superkingdom: 310 (4.386%)<br>• root: 731 (10.343%)	• Bifidobacterium pseudolongum [taxid 1694]: 20 (0.283%)<br>• Bifidobacterium longum [taxid 216816]: 6 (0.084%)<br>• Bifidobacterium magnum [taxid 1692]: 3 (0.042%)<br>• Bifidobacterium cuniculi [taxid 1688]: 2 (0.028%)<br>• Bifidobacterium adolescentis [taxid 1680]: 2 (0.028%)<br>• Beta vulgaris [taxid 161934]: 2 (0.028%)<br>• Bifidobacterium dolichotidis [taxid 2306976]: 2 (0.028%)<br>• Gardnerella vaginalis [taxid 2702]: 1 (0.014%)<br>• other: 25 (0.353%)

Operational Taxonomic Unit (OTU)	Correct identifications	Wrong or overspecific identifications at species rank
Benchmark OTU ID: `CP003497-_Actinobacteria`<br>OTU taxon: Bifidobacterium animalis subsp. lactis B420 [taxid 1168290]<br>Expected: Bifidobacterium animalis [taxid 28025] (species)<br>Number of reads: 7096<br>Number of identified reads: 7072 (99.661%)	• **species: 4449 (62.697%)**<br>• genus: 1194 (16.826%)<br>• family: 184 (2.593%)<br>• order: 0 (0.0%)<br>• class: 154 (2.17%)<br>• phylum: 16 (0.225%)<br>• superkingdom: 344 (4.847%)<br>• root: 727 (10.245%)	• Bifidobacterium pseudolongum [taxid 1694]: 10 (0.14%)<br>• Bifidobacterium adolescentis [taxid 1680]: 6 (0.084%)<br>• Bifidobacterium bifidum [taxid 1681]: 3 (0.042%)<br>• Bifidobacterium tsurumiense [taxid 356829]: 2 (0.028%)<br>• Bifidobacterium longum [taxid 216816]: 2 (0.028%)<br>• Bifidobacterium callitrichos [taxid 762209]: 2 (0.028%)<br>• Bifidobacterium magnum [taxid 1692]: 2 (0.028%)<br>• Bifidobacterium aemilianum [taxid 2493120]: 2 (0.028%)<br>• other: 23 (0.324%)
Benchmark OTU ID: `CP001853-_Actinobacteria`<br>OTU taxon: Bifidobacterium animalis subsp. lactis BB-12 [taxid 552531]<br>Expected: Bifidobacterium animalis [taxid 28025] (species)<br>Number of reads: 7113<br>Number of identified reads: 7090 (99.676%)	• **species: 4530 (63.686%)**<br>• genus: 1174 (16.504%)<br>• family: 155 (2.179%)<br>• order: 1 (0.014%)<br>• class: 177 (2.488%)<br>• phylum: 13 (0.182%)<br>• superkingdom: 313 (4.4%)<br>• root: 720 (10.122%)	• Bifidobacterium choloepi [taxid 2614131]: 5 (0.07%)<br>• Bifidobacterium adolescentis [taxid 1680]: 4 (0.056%)<br>• Bifidobacterium pseudolongum [taxid 1694]: 3 (0.042%)<br>• Bifidobacterium cuniculi [taxid 1688]: 3 (0.042%)<br>• Bifidobacterium bohemicum [taxid 638617]: 2 (0.028%)<br>• Brugia timori [taxid 42155]: 2 (0.028%)<br>• Gardnerella vaginalis [taxid 2702]: 2 (0.028%)<br>• Bifidobacterium pseudocatenulatum [taxid 28026]: 2 (0.028%)<br>• other: 27 (0.379%)
Benchmark OTU ID: `CP003039-_Actinobacteria`<br>OTU taxon: Bifidobacterium animalis subsp. lactis BLC1 [taxid 1075106]<br>Expected: Bifidobacterium animalis [taxid 28025] (species)<br>Number of reads: 7122<br>Number of identified reads: 7101 (99.705%)	• **species: 4535 (63.675%)**<br>• genus: 1183 (16.61%)<br>• family: 153 (2.148%)<br>• order: 1 (0.014%)<br>• class: 178 (2.499%)<br>• phylum: 14 (0.196%)<br>• superkingdom: 335 (4.703%)<br>• root: 696 (9.772%)	• Bifidobacterium pseudolongum [taxid 1694]: 6 (0.084%)<br>• Bifidobacterium bifidum [taxid 1681]: 4 (0.056%)<br>• Bifidobacterium longum [taxid 216816]: 4 (0.056%)<br>• Bifidobacterium choloepi [taxid 2614131]: 3 (0.042%)<br>• Bifidobacterium pullorum [taxid 78448]: 2 (0.028%)<br>• Brugia timori [taxid 42155]: 2 (0.028%)<br>• Dictyobacter aurantiacus [taxid 1936993]: 1 (0.014%)<br>• Faecalibacterium prausnitzii [taxid 853]: 1 (0.014%)<br>• other: 22 (0.308%)
Benchmark OTU ID: `CP001606-_Actinobacteria`<br>OTU taxon: Bifidobacterium animalis subsp. lactis DSM 10140 [taxid 555970]<br>Expected: Bifidobacterium animalis [taxid 28025] (species)<br>Number of reads: 7092<br>Number of identified reads: 7077 (99.788%)	• **species: 4602 (64.89%)**<br>• genus: 1168 (16.469%)<br>• family: 160 (2.256%)<br>• order: 0 (0.0%)<br>• class: 145 (2.044%)<br>• phylum: 18 (0.253%)<br>• superkingdom: 325 (4.582%)<br>• root: 653 (9.207%)	• Bifidobacterium pseudolongum [taxid 1694]: 6 (0.084%)<br>• Bifidobacterium longum [taxid 216816]: 6 (0.084%)<br>• Bifidobacterium magnum [taxid 1692]: 3 (0.042%)<br>• Bifidobacterium bifidum [taxid 1681]: 3 (0.042%)<br>• Bifidobacterium tsurumiense [taxid 356829]: 2 (0.028%)<br>• Bifidobacterium pseudocatenulatum [taxid 28026]: 2 (0.028%)<br>• Bifidobacterium bohemicum [taxid 638617]: 2 (0.028%)<br>• Bifidobacterium margollesii [taxid 2020964]: 1 (0.014%)<br>• other: 26 (0.366%)

Operational Taxonomic Unit (OTU)	Correct identifications	Wrong or overspecific identifications at species rank
Benchmark OTU ID: `CP001892-_Actinobacteria`<br>OTU taxon: Bifidobacterium animalis subsp. lactis V9 [taxid 573236]<br>Expected: Bifidobacterium animalis [taxid 28025] (species)<br>Number of reads: 7123<br>Number of identified reads: 7102 (99.705%)	• **species: 4524 (63.512%)**<br>• genus: 1222 (17.155%)<br>• family: 163 (2.288%)<br>• order: 0 (0.0%)<br>• class: 184 (2.583%)<br>• phylum: 12 (0.168%)<br>• superkingdom: 309 (4.338%)<br>• root: 679 (9.532%)	• Bifidobacterium pseudolongum [taxid 1694]: 8 (0.112%)<br>• Bifidobacterium aemilianum [taxid 2493120]: 3 (0.042%)<br>• Brugia timori [taxid 42155]: 3 (0.042%)<br>• Bifidobacterium longum [taxid 216816]: 2 (0.028%)<br>• Bifidobacterium magnum [taxid 1692]: 2 (0.028%)<br>• Bifidobacterium choloepi [taxid 2614131]: 2 (0.028%)<br>• Bifidobacterium bifidum [taxid 1681]: 2 (0.028%)<br>• Naasia lichenicola [taxid 2565933]: 1 (0.014%)<br>• other: 25 (0.35%)
Benchmark OTU ID: `CP001361-_Actinobacteria`<br>OTU taxon: Bifidobacterium bifidum BGN4 [taxid 484020]<br>Expected: Bifidobacterium bifidum [taxid 1681] (species)<br>Number of reads: 8493<br>Number of identified reads: 8463 (99.646%)	• **species: 4230 (49.805%)**<br>• genus: 2633 (31.002%)<br>• family: 203 (2.39%)<br>• order: 10 (0.117%)<br>• class: 169 (1.989%)<br>• phylum: 24 (0.282%)<br>• superkingdom: 399 (4.697%)<br>• root: 782 (9.207%)	• Bifidobacterium breve [taxid 1685]: 13 (0.153%)<br>• Bifidobacterium adolescentis [taxid 1680]: 7 (0.082%)<br>• Bifidobacterium longum [taxid 216816]: 5 (0.058%)<br>• Bifidobacterium scardovii [taxid 158787]: 5 (0.058%)<br>• Bifidobacterium margollesii [taxid 2020964]: 3 (0.035%)<br>• Bifidobacterium jacchi [taxid 2490545]: 3 (0.035%)<br>• Bifidobacterium tsurumiense [taxid 356829]: 3 (0.035%)<br>• Bifidobacterium hapali [taxid 1630172]: 3 (0.035%)<br>• other: 56 (0.659%)
Benchmark OTU ID: `CP002220-_Actinobacteria`<br>OTU taxon: Bifidobacterium bifidum S17 [taxid 883062]<br>Expected: Bifidobacterium bifidum [taxid 1681] (species)<br>Number of reads: 8312<br>Number of identified reads: 8261 (99.386%)	• **species: 4231 (50.902%)**<br>• genus: 2520 (30.317%)<br>• family: 199 (2.394%)<br>• order: 16 (0.192%)<br>• class: 156 (1.876%)<br>• phylum: 16 (0.192%)<br>• superkingdom: 415 (4.992%)<br>• root: 703 (8.457%)	• Bifidobacterium longum [taxid 216816]: 13 (0.156%)<br>• Bifidobacterium breve [taxid 1685]: 6 (0.072%)<br>• Bifidobacterium thermophilum [taxid 33905]: 5 (0.06%)<br>• Bifidobacterium pseudocatenulatum [taxid 28026]: 4 (0.048%)<br>• Bifidobacterium pullorum [taxid 78448]: 3 (0.036%)<br>• Bifidobacterium hapali [taxid 1630172]: 3 (0.036%)<br>• Bifidobacterium vespertilionis [taxid 2562524]: 3 (0.036%)<br>• Bifidobacterium aquikefiri [taxid 1653207]: 3 (0.036%)<br>• other: 42 (0.505%)
Benchmark OTU ID: `CP002743-_Actinobacteria`<br>OTU taxon: Bifidobacterium breve ACS-071-V-Sch8b [taxid 866777]<br>Expected: Bifidobacterium breve [taxid 1685] (species)<br>Number of reads: 9001<br>Number of identified reads: 8984 (99.811%)	• species: 1526 (16.953%)<br>• **genus: 5813 (64.581%)**<br>• family: 194 (2.155%)<br>• order: 1 (0.011%)<br>• class: 166 (1.844%)<br>• phylum: 15 (0.166%)<br>• superkingdom: 474 (5.266%)<br>• root: 780 (8.665%)	• Bifidobacterium longum [taxid 216816]: 123 (1.366%)<br>• Bifidobacterium bifidum [taxid 1681]: 26 (0.288%)<br>• Bifidobacterium scardovii [taxid 158787]: 6 (0.066%)<br>• Bifidobacterium callitrichidarum [taxid 2052941]: 5 (0.055%)<br>• Bifidobacterium myosotis [taxid 1630166]: 4 (0.044%)<br>• Bifidobacterium pseudolongum [taxid 1694]: 4 (0.044%)<br>• Bifidobacterium pseudocatenulatum [taxid 28026]: 4 (0.044%)<br>• Bifidobacterium pullorum [taxid 78448]: 4 (0.044%)<br>• other: 82 (0.911%)

Operational Taxonomic Unit (OTU)	Correct identifications	Wrong or overspecific identifications at species rank
Benchmark OTU ID: `CP000303-_Actinobacteria`<br>OTU taxon: Bifidobacterium breve UCC2003 [taxid 326426]<br>Expected: Bifidobacterium breve [taxid 1685] (species)<br>Number of reads: 9468<br>Number of identified reads: 9445 (99.757%)	• species: 1064 (11.237%)<br>• **genus: 6775 (71.556%)**<br>• family: 161 (1.7%)<br>• order: 6 (0.063%)<br>• class: 162 (1.711%)<br>• phylum: 35 (0.369%)<br>• superkingdom: 456 (4.816%)<br>• root: 779 (8.227%)	• Bifidobacterium longum [taxid 216816]: 115 (1.214%)<br>• Bifidobacterium bifidum [taxid 1681]: 60 (0.633%)<br>• Bifidobacterium myosotis [taxid 1630166]: 7 (0.073%)<br>• Bifidobacterium imperatoris [taxid 2020965]: 7 (0.073%)<br>• Bifidobacterium pseudocatenulatum [taxid 28026]: 6 (0.063%)<br>• Bifidobacterium scaligerum [taxid 2052656]: 6 (0.063%)<br>• Bifidobacterium adolescentis [taxid 1680]: 6 (0.063%)<br>• Bifidobacterium minimum [taxid 1693]: 5 (0.052%)<br>• other: 108 (1.14%)
Benchmark OTU ID: `CP001750-_Actinobacteria`<br>OTU taxon: Bifidobacterium dentium Bd1 [taxid 401473]<br>Expected: Bifidobacterium dentium [taxid 1689] (species)<br>Number of reads: 10514<br>Number of identified reads: 10488 (99.752%)	• species: 1778 (16.91%)<br>• **genus: 6745 (64.152%)**<br>• family: 199 (1.892%)<br>• order: 12 (0.114%)<br>• class: 213 (2.025%)<br>• phylum: 12 (0.114%)<br>• superkingdom: 503 (4.784%)<br>• root: 1019 (9.691%)	• Bifidobacterium moukalabense [taxid 1333651]: 23 (0.218%)<br>• Bifidobacterium adolescentis [taxid 1680]: 20 (0.19%)<br>• Bifidobacterium pseudocatenulatum [taxid 28026]: 15 (0.142%)<br>• Bifidobacterium longum [taxid 216816]: 11 (0.104%)<br>• Bifidobacterium catenulatum [taxid 1686]: 8 (0.076%)<br>• Bifidobacterium pseudolongum [taxid 1694]: 7 (0.066%)<br>• Bifidobacterium ruminantium [taxid 78346]: 6 (0.057%)<br>• Bifidobacterium mongoliense [taxid 518643]: 6 (0.057%)<br>• other: 110 (1.046%)
Benchmark OTU ID: `AP010889-_Actinobacteria`<br>OTU taxon: Bifidobacterium longum subsp. infantis ATCC 15697 = JCM 1222 = DSM 20088 [taxid 391904]<br>Expected: Bifidobacterium longum [taxid 216816] (species)<br>Number of reads: 11459<br>Number of identified reads: 11424 (99.694%)	• species: 3864 (33.72%)<br>• **genus: 5509 (48.075%)**<br>• family: 175 (1.527%)<br>• order: 1 (0.008%)<br>• class: 194 (1.692%)<br>• phylum: 13 (0.113%)<br>• superkingdom: 567 (4.948%)<br>• root: 1088 (9.494%)	• Bifidobacterium breve [taxid 1685]: 46 (0.401%)<br>• Bifidobacterium catenulatum [taxid 1686]: 12 (0.104%)<br>• Bifidobacterium adolescentis [taxid 1680]: 11 (0.095%)<br>• Bifidobacterium bifidum [taxid 1681]: 7 (0.061%)<br>• Bifidobacterium pseudocatenulatum [taxid 28026]: 6 (0.052%)<br>• Bifidobacterium animalis [taxid 28025]: 5 (0.043%)<br>• Bifidobacterium reuteri [taxid 983706]: 5 (0.043%)<br>• Bifidobacterium scaligerum [taxid 2052656]: 4 (0.034%)<br>• other: 94 (0.82%)
Benchmark OTU ID: `CP001095-_Actinobacteria`<br>OTU taxon: Bifidobacterium longum subsp. infantis ATCC 15697 = JCM 1222 = DSM 20088 [taxid 391904]<br>Expected: Bifidobacterium longum [taxid 216816] (species)<br>Number of reads: 11477<br>Number of identified reads: 11445 (99.721%)	• species: 3918 (34.137%)<br>• **genus: 5511 (48.017%)**<br>• family: 172 (1.498%)<br>• order: 4 (0.034%)<br>• class: 174 (1.516%)<br>• phylum: 17 (0.148%)<br>• superkingdom: 527 (4.591%)<br>• root: 1110 (9.671%)	• Bifidobacterium breve [taxid 1685]: 59 (0.514%)<br>• Bifidobacterium adolescentis [taxid 1680]: 14 (0.121%)<br>• Bifidobacterium reuteri [taxid 983706]: 11 (0.095%)<br>• Bifidobacterium bifidum [taxid 1681]: 7 (0.06%)<br>• Bifidobacterium callitrichidarum [taxid 2052941]: 6 (0.052%)<br>• Bifidobacterium vespertilionis [taxid 2562524]: 6 (0.052%)<br>• Bifidobacterium samirii [taxid 2306974]: 5 (0.043%)<br>• Bifidobacterium pseudocatenulatum [taxid 28026]: 5 (0.043%)<br>• other: 91 (0.792%)

Operational Taxonomic Unit (OTU)	Correct identifications	Wrong or overspecific identifications at species rank
Benchmark OTU ID: `CP002286-_Actinobacteria`<br>OTU taxon: Bifidobacterium longum subsp. longum BBMN68 [taxid 890402]<br>Expected: Bifidobacterium longum [taxid 216816] (species)<br>Number of reads: 8700<br>Number of identified reads: 8692 (99.908%)	• **species: 3681 (42.31%)**<br>• genus: 3489 (40.103%)<br>• family: 172 (1.977%)<br>• order: 0 (0.0%)<br>• class: 174 (2.0%)<br>• phylum: 16 (0.183%)<br>• superkingdom: 505 (5.804%)<br>• root: 650 (7.471%)	• Bifidobacterium breve [taxid 1685]: 28 (0.321%)<br>• Bifidobacterium bifidum [taxid 1681]: 12 (0.137%)<br>• Bifidobacterium simiarum [taxid 2045441]: 7 (0.08%)<br>• Bifidobacterium pullorum [taxid 78448]: 6 (0.068%)<br>• Bifidobacterium reuteri [taxid 983706]: 5 (0.057%)<br>• Bifidobacterium adolescentis [taxid 1680]: 5 (0.057%)<br>• Bifidobacterium catenulatum [taxid 1686]: 5 (0.057%)<br>• Bifidobacterium pseudocatenulatum [taxid 28026]: 5 (0.057%)<br>• other: 77 (0.885%)
Benchmark OTU ID: `FP929034-_Actinobacteria`<br>OTU taxon: Bifidobacterium longum subsp. longum F8 [taxid 722911]<br>Expected: Bifidobacterium longum [taxid 216816] (species)<br>Number of reads: 9186<br>Number of identified reads: 9167 (99.793%)	• species: 3596 (39.146%)<br>• **genus: 4073 (44.339%)**<br>• family: 150 (1.632%)<br>• order: 1 (0.01%)<br>• class: 162 (1.763%)<br>• phylum: 23 (0.25%)<br>• superkingdom: 493 (5.366%)<br>• root: 661 (7.195%)	• Bifidobacterium breve [taxid 1685]: 36 (0.391%)<br>• Bifidobacterium adolescentis [taxid 1680]: 7 (0.076%)<br>• Bifidobacterium catenulatum [taxid 1686]: 6 (0.065%)<br>• Bifidobacterium goeldii [taxid 2306975]: 5 (0.054%)<br>• Bifidobacterium pseudolongum [taxid 1694]: 4 (0.043%)<br>• Bifidobacterium callitrichos [taxid 762209]: 4 (0.043%)<br>• Bifidobacterium reuteri [taxid 983706]: 4 (0.043%)<br>• Bifidobacterium aerophilum [taxid 1798155]: 3 (0.032%)<br>• other: 62 (0.674%)
Benchmark OTU ID: `CP002794-_Actinobacteria`<br>OTU taxon: Bifidobacterium longum subsp. longum KACC 91563 [taxid 1035817]<br>Expected: Bifidobacterium longum [taxid 216816] (species)<br>Number of reads: 9283<br>Number of identified reads: 9254 (99.687%)	• species: 3755 (40.45%)<br>• **genus: 4010 (43.197%)**<br>• family: 166 (1.788%)<br>• order: 0 (0.0%)<br>• class: 158 (1.702%)<br>• phylum: 12 (0.129%)<br>• superkingdom: 455 (4.901%)<br>• root: 695 (7.486%)	• Bifidobacterium breve [taxid 1685]: 29 (0.312%)<br>• Bifidobacterium adolescentis [taxid 1680]: 14 (0.15%)<br>• Bifidobacterium bifidum [taxid 1681]: 12 (0.129%)<br>• Bifidobacterium reuteri [taxid 983706]: 4 (0.043%)<br>• Bifidobacterium scaligerum [taxid 2052656]: 4 (0.043%)<br>• Bifidobacterium samirii [taxid 2306974]: 4 (0.043%)<br>• Bifidobacterium jacchi [taxid 2490545]: 3 (0.032%)<br>• Bifidobacterium tsurumiense [taxid 356829]: 3 (0.032%)<br>• other: 70 (0.754%)
Benchmark OTU ID: `CP004346-_Actinobacteria`<br>OTU taxon: Bifidobacterium thermophilum RBL67 [taxid 1254439]<br>Expected: Bifidobacterium thermophilum [taxid 33905] (species)<br>Number of reads: 8826<br>Number of identified reads: 8790 (99.592%)	• species: 1632 (18.49%)<br>• **genus: 4774 (54.09%)**<br>• family: 336 (3.806%)<br>• order: 1 (0.011%)<br>• class: 203 (2.3%)<br>• phylum: 37 (0.419%)<br>• superkingdom: 468 (5.302%)<br>• root: 1334 (15.114%)	• Bifidobacterium porcinum [taxid 212365]: 29 (0.328%)<br>• Bifidobacterium thermacidophilum [taxid 246618]: 23 (0.26%)<br>• Bifidobacterium apri [taxid 1769423]: 21 (0.237%)<br>• Bifidobacterium boum [taxid 78343]: 14 (0.158%)<br>• Bifidobacterium longum [taxid 216816]: 10 (0.113%)<br>• Bifidobacterium adolescentis [taxid 1680]: 8 (0.09%)<br>• Bifidobacterium bifidum [taxid 1681]: 4 (0.045%)<br>• Bifidobacterium gallicum [taxid 78342]: 3 (0.033%)<br>• other: 61 (0.691%)

Operational Taxonomic Unit (OTU)	Correct identifications	Wrong or overspecific identifications at species rank
Benchmark OTU ID: `ENA|BX470250|BX470250.1-_Proteobacteria`<br>OTU taxon: Bordetella bronchiseptica [taxid 518]<br>Expected: Bordetella bronchiseptica [taxid 518] (species)<br>Number of reads: 11129<br>Number of identified reads: 11099 (99.73%)	• species: 880 (7.907%)<br>• **genus: 6640 (59.663%)**<br>• family: 1106 (9.937%)<br>• order: 452 (4.061%)<br>• class: 141 (1.266%)<br>• phylum: 711 (6.388%)<br>• superkingdom: 482 (4.331%)<br>• root: 680 (6.11%)	• Bordetella pertussis [taxid 520]: 48 (0.431%)<br>• Bordetella parapertussis [taxid 519]: 27 (0.242%)<br>• Bordetella petrii [taxid 94624]: 6 (0.053%)<br>• Bordetella ansorpii [taxid 288768]: 6 (0.053%)<br>• Bordetella trematum [taxid 123899]: 5 (0.044%)<br>• Bordetella hinzii [taxid 103855]: 4 (0.035%)<br>• Bordetella sp. N [taxid 1746199]: 3 (0.026%)<br>• Salmonella enterica [taxid 28901]: 3 (0.026%)<br>• other: 46 (0.413%)
Benchmark OTU ID: `HE965806-_Proteobacteria`<br>OTU taxon: Bordetella bronchiseptica 253 [taxid 568707]<br>Expected: Bordetella bronchiseptica [taxid 518] (species)<br>Number of reads: 10960<br>Number of identified reads: 10937 (99.79%)	• species: 1103 (10.063%)<br>• **genus: 6248 (57.007%)**<br>• family: 1118 (10.2%)<br>• order: 432 (3.941%)<br>• class: 117 (1.067%)<br>• phylum: 778 (7.098%)<br>• superkingdom: 464 (4.233%)<br>• root: 667 (6.085%)	• Bordetella pertussis [taxid 520]: 46 (0.419%)<br>• Bordetella parapertussis [taxid 519]: 33 (0.301%)<br>• Bordetella ansorpii [taxid 288768]: 8 (0.072%)<br>• Bordetella trematum [taxid 123899]: 5 (0.045%)<br>• Bordetella hinzii [taxid 103855]: 4 (0.036%)<br>• Bordetella petrii [taxid 94624]: 3 (0.027%)<br>• Achromobacter xylosoxidans [taxid 85698]: 2 (0.018%)<br>• Algicoccus marinus [taxid 2163011]: 2 (0.018%)<br>• other: 43 (0.392%)
Benchmark OTU ID: `ENA|BX470248|BX470248.1-_Proteobacteria`<br>OTU taxon: Bordetella pertussis [taxid 520]<br>Expected: Bordetella pertussis [taxid 520] (species)<br>Number of reads: 8310<br>Number of identified reads: 8288 (99.735%)	• species: 786 (9.458%)<br>• **genus: 4738 (57.015%)**<br>• family: 883 (10.625%)<br>• order: 334 (4.019%)<br>• class: 114 (1.371%)<br>• phylum: 597 (7.184%)<br>• superkingdom: 332 (3.995%)<br>• root: 498 (5.992%)	• Bordetella bronchiseptica [taxid 518]: 24 (0.288%)<br>• Bordetella parapertussis [taxid 519]: 15 (0.18%)<br>• Bordetella holmesii [taxid 35814]: 13 (0.156%)<br>• Bordetella ansorpii [taxid 288768]: 4 (0.048%)<br>• Bordetella sp. N [taxid 1746199]: 3 (0.036%)<br>• Taylorella asinigenitalis [taxid 84590]: 3 (0.036%)<br>• Bordetella avium [taxid 521]: 3 (0.036%)<br>• Bordetella petrii [taxid 94624]: 3 (0.036%)<br>• other: 34 (0.409%)
Benchmark OTU ID: `HE965805-_Proteobacteria`<br>OTU taxon: Bordetella pertussis 18323 [taxid 568706]<br>Expected: Bordetella pertussis [taxid 520] (species)<br>Number of reads: 8215<br>Number of identified reads: 8191 (99.707%)	• species: 718 (8.74%)<br>• **genus: 4837 (58.88%)**<br>• family: 912 (11.101%)<br>• order: 288 (3.505%)<br>• class: 88 (1.071%)<br>• phylum: 514 (6.256%)<br>• superkingdom: 335 (4.077%)<br>• root: 495 (6.025%)	• Bordetella bronchiseptica [taxid 518]: 33 (0.401%)<br>• Bordetella parapertussis [taxid 519]: 13 (0.158%)<br>• Bordetella holmesii [taxid 35814]: 10 (0.121%)<br>• Bordetella hinzii [taxid 103855]: 6 (0.073%)<br>• Bordetella ansorpii [taxid 288768]: 6 (0.073%)<br>• Bordetella bronchialis [taxid 463025]: 5 (0.06%)<br>• Bordetella petrii [taxid 94624]: 4 (0.048%)<br>• Bordetella trematum [taxid 123899]: 3 (0.036%)<br>• other: 31 (0.377%)

Operational Taxonomic Unit (OTU)	Correct identifications	Wrong or overspecific identifications at species rank
Benchmark OTU ID: `CP002695-_Proteobacteria`<br>OTU taxon: Bordetella pertussis CS [taxid 1017264]<br>Expected: Bordetella pertussis [taxid 520] (species)<br>Number of reads: 8394<br>Number of identified reads: 8383 (99.868%)	• species: 817 (9.733%)<br>• **genus: 4887 (58.22%)**<br>• family: 879 (10.471%)<br>• order: 306 (3.645%)<br>• class: 87 (1.036%)<br>• phylum: 542 (6.456%)<br>• superkingdom: 364 (4.336%)<br>• root: 498 (5.932%)	• Bordetella bronchiseptica [taxid 518]: 29 (0.345%)<br>• Bordetella parapertussis [taxid 519]: 9 (0.107%)<br>• Bordetella holmesii [taxid 35814]: 7 (0.083%)<br>• Bordetella petrii [taxid 94624]: 7 (0.083%)<br>• Bordetella hinzii [taxid 103855]: 5 (0.059%)<br>• Bordetella bronchialis [taxid 463025]: 3 (0.035%)<br>• Bordetella ansorpii [taxid 288768]: 3 (0.035%)<br>• Bordetella trematum [taxid 123899]: 3 (0.035%)<br>• other: 34 (0.405%)
Benchmark OTU ID: `AM902716-_Proteobacteria`<br>OTU taxon: Bordetella petrii [taxid 94624]<br>Expected: Bordetella petrii [taxid 94624] (species)<br>Number of reads: 11014<br>Number of identified reads: 10981 (99.7%)	• **species: 5855 (53.159%)**<br>• genus: 261 (2.369%)<br>• family: 1234 (11.203%)<br>• order: 585 (5.311%)<br>• class: 139 (1.262%)<br>• phylum: 1440 (13.074%)<br>• superkingdom: 532 (4.83%)<br>• root: 925 (8.398%)	• Achromobacter pulmonis [taxid 1389932]: 5 (0.045%)<br>• Bordetella flabilis [taxid 463014]: 2 (0.018%)<br>• Burkholderia multivorans [taxid 87883]: 2 (0.018%)<br>• Pseudomonas stutzeri [taxid 316]: 2 (0.018%)<br>• Xylella fastidiosa [taxid 2371]: 2 (0.018%)<br>• Pusillimonas thiosulfatoxidans [taxid 2028345]: 2 (0.018%)<br>• Pusillimonas noertemannii [taxid 305977]: 2 (0.018%)<br>• Pseudomonas aeruginosa [taxid 287]: 2 (0.018%)<br>• other: 76 (0.69%)
Benchmark OTU ID: `AP012279-_Rhizobium_Bradyrhizobium`<br>OTU taxon: Bradyrhizobium cosmicum [taxid 1404864]<br>Expected: Bradyrhizobium cosmicum [taxid 1404864] (species)<br>Number of reads: 204113<br>Number of identified reads: 203532 (99.715%)	• species: 27524 (13.484%)<br>• **genus: 116684 (57.166%)**<br>• family: 11832 (5.796%)<br>• order: 5384 (2.637%)<br>• class: 5854 (2.868%)<br>• phylum: 5986 (2.932%)<br>• superkingdom: 8247 (4.04%)<br>• root: 21847 (10.703%)	• Bradyrhizobium betae [taxid 244734]: 1840 (0.901%)<br>• Bradyrhizobium symbiodeficiens [taxid 1404367]: 498 (0.243%)<br>• Bradyrhizobium diazoefficiens [taxid 1355477]: 392 (0.192%)<br>• Bradyrhizobium japonicum [taxid 375]: 364 (0.178%)<br>• Bradyrhizobium erythrophlei [taxid 1437360]: 207 (0.101%)<br>• Bradyrhizobium yuanmingense [taxid 108015]: 189 (0.092%)<br>• Bradyrhizobium sp. Gha [taxid 1855318]: 182 (0.089%)<br>• Bradyrhizobium vignae [taxid 1549949]: 180 (0.088%)<br>• other: 2789 (1.366%)
Benchmark OTU ID: `BA000040-_Rhizobium_Bradyrhizobium`<br>OTU taxon: Bradyrhizobium diazoefficiens USDA 110 [taxid 224911]<br>Expected: Bradyrhizobium diazoefficiens [taxid 1355477] (species)<br>Number of reads: 260333<br>Number of identified reads: 259224 (99.574%)	• species: 24870 (9.553%)<br>• **genus: 170038 (65.315%)**<br>• family: 10263 (3.942%)<br>• order: 6382 (2.451%)<br>• class: 6721 (2.581%)<br>• phylum: 6549 (2.515%)<br>• superkingdom: 8876 (3.409%)<br>• root: 25322 (9.726%)	• Bradyrhizobium japonicum [taxid 375]: 1026 (0.394%)<br>• Bradyrhizobium erythrophlei [taxid 1437360]: 276 (0.106%)<br>• Bradyrhizobium vignae [taxid 1549949]: 218 (0.083%)<br>• Bradyrhizobium huanghuaihaiense [taxid 990078]: 182 (0.069%)<br>• Bradyrhizobium yuanmingense [taxid 108015]: 162 (0.062%)<br>• Bradyrhizobium betae [taxid 244734]: 148 (0.056%)<br>• Bradyrhizobium sp. Gha [taxid 1855318]: 148 (0.056%)<br>• Bradyrhizobium centrolobii [taxid 1505087]: 146 (0.056%)<br>• other: 2894 (1.111%)

Operational Taxonomic Unit (OTU)	Correct identifications	Wrong or overspecific identifications at species rank
Benchmark OTU ID: `AP012206-_Rhizobium_Bradyrhizobium`<br>OTU taxon: Bradyrhizobium japonicum USDA 6 [taxid 1037409]<br>Expected: Bradyrhizobium japonicum [taxid 375] (species)<br>Number of reads: 263378<br>Number of identified reads: 262166 (99.539%)	• species: 44237 (16.796%)<br>• **genus: 149963 (56.938%)**<br>• family: 10280 (3.903%)<br>• order: 6198 (2.353%)<br>• class: 6360 (2.414%)<br>• phylum: 6901 (2.62%)<br>• superkingdom: 9436 (3.582%)<br>• root: 28601 (10.859%)	• Bradyrhizobium diazoefficiens [taxid 1355477]: 720 (0.273%)<br>• Bradyrhizobium erythrophlei [taxid 1437360]: 226 (0.085%)<br>• Bradyrhizobium vignae [taxid 1549949]: 218 (0.082%)<br>• Bradyrhizobium arachidis [taxid 858423]: 175 (0.066%)<br>• Bradyrhizobium yuanmingense [taxid 108015]: 150 (0.056%)<br>• Bradyrhizobium huanghuaihaiense [taxid 990078]: 150 (0.056%)<br>• Bradyrhizobium betae [taxid 244734]: 137 (0.052%)<br>• Bradyrhizobium sp. Gha [taxid 1855318]: 137 (0.052%)<br>• other: 2798 (1.062%)
Benchmark OTU ID: `AP012603-_Proteobacteria`<br>OTU taxon: Bradyrhizobium oligotrophicum S58 [taxid 1245469]<br>Expected: Bradyrhizobium oligotrophicum [taxid 44255] (species)<br>Number of reads: 17710<br>Number of identified reads: 17630 (99.548%)	• **species: 7730 (43.647%)**<br>• genus: 2997 (16.922%)<br>• family: 2267 (12.8%)<br>• order: 591 (3.337%)<br>• class: 623 (3.517%)<br>• phylum: 637 (3.596%)<br>• superkingdom: 802 (4.528%)<br>• root: 1959 (11.061%)	• Bradyrhizobium erythrophlei [taxid 1437360]: 10 (0.056%)<br>• Bradyrhizobium nitroreducens [taxid 709803]: 4 (0.022%)<br>• Rhodopseudomonas palustris [taxid 1076]: 4 (0.022%)<br>• Bradyrhizobium japonicum [taxid 375]: 3 (0.016%)<br>• Bradyrhizobium diazoefficiens [taxid 1355477]: 3 (0.016%)<br>• Bradyrhizobium ivorense [taxid 2511166]: 2 (0.011%)<br>• Pseudolabrys sp. GY_H [taxid 2292256]: 2 (0.011%)<br>• Nitrobacter hamburgensis [taxid 912]: 2 (0.011%)<br>• other: 58 (0.327%)
Benchmark OTU ID: `CP000494-_Rhizobium_Bradyrhizobium`<br>OTU taxon: Bradyrhizobium sp. BTAi1 [taxid 288000]<br>Expected: Bradyrhizobium [taxid 374] (genus)<br>Number of reads: 235097<br>Number of identified reads: 234127 (99.587%)	• genus: 56360 (23.973%)<br>• **family: 111394 (47.382%)**<br>• order: 9448 (4.018%)<br>• class: 10198 (4.337%)<br>• phylum: 9991 (4.249%)<br>• superkingdom: 11421 (4.857%)<br>• root: 25110 (10.68%)	• Bradyrhizobium oligotrophicum [taxid 44255]: 397 (0.168%)<br>• Bradyrhizobium erythrophlei [taxid 1437360]: 179 (0.076%)<br>• Rhodopseudomonas palustris [taxid 1076]: 132 (0.056%)<br>• Bradyrhizobium lablabi [taxid 722472]: 106 (0.045%)<br>• Afipia broomeae [taxid 56946]: 78 (0.033%)<br>• Bradyrhizobium sp. Gha [taxid 1855318]: 71 (0.03%)<br>• Rhodoplanes serenus [taxid 200615]: 58 (0.024%)<br>• other: 1481 (0.629%)
Benchmark OTU ID: `CU234118-_Rhizobium_Bradyrhizobium`<br>OTU taxon: Bradyrhizobium sp. ORS 278 [taxid 114615]<br>Expected: Bradyrhizobium [taxid 374] (genus)<br>Number of reads: 210854<br>Number of identified reads: 209855 (99.526%)	• **genus: 127556 (60.494%)**<br>• family: 28709 (13.615%)<br>• order: 7223 (3.425%)<br>• class: 7755 (3.677%)<br>• phylum: 7089 (3.362%)<br>• superkingdom: 9243 (4.383%)<br>• root: 22083 (10.473%)	• Bradyrhizobium oligotrophicum [taxid 44255]: 404 (0.191%)<br>• Bradyrhizobium sp. Gha [taxid 1855318]: 202 (0.095%)<br>• Bradyrhizobium erythrophlei [taxid 1437360]: 151 (0.071%)<br>• Bradyrhizobium lablabi [taxid 722472]: 80 (0.037%)<br>• Bradyrhizobium sp. Ghvi [taxid 1855319]: 64 (0.03%)<br>• Rhodopseudomonas palustris [taxid 1076]: 60 (0.028%)<br>• Bradyrhizobium icense [taxid 1274631]: 42 (0.019%)<br>• other: 998 (0.473%)

Operational Taxonomic Unit (OTU)	Correct identifications	Wrong or overspecific identifications at species rank
Benchmark OTU ID: `AP008955-_Firmicutes`<br>OTU taxon: Brevibacillus brevis NBRC 100599 [taxid 358681]<br>Expected: Brevibacillus brevis [taxid 1393] (species)<br>Number of reads: 9305<br>Number of identified reads: 9262 (99.537%)	• species: 1268 (13.627%)<br>• genus: 578 (6.211%)<br>• family: 221 (2.375%)<br>• **order: 5527 (59.398%)**<br>• class: 20 (0.214%)<br>• phylum: 96 (1.031%)<br>• superkingdom: 452 (4.857%)<br>• root: 1087 (11.681%)	• Brevibacillus formosus [taxid 54913]: 26 (0.279%)<br>• Brevibacillus parabrevis [taxid 54914]: 20 (0.214%)<br>• Brevibacillus fortis [taxid 2126352]: 12 (0.128%)<br>• Brevibacillus antibioticus [taxid 2570228]: 12 (0.128%)<br>• Brevibacillus reuszeri [taxid 54915]: 8 (0.085%)<br>• Brevibacillus choshinensis [taxid 54911]: 6 (0.064%)<br>• Brevibacillus invocatus [taxid 173959]: 5 (0.053%)<br>• Brevibacillus gelatini [taxid 1655277]: 5 (0.053%)<br>• other: 73 (0.784%)
Benchmark OTU ID: `CP003176-_Pathogens`<br>OTU taxon: Brucella abortus A13334 [taxid 1104320]<br>Expected: Brucella abortus [taxid 235] (species)<br>Number of reads: 4095<br>Number of identified reads: 4089 (99.853%)	• species: 140 (3.418%)<br>• **genus: 2075 (50.671%)**<br>• family: 670 (16.361%)<br>• order: 442 (10.793%)<br>• class: 145 (3.54%)<br>• phylum: 90 (2.197%)<br>• superkingdom: 135 (3.296%)<br>• root: 389 (9.499%)	• Brucella ceti [taxid 120577]: 17 (0.415%)<br>• Brucella suis [taxid 29461]: 9 (0.219%)<br>• Brucella ovis [taxid 236]: 7 (0.17%)<br>• Brucella melitensis [taxid 29459]: 7 (0.17%)<br>• Brucella neotomae [taxid 29460]: 5 (0.122%)<br>• Brucella intermedia [taxid 94625]: 3 (0.073%)<br>• Rhizobium etli [taxid 29449]: 2 (0.048%)<br>• Ochrobactrum pecoris [taxid 867683]: 1 (0.024%)<br>• other: 21 (0.512%)
Benchmark OTU ID: `CP003177-_Pathogens`<br>OTU taxon: Brucella abortus A13334 [taxid 1104320]<br>Expected: Brucella abortus [taxid 235] (species)<br>Number of reads: 1692<br>Number of identified reads: 1689 (99.822%)	• species: 65 (3.841%)<br>• **genus: 981 (57.978%)**<br>• family: 199 (11.761%)<br>• order: 131 (7.742%)<br>• class: 48 (2.836%)<br>• phylum: 58 (3.427%)<br>• superkingdom: 53 (3.132%)<br>• root: 153 (9.042%)	• Brucella suis [taxid 29461]: 5 (0.295%)<br>• Brucella ceti [taxid 120577]: 5 (0.295%)<br>• Brucella ovis [taxid 236]: 4 (0.236%)<br>• Brucella canis [taxid 36855]: 3 (0.177%)<br>• Brucella melitensis [taxid 29459]: 2 (0.118%)<br>• Brucella neotomae [taxid 29460]: 2 (0.118%)<br>• Ochrobactrum anthropi [taxid 529]: 1 (0.059%)<br>• Xanthomonas bromi [taxid 56449]: 1 (0.059%)<br>• other: 3 (0.177%)
Benchmark OTU ID: `CP000887-_Pathogens`<br>OTU taxon: Brucella abortus S19 [taxid 430066]<br>Expected: Brucella abortus [taxid 235] (species)<br>Number of reads: 4092<br>Number of identified reads: 4086 (99.853%)	• species: 154 (3.763%)<br>• **genus: 1982 (48.435%)**<br>• family: 725 (17.717%)<br>• order: 477 (11.656%)<br>• class: 141 (3.445%)<br>• phylum: 72 (1.759%)<br>• superkingdom: 121 (2.956%)<br>• root: 410 (10.019%)	• Brucella suis [taxid 29461]: 13 (0.317%)<br>• Brucella melitensis [taxid 29459]: 12 (0.293%)<br>• Brucella ceti [taxid 120577]: 7 (0.171%)<br>• Brucella ovis [taxid 236]: 5 (0.122%)<br>• Brucella canis [taxid 36855]: 4 (0.097%)<br>• Brucella intermedia [taxid 94625]: 4 (0.097%)<br>• Brucella neotomae [taxid 29460]: 2 (0.048%)<br>• Ochrobactrum soli [taxid 2448455]: 2 (0.048%)<br>• other: 27 (0.659%)

Operational Taxonomic Unit (OTU)	Correct identifications	Wrong or overspecific identifications at species rank
Benchmark OTU ID: `CP000873-_Pathogens`<br>OTU taxon: Brucella canis ATCC 23365 [taxid 483179]<br>Expected: Brucella canis [taxid 36855] (species)<br>Number of reads: 1803<br>Number of identified reads: 1800 (99.833%)	• species: 20 (1.109%)<br>• **genus: 1043 (57.848%)**<br>• family: 242 (13.422%)<br>• order: 131 (7.265%)<br>• class: 62 (3.438%)<br>• phylum: 80 (4.437%)<br>• superkingdom: 51 (2.828%)<br>• root: 171 (9.484%)	• Brucella ovis [taxid 236]: 9 (0.499%)<br>• Brucella melitensis [taxid 29459]: 5 (0.277%)<br>• Brucella abortus [taxid 235]: 4 (0.221%)<br>• Brucella suis [taxid 29461]: 3 (0.166%)<br>• Falsochrobactrum shanghaiense [taxid 2201899]: 2 (0.11%)<br>• Brucella neotomae [taxid 29460]: 2 (0.11%)<br>• Brucella ceti [taxid 120577]: 2 (0.11%)<br>• Falsochrobactrum ovis [taxid 1293442]: 2 (0.11%)<br>• other: 9 (0.499%)
Benchmark OTU ID: `AE014291-_Pathogens`<br>OTU taxon: Brucella suis 1330 [taxid 204722]<br>Expected: Brucella suis [taxid 29461] (species)<br>Number of reads: 3984<br>Number of identified reads: 3979 (99.874%)	• species: 36 (0.903%)<br>• **genus: 2084 (52.309%)**<br>• family: 638 (16.014%)<br>• order: 466 (11.696%)<br>• class: 147 (3.689%)<br>• phylum: 81 (2.033%)<br>• superkingdom: 130 (3.263%)<br>• root: 395 (9.914%)	• Brucella melitensis [taxid 29459]: 11 (0.276%)<br>• Brucella ceti [taxid 120577]: 11 (0.276%)<br>• Brucella ovis [taxid 236]: 7 (0.175%)<br>• Brucella abortus [taxid 235]: 7 (0.175%)<br>• Brucella canis [taxid 36855]: 5 (0.125%)<br>• Brucella neotomae [taxid 29460]: 3 (0.075%)<br>• Brucella intermedia [taxid 94625]: 3 (0.075%)<br>• Ochrobactrum sp. Kaboul [taxid 2650568]: 2 (0.05%)<br>• other: 19 (0.476%)
Benchmark OTU ID: `CP000911-_Pathogens`<br>OTU taxon: Brucella suis ATCC 23445 [taxid 470137]<br>Expected: Brucella suis [taxid 29461] (species)<br>Number of reads: 3595<br>Number of identified reads: 3586 (99.749%)	• species: 76 (2.114%)<br>• **genus: 1815 (50.486%)**<br>• family: 605 (16.828%)<br>• order: 436 (12.127%)<br>• class: 98 (2.726%)<br>• phylum: 94 (2.614%)<br>• superkingdom: 122 (3.393%)<br>• root: 336 (9.346%)	• Brucella abortus [taxid 235]: 13 (0.361%)<br>• Brucella ceti [taxid 120577]: 12 (0.333%)<br>• Brucella melitensis [taxid 29459]: 11 (0.305%)<br>• Brucella ovis [taxid 236]: 9 (0.25%)<br>• Brucella intermedia [taxid 94625]: 5 (0.139%)<br>• Brucella pinnipedialis [taxid 120576]: 3 (0.083%)<br>• Falsochrobactrum shanghaiense [taxid 2201899]: 3 (0.083%)<br>• Ochrobactrum lupini [taxid 255457]: 2 (0.055%)<br>• other: 30 (0.834%)
Benchmark OTU ID: `CP000912-_Pathogens`<br>OTU taxon: Brucella suis ATCC 23445 [taxid 470137]<br>Expected: Brucella suis [taxid 29461] (species)<br>Number of reads: 2288<br>Number of identified reads: 2279 (99.606%)	• species: 61 (2.666%)<br>• **genus: 1281 (55.987%)**<br>• family: 303 (13.243%)<br>• order: 205 (8.959%)<br>• class: 49 (2.141%)<br>• phylum: 87 (3.802%)<br>• superkingdom: 67 (2.928%)<br>• root: 222 (9.702%)	• Brucella abortus [taxid 235]: 7 (0.305%)<br>• Brucella ovis [taxid 236]: 7 (0.305%)<br>• Brucella ceti [taxid 120577]: 6 (0.262%)<br>• Brucella melitensis [taxid 29459]: 3 (0.131%)<br>• Brucella pinnipedialis [taxid 120576]: 3 (0.131%)<br>• Brucella canis [taxid 36855]: 2 (0.087%)<br>• Brucella intermedia [taxid 94625]: 2 (0.087%)<br>• Aurantimonas aggregata [taxid 2047720]: 1 (0.043%)<br>• other: 19 (0.83%)

Operational Taxonomic Unit (OTU)	Correct identifications	Wrong or overspecific identifications at species rank
Benchmark OTU ID: `CP001025-_Proteobacteria`<br>OTU taxon: Burkholderia ambifaria MC40-6 [taxid 398577]<br>Expected: Burkholderia ambifaria [taxid 152480] (species)<br>Number of reads: 6864<br>Number of identified reads: 6846 (99.737%)	• species: 1039 (15.136%)<br>• **genus: 3835 (55.871%)**<br>• family: 735 (10.708%)<br>• order: 177 (2.578%)<br>• class: 48 (0.699%)<br>• phylum: 311 (4.53%)<br>• superkingdom: 239 (3.481%)<br>• root: 455 (6.628%)	• Burkholderia cenocepacia [taxid 95486]: 27 (0.393%)<br>• Burkholderia multivorans [taxid 87883]: 14 (0.203%)<br>• Burkholderia lata [taxid 482957]: 14 (0.203%)<br>• Burkholderia ubonensis [taxid 101571]: 11 (0.16%)<br>• Burkholderia vietnamiensis [taxid 60552]: 10 (0.145%)<br>• Burkholderia contaminans [taxid 488447]: 7 (0.101%)<br>• Burkholderia pseudomallei [taxid 28450]: 6 (0.087%)<br>• Burkholderia cepacia [taxid 292]: 5 (0.072%)<br>• other: 71 (1.034%)
Benchmark OTU ID: `CP000378-_Pathogens`<br>OTU taxon: Burkholderia cenocepacia AU 1054 [taxid 331271]<br>Expected: Burkholderia cenocepacia [taxid 95486] (species)<br>Number of reads: 7022<br>Number of identified reads: 6996 (99.629%)	• species: 847 (12.062%)<br>• **genus: 4156 (59.185%)**<br>• family: 657 (9.356%)<br>• order: 160 (2.278%)<br>• class: 52 (0.74%)<br>• phylum: 351 (4.998%)<br>• superkingdom: 242 (3.446%)<br>• root: 528 (7.519%)	• Burkholderia lata [taxid 482957]: 19 (0.27%)<br>• Burkholderia cepacia [taxid 292]: 11 (0.156%)<br>• Burkholderia pseudomallei [taxid 28450]: 10 (0.142%)<br>• Burkholderia ambifaria [taxid 152480]: 10 (0.142%)<br>• Burkholderia multivorans [taxid 87883]: 8 (0.113%)<br>• Burkholderia vietnamiensis [taxid 60552]: 8 (0.113%)<br>• Burkholderia ubonensis [taxid 101571]: 7 (0.099%)<br>• Burkholderia dolosa [taxid 152500]: 7 (0.099%)<br>• other: 64 (0.911%)
Benchmark OTU ID: `CP000379-_Pathogens`<br>OTU taxon: Burkholderia cenocepacia AU 1054 [taxid 331271]<br>Expected: Burkholderia cenocepacia [taxid 95486] (species)<br>Number of reads: 5757<br>Number of identified reads: 5742 (99.739%)	• species: 903 (15.685%)<br>• **genus: 3558 (61.803%)**<br>• family: 316 (5.488%)<br>• order: 68 (1.181%)<br>• class: 11 (0.191%)<br>• phylum: 240 (4.168%)<br>• superkingdom: 177 (3.074%)<br>• root: 469 (8.146%)	• Burkholderia lata [taxid 482957]: 15 (0.26%)<br>• Burkholderia multivorans [taxid 87883]: 9 (0.156%)<br>• Burkholderia ambifaria [taxid 152480]: 8 (0.138%)<br>• Burkholderia pyrrocinia [taxid 60550]: 8 (0.138%)<br>• Burkholderia cepacia [taxid 292]: 7 (0.121%)<br>• Burkholderia contaminans [taxid 488447]: 7 (0.121%)<br>• Burkholderia vietnamiensis [taxid 60552]: 6 (0.104%)<br>• Burkholderia seminalis [taxid 488731]: 5 (0.086%)<br>• other: 50 (0.868%)
Benchmark OTU ID: `CP000380-_Pathogens`<br>OTU taxon: Burkholderia cenocepacia AU 1054 [taxid 331271]<br>Expected: Burkholderia cenocepacia [taxid 95486] (species)<br>Number of reads: 1776<br>Number of identified reads: 1771 (99.718%)	• species: 266 (14.977%)<br>• **genus: 1031 (58.051%)**<br>• family: 153 (8.614%)<br>• order: 34 (1.914%)<br>• class: 5 (0.281%)<br>• phylum: 92 (5.18%)<br>• superkingdom: 63 (3.547%)<br>• root: 124 (6.981%)	• Burkholderia vietnamiensis [taxid 60552]: 3 (0.168%)<br>• Burkholderia cepacia [taxid 292]: 3 (0.168%)<br>• Burkholderia contaminans [taxid 488447]: 3 (0.168%)<br>• Burkholderia pseudomallei [taxid 28450]: 2 (0.112%)<br>• Burkholderia multivorans [taxid 87883]: 2 (0.112%)<br>• Burkholderia lata [taxid 482957]: 2 (0.112%)<br>• Ralstonia solanacearum [taxid 305]: 1 (0.056%)<br>• Burkholderia plantarii [taxid 41899]: 1 (0.056%)<br>• other: 9 (0.506%)

Operational Taxonomic Unit (OTU)	Correct identifications	Wrong or overspecific identifications at species rank
Benchmark OTU ID: `CP000458-_Pathogens`<br>OTU taxon: Burkholderia cenocepacia HI2424 [taxid 331272]<br>Expected: Burkholderia cenocepacia [taxid 95486] (species)<br>Number of reads: 7496<br>Number of identified reads: 7472 (99.679%)	• species: 688 (9.178%)<br>• **genus: 4642 (61.926%)**<br>• family: 779 (10.392%)<br>• order: 134 (1.787%)<br>• class: 58 (0.773%)<br>• phylum: 340 (4.535%)<br>• superkingdom: 298 (3.975%)<br>• root: 527 (7.03%)	• Burkholderia multivorans [taxid 87883]: 26 (0.346%)<br>• Burkholderia lata [taxid 482957]: 20 (0.266%)<br>• Burkholderia cepacia [taxid 292]: 12 (0.16%)<br>• Burkholderia ambifaria [taxid 152480]: 10 (0.133%)<br>• Burkholderia ubonensis [taxid 101571]: 9 (0.12%)<br>• Burkholderia vietnamiensis [taxid 60552]: 9 (0.12%)<br>• Burkholderia reimsis [taxid 2234132]: 8 (0.106%)<br>• Paraburkholderia tropica [taxid 92647]: 7 (0.093%)<br>• other: 88 (1.173%)
Benchmark OTU ID: `CP000459-_Pathogens`<br>OTU taxon: Burkholderia cenocepacia HI2424 [taxid 331272]<br>Expected: Burkholderia cenocepacia [taxid 95486] (species)<br>Number of reads: 6283<br>Number of identified reads: 6266 (99.729%)	• species: 970 (15.438%)<br>• **genus: 3868 (61.562%)**<br>• family: 335 (5.331%)<br>• order: 77 (1.225%)<br>• class: 14 (0.222%)<br>• phylum: 309 (4.918%)<br>• superkingdom: 199 (3.167%)<br>• root: 490 (7.798%)	• Burkholderia lata [taxid 482957]: 15 (0.238%)<br>• Burkholderia cepacia [taxid 292]: 11 (0.175%)<br>• Burkholderia multivorans [taxid 87883]: 9 (0.143%)<br>• Burkholderia contaminans [taxid 488447]: 8 (0.127%)<br>• Burkholderia ambifaria [taxid 152480]: 7 (0.111%)<br>• Burkholderia pyrrocinia [taxid 60550]: 7 (0.111%)<br>• Burkholderia ubonensis [taxid 101571]: 6 (0.095%)<br>• Burkholderia stabilis [taxid 95485]: 6 (0.095%)<br>• other: 51 (0.811%)
Benchmark OTU ID: `CP000959-_Pathogens`<br>OTU taxon: Burkholderia cenocepacia MC0-3 [taxid 406425]<br>Expected: Burkholderia cenocepacia [taxid 95486] (species)<br>Number of reads: 6821<br>Number of identified reads: 6792 (99.574%)	• species: 1183 (17.343%)<br>• **genus: 4073 (59.712%)**<br>• family: 361 (5.292%)<br>• order: 98 (1.436%)<br>• class: 16 (0.234%)<br>• phylum: 302 (4.427%)<br>• superkingdom: 226 (3.313%)<br>• root: 529 (7.755%)	• Burkholderia ambifaria [taxid 152480]: 16 (0.234%)<br>• Burkholderia lata [taxid 482957]: 15 (0.219%)<br>• Burkholderia cepacia [taxid 292]: 13 (0.19%)<br>• Burkholderia pyrrocinia [taxid 60550]: 8 (0.117%)<br>• Burkholderia multivorans [taxid 87883]: 8 (0.117%)<br>• Burkholderia contaminans [taxid 488447]: 7 (0.102%)<br>• Burkholderia ubonensis [taxid 101571]: 7 (0.102%)<br>• Burkholderia pseudomallei [taxid 28450]: 5 (0.073%)<br>• other: 50 (0.733%)
Benchmark OTU ID: `CP000010-_Pathogens`<br>OTU taxon: Burkholderia mallei ATCC 23344 [taxid 243160]<br>Expected: Burkholderia mallei [taxid 13373] (species)<br>Number of reads: 7561<br>Number of identified reads: 7518 (99.431%)	• species: 180 (2.38%)<br>• **genus: 5002 (66.155%)**<br>• family: 927 (12.26%)<br>• order: 171 (2.261%)<br>• class: 53 (0.7%)<br>• phylum: 337 (4.457%)<br>• superkingdom: 321 (4.245%)<br>• root: 519 (6.864%)	• **Burkholderia pseudomallei [taxid 28450]: 347 (4.589%)**<br>• Burkholderia thailandensis [taxid 57975]: 25 (0.33%)<br>• Burkholderia oklahomensis [taxid 342113]: 14 (0.185%)<br>• Burkholderia cenocepacia [taxid 95486]: 6 (0.079%)<br>• Burkholderia singularis [taxid 1503053]: 5 (0.066%)<br>• Trinickia caryophylli [taxid 28094]: 4 (0.052%)<br>• Paraburkholderia ribeironis [taxid 1247936]: 3 (0.039%)<br>• Lupinus albus [taxid 3870]: 2 (0.026%)<br>• other: 50 (0.661%)

Operational Taxonomic Unit (OTU)	Correct identifications	Wrong or overspecific identifications at species rank
Benchmark OTU ID: `CP000526-_Pathogens`<br>OTU taxon: Burkholderia mallei SAVP1 [taxid 320388]<br>Expected: Burkholderia mallei [taxid 13373] (species)<br>Number of reads: 7530<br>Number of identified reads: 7499 (99.588%)	• species: 201 (2.669%)<br>• **genus: 5031 (66.812%)**<br>• family: 916 (12.164%)<br>• order: 160 (2.124%)<br>• class: 80 (1.062%)<br>• phylum: 336 (4.462%)<br>• superkingdom: 304 (4.037%)<br>• root: 461 (6.122%)	• **Burkholderia pseudomallei [taxid 28450]: 314 (4.169%)**<br>• Burkholderia thailandensis [taxid 57975]: 21 (0.278%)<br>• Burkholderia singularis [taxid 1503053]: 14 (0.185%)<br>• Burkholderia oklahomensis [taxid 342113]: 9 (0.119%)<br>• Burkholderia cenocepacia [taxid 95486]: 4 (0.053%)<br>• Burkholderia multivorans [taxid 87883]: 3 (0.039%)<br>• Burkholderia cepacia [taxid 292]: 2 (0.026%)<br>• Robbsia andropogonis [taxid 28092]: 2 (0.026%)<br>• other: 41 (0.544%)
Benchmark OTU ID: `ENA|CM000833|CM000833.1-_Pathogens`<br>OTU taxon: Burkholderia pseudomallei 1710a [taxid 320371]<br>Expected: Burkholderia pseudomallei [taxid 28450] (species)<br>Number of reads: 6696<br>Number of identified reads: 6656 (99.402%)	• species: 1625 (24.268%)<br>• **genus: 3564 (53.225%)**<br>• family: 279 (4.166%)<br>• order: 59 (0.881%)<br>• class: 18 (0.268%)<br>• phylum: 273 (4.077%)<br>• superkingdom: 253 (3.778%)<br>• root: 580 (8.661%)	• Burkholderia mallei [taxid 13373]: 19 (0.283%)<br>• Burkholderia oklahomensis [taxid 342113]: 11 (0.164%)<br>• Burkholderia thailandensis [taxid 57975]: 9 (0.134%)<br>• Burkholderia singularis [taxid 1503053]: 4 (0.059%)<br>• Burkholderia ubonensis [taxid 101571]: 3 (0.044%)<br>• Burkholderia vietnamiensis [taxid 60552]: 3 (0.044%)<br>• Candidatus Kentron sp. FW [taxid 2126338]: 1 (0.014%)<br>• Paraburkholderia rhynchosiae [taxid 487049]: 1 (0.014%)<br>• other: 22 (0.328%)
Benchmark OTU ID: `CP000125-_Pathogens`<br>OTU taxon: Burkholderia pseudomallei 1710b [taxid 320372]<br>Expected: Burkholderia pseudomallei [taxid 28450] (species)<br>Number of reads: 6740<br>Number of identified reads: 6717 (99.658%)	• species: 1692 (25.103%)<br>• **genus: 3611 (53.575%)**<br>• family: 263 (3.902%)<br>• order: 58 (0.86%)<br>• class: 14 (0.207%)<br>• phylum: 249 (3.694%)<br>• superkingdom: 278 (4.124%)<br>• root: 542 (8.041%)	• Burkholderia mallei [taxid 13373]: 31 (0.459%)<br>• Burkholderia thailandensis [taxid 57975]: 19 (0.281%)<br>• Burkholderia oklahomensis [taxid 342113]: 12 (0.178%)<br>• Burkholderia singularis [taxid 1503053]: 6 (0.089%)<br>• Burkholderia ubonensis [taxid 101571]: 6 (0.089%)<br>• Burkholderia stabilis [taxid 95485]: 3 (0.044%)<br>• Burkholderia ambifaria [taxid 152480]: 2 (0.029%)<br>• Burkholderia vietnamiensis [taxid 60552]: 2 (0.029%)<br>• other: 20 (0.296%)
Benchmark OTU ID: `BX571966-_Pathogens`<br>OTU taxon: Burkholderia pseudomallei K96243 [taxid 272560]<br>Expected: Burkholderia pseudomallei [taxid 28450] (species)<br>Number of reads: 6719<br>Number of identified reads: 6693 (99.613%)	• species: 1674 (24.914%)<br>• **genus: 3539 (52.671%)**<br>• family: 249 (3.705%)<br>• order: 71 (1.056%)<br>• class: 14 (0.208%)<br>• phylum: 276 (4.107%)<br>• superkingdom: 280 (4.167%)<br>• root: 580 (8.632%)	• Burkholderia mallei [taxid 13373]: 24 (0.357%)<br>• Burkholderia thailandensis [taxid 57975]: 13 (0.193%)<br>• Burkholderia oklahomensis [taxid 342113]: 4 (0.059%)<br>• Burkholderia cepacia [taxid 292]: 2 (0.029%)<br>• Burkholderia virus phiE122 [taxid 431892]: 2 (0.029%)<br>• Burkholderia singularis [taxid 1503053]: 2 (0.029%)<br>• Burkholderia multivorans [taxid 87883]: 1 (0.014%)<br>• Ditylum brightwellii [taxid 49249]: 1 (0.014%)<br>• other: 18 (0.267%)

Operational Taxonomic Unit (OTU)	Correct identifications	Wrong or overspecific identifications at species rank
Benchmark OTU ID: `CP000086-_Proteobacteria`<br>OTU taxon: Burkholderia thailandensis E264 [taxid 271848]<br>Expected: Burkholderia thailandensis [taxid 57975] (species)<br>Number of reads: 7687<br>Number of identified reads: 7661 (99.661%)	• species: 2043 (26.577%)<br>• **genus: 2974 (38.688%)**<br>• family: 964 (12.54%)<br>• order: 168 (2.185%)<br>• class: 78 (1.014%)<br>• phylum: 395 (5.138%)<br>• superkingdom: 340 (4.423%)<br>• root: 689 (8.963%)	• Burkholderia pseudomallei [taxid 28450]: 67 (0.871%)<br>• Burkholderia singularis [taxid 1503053]: 10 (0.13%)<br>• Burkholderia oklahomensis [taxid 342113]: 7 (0.091%)<br>• Burkholderia mallei [taxid 13373]: 4 (0.052%)<br>• Burkholderia multivorans [taxid 87883]: 4 (0.052%)<br>• Burkholderia cepacia [taxid 292]: 3 (0.039%)<br>• Burkholderia vietnamiensis [taxid 60552]: 3 (0.039%)<br>• Burkholderia ambifaria [taxid 152480]: 2 (0.026%)<br>• other: 51 (0.663%)
Benchmark OTU ID: `CP001810-_Firmicutes`<br>OTU taxon: Butyrivibrio proteoclasticus B316 [taxid 515622]<br>Expected: Butyrivibrio proteoclasticus [taxid 43305] (species)<br>Number of reads: 4914<br>Number of identified reads: 4884 (99.389%)	• **species: 3035 (61.762%)**<br>• genus: 659 (13.41%)<br>• family: 121 (2.462%)<br>• order: 176 (3.581%)<br>• class: 3 (0.061%)<br>• phylum: 88 (1.79%)<br>• superkingdom: 242 (4.924%)<br>• root: 557 (11.334%)	• Ilumatobacter coccineus [taxid 467094]: 1 (0.02%)<br>• Bifidobacterium subtile [taxid 77635]: 1 (0.02%)<br>• Arabidopsis lyrata [taxid 59689]: 1 (0.02%)<br>• Lupinus albus [taxid 3870]: 1 (0.02%)<br>• Paenibacillus whitsoniae [taxid 2496558]: 1 (0.02%)<br>• Streptomyces coeruleorubidus [taxid 116188]: 1 (0.02%)<br>• Robiginitalea myxolifaciens [taxid 400055]: 1 (0.02%)<br>• Blautia obeum [taxid 40520]: 1 (0.02%)<br>• other: 2 (0.04%)
Benchmark OTU ID: `CP001393-_Firmicutes`<br>OTU taxon: Caldicellulosiruptor bescii DSM 6725 [taxid 521460]<br>Expected: Caldicellulosiruptor bescii [taxid 31899] (species)<br>Number of reads: 3903<br>Number of identified reads: 3847 (98.565%)	• species: 983 (25.185%)<br>• **genus: 1955 (50.089%)**<br>• family: 0 (0.0%)<br>• order: 42 (1.076%)<br>• class: 75 (1.921%)<br>• phylum: 57 (1.46%)<br>• superkingdom: 210 (5.38%)<br>• root: 521 (13.348%)	• Caldicellulosiruptor saccharolyticus [taxid 44001]: 13 (0.333%)<br>• Caldicellulosiruptor owensensis [taxid 55205]: 11 (0.281%)<br>• Caldicellulosiruptor kronotskyensis [taxid 413889]: 9 (0.23%)<br>• Caldicellulosiruptor hydrothermalis [taxid 413888]: 9 (0.23%)<br>• Caldicellulosiruptor changbaiensis [taxid 1222016]: 8 (0.204%)<br>• Caldicellulosiruptor obsidiansis [taxid 717609]: 7 (0.179%)<br>• Caldicellulosiruptor lactoaceticus [taxid 52766]: 4 (0.102%)<br>• Caldicellulosiruptor kristjanssonii [taxid 52765]: 1 (0.025%)<br>• other: 11 (0.281%)
Benchmark OTU ID: `CP002219-_Firmicutes`<br>OTU taxon: Caldicellulosiruptor hydrothermalis 108 [taxid 632292]<br>Expected: Caldicellulosiruptor hydrothermalis [taxid 413888] (species)<br>Number of reads: 3664<br>Number of identified reads: 3631 (99.099%)	• species: 1145 (31.25%)<br>• **genus: 1584 (43.231%)**<br>• family: 0 (0.0%)<br>• order: 19 (0.518%)<br>• class: 75 (2.046%)<br>• phylum: 72 (1.965%)<br>• superkingdom: 231 (6.304%)<br>• root: 504 (13.755%)	• Caldicellulosiruptor kronotskyensis [taxid 413889]: 11 (0.3%)<br>• Caldicellulosiruptor owensensis [taxid 55205]: 8 (0.218%)<br>• Caldicellulosiruptor bescii [taxid 31899]: 5 (0.136%)<br>• Caldicellulosiruptor changbaiensis [taxid 1222016]: 5 (0.136%)<br>• Caldicellulosiruptor obsidiansis [taxid 717609]: 4 (0.109%)<br>• Caldicellulosiruptor saccharolyticus [taxid 44001]: 3 (0.081%)<br>• Caldicellulosiruptor kristjanssonii [taxid 52765]: 3 (0.081%)<br>• Streptomyces griseocarneus [taxid 51201]: 1 (0.027%)<br>• other: 6 (0.163%)

Operational Taxonomic Unit (OTU)	Correct identifications	Wrong or overspecific identifications at species rank
Benchmark OTU ID: `CP002326-_Firmicutes`<br>OTU taxon: Caldicellulosiruptor kristjanssonii I77R1B [taxid 632335]<br>Expected: Caldicellulosiruptor kristjanssonii [taxid 52765] (species)<br>Number of reads: 3689<br>Number of identified reads: 3653 (99.024%)	• species: 615 (16.671%)<br>• **genus: 2071 (56.139%)**<br>• family: 1 (0.027%)<br>• order: 25 (0.677%)<br>• class: 69 (1.87%)<br>• phylum: 58 (1.572%)<br>• superkingdom: 230 (6.234%)<br>• root: 581 (15.749%)	• Caldicellulosiruptor lactoaceticus [taxid 52766]: 26 (0.704%)<br>• Caldicellulosiruptor bescii [taxid 31899]: 13 (0.352%)<br>• Caldicellulosiruptor hydrothermalis [taxid 413888]: 7 (0.189%)<br>• Caldicellulosiruptor obsidiansis [taxid 717609]: 6 (0.162%)<br>• Caldicellulosiruptor owensensis [taxid 55205]: 5 (0.135%)<br>• Caldicellulosiruptor saccharolyticus [taxid 44001]: 4 (0.108%)<br>• Caldicellulosiruptor kronotskyensis [taxid 413889]: 3 (0.081%)<br>• Caldicellulosiruptor changbaiensis [taxid 1222016]: 3 (0.081%)<br>• other: 10 (0.271%)
Benchmark OTU ID: `CP003001-_Firmicutes`<br>OTU taxon: Caldicellulosiruptor lactoaceticus 6A [taxid 632516]<br>Expected: Caldicellulosiruptor lactoaceticus [taxid 52766] (species)<br>Number of reads: 3511<br>Number of identified reads: 3461 (98.575%)	• species: 544 (15.494%)<br>• **genus: 1960 (55.824%)**<br>• family: 0 (0.0%)<br>• order: 22 (0.626%)<br>• class: 76 (2.164%)<br>• phylum: 59 (1.68%)<br>• superkingdom: 224 (6.379%)<br>• root: 573 (16.32%)	• Caldicellulosiruptor kristjanssonii [taxid 52765]: 17 (0.484%)<br>• Caldicellulosiruptor owensensis [taxid 55205]: 12 (0.341%)<br>• Caldicellulosiruptor saccharolyticus [taxid 44001]: 6 (0.17%)<br>• Caldicellulosiruptor hydrothermalis [taxid 413888]: 5 (0.142%)<br>• Caldicellulosiruptor bescii [taxid 31899]: 5 (0.142%)<br>• Caldicellulosiruptor changbaiensis [taxid 1222016]: 2 (0.056%)<br>• Geodermatophilus obscurus [taxid 1861]: 1 (0.028%)<br>• Streptococcus salivarius [taxid 1304]: 1 (0.028%)<br>• other: 8 (0.227%)
Benchmark OTU ID: `CP002216-_Firmicutes`<br>OTU taxon: Caldicellulosiruptor owensensis OL [taxid 632518]<br>Expected: Caldicellulosiruptor owensensis [taxid 55205] (species)<br>Number of reads: 3117<br>Number of identified reads: 3087 (99.037%)	• **species: 1150 (36.894%)**<br>• genus: 1143 (36.669%)<br>• family: 0 (0.0%)<br>• order: 28 (0.898%)<br>• class: 73 (2.341%)<br>• phylum: 63 (2.021%)<br>• superkingdom: 226 (7.25%)<br>• root: 401 (12.864%)	• Caldicellulosiruptor obsidiansis [taxid 717609]: 17 (0.545%)<br>• Caldicellulosiruptor hydrothermalis [taxid 413888]: 6 (0.192%)<br>• Caldicellulosiruptor changbaiensis [taxid 1222016]: 3 (0.096%)<br>• Caldicellulosiruptor saccharolyticus [taxid 44001]: 3 (0.096%)<br>• Caldicellulosiruptor kristjanssonii [taxid 52765]: 3 (0.096%)<br>• Caldicellulosiruptor kronotskyensis [taxid 413889]: 2 (0.064%)<br>• Gryllotalpicola protaetiae [taxid 2419771]: 1 (0.032%)<br>• Pseudoflavonifractor capillosus [taxid 106588]: 1 (0.032%)<br>• other: 7 (0.224%)
Benchmark OTU ID: `CP000679-_Firmicutes`<br>OTU taxon: Caldicellulosiruptor saccharolyticus DSM 8903 [taxid 351627]<br>Expected: Caldicellulosiruptor saccharolyticus [taxid 44001] (species)<br>Number of reads: 3983<br>Number of identified reads: 3925 (98.543%)	• species: 1422 (35.701%)<br>• **genus: 1511 (37.936%)**<br>• family: 1 (0.025%)<br>• order: 22 (0.552%)<br>• class: 74 (1.857%)<br>• phylum: 74 (1.857%)<br>• superkingdom: 265 (6.653%)<br>• root: 552 (13.858%)	• Caldicellulosiruptor changbaiensis [taxid 1222016]: 29 (0.728%)<br>• Caldicellulosiruptor bescii [taxid 31899]: 7 (0.175%)<br>• Caldicellulosiruptor hydrothermalis [taxid 413888]: 6 (0.15%)<br>• Caldicellulosiruptor kronotskyensis [taxid 413889]: 4 (0.1%)<br>• Caldicellulosiruptor owensensis [taxid 55205]: 3 (0.075%)<br>• Caldicellulosiruptor lactoaceticus [taxid 52766]: 2 (0.05%)<br>• Lentithecium fluviatile [taxid 690899]: 1 (0.025%)<br>• Caldicellulosiruptor obsidiansis [taxid 717609]: 1 (0.025%)<br>• other: 11 (0.276%)

Operational Taxonomic Unit (OTU)	Correct identifications	Wrong or overspecific identifications at species rank
Benchmark OTU ID: `AP012337-_Chloroflexi`<br>OTU taxon: Caldilinea aerophila DSM 14535 = NBRC 104270 [taxid 926550]<br>Expected: Caldilinea aerophila [taxid 133453] (species)<br>Number of reads: 130610<br>Number of identified reads: 130319 (99.777%)	• **species: 89949 (68.868%)**<br>• genus: 0 (0.0%)<br>• family: 329 (0.251%)<br>• order: 0 (0.0%)<br>• class: 173 (0.132%)<br>• phylum: 9501 (7.274%)<br>• superkingdom: 13833 (10.591%)<br>• root: 16408 (12.562%)	• Roseiflexus castenholzii [taxid 120962]: 9 (0.006%)<br>• Candidatus Acetothermum autotrophicum [taxid 1446466]: 9 (0.006%)<br>• Litorilinea aerophila [taxid 1204385]: 8 (0.006%)<br>• Thermanaerothrix daxensis [taxid 869279]: 6 (0.004%)<br>• Pyrinomonas methylaliphatogenes [taxid 454194]: 6 (0.004%)<br>• Gloeomargarita lithophora [taxid 1188228]: 5 (0.003%)<br>• Pacificimonas flava [taxid 1234595]: 5 (0.003%)<br>• Ditylum brightwellii [taxid 49249]: 4 (0.003%)<br>• other: 270 (0.206%)
Benchmark OTU ID: `CP003378-_Crenarchaeota`<br>OTU taxon: Caldisphaera lagunensis DSM 15908 [taxid 1056495]<br>Expected: Caldisphaera lagunensis [taxid 200415] (species)<br>Number of reads: 2992<br>Number of identified reads: 2900 (96.925%)	• **species: 2124 (70.989%)**<br>• genus: 119 (3.977%)<br>• family: 0 (0.0%)<br>• order: 14 (0.467%)<br>• class: 31 (1.036%)<br>• phylum: 1 (0.033%)<br>• superkingdom: 18 (0.601%)<br>• root: 570 (19.05%)	• Trichechus manatus [taxid 9778]: 1 (0.033%)<br>• Candidatus Altiarchaeales archaeon [taxid 2250256]: 1 (0.033%)<br>• Bifidobacterium angulatum [taxid 1683]: 1 (0.033%)<br>• Desulfurococcaceae archaeon [taxid 2184738]: 1 (0.033%)
Benchmark OTU ID: `CP000852-_Crenarchaeota`<br>OTU taxon: Caldivirga maquilingensis IC-167 [taxid 397948]<br>Expected: Caldivirga maquilingensis [taxid 76887] (species)<br>Number of reads: 4319<br>Number of identified reads: 4169 (96.526%)	• **species: 2688 (62.236%)**<br>• genus: 117 (2.708%)<br>• family: 63 (1.458%)<br>• order: 2 (0.046%)<br>• class: 36 (0.833%)<br>• phylum: 0 (0.0%)<br>• superkingdom: 23 (0.532%)<br>• root: 1180 (27.321%)	• Thermoprotei archaeon [taxid 2250277]: 3 (0.069%)<br>• Vulcanisaeta distributa [taxid 164451]: 2 (0.046%)<br>• Enterococcus canis [taxid 214095]: 1 (0.023%)<br>• Desulfurococcales archaeon [taxid 2480821]: 1 (0.023%)<br>• Thermoproteus uzoniensis [taxid 184117]: 1 (0.023%)<br>• Parashewanella spongiae [taxid 342950]: 1 (0.023%)
Benchmark OTU ID: `CP003610-_Cyanobacteria`<br>OTU taxon: Calothrix sp. PCC 6303 [taxid 1170562]<br>Expected: Calothrix parietina [taxid 32054] (species)<br>Number of reads: 40807<br>Number of identified reads: 40224 (98.571%)	• **species: 25462 (62.396%)**<br>• genus: 1265 (3.099%)<br>• family: 0 (0.0%)<br>• order: 2386 (5.847%)<br>• phylum: 2700 (6.616%)<br>• superkingdom: 1943 (4.761%)<br>• root: 6389 (15.656%)	• Calothrix elsteri [taxid 1247191]: 77 (0.188%)<br>• Calothrix brevissima [taxid 938406]: 6 (0.014%)<br>• Dolichospermum compactum [taxid 136073]: 6 (0.014%)<br>• Nostoc sphaeroides [taxid 446679]: 5 (0.012%)<br>• Tolypothrix campylonemoides [taxid 1136105]: 5 (0.012%)<br>• Calothrix parasitica [taxid 1973486]: 5 (0.012%)<br>• Mastigocoleus testarum [taxid 996925]: 4 (0.009%)<br>• other: 124 (0.303%)
Benchmark OTU ID: `CP003943-_Cyanobacteria`<br>OTU taxon: Calothrix sp. PCC 7507 [taxid 99598]<br>Expected: Calothrix [taxid 1186] (genus)<br>Number of reads: 42493<br>Number of identified reads: 41951 (98.724%)	• **genus: 22988 (54.098%)**<br>• family: 0 (0.0%)<br>• order: 6989 (16.447%)<br>• phylum: 3161 (7.438%)<br>• superkingdom: 2221 (5.226%)<br>• root: 6501 (15.298%)	• Calothrix parietina [taxid 32054]: 21 (0.049%)<br>• Calothrix brevissima [taxid 938406]: 15 (0.035%)<br>• Nostoc linckia [taxid 92942]: 13 (0.03%)<br>• Nostoc sphaeroides [taxid 446679]: 13 (0.03%)<br>• Nostoc punctiforme [taxid 272131]: 12 (0.028%)<br>• Nostoc flagelliforme [taxid 1306274]: 11 (0.025%)<br>• other: 262 (0.616%)

Operational Taxonomic Unit (OTU)	Correct identifications	Wrong or overspecific identifications at species rank
Benchmark OTU ID: `CP000792-_Proteobacteria`<br>OTU taxon: Campylobacter concisus 13826 [taxid 360104]<br>Expected: Campylobacter concisus [taxid 199] (species)<br>Number of reads: 3733<br>Number of identified reads: 3704 (99.223%)	• **species: 1579 (42.298%)**<br>• genus: 1511 (40.476%)<br>• family: 15 (0.401%)<br>• order: 41 (1.098%)<br>• class: 1 (0.026%)<br>• phylum: 83 (2.223%)<br>• superkingdom: 124 (3.321%)<br>• root: 349 (9.349%)	• Campylobacter ureolyticus [taxid 827]: 3 (0.08%)<br>• Helicobacter pylori [taxid 210]: 2 (0.053%)<br>• Campylobacter jejuni [taxid 197]: 2 (0.053%)<br>• Arcobacter cloacae [taxid 1054034]: 1 (0.026%)<br>• Campylobacter blaseri [taxid 2042961]: 1 (0.026%)<br>• Campylobacter fetus [taxid 196]: 1 (0.026%)<br>• Campylobacter mucosalis [taxid 202]: 1 (0.026%)<br>• Sulfurospirillum halorespirans [taxid 194424]: 1 (0.026%)<br>• other: 7 (0.187%)
Benchmark OTU ID: `CP000487-_Proteobacteria`<br>OTU taxon: Campylobacter fetus subsp. fetus 82-40 [taxid 360106]<br>Expected: Campylobacter fetus [taxid 196] (species)<br>Number of reads: 3107<br>Number of identified reads: 3065 (98.648%)	• species: 324 (10.428%)<br>• **genus: 2202 (70.872%)**<br>• family: 13 (0.418%)<br>• order: 57 (1.834%)<br>• class: 5 (0.16%)<br>• phylum: 65 (2.092%)<br>• superkingdom: 104 (3.347%)<br>• root: 291 (9.365%)	• Campylobacter jejuni [taxid 197]: 3 (0.096%)<br>• Campylobacter iguaniorum [taxid 1244531]: 3 (0.096%)<br>• Helicobacter pylori [taxid 210]: 3 (0.096%)<br>• Campylobacter sputorum [taxid 206]: 2 (0.064%)<br>• Campylobacter hyointestinalis [taxid 198]: 2 (0.064%)<br>• Campylobacter concisus [taxid 199]: 2 (0.064%)<br>• Campylobacter ureolyticus [taxid 827]: 1 (0.032%)<br>• Bombilactobacillus bombi [taxid 1303590]: 1 (0.032%)<br>• other: 12 (0.386%)
Benchmark OTU ID: `CP000776-_Proteobacteria`<br>OTU taxon: Campylobacter hominis ATCC BAA-381 [taxid 360107]<br>Expected: Campylobacter hominis [taxid 76517] (species)<br>Number of reads: 2966<br>Number of identified reads: 2885 (97.269%)	• **species: 2004 (67.565%)**<br>• genus: 220 (7.417%)<br>• family: 13 (0.438%)<br>• order: 46 (1.55%)<br>• class: 1 (0.033%)<br>• phylum: 57 (1.921%)<br>• superkingdom: 154 (5.192%)<br>• root: 389 (13.115%)	• Campylobacter ureolyticus [taxid 827]: 20 (0.674%)<br>• Campylobacter blaseri [taxid 2042961]: 2 (0.067%)<br>• Campylobacter iguaniorum [taxid 1244531]: 1 (0.033%)<br>• Campylobacter sputorum [taxid 206]: 1 (0.033%)<br>• Helicobacter ailurogastricus [taxid 1578720]: 1 (0.033%)<br>• Campylobacter coli [taxid 195]: 1 (0.033%)<br>• Campylobacter jejuni [taxid 197]: 1 (0.033%)<br>• Leuconostoc carnosum [taxid 1252]: 1 (0.033%)<br>• other: 5 (0.168%)
Benchmark OTU ID: `ENA|CM000855|CM000855.1-_Pathogens`<br>OTU taxon: Campylobacter jejuni subsp. jejuni 414 [taxid 683083]<br>Expected: Campylobacter jejuni [taxid 197] (species)<br>Number of reads: 2966<br>Number of identified reads: 2920 (98.449%)	• **species: 1326 (44.706%)**<br>• genus: 1222 (41.2%)<br>• family: 4 (0.134%)<br>• order: 41 (1.382%)<br>• class: 1 (0.033%)<br>• phylum: 39 (1.314%)<br>• superkingdom: 84 (2.832%)<br>• root: 202 (6.81%)	• Campylobacter novaezeelandiae [taxid 2267891]: 5 (0.168%)<br>• Campylobacter coli [taxid 195]: 4 (0.134%)<br>• Campylobacter hepaticus [taxid 1813019]: 4 (0.134%)<br>• Campylobacter taeniopygiae [taxid 2510188]: 3 (0.101%)<br>• Campylobacter avium [taxid 522485]: 2 (0.067%)<br>• Campylobacter pinnipediorum [taxid 1965231]: 2 (0.067%)<br>• Campylobacter cuniculorum [taxid 374106]: 2 (0.067%)<br>• Campylobacter lari [taxid 201]: 1 (0.033%)<br>• other: 3 (0.101%)

Operational Taxonomic Unit (OTU)	Correct identifications	Wrong or overspecific identifications at species rank
Benchmark OTU ID: `CP000814-_Pathogens`<br>OTU taxon: Campylobacter jejuni subsp. jejuni 81116 [taxid 407148]<br>Expected: Campylobacter jejuni [taxid 197] (species)<br>Number of reads: 2856<br>Number of identified reads: 2831 (99.124%)	• species: 192 (6.722%)<br>• **genus: 2364 (82.773%)**<br>• family: 6 (0.21%)<br>• order: 30 (1.05%)<br>• class: 4 (0.14%)<br>• phylum: 44 (1.54%)<br>• superkingdom: 73 (2.556%)<br>• root: 115 (4.026%)	• Campylobacter hepaticus [taxid 1813019]: 10 (0.35%)<br>• Campylobacter coli [taxid 195]: 8 (0.28%)<br>• Campylobacter cuniculorum [taxid 374106]: 4 (0.14%)<br>• Campylobacter novaezeelandiae [taxid 2267891]: 3 (0.105%)<br>• Campylobacter aviculae [taxid 2510190]: 3 (0.105%)<br>• Campylobacter geochelonis [taxid 1780362]: 2 (0.07%)<br>• Campylobacter insulaenigrae [taxid 260714]: 2 (0.07%)<br>• Campylobacter taeniopygiae [taxid 2510188]: 2 (0.07%)<br>• other: 13 (0.455%)
Benchmark OTU ID: `CP000932-_Proteobacteria`<br>OTU taxon: Campylobacter lari RM2100 [taxid 306263]<br>Expected: Campylobacter lari [taxid 201] (species)<br>Number of reads: 2548<br>Number of identified reads: 2524 (99.058%)	• species: 344 (13.5%)<br>• **genus: 1867 (73.273%)**<br>• family: 4 (0.156%)<br>• order: 29 (1.138%)<br>• class: 2 (0.078%)<br>• phylum: 30 (1.177%)<br>• superkingdom: 81 (3.178%)<br>• root: 165 (6.475%)	• Campylobacter peloridis [taxid 488546]: 8 (0.313%)<br>• Campylobacter jejuni [taxid 197]: 7 (0.274%)<br>• Campylobacter volucris [taxid 1031542]: 7 (0.274%)<br>• Campylobacter insulaenigrae [taxid 260714]: 6 (0.235%)<br>• Campylobacter coli [taxid 195]: 4 (0.156%)<br>• Campylobacter subantarcticus [taxid 497724]: 3 (0.117%)<br>• Campylobacter avium [taxid 522485]: 2 (0.078%)<br>• Campylobacter blaseri [taxid 2042961]: 1 (0.039%)<br>• other: 9 (0.353%)
Benchmark OTU ID: `CP001715-_Proteobacteria`<br>OTU taxon: Candidatus Accumulibacter phosphatis clade IIA str. UW-1 [taxid 522306]<br>Expected: Candidatus Accumulibacter phosphatis [taxid 327160] (species)<br>Number of reads: 10498<br>Number of identified reads: 10455 (99.59%)	• **species: 6016 (57.306%)**<br>• genus: 983 (9.363%)<br>• class: 800 (7.62%)<br>• phylum: 828 (7.887%)<br>• superkingdom: 577 (5.496%)<br>• root: 1236 (11.773%)	• Candidatus Accumulibacter aalborgensis [taxid 1860102]: 11 (0.104%)<br>• Rhodocyclus tenuis [taxid 1066]: 2 (0.019%)<br>• Propionivibrio dicarboxylicus [taxid 83767]: 2 (0.019%)<br>• Burkholderia vietnamiensis [taxid 60552]: 1 (0.009%)<br>• Pyrodinium bahamense [taxid 73915]: 1 (0.009%)<br>• Umezawaea tangerina [taxid 84725]: 1 (0.009%)<br>• other: 41 (0.39%)
Benchmark OTU ID: `CP001842-_Cyanobacteria`<br>OTU taxon: Candidatus Atelocyanobacterium thalassa isolate ALOHA [taxid 1453429]<br>Expected: Candidatus Atelocyanobacterium thalassa [taxid 713887] (species)<br>Number of reads: 5669<br>Number of identified reads: 5482 (96.701%)	• **species: 3987 (70.329%)**<br>• genus: 0 (0.0%)<br>• family: 65 (1.146%)<br>• order: 35 (0.617%)<br>• phylum: 362 (6.385%)<br>• superkingdom: 352 (6.209%)<br>• root: 663 (11.695%)	• Dorcoceras hygrometricum [taxid 472368]: 1 (0.017%)<br>• Deinococcus hopiensis [taxid 309885]: 1 (0.017%)<br>• Algicoccus marinus [taxid 2163011]: 1 (0.017%)<br>• Paraburkholderia phenazinium [taxid 60549]: 1 (0.017%)<br>• Artemisia annua [taxid 35608]: 1 (0.017%)<br>• Lupinus albus [taxid 3870]: 1 (0.017%)<br>• cyanobacterium endosymbiont of Epithemia turgida [taxid 718217]: 1 (0.017%)<br>• other: 12 (0.211%)

Operational Taxonomic Unit (OTU)	Correct identifications	Wrong or overspecific identifications at species rank
Benchmark OTU ID: `CP000860-_Firmicutes`<br>OTU taxon: Candidatus Desulforudis audaxviator MP104C [taxid 477974]<br>Expected: Candidatus Desulforudis audaxviator [taxid 471827] (species)<br>Number of reads: 2990<br>Number of identified reads: 2981 (99.698%)	• **species: 2086 (69.765%)**<br>• genus: 13 (0.434%)<br>• family: 13 (0.434%)<br>• order: 12 (0.401%)<br>• class: 25 (0.836%)<br>• phylum: 65 (2.173%)<br>• superkingdom: 336 (11.237%)<br>• root: 424 (14.18%)	• Plasmopara halstedii [taxid 4781]: 1 (0.033%)<br>• Candidatus Hakubanella thermoalkaliphilus [taxid 2754717]: 1 (0.033%)<br>• Borreliella garinii [taxid 29519]: 1 (0.033%)<br>• Methanomassiliicoccales archaeon [taxid 1906667]: 1 (0.033%)<br>• Prevotella oris [taxid 28135]: 1 (0.033%)<br>• Hevea brasiliensis [taxid 3981]: 1 (0.033%)<br>• Tepidibacillus decaturensis [taxid 1413211]: 1 (0.033%)<br>• Thermodesulfitimonas autotrophica [taxid 1894989]: 1 (0.033%)<br>• other: 3 (0.1%)
Benchmark OTU ID: `AP009510-_Elusimicrobia`<br>OTU taxon: Candidatus Endomicrobium trichonymphae [taxid 1408204]<br>Expected: Candidatus Endomicrobium trichonymphae [taxid 1408204] (species)<br>Number of reads: 39984<br>Number of identified reads: 39114 (97.824%)	• **species: 24072 (60.204%)**<br>• genus: 371 (0.927%)<br>• family: 0 (0.0%)<br>• order: 0 (0.0%)<br>• class: 0 (0.0%)<br>• phylum: 121 (0.302%)<br>• superkingdom: 3804 (9.513%)<br>• root: 10683 (26.718%)	• Endomicrobium proavitum [taxid 1408281]: 18 (0.045%)<br>• Helicobacter pylori [taxid 210]: 14 (0.035%)<br>• Candidatus Liberibacter americanus [taxid 309868]: 6 (0.015%)<br>• Enhydrobacter aerosaccus [taxid 225324]: 5 (0.012%)<br>• Agrobacterium deltaense [taxid 1183412]: 5 (0.012%)<br>• Candidatus Magnetobacterium bavaricum [taxid 29290]: 5 (0.012%)<br>• Melghirimyces algeriensis [taxid 910412]: 4 (0.01%)<br>• Hydrotalea sandarakina [taxid 1004304]: 3 (0.007%)<br>• other: 110 (0.275%)
Benchmark OTU ID: `CP000360-_Acidobacteria`<br>OTU taxon: Candidatus Koribacter versatilis Ellin345 [taxid 204669]<br>Expected: Candidatus Koribacter versatilis [taxid 658062] (species)<br>Number of reads: 451473<br>Number of identified reads: 450644 (99.816%)	• **species: 337713 (74.802%)**<br>• genus: 0 (0.0%)<br>• family: 1481 (0.328%)<br>• order: 121 (0.026%)<br>• class: 588 (0.13%)<br>• phylum: 12625 (2.796%)<br>• superkingdom: 39883 (8.833%)<br>• root: 57837 (12.81%)	• Candidatus Sulfotelmatobacter kueseliae [taxid 2042962]: 15 (0.003%)<br>• Granulicella tundricola [taxid 940615]: 13 (0.002%)<br>• Terriglobus roseus [taxid 392734]: 12 (0.002%)<br>• Edaphobacter aggregans [taxid 570835]: 11 (0.002%)<br>• Acidisarcina polymorpha [taxid 2211140]: 11 (0.002%)<br>• Candidatus Sulfotelmatomonas gaucii [taxid 2043161]: 10 (0.002%)<br>• Escherichia coli [taxid 562]: 8 (0.001%)<br>• Edaphobacter modestus [taxid 388466]: 8 (0.001%)<br>• other: 745 (0.165%)
Benchmark OTU ID: `CP001677-_Proteobacteria`<br>OTU taxon: Candidatus Liberibacter asiaticus str. psy62 [taxid 537021]<br>Expected: Candidatus Liberibacter asiaticus [taxid 34021] (species)<br>Number of reads: 1877<br>Number of identified reads: 1842 (98.135%)	• **species: 1189 (63.345%)**<br>• genus: 127 (6.766%)<br>• family: 12 (0.639%)<br>• order: 17 (0.905%)<br>• class: 29 (1.545%)<br>• phylum: 20 (1.065%)<br>• superkingdom: 56 (2.983%)<br>• root: 386 (20.564%)	• Candidatus Liberibacter solanacearum [taxid 556287]: 4 (0.213%)<br>• Candidatus Liberibacter africanus [taxid 34020]: 3 (0.159%)<br>• Hevea brasiliensis [taxid 3981]: 1 (0.053%)<br>• Beta vulgaris [taxid 161934]: 1 (0.053%)<br>• Helicobacter pylori [taxid 210]: 1 (0.053%)<br>• Pararhodospirillum photometricum [taxid 1084]: 1 (0.053%)<br>• Candidatus Liberibacter europaeus [taxid 744859]: 1 (0.053%)<br>• Candidatus Liberibacter americanus [taxid 309868]: 1 (0.053%)<br>• other: 4 (0.213%)

Operational Taxonomic Unit (OTU)	Correct identifications	Wrong or overspecific identifications at species rank
Benchmark OTU ID: `CP002371-_Proteobacteria`<br>OTU taxon: Candidatus Liberibacter solanacearum CLso-ZC1 [taxid 658172]<br>Expected: Candidatus Liberibacter solanacearum [taxid 556287] (species)<br>Number of reads: 1947<br>Number of identified reads: 1909 (98.048%)	• **species: 1353 (69.491%)**<br>• genus: 87 (4.468%)<br>• family: 5 (0.256%)<br>• order: 19 (0.975%)<br>• class: 30 (1.54%)<br>• phylum: 32 (1.643%)<br>• superkingdom: 62 (3.184%)<br>• root: 320 (16.435%)	• Candidatus Liberibacter asiaticus [taxid 34021]: 1 (0.051%)<br>• Hypnocyclicus thermotrophus [taxid 1627895]: 1 (0.051%)<br>• Breoghania corrubedonensis [taxid 665038]: 1 (0.051%)
Benchmark OTU ID: `CP000084-_Proteobacteria`<br>OTU taxon: Candidatus Pelagibacter ubique HTCC1062 [taxid 335992]<br>Expected: Candidatus Pelagibacter ubique [taxid 198252] (species)<br>Number of reads: 2061<br>Number of identified reads: 2034 (98.689%)	• **species: 1010 (49.005%)**<br>• genus: 323 (15.672%)<br>• family: 145 (7.035%)<br>• order: 58 (2.814%)<br>• class: 69 (3.347%)<br>• phylum: 93 (4.512%)<br>• superkingdom: 129 (6.259%)<br>• root: 204 (9.898%)	• Candidatus Pelagibacter giovannonii [taxid 2563896]: 3 (0.145%)<br>• Oribacterium sinus [taxid 237576]: 1 (0.048%)<br>• Terriglobus albidus [taxid 1592106]: 1 (0.048%)<br>• Pyramimonas parkeae [taxid 36894]: 1 (0.048%)<br>• Erythrobacter longus [taxid 1044]: 1 (0.048%)<br>• Enteractinococcus helveticum [taxid 1837282]: 1 (0.048%)
Benchmark OTU ID: `CP001751-_Proteobacteria`<br>OTU taxon: Candidatus Puniceispirillum marinum IMCC1322 [taxid 488538]<br>Expected: Candidatus Puniceispirillum marinum [taxid 767892] (species)<br>Number of reads: 5311<br>Number of identified reads: 5297 (99.736%)	• **species: 3866 (72.792%)**<br>• genus: 2 (0.037%)<br>• class: 466 (8.774%)<br>• phylum: 171 (3.219%)<br>• superkingdom: 188 (3.539%)<br>• root: 596 (11.221%)	• Phenylobacterium zucineum [taxid 284016]: 1 (0.018%)<br>• Mesorhizobium tamadayense [taxid 425306]: 1 (0.018%)<br>• Cyanoptyche gloeocystis [taxid 77922]: 1 (0.018%)<br>• Pararhodobacter oceanensis [taxid 2172121]: 1 (0.018%)<br>• Rickettsia endosymbiont of Culicoides newsteadi [taxid 1961830]: 1 (0.018%)<br>• Candidatus Kuenenia stuttgartiensis [taxid 174633]: 1 (0.018%)<br>• other: 9 (0.169%)
Benchmark OTU ID: `CP000473-_Acidobacteria`<br>OTU taxon: Candidatus Solibacter usitatus Ellin6076 [taxid 234267]<br>Expected: Candidatus Solibacter usitatus [taxid 332163] (species)<br>Number of reads: 838990<br>Number of identified reads: 836603 (99.715%)	• **species: 635844 (75.786%)**<br>• genus: 201 (0.023%)<br>• family: 7893 (0.94%)<br>• order: 1966 (0.234%)<br>• class: 5063 (0.603%)<br>• phylum: 12462 (1.485%)<br>• superkingdom: 75227 (8.966%)<br>• root: 97205 (11.585%)	• Pontibacter mucosus [taxid 1649266]: 22 (0.002%)<br>• Acidisarcina polymorpha [taxid 2211140]: 21 (0.002%)<br>• Pseudarthrobacter phenanthrenivorans [taxid 361575]: 14 (0.001%)<br>• bacterium [taxid 1869227]: 13 (0.001%)<br>• Edaphobacter aggregans [taxid 570835]: 11 (0.001%)<br>• Candidatus Koribacter versatilis [taxid 658062]: 11 (0.001%)<br>• Salmonella enterica [taxid 28901]: 10 (0.001%)<br>• Sorangium cellulosum [taxid 56]: 9 (0.001%)<br>• other: 1290 (0.153%)
Benchmark OTU ID: `CP001700-_Actinobacteria`<br>OTU taxon: Catenulispora acidiphila DSM 44928 [taxid 479433]<br>Expected: Catenulispora acidiphila [taxid 304895] (species)<br>Number of reads: 48889<br>Number of identified reads: 48618 (99.445%)	• **species: 33603 (68.733%)**<br>• genus: 596 (1.219%)<br>• family: 0 (0.0%)<br>• order: 5 (0.01%)<br>• class: 6108 (12.493%)<br>• phylum: 43 (0.087%)<br>• superkingdom: 3037 (6.212%)<br>• root: 5149 (10.532%)	• Streptomyces gilvigriseus [taxid 1428644]: 3 (0.006%)<br>• Gordonia alkanivorans [taxid 84096]: 2 (0.004%)<br>• Desulfovibrio magneticus [taxid 184917]: 2 (0.004%)<br>• Micromonospora lupini [taxid 285679]: 2 (0.004%)<br>• Streptomyces viridosporus [taxid 67581]: 2 (0.004%)<br>• Streptomyces griseoviridis [taxid 45398]: 2 (0.004%)<br>• Streptomyces misionensis [taxid 67331]: 2 (0.004%)<br>• Cutibacterium acnes [taxid 1747]: 2 (0.004%)<br>• other: 140 (0.286%)

Operational Taxonomic Unit (OTU)	Correct identifications	Wrong or overspecific identifications at species rank
Benchmark OTU ID: `CP002008-_Proteobacteria`<br>OTU taxon: Caulobacter segnis ATCC 21756 [taxid 509190]<br>Expected: Caulobacter segnis [taxid 88688] (species)<br>Number of reads: 9591<br>Number of identified reads: 9534 (99.405%)	• **species: 3715 (38.734%)**<br>• genus: 3251 (33.896%)<br>• family: 303 (3.159%)<br>• order: 27 (0.281%)<br>• class: 586 (6.109%)<br>• phylum: 408 (4.253%)<br>• superkingdom: 480 (5.004%)<br>• root: 757 (7.892%)	• Caulobacter sp. X [taxid 2048901]: 42 (0.437%)<br>• Caulobacter vibrioides [taxid 155892]: 23 (0.239%)<br>• Caulobacter henricii [taxid 69395]: 3 (0.031%)<br>• Caulobacter rhizosphaerae [taxid 2010972]: 2 (0.02%)<br>• Viridibacillus arvi [taxid 263475]: 1 (0.01%)<br>• Sporosarcina pasteurii [taxid 1474]: 1 (0.01%)<br>• Hibiscus syriacus [taxid 106335]: 1 (0.01%)<br>• Bartonella henselae [taxid 38323]: 1 (0.01%)<br>• other: 27 (0.281%)
Benchmark OTU ID: `AE005673-_Proteobacteria`<br>OTU taxon: Caulobacter vibrioides CB15 [taxid 190650]<br>Expected: Caulobacter vibrioides [taxid 155892] (species)<br>Number of reads: 8154<br>Number of identified reads: 8110 (99.46%)	• **species: 3661 (44.898%)**<br>• genus: 2355 (28.881%)<br>• family: 254 (3.115%)<br>• order: 21 (0.257%)<br>• class: 494 (6.058%)<br>• phylum: 281 (3.446%)<br>• superkingdom: 397 (4.868%)<br>• root: 639 (7.836%)	• Caulobacter segnis [taxid 88688]: 5 (0.061%)<br>• Lupinus albus [taxid 3870]: 3 (0.036%)<br>• Jannaschia rubra [taxid 282197]: 1 (0.012%)<br>• Candidatus Hakubanella thermoalkaliphilus [taxid 2754717]: 1 (0.012%)<br>• Candidatus Pelagibacter ubique [taxid 198252]: 1 (0.012%)<br>• Stenotrophomonas maltophilia [taxid 40324]: 1 (0.012%)<br>• Brevundimonas naejangsanensis [taxid 588932]: 1 (0.012%)<br>• Aquicella siphonis [taxid 254247]: 1 (0.012%)<br>• other: 15 (0.183%)
Benchmark OTU ID: `CP001340-_Proteobacteria`<br>OTU taxon: Caulobacter vibrioides NA1000 [taxid 565050]<br>Expected: Caulobacter vibrioides [taxid 155892] (species)<br>Number of reads: 8213<br>Number of identified reads: 8167 (99.439%)	• **species: 3774 (45.951%)**<br>• genus: 2325 (28.308%)<br>• family: 270 (3.287%)<br>• order: 18 (0.219%)<br>• class: 534 (6.501%)<br>• phylum: 281 (3.421%)<br>• superkingdom: 357 (4.346%)<br>• root: 602 (7.329%)	• Caulobacter sp. X [taxid 2048901]: 5 (0.06%)<br>• Caulobacter segnis [taxid 88688]: 3 (0.036%)<br>• Caulobacter henricii [taxid 69395]: 2 (0.024%)<br>• gamma proteobacterium symbiont of Ctena orbiculata [taxid 1968598]: 1 (0.012%)<br>• Gemmobacter aestuarii [taxid 1445661]: 1 (0.012%)<br>• Phenylobacterium parvum [taxid 2201350]: 1 (0.012%)<br>• Lichenibacterium minor [taxid 2316528]: 1 (0.012%)<br>• Hevea brasiliensis [taxid 3981]: 1 (0.012%)<br>• other: 20 (0.243%)
Benchmark OTU ID: `CP001964-_Actinobacteria`<br>OTU taxon: Cellulomonas flavigena DSM 20109 [taxid 446466]<br>Expected: Cellulomonas flavigena [taxid 1711] (species)<br>Number of reads: 17800<br>Number of identified reads: 17744 (99.685%)	• **species: 9915 (55.702%)**<br>• genus: 1941 (10.904%)<br>• family: 48 (0.269%)<br>• order: 1041 (5.848%)<br>• class: 1879 (10.556%)<br>• phylum: 23 (0.129%)<br>• superkingdom: 1308 (7.348%)<br>• root: 1570 (8.82%)	• Cellulomonas soli [taxid 931535]: 4 (0.022%)<br>• Cellulomonas shaoxiangyii [taxid 2566013]: 3 (0.016%)<br>• Kineococcus rhizosphaerae [taxid 559628]: 2 (0.011%)<br>• Cellulomonas gelida [taxid 1712]: 2 (0.011%)<br>• Cellulomonas uda [taxid 1714]: 2 (0.011%)<br>• Clavibacter michiganensis [taxid 28447]: 2 (0.011%)<br>• Salmonella enterica [taxid 28901]: 2 (0.011%)<br>• Cellulomonas aerilata [taxid 515326]: 2 (0.011%)<br>• other: 66 (0.37%)

Operational Taxonomic Unit (OTU)	Correct identifications	Wrong or overspecific identifications at species rank
Benchmark OTU ID: `CP002665-_Actinobacteria`<br>OTU taxon: Cellulomonas gilvus ATCC 13127 [taxid 593907]<br>Expected: Cellulomonas gilvus [taxid 11] (species)<br>Number of reads: 14876<br>Number of identified reads: 14833 (99.71%)	• **species: 8448 (56.789%)**<br>• genus: 1508 (10.137%)<br>• family: 54 (0.363%)<br>• order: 935 (6.285%)<br>• class: 1572 (10.567%)<br>• phylum: 15 (0.1%)<br>• superkingdom: 1087 (7.307%)<br>• root: 1205 (8.1%)	• Cellulomonas flavigena [taxid 1711]: 4 (0.026%)<br>• Cellulomonas persica [taxid 76861]: 4 (0.026%)<br>• Cellulomonas gelida [taxid 1712]: 3 (0.02%)<br>• Cellulomonas marina [taxid 988821]: 2 (0.013%)<br>• Krasilnikoviella flava [taxid 526729]: 2 (0.013%)<br>• Cellulomonas composti [taxid 266130]: 2 (0.013%)<br>• Cellulomonas uda [taxid 1714]: 2 (0.013%)<br>• Zhihengliuella halotolerans [taxid 370736]: 2 (0.013%)<br>• other: 63 (0.423%)
Benchmark OTU ID: `CP002453-_Bacteroidetes`<br>OTU taxon: Cellulophaga algicola DSM 14237 [taxid 688270]<br>Expected: Cellulophaga algicola [taxid 59600] (species)<br>Number of reads: 33340<br>Number of identified reads: 32889 (98.647%)	• **species: 18844 (56.52%)**<br>• genus: 3536 (10.605%)<br>• family: 4608 (13.821%)<br>• order: 304 (0.911%)<br>• class: 22 (0.065%)<br>• phylum: 960 (2.879%)<br>• superkingdom: 1498 (4.493%)<br>• root: 3083 (9.247%)	• Cellulophaga baltica [taxid 76594]: 94 (0.281%)<br>• Bizionia saleffrena [taxid 291189]: 40 (0.119%)<br>• Cellulophaga tyrosinoxydans [taxid 504486]: 10 (0.029%)<br>• Algibacter aquaticus [taxid 1849968]: 4 (0.011%)<br>• Cellulophaga fucicola [taxid 76595]: 4 (0.011%)<br>• Kriegella aquimaris [taxid 192904]: 4 (0.011%)<br>• Arenibacter nanhaiticus [taxid 558155]: 4 (0.011%)<br>• Maribacter orientalis [taxid 228957]: 3 (0.008%)<br>• other: 166 (0.497%)
Benchmark OTU ID: `CP002534-_Bacteroidetes`<br>OTU taxon: Cellulophaga lytica DSM 7489 [taxid 867900]<br>Expected: Cellulophaga lytica [taxid 979] (species)<br>Number of reads: 24810<br>Number of identified reads: 24528 (98.863%)	• species: 3101 (12.498%)<br>• **genus: 10324 (41.612%)**<br>• family: 7219 (29.097%)<br>• order: 305 (1.229%)<br>• class: 13 (0.052%)<br>• phylum: 722 (2.91%)<br>• superkingdom: 972 (3.917%)<br>• root: 1860 (7.496%)	• Cellulophaga fucicola [taxid 76595]: 48 (0.193%)<br>• Cellulophaga baltica [taxid 76594]: 6 (0.024%)<br>• Cellulophaga algicola [taxid 59600]: 5 (0.02%)<br>• Capnocytophaga canimorsus [taxid 28188]: 4 (0.016%)<br>• Maribacter vaceletii [taxid 1206816]: 4 (0.016%)<br>• Leptobacterium flavescens [taxid 472055]: 3 (0.012%)<br>• Ulvibacter litoralis [taxid 227084]: 3 (0.012%)<br>• Tenacibaculum skagerrakense [taxid 186571]: 3 (0.012%)<br>• other: 116 (0.467%)
Benchmark OTU ID: `CP000934-_Proteobacteria`<br>OTU taxon: Cellvibrio japonicus Ueda107 [taxid 498211]<br>Expected: Cellvibrio japonicus [taxid 155077] (species)<br>Number of reads: 9413<br>Number of identified reads: 9398 (99.84%)	• **species: 6416 (68.161%)**<br>• genus: 322 (3.42%)<br>• family: 24 (0.254%)<br>• order: 32 (0.339%)<br>• class: 818 (8.69%)<br>• phylum: 346 (3.675%)<br>• superkingdom: 334 (3.548%)<br>• root: 1101 (11.696%)	• Cellvibrio sp. BR [taxid 1134474]: 4 (0.042%)<br>• Rhipicephalus microplus [taxid 6941]: 1 (0.01%)<br>• Lupinus albus [taxid 3870]: 1 (0.01%)<br>• Thauera aminoaromatica [taxid 164330]: 1 (0.01%)<br>• Cellvibrio mixtus [taxid 39650]: 1 (0.01%)<br>• Pseudomonas lundensis [taxid 86185]: 1 (0.01%)<br>• Pseudomonas chlororaphis [taxid 587753]: 1 (0.01%)<br>• Halomonas axialensis [taxid 115555]: 1 (0.01%)<br>• other: 21 (0.223%)

Operational Taxonomic Unit (OTU)	Correct identifications	Wrong or overspecific identifications at species rank
Benchmark OTU ID: `CP003600-_Cyanobacteria`<br>OTU taxon: Chamaesiphon minutus PCC 6605 [taxid 1173020]<br>Expected: Chamaesiphon minutus [taxid 1173032] (species)<br>Number of reads: 37614<br>Number of identified reads: 37332 (99.25%)	• **species: 20956 (55.713%)**<br>• genus: 5237 (13.923%)<br>• family: 0 (0.0%)<br>• order: 353 (0.938%)<br>• phylum: 2010 (5.343%)<br>• superkingdom: 2419 (6.431%)<br>• root: 6285 (16.709%)	• Chamaesiphon polymorphus [taxid 2107691]: 268 (0.712%)<br>• Microcystis aeruginosa [taxid 1126]: 4 (0.01%)<br>• Pseudanabaena frigida [taxid 945775]: 3 (0.007%)<br>• Merismopedia glauca [taxid 292586]: 3 (0.007%)<br>• Leptolyngbya boryana [taxid 1184]: 2 (0.005%)<br>• Aphanothece sacrum [taxid 1122]: 2 (0.005%)<br>• Chroococcidiopsis cubana [taxid 171392]: 2 (0.005%)<br>• other: 99 (0.263%)
Benchmark OTU ID: `CP000390-_Proteobacteria`<br>OTU taxon: Chelativorans sp. BNC1 [taxid 266779]<br>Expected: Chelativorans [taxid 449972] (genus)<br>Number of reads: 9044<br>Number of identified reads: 9011 (99.635%)	• **genus: 5024 (55.55%)**<br>• family: 738 (8.16%)<br>• order: 771 (8.524%)<br>• class: 530 (5.86%)<br>• phylum: 297 (3.283%)<br>• superkingdom: 425 (4.699%)<br>• root: 1216 (13.445%)	• Lupinus albus [taxid 3870]: 5 (0.055%)<br>• Mesorhizobium soli [taxid 1295366]: 2 (0.022%)<br>• Rhodovulum visakhapatnamense [taxid 364297]: 1 (0.011%)<br>• Mesorhizobium helmanticense [taxid 1776423]: 1 (0.011%)<br>• Detonula confervacea [taxid 83371]: 1 (0.011%)<br>• Soonwooa buanensis [taxid 619805]: 1 (0.011%)<br>• Pannonibacter phragmitetus [taxid 121719]: 1 (0.011%)<br>• other: 42 (0.464%)
Benchmark OTU ID: `CP001699-_Bacteroidetes`<br>OTU taxon: Chitinophaga pinensis DSM 2588 [taxid 485918]<br>Expected: Chitinophaga pinensis [taxid 79329] (species)<br>Number of reads: 65556<br>Number of identified reads: 65321 (99.641%)	• **species: 30056 (45.847%)**<br>• genus: 21311 (32.508%)<br>• family: 559 (0.852%)<br>• order: 0 (0.0%)<br>• class: 0 (0.0%)<br>• phylum: 3330 (5.079%)<br>• superkingdom: 2734 (4.17%)<br>• root: 7285 (11.112%)	• Chitinophaga ginsengisoli [taxid 363837]: 26 (0.039%)<br>• Chitinophaga filiformis [taxid 104663]: 19 (0.028%)<br>• Chitinophaga japonensis [taxid 104662]: 15 (0.022%)<br>• Chitinophaga dinghuensis [taxid 1539050]: 10 (0.015%)<br>• Chitinophaga silvisoli [taxid 2291814]: 9 (0.013%)<br>• Chitinophaga oryziterrae [taxid 1031224]: 8 (0.012%)<br>• Chitinophaga parva [taxid 2169414]: 8 (0.012%)<br>• Chitinophaga sancti [taxid 1004]: 8 (0.012%)<br>• other: 196 (0.298%)
Benchmark OTU ID: `CP002514-_Acidobacteria`<br>OTU taxon: Chloracidobacterium thermophilum B [taxid 981222]<br>Expected: Chloracidobacterium thermophilum [taxid 458033] (species)<br>Number of reads: 185039<br>Number of identified reads: 184508 (99.713%)	• **species: 127609 (68.963%)**<br>• genus: 9443 (5.103%)<br>• class: 183 (0.098%)<br>• phylum: 1180 (0.637%)<br>• superkingdom: 21388 (11.558%)<br>• root: 24527 (13.255%)	• bacterium [taxid 1869227]: 9 (0.004%)<br>• Helicobacter pylori [taxid 210]: 8 (0.004%)<br>• Tanacetum cinerariifolium [taxid 118510]: 5 (0.002%)<br>• Martelella lutilitoris [taxid 2583532]: 5 (0.002%)<br>• Echinicola vietnamensis [taxid 390884]: 4 (0.002%)<br>• Blautia obeum [taxid 40520]: 3 (0.001%)<br>• other: 399 (0.215%)
Benchmark OTU ID: `CP001099-_Chlorobi`<br>OTU taxon: Chlorobaculum parvum NCIB 8327 [taxid 517417]<br>Expected: Chlorobaculum parvum [taxid 274539] (species)<br>Number of reads: 27607<br>Number of identified reads: 27559 (99.826%)	• **species: 16339 (59.184%)**<br>• genus: 3010 (10.903%)<br>• family: 1686 (6.107%)<br>• order: 12 (0.043%)<br>• class: 0 (0.0%)<br>• phylum: 4 (0.014%)<br>• superkingdom: 3210 (11.627%)<br>• root: 3280 (11.881%)	• Chlorobaculum tepidum [taxid 1097]: 37 (0.134%)<br>• Chlorobaculum thiosulfatiphilum [taxid 115852]: 14 (0.05%)<br>• Photorhabdus luminescens [taxid 29488]: 5 (0.018%)<br>• Chlorobaculum limnaeum [taxid 274537]: 5 (0.018%)<br>• Chlorobium phaeovibrioides [taxid 1094]: 4 (0.014%)<br>• Pelodictyon phaeoclathratiforme [taxid 34090]: 4 (0.014%)<br>• Marinimicrobium koreense [taxid 306545]: 3 (0.01%)<br>• Chlorobium phaeobacteroides [taxid 1096]: 3 (0.01%)<br>• other: 57 (0.206%)

Operational Taxonomic Unit (OTU)	Correct identifications	Wrong or overspecific identifications at species rank
Benchmark OTU ID: `AE006470-_Chlorobi`<br>OTU taxon: Chlorobaculum tepidum TLS [taxid 194439]<br>Expected: Chlorobaculum tepidum [taxid 1097] (species)<br>Number of reads: 25650<br>Number of identified reads: 25606 (99.828%)	• **species: 12897 (50.28%)**<br>• genus: 4762 (18.565%)<br>• family: 1547 (6.031%)<br>• order: 19 (0.074%)<br>• class: 0 (0.0%)<br>• phylum: 3 (0.011%)<br>• superkingdom: 3151 (12.284%)<br>• root: 3207 (12.502%)	• Chlorobaculum parvum [taxid 274539]: 79 (0.307%)<br>• Chlorobaculum limnaeum [taxid 274537]: 52 (0.202%)<br>• Chlorobaculum thiosulfatiphilum [taxid 115852]: 23 (0.089%)<br>• Streptococcus anginosus [taxid 1328]: 4 (0.015%)<br>• Chlorobium chlorochromatii [taxid 337090]: 3 (0.011%)<br>• Gemmata massiliana [taxid 1210884]: 2 (0.007%)<br>• Chlorobium limicola [taxid 1092]: 2 (0.007%)<br>• Chlorobium phaeobacteroides [taxid 1096]: 2 (0.007%)<br>• other: 62 (0.241%)
Benchmark OTU ID: `CP000108-_Chlorobi`<br>OTU taxon: Chlorobium chlorochromatii CaD3 [taxid 340177]<br>Expected: Chlorobium chlorochromatii [taxid 337090] (species)<br>Number of reads: 31722<br>Number of identified reads: 31621 (99.681%)	• **species: 22773 (71.789%)**<br>• genus: 537 (1.692%)<br>• family: 1210 (3.814%)<br>• order: 34 (0.107%)<br>• class: 0 (0.0%)<br>• phylum: 2 (0.006%)<br>• superkingdom: 3515 (11.08%)<br>• root: 3533 (11.137%)	• Chlorobaculum tepidum [taxid 1097]: 6 (0.018%)<br>• Pelodictyon phaeoclathratiforme [taxid 34090]: 4 (0.012%)<br>• Chlorobium phaeobacteroides [taxid 1096]: 3 (0.009%)<br>• Chlorobium ferrooxidans [taxid 84205]: 3 (0.009%)<br>• Chlorobaculum parvum [taxid 274539]: 3 (0.009%)<br>• Rickettsia philipii [taxid 1105106]: 2 (0.006%)<br>• Chlorobium limicola [taxid 1092]: 2 (0.006%)<br>• Chlorobium phaeovibrioides [taxid 1094]: 1 (0.003%)<br>• other: 53 (0.167%)
Benchmark OTU ID: `CP001097_rep10-_Chlorobi`<br>OTU taxon: Chlorobium limicola DSM 245 [taxid 290315]<br>Expected: Chlorobium limicola [taxid 1092] (species)<br>Number of reads: 34502<br>Number of identified reads: 34443 (99.828%)	• **species: 21408 (62.048%)**<br>• genus: 1202 (3.483%)<br>• family: 2043 (5.921%)<br>• order: 20 (0.057%)<br>• class: 0 (0.0%)<br>• phylum: 3 (0.008%)<br>• superkingdom: 3614 (10.474%)<br>• root: 6105 (17.694%)	• Chlorobium phaeobacteroides [taxid 1096]: 47 (0.136%)<br>• Chlorobaculum parvum [taxid 274539]: 14 (0.04%)<br>• Chlorobaculum limnaeum [taxid 274537]: 8 (0.023%)<br>• Chlorobaculum tepidum [taxid 1097]: 8 (0.023%)<br>• Chlorobium phaeovibrioides [taxid 1094]: 5 (0.014%)<br>• Chlorobium chlorochromatii [taxid 337090]: 5 (0.014%)<br>• Chlorobium ferrooxidans [taxid 84205]: 5 (0.014%)<br>• Prosthecochloris marina [taxid 2017681]: 5 (0.014%)<br>• other: 97 (0.281%)
Benchmark OTU ID: `CP001097_rep11-_Chlorobi`<br>OTU taxon: Chlorobium limicola DSM 245 [taxid 290315]<br>Expected: Chlorobium limicola [taxid 1092] (species)<br>Number of reads: 34502<br>Number of identified reads: 34425 (99.776%)	• **species: 21388 (61.99%)**<br>• genus: 1287 (3.73%)<br>• family: 2056 (5.959%)<br>• order: 15 (0.043%)<br>• class: 0 (0.0%)<br>• phylum: 2 (0.005%)<br>• superkingdom: 3619 (10.489%)<br>• root: 6018 (17.442%)	• Chlorobium phaeobacteroides [taxid 1096]: 54 (0.156%)<br>• Chlorobaculum tepidum [taxid 1097]: 7 (0.02%)<br>• Pelodictyon phaeoclathratiforme [taxid 34090]: 6 (0.017%)<br>• Chlorobaculum parvum [taxid 274539]: 6 (0.017%)<br>• Pelodictyon luteolum [taxid 1100]: 4 (0.011%)<br>• Chlorobium ferrooxidans [taxid 84205]: 4 (0.011%)<br>• Chlorobium phaeovibrioides [taxid 1094]: 3 (0.008%)<br>• Prosthecochloris marina [taxid 2017681]: 3 (0.008%)<br>• other: 85 (0.246%)

Operational Taxonomic Unit (OTU)	Correct identifications	Wrong or overspecific identifications at species rank
Benchmark OTU ID: `CP001097_rep12-_Chlorobi`<br>OTU taxon: Chlorobium limicola DSM 245 [taxid 290315]<br>Expected: Chlorobium limicola [taxid 1092] (species)<br>Number of reads: 34502<br>Number of identified reads: 34436 (99.808%)	• **species: 21265 (61.634%)**<br>• genus: 1277 (3.701%)<br>• family: 2044 (5.924%)<br>• order: 20 (0.057%)<br>• class: 0 (0.0%)<br>• phylum: 3 (0.008%)<br>• superkingdom: 3661 (10.61%)<br>• root: 6132 (17.772%)	• Chlorobium phaeobacteroides [taxid 1096]: 53 (0.153%)<br>• Chlorobaculum tepidum [taxid 1097]: 16 (0.046%)<br>• Chlorobaculum limnaeum [taxid 274537]: 12 (0.034%)<br>• Chlorobium ferrooxidans [taxid 84205]: 8 (0.023%)<br>• Prosthecochloris sp. ZM [taxid 2283143]: 7 (0.02%)<br>• Chlorobium phaeovibrioides [taxid 1094]: 7 (0.02%)<br>• Pelodictyon phaeoclathratiforme [taxid 34090]: 4 (0.011%)<br>• Chlorobaculum parvum [taxid 274539]: 4 (0.011%)<br>• other: 82 (0.237%)
Benchmark OTU ID: `CP001097_rep13-_Chlorobi`<br>OTU taxon: Chlorobium limicola DSM 245 [taxid 290315]<br>Expected: Chlorobium limicola [taxid 1092] (species)<br>Number of reads: 34502<br>Number of identified reads: 34428 (99.785%)	• **species: 21213 (61.483%)**<br>• genus: 1267 (3.672%)<br>• family: 2058 (5.964%)<br>• order: 18 (0.052%)<br>• class: 0 (0.0%)<br>• phylum: 1 (0.002%)<br>• superkingdom: 3652 (10.584%)<br>• root: 6187 (17.932%)	• Chlorobium phaeobacteroides [taxid 1096]: 48 (0.139%)<br>• Chlorobaculum parvum [taxid 274539]: 8 (0.023%)<br>• Chlorobaculum limnaeum [taxid 274537]: 8 (0.023%)<br>• Chlorobium chlorochromatii [taxid 337090]: 8 (0.023%)<br>• Chlorobium phaeovibrioides [taxid 1094]: 8 (0.023%)<br>• Prosthecochloris sp. ZM [taxid 2283143]: 5 (0.014%)<br>• Chlorobaculum tepidum [taxid 1097]: 5 (0.014%)<br>• Pelodictyon phaeoclathratiforme [taxid 34090]: 5 (0.014%)<br>• other: 73 (0.211%)
Benchmark OTU ID: `CP001097_rep14-_Chlorobi`<br>OTU taxon: Chlorobium limicola DSM 245 [taxid 290315]<br>Expected: Chlorobium limicola [taxid 1092] (species)<br>Number of reads: 34502<br>Number of identified reads: 34438 (99.814%)	• **species: 21183 (61.396%)**<br>• genus: 1274 (3.692%)<br>• family: 2123 (6.153%)<br>• order: 22 (0.063%)<br>• class: 0 (0.0%)<br>• phylum: 0 (0.0%)<br>• superkingdom: 3743 (10.848%)<br>• root: 6064 (17.575%)	• Chlorobium phaeobacteroides [taxid 1096]: 50 (0.144%)<br>• Pelodictyon phaeoclathratiforme [taxid 34090]: 11 (0.031%)<br>• Chlorobaculum tepidum [taxid 1097]: 7 (0.02%)<br>• Chlorobium ferrooxidans [taxid 84205]: 6 (0.017%)<br>• Chlorobium phaeovibrioides [taxid 1094]: 5 (0.014%)<br>• Chlorobaculum limnaeum [taxid 274537]: 5 (0.014%)<br>• Prosthecochloris sp. ZM [taxid 2283143]: 5 (0.014%)<br>• Prosthecochloris marina [taxid 2017681]: 4 (0.011%)<br>• other: 82 (0.237%)
Benchmark OTU ID: `CP001097_rep15-_Chlorobi`<br>OTU taxon: Chlorobium limicola DSM 245 [taxid 290315]<br>Expected: Chlorobium limicola [taxid 1092] (species)<br>Number of reads: 34502<br>Number of identified reads: 34425 (99.776%)	• **species: 21361 (61.912%)**<br>• genus: 1261 (3.654%)<br>• family: 2030 (5.883%)<br>• order: 16 (0.046%)<br>• class: 0 (0.0%)<br>• phylum: 4 (0.011%)<br>• superkingdom: 3697 (10.715%)<br>• root: 6012 (17.425%)	• Chlorobium phaeobacteroides [taxid 1096]: 47 (0.136%)<br>• Chlorobaculum limnaeum [taxid 274537]: 10 (0.028%)<br>• Chlorobaculum parvum [taxid 274539]: 10 (0.028%)<br>• Chlorobium phaeovibrioides [taxid 1094]: 8 (0.023%)<br>• Prosthecochloris marina [taxid 2017681]: 7 (0.02%)<br>• Chlorobium ferrooxidans [taxid 84205]: 7 (0.02%)<br>• Pelodictyon phaeoclathratiforme [taxid 34090]: 7 (0.02%)<br>• Prosthecochloris sp. ZM [taxid 2283143]: 6 (0.017%)<br>• other: 92 (0.266%)

Operational Taxonomic Unit (OTU)	Correct identifications	Wrong or overspecific identifications at species rank
Benchmark OTU ID: `CP001097_rep16-_Chlorobi`<br>OTU taxon: Chlorobium limicola DSM 245 [taxid 290315]<br>Expected: Chlorobium limicola [taxid 1092] (species)<br>Number of reads: 34502<br>Number of identified reads: 34438 (99.814%)	• **species: 21469 (62.225%)**<br>• genus: 1280 (3.709%)<br>• family: 2050 (5.941%)<br>• order: 27 (0.078%)<br>• class: 0 (0.0%)<br>• phylum: 3 (0.008%)<br>• superkingdom: 3624 (10.503%)<br>• root: 5936 (17.204%)	• Chlorobium phaeobacteroides [taxid 1096]: 46 (0.133%)<br>• Chlorobaculum tepidum [taxid 1097]: 12 (0.034%)<br>• Chlorobium phaeovibrioides [taxid 1094]: 11 (0.031%)<br>• Chlorobaculum parvum [taxid 274539]: 10 (0.028%)<br>• Pelodictyon phaeoclathratiforme [taxid 34090]: 7 (0.02%)<br>• Prosthecochloris sp. ZM [taxid 2283143]: 6 (0.017%)<br>• Chlorobium ferrooxidans [taxid 84205]: 6 (0.017%)<br>• Salmonella enterica [taxid 28901]: 5 (0.014%)<br>• other: 117 (0.339%)
Benchmark OTU ID: `CP001097_rep17-_Chlorobi`<br>OTU taxon: Chlorobium limicola DSM 245 [taxid 290315]<br>Expected: Chlorobium limicola [taxid 1092] (species)<br>Number of reads: 34502<br>Number of identified reads: 34457 (99.869%)	• **species: 21219 (61.5%)**<br>• genus: 1291 (3.741%)<br>• family: 2120 (6.144%)<br>• order: 22 (0.063%)<br>• class: 0 (0.0%)<br>• phylum: 3 (0.008%)<br>• superkingdom: 3703 (10.732%)<br>• root: 6053 (17.543%)	• Chlorobium phaeobacteroides [taxid 1096]: 47 (0.136%)<br>• Chlorobaculum tepidum [taxid 1097]: 9 (0.026%)<br>• Chlorobaculum limnaeum [taxid 274537]: 8 (0.023%)<br>• Chlorobaculum parvum [taxid 274539]: 8 (0.023%)<br>• Pelodictyon phaeoclathratiforme [taxid 34090]: 6 (0.017%)<br>• Chlorobium ferrooxidans [taxid 84205]: 5 (0.014%)<br>• Prosthecochloris marina [taxid 2017681]: 5 (0.014%)<br>• Chlorobium phaeovibrioides [taxid 1094]: 4 (0.011%)<br>• other: 83 (0.24%)
Benchmark OTU ID: `CP001097_rep18-_Chlorobi`<br>OTU taxon: Chlorobium limicola DSM 245 [taxid 290315]<br>Expected: Chlorobium limicola [taxid 1092] (species)<br>Number of reads: 34502<br>Number of identified reads: 34439 (99.817%)	• **species: 21208 (61.468%)**<br>• genus: 1330 (3.854%)<br>• family: 2063 (5.979%)<br>• order: 16 (0.046%)<br>• class: 0 (0.0%)<br>• phylum: 5 (0.014%)<br>• superkingdom: 3788 (10.979%)<br>• root: 5997 (17.381%)	• Chlorobium phaeobacteroides [taxid 1096]: 47 (0.136%)<br>• Chlorobaculum tepidum [taxid 1097]: 11 (0.031%)<br>• Chlorobaculum parvum [taxid 274539]: 9 (0.026%)<br>• Chlorobium ferrooxidans [taxid 84205]: 7 (0.02%)<br>• Pelodictyon phaeoclathratiforme [taxid 34090]: 6 (0.017%)<br>• Chlorobium chlorochromatii [taxid 337090]: 6 (0.017%)<br>• Prosthecochloris sp. ZM [taxid 2283143]: 4 (0.011%)<br>• Chlorobium phaeovibrioides [taxid 1094]: 4 (0.011%)<br>• other: 77 (0.223%)
Benchmark OTU ID: `CP001097_rep19-_Chlorobi`<br>OTU taxon: Chlorobium limicola DSM 245 [taxid 290315]<br>Expected: Chlorobium limicola [taxid 1092] (species)<br>Number of reads: 34502<br>Number of identified reads: 34443 (99.828%)	• **species: 21409 (62.051%)**<br>• genus: 1307 (3.788%)<br>• family: 2050 (5.941%)<br>• order: 25 (0.072%)<br>• class: 0 (0.0%)<br>• phylum: 2 (0.005%)<br>• superkingdom: 3606 (10.451%)<br>• root: 5999 (17.387%)	• Chlorobium phaeobacteroides [taxid 1096]: 59 (0.171%)<br>• Chlorobaculum limnaeum [taxid 274537]: 11 (0.031%)<br>• Chlorobium phaeovibrioides [taxid 1094]: 8 (0.023%)<br>• Chlorobaculum parvum [taxid 274539]: 7 (0.02%)<br>• Chlorobium chlorochromatii [taxid 337090]: 7 (0.02%)<br>• Chlorobium ferrooxidans [taxid 84205]: 6 (0.017%)<br>• Pelodictyon phaeoclathratiforme [taxid 34090]: 6 (0.017%)<br>• Prosthecochloris sp. ZM [taxid 2283143]: 5 (0.014%)<br>• other: 94 (0.272%)

Operational Taxonomic Unit (OTU)	Correct identifications	Wrong or overspecific identifications at species rank
Benchmark OTU ID: `CP001097_rep1-_Chlorobi`<br>OTU taxon: Chlorobium limicola DSM 245 [taxid 290315]<br>Expected: Chlorobium limicola [taxid 1092] (species)<br>Number of reads: 34502<br>Number of identified reads: 34420 (99.762%)	• **species: 21466 (62.216%)**<br>• genus: 1273 (3.689%)<br>• family: 1966 (5.698%)<br>• order: 15 (0.043%)<br>• class: 0 (0.0%)<br>• phylum: 1 (0.002%)<br>• superkingdom: 3621 (10.495%)<br>• root: 6041 (17.509%)	• Chlorobium phaeobacteroides [taxid 1096]: 52 (0.15%)<br>• Chlorobaculum limnaeum [taxid 274537]: 11 (0.031%)<br>• Chlorobium ferrooxidans [taxid 84205]: 11 (0.031%)<br>• Chlorobium phaeovibrioides [taxid 1094]: 8 (0.023%)<br>• Prosthecochloris sp. ZM [taxid 2283143]: 6 (0.017%)<br>• Prosthecochloris marina [taxid 2017681]: 4 (0.011%)<br>• Chlorobaculum parvum [taxid 274539]: 4 (0.011%)<br>• Pelodictyon luteolum [taxid 1100]: 3 (0.008%)<br>• other: 87 (0.252%)
Benchmark OTU ID: `CP001097_rep20-_Chlorobi`<br>OTU taxon: Chlorobium limicola DSM 245 [taxid 290315]<br>Expected: Chlorobium limicola [taxid 1092] (species)<br>Number of reads: 34502<br>Number of identified reads: 34425 (99.776%)	• **species: 21548 (62.454%)**<br>• genus: 1212 (3.512%)<br>• family: 2026 (5.872%)<br>• order: 22 (0.063%)<br>• class: 0 (0.0%)<br>• phylum: 1 (0.002%)<br>• superkingdom: 3661 (10.61%)<br>• root: 5911 (17.132%)	• Chlorobium phaeobacteroides [taxid 1096]: 61 (0.176%)<br>• Chlorobaculum tepidum [taxid 1097]: 10 (0.028%)<br>• Chlorobaculum limnaeum [taxid 274537]: 8 (0.023%)<br>• Chlorobaculum parvum [taxid 274539]: 6 (0.017%)<br>• Chlorobium ferrooxidans [taxid 84205]: 4 (0.011%)<br>• Prosthecochloris marina [taxid 2017681]: 4 (0.011%)<br>• Pelodictyon phaeoclathratiforme [taxid 34090]: 4 (0.011%)<br>• Prosthecochloris sp. ZM [taxid 2283143]: 4 (0.011%)<br>• other: 81 (0.234%)
Benchmark OTU ID: `CP001097_rep2-_Chlorobi`<br>OTU taxon: Chlorobium limicola DSM 245 [taxid 290315]<br>Expected: Chlorobium limicola [taxid 1092] (species)<br>Number of reads: 34502<br>Number of identified reads: 34438 (99.814%)	• **species: 21391 (61.999%)**<br>• genus: 1275 (3.695%)<br>• family: 2083 (6.037%)<br>• order: 19 (0.055%)<br>• class: 0 (0.0%)<br>• phylum: 1 (0.002%)<br>• superkingdom: 3643 (10.558%)<br>• root: 5992 (17.367%)	• Chlorobium phaeobacteroides [taxid 1096]: 49 (0.142%)<br>• Chlorobaculum tepidum [taxid 1097]: 13 (0.037%)<br>• Chlorobium ferrooxidans [taxid 84205]: 12 (0.034%)<br>• Chlorobaculum parvum [taxid 274539]: 11 (0.031%)<br>• Chlorobaculum limnaeum [taxid 274537]: 7 (0.02%)<br>• Pelodictyon phaeoclathratiforme [taxid 34090]: 7 (0.02%)<br>• Prosthecochloris sp. ZM [taxid 2283143]: 7 (0.02%)<br>• Prosthecochloris marina [taxid 2017681]: 6 (0.017%)<br>• other: 92 (0.266%)
Benchmark OTU ID: `CP001097_rep3-_Chlorobi`<br>OTU taxon: Chlorobium limicola DSM 245 [taxid 290315]<br>Expected: Chlorobium limicola [taxid 1092] (species)<br>Number of reads: 34502<br>Number of identified reads: 34429 (99.788%)	• **species: 21258 (61.613%)**<br>• genus: 1240 (3.593%)<br>• family: 2109 (6.112%)<br>• order: 14 (0.04%)<br>• class: 0 (0.0%)<br>• phylum: 4 (0.011%)<br>• superkingdom: 3716 (10.77%)<br>• root: 6044 (17.517%)	• Chlorobium phaeobacteroides [taxid 1096]: 50 (0.144%)<br>• Chlorobaculum limnaeum [taxid 274537]: 16 (0.046%)<br>• Pelodictyon luteolum [taxid 1100]: 10 (0.028%)<br>• Chlorobaculum parvum [taxid 274539]: 7 (0.02%)<br>• Chlorobaculum tepidum [taxid 1097]: 7 (0.02%)<br>• Chlorobium phaeovibrioides [taxid 1094]: 6 (0.017%)<br>• Pelodictyon phaeoclathratiforme [taxid 34090]: 4 (0.011%)<br>• Streptococcus anginosus [taxid 1328]: 4 (0.011%)<br>• other: 90 (0.26%)

Operational Taxonomic Unit (OTU)	Correct identifications	Wrong or overspecific identifications at species rank
Benchmark OTU ID: `CP001097_rep4-_Chlorobi`<br>OTU taxon: Chlorobium limicola DSM 245 [taxid 290315]<br>Expected: Chlorobium limicola [taxid 1092] (species)<br>Number of reads: 34502<br>Number of identified reads: 34435 (99.805%)	• **species: 21222 (61.509%)**<br>• genus: 1300 (3.767%)<br>• family: 2063 (5.979%)<br>• order: 22 (0.063%)<br>• class: 0 (0.0%)<br>• phylum: 0 (0.0%)<br>• superkingdom: 3697 (10.715%)<br>• root: 6093 (17.659%)	• Chlorobium phaeobacteroides [taxid 1096]: 42 (0.121%)<br>• Chlorobaculum limnaeum [taxid 274537]: 17 (0.049%)<br>• Chlorobaculum tepidum [taxid 1097]: 11 (0.031%)<br>• Chlorobium phaeovibrioides [taxid 1094]: 7 (0.02%)<br>• Chlorobium ferrooxidans [taxid 84205]: 7 (0.02%)<br>• Chlorobium chlorochromatii [taxid 337090]: 6 (0.017%)<br>• Prosthecochloris marina [taxid 2017681]: 5 (0.014%)<br>• Chlorobaculum parvum [taxid 274539]: 4 (0.011%)<br>• other: 106 (0.307%)
Benchmark OTU ID: `CP001097_rep5-_Chlorobi`<br>OTU taxon: Chlorobium limicola DSM 245 [taxid 290315]<br>Expected: Chlorobium limicola [taxid 1092] (species)<br>Number of reads: 34502<br>Number of identified reads: 34426 (99.779%)	• **species: 21390 (61.996%)**<br>• genus: 1325 (3.84%)<br>• family: 1987 (5.759%)<br>• order: 22 (0.063%)<br>• class: 0 (0.0%)<br>• phylum: 3 (0.008%)<br>• superkingdom: 3655 (10.593%)<br>• root: 6011 (17.422%)	• Chlorobium phaeobacteroides [taxid 1096]: 62 (0.179%)<br>• Chlorobaculum tepidum [taxid 1097]: 9 (0.026%)<br>• Prosthecochloris sp. ZM [taxid 2283143]: 8 (0.023%)<br>• Chlorobium phaeovibrioides [taxid 1094]: 7 (0.02%)<br>• Chlorobaculum limnaeum [taxid 274537]: 7 (0.02%)<br>• Chlorobaculum parvum [taxid 274539]: 7 (0.02%)<br>• Chlorobium chlorochromatii [taxid 337090]: 5 (0.014%)<br>• Pelodictyon phaeoclathratiforme [taxid 34090]: 5 (0.014%)<br>• other: 78 (0.226%)
Benchmark OTU ID: `CP001097_rep6-_Chlorobi`<br>OTU taxon: Chlorobium limicola DSM 245 [taxid 290315]<br>Expected: Chlorobium limicola [taxid 1092] (species)<br>Number of reads: 34502<br>Number of identified reads: 34444 (99.831%)	• **species: 21405 (62.039%)**<br>• genus: 1285 (3.724%)<br>• family: 2051 (5.944%)<br>• order: 22 (0.063%)<br>• class: 0 (0.0%)<br>• phylum: 2 (0.005%)<br>• superkingdom: 3612 (10.468%)<br>• root: 6027 (17.468%)	• Chlorobium phaeobacteroides [taxid 1096]: 50 (0.144%)<br>• Chlorobaculum limnaeum [taxid 274537]: 11 (0.031%)<br>• Prosthecochloris sp. ZM [taxid 2283143]: 8 (0.023%)<br>• Chlorobium ferrooxidans [taxid 84205]: 8 (0.023%)<br>• Chlorobium chlorochromatii [taxid 337090]: 7 (0.02%)<br>• Chlorobaculum parvum [taxid 274539]: 6 (0.017%)<br>• Chlorobium phaeovibrioides [taxid 1094]: 5 (0.014%)<br>• Pelodictyon luteolum [taxid 1100]: 4 (0.011%)<br>• other: 98 (0.284%)
Benchmark OTU ID: `CP001097_rep7-_Chlorobi`<br>OTU taxon: Chlorobium limicola DSM 245 [taxid 290315]<br>Expected: Chlorobium limicola [taxid 1092] (species)<br>Number of reads: 34502<br>Number of identified reads: 34413 (99.742%)	• **species: 21220 (61.503%)**<br>• genus: 1260 (3.651%)<br>• family: 2013 (5.834%)<br>• order: 11 (0.031%)<br>• class: 0 (0.0%)<br>• phylum: 2 (0.005%)<br>• superkingdom: 3685 (10.68%)<br>• root: 6186 (17.929%)	• Chlorobium phaeobacteroides [taxid 1096]: 42 (0.121%)<br>• Chlorobaculum tepidum [taxid 1097]: 12 (0.034%)<br>• Pelodictyon phaeoclathratiforme [taxid 34090]: 10 (0.028%)<br>• Chlorobaculum parvum [taxid 274539]: 7 (0.02%)<br>• Chlorobium ferrooxidans [taxid 84205]: 6 (0.017%)<br>• Prosthecochloris marina [taxid 2017681]: 5 (0.014%)<br>• Chlorobium phaeovibrioides [taxid 1094]: 5 (0.014%)<br>• Pelodictyon luteolum [taxid 1100]: 5 (0.014%)<br>• other: 80 (0.231%)

Operational Taxonomic Unit (OTU)	Correct identifications	Wrong or overspecific identifications at species rank
Benchmark OTU ID: `CP001097_rep8-_Chlorobi`<br>OTU taxon: Chlorobium limicola DSM 245 [taxid 290315]<br>Expected: Chlorobium limicola [taxid 1092] (species)<br>Number of reads: 34502<br>Number of identified reads: 34456 (99.866%)	• **species: 21292 (61.712%)**<br>• genus: 1303 (3.776%)<br>• family: 2044 (5.924%)<br>• order: 18 (0.052%)<br>• class: 0 (0.0%)<br>• phylum: 0 (0.0%)<br>• superkingdom: 3688 (10.689%)<br>• root: 6071 (17.596%)	• Chlorobium phaeobacteroides [taxid 1096]: 51 (0.147%)<br>• Chlorobaculum tepidum [taxid 1097]: 10 (0.028%)<br>• Chlorobaculum parvum [taxid 274539]: 9 (0.026%)<br>• Chlorobaculum limnaeum [taxid 274537]: 8 (0.023%)<br>• Pelodictyon phaeoclathratiforme [taxid 34090]: 5 (0.014%)<br>• Prosthecochloris marina [taxid 2017681]: 4 (0.011%)<br>• Chlorobium ferrooxidans [taxid 84205]: 4 (0.011%)<br>• Prosthecochloris sp. ZM [taxid 2283143]: 3 (0.008%)<br>• other: 78 (0.226%)
Benchmark OTU ID: `CP001097_rep9-_Chlorobi`<br>OTU taxon: Chlorobium limicola DSM 245 [taxid 290315]<br>Expected: Chlorobium limicola [taxid 1092] (species)<br>Number of reads: 34502<br>Number of identified reads: 34443 (99.828%)	• **species: 21194 (61.428%)**<br>• genus: 1343 (3.892%)<br>• family: 2109 (6.112%)<br>• order: 13 (0.037%)<br>• class: 0 (0.0%)<br>• phylum: 1 (0.002%)<br>• superkingdom: 3673 (10.645%)<br>• root: 6079 (17.619%)	• Chlorobium phaeobacteroides [taxid 1096]: 60 (0.173%)<br>• Pelodictyon phaeoclathratiforme [taxid 34090]: 7 (0.02%)<br>• Chlorobaculum tepidum [taxid 1097]: 7 (0.02%)<br>• Chlorobaculum parvum [taxid 274539]: 6 (0.017%)<br>• Chlorobium phaeovibrioides [taxid 1094]: 5 (0.014%)<br>• Chlorobium ferrooxidans [taxid 84205]: 5 (0.014%)<br>• Chlorobaculum limnaeum [taxid 274537]: 4 (0.011%)<br>• Prosthecochloris sp. ZM [taxid 2283143]: 4 (0.011%)<br>• other: 75 (0.217%)
Benchmark OTU ID: `CP001101-_Chlorobi`<br>OTU taxon: Chlorobium phaeobacteroides BS1 [taxid 331678]<br>Expected: Chlorobium phaeobacteroides [taxid 1096] (species)<br>Number of reads: 34114<br>Number of identified reads: 34011 (99.698%)	• species: 9451 (27.704%)<br>• genus: 224 (0.656%)<br>• **family: 14822 (43.448%)**<br>• order: 13 (0.038%)<br>• class: 0 (0.0%)<br>• phylum: 1 (0.002%)<br>• superkingdom: 3660 (10.728%)<br>• root: 5810 (17.031%)	• Chlorobium phaeovibrioides [taxid 1094]: 55 (0.161%)<br>• Prosthecochloris marina [taxid 2017681]: 50 (0.146%)<br>• Prosthecochloris aestuarii [taxid 1102]: 14 (0.041%)<br>• Prosthecochloris sp. ZM [taxid 2283143]: 11 (0.032%)<br>• Chlorobaculum parvum [taxid 274539]: 9 (0.026%)<br>• Chlorobaculum tepidum [taxid 1097]: 9 (0.026%)<br>• Chlorobium limicola [taxid 1092]: 8 (0.023%)<br>• Chlorobium chlorochromatii [taxid 337090]: 7 (0.02%)<br>• other: 115 (0.337%)
Benchmark OTU ID: `CP000492-_Chlorobi`<br>OTU taxon: Chlorobium phaeobacteroides DSM 266 [taxid 290317]<br>Expected: Chlorobium phaeobacteroides [taxid 1096] (species)<br>Number of reads: 39897<br>Number of identified reads: 39751 (99.634%)	• **species: 24188 (60.626%)**<br>• genus: 1539 (3.857%)<br>• family: 2405 (6.028%)<br>• order: 18 (0.045%)<br>• class: 0 (0.0%)<br>• phylum: 0 (0.0%)<br>• superkingdom: 3983 (9.983%)<br>• root: 7564 (18.958%)	• Chlorobium limicola [taxid 1092]: 63 (0.157%)<br>• Pelodictyon phaeoclathratiforme [taxid 34090]: 22 (0.055%)<br>• Chlorobaculum tepidum [taxid 1097]: 4 (0.01%)<br>• Chlorobaculum parvum [taxid 274539]: 4 (0.01%)<br>• Pelodictyon luteolum [taxid 1100]: 3 (0.007%)<br>• Chlorobium ferrooxidans [taxid 84205]: 3 (0.007%)<br>• Chlorobium phaeovibrioides [taxid 1094]: 3 (0.007%)<br>• Haloterrigena hispanica [taxid 392421]: 2 (0.005%)<br>• other: 99 (0.248%)

Operational Taxonomic Unit (OTU)	Correct identifications	Wrong or overspecific identifications at species rank
Benchmark OTU ID: `CP000607-_Chlorobi`<br>OTU taxon: Chlorobium phaeovibrioides DSM 265 [taxid 290318]<br>Expected: Chlorobium phaeovibrioides [taxid 1094] (species)<br>Number of reads: 22916<br>Number of identified reads: 22879 (99.838%)	• **species: 14388 (62.785%)**<br>• genus: 447 (1.95%)<br>• family: 2170 (9.469%)<br>• order: 16 (0.069%)<br>• class: 0 (0.0%)<br>• phylum: 4 (0.017%)<br>• superkingdom: 2355 (10.276%)<br>• root: 3477 (15.172%)	• Pelodictyon luteolum [taxid 1100]: 10 (0.043%)<br>• Chlorobium ferrooxidans [taxid 84205]: 4 (0.017%)<br>• Chlorobium chlorochromatii [taxid 337090]: 4 (0.017%)<br>• Chlorobaculum tepidum [taxid 1097]: 3 (0.013%)<br>• Chlorobium limicola [taxid 1092]: 2 (0.008%)<br>• Chlorobium phaeobacteroides [taxid 1096]: 2 (0.008%)<br>• Prosthecochloris sp. ZM [taxid 2283143]: 2 (0.008%)<br>• Chlorobaculum parvum [taxid 274539]: 2 (0.008%)<br>• other: 37 (0.161%)
Benchmark OTU ID: `CP001337-_Chloroflexi`<br>OTU taxon: Chloroflexus aggregans DSM 9485 [taxid 326427]<br>Expected: Chloroflexus aggregans [taxid 152260] (species)<br>Number of reads: 117871<br>Number of identified reads: 116978 (99.242%)	• **species: 50700 (43.013%)**<br>• genus: 5030 (4.267%)<br>• family: 0 (0.0%)<br>• order: 1359 (1.152%)<br>• class: 28 (0.023%)<br>• phylum: 27846 (23.624%)<br>• superkingdom: 12756 (10.822%)<br>• root: 19138 (16.236%)	• Chloroflexus islandicus [taxid 1707952]: 223 (0.189%)<br>• Roseiflexus castenholzii [taxid 120962]: 50 (0.042%)<br>• Oscillochloris trichoides [taxid 104176]: 28 (0.023%)<br>• Candidatus Chloroploca asiatica [taxid 1506545]: 24 (0.02%)<br>• Candidatus Viridilinea mediisalina [taxid 2024553]: 18 (0.015%)<br>• Candidatus Viridilinea halotolerans [taxid 2491704]: 17 (0.014%)<br>• Kouleothrix aurantiaca [taxid 186479]: 14 (0.011%)<br>• bacterium [taxid 1869227]: 4 (0.003%)<br>• other: 273 (0.231%)
Benchmark OTU ID: `CP000909-_Chloroflexi`<br>OTU taxon: Chloroflexus aurantiacus J-10-fl [taxid 324602]<br>Expected: Chloroflexus aurantiacus [taxid 1108] (species)<br>Number of reads: 133759<br>Number of identified reads: 132771 (99.261%)	• species: 3787 (2.831%)<br>• genus: 10160 (7.595%)<br>• family: 0 (0.0%)<br>• order: 467 (0.349%)<br>• class: 26 (0.019%)<br>• **phylum: 78946 (59.021%)**<br>• superkingdom: 13071 (9.772%)<br>• root: 26151 (19.55%)	• Chloroflexus aggregans [taxid 152260]: 202 (0.151%)<br>• Chloroflexus islandicus [taxid 1707952]: 202 (0.151%)<br>• Candidatus Chloroploca asiatica [taxid 1506545]: 57 (0.042%)<br>• Oscillochloris trichoides [taxid 104176]: 38 (0.028%)<br>• Candidatus Viridilinea mediisalina [taxid 2024553]: 25 (0.018%)<br>• Candidatus Viridilinea halotolerans [taxid 2491704]: 22 (0.016%)<br>• Roseiflexus castenholzii [taxid 120962]: 15 (0.011%)<br>• Kouleothrix aurantiaca [taxid 186479]: 11 (0.008%)<br>• other: 331 (0.247%)
Benchmark OTU ID: `CP001364-_Chloroflexi`<br>OTU taxon: Chloroflexus aurantiacus Y-400-fl [taxid 480224]<br>Expected: Chloroflexus aurantiacus [taxid 1108] (species)<br>Number of reads: 134048<br>Number of identified reads: 133097 (99.29%)	• species: 3705 (2.763%)<br>• genus: 10150 (7.571%)<br>• family: 0 (0.0%)<br>• order: 506 (0.377%)<br>• class: 15 (0.011%)<br>• **phylum: 79203 (59.085%)**<br>• superkingdom: 13175 (9.828%)<br>• root: 26190 (19.537%)	• Chloroflexus islandicus [taxid 1707952]: 222 (0.165%)<br>• Chloroflexus aggregans [taxid 152260]: 163 (0.121%)<br>• Oscillochloris trichoides [taxid 104176]: 51 (0.038%)<br>• Candidatus Chloroploca asiatica [taxid 1506545]: 47 (0.035%)<br>• Candidatus Viridilinea mediisalina [taxid 2024553]: 30 (0.022%)<br>• Candidatus Viridilinea halotolerans [taxid 2491704]: 24 (0.017%)<br>• Roseiflexus castenholzii [taxid 120962]: 10 (0.007%)<br>• Kouleothrix aurantiaca [taxid 186479]: 10 (0.007%)<br>• other: 317 (0.236%)

Operational Taxonomic Unit (OTU)	Correct identifications	Wrong or overspecific identifications at species rank
Benchmark OTU ID: `CP001100-_Chlorobi`<br>OTU taxon: Chloroherpeton thalassium ATCC 35110 [taxid 517418]<br>Expected: Chloroherpeton thalassium [taxid 100716] (species)<br>Number of reads: 42218<br>Number of identified reads: 42098 (99.715%)	• **species: 31974 (75.735%)**<br>• genus: 0 (0.0%)<br>• family: 246 (0.582%)<br>• order: 104 (0.246%)<br>• class: 0 (0.0%)<br>• phylum: 2 (0.004%)<br>• superkingdom: 4410 (10.445%)<br>• root: 5338 (12.643%)	• Desulfurivibrio alkaliphilus [taxid 427923]: 3 (0.007%)<br>• Chlorobium phaeobacteroides [taxid 1096]: 2 (0.004%)<br>• Chlorobaculum tepidum [taxid 1097]: 2 (0.004%)<br>• Candidatus Magnetoglobus multicellularis [taxid 418099]: 2 (0.004%)<br>• Marinomonas aquimarina [taxid 295068]: 2 (0.004%)<br>• Streptococcus mitis [taxid 28037]: 1 (0.002%)<br>• Paramoeba aestuarina [taxid 180227]: 1 (0.002%)<br>• Roseburia intestinalis [taxid 166486]: 1 (0.002%)<br>• other: 71 (0.168%)
Benchmark OTU ID: `CP003597-_Cyanobacteria`<br>OTU taxon: Chroococcidiopsis thermalis PCC 7203 [taxid 251229]<br>Expected: Chroococcidiopsis thermalis [taxid 54299] (species)<br>Number of reads: 37824<br>Number of identified reads: 37506 (99.159%)	• species: 2012 (5.319%)<br>• genus: 2458 (6.498%)<br>• family: 17 (0.044%)<br>• order: 0 (0.0%)<br>• **phylum: 25509 (67.441%)**<br>• superkingdom: 2303 (6.088%)<br>• root: 5127 (13.554%)	• Chroococcidiopsis cubana [taxid 171392]: 262 (0.692%)<br>• Cyanosarcina burmensis [taxid 2107696]: 137 (0.362%)<br>• Aliterella atlantica [taxid 1827278]: 10 (0.026%)<br>• Merismopedia glauca [taxid 292586]: 8 (0.021%)<br>• Chlorogloeopsis fritschii [taxid 1124]: 7 (0.018%)<br>• Leptolyngbya sp. 'hensonii' [taxid 1922337]: 6 (0.015%)<br>• Microcystis aeruginosa [taxid 1126]: 6 (0.015%)<br>• other: 238 (0.629%)
Benchmark OTU ID:<br>`Turtle_chrM_JH584390_JH584391-_Eukaryotes`<br>OTU taxon: Chrysemys picta bellii [taxid 8478]<br>Expected: Chrysemys picta [taxid 8479] (species)<br>Number of reads: 692<br>Number of identified reads: 642 (92.774%)	• species: 32 (4.624%)<br>• genus: 0 (0.0%)<br>• family: 20 (2.89%)<br>• order: 21 (3.034%)<br>• phylum: 86 (12.427%)<br>• superkingdom: 38 (5.491%)<br>• **root: 434 (62.716%)**	• Anguilla anguilla [taxid 7936]: 6 (0.867%)<br>• Malaclemys terrapin [taxid 8485]: 2 (0.289%)<br>• Apolygus lucorum [taxid 248454]: 2 (0.289%)<br>• Chlamydia abortus [taxid 83555]: 2 (0.289%)<br>• Dermochelys coriacea [taxid 27794]: 1 (0.144%)<br>• Paradactylodon mustersi [taxid 324332]: 1 (0.144%)<br>• Liolaemus pseudolemniscatus [taxid 478549]: 1 (0.144%)<br>• other: 9 (1.3%)
Benchmark OTU ID: `JH584390-_Eukaryotes`<br>OTU taxon: Chrysemys picta bellii [taxid 8478]<br>Expected: Chrysemys picta [taxid 8479] (species)<br>Number of reads: 44297<br>Number of identified reads: 36748 (82.958%)	• species: 0 (0.0%)<br>• genus: 0 (0.0%)<br>• family: 888 (2.004%)<br>• order: 2225 (5.022%)<br>• phylum: 1542 (3.481%)<br>• superkingdom: 496 (1.119%)<br>• **root: 30751 (69.42%)**	• **Terrapene carolina [taxid 158814]: 888 (2.004%)**<br>• Chelonia mydas [taxid 8469]: 375 (0.846%)<br>• Gopherus agassizii [taxid 38772]: 342 (0.772%)<br>• Pelodiscus sinensis [taxid 13735]: 264 (0.595%)<br>• Platysternon megacephalum [taxid 55544]: 119 (0.268%)<br>• Limosa lapponica [taxid 161683]: 47 (0.106%)<br>• Hirundo rustica [taxid 43150]: 9 (0.02%)<br>• other: 241 (0.544%)
Benchmark OTU ID: `JH584391-_Eukaryotes`<br>OTU taxon: Chrysemys picta bellii [taxid 8478]<br>Expected: Chrysemys picta [taxid 8479] (species)<br>Number of reads: 4766<br>Number of identified reads: 4311 (90.453%)	• species: 0 (0.0%)<br>• genus: 0 (0.0%)<br>• family: 15 (0.314%)<br>• order: 65 (1.363%)<br>• phylum: 42 (0.881%)<br>• superkingdom: 34 (0.713%)<br>• **root: 4089 (85.795%)**	• Chelonia mydas [taxid 8469]: 21 (0.44%)<br>• Terrapene carolina [taxid 158814]: 15 (0.314%)<br>• Gopherus agassizii [taxid 38772]: 15 (0.314%)<br>• Pelodiscus sinensis [taxid 13735]: 14 (0.293%)<br>• Limosa lapponica [taxid 161683]: 3 (0.062%)<br>• Trichomonas vaginalis [taxid 5722]: 2 (0.041%)<br>• Tetrahymena thermophila [taxid 5911]: 2 (0.041%)<br>• other: 17 (0.356%)

Operational Taxonomic Unit (OTU)	Correct identifications	Wrong or overspecific identifications at species rank
Benchmark OTU ID: `AM711867-_Actinobacteria`<br>OTU taxon: Clavibacter michiganensis subsp. michiganensis NCPPB 382 [taxid 443906]<br>Expected: Clavibacter michiganensis [taxid 28447] (species)<br>Number of reads: 13756<br>Number of identified reads: 13710 (99.665%)	• **species: 5746 (41.77%)**<br>• genus: 4117 (29.928%)<br>• family: 1079 (7.843%)<br>• order: 229 (1.664%)<br>• class: 646 (4.696%)<br>• phylum: 15 (0.109%)<br>• superkingdom: 856 (6.222%)<br>• root: 1006 (7.313%)	• Labedella populi [taxid 2498850]: 2 (0.014%)<br>• Gordonia rhizosphera [taxid 83341]: 2 (0.014%)<br>• Embleya hyalina [taxid 516124]: 1 (0.007%)<br>• Aeromicrobium choanae [taxid 1736691]: 1 (0.007%)<br>• Blautia wexlerae [taxid 418240]: 1 (0.007%)<br>• Microbacterium foliorum [taxid 104336]: 1 (0.007%)<br>• Oerskovia enterophila [taxid 43678]: 1 (0.007%)<br>• Rathayibacter rathayi [taxid 33887]: 1 (0.007%)<br>• other: 37 (0.268%)
Benchmark OTU ID: `AM849034-_Actinobacteria`<br>OTU taxon: Clavibacter michiganensis subsp. sepedonicus [taxid 31964]<br>Expected: Clavibacter michiganensis [taxid 28447] (species)<br>Number of reads: 13564<br>Number of identified reads: 13496 (99.498%)	• **species: 5546 (40.887%)**<br>• genus: 3971 (29.276%)<br>• family: 1122 (8.271%)<br>• order: 226 (1.666%)<br>• class: 735 (5.418%)<br>• phylum: 6 (0.044%)<br>• superkingdom: 790 (5.824%)<br>• root: 1090 (8.035%)	• Pseudoclavibacter endophyticus [taxid 1778590]: 3 (0.022%)<br>• Klugiella xanthotipulae [taxid 244735]: 3 (0.022%)<br>• Protaetiibacter intestinalis [taxid 2419774]: 2 (0.014%)<br>• Cnuibacter physcomitrellae [taxid 1619308]: 1 (0.007%)<br>• Agromyces albus [taxid 205332]: 1 (0.007%)<br>• Kocuria sp. UCD-OTCP [taxid 1292021]: 1 (0.007%)<br>• Mycolicibacterium aromaticivorans [taxid 318425]: 1 (0.007%)<br>• Leifsonia xyli [taxid 1575]: 1 (0.007%)<br>• other: 32 (0.235%)
Benchmark OTU ID: `FN665652-_Firmicutes`<br>OTU taxon: Clostridioides difficile CF5 [taxid 699036]<br>Expected: Clostridioides difficile [taxid 1496] (species)<br>Number of reads: 5886<br>Number of identified reads: 5718 (97.145%)	• **species: 4151 (70.523%)**<br>• genus: 0 (0.0%)<br>• family: 201 (3.414%)<br>• order: 239 (4.06%)<br>• class: 6 (0.101%)<br>• phylum: 188 (3.194%)<br>• superkingdom: 271 (4.604%)<br>• root: 653 (11.094%)	• Peptacetobacter hiranonis [taxid 89152]: 3 (0.05%)<br>• Clostridium paraputrificum [taxid 29363]: 2 (0.033%)<br>• Lactobacillus equicursoris [taxid 420645]: 1 (0.016%)<br>• Alkalihalobacillus wakoensis [taxid 127891]: 1 (0.016%)<br>• Caldanaerovirga acetigignens [taxid 447595]: 1 (0.016%)<br>• Enterococcus thailandicus [taxid 417368]: 1 (0.016%)<br>• Romboutsia maritimum [taxid 2020948]: 1 (0.016%)<br>• Clostridium saccharoperbutylacetonicum [taxid 36745]: 1 (0.016%)<br>• other: 13 (0.22%)
Benchmark OTU ID: `CP000721-_Firmicutes`<br>OTU taxon: Clostridium beijerinckii NCIMB 8052 [taxid 290402]<br>Expected: Clostridium beijerinckii [taxid 1520] (species)<br>Number of reads: 8832<br>Number of identified reads: 8546 (96.761%)	• species: 2263 (25.622%)<br>• **genus: 4406 (49.886%)**<br>• family: 16 (0.181%)<br>• order: 162 (1.834%)<br>• class: 17 (0.192%)<br>• phylum: 211 (2.389%)<br>• superkingdom: 352 (3.985%)<br>• root: 1103 (12.488%)	• Clostridium chromiireducens [taxid 225345]: 11 (0.124%)<br>• Clostridium saccharoperbutylacetonicum [taxid 36745]: 9 (0.101%)<br>• Clostridium sp. DL-VIII [taxid 641107]: 8 (0.09%)<br>• Clostridium puniceum [taxid 29367]: 6 (0.067%)<br>• Toxocara canis [taxid 6265]: 5 (0.056%)<br>• Clostridium uliginosum [taxid 119641]: 5 (0.056%)<br>• Clostridium butyricum [taxid 1492]: 4 (0.045%)<br>• Clostridium diolis [taxid 223919]: 4 (0.045%)<br>• other: 44 (0.498%)

Operational Taxonomic Unit (OTU)	Correct identifications	Wrong or overspecific identifications at species rank
Benchmark OTU ID: `CP002745-_Proteobacteria`<br>OTU taxon: Collimonas fungivorans Ter331 [taxid 1005048]<br>Expected: Collimonas fungivorans [taxid 158899] (species)<br>Number of reads: 10785<br>Number of identified reads: 10772 (99.879%)	• **species: 3794 (35.178%)**<br>• genus: 2986 (27.686%)<br>• family: 809 (7.501%)<br>• order: 689 (6.388%)<br>• class: 173 (1.604%)<br>• phylum: 754 (6.991%)<br>• superkingdom: 467 (4.33%)<br>• root: 1087 (10.078%)	• Collimonas arenae [taxid 279058]: 27 (0.25%)<br>• Collimonas pratensis [taxid 279113]: 21 (0.194%)<br>• Salmonella enterica [taxid 28901]: 2 (0.018%)<br>• Lupinus albus [taxid 3870]: 2 (0.018%)<br>• Sandarakinorhabdus cyanobacteriorum [taxid 1981098]: 1 (0.009%)<br>• Aggregatibacter segnis [taxid 739]: 1 (0.009%)<br>• Polaromonas vacuolata [taxid 37448]: 1 (0.009%)<br>• Cupriavidus necator [taxid 106590]: 1 (0.009%)<br>• other: 30 (0.278%)
Benchmark OTU ID: `CP000083-_Proteobacteria`<br>OTU taxon: Colwellia psychrerythraea 34H [taxid 167879]<br>Expected: Colwellia psychrerythraea [taxid 28229] (species)<br>Number of reads: 11205<br>Number of identified reads: 11063 (98.732%)	• species: 2021 (18.036%)<br>• **genus: 5948 (53.083%)**<br>• family: 323 (2.882%)<br>• order: 304 (2.713%)<br>• class: 735 (6.559%)<br>• phylum: 234 (2.088%)<br>• superkingdom: 314 (2.802%)<br>• root: 1175 (10.486%)	• Colwellia demingiae [taxid 89401]: 23 (0.205%)<br>• Colwellia ponticola [taxid 2304625]: 15 (0.133%)<br>• Colwellia chukchiensis [taxid 641665]: 4 (0.035%)<br>• Thalassotalea euphylliae [taxid 1655234]: 3 (0.026%)<br>• Pararhodospirillum photometricum [taxid 1084]: 2 (0.017%)<br>• Celerinatantimonas diazotrophica [taxid 412034]: 2 (0.017%)<br>• Paraglaciecola psychrophila [taxid 326544]: 2 (0.017%)<br>• Thioalbus denitrificans [taxid 547122]: 1 (0.008%)<br>• other: 30 (0.267%)
Benchmark OTU ID: `CP001220-_Proteobacteria`<br>OTU taxon: Comamonas thiooxydans [taxid 363952]<br>Expected: Comamonas thiooxydans [taxid 363952] (species)<br>Number of reads: 11207<br>Number of identified reads: 11173 (99.696%)	• species: 50 (0.446%)<br>• **genus: 6852 (61.14%)**<br>• family: 974 (8.69%)<br>• order: 876 (7.816%)<br>• class: 116 (1.035%)<br>• phylum: 721 (6.433%)<br>• superkingdom: 414 (3.694%)<br>• root: 1153 (10.288%)	• **Comamonas testosteroni [taxid 285]: 1095 (9.77%)**<br>• Comamonas aquatica [taxid 225991]: 5 (0.044%)<br>• Comamonas terrigena [taxid 32013]: 4 (0.035%)<br>• Comamonas kerstersii [taxid 225992]: 4 (0.035%)<br>• Curvibacter putative symbiont of Hydra magnipapillata [taxid 667019]: 3 (0.026%)<br>• Rhodoferax sediminis [taxid 2509614]: 2 (0.017%)<br>• Simplicispira metamorpha [taxid 80881]: 2 (0.017%)<br>• Comamonas sp. JNW [taxid 2170731]: 2 (0.017%)<br>• other: 42 (0.374%)
Benchmark OTU ID: `CP001854-_Actinobacteria`<br>OTU taxon: Conexibacter woesei DSM 14684 [taxid 469383]<br>Expected: Conexibacter woesei [taxid 191495] (species)<br>Number of reads: 28758<br>Number of identified reads: 28707 (99.822%)	• **species: 20317 (70.648%)**<br>• genus: 6 (0.02%)<br>• family: 0 (0.0%)<br>• order: 100 (0.347%)<br>• class: 1 (0.003%)<br>• phylum: 2845 (9.892%)<br>• superkingdom: 2905 (10.101%)<br>• root: 2501 (8.696%)	• Gambierdiscus australes [taxid 439317]: 2 (0.006%)<br>• Syntrophomonas wolfei [taxid 863]: 1 (0.003%)<br>• Micromonospora deserti [taxid 2070366]: 1 (0.003%)<br>• Oricola cellulosilytica [taxid 1429082]: 1 (0.003%)<br>• Actinokineospora auranticolor [taxid 155976]: 1 (0.003%)<br>• Micropruina glycogenica [taxid 75385]: 1 (0.003%)<br>• Haloactinopolyspora alba [taxid 648780]: 1 (0.003%)<br>• Chloracidobacterium thermophilum [taxid 458033]: 1 (0.003%)<br>• other: 55 (0.191%)

Operational Taxonomic Unit (OTU)	Correct identifications	Wrong or overspecific identifications at species rank
Benchmark OTU ID: `FP929039-_Firmicutes`<br>OTU taxon: Coprococcus sp. ART55/1 [taxid 751585]<br>Expected: Coprococcus [taxid 33042] (genus)<br>Number of reads: 3591<br>Number of identified reads: 3575 (99.554%)	• **genus: 2308 (64.271%)**<br>• family: 76 (2.116%)<br>• order: 405 (11.278%)<br>• class: 2 (0.055%)<br>• phylum: 92 (2.561%)<br>• superkingdom: 152 (4.232%)<br>• root: 540 (15.037%)	• Coprococcus eutactus [taxid 33043]: 62 (1.726%)<br>• Roseburia inulinivorans [taxid 360807]: 2 (0.055%)<br>• Eubacterium ventriosum [taxid 39496]: 1 (0.027%)<br>• Anaerobacterium chartisolvens [taxid 1297424]: 1 (0.027%)<br>• Blautia coccoides [taxid 1532]: 1 (0.027%)<br>• Mediterraneibacter butyricigenes [taxid 2316025]: 1 (0.027%)<br>• Paenibacillus thiaminolyticus [taxid 49283]: 1 (0.027%)<br>• other: 13 (0.362%)
Benchmark OTU ID: `CP001145-_Firmicutes`<br>OTU taxon: Coprothermobacter proteolyticus DSM 5265 [taxid 309798]<br>Expected: Coprothermobacter proteolyticus [taxid 35786] (species)<br>Number of reads: 1511<br>Number of identified reads: 1504 (99.536%)	• **species: 854 (56.518%)**<br>• genus: 312 (20.648%)<br>• family: 0 (0.0%)<br>• order: 0 (0.0%)<br>• class: 0 (0.0%)<br>• phylum: 0 (0.0%)<br>• superkingdom: 150 (9.927%)<br>• root: 185 (12.243%)	• Sinobacterium caligoides [taxid 933926]: 1 (0.066%)<br>• Lupinus albus [taxid 3870]: 1 (0.066%)<br>• Trichuris trichiura [taxid 36087]: 1 (0.066%)<br>• Pelomonas puraquae [taxid 431059]: 1 (0.066%)
Benchmark OTU ID: `CP001998-_Verrucomicrobia`<br>OTU taxon: Coraliomargarita akajimensis DSM 45221 [taxid 583355]<br>Expected: Coraliomargarita akajimensis [taxid 395922] (species)<br>Number of reads: 259467<br>Number of identified reads: 258953 (99.801%)	• **species: 194087 (74.802%)**<br>• genus: 660 (0.254%)<br>• family: 1971 (0.759%)<br>• order: 25 (0.009%)<br>• class: 4719 (1.818%)<br>• phylum: 5495 (2.117%)<br>• superkingdom: 22087 (8.512%)<br>• root: 29668 (11.434%)	• Coraliomargarita sinensis [taxid 2174842]: 27 (0.01%)<br>• Salmonella enterica [taxid 28901]: 7 (0.002%)<br>• Lupinus albus [taxid 3870]: 6 (0.002%)<br>• Rariglobus hedericola [taxid 2597822]: 5 (0.001%)<br>• Helicobacter pylori [taxid 210]: 5 (0.001%)<br>• Sphingobacterium faecium [taxid 34087]: 4 (0.001%)<br>• Xenopus tropicalis [taxid 8364]: 4 (0.001%)<br>• Candidatus Moanabacter tarae [taxid 2200854]: 4 (0.001%)<br>• other: 392 (0.151%)
Benchmark OTU ID: `CP003389-_Proteobacteria`<br>OTU taxon: Corallococcus coralloides DSM 2259 [taxid 1144275]<br>Expected: Corallococcus coralloides [taxid 184914] (species)<br>Number of reads: 21797<br>Number of identified reads: 21663 (99.385%)	• species: 4449 (20.411%)<br>• **genus: 11077 (50.818%)**<br>• family: 1973 (9.051%)<br>• order: 719 (3.298%)<br>• class: 68 (0.311%)<br>• phylum: 530 (2.431%)<br>• superkingdom: 1147 (5.262%)<br>• root: 1684 (7.725%)	• Corallococcus carmarthensis [taxid 2316728]: 21 (0.096%)<br>• Corallococcus interemptor [taxid 2316720]: 19 (0.087%)<br>• Corallococcus exercitus [taxid 2316736]: 15 (0.068%)<br>• Corallococcus aberystwythensis [taxid 2316722]: 12 (0.055%)<br>• Corallococcus sicarius [taxid 2316726]: 8 (0.036%)<br>• Corallococcus terminator [taxid 2316733]: 7 (0.032%)<br>• Corallococcus exiguus [taxid 83462]: 5 (0.022%)<br>• Corallococcus llansteffanensis [taxid 2316731]: 5 (0.022%)<br>• other: 53 (0.243%)

Operational Taxonomic Unit (OTU)	Correct identifications	Wrong or overspecific identifications at species rank
Benchmark OTU ID: `CP002830-_Proteobacteria`<br>OTU taxon: Corallococcus macrosporus [taxid 35]<br>Expected: Corallococcus macrosporus [taxid 35] (species)<br>Number of reads: 19374<br>Number of identified reads: 19247 (99.344%)	• species: 226 (1.166%)<br>• genus: 65 (0.335%)<br>• **family: 14803 (76.406%)**<br>• order: 764 (3.943%)<br>• class: 48 (0.247%)<br>• phylum: 520 (2.684%)<br>• superkingdom: 1123 (5.796%)<br>• root: 1678 (8.661%)	• **Myxococcus fulvus [taxid 33]: 2935 (15.149%)**<br>• Myxococcus xanthus [taxid 34]: 71 (0.366%)<br>• Myxococcus hansupus [taxid 1297742]: 44 (0.227%)<br>• Myxococcus virescens [taxid 83456]: 27 (0.139%)<br>• Myxococcus llanfairpwllgwyngyllgogerychwyrndrobwllllantysiliogogogochensis [taxid 2590453]: 14 (0.072%)<br>• Myxococcus stipitatus [taxid 83455]: 11 (0.056%)<br>• Stigmatella aurantiaca [taxid 41]: 4 (0.02%)<br>• Corallococcus coralloides [taxid 184914]: 2 (0.01%)<br>• other: 49 (0.252%)
Benchmark OTU ID: `CP001601-_Actinobacteria`<br>OTU taxon: Corynebacterium aurimucosum ATCC 700975 [taxid 548476]<br>Expected: Corynebacterium aurimucosum [taxid 169292] (species)<br>Number of reads: 11268<br>Number of identified reads: 11183 (99.245%)	• species: 2199 (19.515%)<br>• **genus: 6801 (60.356%)**<br>• family: 0 (0.0%)<br>• order: 102 (0.905%)<br>• class: 357 (3.168%)<br>• phylum: 9 (0.079%)<br>• superkingdom: 501 (4.446%)<br>• root: 1201 (10.658%)	• Corynebacterium minutissimum [taxid 38301]: 16 (0.141%)<br>• Corynebacterium flavescens [taxid 28028]: 7 (0.062%)<br>• Corynebacterium singulare [taxid 161899]: 7 (0.062%)<br>• Corynebacterium pseudogenitalium [taxid 38303]: 7 (0.062%)<br>• Corynebacterium simulans [taxid 146827]: 5 (0.044%)<br>• Corynebacterium striatum [taxid 43770]: 5 (0.044%)<br>• Corynebacterium accolens [taxid 38284]: 4 (0.035%)<br>• Corynebacterium tapiri [taxid 1448266]: 4 (0.035%)<br>• other: 93 (0.825%)
Benchmark OTU ID: `CP003211-_Actinobacteria`<br>OTU taxon: Corynebacterium diphtheriae CDCE 8392 [taxid 698965]<br>Expected: Corynebacterium diphtheriae [taxid 1717] (species)<br>Number of reads: 9520<br>Number of identified reads: 9481 (99.59%)	• species: 2183 (22.93%)<br>• **genus: 4956 (52.058%)**<br>• family: 0 (0.0%)<br>• order: 132 (1.386%)<br>• class: 367 (3.855%)<br>• phylum: 7 (0.073%)<br>• superkingdom: 481 (5.052%)<br>• root: 1345 (14.128%)	• Corynebacterium belfantii [taxid 2014537]: 51 (0.535%)<br>• Corynebacterium rouxii [taxid 2719119]: 34 (0.357%)<br>• Corynebacterium urealyticum [taxid 43771]: 8 (0.084%)<br>• Corynebacterium kutscheri [taxid 35755]: 5 (0.052%)<br>• Corynebacterium argentoratense [taxid 42817]: 5 (0.052%)<br>• Corynebacterium ulcerans [taxid 65058]: 4 (0.042%)<br>• Corynebacterium pseudotuberculosis [taxid 1719]: 4 (0.042%)<br>• Corynebacterium mustelae [taxid 571915]: 4 (0.042%)<br>• other: 78 (0.819%)
Benchmark OTU ID: `CP003213-_Actinobacteria`<br>OTU taxon: Corynebacterium diphtheriae HC02 [taxid 698968]<br>Expected: Corynebacterium diphtheriae [taxid 1717] (species)<br>Number of reads: 9693<br>Number of identified reads: 9653 (99.587%)	• species: 2131 (21.984%)<br>• **genus: 5031 (51.903%)**<br>• family: 0 (0.0%)<br>• order: 129 (1.33%)<br>• class: 576 (5.942%)<br>• phylum: 10 (0.103%)<br>• superkingdom: 414 (4.271%)<br>• root: 1345 (13.875%)	• Corynebacterium rouxii [taxid 2719119]: 63 (0.649%)<br>• Corynebacterium belfantii [taxid 2014537]: 40 (0.412%)<br>• Corynebacterium atypicum [taxid 191610]: 13 (0.134%)<br>• Corynebacterium pseudogenitalium [taxid 38303]: 9 (0.092%)<br>• Gleimia hominis [taxid 595468]: 7 (0.072%)<br>• Corynebacterium accolens [taxid 38284]: 6 (0.061%)<br>• Corynebacterium ulcerans [taxid 65058]: 5 (0.051%)<br>• Corynebacterium pseudotuberculosis [taxid 1719]: 4 (0.041%)<br>• other: 79 (0.815%)

Operational Taxonomic Unit (OTU)	Correct identifications	Wrong or overspecific identifications at species rank
Benchmark OTU ID: `CP003214-_Actinobacteria`<br>OTU taxon: Corynebacterium diphtheriae HC03 [taxid 698969]<br>Expected: Corynebacterium diphtheriae [taxid 1717] (species)<br>Number of reads: 9741<br>Number of identified reads: 9706 (99.64%)	• species: 2191 (22.492%)<br>• **genus: 5028 (51.616%)**<br>• family: 0 (0.0%)<br>• order: 134 (1.375%)<br>• class: 415 (4.26%)<br>• phylum: 3 (0.03%)<br>• superkingdom: 461 (4.732%)<br>• root: 1462 (15.008%)	• Corynebacterium belfantii [taxid 2014537]: 65 (0.667%)<br>• Corynebacterium rouxii [taxid 2719119]: 49 (0.503%)<br>• Corynebacterium accolens [taxid 38284]: 6 (0.061%)<br>• Corynebacterium glutamicum [taxid 1718]: 6 (0.061%)<br>• Corynebacterium pseudotuberculosis [taxid 1719]: 5 (0.051%)<br>• Corynebacterium striatum [taxid 43770]: 4 (0.041%)<br>• Corynebacterium endometrii [taxid 2488819]: 3 (0.03%)<br>• Corynebacterium tuscaniense [taxid 302449]: 3 (0.03%)<br>• other: 74 (0.759%)
Benchmark OTU ID: `CP003215-_Actinobacteria`<br>OTU taxon: Corynebacterium diphtheriae HC04 [taxid 698970]<br>Expected: Corynebacterium diphtheriae [taxid 1717] (species)<br>Number of reads: 9770<br>Number of identified reads: 9731 (99.6%)	• species: 2307 (23.613%)<br>• **genus: 4997 (51.146%)**<br>• family: 0 (0.0%)<br>• order: 158 (1.617%)<br>• class: 441 (4.513%)<br>• phylum: 10 (0.102%)<br>• superkingdom: 457 (4.677%)<br>• root: 1351 (13.828%)	• Corynebacterium rouxii [taxid 2719119]: 53 (0.542%)<br>• Corynebacterium belfantii [taxid 2014537]: 51 (0.522%)<br>• Corynebacterium ureicelerivorans [taxid 401472]: 8 (0.081%)<br>• Corynebacterium ulcerans [taxid 65058]: 7 (0.071%)<br>• Corynebacterium pseudotuberculosis [taxid 1719]: 7 (0.071%)<br>• Corynebacterium glutamicum [taxid 1718]: 4 (0.04%)<br>• Corynebacterium accolens [taxid 38284]: 3 (0.03%)<br>• Corynebacterium camporealensis [taxid 161896]: 3 (0.03%)<br>• other: 90 (0.921%)
Benchmark OTU ID: `BA000035-_Actinobacteria`<br>OTU taxon: Corynebacterium efficiens YS-314 [taxid 196164]<br>Expected: Corynebacterium efficiens [taxid 152794] (species)<br>Number of reads: 13017<br>Number of identified reads: 12962 (99.577%)	• **species: 8425 (64.723%)**<br>• genus: 1714 (13.167%)<br>• family: 0 (0.0%)<br>• order: 187 (1.436%)<br>• class: 659 (5.062%)<br>• phylum: 8 (0.061%)<br>• superkingdom: 655 (5.031%)<br>• root: 1303 (10.009%)	• Corynebacterium deserti [taxid 1408191]: 8 (0.061%)<br>• Corynebacterium rouxii [taxid 2719119]: 6 (0.046%)<br>• Corynebacterium suranareeae [taxid 2506452]: 4 (0.03%)<br>• Corynebacterium callunae [taxid 1721]: 3 (0.023%)<br>• Corynebacterium renale [taxid 1724]: 3 (0.023%)<br>• Corynebacterium glutamicum [taxid 1718]: 3 (0.023%)<br>• Corynebacterium otitidis [taxid 29321]: 3 (0.023%)<br>• Mycobacterium tuberculosis [taxid 1773]: 2 (0.015%)<br>• other: 56 (0.43%)
Benchmark OTU ID: `HE802067-_Actinobacteria`<br>OTU taxon: Corynebacterium glutamicum K051 [taxid 1204414]<br>Expected: Corynebacterium glutamicum [taxid 1718] (species)<br>Number of reads: 13813<br>Number of identified reads: 13766 (99.659%)	• species: 2212 (16.013%)<br>• **genus: 8339 (60.37%)**<br>• family: 0 (0.0%)<br>• order: 148 (1.071%)<br>• class: 514 (3.721%)<br>• phylum: 4 (0.028%)<br>• superkingdom: 619 (4.481%)<br>• root: 1912 (13.842%)	• Corynebacterium suranareeae [taxid 2506452]: 41 (0.296%)<br>• Corynebacterium crudilactis [taxid 1652495]: 28 (0.202%)<br>• Corynebacterium deserti [taxid 1408191]: 23 (0.166%)<br>• Corynebacterium efficiens [taxid 152794]: 12 (0.086%)<br>• Corynebacterium callunae [taxid 1721]: 10 (0.072%)<br>• Corynebacterium crenatum [taxid 168810]: 6 (0.043%)<br>• Corynebacterium rouxii [taxid 2719119]: 6 (0.043%)<br>• Corynebacterium otitidis [taxid 29321]: 3 (0.021%)<br>• other: 90 (0.651%)

Operational Taxonomic Unit (OTU)	Correct identifications	Wrong or overspecific identifications at species rank
Benchmark OTU ID: `AP009044-_Actinobacteria`<br>OTU taxon: Corynebacterium glutamicum R [taxid 340322]<br>Expected: Corynebacterium glutamicum [taxid 1718] (species)<br>Number of reads: 13827<br>Number of identified reads: 13791 (99.739%)	• species: 1351 (9.77%)<br>• **genus: 9191 (66.471%)**<br>• family: 0 (0.0%)<br>• order: 145 (1.048%)<br>• class: 578 (4.18%)<br>• phylum: 4 (0.028%)<br>• superkingdom: 612 (4.426%)<br>• root: 1896 (13.712%)	• Corynebacterium crenatum [taxid 168810]: 38 (0.274%)<br>• Corynebacterium suranareeae [taxid 2506452]: 36 (0.26%)<br>• Corynebacterium crudilactis [taxid 1652495]: 27 (0.195%)<br>• Corynebacterium deserti [taxid 1408191]: 16 (0.115%)<br>• Corynebacterium efficiens [taxid 152794]: 16 (0.115%)<br>• Corynebacterium callunae [taxid 1721]: 10 (0.072%)<br>• Corynebacterium rouxii [taxid 2719119]: 4 (0.028%)<br>• Corynebacterium durum [taxid 61592]: 3 (0.021%)<br>• other: 79 (0.571%)
Benchmark OTU ID: `CP003697-_Actinobacteria`<br>OTU taxon: Corynebacterium halotolerans YIM 70093 = DSM 44683 [taxid 1121362]<br>Expected: Corynebacterium halotolerans [taxid 225326] (species)<br>Number of reads: 12962<br>Number of identified reads: 12886 (99.413%)	• **species: 8375 (64.611%)**<br>• genus: 1572 (12.127%)<br>• family: 0 (0.0%)<br>• order: 201 (1.55%)<br>• class: 780 (6.017%)<br>• phylum: 10 (0.077%)<br>• superkingdom: 779 (6.009%)<br>• root: 1154 (8.902%)	• Corynebacterium efficiens [taxid 152794]: 5 (0.038%)<br>• Corynebacterium pilosum [taxid 35756]: 4 (0.03%)<br>• Corynebacterium testudinoris [taxid 136857]: 3 (0.023%)<br>• Corynebacterium rouxii [taxid 2719119]: 3 (0.023%)<br>• Corynebacterium maris [taxid 575200]: 3 (0.023%)<br>• Corynebacterium frankenforstense [taxid 1230998]: 2 (0.015%)<br>• Corynebacterium canis [taxid 679663]: 2 (0.015%)<br>• Arthrobacter crusticola [taxid 2547960]: 1 (0.007%)<br>• other: 47 (0.362%)
Benchmark OTU ID: `CP001620-_Actinobacteria`<br>OTU taxon: Corynebacterium kroppenstedtii DSM 44385 [taxid 645127]<br>Expected: Corynebacterium kroppenstedtii [taxid 161879] (species)<br>Number of reads: 9586<br>Number of identified reads: 9533 (99.447%)	• **species: 6507 (67.88%)**<br>• genus: 342 (3.567%)<br>• family: 0 (0.0%)<br>• order: 140 (1.46%)<br>• class: 389 (4.058%)<br>• phylum: 3 (0.031%)<br>• superkingdom: 489 (5.101%)<br>• root: 1650 (17.212%)	• Corynebacterium rouxii [taxid 2719119]: 2 (0.02%)<br>• Plesiocystis pacifica [taxid 191768]: 1 (0.01%)<br>• Streptomyces bohaiensis [taxid 1431344]: 1 (0.01%)<br>• Sorangium cellulosum [taxid 56]: 1 (0.01%)<br>• Corynebacterium lactis [taxid 1231000]: 1 (0.01%)<br>• Pseudo-nitzschia pungens [taxid 37318]: 1 (0.01%)<br>• Streptomyces alkaliterrae [taxid 2213162]: 1 (0.01%)<br>• Corynebacterium accolens [taxid 38284]: 1 (0.01%)<br>• other: 33 (0.344%)
Benchmark OTU ID: `CP003152-_Actinobacteria`<br>OTU taxon: Corynebacterium pseudotuberculosis 3/99-5 [taxid 1087452]<br>Expected: Corynebacterium pseudotuberculosis [taxid 1719] (species)<br>Number of reads: 9053<br>Number of identified reads: 9002 (99.436%)	• **species: 4060 (44.847%)**<br>• genus: 2770 (30.597%)<br>• family: 0 (0.0%)<br>• order: 106 (1.17%)<br>• class: 356 (3.932%)<br>• phylum: 4 (0.044%)<br>• superkingdom: 394 (4.352%)<br>• root: 1305 (14.415%)	• Corynebacterium ulcerans [taxid 65058]: 29 (0.32%)<br>• Corynebacterium pseudodiphtheriticum [taxid 37637]: 6 (0.066%)<br>• Corynebacterium rouxii [taxid 2719119]: 5 (0.055%)<br>• Corynebacterium efficiens [taxid 152794]: 5 (0.055%)<br>• Corynebacterium resistens [taxid 258224]: 3 (0.033%)<br>• Corynebacterium striatum [taxid 43770]: 2 (0.022%)<br>• Corynebacterium diphtheriae [taxid 1717]: 2 (0.022%)<br>• Corynebacterium vitaeruminis [taxid 38305]: 2 (0.022%)<br>• other: 40 (0.441%)

Operational Taxonomic Unit (OTU)	Correct identifications	Wrong or overspecific identifications at species rank
Benchmark OTU ID: `CP003421-_Actinobacteria`<br>OTU taxon: Corynebacterium pseudotuberculosis 31 [taxid 1087451]<br>Expected: Corynebacterium pseudotuberculosis [taxid 1719] (species)<br>Number of reads: 8852<br>Number of identified reads: 8813 (99.559%)	• **species: 3894 (43.99%)**<br>• genus: 2776 (31.36%)<br>• family: 0 (0.0%)<br>• order: 121 (1.366%)<br>• class: 342 (3.863%)<br>• phylum: 7 (0.079%)<br>• superkingdom: 368 (4.157%)<br>• root: 1291 (14.584%)	• Corynebacterium ulcerans [taxid 65058]: 30 (0.338%)<br>• Corynebacterium phage LGCM-VI [taxid 1965283]: 6 (0.067%)<br>• Corynebacterium kroppenstedtii [taxid 161879]: 4 (0.045%)<br>• Corynebacterium kutscheri [taxid 35755]: 3 (0.033%)<br>• Corynebacterium efficiens [taxid 152794]: 3 (0.033%)<br>• Corynebacterium mustelae [taxid 571915]: 3 (0.033%)<br>• Corynebacterium durum [taxid 61592]: 3 (0.033%)<br>• Corynebacterium vitaeruminis [taxid 38305]: 3 (0.033%)<br>• other: 44 (0.497%)
Benchmark OTU ID: `CP001829-_Actinobacteria`<br>OTU taxon: Corynebacterium pseudotuberculosis C231 [taxid 681645]<br>Expected: Corynebacterium pseudotuberculosis [taxid 1719] (species)<br>Number of reads: 9005<br>Number of identified reads: 8955 (99.444%)	• **species: 4111 (45.652%)**<br>• genus: 2701 (29.994%)<br>• family: 0 (0.0%)<br>• order: 126 (1.399%)<br>• class: 326 (3.62%)<br>• phylum: 2 (0.022%)<br>• superkingdom: 426 (4.73%)<br>• root: 1260 (13.992%)	• Corynebacterium ulcerans [taxid 65058]: 34 (0.377%)<br>• Corynebacterium rouxii [taxid 2719119]: 7 (0.077%)<br>• Corynebacterium resistens [taxid 258224]: 4 (0.044%)<br>• Corynebacterium pseudodiphtheriticum [taxid 37637]: 4 (0.044%)<br>• Corynebacterium durum [taxid 61592]: 3 (0.033%)<br>• Corynebacterium efficiens [taxid 152794]: 3 (0.033%)<br>• Corynebacterium pseudogenitalium [taxid 38303]: 3 (0.033%)<br>• Corynebacterium geronticis [taxid 2079234]: 2 (0.022%)<br>• other: 39 (0.433%)
Benchmark OTU ID: `CP003061-_Actinobacteria`<br>OTU taxon: Corynebacterium pseudotuberculosis CIP 52.97 [taxid 935697]<br>Expected: Corynebacterium pseudotuberculosis [taxid 1719] (species)<br>Number of reads: 8967<br>Number of identified reads: 8919 (99.464%)	• **species: 3965 (44.217%)**<br>• genus: 2735 (30.5%)<br>• family: 0 (0.0%)<br>• order: 127 (1.416%)<br>• class: 334 (3.724%)<br>• phylum: 6 (0.066%)<br>• superkingdom: 411 (4.583%)<br>• root: 1327 (14.798%)	• Corynebacterium ulcerans [taxid 65058]: 30 (0.334%)<br>• Corynebacterium rouxii [taxid 2719119]: 5 (0.055%)<br>• Corynebacterium kutscheri [taxid 35755]: 4 (0.044%)<br>• Sarcoptes scabiei [taxid 52283]: 4 (0.044%)<br>• Nocardia asteroides [taxid 1824]: 2 (0.022%)<br>• Corynebacterium renale [taxid 1724]: 2 (0.022%)<br>• Corynebacterium diphtheriae [taxid 1717]: 2 (0.022%)<br>• Corynebacterium ammoniagenes [taxid 1697]: 2 (0.022%)<br>• other: 50 (0.557%)
Benchmark OTU ID: `CP003652-_Actinobacteria`<br>OTU taxon: Corynebacterium pseudotuberculosis Cp162 [taxid 1161911]<br>Expected: Corynebacterium pseudotuberculosis [taxid 1719] (species)<br>Number of reads: 8835<br>Number of identified reads: 8790 (99.49%)	• **species: 3712 (42.014%)**<br>• genus: 2831 (32.043%)<br>• family: 0 (0.0%)<br>• order: 98 (1.109%)<br>• class: 353 (3.995%)<br>• phylum: 2 (0.022%)<br>• superkingdom: 426 (4.821%)<br>• root: 1357 (15.359%)	• Corynebacterium ulcerans [taxid 65058]: 41 (0.464%)<br>• Corynebacterium diphtheriae [taxid 1717]: 4 (0.045%)<br>• Corynebacterium choanis [taxid 1862358]: 3 (0.033%)<br>• Corynebacterium argentoratense [taxid 42817]: 3 (0.033%)<br>• Corynebacterium genitalium [taxid 38288]: 3 (0.033%)<br>• Corynebacterium testudinoris [taxid 136857]: 3 (0.033%)<br>• Tanacetum cinerariifolium [taxid 118510]: 2 (0.022%)<br>• Corynebacterium glutamicum [taxid 1718]: 2 (0.022%)<br>• other: 51 (0.577%)

Operational Taxonomic Unit (OTU)	Correct identifications	Wrong or overspecific identifications at species rank
Benchmark OTU ID: `CP002097-_Actinobacteria`<br>OTU taxon: Corynebacterium pseudotuberculosis FRC41 [taxid 765874]<br>Expected: Corynebacterium pseudotuberculosis [taxid 1719] (species)<br>Number of reads: 9053<br>Number of identified reads: 9015 (99.58%)	• **species: 4146 (45.796%)**<br>• genus: 2709 (29.923%)<br>• family: 0 (0.0%)<br>• order: 109 (1.204%)<br>• class: 375 (4.142%)<br>• phylum: 7 (0.077%)<br>• superkingdom: 411 (4.539%)<br>• root: 1253 (13.84%)	• Corynebacterium ulcerans [taxid 65058]: 27 (0.298%)<br>• Corynebacterium rouxii [taxid 2719119]: 12 (0.132%)<br>• Corynebacterium kutscheri [taxid 35755]: 4 (0.044%)<br>• Corynebacterium durum [taxid 61592]: 3 (0.033%)<br>• Corynebacterium pseudodiphtheriticum [taxid 37637]: 3 (0.033%)<br>• Mycobacterium pseudoshottsii [taxid 265949]: 2 (0.022%)<br>• Corynebacterium testudinoris [taxid 136857]: 2 (0.022%)<br>• Corynebacterium callunae [taxid 1721]: 2 (0.022%)<br>• other: 51 (0.563%)
Benchmark OTU ID: `CP002791-_Actinobacteria`<br>OTU taxon: Corynebacterium ulcerans BR-AD22 [taxid 945712]<br>Expected: Corynebacterium ulcerans [taxid 65058] (species)<br>Number of reads: 10368<br>Number of identified reads: 10307 (99.411%)	• species: 3499 (33.748%)<br>• **genus: 4070 (39.255%)**<br>• family: 0 (0.0%)<br>• order: 134 (1.292%)<br>• class: 394 (3.8%)<br>• phylum: 3 (0.028%)<br>• superkingdom: 486 (4.687%)<br>• root: 1707 (16.464%)	• Corynebacterium pseudotuberculosis [taxid 1719]: 53 (0.511%)<br>• Corynebacterium rouxii [taxid 2719119]: 26 (0.25%)<br>• Corynebacterium diphtheriae [taxid 1717]: 15 (0.144%)<br>• Kytococcus sedentarius [taxid 1276]: 5 (0.048%)<br>• Corynebacterium vitaeruminis [taxid 38305]: 4 (0.038%)<br>• Corynebacterium kutscheri [taxid 35755]: 4 (0.038%)<br>• Corynebacterium pseudogenitalium [taxid 38303]: 3 (0.028%)<br>• Corynebacterium efficiens [taxid 152794]: 3 (0.028%)<br>• other: 65 (0.626%)
Benchmark OTU ID: `AM942444-_Actinobacteria`<br>OTU taxon: Corynebacterium urealyticum DSM 7109 [taxid 504474]<br>Expected: Corynebacterium urealyticum [taxid 43771] (species)<br>Number of reads: 9206<br>Number of identified reads: 9145 (99.337%)	• species: 3135 (34.053%)<br>• **genus: 3847 (41.787%)**<br>• family: 0 (0.0%)<br>• order: 130 (1.412%)<br>• class: 550 (5.974%)<br>• phylum: 5 (0.054%)<br>• superkingdom: 536 (5.822%)<br>• root: 929 (10.091%)	• Corynebacterium kroppenstedtii [taxid 161879]: 4 (0.043%)<br>• Corynebacterium terpenotabidum [taxid 89154]: 4 (0.043%)<br>• Corynebacterium glutamicum [taxid 1718]: 3 (0.032%)<br>• Bifidobacterium choerinum [taxid 35760]: 3 (0.032%)<br>• Corynebacterium pseudogenitalium [taxid 38303]: 3 (0.032%)<br>• Corynebacterium heidelbergense [taxid 2055947]: 3 (0.032%)<br>• Corynebacterium jeikeium [taxid 38289]: 2 (0.021%)<br>• Corynebacterium kutscheri [taxid 35755]: 2 (0.021%)<br>• other: 55 (0.597%)
Benchmark OTU ID: `CP004085-_Actinobacteria`<br>OTU taxon: Corynebacterium urealyticum DSM 7111 [taxid 1267754]<br>Expected: Corynebacterium urealyticum [taxid 43771] (species)<br>Number of reads: 8945<br>Number of identified reads: 8876 (99.228%)	• species: 2652 (29.647%)<br>• **genus: 4017 (44.907%)**<br>• family: 0 (0.0%)<br>• order: 126 (1.408%)<br>• class: 518 (5.79%)<br>• phylum: 3 (0.033%)<br>• superkingdom: 575 (6.428%)<br>• root: 979 (10.944%)	• Corynebacterium jeikeium [taxid 38289]: 74 (0.827%)<br>• Corynebacterium pseudotuberculosis [taxid 1719]: 3 (0.033%)<br>• Corynebacterium glutamicum [taxid 1718]: 3 (0.033%)<br>• Corynebacterium minutissimum [taxid 38301]: 3 (0.033%)<br>• Corynebacterium heidelbergense [taxid 2055947]: 3 (0.033%)<br>• Corynebacterium renale [taxid 1724]: 3 (0.033%)<br>• Corynebacterium resistens [taxid 258224]: 3 (0.033%)<br>• Corynebacterium bovis [taxid 36808]: 3 (0.033%)<br>• other: 73 (0.816%)

Operational Taxonomic Unit (OTU)	Correct identifications	Wrong or overspecific identifications at species rank
Benchmark OTU ID: `CP002917-_Actinobacteria`<br>OTU taxon: Corynebacterium variabile DSM 44702 [taxid 858619]<br>Expected: Corynebacterium variabile [taxid 1727] (species)<br>Number of reads: 14419<br>Number of identified reads: 14356 (99.563%)	• **species: 9103 (63.131%)**<br>• genus: 1875 (13.003%)<br>• family: 0 (0.0%)<br>• order: 207 (1.435%)<br>• class: 830 (5.756%)<br>• phylum: 9 (0.062%)<br>• superkingdom: 873 (6.054%)<br>• root: 1444 (10.014%)	• Corynebacterium provencense [taxid 1737425]: 13 (0.09%)<br>• Corynebacterium nuruki [taxid 1032851]: 11 (0.076%)<br>• Corynebacterium terpenotabidum [taxid 89154]: 6 (0.041%)<br>• Corynebacterium casei [taxid 160386]: 5 (0.034%)<br>• Corynebacterium glyciniphilum [taxid 1404244]: 4 (0.027%)<br>• Corynebacterium glutamicum [taxid 1718]: 4 (0.027%)<br>• Corynebacterium efficiens [taxid 152794]: 4 (0.027%)<br>• Corynebacterium rouxii [taxid 2719119]: 4 (0.027%)<br>• other: 65 (0.45%)
Benchmark OTU ID: `CP000890-_Proteobacteria`<br>OTU taxon: Coxiella burnetii RSA 331 [taxid 360115]<br>Expected: Coxiella burnetii [taxid 777] (species)<br>Number of reads: 3653<br>Number of identified reads: 3630 (99.37%)	• **species: 2604 (71.283%)**<br>• genus: 36 (0.985%)<br>• family: 30 (0.821%)<br>• order: 8 (0.218%)<br>• class: 117 (3.202%)<br>• phylum: 96 (2.627%)<br>• superkingdom: 156 (4.27%)<br>• root: 582 (15.932%)	• Candidatus Coxiella mudrowiae [taxid 2054173]: 1 (0.027%)<br>• Candidatus Portiera aleyrodidarum [taxid 91844]: 1 (0.027%)<br>• Streptomyces harbinensis [taxid 1176198]: 1 (0.027%)<br>• Coxiella-like endosymbiont [taxid 1592897]: 1 (0.027%)<br>• Methylomicrobium kenyense [taxid 269709]: 1 (0.027%)<br>• Ideonella sakaiensis [taxid 1547922]: 1 (0.027%)<br>• bacterium [taxid 1869227]: 1 (0.027%)<br>• [Eubacterium] saphenum [taxid 51123]: 1 (0.027%)
Benchmark OTU ID: `CP003620-_Cyanobacteria`<br>OTU taxon: Crinalium epipsammum PCC 9333 [taxid 1173022]<br>Expected: Crinalium epipsammum [taxid 241425] (species)<br>Number of reads: 31222<br>Number of identified reads: 30859 (98.837%)	• **species: 20421 (65.405%)**<br>• genus: 0 (0.0%)<br>• family: 11 (0.035%)<br>• order: 469 (1.502%)<br>• phylum: 3753 (12.02%)<br>• superkingdom: 1942 (6.219%)<br>• root: 4198 (13.445%)	• Phormidesmis priestleyi [taxid 268141]: 6 (0.019%)<br>• Phormidium ambiguum [taxid 71191]: 5 (0.016%)<br>• Gloeomargarita lithophora [taxid 1188228]: 4 (0.012%)<br>• Calothrix parietina [taxid 32054]: 4 (0.012%)<br>• Cyanophora sudae [taxid 1522369]: 3 (0.009%)<br>• Merismopedia glauca [taxid 292586]: 3 (0.009%)<br>• Moorea producens [taxid 1155739]: 3 (0.009%)<br>• other: 106 (0.339%)
Benchmark OTU ID: `CP000806-_Cyanobacteria`<br>OTU taxon: Crocosphaera subtropica ATCC 51142 [taxid 43989]<br>Expected: Crocosphaera subtropica [taxid 2546360] (species)<br>Number of reads: 28706<br>Number of identified reads: 28237 (98.366%)	• **species: 17111 (59.607%)**<br>• genus: 3347 (11.659%)<br>• family: 400 (1.393%)<br>• order: 388 (1.351%)<br>• phylum: 2462 (8.576%)<br>• superkingdom: 1516 (5.281%)<br>• root: 2952 (10.283%)	• Crocosphaera chwakensis [taxid 2546361]: 54 (0.188%)<br>• Crocosphaera watsonii [taxid 263511]: 30 (0.104%)<br>• Candidatus Atelocyanobacterium thalassa [taxid 713887]: 5 (0.017%)<br>• Rippkaea orientalis [taxid 2546366]: 4 (0.013%)<br>• cyanobacterium endosymbiont of Rhopalodia gibberula [taxid 1763363]: 3 (0.01%)<br>• Moorea producens [taxid 1155739]: 2 (0.006%)<br>• Prochlorococcus marinus [taxid 1219]: 2 (0.006%)<br>• other: 68 (0.236%)

Operational Taxonomic Unit (OTU)	Correct identifications	Wrong or overspecific identifications at species rank
Benchmark OTU ID: `CP000783-_Proteobacteria`<br>OTU taxon: Cronobacter sakazakii ATCC BAA-894 [taxid 290339]<br>Expected: Cronobacter sakazakii [taxid 28141] (species)<br>Number of reads: 8945<br>Number of identified reads: 8914 (99.653%)	• species: 1528 (17.082%)<br>• **genus: 2698 (30.162%)**<br>• family: 2300 (25.712%)<br>• order: 857 (9.58%)<br>• class: 312 (3.487%)<br>• phylum: 172 (1.922%)<br>• superkingdom: 283 (3.163%)<br>• root: 763 (8.529%)	• Salmonella enterica [taxid 28901]: 107 (1.196%)<br>• Escherichia coli [taxid 562]: 51 (0.57%)<br>• Cronobacter turicensis [taxid 413502]: 50 (0.558%)<br>• Cronobacter malonaticus [taxid 413503]: 17 (0.19%)<br>• Cronobacter condimenti [taxid 1163710]: 14 (0.156%)<br>• Cronobacter dublinensis [taxid 413497]: 10 (0.111%)<br>• Klebsiella pneumoniae [taxid 573]: 8 (0.089%)<br>• Cronobacter muytjensii [taxid 413501]: 7 (0.078%)<br>• other: 93 (1.039%)
Benchmark OTU ID: `CP003312-_Proteobacteria`<br>OTU taxon: Cronobacter sakazakii ES15 [taxid 1138308]<br>Expected: Cronobacter sakazakii [taxid 28141] (species)<br>Number of reads: 8720<br>Number of identified reads: 8696 (99.724%)	• species: 1196 (13.715%)<br>• **genus: 2783 (31.915%)**<br>• family: 2237 (25.653%)<br>• order: 913 (10.47%)<br>• class: 280 (3.211%)<br>• phylum: 197 (2.259%)<br>• superkingdom: 250 (2.866%)<br>• root: 816 (9.357%)	• Salmonella enterica [taxid 28901]: 101 (1.158%)<br>• Escherichia coli [taxid 562]: 55 (0.63%)<br>• Cronobacter turicensis [taxid 413502]: 41 (0.47%)<br>• Cronobacter malonaticus [taxid 413503]: 31 (0.355%)<br>• Cronobacter dublinensis [taxid 413497]: 15 (0.172%)<br>• Cronobacter condimenti [taxid 1163710]: 13 (0.149%)<br>• Cronobacter universalis [taxid 535744]: 10 (0.114%)<br>• Cronobacter muytjensii [taxid 413501]: 9 (0.103%)<br>• other: 87 (0.997%)
Benchmark OTU ID: `CP004091-_Proteobacteria`<br>OTU taxon: Cronobacter sakazakii SP291 [taxid 956149]<br>Expected: Cronobacter sakazakii [taxid 28141] (species)<br>Number of reads: 8890<br>Number of identified reads: 8868 (99.752%)	• species: 1402 (15.77%)<br>• **genus: 2747 (30.899%)**<br>• family: 2296 (25.826%)<br>• order: 913 (10.269%)<br>• class: 298 (3.352%)<br>• phylum: 168 (1.889%)<br>• superkingdom: 253 (2.845%)<br>• root: 788 (8.863%)	• Salmonella enterica [taxid 28901]: 92 (1.034%)<br>• Escherichia coli [taxid 562]: 58 (0.652%)<br>• Cronobacter turicensis [taxid 413502]: 38 (0.427%)<br>• Cronobacter dublinensis [taxid 413497]: 18 (0.202%)<br>• Klebsiella pneumoniae [taxid 573]: 13 (0.146%)<br>• Cronobacter malonaticus [taxid 413503]: 9 (0.101%)<br>• Cronobacter condimenti [taxid 1163710]: 8 (0.089%)<br>• Enterobacter hormaechei [taxid 158836]: 4 (0.044%)<br>• other: 103 (1.158%)
Benchmark OTU ID: `CU633749-_Proteobacteria`<br>OTU taxon: Cupriavidus taiwanensis LMG 19424 [taxid 977880]<br>Expected: Cupriavidus taiwanensis [taxid 164546] (species)<br>Number of reads: 6804<br>Number of identified reads: 6788 (99.764%)	• species: 1284 (18.871%)<br>• **genus: 3399 (49.955%)**<br>• family: 543 (7.98%)<br>• order: 312 (4.585%)<br>• class: 93 (1.366%)<br>• phylum: 427 (6.275%)<br>• superkingdom: 273 (4.012%)<br>• root: 452 (6.643%)	• Cupriavidus necator [taxid 106590]: 15 (0.22%)<br>• Cupriavidus oxalaticus [taxid 96344]: 11 (0.161%)<br>• Cupriavidus neocaledonicus [taxid 1040979]: 10 (0.146%)<br>• Cupriavidus gilardii [taxid 82541]: 5 (0.073%)<br>• Cupriavidus pinatubonensis [taxid 248026]: 4 (0.058%)<br>• Cupriavidus alkaliphilus [taxid 942866]: 4 (0.058%)<br>• Cupriavidus basilensis [taxid 68895]: 3 (0.044%)<br>• Caballeronia sordidicola [taxid 196367]: 2 (0.029%)<br>• other: 32 (0.47%)

Operational Taxonomic Unit (OTU)	Correct identifications	Wrong or overspecific identifications at species rank
Benchmark OTU ID: `CU633750-_Proteobacteria`<br>OTU taxon: Cupriavidus taiwanensis LMG 19424 [taxid 977880]<br>Expected: Cupriavidus taiwanensis [taxid 164546] (species)<br>Number of reads: 4746<br>Number of identified reads: 4727 (99.599%)	• species: 1407 (29.646%)<br>• **genus: 2068 (43.573%)**<br>• family: 191 (4.024%)<br>• order: 170 (3.581%)<br>• class: 31 (0.653%)<br>• phylum: 321 (6.763%)<br>• superkingdom: 199 (4.193%)<br>• root: 338 (7.121%)	• Cupriavidus necator [taxid 106590]: 12 (0.252%)<br>• Cupriavidus pinatubonensis [taxid 248026]: 6 (0.126%)<br>• Cupriavidus neocaledonicus [taxid 1040979]: 4 (0.084%)<br>• Cupriavidus basilensis [taxid 68895]: 2 (0.042%)<br>• Cupriavidus oxalaticus [taxid 96344]: 2 (0.042%)<br>• Trinickia symbiotica [taxid 863227]: 1 (0.021%)<br>• Heterocapsa rotundata [taxid 89963]: 1 (0.021%)<br>• Cupriavidus pauculus [taxid 82633]: 1 (0.021%)<br>• other: 12 (0.252%)
Benchmark OTU ID: `CP003877-_Actinobacteria`<br>OTU taxon: Cutibacterium acnes C1 [taxid 1234380]<br>Expected: Cutibacterium acnes [taxid 1747] (species)<br>Number of reads: 9940<br>Number of identified reads: 9871 (99.305%)	• species: 2041 (20.533%)<br>• **genus: 2358 (23.722%)**<br>• family: 2267 (22.806%)<br>• order: 47 (0.472%)<br>• class: 917 (9.225%)<br>• phylum: 10 (0.1%)<br>• superkingdom: 717 (7.213%)<br>• root: 1490 (14.989%)	• [Propionibacterium] humerusii [taxid 1050843]: 58 (0.583%)<br>• Cutibacterium avidum [taxid 33010]: 26 (0.261%)<br>• Corynebacterium variabile [taxid 1727]: 9 (0.09%)<br>• Cutibacterium namnetense [taxid 1574624]: 9 (0.09%)<br>• Cutibacterium granulosum [taxid 33011]: 9 (0.09%)<br>• Corynebacterium camporealensis [taxid 161896]: 3 (0.03%)<br>• Ditylum brightwellii [taxid 49249]: 3 (0.03%)<br>• Enterobius vermicularis [taxid 51028]: 2 (0.02%)<br>• other: 41 (0.412%)
Benchmark OTU ID: `CP003293-_Actinobacteria`<br>OTU taxon: Cutibacterium acnes HL096PA1 [taxid 1134454]<br>Expected: Cutibacterium acnes [taxid 1747] (species)<br>Number of reads: 9818<br>Number of identified reads: 9756 (99.368%)	• species: 2204 (22.448%)<br>• **genus: 2263 (23.049%)**<br>• family: 2159 (21.99%)<br>• order: 32 (0.325%)<br>• class: 871 (8.871%)<br>• phylum: 9 (0.091%)<br>• superkingdom: 698 (7.109%)<br>• root: 1500 (15.278%)	• [Propionibacterium] humerusii [taxid 1050843]: 25 (0.254%)<br>• Cutibacterium avidum [taxid 33010]: 20 (0.203%)<br>• Cutibacterium granulosum [taxid 33011]: 7 (0.071%)<br>• Cutibacterium namnetense [taxid 1574624]: 6 (0.061%)<br>• Enterobius vermicularis [taxid 51028]: 4 (0.04%)<br>• Ditylum brightwellii [taxid 49249]: 4 (0.04%)<br>• Nocardia cerradoensis [taxid 85688]: 2 (0.02%)<br>• Thalassiosira rotula [taxid 49265]: 2 (0.02%)<br>• other: 33 (0.336%)
Benchmark OTU ID: `CP003196-_Actinobacteria`<br>OTU taxon: Cutibacterium acnes TypeIA2 P.acn17 [taxid 1114967]<br>Expected: Cutibacterium acnes [taxid 1747] (species)<br>Number of reads: 9959<br>Number of identified reads: 9895 (99.357%)	• **species: 2364 (23.737%)**<br>• genus: 2300 (23.094%)<br>• family: 2097 (21.056%)<br>• order: 41 (0.411%)<br>• class: 835 (8.384%)<br>• phylum: 10 (0.1%)<br>• superkingdom: 734 (7.37%)<br>• root: 1491 (14.971%)	• [Propionibacterium] humerusii [taxid 1050843]: 29 (0.291%)<br>• Cutibacterium avidum [taxid 33010]: 23 (0.23%)<br>• Cutibacterium granulosum [taxid 33011]: 9 (0.09%)<br>• Cutibacterium namnetense [taxid 1574624]: 5 (0.05%)<br>• Acidipropionibacterium acidipropionici [taxid 1748]: 4 (0.04%)<br>• Thalassiosira rotula [taxid 49265]: 4 (0.04%)<br>• Acidipropionibacterium jensenii [taxid 1749]: 2 (0.02%)<br>• Enterobius vermicularis [taxid 51028]: 2 (0.02%)<br>• other: 35 (0.351%)

Operational Taxonomic Unit (OTU)	Correct identifications	Wrong or overspecific identifications at species rank
Benchmark OTU ID: `CP003197-_Actinobacteria`<br>OTU taxon: Cutibacterium acnes TypeIA2 P.acn31 [taxid 1114969]<br>Expected: Cutibacterium acnes [taxid 1747] (species)<br>Number of reads: 9841<br>Number of identified reads: 9772 (99.298%)	• species: 2175 (22.101%)<br>• **genus: 2315 (23.524%)**<br>• family: 2193 (22.284%)<br>• order: 42 (0.426%)<br>• class: 796 (8.088%)<br>• phylum: 13 (0.132%)<br>• superkingdom: 747 (7.59%)<br>• root: 1473 (14.967%)	• [Propionibacterium] humerusii [taxid 1050843]: 28 (0.284%)<br>• Cutibacterium avidum [taxid 33010]: 16 (0.162%)<br>• Cutibacterium granulosum [taxid 33011]: 5 (0.05%)<br>• Enterobius vermicularis [taxid 51028]: 5 (0.05%)<br>• Cutibacterium namnetense [taxid 1574624]: 5 (0.05%)<br>• Phlebotomus kandelakii [taxid 1109342]: 2 (0.02%)<br>• Acidipropionibacterium acidipropionici [taxid 1748]: 2 (0.02%)<br>• Propionimicrobium lymphophilum [taxid 33012]: 2 (0.02%)<br>• other: 31 (0.315%)
Benchmark OTU ID: `CP003195-_Actinobacteria`<br>OTU taxon: Cutibacterium acnes TypeIA2 P.acn33 [taxid 1114966]<br>Expected: Cutibacterium acnes [taxid 1747] (species)<br>Number of reads: 9794<br>Number of identified reads: 9735 (99.397%)	• species: 2237 (22.84%)<br>• **genus: 2256 (23.034%)**<br>• family: 2172 (22.176%)<br>• order: 40 (0.408%)<br>• class: 884 (9.025%)<br>• phylum: 13 (0.132%)<br>• superkingdom: 672 (6.861%)<br>• root: 1450 (14.804%)	• [Propionibacterium] humerusii [taxid 1050843]: 30 (0.306%)<br>• Cutibacterium avidum [taxid 33010]: 12 (0.122%)<br>• Cutibacterium namnetense [taxid 1574624]: 9 (0.091%)<br>• Cutibacterium granulosum [taxid 33011]: 4 (0.04%)<br>• Enterobius vermicularis [taxid 51028]: 3 (0.03%)<br>• Ornithodoros moubata [taxid 6938]: 2 (0.02%)<br>• Acidipropionibacterium jensenii [taxid 1749]: 2 (0.02%)<br>• Luteococcus japonicus [taxid 33984]: 1 (0.01%)<br>• other: 32 (0.326%)
Benchmark OTU ID: `CP003084-_Actinobacteria`<br>OTU taxon: Cutibacterium acnes subsp. defendens ATCC 11828 [taxid 1091045]<br>Expected: Cutibacterium acnes [taxid 1747] (species)<br>Number of reads: 9791<br>Number of identified reads: 9731 (99.387%)	• **species: 2870 (29.312%)**<br>• genus: 2308 (23.572%)<br>• family: 1503 (15.35%)<br>• order: 40 (0.408%)<br>• class: 775 (7.915%)<br>• phylum: 4 (0.04%)<br>• superkingdom: 757 (7.731%)<br>• root: 1453 (14.84%)	• [Propionibacterium] humerusii [taxid 1050843]: 32 (0.326%)<br>• Cutibacterium avidum [taxid 33010]: 18 (0.183%)<br>• Cutibacterium granulosum [taxid 33011]: 5 (0.051%)<br>• Cutibacterium namnetense [taxid 1574624]: 4 (0.04%)<br>• Mycobacterium tuberculosis [taxid 1773]: 3 (0.03%)<br>• Thalassiosira rotula [taxid 49265]: 3 (0.03%)<br>• Acidipropionibacterium virtanenii [taxid 2057246]: 3 (0.03%)<br>• Acidipropionibacterium jensenii [taxid 1749]: 2 (0.02%)<br>• other: 32 (0.326%)
Benchmark OTU ID: `CP005287-_Actinobacteria`<br>OTU taxon: Cutibacterium avidum 44067 [taxid 1170318]<br>Expected: Cutibacterium avidum [taxid 33010] (species)<br>Number of reads: 9975<br>Number of identified reads: 9929 (99.538%)	• **species: 4466 (44.771%)**<br>• genus: 1519 (15.228%)<br>• family: 879 (8.812%)<br>• order: 47 (0.471%)<br>• class: 811 (8.13%)<br>• phylum: 17 (0.17%)<br>• superkingdom: 647 (6.486%)<br>• root: 1522 (15.258%)	• Cutibacterium acnes [taxid 1747]: 41 (0.411%)<br>• [Propionibacterium] humerusii [taxid 1050843]: 21 (0.21%)<br>• Corynebacterium falsenii [taxid 108486]: 15 (0.15%)<br>• Cutibacterium granulosum [taxid 33011]: 12 (0.12%)<br>• Varibaculum cambriense [taxid 184870]: 8 (0.08%)<br>• Acidipropionibacterium virtanenii [taxid 2057246]: 7 (0.07%)<br>• Propionibacterium cyclohexanicum [taxid 64702]: 3 (0.03%)<br>• Schaalia turicensis [taxid 131111]: 2 (0.02%)<br>• other: 49 (0.491%)

Operational Taxonomic Unit (OTU)	Correct identifications	Wrong or overspecific identifications at species rank
Benchmark OTU ID: `CP003947-_Cyanobacteria`<br>OTU taxon: Cyanobacterium aponinum PCC 10605 [taxid 755178]<br>Expected: Cyanobacterium aponinum [taxid 379064] (species)<br>Number of reads: 23292<br>Number of identified reads: 22727 (97.574%)	• **species: 12868 (55.246%)**<br>• genus: 123 (0.528%)<br>• family: 0 (0.0%)<br>• order: 654 (2.807%)<br>• phylum: 5103 (21.908%)<br>• superkingdom: 1355 (5.817%)<br>• root: 2590 (11.119%)	• Calothrix parietina [taxid 32054]: 4 (0.017%)<br>• Phormidesmis priestleyi [taxid 268141]: 4 (0.017%)<br>• Cyanobacterium stanieri [taxid 102235]: 4 (0.017%)<br>• Gloeomargarita lithophora [taxid 1188228]: 3 (0.012%)<br>• Hyella patelloides [taxid 1982969]: 2 (0.008%)<br>• Prochlorococcus marinus [taxid 1219]: 2 (0.008%)<br>• Acaryochloris marina [taxid 155978]: 2 (0.008%)<br>• other: 57 (0.244%)
Benchmark OTU ID: `CP003940-_Cyanobacteria`<br>OTU taxon: Cyanobacterium stanieri PCC 7202 [taxid 292563]<br>Expected: Cyanobacterium stanieri [taxid 102235] (species)<br>Number of reads: 17018<br>Number of identified reads: 16785 (98.63%)	• **species: 10437 (61.329%)**<br>• genus: 1518 (8.919%)<br>• family: 0 (0.0%)<br>• order: 429 (2.52%)<br>• phylum: 1436 (8.438%)<br>• superkingdom: 989 (5.811%)<br>• root: 1928 (11.329%)	• Cyanobacterium aponinum [taxid 379064]: 15 (0.088%)<br>• Prochlorococcus marinus [taxid 1219]: 3 (0.017%)<br>• Solanum chacoense [taxid 4108]: 3 (0.017%)<br>• Crocosphaera chwakensis [taxid 2546361]: 2 (0.011%)<br>• Pseudomonas japonica [taxid 256466]: 1 (0.005%)<br>• Streptomyces hygroscopicus [taxid 1912]: 1 (0.005%)<br>• Prorocentrum minimum [taxid 39449]: 1 (0.005%)<br>• other: 36 (0.211%)
Benchmark OTU ID: `CP003495-_Cyanobacteria`<br>OTU taxon: Cyanobium gracile PCC 6307 [taxid 292564]<br>Expected: Cyanobium gracile [taxid 59930] (species)<br>Number of reads: 18199<br>Number of identified reads: 18120 (99.565%)	• **species: 7841 (43.084%)**<br>• genus: 365 (2.005%)<br>• family: 515 (2.829%)<br>• order: 1094 (6.011%)<br>• phylum: 4585 (25.193%)<br>• superkingdom: 1819 (9.995%)<br>• root: 1858 (10.209%)	• Aphanothece minutissima [taxid 543815]: 74 (0.406%)<br>• Synechococcus sp. Ace-Pa [taxid 2572902]: 14 (0.076%)<br>• Synechococcus elongatus [taxid 32046]: 2 (0.01%)<br>• Prochlorococcus marinus [taxid 1219]: 2 (0.01%)<br>• Candidatus Synechococcus spongiarum [taxid 431041]: 2 (0.01%)<br>• Synechococcus sp. Lanier [taxid 1910957]: 2 (0.01%)<br>• Synechococcus lacustris [taxid 2116544]: 2 (0.01%)<br>• other: 56 (0.307%)
Benchmark OTU ID: `CP001344-_Cyanobacteria`<br>OTU taxon: Cyanothece sp. PCC 7425 [taxid 395961]<br>Expected: Cyanothece [taxid 43988] (genus)<br>Number of reads: 31612<br>Number of identified reads: 31457 (99.509%)	• **genus: 21883 (69.223%)**<br>• family: 0 (0.0%)<br>• order: 196 (0.62%)<br>• phylum: 2517 (7.962%)<br>• superkingdom: 2152 (6.807%)<br>• root: 4639 (14.674%)	• Phormidesmis priestleyi [taxid 268141]: 5 (0.015%)<br>• Leptolyngbya sp. 'hensonii' [taxid 1922337]: 4 (0.012%)<br>• Stenomitos frigidus [taxid 1886765]: 3 (0.009%)<br>• Cyanosarcina burmensis [taxid 2107696]: 3 (0.009%)<br>• Thermosynechococcus elongatus [taxid 146786]: 3 (0.009%)<br>• Acaryochloris marina [taxid 155978]: 2 (0.006%)<br>• other: 94 (0.297%)
Benchmark OTU ID: `CP002955-_Bacteroidetes`<br>OTU taxon: Cyclobacterium marinum DSM 745 [taxid 880070]<br>Expected: Cyclobacterium marinum [taxid 104] (species)<br>Number of reads: 43470<br>Number of identified reads: 42871 (98.622%)	• **species: 26466 (60.883%)**<br>• genus: 5413 (12.452%)<br>• family: 901 (2.072%)<br>• order: 684 (1.573%)<br>• class: 0 (0.0%)<br>• phylum: 2079 (4.782%)<br>• superkingdom: 1964 (4.518%)<br>• root: 5341 (12.286%)	• Cyclobacterium amurskyense [taxid 320787]: 102 (0.234%)<br>• Cyclobacterium qasimii [taxid 1350429]: 87 (0.2%)<br>• Cyclobacterium lianum [taxid 388280]: 14 (0.032%)<br>• Cyclobacterium xiamenense [taxid 1297121]: 13 (0.029%)<br>• Lupinus albus [taxid 3870]: 3 (0.006%)<br>• Rhodonellum psychrophilum [taxid 336828]: 3 (0.006%)<br>• Mariniradius saccharolyticus [taxid 1245591]: 2 (0.004%)<br>• Echinicola vietnamensis [taxid 390884]: 2 (0.004%)<br>• other: 80 (0.184%)

Operational Taxonomic Unit (OTU)	Correct identifications	Wrong or overspecific identifications at species rank
Benchmark OTU ID: `CP003642-_Cyanobacteria`<br>OTU taxon: Cylindrospermum stagnale PCC 7417 [taxid 56107]<br>Expected: Cylindrospermum stagnale [taxid 142864] (species)<br>Number of reads: 42363<br>Number of identified reads: 41847 (98.781%)	• **species: 22532 (53.187%)**<br>• genus: 1055 (2.49%)<br>• family: 1818 (4.291%)<br>• order: 4469 (10.549%)<br>• phylum: 3103 (7.324%)<br>• superkingdom: 2089 (4.931%)<br>• root: 6704 (15.825%)	• Nodularia spumigena [taxid 70799]: 10 (0.023%)<br>• Nostoc minutum [taxid 1841509]: 9 (0.021%)<br>• Anabaena cylindrica [taxid 1165]: 9 (0.021%)<br>• Trichormus azollae [taxid 1164]: 9 (0.021%)<br>• Nostoc sphaeroides [taxid 446679]: 8 (0.018%)<br>• Trichormus variabilis [taxid 264691]: 7 (0.016%)<br>• Calothrix parietina [taxid 32054]: 6 (0.014%)<br>• other: 169 (0.398%)
Benchmark OTU ID: `ENA|BX842601|BX842601.2-_Proteobacteria`<br>OTU taxon: Cythereinae [taxid 50701]<br>Expected: Cythereinae [taxid 50701] (subfamily)<br>Number of reads: 7628<br>Number of identified reads: 7622 (99.921%)	• family: 0 (0.0%)<br>• order: 0 (0.0%)<br>• class: 0 (0.0%)<br>• phylum: 0 (0.0%)<br>• superkingdom: 9 (0.117%)<br>• **root: 746 (9.779%)**	• **Bdellovibrio bacteriovorus [taxid 959]: 5837 (76.52%)**<br>• Pseudomonas fluorescens [taxid 294]: 1 (0.013%)<br>• Bdellovibrio sp. ArHS [taxid 1569284]: 1 (0.013%)<br>• Candidatus Blochmannia pennsylvanicus [taxid 101534]: 1 (0.013%)<br>• Eggerthella sinensis [taxid 242230]: 1 (0.013%)<br>• Arcobacter ellisii [taxid 913109]: 1 (0.013%)<br>• other: 8 (0.104%)
Benchmark OTU ID: `CP000383-_Bacteroidetes`<br>OTU taxon: Cytophaga hutchinsonii ATCC 33406 [taxid 269798]<br>Expected: Cytophaga hutchinsonii [taxid 985] (species)<br>Number of reads: 29881<br>Number of identified reads: 29642 (99.2%)	• **species: 23319 (78.039%)**<br>• genus: 4 (0.013%)<br>• family: 110 (0.368%)<br>• order: 274 (0.916%)<br>• class: 1 (0.003%)<br>• phylum: 1334 (4.464%)<br>• superkingdom: 1534 (5.133%)<br>• root: 3045 (10.19%)	• Ephemera danica [taxid 1049336]: 2 (0.006%)<br>• Escherichia coli [taxid 562]: 2 (0.006%)<br>• Phytophthora megakarya [taxid 4795]: 2 (0.006%)<br>• Pedobacter xixiisoli [taxid 1476464]: 1 (0.003%)<br>• Bacteroides helcogenes [taxid 290053]: 1 (0.003%)<br>• Amanita thiersii [taxid 235537]: 1 (0.003%)<br>• Helicobacter pylori [taxid 210]: 1 (0.003%)<br>• Antithamnionella ternifolia [taxid 207919]: 1 (0.003%)<br>• other: 32 (0.107%)
Benchmark OTU ID: `CP003944-_Cyanobacteria`<br>OTU taxon: Dactylococcopsis salina PCC 8305 [taxid 13035]<br>Expected: Dactylococcopsis salina [taxid 292566] (species)<br>Number of reads: 21095<br>Number of identified reads: 20848 (98.829%)	• species: 6345 (30.078%)<br>• genus: 0 (0.0%)<br>• family: 25 (0.118%)<br>• order: 63 (0.298%)<br>• **phylum: 10328 (48.959%)**<br>• superkingdom: 1173 (5.56%)<br>• root: 2861 (13.562%)	• Euhalothece natronophila [taxid 577489]: 37 (0.175%)<br>• Rubidibacter lacunae [taxid 582514]: 7 (0.033%)<br>• Microcystis aeruginosa [taxid 1126]: 5 (0.023%)<br>• Trichormus azollae [taxid 1164]: 4 (0.018%)<br>• Hyella patelloides [taxid 1982969]: 4 (0.018%)<br>• Moorea sp. SIOASIH [taxid 2607817]: 3 (0.014%)<br>• Arthrospira platensis [taxid 118562]: 3 (0.014%)<br>• other: 100 (0.474%)
Benchmark OTU ID: `CP000027-_Chloroflexi`<br>OTU taxon: Dehalococcoides mccartyi 195 [taxid 243164]<br>Expected: Dehalococcoides mccartyi [taxid 61435] (species)<br>Number of reads: 28810<br>Number of identified reads: 28706 (99.639%)	• species: 6774 (23.512%)<br>• genus: 876 (3.04%)<br>• family: 0 (0.0%)<br>• order: 6 (0.02%)<br>• class: 145 (0.503%)<br>• phylum: 308 (1.069%)<br>• superkingdom: 8139 (28.25%)<br>• **root: 12312 (42.735%)**	• Candidatus Weimeria bifida [taxid 2599074]: 19 (0.065%)<br>• Hespellia stercorisuis [taxid 180311]: 9 (0.031%)<br>• Helicobacter pylori [taxid 210]: 7 (0.024%)<br>• Enterocloster clostridioformis [taxid 1531]: 5 (0.017%)<br>• bacterium [taxid 1869227]: 4 (0.013%)<br>• Dehalogenimonas sp. GP [taxid 1536648]: 3 (0.01%)<br>• Dehalogenimonas lykanthroporepellens [taxid 552810]: 3 (0.01%)<br>• Dehalogenimonas alkenigignens [taxid 1217799]: 3 (0.01%)<br>• other: 244 (0.846%)

Operational Taxonomic Unit (OTU)	Correct identifications	Wrong or overspecific identifications at species rank
Benchmark OTU ID: `CP000688-_Chloroflexi`<br>OTU taxon: Dehalococcoides mccartyi BAV1 [taxid 216389]<br>Expected: Dehalococcoides mccartyi [taxid 61435] (species)<br>Number of reads: 25267<br>Number of identified reads: 25153 (99.548%)	• **species: 12602 (49.875%)**<br>• genus: 1665 (6.589%)<br>• family: 0 (0.0%)<br>• order: 6 (0.023%)<br>• class: 172 (0.68%)<br>• phylum: 225 (0.89%)<br>• superkingdom: 4023 (15.921%)<br>• root: 6407 (25.357%)	• Dehalogenimonas lykanthroporepellens [taxid 552810]: 10 (0.039%)<br>• Salmonella enterica [taxid 28901]: 5 (0.019%)<br>• Gardnerella vaginalis [taxid 2702]: 4 (0.015%)<br>• Bathymodiolus thermophilus thioautotrophic gill symbiont [taxid 2360]: 3 (0.011%)<br>• Dehalogenimonas formicexedens [taxid 1839801]: 3 (0.011%)<br>• Dehalogenimonas alkenigignens [taxid 1217799]: 2 (0.007%)<br>• Candidatus Hakubanella thermoalkaliphilus [taxid 2754717]: 2 (0.007%)<br>• Candidatus Magnetobacterium bavaricum [taxid 29290]: 2 (0.007%)<br>• other: 90 (0.356%)
Benchmark OTU ID: `CP004080-_Chloroflexi`<br>OTU taxon: Dehalococcoides mccartyi BTF08 [taxid 1193806]<br>Expected: Dehalococcoides mccartyi [taxid 61435] (species)<br>Number of reads: 28328<br>Number of identified reads: 28186 (99.498%)	• **species: 14413 (50.878%)**<br>• genus: 1984 (7.003%)<br>• family: 0 (0.0%)<br>• order: 9 (0.031%)<br>• class: 171 (0.603%)<br>• phylum: 210 (0.741%)<br>• superkingdom: 4776 (16.859%)<br>• root: 6566 (23.178%)	• Oscillibacter valericigenes [taxid 351091]: 106 (0.374%)<br>• Acetanaerobacterium elongatum [taxid 258515]: 70 (0.247%)<br>• Desulfosporosinus sp. Tol-M [taxid 1536651]: 39 (0.137%)<br>• Aminicella lysinilytica [taxid 433323]: 28 (0.098%)<br>• Hespellia stercorisuis [taxid 180311]: 10 (0.035%)<br>• Dehalogenimonas alkenigignens [taxid 1217799]: 6 (0.021%)<br>• Salmonella enterica [taxid 28901]: 4 (0.014%)<br>• Macrococcus bohemicus [taxid 1903056]: 4 (0.014%)<br>• other: 128 (0.451%)
Benchmark OTU ID: `AJ965256-_Chloroflexi`<br>OTU taxon: Dehalococcoides mccartyi CBDB1 [taxid 255470]<br>Expected: Dehalococcoides mccartyi [taxid 61435] (species)<br>Number of reads: 26750<br>Number of identified reads: 26636 (99.573%)	• **species: 14477 (54.119%)**<br>• genus: 1803 (6.74%)<br>• family: 0 (0.0%)<br>• order: 11 (0.041%)<br>• class: 172 (0.642%)<br>• phylum: 247 (0.923%)<br>• superkingdom: 3923 (14.665%)<br>• root: 5964 (22.295%)	• Dehalogenimonas formicexedens [taxid 1839801]: 6 (0.022%)<br>• Dehalogenimonas sp. GP [taxid 1536648]: 4 (0.014%)<br>• Helicobacter pylori [taxid 210]: 2 (0.007%)<br>• bacterium [taxid 1869227]: 2 (0.007%)<br>• Tengunoibacter tsumagoiensis [taxid 2014871]: 2 (0.007%)<br>• Niastella caeni [taxid 2569763]: 1 (0.003%)<br>• Orientia chuto [taxid 911112]: 1 (0.003%)<br>• Alcanivorax borkumensis [taxid 59754]: 1 (0.003%)<br>• other: 93 (0.347%)
Benchmark OTU ID: `CP004079-_Chloroflexi`<br>OTU taxon: Dehalococcoides mccartyi DCMB5 [taxid 1193807]<br>Expected: Dehalococcoides mccartyi [taxid 61435] (species)<br>Number of reads: 27763<br>Number of identified reads: 27605 (99.43%)	• **species: 15054 (54.223%)**<br>• genus: 1552 (5.59%)<br>• family: 0 (0.0%)<br>• order: 3 (0.01%)<br>• class: 177 (0.637%)<br>• phylum: 223 (0.803%)<br>• superkingdom: 4314 (15.538%)<br>• root: 6225 (22.421%)	• Dehalogenimonas alkenigignens [taxid 1217799]: 8 (0.028%)<br>• Desulfosporosinus sp. OL [taxid 1888891]: 6 (0.021%)<br>• Salmonella enterica [taxid 28901]: 6 (0.021%)<br>• Dehalogenimonas lykanthroporepellens [taxid 552810]: 3 (0.01%)<br>• bacterium [taxid 1869227]: 3 (0.01%)<br>• Desulfobacterium vacuolatum [taxid 2298]: 2 (0.007%)<br>• Dehalogenimonas sp. GP [taxid 1536648]: 2 (0.007%)<br>• Prevotella copri [taxid 165179]: 2 (0.007%)<br>• other: 105 (0.378%)

Operational Taxonomic Unit (OTU)	Correct identifications	Wrong or overspecific identifications at species rank
Benchmark OTU ID: `CP001924-_Chloroflexi`<br>OTU taxon: Dehalococcoides mccartyi GT [taxid 633145]<br>Expected: Dehalococcoides mccartyi [taxid 61435] (species)<br>Number of reads: 25774<br>Number of identified reads: 25654 (99.534%)	• **species: 13655 (52.979%)**<br>• genus: 1967 (7.631%)<br>• family: 0 (0.0%)<br>• order: 5 (0.019%)<br>• class: 142 (0.55%)<br>• phylum: 225 (0.872%)<br>• superkingdom: 3856 (14.96%)<br>• root: 5755 (22.328%)	• Desulfatirhabdium butyrativorans [taxid 340467]: 4 (0.015%)<br>• bacterium [taxid 1869227]: 3 (0.011%)<br>• Lactobacillus uvarum [taxid 303240]: 2 (0.007%)<br>• Breznakibacter xylanolyticus [taxid 990]: 2 (0.007%)<br>• Helicobacter pylori [taxid 210]: 2 (0.007%)<br>• Halomonas chromatireducens [taxid 507626]: 1 (0.003%)<br>• Geotrichum candidum [taxid 1173061]: 1 (0.003%)<br>• Salmonella enterica [taxid 28901]: 1 (0.003%)<br>• other: 92 (0.356%)
Benchmark OTU ID: `CP001827-_Chloroflexi`<br>OTU taxon: Dehalococcoides mccartyi VS [taxid 311424]<br>Expected: Dehalococcoides mccartyi [taxid 61435] (species)<br>Number of reads: 27250<br>Number of identified reads: 27138 (99.588%)	• species: 6456 (23.691%)<br>• **genus: 8022 (29.438%)**<br>• family: 0 (0.0%)<br>• order: 4 (0.014%)<br>• class: 165 (0.605%)<br>• phylum: 249 (0.913%)<br>• superkingdom: 4951 (18.168%)<br>• root: 7215 (26.477%)	• bacterium [taxid 1869227]: 4 (0.014%)<br>• Couchioplanes caeruleus [taxid 56438]: 2 (0.007%)<br>• Methanosarcinales archaeon [taxid 2250255]: 2 (0.007%)<br>• Yoonia maricola [taxid 420999]: 2 (0.007%)<br>• Dehalogenimonas formicexedens [taxid 1839801]: 2 (0.007%)<br>• Dehalogenimonas lykanthroporepellens [taxid 552810]: 2 (0.007%)<br>• Eumeta japonica [taxid 151549]: 2 (0.007%)<br>• Algoriphagus boritolerans [taxid 308111]: 1 (0.003%)<br>• other: 148 (0.543%)
Benchmark OTU ID: `CP002084-_Chloroflexi`<br>OTU taxon: Dehalogenimonas lykanthroporepellens BL-DC-9 [taxid 552811]<br>Expected: Dehalogenimonas lykanthroporepellens [taxid 552810] (species)<br>Number of reads: 34815<br>Number of identified reads: 34726 (99.744%)	• **species: 23627 (67.864%)**<br>• genus: 600 (1.723%)<br>• class: 550 (1.579%)<br>• phylum: 208 (0.597%)<br>• superkingdom: 4615 (13.255%)<br>• root: 5092 (14.625%)	• Dehalogenimonas formicexedens [taxid 1839801]: 18 (0.051%)<br>• Dehalogenimonas alkenigignens [taxid 1217799]: 12 (0.034%)<br>• Pseudodesulfovibrio profundus [taxid 57320]: 6 (0.017%)<br>• Dehalococcoides mccartyi [taxid 61435]: 4 (0.011%)<br>• Desulfovibrio marinus [taxid 370038]: 3 (0.008%)<br>• Anaeromyces robustus [taxid 1754192]: 2 (0.005%)<br>• other: 84 (0.241%)
Benchmark OTU ID: `CP003273-_Firmicutes`<br>OTU taxon: Desulfallas gibsoniae DSM 7213 [taxid 767817]<br>Expected: Desulfallas gibsoniae [taxid 102134] (species)<br>Number of reads: 7000<br>Number of identified reads: 6933 (99.042%)	• **species: 4345 (62.071%)**<br>• genus: 312 (4.457%)<br>• family: 215 (3.071%)<br>• order: 85 (1.214%)<br>• class: 22 (0.314%)<br>• phylum: 209 (2.985%)<br>• superkingdom: 536 (7.657%)<br>• root: 1204 (17.2%)	• Desulfallas arcticus [taxid 341036]: 10 (0.142%)<br>• Desulfallas thermosapovorans [taxid 58137]: 9 (0.128%)<br>• Desulfallas geothermicus [taxid 39060]: 2 (0.028%)<br>• Pelotomaculum sp. FP [taxid 261474]: 2 (0.028%)<br>• Pelotomaculum thermopropionicum [taxid 110500]: 1 (0.014%)<br>• Dickeya poaceiphila [taxid 568768]: 1 (0.014%)<br>• Bacillus thuringiensis [taxid 1428]: 1 (0.014%)<br>• Vibrio rumoiensis [taxid 76258]: 1 (0.014%)<br>• other: 25 (0.357%)

Operational Taxonomic Unit (OTU)	Correct identifications	Wrong or overspecific identifications at species rank
Benchmark OTU ID: `CP002085-_Proteobacteria`<br>OTU taxon: Desulfarculus baarsii DSM 2075 [taxid 644282]<br>Expected: Desulfarculus baarsii [taxid 453230] (species)<br>Number of reads: 7341<br>Number of identified reads: 7305 (99.509%)	• **species: 5701 (77.659%)**<br>• genus: 6 (0.081%)<br>• family: 32 (0.435%)<br>• order: 0 (0.0%)<br>• class: 124 (1.689%)<br>• phylum: 267 (3.637%)<br>• superkingdom: 571 (7.778%)<br>• root: 603 (8.214%)	• Desulfomicrobium norvegicum [taxid 52561]: 6 (0.081%)<br>• Thiohalophilus thiocyanatoxydans [taxid 381308]: 1 (0.013%)<br>• Syntrophomonas wolfei [taxid 863]: 1 (0.013%)<br>• Marispirochaeta aestuarii [taxid 1963862]: 1 (0.013%)<br>• Geobacter soli [taxid 1510391]: 1 (0.013%)<br>• Actinopolymorpha cephalotaxi [taxid 504797]: 1 (0.013%)<br>• Syntrophobacter fumaroxidans [taxid 119484]: 1 (0.013%)<br>• Hordeum vulgare [taxid 4513]: 1 (0.013%)<br>• other: 3 (0.04%)
Benchmark OTU ID: `CP001322-_Proteobacteria`<br>OTU taxon: Desulfatibacillum aliphaticivorans [taxid 218208]<br>Expected: Desulfatibacillum aliphaticivorans [taxid 218208] (species)<br>Number of reads: 13779<br>Number of identified reads: 13736 (99.687%)	• **species: 6867 (49.836%)**<br>• genus: 3675 (26.671%)<br>• family: 66 (0.478%)<br>• order: 13 (0.094%)<br>• class: 155 (1.124%)<br>• phylum: 258 (1.872%)<br>• superkingdom: 661 (4.797%)<br>• root: 2027 (14.71%)	• Desulfatibacillum alkenivorans [taxid 259354]: 77 (0.558%)<br>• Desulfovibrio bizertensis [taxid 376490]: 1 (0.007%)<br>• Desulfoglaeba alkanexedens [taxid 361111]: 1 (0.007%)<br>• Smithella sp. SDB [taxid 1735324]: 1 (0.007%)<br>• Flavobacterium circumlabens [taxid 2133765]: 1 (0.007%)<br>• Rhizobium leguminosarum [taxid 384]: 1 (0.007%)<br>• Massilia glaciei [taxid 1524097]: 1 (0.007%)<br>• Parahaliea aestuarii [taxid 1852021]: 1 (0.007%)<br>• other: 20 (0.145%)
Benchmark OTU ID: `CP001336-_Firmicutes`<br>OTU taxon: Desulfitobacterium hafniense DCB-2 [taxid 272564]<br>Expected: Desulfitobacterium hafniense [taxid 49338] (species)<br>Number of reads: 7678<br>Number of identified reads: 7641 (99.518%)	• species: 1116 (14.535%)<br>• genus: 732 (9.533%)<br>• family: 308 (4.011%)<br>• order: 210 (2.735%)<br>• class: 27 (0.351%)<br>• phylum: 529 (6.889%)<br>• superkingdom: 1471 (19.158%)<br>• **root: 3209 (41.794%)**	• Desulfitobacterium dehalogenans [taxid 36854]: 22 (0.286%)<br>• Desulfitobacterium chlororespirans [taxid 51616]: 16 (0.208%)<br>• Desulfitobacterium dichloroeliminans [taxid 233055]: 9 (0.117%)<br>• Desulfitobacterium sp. LBE [taxid 884086]: 8 (0.104%)<br>• Desulfosporosinus sp. Tol-M [taxid 1536651]: 3 (0.039%)<br>• Candidatus Desulfosporosinus infrequens [taxid 2043169]: 2 (0.026%)<br>• Desulfitobacterium metallireducens [taxid 142877]: 2 (0.026%)<br>• Desulfotomaculum copahuensis [taxid 1838280]: 2 (0.026%)<br>• other: 100 (1.302%)
Benchmark OTU ID: `CP001087-_Proteobacteria`<br>OTU taxon: Desulfobacterium autotrophicum HRM2 [taxid 177437]<br>Expected: Desulfobacterium autotrophicum [taxid 2296] (species)<br>Number of reads: 11691<br>Number of identified reads: 11663 (99.76%)	• **species: 8199 (70.13%)**<br>• genus: 41 (0.35%)<br>• family: 206 (1.762%)<br>• order: 44 (0.376%)<br>• class: 235 (2.01%)<br>• phylum: 267 (2.283%)<br>• superkingdom: 613 (5.243%)<br>• root: 2048 (17.517%)	• Desulfoplanes formicivorans [taxid 1592317]: 3 (0.025%)<br>• Desulfotignum phosphitoxidans [taxid 190898]: 2 (0.017%)<br>• Desulfovibrio gilichinskyi [taxid 1519643]: 2 (0.017%)<br>• Desulfobacter hydrogenophilus [taxid 2291]: 2 (0.017%)<br>• Lupinus albus [taxid 3870]: 2 (0.017%)<br>• Desulfobacterium vacuolatum [taxid 2298]: 1 (0.008%)<br>• Halodesulfovibrio spirochaetisodalis [taxid 1560234]: 1 (0.008%)<br>• Vombatus ursinus [taxid 29139]: 1 (0.008%)<br>• other: 18 (0.153%)

Operational Taxonomic Unit (OTU)	Correct identifications	Wrong or overspecific identifications at species rank
Benchmark OTU ID: `CP002364-_Proteobacteria`<br>OTU taxon: Desulfobulbus propionicus DSM 2032 [taxid 577650]<br>Expected: Desulfobulbus propionicus [taxid 894] (species)<br>Number of reads: 7783<br>Number of identified reads: 7758 (99.678%)	• species: 148 (1.901%)<br>• genus: 121 (1.554%)<br>• family: 359 (4.612%)<br>• order: 169 (2.171%)<br>• class: 448 (5.756%)<br>• phylum: 651 (8.364%)<br>• superkingdom: 1913 (24.579%)<br>• **root: 3920 (50.366%)**	• Desulfobulbus oralis [taxid 1986146]: 42 (0.539%)<br>• Desulfobulbus sp. Tol-SR [taxid 1536652]: 18 (0.231%)<br>• Desulfopila aestuarii [taxid 231440]: 16 (0.205%)<br>• Candidatus Electrothrix aarhusiensis [taxid 1859131]: 9 (0.115%)<br>• Candidatus Electronema sp. GS [taxid 2005002]: 8 (0.102%)<br>• Desulforhopalus singaporensis [taxid 91360]: 7 (0.089%)<br>• Desulfofustis glycolicus [taxid 51195]: 7 (0.089%)<br>• Desulfocapsa sulfexigens [taxid 65555]: 5 (0.064%)<br>• other: 178 (2.287%)
Benchmark OTU ID: `CP000859-_Proteobacteria`<br>OTU taxon: Desulfococcus oleovorans Hxd3 [taxid 96561]<br>Expected: Desulfococcus oleovorans [taxid 181663] (species)<br>Number of reads: 7990<br>Number of identified reads: 7973 (99.787%)	• **species: 6160 (77.096%)**<br>• genus: 36 (0.45%)<br>• family: 77 (0.963%)<br>• order: 17 (0.212%)<br>• class: 133 (1.664%)<br>• phylum: 177 (2.215%)<br>• superkingdom: 467 (5.844%)<br>• root: 902 (11.289%)	• Halogranum gelatinilyticum [taxid 660521]: 1 (0.012%)<br>• Desulfohalobium retbaense [taxid 45663]: 1 (0.012%)<br>• Clostridium chromiireducens [taxid 225345]: 1 (0.012%)<br>• Lichtheimia ramosa [taxid 688394]: 1 (0.012%)<br>• Janthinobacterium sp. Marseille [taxid 375286]: 1 (0.012%)<br>• Schlesneria paludicola [taxid 360056]: 1 (0.012%)<br>• Desulfosarcina alkanivorans [taxid 571177]: 1 (0.012%)<br>• Pseudoruegeria lutimaris [taxid 571298]: 1 (0.012%)<br>• other: 13 (0.162%)
Benchmark OTU ID: `CP002770-_Firmicutes`<br>OTU taxon: Desulfofundulus kuznetsovii DSM 6115 [taxid 760568]<br>Expected: Desulfofundulus kuznetsovii [taxid 58135] (species)<br>Number of reads: 4993<br>Number of identified reads: 4963 (99.399%)	• species: 0 (0.0%)<br>• **genus: 2140 (42.86%)**<br>• family: 406 (8.131%)<br>• order: 32 (0.64%)<br>• class: 133 (2.663%)<br>• phylum: 176 (3.524%)<br>• superkingdom: 662 (13.258%)<br>• root: 1403 (28.099%)	• **Desulfofundulus thermosubterraneus [taxid 348840]: 566 (11.335%)**<br>• **Desulfofundulus salinum [taxid 2419843]: 364 (7.29%)**<br>• **Desulfofundulus australicus [taxid 1566]: 265 (5.307%)**<br>• Desulfofundulus thermobenzoicus [taxid 29376]: 25 (0.5%)<br>• Candidatus Desulforudis audaxviator [taxid 471827]: 8 (0.16%)<br>• Moorella thermoacetica [taxid 1525]: 5 (0.1%)<br>• Moorella glycerini [taxid 55779]: 5 (0.1%)<br>• Desulfocucumis palustris [taxid 1898651]: 4 (0.08%)<br>• other: 57 (1.141%)
Benchmark OTU ID: `CP001629-_Proteobacteria`<br>OTU taxon: Desulfomicrobium baculatum DSM 4028 [taxid 525897]<br>Expected: Desulfomicrobium baculatum [taxid 899] (species)<br>Number of reads: 7987<br>Number of identified reads: 7973 (99.824%)	• **species: 4368 (54.688%)**<br>• genus: 590 (7.387%)<br>• family: 9 (0.112%)<br>• order: 134 (1.677%)<br>• class: 1231 (15.412%)<br>• phylum: 269 (3.367%)<br>• superkingdom: 444 (5.559%)<br>• root: 925 (11.581%)	• Desulfomicrobium norvegicum [taxid 52561]: 9 (0.112%)<br>• Desulfomicrobium apsheronum [taxid 52560]: 7 (0.087%)<br>• Desulfomicrobium orale [taxid 132132]: 5 (0.062%)<br>• Desulfoplanes formicivorans [taxid 1592317]: 2 (0.025%)<br>• Desulfovibrio gigas [taxid 879]: 2 (0.025%)<br>• Desulfovibrio sp. TomC [taxid 1562888]: 1 (0.012%)<br>• Oricola cellulosilytica [taxid 1429082]: 1 (0.012%)<br>• Ruegeria pomeroyi [taxid 89184]: 1 (0.012%)<br>• other: 15 (0.187%)

Operational Taxonomic Unit (OTU)	Correct identifications	Wrong or overspecific identifications at species rank
Benchmark OTU ID: `CP003629-_Firmicutes`<br>OTU taxon: Desulfosporosinus meridiei DSM 13257 [taxid 768704]<br>Expected: Desulfosporosinus meridiei [taxid 79209] (species)<br>Number of reads: 7029<br>Number of identified reads: 6925 (98.52%)	• species: 2295 (32.65%)<br>• **genus: 2580 (36.705%)**<br>• family: 242 (3.442%)<br>• order: 120 (1.707%)<br>• class: 24 (0.341%)<br>• phylum: 218 (3.101%)<br>• superkingdom: 476 (6.771%)<br>• root: 961 (13.671%)	• Desulfosporosinus hippei [taxid 569859]: 19 (0.27%)<br>• Desulfosporosinus lacus [taxid 329936]: 12 (0.17%)<br>• Desulfosporosinus sp. OT [taxid 913865]: 8 (0.113%)<br>• Desulfosporosinus acidiphilus [taxid 885581]: 4 (0.056%)<br>• Desulfosporosinus youngiae [taxid 339862]: 4 (0.056%)<br>• Desulfosporosinus acididurans [taxid 476652]: 3 (0.042%)<br>• Candidatus Desulfosporosinus infrequens [taxid 2043169]: 3 (0.042%)<br>• Desulfosporosinus sp. Tol-M [taxid 1536651]: 2 (0.028%)<br>• other: 35 (0.497%)
Benchmark OTU ID: `CP003108-_Firmicutes`<br>OTU taxon: Desulfosporosinus orientis DSM 765 [taxid 768706]<br>Expected: Desulfosporosinus orientis [taxid 1563] (species)<br>Number of reads: 8612<br>Number of identified reads: 8523 (98.966%)	• **species: 5308 (61.634%)**<br>• genus: 781 (9.068%)<br>• family: 283 (3.286%)<br>• order: 140 (1.625%)<br>• class: 21 (0.243%)<br>• phylum: 246 (2.856%)<br>• superkingdom: 528 (6.13%)<br>• root: 1208 (14.026%)	• Desulfosporosinus youngiae [taxid 339862]: 5 (0.058%)<br>• Desulfosporosinus acidiphilus [taxid 885581]: 4 (0.046%)<br>• Desulfosporosinus sp. BG [taxid 1633135]: 4 (0.046%)<br>• Desulfosporosinus sp. Sb-LF [taxid 2560027]: 3 (0.034%)<br>• Desulfosporosinus lacus [taxid 329936]: 3 (0.034%)<br>• Desulfosporosinus hippei [taxid 569859]: 3 (0.034%)<br>• Desulfosporosinus meridiei [taxid 79209]: 3 (0.034%)<br>• Desulfosporosinus fructosivorans [taxid 2018669]: 3 (0.034%)<br>• other: 38 (0.441%)
Benchmark OTU ID: `CP002736-_Firmicutes`<br>OTU taxon: Desulfotomaculum nigrificans CO-1-SRB [taxid 868595]<br>Expected: Desulfotomaculum nigrificans [taxid 1565] (species)<br>Number of reads: 3859<br>Number of identified reads: 3824 (99.093%)	• **species: 2231 (57.812%)**<br>• genus: 397 (10.287%)<br>• family: 63 (1.632%)<br>• order: 38 (0.984%)<br>• class: 31 (0.803%)<br>• phylum: 160 (4.146%)<br>• superkingdom: 347 (8.991%)<br>• root: 547 (14.174%)	• Desulfotomaculum putei [taxid 74701]: 6 (0.155%)<br>• Desulfotomaculum ferrireducens [taxid 1833852]: 4 (0.103%)<br>• Desulfotomaculum ruminis [taxid 1564]: 3 (0.077%)<br>• Desulfotomaculum reducens [taxid 59610]: 3 (0.077%)<br>• Desulfotomaculum hydrothermale [taxid 412895]: 2 (0.051%)<br>• Lupinus albus [taxid 3870]: 2 (0.051%)<br>• Desulfotomaculum profundi [taxid 1383067]: 2 (0.051%)<br>• Erysipelatoclostridium ramosum [taxid 1547]: 1 (0.025%)<br>• other: 22 (0.57%)
Benchmark OTU ID: `CP000612-_Firmicutes`<br>OTU taxon: Desulfotomaculum reducens MI-1 [taxid 349161]<br>Expected: Desulfotomaculum reducens [taxid 59610] (species)<br>Number of reads: 5004<br>Number of identified reads: 4945 (98.82%)	• **species: 3234 (64.628%)**<br>• genus: 290 (5.795%)<br>• family: 42 (0.839%)<br>• order: 51 (1.019%)<br>• class: 17 (0.339%)<br>• phylum: 184 (3.677%)<br>• superkingdom: 421 (8.413%)<br>• root: 701 (14.008%)	• Desulfotomaculum aeronauticum [taxid 53343]: 5 (0.099%)<br>• Lupinus albus [taxid 3870]: 3 (0.059%)<br>• Desulfotomaculum ferrireducens [taxid 1833852]: 3 (0.059%)<br>• Pseudoflavonifractor capillosus [taxid 106588]: 2 (0.039%)<br>• Desulfotomaculum hydrothermale [taxid 412895]: 2 (0.039%)<br>• Megasphaera cerevisiae [taxid 39029]: 1 (0.019%)<br>• Desulfosporosinus sp. Tol-M [taxid 1536651]: 1 (0.019%)<br>• Herbinix luporum [taxid 1679721]: 1 (0.019%)<br>• other: 18 (0.359%)

Operational Taxonomic Unit (OTU)	Correct identifications	Wrong or overspecific identifications at species rank
Benchmark OTU ID: `FO203522-_Proteobacteria`<br>OTU taxon: Desulfovibrio hydrothermalis AM13 = DSM 14728 [taxid 1121451]<br>Expected: Maridesulfovibrio hydrothermalis [taxid 191026] (species)<br>Number of reads: 7447<br>Number of identified reads: 7419 (99.624%)	• **species: 5008 (67.248%)**<br>• genus: 83 (1.114%)<br>• family: 346 (4.646%)<br>• order: 38 (0.51%)<br>• class: 78 (1.047%)<br>• phylum: 197 (2.645%)<br>• superkingdom: 699 (9.386%)<br>• root: 967 (12.985%)	• Desulfovibrio gilichinskyi [taxid 1519643]: 3 (0.04%)<br>• Maridesulfovibrio ferrireducens [taxid 246191]: 2 (0.026%)<br>• Desulfovibrio senegalensis [taxid 1721087]: 2 (0.026%)<br>• Maridesulfovibrio salexigens [taxid 880]: 2 (0.026%)<br>• Marinobacter hydrocarbonoclasticus [taxid 2743]: 1 (0.013%)<br>• Acetobacter orientalis [taxid 146474]: 1 (0.013%)<br>• Chryseolinea serpens [taxid 947013]: 1 (0.013%)<br>• Streptomyces cinnamoneus [taxid 53446]: 1 (0.013%)<br>• other: 18 (0.241%)
Benchmark OTU ID: `CP001140-_Crenarchaeota`<br>OTU taxon: Desulfurococcus amylolyticus 1221n [taxid 490899]<br>Expected: Desulfurococcus amylolyticus [taxid 94694] (species)<br>Number of reads: 2538<br>Number of identified reads: 2483 (97.832%)	• **species: 1561 (61.505%)**<br>• genus: 43 (1.694%)<br>• family: 52 (2.048%)<br>• order: 1 (0.039%)<br>• class: 34 (1.339%)<br>• phylum: 0 (0.0%)<br>• superkingdom: 30 (1.182%)<br>• root: 731 (28.802%)	• Sulfolobus acidocaldarius [taxid 2285]: 2 (0.078%)<br>• Thermogladius calderae [taxid 1200300]: 2 (0.078%)<br>• Desulfurococcus mucosus [taxid 2275]: 1 (0.039%)<br>• Thermoprotei archaeon [taxid 2250277]: 1 (0.039%)<br>• Alistipes indistinctus [taxid 626932]: 1 (0.039%)<br>• Methanocaldococcus villosus [taxid 667126]: 1 (0.039%)<br>• Rhipicephalus pulchellus [taxid 72859]: 1 (0.039%)
Benchmark OTU ID: `CP003321-_Crenarchaeota`<br>OTU taxon: Desulfurococcus amylolyticus DSM 16532 [taxid 768672]<br>Expected: Desulfurococcus amylolyticus [taxid 94694] (species)<br>Number of reads: 2585<br>Number of identified reads: 2533 (97.988%)	• **species: 1586 (61.353%)**<br>• genus: 39 (1.508%)<br>• family: 40 (1.547%)<br>• order: 4 (0.154%)<br>• class: 36 (1.392%)<br>• phylum: 0 (0.0%)<br>• superkingdom: 30 (1.16%)<br>• root: 768 (29.709%)	• Desulfurococcus mucosus [taxid 2275]: 2 (0.077%)<br>• Thermosphaera aggregans [taxid 54254]: 1 (0.038%)<br>• Halarcobacter mediterraneus [taxid 2023153]: 1 (0.038%)<br>• Euryarchaeota archaeon [taxid 2026739]: 1 (0.038%)<br>• Desulfurococcales archaeon [taxid 2480821]: 1 (0.038%)<br>• Halococcus saccharolyticus [taxid 62319]: 1 (0.038%)<br>• Acidilobales archaeon [taxid 2268176]: 1 (0.038%)
Benchmark OTU ID: `CP002363-_Crenarchaeota`<br>OTU taxon: Desulfurococcus mucosus DSM 2162 [taxid 765177]<br>Expected: Desulfurococcus mucosus [taxid 2275] (species)<br>Number of reads: 2412<br>Number of identified reads: 2353 (97.553%)	• **species: 1400 (58.043%)**<br>• genus: 57 (2.363%)<br>• family: 40 (1.658%)<br>• order: 5 (0.207%)<br>• class: 18 (0.746%)<br>• phylum: 0 (0.0%)<br>• superkingdom: 19 (0.787%)<br>• root: 776 (32.172%)	• Desulfurococcus amylolyticus [taxid 94694]: 2 (0.082%)<br>• Fasciola hepatica [taxid 6192]: 1 (0.041%)<br>• Candidatus Woesearchaeota archaeon [taxid 2026803]: 1 (0.041%)<br>• Candidatus Korarchaeota archaeon [taxid 2056630]: 1 (0.041%)<br>• Staphylococcus simiae [taxid 308354]: 1 (0.041%)<br>• Minutocellus polymorphus [taxid 265543]: 1 (0.041%)
Benchmark OTU ID: `CP000513-_Proteobacteria`<br>OTU taxon: Dichelobacter nodosus VCS1703A [taxid 246195]<br>Expected: Dichelobacter nodosus [taxid 870] (species)<br>Number of reads: 2242<br>Number of identified reads: 2232 (99.553%)	• **species: 1650 (73.595%)**<br>• genus: 0 (0.0%)<br>• family: 18 (0.802%)<br>• order: 0 (0.0%)<br>• class: 79 (3.523%)<br>• phylum: 93 (4.148%)<br>• superkingdom: 120 (5.352%)<br>• root: 271 (12.087%)	• Suttonella indologenes [taxid 13276]: 2 (0.089%)<br>• Haemophilus parainfluenzae [taxid 729]: 1 (0.044%)<br>• Lacticaseibacillus saniviri [taxid 931533]: 1 (0.044%)<br>• Nitrosomonas ureae [taxid 44577]: 1 (0.044%)<br>• Chiloscyllium punctatum [taxid 137246]: 1 (0.044%)<br>• Porphyromonas gingivalis [taxid 837]: 1 (0.044%)<br>• Neisseria lactamica [taxid 486]: 1 (0.044%)<br>• Neisseria cinerea [taxid 483]: 1 (0.044%)

Operational Taxonomic Unit (OTU)	Correct identifications	Wrong or overspecific identifications at species rank
Benchmark OTU ID: `CP002038-_Proteobacteria`<br>OTU taxon: Dickeya dadantii 3937 [taxid 198628]<br>Expected: Dickeya dadantii [taxid 204038] (species)<br>Number of reads: 10191<br>Number of identified reads: 10162 (99.715%)	• species: 2038 (19.998%)<br>• **genus: 3609 (35.413%)**<br>• family: 386 (3.787%)<br>• order: 1937 (19.006%)<br>• class: 442 (4.337%)<br>• phylum: 306 (3.002%)<br>• superkingdom: 357 (3.503%)<br>• root: 1072 (10.519%)	• Dickeya solani [taxid 1089444]: 42 (0.412%)<br>• Salmonella enterica [taxid 28901]: 25 (0.245%)<br>• Dickeya zeae [taxid 204042]: 25 (0.245%)<br>• Dickeya undicola [taxid 1577887]: 17 (0.166%)<br>• Escherichia coli [taxid 562]: 15 (0.147%)<br>• Dickeya aquatica [taxid 1401087]: 15 (0.147%)<br>• Dickeya dianthicola [taxid 204039]: 14 (0.137%)<br>• Dickeya paradisiaca [taxid 69223]: 14 (0.137%)<br>• other: 112 (1.099%)
Benchmark OTU ID: `CP001654-_Proteobacteria`<br>OTU taxon: Dickeya paradisiaca Ech703 [taxid 579405]<br>Expected: Dickeya paradisiaca [taxid 69223] (species)<br>Number of reads: 9645<br>Number of identified reads: 9612 (99.657%)	• **species: 4643 (48.138%)**<br>• genus: 442 (4.582%)<br>• family: 354 (3.67%)<br>• order: 1945 (20.165%)<br>• class: 430 (4.458%)<br>• phylum: 301 (3.12%)<br>• superkingdom: 331 (3.431%)<br>• root: 1160 (12.026%)	• Salmonella enterica [taxid 28901]: 31 (0.321%)<br>• Escherichia coli [taxid 562]: 14 (0.145%)<br>• Klebsiella pneumoniae [taxid 573]: 5 (0.051%)<br>• Izhakiella capsodis [taxid 1367852]: 4 (0.041%)<br>• Dickeya aquatica [taxid 1401087]: 3 (0.031%)<br>• Dickeya zeae [taxid 204042]: 3 (0.031%)<br>• Buchnera aphidicola [taxid 9]: 3 (0.031%)<br>• Dickeya chrysanthemi [taxid 556]: 3 (0.031%)<br>• other: 74 (0.767%)
Benchmark OTU ID: `CP001836-_Proteobacteria`<br>OTU taxon: Dickeya zeae Ech586 [taxid 590409]<br>Expected: Dickeya zeae [taxid 204042] (species)<br>Number of reads: 9957<br>Number of identified reads: 9920 (99.628%)	• **species: 3228 (32.419%)**<br>• genus: 2025 (20.337%)<br>• family: 432 (4.338%)<br>• order: 2019 (20.277%)<br>• class: 444 (4.459%)<br>• phylum: 277 (2.781%)<br>• superkingdom: 340 (3.414%)<br>• root: 1151 (11.559%)	• Salmonella enterica [taxid 28901]: 28 (0.281%)<br>• Dickeya dadantii [taxid 204038]: 19 (0.19%)<br>• Escherichia coli [taxid 562]: 15 (0.15%)<br>• Dickeya aquatica [taxid 1401087]: 11 (0.11%)<br>• Dickeya solani [taxid 1089444]: 9 (0.09%)<br>• Dickeya dianthicola [taxid 204039]: 9 (0.09%)<br>• Dickeya poaceiphila [taxid 568768]: 4 (0.04%)<br>• Dickeya paradisiaca [taxid 69223]: 3 (0.03%)<br>• other: 91 (0.913%)
Benchmark OTU ID: `CP002528-_Bacteroidetes`<br>OTU taxon: Dokdonia sp. 4H-3-7-5 [taxid 983548]<br>Expected: Dokdonia [taxid 326319] (genus)<br>Number of reads: 21952<br>Number of identified reads: 21757 (99.111%)	• **genus: 15609 (71.105%)**<br>• family: 2429 (11.065%)<br>• order: 302 (1.375%)<br>• class: 11 (0.05%)<br>• phylum: 755 (3.439%)<br>• superkingdom: 1001 (4.559%)<br>• root: 1642 (7.479%)	• Dokdonia donghaensis [taxid 326320]: 36 (0.163%)<br>• Dokdonia sinensis [taxid 2479847]: 16 (0.072%)<br>• Dokdonia pacifica [taxid 1627892]: 5 (0.022%)<br>• Capnocytophaga canimorsus [taxid 28188]: 3 (0.013%)<br>• Aequorivita antarctica [taxid 153266]: 3 (0.013%)<br>• Leeuwenhoekiella aequorea [taxid 283736]: 2 (0.009%)<br>• Candidatus Hakubanella thermoalkaliphilus [taxid 2754717]: 2 (0.009%)<br>• other: 63 (0.286%)

Operational Taxonomic Unit (OTU)	Correct identifications	Wrong or overspecific identifications at species rank
Benchmark OTU ID: `CP003346-_Bacteroidetes`<br>OTU taxon: Echinicola vietnamensis DSM 17526 [taxid 926556]<br>Expected: Echinicola vietnamensis [taxid 390884] (species)<br>Number of reads: 38810<br>Number of identified reads: 38593 (99.44%)	• **species: 21983 (56.642%)**<br>• genus: 4798 (12.362%)<br>• family: 1030 (2.653%)<br>• order: 850 (2.19%)<br>• class: 1 (0.002%)<br>• phylum: 2323 (5.985%)<br>• superkingdom: 1817 (4.681%)<br>• root: 5771 (14.869%)	• Echinicola strongylocentroti [taxid 1795355]: 64 (0.164%)<br>• Cyclobacterium lianum [taxid 388280]: 6 (0.015%)<br>• Marivirga sericea [taxid 1028]: 4 (0.01%)<br>• Diaphanoeca grandis [taxid 28014]: 4 (0.01%)<br>• Varibaculum cambriense [taxid 184870]: 4 (0.01%)<br>• Belliella baltica [taxid 232259]: 4 (0.01%)<br>• Mariniradius saccharolyticus [taxid 1245591]: 3 (0.007%)<br>• Flammeovirga yaeyamensis [taxid 367791]: 3 (0.007%)<br>• other: 110 (0.283%)
Benchmark OTU ID: `CP001600-_Proteobacteria`<br>OTU taxon: Edwardsiella ictaluri 93-146 [taxid 634503]<br>Expected: Edwardsiella ictaluri [taxid 67780] (species)<br>Number of reads: 7694<br>Number of identified reads: 7655 (99.493%)	• **species: 2420 (31.453%)**<br>• genus: 1773 (23.043%)<br>• family: 21 (0.272%)<br>• order: 1720 (22.355%)<br>• class: 368 (4.782%)<br>• phylum: 185 (2.404%)<br>• superkingdom: 245 (3.184%)<br>• root: 918 (11.931%)	• Salmonella enterica [taxid 28901]: 35 (0.454%)<br>• Edwardsiella tarda [taxid 636]: 19 (0.246%)<br>• Escherichia coli [taxid 562]: 17 (0.22%)<br>• Edwardsiella anguillarum [taxid 1821960]: 8 (0.103%)<br>• Edwardsiella hoshinae [taxid 93378]: 6 (0.077%)<br>• Edwardsiella piscicida [taxid 1263550]: 5 (0.064%)<br>• Klebsiella pneumoniae [taxid 573]: 4 (0.051%)<br>• Xenorhabdus bovienii [taxid 40576]: 4 (0.051%)<br>• other: 65 (0.844%)
Benchmark OTU ID: `CP004141-_Proteobacteria`<br>OTU taxon: Edwardsiella piscicida C07-087 [taxid 1288122]<br>Expected: Edwardsiella piscicida [taxid 1263550] (species)<br>Number of reads: 7794<br>Number of identified reads: 7760 (99.563%)	• species: 56 (0.718%)<br>• **genus: 4012 (51.475%)**<br>• family: 15 (0.192%)<br>• order: 1927 (24.724%)<br>• class: 382 (4.901%)<br>• phylum: 174 (2.232%)<br>• superkingdom: 288 (3.695%)<br>• root: 897 (11.508%)	• **Edwardsiella tarda [taxid 636]: 170 (2.181%)**<br>• Salmonella enterica [taxid 28901]: 57 (0.731%)<br>• Edwardsiella anguillarum [taxid 1821960]: 23 (0.295%)<br>• Edwardsiella ictaluri [taxid 67780]: 21 (0.269%)<br>• Escherichia coli [taxid 562]: 18 (0.23%)<br>• Edwardsiella hoshinae [taxid 93378]: 12 (0.153%)<br>• Klebsiella aerogenes [taxid 548]: 4 (0.051%)<br>• Serratia symbiotica [taxid 138074]: 4 (0.051%)<br>• other: 89 (1.141%)
Benchmark OTU ID: `CP001135-_Proteobacteria`<br>OTU taxon: Edwardsiella tarda EIB202 [taxid 498217]<br>Expected: Edwardsiella tarda [taxid 636] (species)<br>Number of reads: 7577<br>Number of identified reads: 7547 (99.604%)	• species: 251 (3.312%)<br>• **genus: 3966 (52.342%)**<br>• family: 22 (0.29%)<br>• order: 1798 (23.729%)<br>• class: 384 (5.067%)<br>• phylum: 165 (2.177%)<br>• superkingdom: 236 (3.114%)<br>• root: 720 (9.502%)	• Edwardsiella piscicida [taxid 1263550]: 52 (0.686%)<br>• Salmonella enterica [taxid 28901]: 36 (0.475%)<br>• Edwardsiella anguillarum [taxid 1821960]: 25 (0.329%)<br>• Edwardsiella ictaluri [taxid 67780]: 20 (0.263%)<br>• Escherichia coli [taxid 562]: 16 (0.211%)<br>• Edwardsiella hoshinae [taxid 93378]: 9 (0.118%)<br>• Serratia symbiotica [taxid 138074]: 5 (0.065%)<br>• Klebsiella pneumoniae [taxid 573]: 3 (0.039%)<br>• other: 49 (0.646%)

Operational Taxonomic Unit (OTU)	Correct identifications	Wrong or overspecific identifications at species rank
Benchmark OTU ID: `AP012211-_Actinobacteria`<br>OTU taxon: Eggerthella sp. YY7918 [taxid 502558]<br>Expected: Eggerthella [taxid 84111] (genus)<br>Number of reads: 12903<br>Number of identified reads: 12883 (99.844%)	• **genus: 7887 (61.125%)**<br>• family: 1280 (9.92%)<br>• order: 5 (0.038%)<br>• class: 626 (4.851%)<br>• phylum: 179 (1.387%)<br>• superkingdom: 1195 (9.261%)<br>• root: 1704 (13.206%)	• Eggerthella sinensis [taxid 242230]: 6 (0.046%)<br>• Enteroscipio rubneri [taxid 2070686]: 3 (0.023%)<br>• Adlercreutzia equolifaciens [taxid 446660]: 3 (0.023%)<br>• Paraeggerthella hongkongensis [taxid 230658]: 3 (0.023%)<br>• Collinsella aerofaciens [taxid 74426]: 2 (0.015%)<br>• Slackia equolifaciens [taxid 498718]: 2 (0.015%)<br>• Eggerthella lenta [taxid 84112]: 2 (0.015%)<br>• other: 28 (0.217%)
Benchmark OTU ID: `CR767821-_Pathogens`<br>OTU taxon: Ehrlichia ruminantium str. Welgevonden [taxid 254945]<br>Expected: Ehrlichia ruminantium [taxid 779] (species)<br>Number of reads: 2577<br>Number of identified reads: 2243 (87.039%)	• **species: 1435 (55.684%)**<br>• genus: 78 (3.026%)<br>• family: 22 (0.853%)<br>• order: 4 (0.155%)<br>• class: 25 (0.97%)<br>• phylum: 28 (1.086%)<br>• superkingdom: 75 (2.91%)<br>• root: 574 (22.273%)	• Ehrlichia muris [taxid 35795]: 1 (0.038%)<br>• Elusimicrobium minutum [taxid 423605]: 1 (0.038%)<br>• Orientia chuto [taxid 911112]: 1 (0.038%)<br>• Varibaculum cambriense [taxid 184870]: 1 (0.038%)
Benchmark OTU ID: `CR925678-_Pathogens`<br>OTU taxon: Ehrlichia ruminantium str. Welgevonden [taxid 254945]<br>Expected: Ehrlichia ruminantium [taxid 779] (species)<br>Number of reads: 2568<br>Number of identified reads: 2263 (88.123%)	• **species: 1441 (56.113%)**<br>• genus: 85 (3.309%)<br>• family: 31 (1.207%)<br>• order: 4 (0.155%)<br>• class: 27 (1.051%)<br>• phylum: 18 (0.7%)<br>• superkingdom: 68 (2.647%)<br>• root: 581 (22.624%)	• Aphanomyces euteiches [taxid 100861]: 1 (0.038%)<br>• Hepatocystis sp. ex Piliocolobus tephrosceles [taxid 2600580]: 1 (0.038%)<br>• Synechococcus virus STIM4 [taxid 2734148]: 1 (0.038%)<br>• Candidatus Neoehrlichia lotoris [taxid 467750]: 1 (0.038%)<br>• Aerococcus urinae [taxid 1376]: 1 (0.038%)<br>• Hevea brasiliensis [taxid 3981]: 1 (0.038%)
Benchmark OTU ID: `CP001055-_Elusimicrobia`<br>OTU taxon: Elusimicrobium minutum Pei191 [taxid 445932]<br>Expected: Elusimicrobium minutum [taxid 423605] (species)<br>Number of reads: 61210<br>Number of identified reads: 60563 (98.942%)	• **species: 46873 (76.577%)**<br>• genus: 77 (0.125%)<br>• family: 0 (0.0%)<br>• order: 0 (0.0%)<br>• class: 4 (0.006%)<br>• phylum: 412 (0.673%)<br>• superkingdom: 4973 (8.124%)<br>• root: 8180 (13.363%)	• Lacticaseibacillus paracasei [taxid 1597]: 3 (0.004%)<br>• Cryptomonas curvata [taxid 233186]: 2 (0.003%)<br>• [Clostridium] populeti [taxid 37658]: 2 (0.003%)<br>• Escherichia coli [taxid 562]: 2 (0.003%)<br>• Klebsormidium nitens [taxid 105231]: 2 (0.003%)<br>• Hordeum vulgare [taxid 4513]: 2 (0.003%)<br>• Rhodovulum sulfidophilum [taxid 35806]: 2 (0.003%)<br>• Saccharopolyspora erythraea [taxid 1836]: 1 (0.001%)<br>• other: 68 (0.111%)
Benchmark OTU ID: `CP003678-_Proteobacteria`<br>OTU taxon: Enterobacter cloacae subsp. dissolvens SDM [taxid 1104326]<br>Expected: Enterobacter cloacae [taxid 550] (species)<br>Number of reads: 10294<br>Number of identified reads: 10270 (99.766%)	• species: 949 (9.218%)<br>• genus: 560 (5.44%)<br>• **family: 6322 (61.414%)**<br>• order: 784 (7.616%)<br>• class: 285 (2.768%)<br>• phylum: 229 (2.224%)<br>• superkingdom: 296 (2.875%)<br>• root: 836 (8.121%)	• Salmonella enterica [taxid 28901]: 149 (1.447%)<br>• Escherichia coli [taxid 562]: 108 (1.049%)<br>• Klebsiella pneumoniae [taxid 573]: 51 (0.495%)<br>• Enterobacter ludwigii [taxid 299767]: 45 (0.437%)<br>• Enterobacter hormaechei [taxid 158836]: 35 (0.34%)<br>• Enterobacter asburiae [taxid 61645]: 22 (0.213%)<br>• Enterobacter cancerogenus [taxid 69218]: 14 (0.136%)<br>• Lelliottia nimipressuralis [taxid 69220]: 6 (0.058%)<br>• other: 176 (1.709%)

Operational Taxonomic Unit (OTU)	Correct identifications	Wrong or overspecific identifications at species rank
Benchmark OTU ID: `CP003737-_Proteobacteria`<br>OTU taxon: Enterobacter kobei [taxid 208224]<br>Expected: Enterobacter kobei [taxid 208224] (species)<br>Number of reads: 9751<br>Number of identified reads: 9726 (99.743%)	• species: 26 (0.266%)<br>• genus: 533 (5.466%)<br>• **family: 6348 (65.101%)**<br>• order: 1268 (13.003%)<br>• class: 282 (2.892%)<br>• phylum: 194 (1.989%)<br>• superkingdom: 270 (2.768%)<br>• root: 791 (8.111%)	• Salmonella enterica [taxid 28901]: 188 (1.928%)<br>• Escherichia coli [taxid 562]: 115 (1.179%)<br>• Enterobacter cloacae [taxid 550]: 53 (0.543%)<br>• Enterobacter ludwigii [taxid 299767]: 41 (0.42%)<br>• Enterobacter hormaechei [taxid 158836]: 31 (0.317%)<br>• Enterobacter asburiae [taxid 61645]: 29 (0.297%)<br>• Klebsiella pneumoniae [taxid 573]: 21 (0.215%)<br>• Enterobacter cancerogenus [taxid 69218]: 19 (0.194%)<br>• other: 219 (2.245%)
Benchmark OTU ID: `CP002886-_Proteobacteria`<br>OTU taxon: Enterobacter ludwigii [taxid 299767]<br>Expected: Enterobacter ludwigii [taxid 299767] (species)<br>Number of reads: 9768<br>Number of identified reads: 9754 (99.856%)	• species: 603 (6.173%)<br>• genus: 418 (4.279%)<br>• **family: 6511 (66.656%)**<br>• order: 738 (7.555%)<br>• class: 267 (2.733%)<br>• phylum: 192 (1.965%)<br>• superkingdom: 250 (2.559%)<br>• root: 772 (7.903%)	• Salmonella enterica [taxid 28901]: 166 (1.699%)<br>• Escherichia coli [taxid 562]: 86 (0.88%)<br>• Enterobacter cloacae [taxid 550]: 32 (0.327%)<br>• Klebsiella pneumoniae [taxid 573]: 19 (0.194%)<br>• Enterobacter hormaechei [taxid 158836]: 17 (0.174%)<br>• Enterobacter asburiae [taxid 61645]: 14 (0.143%)<br>• Enterobacter cancerogenus [taxid 69218]: 7 (0.071%)<br>• Lelliottia amnigena [taxid 61646]: 6 (0.061%)<br>• other: 157 (1.607%)
Benchmark OTU ID: `CP000653-_Proteobacteria`<br>OTU taxon: Enterobacter sp. 638 [taxid 399742]<br>Expected: Enterobacter [taxid 547] (genus)<br>Number of reads: 9283<br>Number of identified reads: 9263 (99.784%)	• genus: 1919 (20.672%)<br>• **family: 5005 (53.915%)**<br>• order: 761 (8.197%)<br>• class: 311 (3.35%)<br>• phylum: 166 (1.788%)<br>• superkingdom: 241 (2.596%)<br>• root: 848 (9.134%)	• Salmonella enterica [taxid 28901]: 164 (1.766%)<br>• Escherichia coli [taxid 562]: 91 (0.98%)<br>• Lelliottia amnigena [taxid 61646]: 26 (0.28%)<br>• Enterobacter ludwigii [taxid 299767]: 20 (0.215%)<br>• Klebsiella pneumoniae [taxid 573]: 20 (0.215%)<br>• Enterobacter hormaechei [taxid 158836]: 15 (0.161%)<br>• Enterobacter cloacae [taxid 550]: 11 (0.118%)<br>• other: 160 (1.723%)
Benchmark OTU ID: `CP003938-_Proteobacteria`<br>OTU taxon: Enterobacteriaceae bacterium strain FGI 57 [taxid 693444]<br>Expected: Enterobacteriaceae [taxid 543] (family)<br>Number of reads: 9831<br>Number of identified reads: 9804 (99.725%)	• **family: 7545 (76.747%)**<br>• order: 743 (7.557%)<br>• class: 292 (2.97%)<br>• phylum: 181 (1.841%)<br>• superkingdom: 228 (2.319%)<br>• root: 814 (8.279%)	• **Salmonella enterica [taxid 28901]: 364 (3.702%)**<br>• **Escherichia coli [taxid 562]: 217 (2.207%)**<br>• Pseudocitrobacter faecalis [taxid 1398493]: 25 (0.254%)<br>• Klebsiella pneumoniae [taxid 573]: 25 (0.254%)<br>• Citrobacter koseri [taxid 545]: 10 (0.101%)<br>• Shigella flexneri [taxid 623]: 7 (0.071%)<br>• other: 154 (1.566%)

Operational Taxonomic Unit (OTU)	Correct identifications	Wrong or overspecific identifications at species rank
Benchmark OTU ID: `FN666575-_Proteobacteria`<br>OTU taxon: Erwinia amylovora ATCC 49946 [taxid 716540]<br>Expected: Erwinia amylovora [taxid 552] (species)<br>Number of reads: 7679<br>Number of identified reads: 7648 (99.596%)	• **species: 2932 (38.182%)**<br>• genus: 1012 (13.178%)<br>• family: 441 (5.742%)<br>• order: 1646 (21.435%)<br>• class: 319 (4.154%)<br>• phylum: 132 (1.718%)<br>• superkingdom: 240 (3.125%)<br>• root: 921 (11.993%)	• Salmonella enterica [taxid 28901]: 20 (0.26%)<br>• Escherichia coli [taxid 562]: 12 (0.156%)<br>• Erwinia pyrifoliae [taxid 79967]: 10 (0.13%)<br>• Candidatus Erwinia dacicola [taxid 252393]: 5 (0.065%)<br>• Erwinia tracheiphila [taxid 65700]: 5 (0.065%)<br>• Erwinia piriflorinigrans [taxid 665097]: 5 (0.065%)<br>• Pantoea sp. Aalb [taxid 2576762]: 4 (0.052%)<br>• Erwinia tasmaniensis [taxid 338565]: 3 (0.039%)<br>• other: 78 (1.015%)
Benchmark OTU ID: `FN434113-_Proteobacteria`<br>OTU taxon: Erwinia amylovora CFBP1430 [taxid 665029]<br>Expected: Erwinia amylovora [taxid 552] (species)<br>Number of reads: 7678<br>Number of identified reads: 7664 (99.817%)	• **species: 2885 (37.574%)**<br>• genus: 981 (12.776%)<br>• family: 411 (5.352%)<br>• order: 1679 (21.867%)<br>• class: 318 (4.141%)<br>• phylum: 157 (2.044%)<br>• superkingdom: 246 (3.203%)<br>• root: 981 (12.776%)	• Salmonella enterica [taxid 28901]: 19 (0.247%)<br>• Escherichia coli [taxid 562]: 13 (0.169%)<br>• Erwinia tasmaniensis [taxid 338565]: 9 (0.117%)<br>• Erwinia pyrifoliae [taxid 79967]: 7 (0.091%)<br>• Erwinia piriflorinigrans [taxid 665097]: 7 (0.091%)<br>• Candidatus Erwinia haradaeae [taxid 1922217]: 4 (0.052%)<br>• Serratia symbiotica [taxid 138074]: 3 (0.039%)<br>• Citrobacter koseri [taxid 545]: 3 (0.039%)<br>• other: 61 (0.794%)
Benchmark OTU ID: `FN392235-_Proteobacteria`<br>OTU taxon: Erwinia pyrifoliae DSM 12163 [taxid 644651]<br>Expected: Erwinia pyrifoliae [taxid 79967] (species)<br>Number of reads: 7866<br>Number of identified reads: 7831 (99.555%)	• species: 1216 (15.458%)<br>• **genus: 2785 (35.405%)**<br>• family: 450 (5.72%)<br>• order: 1726 (21.942%)<br>• class: 322 (4.093%)<br>• phylum: 151 (1.919%)<br>• superkingdom: 240 (3.051%)<br>• root: 937 (11.912%)	• Salmonella enterica [taxid 28901]: 29 (0.368%)<br>• Erwinia amylovora [taxid 552]: 19 (0.241%)<br>• Escherichia coli [taxid 562]: 16 (0.203%)<br>• Erwinia piriflorinigrans [taxid 665097]: 15 (0.19%)<br>• Erwinia tasmaniensis [taxid 338565]: 14 (0.177%)<br>• Serratia symbiotica [taxid 138074]: 5 (0.063%)<br>• Erwinia tracheiphila [taxid 65700]: 4 (0.05%)<br>• Candidatus Erwinia dacicola [taxid 252393]: 3 (0.038%)<br>• other: 76 (0.966%)
Benchmark OTU ID: `FP236842-_Proteobacteria`<br>OTU taxon: Erwinia pyrifoliae Ep1/96 [taxid 634499]<br>Expected: Erwinia pyrifoliae [taxid 79967] (species)<br>Number of reads: 8175<br>Number of identified reads: 8135 (99.51%)	• species: 1312 (16.048%)<br>• **genus: 2918 (35.694%)**<br>• family: 459 (5.614%)<br>• order: 1774 (21.7%)<br>• class: 310 (3.792%)<br>• phylum: 173 (2.116%)<br>• superkingdom: 212 (2.593%)<br>• root: 971 (11.877%)	• Erwinia amylovora [taxid 552]: 27 (0.33%)<br>• Salmonella enterica [taxid 28901]: 25 (0.305%)<br>• Erwinia piriflorinigrans [taxid 665097]: 21 (0.256%)<br>• Escherichia coli [taxid 562]: 17 (0.207%)<br>• Erwinia tasmaniensis [taxid 338565]: 14 (0.171%)<br>• Candidatus Erwinia dacicola [taxid 252393]: 5 (0.061%)<br>• Klebsiella pneumoniae [taxid 573]: 5 (0.061%)<br>• Erwinia rhapontici [taxid 55212]: 5 (0.061%)<br>• other: 110 (1.345%)

Operational Taxonomic Unit (OTU)	Correct identifications	Wrong or overspecific identifications at species rank
Benchmark OTU ID: `CU468135-_Proteobacteria`<br>OTU taxon: Erwinia tasmaniensis Et1/99 [taxid 465817]<br>Expected: Erwinia tasmaniensis [taxid 338565] (species)<br>Number of reads: 7854<br>Number of identified reads: 7825 (99.63%)	• **species: 2680 (34.122%)**<br>• genus: 1070 (13.623%)<br>• family: 490 (6.238%)<br>• order: 1825 (23.236%)<br>• class: 343 (4.367%)<br>• phylum: 197 (2.508%)<br>• superkingdom: 231 (2.941%)<br>• root: 979 (12.464%)	• Salmonella enterica [taxid 28901]: 28 (0.356%)<br>• Escherichia coli [taxid 562]: 19 (0.241%)<br>• Erwinia piriflorinigrans [taxid 665097]: 15 (0.19%)<br>• Candidatus Erwinia dacicola [taxid 252393]: 9 (0.114%)<br>• Erwinia amylovora [taxid 552]: 6 (0.076%)<br>• Erwinia pyrifoliae [taxid 79967]: 6 (0.076%)<br>• Serratia marcescens [taxid 615]: 5 (0.063%)<br>• Erwinia tracheiphila [taxid 65700]: 5 (0.063%)<br>• other: 75 (0.954%)
Benchmark OTU ID: `AP012027-_Firmicutes`<br>OTU taxon: Erysipelothrix rhusiopathiae str. Fujisawa [taxid 650150]<br>Expected: Erysipelothrix rhusiopathiae [taxid 1648] (species)<br>Number of reads: 2092<br>Number of identified reads: 2073 (99.091%)	• **species: 1326 (63.384%)**<br>• genus: 383 (18.307%)<br>• family: 3 (0.143%)<br>• order: 0 (0.0%)<br>• class: 0 (0.0%)<br>• phylum: 95 (4.541%)<br>• superkingdom: 96 (4.588%)<br>• root: 166 (7.934%)	• Erysipelothrix larvae [taxid 1514105]: 2 (0.095%)<br>• Chlamydia pneumoniae [taxid 83558]: 1 (0.047%)<br>• Erysipelothrix piscisicarius [taxid 2485784]: 1 (0.047%)<br>• Candidatus Microthrix parvicella [taxid 41950]: 1 (0.047%)<br>• Helicobacter pylori [taxid 210]: 1 (0.047%)<br>• Aegilops tauschii [taxid 37682]: 1 (0.047%)<br>• Beta vulgaris [taxid 161934]: 1 (0.047%)<br>• Amedibacillus dolichus [taxid 31971]: 1 (0.047%)
Benchmark OTU ID: `CP004009-_Pathogens`<br>OTU taxon: Escherichia coli APEC O78 [taxid 1274814]<br>Expected: Escherichia coli [taxid 562] (species)<br>Number of reads: 10782<br>Number of identified reads: 10752 (99.721%)	• species: 2043 (18.948%)<br>• genus: 177 (1.641%)<br>• **family: 6827 (63.318%)**<br>• order: 451 (4.182%)<br>• class: 258 (2.392%)<br>• phylum: 142 (1.317%)<br>• superkingdom: 244 (2.263%)<br>• root: 607 (5.629%)	• Salmonella enterica [taxid 28901]: 183 (1.697%)<br>• Shigella sonnei [taxid 624]: 87 (0.806%)<br>• Shigella flexneri [taxid 623]: 69 (0.639%)<br>• Shigella dysenteriae [taxid 622]: 21 (0.194%)<br>• Klebsiella pneumoniae [taxid 573]: 15 (0.139%)<br>• Escherichia fergusonii [taxid 564]: 13 (0.12%)<br>• Shigella boydii [taxid 621]: 9 (0.083%)<br>• Citrobacter amalonaticus [taxid 35703]: 7 (0.064%)<br>• other: 110 (1.02%)
Benchmark OTU ID: `CP000946-_Proteobacteria`<br>OTU taxon: Escherichia coli ATCC 8739 [taxid 481805]<br>Expected: Escherichia coli [taxid 562] (species)<br>Number of reads: 9795<br>Number of identified reads: 9772 (99.765%)	• species: 1978 (20.193%)<br>• genus: 180 (1.837%)<br>• **family: 6035 (61.613%)**<br>• order: 418 (4.267%)<br>• class: 239 (2.44%)<br>• phylum: 124 (1.265%)<br>• superkingdom: 288 (2.94%)<br>• root: 503 (5.135%)	• Salmonella enterica [taxid 28901]: 140 (1.429%)<br>• Shigella sonnei [taxid 624]: 73 (0.745%)<br>• Shigella flexneri [taxid 623]: 61 (0.622%)<br>• Klebsiella pneumoniae [taxid 573]: 22 (0.224%)<br>• Shigella dysenteriae [taxid 622]: 16 (0.163%)<br>• Shigella boydii [taxid 621]: 9 (0.091%)<br>• Citrobacter freundii [taxid 546]: 7 (0.071%)<br>• Enterobacter cloacae [taxid 550]: 4 (0.04%)<br>• other: 104 (1.061%)

Operational Taxonomic Unit (OTU)	Correct identifications	Wrong or overspecific identifications at species rank
Benchmark OTU ID: `FN649414-_Pathogens`<br>OTU taxon: Escherichia coli ETEC H10407 [taxid 316401]<br>Expected: Escherichia coli [taxid 562] (species)<br>Number of reads: 11670<br>Number of identified reads: 11636 (99.708%)	• species: 2568 (22.005%)<br>• genus: 236 (2.022%)<br>• **family: 6938 (59.451%)**<br>• order: 458 (3.924%)<br>• class: 241 (2.065%)<br>• phylum: 172 (1.473%)<br>• superkingdom: 272 (2.33%)<br>• root: 746 (6.392%)	• Salmonella enterica [taxid 28901]: 167 (1.431%)<br>• Shigella sonnei [taxid 624]: 81 (0.694%)<br>• Shigella flexneri [taxid 623]: 50 (0.428%)<br>• Shigella dysenteriae [taxid 622]: 21 (0.179%)<br>• Klebsiella pneumoniae [taxid 573]: 17 (0.145%)<br>• Shigella boydii [taxid 621]: 11 (0.094%)<br>• Citrobacter koseri [taxid 545]: 7 (0.059%)<br>• Klebsiella oxytoca [taxid 571]: 6 (0.051%)<br>• other: 123 (1.053%)
Benchmark OTU ID: `CP002516-_Pathogens`<br>OTU taxon: Escherichia coli KO11FL [taxid 595495]<br>Expected: Escherichia coli [taxid 562] (species)<br>Number of reads: 11086<br>Number of identified reads: 11053 (99.702%)	• species: 2246 (20.259%)<br>• genus: 212 (1.912%)<br>• **family: 6723 (60.644%)**<br>• order: 465 (4.194%)<br>• class: 238 (2.146%)<br>• phylum: 207 (1.867%)<br>• superkingdom: 249 (2.246%)<br>• root: 702 (6.332%)	• Salmonella enterica [taxid 28901]: 146 (1.316%)<br>• Shigella sonnei [taxid 624]: 78 (0.703%)<br>• Shigella flexneri [taxid 623]: 66 (0.595%)<br>• Shigella dysenteriae [taxid 622]: 18 (0.162%)<br>• Klebsiella pneumoniae [taxid 573]: 16 (0.144%)<br>• Shigella boydii [taxid 621]: 9 (0.081%)<br>• Escherichia fergusonii [taxid 564]: 7 (0.063%)<br>• Escherichia albertii [taxid 208962]: 7 (0.063%)<br>• other: 128 (1.154%)
Benchmark OTU ID: `CP002970-_Pathogens`<br>OTU taxon: Escherichia coli KO11FL [taxid 595495]<br>Expected: Escherichia coli [taxid 562] (species)<br>Number of reads: 11341<br>Number of identified reads: 11299 (99.629%)	• species: 2189 (19.301%)<br>• genus: 189 (1.666%)<br>• **family: 6710 (59.165%)**<br>• order: 553 (4.876%)<br>• class: 380 (3.35%)<br>• phylum: 273 (2.407%)<br>• superkingdom: 315 (2.777%)<br>• root: 680 (5.995%)	• Salmonella enterica [taxid 28901]: 141 (1.243%)<br>• Zymomonas mobilis [taxid 542]: 95 (0.837%)<br>• Shigella sonnei [taxid 624]: 86 (0.758%)<br>• Shigella flexneri [taxid 623]: 57 (0.502%)<br>• Klebsiella pneumoniae [taxid 573]: 21 (0.185%)<br>• Shigella dysenteriae [taxid 622]: 21 (0.185%)<br>• Escherichia albertii [taxid 208962]: 8 (0.07%)<br>• Escherichia fergusonii [taxid 564]: 6 (0.052%)<br>• other: 146 (1.287%)
Benchmark OTU ID: `CP003301-_Pathogens`<br>OTU taxon: Escherichia coli O104:H4 str. 2009EL-2071 [taxid 1133853]<br>Expected: Escherichia coli [taxid 562] (species)<br>Number of reads: 12067<br>Number of identified reads: 12024 (99.643%)	• species: 2667 (22.101%)<br>• genus: 194 (1.607%)<br>• **family: 7099 (58.829%)**<br>• order: 489 (4.052%)<br>• class: 260 (2.154%)<br>• phylum: 161 (1.334%)<br>• superkingdom: 274 (2.27%)<br>• root: 854 (7.077%)	• Salmonella enterica [taxid 28901]: 191 (1.582%)<br>• Shigella sonnei [taxid 624]: 81 (0.671%)<br>• Shigella flexneri [taxid 623]: 60 (0.497%)<br>• Klebsiella pneumoniae [taxid 573]: 21 (0.174%)<br>• Shigella dysenteriae [taxid 622]: 21 (0.174%)<br>• Escherichia fergusonii [taxid 564]: 11 (0.091%)<br>• Shigella boydii [taxid 621]: 9 (0.074%)<br>• Cronobacter sakazakii [taxid 28141]: 8 (0.066%)<br>• other: 133 (1.102%)

Operational Taxonomic Unit (OTU)	Correct identifications	Wrong or overspecific identifications at species rank
Benchmark OTU ID: `CP003289-_Pathogens`<br>OTU taxon: Escherichia coli O104:H4 str. 2011C-3493 [taxid 1133852]<br>Expected: Escherichia coli [taxid 562] (species)<br>Number of reads: 11969<br>Number of identified reads: 11915 (99.548%)	• species: 2530 (21.137%)<br>• genus: 170 (1.42%)<br>• **family: 7154 (59.771%)**<br>• order: 513 (4.286%)<br>• class: 269 (2.247%)<br>• phylum: 152 (1.269%)<br>• superkingdom: 301 (2.514%)<br>• root: 810 (6.767%)	• Salmonella enterica [taxid 28901]: 171 (1.428%)<br>• Shigella sonnei [taxid 624]: 90 (0.751%)<br>• Shigella flexneri [taxid 623]: 72 (0.601%)<br>• Klebsiella pneumoniae [taxid 573]: 24 (0.2%)<br>• Shigella dysenteriae [taxid 622]: 22 (0.183%)<br>• Shigella boydii [taxid 621]: 9 (0.075%)<br>• Citrobacter freundii [taxid 546]: 6 (0.05%)<br>• Escherichia albertii [taxid 208962]: 6 (0.05%)<br>• other: 112 (0.935%)
Benchmark OTU ID: `CP001164-_Pathogens`<br>OTU taxon: Escherichia coli O157:H7 str. EC4115 [taxid 444450]<br>Expected: Escherichia coli [taxid 562] (species)<br>Number of reads: 12716<br>Number of identified reads: 12677 (99.693%)	• species: 3413 (26.84%)<br>• genus: 258 (2.028%)<br>• **family: 6789 (53.389%)**<br>• order: 505 (3.971%)<br>• class: 300 (2.359%)<br>• phylum: 174 (1.368%)<br>• superkingdom: 310 (2.437%)<br>• root: 909 (7.148%)	• Salmonella enterica [taxid 28901]: 162 (1.273%)<br>• Shigella sonnei [taxid 624]: 71 (0.558%)<br>• Shigella flexneri [taxid 623]: 63 (0.495%)<br>• Shigella dysenteriae [taxid 622]: 21 (0.165%)<br>• Klebsiella pneumoniae [taxid 573]: 18 (0.141%)<br>• Shigella boydii [taxid 621]: 12 (0.094%)<br>• Citrobacter freundii [taxid 546]: 10 (0.078%)<br>• Escherichia fergusonii [taxid 564]: 6 (0.047%)<br>• other: 149 (1.171%)
Benchmark OTU ID: `CP001846-_Pathogens`<br>OTU taxon: Escherichia coli O55:H7 str. CB9615 [taxid 701177]<br>Expected: Escherichia coli [taxid 562] (species)<br>Number of reads: 12252<br>Number of identified reads: 12204 (99.608%)	• species: 3155 (25.75%)<br>• genus: 285 (2.326%)<br>• **family: 6792 (55.435%)**<br>• order: 484 (3.95%)<br>• class: 262 (2.138%)<br>• phylum: 181 (1.477%)<br>• superkingdom: 283 (2.309%)<br>• root: 753 (6.145%)	• Salmonella enterica [taxid 28901]: 181 (1.477%)<br>• Shigella sonnei [taxid 624]: 70 (0.571%)<br>• Shigella flexneri [taxid 623]: 49 (0.399%)<br>• Shigella dysenteriae [taxid 622]: 17 (0.138%)<br>• Klebsiella pneumoniae [taxid 573]: 17 (0.138%)<br>• Shigella boydii [taxid 621]: 14 (0.114%)<br>• Escherichia fergusonii [taxid 564]: 10 (0.081%)<br>• Enterobacter cloacae [taxid 550]: 9 (0.073%)<br>• other: 135 (1.101%)
Benchmark OTU ID: `CP003034-_Pathogens`<br>OTU taxon: Escherichia coli O7:K1 str. CE10 [taxid 1072459]<br>Expected: Escherichia coli [taxid 562] (species)<br>Number of reads: 12070<br>Number of identified reads: 12038 (99.734%)	• species: 2994 (24.805%)<br>• genus: 389 (3.222%)<br>• **family: 6682 (55.36%)**<br>• order: 509 (4.217%)<br>• class: 300 (2.485%)<br>• phylum: 175 (1.449%)<br>• superkingdom: 260 (2.154%)<br>• root: 720 (5.965%)	• Salmonella enterica [taxid 28901]: 150 (1.242%)<br>• Shigella sonnei [taxid 624]: 72 (0.596%)<br>• Shigella flexneri [taxid 623]: 60 (0.497%)<br>• Klebsiella pneumoniae [taxid 573]: 20 (0.165%)<br>• Shigella dysenteriae [taxid 622]: 19 (0.157%)<br>• Shigella boydii [taxid 621]: 9 (0.074%)<br>• Escherichia fergusonii [taxid 564]: 8 (0.066%)<br>• Pluralibacter gergoviae [taxid 61647]: 7 (0.057%)<br>• other: 138 (1.143%)

Operational Taxonomic Unit (OTU)	Correct identifications	Wrong or overspecific identifications at species rank
Benchmark OTU ID: `CP002729-_Pathogens`<br>OTU taxon: Escherichia coli UMNK88 [taxid 696406]<br>Expected: Escherichia coli [taxid 562] (species)<br>Number of reads: 11752<br>Number of identified reads: 11709 (99.634%)	• species: 2631 (22.387%)<br>• genus: 169 (1.438%)<br>• **family: 6941 (59.062%)**<br>• order: 460 (3.914%)<br>• class: 285 (2.425%)<br>• phylum: 154 (1.31%)<br>• superkingdom: 290 (2.467%)<br>• root: 768 (6.535%)	• Salmonella enterica [taxid 28901]: 173 (1.472%)<br>• Shigella sonnei [taxid 624]: 83 (0.706%)<br>• Shigella flexneri [taxid 623]: 60 (0.51%)<br>• Shigella dysenteriae [taxid 622]: 26 (0.221%)<br>• Klebsiella pneumoniae [taxid 573]: 23 (0.195%)<br>• Citrobacter freundii [taxid 546]: 11 (0.093%)<br>• Citrobacter amalonaticus [taxid 35703]: 6 (0.051%)<br>• Escherichia albertii [taxid 208962]: 5 (0.042%)<br>• other: 135 (1.148%)
Benchmark OTU ID: `AP012306-_Pathogens`<br>OTU taxon: Escherichia coli str. K-12 substr. MDS42 [taxid 1110693]<br>Expected: Escherichia coli [taxid 562] (species)<br>Number of reads: 8726<br>Number of identified reads: 8698 (99.679%)	• species: 1450 (16.617%)<br>• genus: 130 (1.489%)<br>• **family: 5681 (65.104%)**<br>• order: 410 (4.698%)<br>• class: 230 (2.635%)<br>• phylum: 124 (1.421%)<br>• superkingdom: 211 (2.418%)<br>• root: 459 (5.26%)	• Salmonella enterica [taxid 28901]: 139 (1.592%)<br>• Shigella sonnei [taxid 624]: 67 (0.767%)<br>• Shigella flexneri [taxid 623]: 49 (0.561%)<br>• Klebsiella pneumoniae [taxid 573]: 19 (0.217%)<br>• Shigella dysenteriae [taxid 622]: 19 (0.217%)<br>• Shigella boydii [taxid 621]: 9 (0.103%)<br>• Citrobacter amalonaticus [taxid 35703]: 6 (0.068%)<br>• Escherichia fergusonii [taxid 564]: 5 (0.057%)<br>• other: 113 (1.294%)
Benchmark OTU ID: `AP009048-_Pathogens`<br>OTU taxon: Escherichia coli str. K-12 substr. W3110 [taxid 316407]<br>Expected: Escherichia coli [taxid 562] (species)<br>Number of reads: 10402<br>Number of identified reads: 10363 (99.625%)	• species: 2026 (19.477%)<br>• genus: 161 (1.547%)<br>• **family: 6506 (62.545%)**<br>• order: 474 (4.556%)<br>• class: 300 (2.884%)<br>• phylum: 138 (1.326%)<br>• superkingdom: 227 (2.182%)<br>• root: 524 (5.037%)	• Salmonella enterica [taxid 28901]: 154 (1.48%)<br>• Shigella sonnei [taxid 624]: 87 (0.836%)<br>• Shigella flexneri [taxid 623]: 55 (0.528%)<br>• Shigella dysenteriae [taxid 622]: 24 (0.23%)<br>• Klebsiella pneumoniae [taxid 573]: 18 (0.173%)<br>• Shigella boydii [taxid 621]: 9 (0.086%)<br>• Citrobacter amalonaticus [taxid 35703]: 8 (0.076%)<br>• Citrobacter koseri [taxid 545]: 8 (0.076%)<br>• other: 129 (1.24%)
Benchmark OTU ID: `CU928158-_Proteobacteria`<br>OTU taxon: Escherichia fergusonii ATCC 35469 [taxid 585054]<br>Expected: Escherichia fergusonii [taxid 564] (species)<br>Number of reads: 9441<br>Number of identified reads: 9416 (99.735%)	• species: 1623 (17.19%)<br>• genus: 1459 (15.453%)<br>• **family: 4402 (46.626%)**<br>• order: 508 (5.38%)<br>• class: 269 (2.849%)<br>• phylum: 148 (1.567%)<br>• superkingdom: 238 (2.52%)<br>• root: 764 (8.092%)	• **Escherichia coli [taxid 562]: 647 (6.853%)**<br>• **Salmonella enterica [taxid 28901]: 241 (2.552%)**<br>• Shigella sonnei [taxid 624]: 24 (0.254%)<br>• Shigella flexneri [taxid 623]: 22 (0.233%)<br>• Shigella dysenteriae [taxid 622]: 6 (0.063%)<br>• Escherichia albertii [taxid 208962]: 4 (0.042%)<br>• Klebsiella pneumoniae [taxid 573]: 4 (0.042%)<br>• Azospirillum brasilense [taxid 192]: 4 (0.042%)<br>• other: 88 (0.932%)

Operational Taxonomic Unit (OTU)	Correct identifications	Wrong or overspecific identifications at species rank
Benchmark OTU ID: `CP002400-_Firmicutes`<br>OTU taxon: Ethanoligenens harbinense YUAN-3 [taxid 663278]<br>Expected: Ethanoligenens harbinense [taxid 253239] (species)<br>Number of reads: 4045<br>Number of identified reads: 4038 (99.826%)	• **species: 2970 (73.423%)**<br>• genus: 0 (0.0%)<br>• family: 51 (1.26%)<br>• order: 136 (3.362%)<br>• class: 5 (0.123%)<br>• phylum: 91 (2.249%)<br>• superkingdom: 255 (6.304%)<br>• root: 526 (13.003%)	• Sporolactobacillus nakayamae [taxid 269670]: 1 (0.024%)<br>• Parascardovia denticolens [taxid 78258]: 1 (0.024%)<br>• Enterocloster bolteae [taxid 208479]: 1 (0.024%)<br>• Clostridium estertheticum [taxid 238834]: 1 (0.024%)<br>• Lupinus albus [taxid 3870]: 1 (0.024%)<br>• Acetanaerobacterium elongatum [taxid 258515]: 1 (0.024%)<br>• Bacillus mycoides [taxid 1405]: 1 (0.024%)<br>• Kiloniella litopenaei [taxid 1549748]: 1 (0.024%)<br>• other: 6 (0.148%)
Benchmark OTU ID: `CP001022-_Firmicutes`<br>OTU taxon: Exiguobacterium sibiricum 255-15 [taxid 262543]<br>Expected: Exiguobacterium sibiricum [taxid 332410] (species)<br>Number of reads: 4086<br>Number of identified reads: 4066 (99.51%)	• species: 1102 (26.97%)<br>• **genus: 1886 (46.157%)**<br>• order: 269 (6.583%)<br>• class: 45 (1.101%)<br>• phylum: 44 (1.076%)<br>• superkingdom: 250 (6.118%)<br>• root: 464 (11.355%)	• Exiguobacterium antarcticum [taxid 132920]: 3 (0.073%)<br>• Syntrophaceticus schinkii [taxid 499207]: 2 (0.048%)<br>• Lupinus albus [taxid 3870]: 2 (0.048%)<br>• Mycobacterium tuberculosis [taxid 1773]: 2 (0.048%)<br>• Staphylococcus aureus [taxid 1280]: 1 (0.024%)<br>• Salipaludibacillus aurantiacus [taxid 1601833]: 1 (0.024%)<br>• Virgibacillus dokdonensis [taxid 302167]: 1 (0.024%)<br>• other: 12 (0.293%)
Benchmark OTU ID: `CP002209-_Proteobacteria`<br>OTU taxon: Ferrimonas balearica DSM 9799 [taxid 550540]<br>Expected: Ferrimonas balearica [taxid 44012] (species)<br>Number of reads: 8744<br>Number of identified reads: 8713 (99.645%)	• **species: 6015 (68.79%)**<br>• genus: 174 (1.989%)<br>• family: 0 (0.0%)<br>• order: 275 (3.145%)<br>• class: 817 (9.343%)<br>• phylum: 256 (2.927%)<br>• superkingdom: 351 (4.014%)<br>• root: 820 (9.377%)	• Escherichia coli [taxid 562]: 4 (0.045%)<br>• Ferrimonas marina [taxid 299255]: 2 (0.022%)<br>• Providencia stuartii [taxid 588]: 2 (0.022%)<br>• Salmonella enterica [taxid 28901]: 2 (0.022%)<br>• Shewanella decolorationis [taxid 256839]: 2 (0.022%)<br>• Paenibacillus rigui [taxid 554312]: 2 (0.022%)<br>• Malonomonas rubra [taxid 57040]: 1 (0.011%)<br>• Anaerocolumna cellulosilytica [taxid 433286]: 1 (0.011%)<br>• other: 25 (0.285%)
Benchmark OTU ID: `CP001899-_Euryarchaeota`<br>OTU taxon: Ferroglobus placidus DSM 10642 [taxid 589924]<br>Expected: Ferroglobus placidus [taxid 54261] (species)<br>Number of reads: 1909<br>Number of identified reads: 1894 (99.214%)	• **species: 1213 (63.541%)**<br>• genus: 0 (0.0%)<br>• family: 39 (2.042%)<br>• order: 16 (0.838%)<br>• class: 9 (0.471%)<br>• phylum: 15 (0.785%)<br>• superkingdom: 8 (0.419%)<br>• root: 565 (29.596%)	• Archaeoglobus sulfaticallidus [taxid 1316941]: 1 (0.052%)<br>• Cuerna arida [taxid 1464854]: 1 (0.052%)<br>• Dendrobium catenatum [taxid 906689]: 1 (0.052%)<br>• Roseomonas rosea [taxid 198092]: 1 (0.052%)<br>• Paxillus rubicundulus [taxid 463315]: 1 (0.052%)<br>• Geoglobus ahangari [taxid 113653]: 1 (0.052%)<br>• Thermosulfidibacter takaii [taxid 412593]: 1 (0.052%)<br>• Escherichia coli [taxid 562]: 1 (0.052%)<br>• other: 2 (0.104%)

Operational Taxonomic Unit (OTU)	Correct identifications	Wrong or overspecific identifications at species rank
Benchmark OTU ID: `CP003423-_Crenarchaeota`<br>OTU taxon: Fervidicoccus fontis Kam940 [taxid 1163730]<br>Expected: Fervidicoccus fontis [taxid 683846] (species)<br>Number of reads: 2423<br>Number of identified reads: 2369 (97.771%)	• **species: 1603 (66.157%)**<br>• genus: 92 (3.796%)<br>• family: 0 (0.0%)<br>• order: 0 (0.0%)<br>• class: 23 (0.949%)<br>• phylum: 0 (0.0%)<br>• superkingdom: 26 (1.073%)<br>• root: 603 (24.886%)	• Methanoculleus bourgensis [taxid 83986]: 1 (0.041%)
Benchmark OTU ID: `AP008971-_Firmicutes`<br>OTU taxon: Finegoldia magna ATCC 29328 [taxid 334413]<br>Expected: Finegoldia magna [taxid 1260] (species)<br>Number of reads: 2107<br>Number of identified reads: 2086 (99.003%)	• **species: 1035 (49.121%)**<br>• genus: 593 (28.144%)<br>• family: 33 (1.566%)<br>• order: 1 (0.047%)<br>• class: 5 (0.237%)<br>• phylum: 144 (6.834%)<br>• superkingdom: 87 (4.129%)<br>• root: 188 (8.922%)	• [Eubacterium] sulci [taxid 143393]: 1 (0.047%)<br>• Mycoplasma haemocanis [taxid 136241]: 1 (0.047%)<br>• Caloramator quimbayensis [taxid 1147123]: 1 (0.047%)<br>• Pandoraea apista [taxid 93218]: 1 (0.047%)<br>• Streptococcus pneumoniae [taxid 1313]: 1 (0.047%)
Benchmark OTU ID: `CP001673-_Bacteroidetes`<br>OTU taxon: Flavobacteriaceae bacterium 3519-10 [taxid 531844]<br>Expected: Flavobacteriaceae [taxid 49546] (family)<br>Number of reads: 17227<br>Number of identified reads: 17163 (99.628%)	• **family: 8087 (46.943%)**<br>• order: 5927 (34.405%)<br>• class: 3 (0.017%)<br>• phylum: 525 (3.047%)<br>• superkingdom: 693 (4.022%)<br>• root: 1908 (11.075%)	• Flavobacteriaceae bacterium JJC [taxid 512012]: 256 (1.486%)<br>• Lupinus albus [taxid 3870]: 8 (0.046%)<br>• Chryseobacterium haifense [taxid 421525]: 7 (0.04%)<br>• Bergeyella cardium [taxid 1585976]: 3 (0.017%)<br>• Chryseobacterium taklimakanense [taxid 536441]: 3 (0.017%)<br>• Chryseobacterium jeonii [taxid 266749]: 3 (0.017%)<br>• other: 90 (0.522%)
Benchmark OTU ID: `FQ859183-_Bacteroidetes`<br>OTU taxon: Flavobacterium branchiophilum FL-15 [taxid 1034807]<br>Expected: Flavobacterium branchiophilum [taxid 55197] (species)<br>Number of reads: 23243<br>Number of identified reads: 22846 (98.291%)	• **species: 15499 (66.682%)**<br>• genus: 1664 (7.159%)<br>• family: 983 (4.229%)<br>• order: 295 (1.269%)<br>• class: 53 (0.228%)<br>• phylum: 700 (3.011%)<br>• superkingdom: 857 (3.687%)<br>• root: 2766 (11.9%)	• Beta vulgaris [taxid 161934]: 5 (0.021%)<br>• Flavobacterium kingsejongi [taxid 1678728]: 4 (0.017%)<br>• Flavobacterium hiemivividum [taxid 2541734]: 3 (0.012%)<br>• Flavobacterium columnare [taxid 996]: 3 (0.012%)<br>• Flavobacterium terrigena [taxid 402734]: 3 (0.012%)<br>• Lupinus albus [taxid 3870]: 3 (0.012%)<br>• Flavobacterium xueshanense [taxid 935223]: 2 (0.008%)<br>• Flavobacterium faecale [taxid 1355330]: 2 (0.008%)<br>• other: 86 (0.37%)
Benchmark OTU ID: `HE774682-_Bacteroidetes`<br>OTU taxon: Flavobacterium indicum GPTSA100-9 = DSM 17447 [taxid 1094466]<br>Expected: Flavobacterium indicum [taxid 312277] (species)<br>Number of reads: 18936<br>Number of identified reads: 18710 (98.806%)	• **species: 12157 (64.2%)**<br>• genus: 1492 (7.879%)<br>• family: 1340 (7.076%)<br>• order: 444 (2.344%)<br>• class: 116 (0.612%)<br>• phylum: 686 (3.622%)<br>• superkingdom: 737 (3.892%)<br>• root: 1722 (9.093%)	• Flavobacterium terrigena [taxid 402734]: 20 (0.105%)<br>• Cecembia rubra [taxid 1485585]: 11 (0.058%)<br>• Flavobacterium urocaniciphilum [taxid 1299341]: 5 (0.026%)<br>• Beta vulgaris [taxid 161934]: 5 (0.026%)<br>• Flavobacterium tibetense [taxid 2233533]: 3 (0.015%)<br>• Flavobacterium cauense [taxid 510946]: 3 (0.015%)<br>• Flavobacterium columnare [taxid 996]: 3 (0.015%)<br>• Flavobacterium cheniae [taxid 295428]: 2 (0.01%)<br>• other: 82 (0.433%)

Operational Taxonomic Unit (OTU)	Correct identifications	Wrong or overspecific identifications at species rank
Benchmark OTU ID: `CP002542-_Bacteroidetes`<br>OTU taxon: Fluviicola taffensis DSM 16823 [taxid 755732]<br>Expected: Fluviicola taffensis [taxid 191579] (species)<br>Number of reads: 31404<br>Number of identified reads: 31194 (99.331%)	• **species: 22865 (72.809%)**<br>• genus: 1670 (5.317%)<br>• family: 166 (0.528%)<br>• order: 606 (1.929%)<br>• class: 47 (0.149%)<br>• phylum: 1237 (3.938%)<br>• superkingdom: 1226 (3.903%)<br>• root: 3356 (10.686%)	• Fluviicola chungangensis [taxid 2597671]: 28 (0.089%)<br>• Siphonobacter aquaeclarae [taxid 563176]: 2 (0.006%)<br>• Parabacteroides merdae [taxid 46503]: 2 (0.006%)<br>• Porphyromonas crevioricanis [taxid 393921]: 2 (0.006%)<br>• Flavobacterium aurantiibacter [taxid 2023067]: 2 (0.006%)<br>• Flavobacterium branchiophilum [taxid 55197]: 2 (0.006%)<br>• Mycoplasma wenyonii [taxid 65123]: 2 (0.006%)<br>• Gossypium mustelinum [taxid 34275]: 1 (0.003%)<br>• other: 43 (0.136%)
Benchmark OTU ID: `CP002558-_Pathogens`<br>OTU taxon: Francisella hispaniensis [taxid 622488]<br>Expected: Francisella hispaniensis [taxid 622488] (species)<br>Number of reads: 3649<br>Number of identified reads: 3609 (98.903%)	• **species: 1467 (40.202%)**<br>• genus: 1238 (33.927%)<br>• family: 356 (9.756%)<br>• order: 2 (0.054%)<br>• class: 79 (2.164%)<br>• phylum: 55 (1.507%)<br>• superkingdom: 109 (2.987%)<br>• root: 300 (8.221%)	• Francisella tularensis [taxid 263]: 8 (0.219%)<br>• Francisella opportunistica [taxid 2016517]: 7 (0.191%)<br>• Francisella persica [taxid 954]: 6 (0.164%)<br>• Francisella uliginis [taxid 573570]: 4 (0.109%)<br>• Francisella philomiragia [taxid 28110]: 4 (0.109%)<br>• Francisella frigiditurris [taxid 1542390]: 3 (0.082%)<br>• Francisella adeliensis [taxid 2007306]: 3 (0.082%)<br>• Entamoeba invadens [taxid 33085]: 1 (0.027%)<br>• other: 10 (0.274%)
Benchmark OTU ID: `CP000937-_Pathogens`<br>OTU taxon: Francisella philomiragia subsp. philomiragia ATCC 25017 [taxid 484022]<br>Expected: Francisella philomiragia [taxid 28110] (species)<br>Number of reads: 3900<br>Number of identified reads: 3861 (99.0%)	• species: 1356 (34.769%)<br>• **genus: 1671 (42.846%)**<br>• family: 247 (6.333%)<br>• order: 2 (0.051%)<br>• class: 63 (1.615%)<br>• phylum: 73 (1.871%)<br>• superkingdom: 152 (3.897%)<br>• root: 288 (7.384%)	• Francisella uliginis [taxid 573570]: 9 (0.23%)<br>• Francisella orientalis [taxid 299583]: 6 (0.153%)<br>• Francisella tularensis [taxid 263]: 6 (0.153%)<br>• Francisella adeliensis [taxid 2007306]: 5 (0.128%)<br>• Francisella hispaniensis [taxid 622488]: 4 (0.102%)<br>• Amblyomma maculatum [taxid 34609]: 4 (0.102%)<br>• Francisella opportunistica [taxid 2016517]: 4 (0.102%)<br>• Elysia chlorotica [taxid 188477]: 2 (0.051%)<br>• other: 18 (0.461%)
Benchmark OTU ID: `CP002872-_Pathogens`<br>OTU taxon: Francisella salina [taxid 573569]<br>Expected: Francisella salina [taxid 573569] (species)<br>Number of reads: 3876<br>Number of identified reads: 3821 (98.581%)	• species: 810 (20.897%)<br>• **genus: 2239 (57.765%)**<br>• family: 196 (5.056%)<br>• order: 2 (0.051%)<br>• class: 73 (1.883%)<br>• phylum: 63 (1.625%)<br>• superkingdom: 130 (3.353%)<br>• root: 304 (7.843%)	• Francisella marina [taxid 2249302]: 22 (0.567%)<br>• Francisella philomiragia [taxid 28110]: 18 (0.464%)<br>• Francisella frigiditurris [taxid 1542390]: 5 (0.128%)<br>• Francisella uliginis [taxid 573570]: 5 (0.128%)<br>• Francisella adeliensis [taxid 2007306]: 5 (0.128%)<br>• Francisella orientalis [taxid 299583]: 5 (0.128%)<br>• Francisella hispaniensis [taxid 622488]: 4 (0.103%)<br>• Francisella tularensis [taxid 263]: 3 (0.077%)<br>• other: 10 (0.257%)

Operational Taxonomic Unit (OTU)	Correct identifications	Wrong or overspecific identifications at species rank
Benchmark OTU ID: `CP003862-_Pathogens`<br>OTU taxon: Francisella tularensis subsp. holarctica FSC200 [taxid 351581]<br>Expected: Francisella tularensis [taxid 263] (species)<br>Number of reads: 3521<br>Number of identified reads: 3469 (98.523%)	• species: 1047 (29.735%)<br>• **genus: 1535 (43.595%)**<br>• family: 354 (10.053%)<br>• order: 2 (0.056%)<br>• class: 70 (1.988%)<br>• phylum: 57 (1.618%)<br>• superkingdom: 135 (3.834%)<br>• root: 267 (7.583%)	• Francisella persica [taxid 954]: 9 (0.255%)<br>• Francisella hispaniensis [taxid 622488]: 7 (0.198%)<br>• Francisella adeliensis [taxid 2007306]: 7 (0.198%)<br>• Francisella endosymbiont of Ornithodoros moubata [taxid 1981171]: 4 (0.113%)<br>• Francisella opportunistica [taxid 2016517]: 4 (0.113%)<br>• Francisella halioticida [taxid 549298]: 3 (0.085%)<br>• Francisella philomiragia [taxid 28110]: 3 (0.085%)<br>• Francisella orientalis [taxid 299583]: 3 (0.085%)<br>• other: 9 (0.255%)
Benchmark OTU ID: `CP000803-_Pathogens`<br>OTU taxon: Francisella tularensis subsp. holarctica FTNF002-00 [taxid 458234]<br>Expected: Francisella tularensis [taxid 263] (species)<br>Number of reads: 3513<br>Number of identified reads: 3453 (98.292%)	• species: 1045 (29.746%)<br>• **genus: 1534 (43.666%)**<br>• family: 341 (9.706%)<br>• order: 4 (0.113%)<br>• class: 52 (1.48%)<br>• phylum: 61 (1.736%)<br>• superkingdom: 106 (3.017%)<br>• root: 306 (8.71%)	• Francisella opportunistica [taxid 2016517]: 4 (0.113%)<br>• Francisella adeliensis [taxid 2007306]: 4 (0.113%)<br>• Francisella hispaniensis [taxid 622488]: 4 (0.113%)<br>• Francisella halioticida [taxid 549298]: 3 (0.085%)<br>• Allofrancisella frigidaquae [taxid 1085644]: 3 (0.085%)<br>• Francisella endosymbiont of Ornithodoros moubata [taxid 1981171]: 3 (0.085%)<br>• Francisella philomiragia [taxid 28110]: 2 (0.056%)<br>• Alcanivorax profundi [taxid 2338368]: 1 (0.028%)<br>• other: 10 (0.284%)
Benchmark OTU ID: `BK006741-_Pathogens`<br>OTU taxon: Francisella tularensis subsp. holarctica OSU18 [taxid 393011]<br>Expected: Francisella tularensis [taxid 263] (species)<br>Number of reads: 3525<br>Number of identified reads: 3481 (98.751%)	• species: 1068 (30.297%)<br>• **genus: 1559 (44.226%)**<br>• family: 315 (8.936%)<br>• order: 2 (0.056%)<br>• class: 56 (1.588%)<br>• phylum: 68 (1.929%)<br>• superkingdom: 125 (3.546%)<br>• root: 281 (7.971%)	• Francisella hispaniensis [taxid 622488]: 11 (0.312%)<br>• Francisella opportunistica [taxid 2016517]: 5 (0.141%)<br>• Francisella persica [taxid 954]: 5 (0.141%)<br>• Francisella philomiragia [taxid 28110]: 4 (0.113%)<br>• Francisella halioticida [taxid 549298]: 3 (0.085%)<br>• Francisella adeliensis [taxid 2007306]: 3 (0.085%)<br>• Francisella uliginis [taxid 573570]: 3 (0.085%)<br>• Francisella orientalis [taxid 299583]: 3 (0.085%)<br>• other: 14 (0.397%)
Benchmark OTU ID: `CP000437-_Pathogens`<br>OTU taxon: Francisella tularensis subsp. holarctica OSU18 [taxid 393011]<br>Expected: Francisella tularensis [taxid 263] (species)<br>Number of reads: 3525<br>Number of identified reads: 3468 (98.382%)	• species: 1081 (30.666%)<br>• **genus: 1542 (43.744%)**<br>• family: 334 (9.475%)<br>• order: 1 (0.028%)<br>• class: 46 (1.304%)<br>• phylum: 61 (1.73%)<br>• superkingdom: 126 (3.574%)<br>• root: 270 (7.659%)	• Francisella persica [taxid 954]: 6 (0.17%)<br>• Francisella opportunistica [taxid 2016517]: 6 (0.17%)<br>• Francisella hispaniensis [taxid 622488]: 5 (0.141%)<br>• Francisella philomiragia [taxid 28110]: 5 (0.141%)<br>• Francisella frigiditurris [taxid 1542390]: 3 (0.085%)<br>• Francisella halioticida [taxid 549298]: 3 (0.085%)<br>• Francisella adeliensis [taxid 2007306]: 3 (0.085%)<br>• Francisella orientalis [taxid 299583]: 3 (0.085%)<br>• other: 19 (0.539%)

Operational Taxonomic Unit (OTU)	Correct identifications	Wrong or overspecific identifications at species rank
Benchmark OTU ID: `CP003048-_Pathogens`<br>OTU taxon: Francisella tularensis subsp. tularensis TIGB03 [taxid 1001542]<br>Expected: Francisella tularensis [taxid 263] (species)<br>Number of reads: 3708<br>Number of identified reads: 3659 (98.678%)	• species: 1053 (28.398%)<br>• **genus: 1678 (45.253%)**<br>• family: 342 (9.223%)<br>• order: 1 (0.026%)<br>• class: 81 (2.184%)<br>• phylum: 63 (1.699%)<br>• superkingdom: 137 (3.694%)<br>• root: 296 (7.982%)	• Francisella opportunistica [taxid 2016517]: 9 (0.242%)<br>• Francisella hispaniensis [taxid 622488]: 8 (0.215%)<br>• Francisella uliginis [taxid 573570]: 4 (0.107%)<br>• Francisella frigiditurris [taxid 1542390]: 4 (0.107%)<br>• Francisella adeliensis [taxid 2007306]: 4 (0.107%)<br>• Francisella halioticida [taxid 549298]: 4 (0.107%)<br>• Francisella persica [taxid 954]: 3 (0.08%)<br>• Francisella philomiragia [taxid 28110]: 3 (0.08%)<br>• other: 19 (0.512%)
Benchmark OTU ID: `CT573213-_Actinobacteria`<br>OTU taxon: Frankia alni ACN14a [taxid 326424]<br>Expected: Frankia alni [taxid 1859] (species)<br>Number of reads: 34337<br>Number of identified reads: 34159 (99.481%)	• **species: 13923 (40.548%)**<br>• genus: 10081 (29.359%)<br>• family: 0 (0.0%)<br>• order: 4 (0.011%)<br>• class: 3245 (9.45%)<br>• phylum: 40 (0.116%)<br>• superkingdom: 2645 (7.703%)<br>• root: 4163 (12.123%)	• Frankia torreyi [taxid 1856]: 60 (0.174%)<br>• Frankia symbiont of Alnus alnobetula [taxid 2699483]: 37 (0.107%)<br>• Frankia canadensis [taxid 1836972]: 35 (0.101%)<br>• Frankia inefficax [taxid 298654]: 6 (0.017%)<br>• Candidatus Frankia californiensis [taxid 1839754]: 3 (0.008%)<br>• Frankia casuarinae [taxid 106370]: 3 (0.008%)<br>• Frankia asymbiotica [taxid 1834516]: 3 (0.008%)<br>• Candidatus Frankia datiscae [taxid 2716812]: 3 (0.008%)<br>• other: 129 (0.375%)
Benchmark OTU ID: `CP002299-_Actinobacteria`<br>OTU taxon: Frankia inefficax [taxid 298654]<br>Expected: Frankia inefficax [taxid 298654] (species)<br>Number of reads: 40794<br>Number of identified reads: 40575 (99.463%)	• **species: 26058 (63.877%)**<br>• genus: 2469 (6.052%)<br>• family: 0 (0.0%)<br>• order: 1 (0.002%)<br>• class: 3995 (9.793%)<br>• phylum: 80 (0.196%)<br>• superkingdom: 3141 (7.699%)<br>• root: 4771 (11.695%)	• Frankia asymbiotica [taxid 1834516]: 11 (0.026%)<br>• Frankia canadensis [taxid 1836972]: 4 (0.009%)<br>• Mycobacteroides abscessus [taxid 36809]: 3 (0.007%)<br>• Streptomyces glauciniger [taxid 235986]: 2 (0.004%)<br>• Corethrella appendiculata [taxid 1370023]: 2 (0.004%)<br>• Cutibacterium acnes [taxid 1747]: 2 (0.004%)<br>• Cryptosporangium phraense [taxid 2593070]: 2 (0.004%)<br>• Glycomyces paridis [taxid 2126555]: 2 (0.004%)<br>• other: 138 (0.338%)
Benchmark OTU ID: `CP000820-_Actinobacteria`<br>OTU taxon: Frankia sp. EAN1pec [taxid 298653]<br>Expected: Frankia [taxid 1854] (genus)<br>Number of reads: 41609<br>Number of identified reads: 41299 (99.254%)	• **genus: 28825 (69.275%)**<br>• family: 0 (0.0%)<br>• order: 0 (0.0%)<br>• class: 3828 (9.199%)<br>• phylum: 48 (0.115%)<br>• superkingdom: 2891 (6.948%)<br>• root: 5633 (13.537%)	• Frankia soli [taxid 2599596]: 84 (0.201%)<br>• Frankia symbiont of Alnus alnobetula [taxid 2699483]: 33 (0.079%)<br>• Frankia canadensis [taxid 1836972]: 20 (0.048%)<br>• Frankia irregularis [taxid 795642]: 19 (0.045%)<br>• Frankia inefficax [taxid 298654]: 18 (0.043%)<br>• Frankia alni [taxid 1859]: 11 (0.026%)<br>• Frankia torreyi [taxid 1856]: 6 (0.014%)<br>• other: 163 (0.391%)

Operational Taxonomic Unit (OTU)	Correct identifications	Wrong or overspecific identifications at species rank
Benchmark OTU ID: `CP002159-_Proteobacteria`<br>OTU taxon: Gallionella capsiferriformans ES-2 [taxid 395494]<br>Expected: Gallionella capsiferriformans [taxid 370405] (species)<br>Number of reads: 6231<br>Number of identified reads: 6213 (99.711%)	• **species: 3005 (48.226%)**<br>• genus: 22 (0.353%)<br>• family: 30 (0.481%)<br>• order: 1375 (22.067%)<br>• class: 272 (4.365%)<br>• phylum: 525 (8.425%)<br>• superkingdom: 272 (4.365%)<br>• root: 708 (11.362%)	• Acidovorax delafieldii [taxid 47920]: 1 (0.016%)<br>• Photorhabdus namnaonensis [taxid 1851568]: 1 (0.016%)<br>• Ferriphaselus amnicola [taxid 1188319]: 1 (0.016%)<br>• Desulfobacterium vacuolatum [taxid 2298]: 1 (0.016%)<br>• Bodo saltans [taxid 75058]: 1 (0.016%)<br>• Candidatus Kentron sp. TC [taxid 2126339]: 1 (0.016%)<br>• Francisella persica [taxid 954]: 1 (0.016%)<br>• Pseudomonas pohangensis [taxid 364197]: 1 (0.016%)<br>• other: 11 (0.176%)
Benchmark OTU ID: `chr28-_Eukaryotes`<br>OTU taxon: Gallus gallus [taxid 9031]<br>Expected: Gallus gallus [taxid 9031] (species)<br>Number of reads: 171647<br>Number of identified reads: 159161 (92.725%)	• species: 2416 (1.407%)<br>• genus: 0 (0.0%)<br>• family: 2621 (1.526%)<br>• order: 1446 (0.842%)<br>• class: 4715 (2.746%)<br>• phylum: 6878 (4.007%)<br>• superkingdom: 2880 (1.677%)<br>• **root: 134222 (78.196%)**	• Bambusicola thoracicus [taxid 9083]: 360 (0.209%)<br>• Phasianus colchicus [taxid 9054]: 232 (0.135%)<br>• Limosa lapponica [taxid 161683]: 148 (0.086%)<br>• Meleagris gallopavo [taxid 9103]: 98 (0.057%)<br>• Callipepla squamata [taxid 9009]: 72 (0.041%)<br>• Hirundo rustica [taxid 43150]: 62 (0.036%)<br>• Anas platyrhynchos [taxid 8839]: 50 (0.029%)<br>• Colinus virginianus [taxid 9014]: 40 (0.023%)<br>• other: 1298 (0.756%)
Benchmark OTU ID: `CP001849-_Actinobacteria`<br>OTU taxon: Gardnerella vaginalis 409-05 [taxid 553190]<br>Expected: Gardnerella vaginalis [taxid 2702] (species)<br>Number of reads: 5523<br>Number of identified reads: 5492 (99.438%)	• species: 489 (8.853%)<br>• genus: 37 (0.669%)<br>• **family: 3273 (59.261%)**<br>• order: 0 (0.0%)<br>• class: 153 (2.77%)<br>• phylum: 13 (0.235%)<br>• superkingdom: 1027 (18.594%)<br>• root: 495 (8.962%)	• Bifidobacterium longum [taxid 216816]: 6 (0.108%)<br>• Bifidobacterium breve [taxid 1685]: 2 (0.036%)<br>• Bifidobacterium bombi [taxid 471511]: 2 (0.036%)<br>• Bifidobacterium pseudolongum [taxid 1694]: 2 (0.036%)<br>• Bifidobacterium minimum [taxid 1693]: 2 (0.036%)<br>• Bifidobacterium adolescentis [taxid 1680]: 2 (0.036%)<br>• Bifidobacterium bifidum [taxid 1681]: 2 (0.036%)<br>• Bifidobacterium animalis [taxid 28025]: 2 (0.036%)<br>• other: 34 (0.615%)
Benchmark OTU ID: `CP002104-_Actinobacteria`<br>OTU taxon: Gardnerella vaginalis ATCC 14019 [taxid 525284]<br>Expected: Gardnerella vaginalis [taxid 2702] (species)<br>Number of reads: 5767<br>Number of identified reads: 5733 (99.41%)	• **species: 2890 (50.112%)**<br>• genus: 22 (0.381%)<br>• family: 1782 (30.899%)<br>• order: 0 (0.0%)<br>• class: 97 (1.681%)<br>• phylum: 6 (0.104%)<br>• superkingdom: 327 (5.67%)<br>• root: 603 (10.456%)	• Bifidobacterium tsurumiense [taxid 356829]: 3 (0.052%)<br>• Bifidobacterium bifidum [taxid 1681]: 3 (0.052%)<br>• Peptoniphilus lacrimalis [taxid 33031]: 2 (0.034%)<br>• Bombiscardovia coagulans [taxid 686666]: 2 (0.034%)<br>• Bifidobacterium minimum [taxid 1693]: 2 (0.034%)<br>• Bifidobacterium adolescentis [taxid 1680]: 2 (0.034%)<br>• Bifidobacterium psychraerophilum [taxid 218140]: 2 (0.034%)<br>• Bifidobacterium mongoliense [taxid 518643]: 2 (0.034%)<br>• other: 25 (0.433%)

Operational Taxonomic Unit (OTU)	Correct identifications	Wrong or overspecific identifications at species rank
Benchmark OTU ID: `CP002725-_Actinobacteria`<br>OTU taxon: Gardnerella vaginalis HMP9231 [taxid 1009464]<br>Expected: Gardnerella vaginalis [taxid 2702] (species)<br>Number of reads: 6057<br>Number of identified reads: 6036 (99.653%)	• **species: 2968 (49.001%)**<br>• genus: 22 (0.363%)<br>• family: 1903 (31.418%)<br>• order: 0 (0.0%)<br>• class: 117 (1.931%)<br>• phylum: 5 (0.082%)<br>• superkingdom: 365 (6.026%)<br>• root: 649 (10.714%)	• Brugia timori [taxid 42155]: 3 (0.049%)<br>• Bifidobacterium longum [taxid 216816]: 3 (0.049%)<br>• Bifidobacterium psychraerophilum [taxid 218140]: 2 (0.033%)<br>• Pseudoscardovia radai [taxid 987066]: 2 (0.033%)<br>• Bifidobacterium bombi [taxid 471511]: 2 (0.033%)<br>• Bifidobacterium adolescentis [taxid 1680]: 2 (0.033%)<br>• Bifidobacterium bifidum [taxid 1681]: 2 (0.033%)<br>• Bifidobacterium minimum [taxid 1693]: 1 (0.016%)<br>• other: 18 (0.297%)
Benchmark OTU ID: `CP003591-_Cyanobacteria`<br>OTU taxon: Geitlerinema sp. PCC 7407 [taxid 1173025]<br>Expected: Geitlerinema [taxid 63132] (genus)<br>Number of reads: 27035<br>Number of identified reads: 26848 (99.308%)	• **genus: 17599 (65.097%)**<br>• family: 14 (0.051%)<br>• order: 262 (0.969%)<br>• phylum: 2767 (10.234%)<br>• superkingdom: 1985 (7.342%)<br>• root: 4130 (15.276%)	• Phormidesmis priestleyi [taxid 268141]: 4 (0.014%)<br>• Synechococcus elongatus [taxid 32046]: 3 (0.011%)<br>• Salmonella enterica [taxid 28901]: 3 (0.011%)<br>• Roseofilum reptotaenium [taxid 1233427]: 3 (0.011%)<br>• Oscillatoria acuminata [taxid 118323]: 2 (0.007%)<br>• Aphanothece hegewaldii [taxid 1521625]: 2 (0.007%)<br>• other: 84 (0.31%)
Benchmark OTU ID: `AP009153-_Gemmatimonadetes`<br>OTU taxon: Gemmatimonas aurantiaca T-27 [taxid 379066]<br>Expected: Gemmatimonas aurantiaca [taxid 173480] (species)<br>Number of reads: 202389<br>Number of identified reads: 201637 (99.628%)	• **species: 147928 (73.09%)**<br>• genus: 4775 (2.359%)<br>• family: 247 (0.122%)<br>• order: 92 (0.045%)<br>• class: 0 (0.0%)<br>• phylum: 3977 (1.965%)<br>• superkingdom: 23554 (11.637%)<br>• root: 20908 (10.33%)	• Gemmatimonas phototrophica [taxid 1379270]: 47 (0.023%)<br>• Pelagomonas calceolata [taxid 35677]: 7 (0.003%)<br>• Gemmatirosa kalamazoonesis [taxid 861299]: 6 (0.002%)<br>• Beta vulgaris [taxid 161934]: 5 (0.002%)<br>• bacterium [taxid 1869227]: 4 (0.001%)<br>• Salmonella enterica [taxid 28901]: 3 (0.001%)<br>• Cellulomonas gilvus [taxid 11]: 3 (0.001%)<br>• Alexandrium monilatum [taxid 311494]: 3 (0.001%)<br>• other: 324 (0.16%)
Benchmark OTU ID: `CP001638-_Firmicutes`<br>OTU taxon: Geobacillus sp. WCH70 [taxid 471223]<br>Expected: Geobacillus [taxid 129337] (genus)<br>Number of reads: 4774<br>Number of identified reads: 4740 (99.287%)	• genus: 543 (11.374%)<br>• **family: 3014 (63.133%)**<br>• order: 281 (5.886%)<br>• class: 37 (0.775%)<br>• phylum: 60 (1.256%)<br>• superkingdom: 230 (4.817%)<br>• root: 570 (11.939%)	• Parageobacillus toebii [taxid 153151]: 41 (0.858%)<br>• Parageobacillus galactosidasius [taxid 883812]: 19 (0.397%)<br>• Parageobacillus caldoxylosilyticus [taxid 81408]: 16 (0.335%)<br>• Parageobacillus thermoglucosidasius [taxid 1426]: 13 (0.272%)<br>• Geobacillus stearothermophilus [taxid 1422]: 10 (0.209%)<br>• Geobacillus kaustophilus [taxid 1462]: 8 (0.167%)<br>• Geobacillus subterraneus [taxid 129338]: 7 (0.146%)<br>• other: 85 (1.78%)
Benchmark OTU ID: `CP002293-_Firmicutes`<br>OTU taxon: Geobacillus sp. Y4.1MC1 [taxid 581103]<br>Expected: Geobacillus [taxid 129337] (genus)<br>Number of reads: 5375<br>Number of identified reads: 5345 (99.441%)	• genus: 28 (0.52%)<br>• **family: 3964 (73.748%)**<br>• order: 232 (4.316%)<br>• class: 49 (0.911%)<br>• phylum: 65 (1.209%)<br>• superkingdom: 283 (5.265%)<br>• root: 712 (13.246%)	• **Parageobacillus thermoglucosidasius [taxid 1426]: 383 (7.125%)**<br>• Anoxybacillus flavithermus [taxid 33934]: 10 (0.186%)<br>• Parageobacillus thermantarcticus [taxid 186116]: 6 (0.111%)<br>• Parageobacillus caldoxylosilyticus [taxid 81408]: 6 (0.111%)<br>• Daphnia magna [taxid 35525]: 5 (0.093%)<br>• Geobacillus thermoleovorans [taxid 33941]: 5 (0.093%)<br>• Anoxybacillus vitaminiphilus [taxid 581036]: 4 (0.074%)<br>• other: 69 (1.283%)

Operational Taxonomic Unit (OTU)	Correct identifications	Wrong or overspecific identifications at species rank
Benchmark OTU ID: `CP002442-_Firmicutes`<br>OTU taxon: Geobacillus sp. Y412MC52 [taxid 550542]<br>Expected: Geobacillus [taxid 129337] (genus)<br>Number of reads: 5037<br>Number of identified reads: 5017 (99.602%)	• **genus: 3156 (62.656%)**<br>• family: 634 (12.586%)<br>• order: 232 (4.605%)<br>• class: 36 (0.714%)<br>• phylum: 58 (1.151%)<br>• superkingdom: 265 (5.261%)<br>• root: 628 (12.467%)	• Geobacillus stearothermophilus [taxid 1422]: 36 (0.714%)<br>• Geobacillus thermoleovorans [taxid 33941]: 24 (0.476%)<br>• Geobacillus kaustophilus [taxid 1462]: 21 (0.416%)<br>• Geobacillus proteiniphilus [taxid 860353]: 16 (0.317%)<br>• Geobacillus subterraneus [taxid 129338]: 5 (0.099%)<br>• Geobacillus icigianus [taxid 1430331]: 2 (0.039%)<br>• Hevea brasiliensis [taxid 3981]: 2 (0.039%)<br>• other: 34 (0.675%)
Benchmark OTU ID: `CP001794-_Firmicutes`<br>OTU taxon: Geobacillus sp. Y412MC61 [taxid 544556]<br>Expected: Geobacillus [taxid 129337] (genus)<br>Number of reads: 5027<br>Number of identified reads: 5005 (99.562%)	• **genus: 3157 (62.8%)**<br>• family: 627 (12.472%)<br>• order: 231 (4.595%)<br>• class: 45 (0.895%)<br>• phylum: 69 (1.372%)<br>• superkingdom: 259 (5.152%)<br>• root: 614 (12.214%)	• Geobacillus stearothermophilus [taxid 1422]: 32 (0.636%)<br>• Geobacillus kaustophilus [taxid 1462]: 27 (0.537%)<br>• Geobacillus proteiniphilus [taxid 860353]: 22 (0.437%)<br>• Geobacillus thermoleovorans [taxid 33941]: 16 (0.318%)<br>• Parageobacillus galactosidasius [taxid 883812]: 3 (0.059%)<br>• Geobacillus thermodenitrificans [taxid 33940]: 2 (0.039%)<br>• [Bacillus] caldolyticus [taxid 1394]: 2 (0.039%)<br>• other: 36 (0.716%)
Benchmark OTU ID: `CP000557-_Firmicutes`<br>OTU taxon: Geobacillus thermodenitrificans NG80-2 [taxid 420246]<br>Expected: Geobacillus thermodenitrificans [taxid 33940] (species)<br>Number of reads: 4911<br>Number of identified reads: 4879 (99.348%)	• species: 381 (7.758%)<br>• **genus: 2488 (50.661%)**<br>• family: 715 (14.559%)<br>• order: 246 (5.009%)<br>• class: 30 (0.61%)<br>• phylum: 68 (1.384%)<br>• superkingdom: 254 (5.172%)<br>• root: 689 (14.029%)	• Geobacillus subterraneus [taxid 129338]: 6 (0.122%)<br>• Geobacillus kaustophilus [taxid 1462]: 5 (0.101%)<br>• Geobacillus stearothermophilus [taxid 1422]: 5 (0.101%)<br>• Parageobacillus thermoglucosidasius [taxid 1426]: 4 (0.081%)<br>• Geobacillus thermoleovorans [taxid 33941]: 4 (0.081%)<br>• Parageobacillus caldoxylosilyticus [taxid 81408]: 3 (0.061%)<br>• Bacillus coahuilensis [taxid 408580]: 1 (0.02%)<br>• Geobacillus proteiniphilus [taxid 860353]: 1 (0.02%)<br>• other: 26 (0.529%)
Benchmark OTU ID: `CP000148-_Proteobacteria`<br>OTU taxon: Geobacter metallireducens GS-15 [taxid 269799]<br>Expected: Geobacter metallireducens [taxid 28232] (species)<br>Number of reads: 8110<br>Number of identified reads: 8086 (99.704%)	• **species: 5050 (62.268%)**<br>• genus: 384 (4.734%)<br>• family: 102 (1.257%)<br>• order: 387 (4.771%)<br>• class: 120 (1.479%)<br>• phylum: 320 (3.945%)<br>• superkingdom: 656 (8.088%)<br>• root: 1060 (13.07%)	• Geobacter thiogenes [taxid 115783]: 2 (0.024%)<br>• Geobacter lovleyi [taxid 313985]: 2 (0.024%)<br>• Geobacter sulfurreducens [taxid 35554]: 2 (0.024%)<br>• Geobacter pickeringii [taxid 345632]: 2 (0.024%)<br>• Erwinia tracheiphila [taxid 65700]: 1 (0.012%)<br>• bacterium [taxid 1869227]: 1 (0.012%)<br>• Escherichia coli [taxid 562]: 1 (0.012%)<br>• Tetraselmis striata [taxid 3165]: 1 (0.012%)<br>• other: 15 (0.184%)

Operational Taxonomic Unit (OTU)	Correct identifications	Wrong or overspecific identifications at species rank
Benchmark OTU ID: `CP002479-_Proteobacteria`<br>OTU taxon: Geobacter sp. M18 [taxid 443143]<br>Expected: Geobacter [taxid 28231] (genus)<br>Number of reads: 10990<br>Number of identified reads: 10953 (99.663%)	• **genus: 6886 (62.656%)**<br>• family: 1264 (11.501%)<br>• order: 153 (1.392%)<br>• class: 126 (1.146%)<br>• phylum: 368 (3.348%)<br>• superkingdom: 828 (7.534%)<br>• root: 1319 (12.001%)	• Geomonas paludis [taxid 2740185]: 17 (0.154%)<br>• Geobacter pelophilus [taxid 60036]: 10 (0.09%)<br>• Geobacter bemidjiensis [taxid 225194]: 9 (0.081%)<br>• Geobacter sp. SVR [taxid 2495594]: 6 (0.054%)<br>• Geobacter uraniireducens [taxid 351604]: 5 (0.045%)<br>• Geomonas terrae [taxid 2562681]: 3 (0.027%)<br>• Geobacter argillaceus [taxid 345631]: 2 (0.018%)<br>• other: 41 (0.373%)
Benchmark OTU ID: `CP002031-_Proteobacteria`<br>OTU taxon: Geobacter sulfurreducens KN400 [taxid 663917]<br>Expected: Geobacter sulfurreducens [taxid 35554] (species)<br>Number of reads: 7473<br>Number of identified reads: 7445 (99.625%)	• **species: 3225 (43.155%)**<br>• genus: 1836 (24.568%)<br>• family: 92 (1.231%)<br>• order: 289 (3.867%)<br>• class: 98 (1.311%)<br>• phylum: 289 (3.867%)<br>• superkingdom: 654 (8.751%)<br>• root: 954 (12.765%)	• Geobacter soli [taxid 1510391]: 21 (0.281%)<br>• Geobacter metallireducens [taxid 28232]: 4 (0.053%)<br>• Geobacter pickeringii [taxid 345632]: 3 (0.04%)<br>• Geobacter thiogenes [taxid 115783]: 3 (0.04%)<br>• Geobacter uraniireducens [taxid 351604]: 2 (0.026%)<br>• Rheinheimera nanhaiensis [taxid 1163621]: 2 (0.026%)<br>• Vibrio alginolyticus [taxid 663]: 1 (0.013%)<br>• Plasmopara halstedii [taxid 4781]: 1 (0.013%)<br>• other: 30 (0.401%)
Benchmark OTU ID: `AE017180-_Proteobacteria`<br>OTU taxon: Geobacter sulfurreducens PCA [taxid 243231]<br>Expected: Geobacter sulfurreducens [taxid 35554] (species)<br>Number of reads: 7698<br>Number of identified reads: 7669 (99.623%)	• **species: 3496 (45.414%)**<br>• genus: 1844 (23.954%)<br>• family: 86 (1.117%)<br>• order: 309 (4.014%)<br>• class: 109 (1.415%)<br>• phylum: 270 (3.507%)<br>• superkingdom: 619 (8.041%)<br>• root: 934 (12.133%)	• Geobacter soli [taxid 1510391]: 10 (0.129%)<br>• Geobacter metallireducens [taxid 28232]: 3 (0.038%)<br>• Escherichia coli [taxid 562]: 2 (0.025%)<br>• Geobacter lovleyi [taxid 313985]: 2 (0.025%)<br>• Geobacter argillaceus [taxid 345631]: 2 (0.025%)<br>• Geoalkalibacter ferrihydriticus [taxid 392333]: 2 (0.025%)<br>• Geobacter daltonii [taxid 1203471]: 2 (0.025%)<br>• Geobacter pickeringii [taxid 345632]: 2 (0.025%)<br>• other: 24 (0.311%)
Benchmark OTU ID: `CP000698-_Proteobacteria`<br>OTU taxon: Geobacter uraniireducens Rf4 [taxid 351605]<br>Expected: Geobacter uraniireducens [taxid 351604] (species)<br>Number of reads: 10673<br>Number of identified reads: 10647 (99.756%)	• **species: 6841 (64.096%)**<br>• genus: 345 (3.232%)<br>• family: 264 (2.473%)<br>• order: 247 (2.314%)<br>• class: 138 (1.292%)<br>• phylum: 327 (3.063%)<br>• superkingdom: 789 (7.392%)<br>• root: 1687 (15.806%)	• Calditerrivibrio nitroreducens [taxid 477976]: 2 (0.018%)<br>• Geobacter pickeringii [taxid 345632]: 2 (0.018%)<br>• Staphylococcus aureus [taxid 1280]: 2 (0.018%)<br>• bacterium [taxid 1869227]: 1 (0.009%)<br>• Paenibacillus mucilaginosus [taxid 61624]: 1 (0.009%)<br>• Geoalkalibacter subterraneus [taxid 483547]: 1 (0.009%)<br>• Lentzea xinjiangensis [taxid 402600]: 1 (0.009%)<br>• Geobacter soli [taxid 1510391]: 1 (0.009%)<br>• other: 21 (0.196%)

Operational Taxonomic Unit (OTU)	Correct identifications	Wrong or overspecific identifications at species rank
Benchmark OTU ID: `CP003060-_Proteobacteria`<br>OTU taxon: Glaciecola nitratireducens FR1064 [taxid 1085623]<br>Expected: Glaciecola nitratireducens [taxid 300231] (species)<br>Number of reads: 8418<br>Number of identified reads: 8379 (99.536%)	• **species: 5497 (65.3%)**<br>• genus: 583 (6.925%)<br>• family: 224 (2.66%)<br>• order: 167 (1.983%)<br>• class: 551 (6.545%)<br>• phylum: 204 (2.423%)<br>• superkingdom: 297 (3.528%)<br>• root: 847 (10.061%)	• Glaciecola pallidula [taxid 56807]: 7 (0.083%)<br>• Kangiella profundi [taxid 1561924]: 1 (0.011%)<br>• Bodo saltans [taxid 75058]: 1 (0.011%)<br>• Woeseia oceani [taxid 1548547]: 1 (0.011%)<br>• Methylomicrobium alcaliphilum [taxid 271065]: 1 (0.011%)<br>• Helicobacter pylori [taxid 210]: 1 (0.011%)<br>• Paraphotobacterium marinum [taxid 1755811]: 1 (0.011%)<br>• Shewanella xiamenensis [taxid 332186]: 1 (0.011%)<br>• other: 26 (0.308%)
Benchmark OTU ID: `CP002526-_Proteobacteria`<br>OTU taxon: Glaciecola sp. 4H-3-7+YE-5 [taxid 983545]<br>Expected: Glaciecola [taxid 89404] (genus)<br>Number of reads: 10484<br>Number of identified reads: 10422 (99.408%)	• genus: 7 (0.066%)<br>• **family: 6131 (58.479%)**<br>• order: 838 (7.993%)<br>• class: 823 (7.85%)<br>• phylum: 951 (9.07%)<br>• superkingdom: 398 (3.796%)<br>• root: 1261 (12.027%)	• **Paraglaciecola agarilytica [taxid 395568]: 390 (3.719%)**<br>• **Paraglaciecola chathamensis [taxid 368405]: 245 (2.336%)**<br>• Paraglaciecola polaris [taxid 222814]: 41 (0.391%)<br>• Paraglaciecola mesophila [taxid 197222]: 19 (0.181%)<br>• Pseudoalteromonas atlantica [taxid 288]: 10 (0.095%)<br>• Ostreococcus mediterraneus [taxid 1486918]: 6 (0.057%)<br>• Paraglaciecola hydrolytica [taxid 1799789]: 5 (0.047%)<br>• other: 77 (0.734%)
Benchmark OTU ID: `CP001321-_Proteobacteria`<br>OTU taxon: Glaesserella parasuis SH0165 [taxid 557723]<br>Expected: Glaesserella parasuis [taxid 738] (species)<br>Number of reads: 4221<br>Number of identified reads: 4181 (99.052%)	• **species: 1695 (40.156%)**<br>• genus: 54 (1.279%)<br>• family: 1424 (33.736%)<br>• order: 0 (0.0%)<br>• class: 385 (9.121%)<br>• phylum: 95 (2.25%)<br>• superkingdom: 141 (3.34%)<br>• root: 384 (9.097%)	• Actinobacillus indolicus [taxid 51049]: 6 (0.142%)<br>• Avibacterium paragallinarum [taxid 728]: 4 (0.094%)<br>• Gallibacterium anatis [taxid 750]: 3 (0.071%)<br>• Actinobacillus minor [taxid 51047]: 3 (0.071%)<br>• Haemophilus influenzae [taxid 727]: 3 (0.071%)<br>• Glaesserella australis [taxid 2094024]: 3 (0.071%)<br>• Pasteurella bettyae [taxid 752]: 2 (0.047%)<br>• Muribacter muris [taxid 67855]: 2 (0.047%)<br>• other: 37 (0.876%)
Benchmark OTU ID: `BA000045-_Cyanobacteria`<br>OTU taxon: Gloeobacter violaceus PCC 7421 [taxid 251221]<br>Expected: Gloeobacter violaceus [taxid 33072] (species)<br>Number of reads: 26889<br>Number of identified reads: 26800 (99.669%)	• **species: 19729 (73.372%)**<br>• genus: 455 (1.692%)<br>• family: 0 (0.0%)<br>• order: 0 (0.0%)<br>• class: 0 (0.0%)<br>• phylum: 651 (2.421%)<br>• superkingdom: 2701 (10.044%)<br>• root: 3230 (12.012%)	• Gloeobacter kilaueensis [taxid 1416614]: 17 (0.063%)<br>• Phormidesmis priestleyi [taxid 268141]: 2 (0.007%)<br>• Avibacterium paragallinarum [taxid 728]: 1 (0.003%)<br>• Peptidiphaga gingivicola [taxid 2741497]: 1 (0.003%)<br>• Leptosphaeria maculans [taxid 5022]: 1 (0.003%)<br>• Myripristis murdjan [taxid 586833]: 1 (0.003%)<br>• bacterium [taxid 1869227]: 1 (0.003%)<br>• Halomicronema hongdechloris [taxid 1209493]: 1 (0.003%)<br>• other: 41 (0.152%)
Benchmark OTU ID: `CP003646-_Cyanobacteria`<br>OTU taxon: Gloeocapsa sp. PCC 7428 [taxid 1173026]<br>Expected: Gloeocapsa [taxid 102231] (genus)<br>Number of reads: 31987<br>Number of identified reads: 31710 (99.134%)	• **genus: 10998 (34.382%)**<br>• family: 4068 (12.717%)<br>• order: 1650 (5.158%)<br>• phylum: 8523 (26.645%)<br>• superkingdom: 2036 (6.365%)<br>• root: 4370 (13.661%)	• Chroogloeocystis siderophila [taxid 329163]: 86 (0.268%)<br>• Aliterella atlantica [taxid 1827278]: 8 (0.025%)<br>• Phormidesmis priestleyi [taxid 268141]: 6 (0.018%)<br>• Leptolyngbya sp. 'hensonii' [taxid 1922337]: 5 (0.015%)<br>• Oscillatoria acuminata [taxid 118323]: 4 (0.012%)<br>• Microcystis aeruginosa [taxid 1126]: 4 (0.012%)<br>• other: 155 (0.484%)

Operational Taxonomic Unit (OTU)	Correct identifications	Wrong or overspecific identifications at species rank
Benchmark OTU ID: `CP001291-_Cyanobacteria`<br>OTU taxon: Gloeothece citriformis PCC 7424 [taxid 65393]<br>Expected: Gloeothece citriformis [taxid 2546356] (species)<br>Number of reads: 35361<br>Number of identified reads: 34656 (98.006%)	• **species: 23232 (65.699%)**<br>• genus: 836 (2.364%)<br>• family: 182 (0.514%)<br>• order: 826 (2.335%)<br>• phylum: 3071 (8.684%)<br>• superkingdom: 1929 (5.455%)<br>• root: 4507 (12.745%)	• Gloeothece verrucosa [taxid 2546359]: 47 (0.132%)<br>• Aphanothece sacrum [taxid 1122]: 5 (0.014%)<br>• Aphanothece hegewaldii [taxid 1521625]: 5 (0.014%)<br>• Rippkaea orientalis [taxid 2546366]: 4 (0.011%)<br>• Cyanobacterium stanieri [taxid 102235]: 3 (0.008%)<br>• Crocosphaera watsonii [taxid 263511]: 3 (0.008%)<br>• Hyella patelloides [taxid 1982969]: 3 (0.008%)<br>• other: 97 (0.274%)
Benchmark OTU ID: `CP002198-_Cyanobacteria`<br>OTU taxon: Gloeothece verrucosa PCC 7822 [taxid 497965]<br>Expected: Gloeothece verrucosa [taxid 2546359] (species)<br>Number of reads: 36345<br>Number of identified reads: 35817 (98.547%)	• **species: 24408 (67.156%)**<br>• genus: 802 (2.206%)<br>• family: 176 (0.484%)<br>• order: 834 (2.294%)<br>• phylum: 2959 (8.141%)<br>• superkingdom: 2040 (5.612%)<br>• root: 4527 (12.455%)	• Gloeothece citriformis [taxid 2546356]: 33 (0.09%)<br>• Microcystis aeruginosa [taxid 1126]: 6 (0.016%)<br>• Crinalium epipsammum [taxid 241425]: 3 (0.008%)<br>• Mastigocoleus testarum [taxid 996925]: 3 (0.008%)<br>• Aphanothece sacrum [taxid 1122]: 3 (0.008%)<br>• Spirosoma montaniterrae [taxid 1178516]: 3 (0.008%)<br>• Entamoeba invadens [taxid 33085]: 2 (0.005%)<br>• other: 90 (0.247%)
Benchmark OTU ID: `CP001189-_Proteobacteria`<br>OTU taxon: Gluconacetobacter diazotrophicus PA1 5 [taxid 272568]<br>Expected: Gluconacetobacter diazotrophicus [taxid 33996] (species)<br>Number of reads: 7863<br>Number of identified reads: 7840 (99.707%)	• **species: 5011 (63.728%)**<br>• genus: 197 (2.505%)<br>• family: 849 (10.797%)<br>• order: 34 (0.432%)<br>• class: 373 (4.743%)<br>• phylum: 260 (3.306%)<br>• superkingdom: 390 (4.959%)<br>• root: 722 (9.182%)	• Gluconacetobacter liquefaciens [taxid 89584]: 6 (0.076%)<br>• Acetobacter aceti [taxid 435]: 5 (0.063%)<br>• Neoasaia chiangmaiensis [taxid 320497]: 4 (0.05%)<br>• Komagataeibacter rhaeticus [taxid 215221]: 3 (0.038%)<br>• Acetobacter cibinongensis [taxid 146475]: 2 (0.025%)<br>• Roseomonas cervicalis [taxid 204525]: 2 (0.025%)<br>• Acetobacter pomorum [taxid 65959]: 2 (0.025%)<br>• Acetobacter indonesiensis [taxid 104101]: 1 (0.012%)<br>• other: 32 (0.406%)
Benchmark OTU ID: `CP000009-_Proteobacteria`<br>OTU taxon: Gluconobacter oxydans 621H [taxid 290633]<br>Expected: Gluconobacter oxydans [taxid 442] (species)<br>Number of reads: 5196<br>Number of identified reads: 5184 (99.769%)	• species: 1506 (28.983%)<br>• **genus: 2146 (41.301%)**<br>• family: 491 (9.449%)<br>• order: 18 (0.346%)<br>• class: 148 (2.848%)<br>• phylum: 126 (2.424%)<br>• superkingdom: 172 (3.31%)<br>• root: 572 (11.008%)	• Gluconobacter kanchanaburiensis [taxid 563199]: 13 (0.25%)<br>• Gluconobacter albidus [taxid 318683]: 10 (0.192%)<br>• Gluconobacter sphaericus [taxid 574987]: 10 (0.192%)<br>• Gluconobacter morbifer [taxid 479935]: 9 (0.173%)<br>• Acetobacter aceti [taxid 435]: 5 (0.096%)<br>• Gluconobacter roseus [taxid 586239]: 4 (0.076%)<br>• Gluconobacter wancherniae [taxid 1307955]: 2 (0.038%)<br>• Gluconobacter frateurii [taxid 38308]: 2 (0.038%)<br>• other: 29 (0.558%)

Operational Taxonomic Unit (OTU)	Correct identifications	Wrong or overspecific identifications at species rank
Benchmark OTU ID: `CP003926-_Proteobacteria`<br>OTU taxon: Gluconobacter oxydans H24 [taxid 1224746]<br>Expected: Gluconobacter oxydans [taxid 442] (species)<br>Number of reads: 7221<br>Number of identified reads: 7201 (99.723%)	• species: 796 (11.023%)<br>• **genus: 3934 (54.479%)**<br>• family: 696 (9.638%)<br>• order: 20 (0.276%)<br>• class: 194 (2.686%)<br>• phylum: 167 (2.312%)<br>• superkingdom: 273 (3.78%)<br>• root: 1113 (15.413%)	• Gluconobacter frateurii [taxid 38308]: 70 (0.969%)<br>• Gluconobacter thailandicus [taxid 257438]: 58 (0.803%)<br>• Gluconobacter japonicus [taxid 376620]: 8 (0.11%)<br>• Gluconobacter morbifer [taxid 479935]: 6 (0.083%)<br>• Gluconobacter cerinus [taxid 38307]: 4 (0.055%)<br>• Gluconobacter wancherniae [taxid 1307955]: 4 (0.055%)<br>• Gluconobacter sp. Gdi [taxid 2691888]: 3 (0.041%)<br>• Gluconobacter kanchanaburiensis [taxid 563199]: 3 (0.041%)<br>• other: 51 (0.706%)
Benchmark OTU ID: `FQ311875-_Actinobacteria`<br>OTU taxon: Glutamicibacter arilaitensis Re117 [taxid 861360]<br>Expected: Glutamicibacter arilaitensis [taxid 256701] (species)<br>Number of reads: 16507<br>Number of identified reads: 16453 (99.672%)	• **species: 6754 (40.915%)**<br>• genus: 1068 (6.469%)<br>• family: 4306 (26.085%)<br>• order: 424 (2.568%)<br>• class: 794 (4.81%)<br>• phylum: 13 (0.078%)<br>• superkingdom: 878 (5.318%)<br>• root: 2199 (13.321%)	• Glutamicibacter mysorens [taxid 257984]: 8 (0.048%)<br>• Glutamicibacter nicotianae [taxid 37929]: 7 (0.042%)<br>• Glutamicibacter halophytocola [taxid 1933880]: 6 (0.036%)<br>• Glutamicibacter sp. HZAU [taxid 2049891]: 6 (0.036%)<br>• Glutamicibacter sp. ZJUTW [taxid 1155384]: 5 (0.03%)<br>• Glutamicibacter uratoxydans [taxid 43667]: 5 (0.03%)<br>• Brevibacterium aurantiacum [taxid 273384]: 4 (0.024%)<br>• Glutamicibacter mishrai [taxid 1775880]: 4 (0.024%)<br>• other: 74 (0.448%)
Benchmark OTU ID: `CP001802-_Actinobacteria`<br>OTU taxon: Gordonia bronchialis DSM 43247 [taxid 526226]<br>Expected: Gordonia bronchialis [taxid 2054] (species)<br>Number of reads: 23119<br>Number of identified reads: 23024 (99.589%)	• **species: 12628 (54.621%)**<br>• genus: 2059 (8.906%)<br>• family: 58 (0.25%)<br>• order: 2270 (9.818%)<br>• class: 1952 (8.443%)<br>• phylum: 19 (0.082%)<br>• superkingdom: 1414 (6.116%)<br>• root: 2601 (11.25%)	• Gordonia insulae [taxid 2420509]: 10 (0.043%)<br>• Gordonia rhizosphera [taxid 83341]: 10 (0.043%)<br>• Gordonia effusa [taxid 263908]: 10 (0.043%)<br>• Gordonia amarae [taxid 36821]: 8 (0.034%)<br>• Tetrasphaera australiensis [taxid 99480]: 8 (0.034%)<br>• Gordonia soli [taxid 320799]: 6 (0.025%)<br>• Gordonia otitidis [taxid 249058]: 6 (0.025%)<br>• Gordonia westfalica [taxid 158898]: 5 (0.021%)<br>• other: 158 (0.683%)
Benchmark OTU ID: `CP003119-_Actinobacteria`<br>OTU taxon: Gordonia polyisoprenivorans VH2 [taxid 1112204]<br>Expected: Gordonia polyisoprenivorans [taxid 84595] (species)<br>Number of reads: 25379<br>Number of identified reads: 25304 (99.704%)	• species: 5457 (21.502%)<br>• **genus: 12116 (47.74%)**<br>• family: 30 (0.118%)<br>• order: 1801 (7.096%)<br>• class: 1804 (7.108%)<br>• phylum: 13 (0.051%)<br>• superkingdom: 1358 (5.35%)<br>• root: 2698 (10.63%)	• Gordonia oryzae [taxid 2487349]: 48 (0.189%)<br>• Gordonia rhizosphera [taxid 83341]: 15 (0.059%)<br>• Gordonia otitidis [taxid 249058]: 9 (0.035%)<br>• Gordonia amarae [taxid 36821]: 9 (0.035%)<br>• Gordonia bronchialis [taxid 2054]: 8 (0.031%)<br>• Gordonia effusa [taxid 263908]: 7 (0.027%)<br>• Gordonia hirsuta [taxid 53427]: 6 (0.023%)<br>• Gordonia insulae [taxid 2420509]: 6 (0.023%)<br>• other: 119 (0.468%)

Operational Taxonomic Unit (OTU)	Correct identifications	Wrong or overspecific identifications at species rank
Benchmark OTU ID: `CP002907-_Actinobacteria`<br>OTU taxon: Gordonia sp. KTR9 [taxid 337191]<br>Expected: Gordonia [taxid 2053] (genus)<br>Number of reads: 24259<br>Number of identified reads: 24182 (99.682%)	• **genus: 14873 (61.309%)**<br>• family: 289 (1.191%)<br>• order: 3475 (14.324%)<br>• class: 1606 (6.62%)<br>• phylum: 16 (0.065%)<br>• superkingdom: 1267 (5.222%)<br>• root: 2608 (10.75%)	• Gordonia terrae [taxid 2055]: 281 (1.158%)<br>• Gordonia lacunae [taxid 417102]: 28 (0.115%)<br>• Gordonia paraffinivorans [taxid 175628]: 21 (0.086%)<br>• Gordonia bronchialis [taxid 2054]: 17 (0.07%)<br>• Gordonia westfalica [taxid 158898]: 13 (0.053%)<br>• Gordonia rubripertincta [taxid 36822]: 11 (0.045%)<br>• Gordonia iterans [taxid 1004901]: 7 (0.028%)<br>• other: 165 (0.68%)
Benchmark OTU ID: `FP929047-_Actinobacteria`<br>OTU taxon: Gordonibacter pamelaeae 7-10-1-b [taxid 657308]<br>Expected: Gordonibacter pamelaeae [taxid 471189] (species)<br>Number of reads: 12499<br>Number of identified reads: 12430 (99.447%)	• **species: 4381 (35.05%)**<br>• genus: 2941 (23.529%)<br>• family: 1565 (12.521%)<br>• order: 2 (0.016%)<br>• class: 904 (7.232%)<br>• phylum: 280 (2.24%)<br>• superkingdom: 1102 (8.816%)<br>• root: 1244 (9.952%)	• Gordonibacter urolithinfaciens [taxid 1335613]: 78 (0.624%)<br>• Eggerthella lenta [taxid 84112]: 14 (0.112%)<br>• Slackia isoflavoniconvertens [taxid 572010]: 6 (0.048%)<br>• Paraeggerthella hongkongensis [taxid 230658]: 6 (0.048%)<br>• Collinsella aerofaciens [taxid 74426]: 5 (0.04%)<br>• Denitrobacterium detoxificans [taxid 79604]: 4 (0.032%)<br>• Enteroscipio rubneri [taxid 2070686]: 4 (0.032%)<br>• Eggerthella sinensis [taxid 242230]: 3 (0.024%)<br>• other: 39 (0.312%)
Benchmark OTU ID: `CU207366-_Bacteroidetes`<br>OTU taxon: Gramella forsetii KT0803 [taxid 411154]<br>Expected: Gramella forsetii [taxid 411153] (species)<br>Number of reads: 25057<br>Number of identified reads: 24757 (98.802%)	• **species: 13955 (55.693%)**<br>• genus: 2820 (11.254%)<br>• family: 3456 (13.792%)<br>• order: 260 (1.037%)<br>• class: 6 (0.023%)<br>• phylum: 806 (3.216%)<br>• superkingdom: 1012 (4.038%)<br>• root: 2430 (9.697%)	• Gramella echinicola [taxid 279359]: 12 (0.047%)<br>• Gramella gaetbulicola [taxid 703340]: 11 (0.043%)<br>• Gramella sabulilitoris [taxid 2583991]: 10 (0.039%)<br>• Gramella fulva [taxid 2126553]: 9 (0.035%)<br>• Salegentibacter echinorum [taxid 1073325]: 5 (0.019%)<br>• Gramella salexigens [taxid 1913577]: 4 (0.015%)<br>• Gramella flava [taxid 1486245]: 3 (0.011%)<br>• Salegentibacter flavus [taxid 287099]: 3 (0.011%)<br>• other: 94 (0.375%)
Benchmark OTU ID: `CP000394-_Proteobacteria`<br>OTU taxon: Granulibacter bethesdensis CGDNIH1 [taxid 391165]<br>Expected: Granulibacter bethesdensis [taxid 364410] (species)<br>Number of reads: 5210<br>Number of identified reads: 5199 (99.788%)	• **species: 3663 (70.307%)**<br>• genus: 0 (0.0%)<br>• family: 129 (2.476%)<br>• order: 48 (0.921%)<br>• class: 288 (5.527%)<br>• phylum: 200 (3.838%)<br>• superkingdom: 227 (4.357%)<br>• root: 633 (12.149%)	• Rhipicephalus microplus [taxid 6941]: 1 (0.019%)<br>• Prorocentrum minimum [taxid 39449]: 1 (0.019%)<br>• Rhodopila globiformis [taxid 1071]: 1 (0.019%)<br>• Noctiluca scintillans [taxid 2966]: 1 (0.019%)<br>• Gluconacetobacter liquefaciens [taxid 89584]: 1 (0.019%)<br>• Verticiella sediminum [taxid 1247510]: 1 (0.019%)<br>• Paramormyrops kingsleyae [taxid 1676925]: 1 (0.019%)<br>• Acetobacter orientalis [taxid 146474]: 1 (0.019%)<br>• other: 9 (0.172%)

Operational Taxonomic Unit (OTU)	Correct identifications	Wrong or overspecific identifications at species rank
Benchmark OTU ID: `CP003130-_Acidobacteria`<br>OTU taxon: Granulicella mallensis MP5ACTX8 [taxid 682795]<br>Expected: Granulicella mallensis [taxid 940614] (species)<br>Number of reads: 504207<br>Number of identified reads: 502877 (99.736%)	• **species: 352552 (69.922%)**<br>• genus: 7197 (1.427%)<br>• family: 15624 (3.098%)<br>• order: 2269 (0.45%)<br>• class: 370 (0.073%)<br>• phylum: 4777 (0.947%)<br>• superkingdom: 47842 (9.488%)<br>• root: 71723 (14.224%)	• Terriglobus saanensis [taxid 870903]: 70 (0.013%)<br>• Terriglobus roseus [taxid 392734]: 68 (0.013%)<br>• Edaphobacter modestus [taxid 388466]: 65 (0.012%)<br>• Bryocella elongata [taxid 863522]: 55 (0.01%)<br>• Granulicella sibirica [taxid 2479048]: 44 (0.008%)<br>• Terriglobus albidus [taxid 1592106]: 43 (0.008%)<br>• Granulicella tundricola [taxid 940615]: 42 (0.008%)<br>• Granulicella rosea [taxid 474952]: 41 (0.008%)<br>• other: 1095 (0.217%)
Benchmark OTU ID: `CP002480-_Acidobacteria`<br>OTU taxon: Granulicella tundricola MP5ACTX9 [taxid 1198114]<br>Expected: Granulicella tundricola [taxid 940615] (species)<br>Number of reads: 331036<br>Number of identified reads: 329642 (99.578%)	• **species: 229062 (69.195%)**<br>• genus: 7832 (2.365%)<br>• family: 9424 (2.846%)<br>• order: 2511 (0.758%)<br>• class: 256 (0.077%)<br>• phylum: 3444 (1.04%)<br>• superkingdom: 31612 (9.549%)<br>• root: 45007 (13.595%)	• Granulicella sibirica [taxid 2479048]: 155 (0.046%)<br>• Granulicella rosea [taxid 474952]: 105 (0.031%)<br>• Acidisarcina polymorpha [taxid 2211140]: 69 (0.02%)<br>• Granulicella pectinivorans [taxid 474950]: 57 (0.017%)<br>• Terriglobus saanensis [taxid 870903]: 48 (0.014%)<br>• Terriglobus roseus [taxid 392734]: 37 (0.011%)<br>• Edaphobacter modestus [taxid 388466]: 33 (0.009%)<br>• Bryocella elongata [taxid 863522]: 32 (0.009%)<br>• other: 770 (0.232%)
Benchmark OTU ID: `FQ670178-_Pathogens`<br>OTU taxon: Haemophilus influenzae F3031 [taxid 866630]<br>Expected: Haemophilus influenzae [taxid 727] (species)<br>Number of reads: 3751<br>Number of identified reads: 3724 (99.28%)	• species: 1052 (28.045%)<br>• **genus: 1396 (37.216%)**<br>• family: 571 (15.222%)<br>• order: 0 (0.0%)<br>• class: 229 (6.105%)<br>• phylum: 75 (1.999%)<br>• superkingdom: 114 (3.039%)<br>• root: 283 (7.544%)	• Haemophilus haemolyticus [taxid 726]: 13 (0.346%)<br>• Haemophilus aegyptius [taxid 197575]: 10 (0.266%)<br>• Haemophilus parainfluenzae [taxid 729]: 4 (0.106%)<br>• Pasteurella multocida [taxid 747]: 2 (0.053%)<br>• Vibrio campbellii [taxid 680]: 2 (0.053%)<br>• Rodentibacter heylii [taxid 1906744]: 1 (0.026%)<br>• Cricetibacter osteomyelitidis [taxid 1521931]: 1 (0.026%)<br>• Halomonas titanicae [taxid 664683]: 1 (0.026%)<br>• other: 17 (0.453%)
Benchmark OTU ID: `CP000671-_Pathogens`<br>OTU taxon: Haemophilus influenzae PittEE [taxid 374930]<br>Expected: Haemophilus influenzae [taxid 727] (species)<br>Number of reads: 3319<br>Number of identified reads: 3294 (99.246%)	• species: 925 (27.869%)<br>• **genus: 1135 (34.197%)**<br>• family: 588 (17.716%)<br>• order: 0 (0.0%)<br>• class: 235 (7.08%)<br>• phylum: 80 (2.41%)<br>• superkingdom: 90 (2.711%)<br>• root: 233 (7.02%)	• Haemophilus haemolyticus [taxid 726]: 8 (0.241%)<br>• Haemophilus parahaemolyticus [taxid 735]: 5 (0.15%)<br>• Avibacterium paragallinarum [taxid 728]: 4 (0.12%)<br>• Haemophilus parainfluenzae [taxid 729]: 3 (0.09%)<br>• Clonorchis sinensis [taxid 79923]: 3 (0.09%)<br>• Rodentibacter pneumotropicus [taxid 758]: 2 (0.06%)<br>• Photinus pyralis [taxid 7054]: 2 (0.06%)<br>• Pararhodospirillum photometricum [taxid 1084]: 2 (0.06%)<br>• other: 31 (0.934%)

Operational Taxonomic Unit (OTU)	Correct identifications	Wrong or overspecific identifications at species rank
Benchmark OTU ID: `CP000672-_Pathogens`<br>OTU taxon: Haemophilus influenzae PittGG [taxid 374931]<br>Expected: Haemophilus influenzae [taxid 727] (species)<br>Number of reads: 3503<br>Number of identified reads: 3470 (99.057%)	• species: 973 (27.776%)<br>• **genus: 1223 (34.912%)**<br>• family: 615 (17.556%)<br>• order: 0 (0.0%)<br>• class: 245 (6.994%)<br>• phylum: 90 (2.569%)<br>• superkingdom: 95 (2.711%)<br>• root: 221 (6.308%)	• Haemophilus haemolyticus [taxid 726]: 13 (0.371%)<br>• Haemophilus pittmaniae [taxid 249188]: 3 (0.085%)<br>• Haemophilus parainfluenzae [taxid 729]: 3 (0.085%)<br>• Avibacterium paragallinarum [taxid 728]: 3 (0.085%)<br>• Haemophilus paraphrohaemolyticus [taxid 736]: 2 (0.057%)<br>• Actinobacillus delphinicola [taxid 51161]: 2 (0.057%)<br>• Escherichia coli [taxid 562]: 2 (0.057%)<br>• Volucribacter psittacicida [taxid 203482]: 1 (0.028%)<br>• other: 23 (0.656%)
Benchmark OTU ID: `FQ312002-_Proteobacteria`<br>OTU taxon: Haemophilus parainfluenzae T3T1 [taxid 862965]<br>Expected: Haemophilus parainfluenzae [taxid 729] (species)<br>Number of reads: 3811<br>Number of identified reads: 3792 (99.501%)	• species: 755 (19.811%)<br>• **genus: 1265 (33.193%)**<br>• family: 919 (24.114%)<br>• order: 0 (0.0%)<br>• class: 339 (8.895%)<br>• phylum: 85 (2.23%)<br>• superkingdom: 158 (4.145%)<br>• root: 263 (6.901%)	• Haemophilus influenzae [taxid 727]: 7 (0.183%)<br>• Haemophilus haemolyticus [taxid 726]: 6 (0.157%)<br>• Escherichia coli [taxid 562]: 4 (0.104%)<br>• Photinus pyralis [taxid 7054]: 4 (0.104%)<br>• Haemophilus pittmaniae [taxid 249188]: 3 (0.078%)<br>• Actinobacillus succinogenes [taxid 67854]: 3 (0.078%)<br>• Arsenophonus endosymbiont of Aleurodicus dispersus [taxid 235559]: 2 (0.052%)<br>• Rodentibacter myodis [taxid 1907939]: 2 (0.052%)<br>• other: 37 (0.97%)
Benchmark OTU ID: `CP000155-_Proteobacteria`<br>OTU taxon: Hahella chejuensis KCTC 2396 [taxid 349521]<br>Expected: Hahella chejuensis [taxid 158327] (species)<br>Number of reads: 15350<br>Number of identified reads: 15298 (99.661%)	• **species: 7590 (49.446%)**<br>• genus: 3835 (24.983%)<br>• family: 9 (0.058%)<br>• order: 93 (0.605%)<br>• class: 922 (6.006%)<br>• phylum: 462 (3.009%)<br>• superkingdom: 535 (3.485%)<br>• root: 1839 (11.98%)	• Marinomonas gallaica [taxid 1806667]: 3 (0.019%)<br>• Pandoraea vervacti [taxid 656178]: 1 (0.006%)<br>• Oleiphilus messinensis [taxid 141451]: 1 (0.006%)<br>• Endozoicomonas montiporae [taxid 1027273]: 1 (0.006%)<br>• Fistulifera solaris [taxid 1519565]: 1 (0.006%)<br>• Escherichia coli [taxid 562]: 1 (0.006%)<br>• Streptomyces hundungensis [taxid 1077946]: 1 (0.006%)<br>• Pseudomonas jessenii [taxid 77298]: 1 (0.006%)<br>• other: 40 (0.26%)
Benchmark OTU ID: `Haliaeetus_leucocephalus_scaffold3-_Eukaryotes`<br>OTU taxon: Haliaeetus leucocephalus [taxid 52644]<br>Expected: Haliaeetus leucocephalus [taxid 52644] (species)<br>Number of reads: 242103<br>Number of identified reads: 224378 (92.678%)	• species: 0 (0.0%)<br>• genus: 195 (0.08%)<br>• family: 363 (0.149%)<br>• order: 33 (0.013%)<br>• class: 3229 (1.333%)<br>• phylum: 2534 (1.046%)<br>• superkingdom: 2044 (0.844%)<br>• **root: 212523 (87.782%)**	• Limosa lapponica [taxid 161683]: 366 (0.151%)<br>• Aquila chrysaetos [taxid 8962]: 230 (0.095%)<br>• Haliaeetus albicilla [taxid 8969]: 195 (0.08%)<br>• Athene cunicularia [taxid 194338]: 41 (0.016%)<br>• Strigops habroptila [taxid 2489341]: 34 (0.014%)<br>• Cathartes aura [taxid 43455]: 27 (0.011%)<br>• Anas platyrhynchos [taxid 8839]: 26 (0.01%)<br>• Patagioenas fasciata [taxid 372321]: 22 (0.009%)<br>• other: 1344 (0.555%)

Operational Taxonomic Unit (OTU)	Correct identifications	Wrong or overspecific identifications at species rank
Benchmark OTU ID: `CP001804-_Proteobacteria`<br>OTU taxon: Haliangium ochraceum DSM 14365 [taxid 502025]<br>Expected: Haliangium ochraceum [taxid 80816] (species)<br>Number of reads: 20370<br>Number of identified reads: 20276 (99.538%)	• **species: 16041 (78.748%)**<br>• genus: 0 (0.0%)<br>• family: 1 (0.004%)<br>• order: 74 (0.363%)<br>• class: 99 (0.486%)<br>• phylum: 625 (3.068%)<br>• superkingdom: 1332 (6.539%)<br>• root: 2078 (10.201%)	• Flavobacterium aquidurense [taxid 362413]: 1 (0.004%)<br>• Yersinia kristensenii [taxid 28152]: 1 (0.004%)<br>• Thermotoga naphthophila [taxid 93930]: 1 (0.004%)<br>• Burkholderia pseudomallei [taxid 28450]: 1 (0.004%)<br>• Providencia rettgeri [taxid 587]: 1 (0.004%)<br>• Phlebotomus kandelakii [taxid 1109342]: 1 (0.004%)<br>• Phytohabitans flavus [taxid 1076124]: 1 (0.004%)<br>• Stenotrophomonas humi [taxid 405444]: 1 (0.004%)<br>• other: 32 (0.157%)
Benchmark OTU ID: `CP002691-_Bacteroidetes`<br>OTU taxon: Haliscomenobacter hydrossis DSM 1100 [taxid 760192]<br>Expected: Haliscomenobacter hydrossis [taxid 2350] (species)<br>Number of reads: 59813<br>Number of identified reads: 59681 (99.779%)	• **species: 45913 (76.76%)**<br>• genus: 0 (0.0%)<br>• family: 36 (0.06%)<br>• order: 113 (0.188%)<br>• class: 0 (0.0%)<br>• phylum: 2935 (4.906%)<br>• superkingdom: 2897 (4.843%)<br>• root: 7735 (12.931%)	• Asterionellopsis glacialis [taxid 33640]: 2 (0.003%)<br>• Dyadobacter luticola [taxid 1979387]: 2 (0.003%)<br>• Massilia buxea [taxid 1949069]: 2 (0.003%)<br>• Flavilitoribacter nigricans [taxid 70997]: 2 (0.003%)<br>• Mucilaginibacter yixingensis [taxid 1295612]: 2 (0.003%)<br>• Pedobacter rhizosphaerae [taxid 390241]: 2 (0.003%)<br>• Flavobacterium aurantiibacter [taxid 2023067]: 2 (0.003%)<br>• Marinoscillum furvescens [taxid 1026]: 2 (0.003%)<br>• other: 99 (0.165%)
Benchmark OTU ID: `AY596297-_Euryarchaeota`<br>OTU taxon: Haloarcula marismortui ATCC 43049 [taxid 272569]<br>Expected: Haloarcula marismortui [taxid 2238] (species)<br>Number of reads: 2891<br>Number of identified reads: 2873 (99.377%)	• species: 183 (6.329%)<br>• **genus: 1658 (57.35%)**<br>• family: 30 (1.037%)<br>• order: 85 (2.94%)<br>• class: 185 (6.399%)<br>• phylum: 3 (0.103%)<br>• superkingdom: 3 (0.103%)<br>• root: 685 (23.694%)	• Haloarcula argentinensis [taxid 43776]: 7 (0.242%)<br>• Haloarcula quadrata [taxid 182779]: 6 (0.207%)<br>• Haloarcula vallismortis [taxid 28442]: 3 (0.103%)<br>• Natrinema versiforme [taxid 88724]: 2 (0.069%)<br>• Haloarcula californiae [taxid 244363]: 2 (0.069%)<br>• Haloquadratum walsbyi [taxid 293091]: 1 (0.034%)<br>• Natrarchaeobius chitinivorans [taxid 1679083]: 1 (0.034%)<br>• Haloarcula amylolytica [taxid 396317]: 1 (0.034%)<br>• other: 11 (0.38%)
Benchmark OTU ID: `FQ312005-_Proteobacteria`<br>OTU taxon: Halobacteriovorax marinus SJ [taxid 862908]<br>Expected: Halobacteriovorax marinus [taxid 97084] (species)<br>Number of reads: 6847<br>Number of identified reads: 6784 (99.079%)	• **species: 5117 (74.733%)**<br>• genus: 439 (6.411%)<br>• family: 23 (0.335%)<br>• order: 58 (0.847%)<br>• class: 49 (0.715%)<br>• phylum: 171 (2.497%)<br>• superkingdom: 280 (4.089%)<br>• root: 646 (9.434%)	• Thiorhodospira sibirica [taxid 154347]: 1 (0.014%)<br>• Desulfococcus multivorans [taxid 897]: 1 (0.014%)<br>• Arcobacter venerupis [taxid 1054033]: 1 (0.014%)<br>• Desulfovibrio magneticus [taxid 184917]: 1 (0.014%)<br>• Campylobacter showae [taxid 204]: 1 (0.014%)<br>• Flavobacterium segetis [taxid 271157]: 1 (0.014%)

Operational Taxonomic Unit (OTU)	Correct identifications	Wrong or overspecific identifications at species rank
Benchmark OTU ID: `AE004437-_Euryarchaeota`<br>OTU taxon: Halobacterium salinarum NRC-1 [taxid 64091]<br>Expected: Halobacterium salinarum [taxid 2242] (species)<br>Number of reads: 1718<br>Number of identified reads: 1708 (99.417%)	• **species: 983 (57.217%)**<br>• genus: 64 (3.725%)<br>• family: 15 (0.873%)<br>• order: 37 (2.153%)<br>• class: 117 (6.81%)<br>• phylum: 4 (0.232%)<br>• superkingdom: 3 (0.174%)<br>• root: 461 (26.833%)	• Natrinema pellirubrum [taxid 69525]: 1 (0.058%)<br>• Halorubrum ezzemoulense [taxid 337243]: 1 (0.058%)<br>• Haloarcula argentinensis [taxid 43776]: 1 (0.058%)<br>• Haloquadratum walsbyi [taxid 293091]: 1 (0.058%)<br>• Nocardioides dokdonensis [taxid 450734]: 1 (0.058%)<br>• Halorubrum vacuolatum [taxid 63740]: 1 (0.058%)<br>• halophilic archaeon [taxid 29295]: 1 (0.058%)<br>• Bigelowiella natans [taxid 227086]: 1 (0.058%)
Benchmark OTU ID: `FN869568-_Proteobacteria`<br>OTU taxon: Halomonas elongata DSM 2581 [taxid 768066]<br>Expected: Halomonas elongata [taxid 2746] (species)<br>Number of reads: 8254<br>Number of identified reads: 8225 (99.648%)	• species: 2883 (34.928%)<br>• **genus: 2900 (35.134%)**<br>• family: 415 (5.027%)<br>• order: 47 (0.569%)<br>• class: 549 (6.651%)<br>• phylum: 354 (4.288%)<br>• superkingdom: 337 (4.082%)<br>• root: 738 (8.941%)	• Halomonas halmophila [taxid 252]: 15 (0.181%)<br>• Halomonas caseinilytica [taxid 438744]: 14 (0.169%)<br>• Halomonas eurihalina [taxid 42566]: 13 (0.157%)<br>• Halomonas beimenensis [taxid 475662]: 4 (0.048%)<br>• Halomonas endophytica [taxid 2033802]: 3 (0.036%)<br>• Halomonas pacifica [taxid 77098]: 3 (0.036%)<br>• Halomonas titanicae [taxid 664683]: 3 (0.036%)<br>• Halomonas aestuarii [taxid 1897729]: 2 (0.024%)<br>• other: 43 (0.52%)
Benchmark OTU ID: `CP002839-_Euryarchaeota`<br>OTU taxon: Halopiger xanaduensis SH-6 [taxid 797210]<br>Expected: Halopiger xanaduensis [taxid 387343] (species)<br>Number of reads: 3454<br>Number of identified reads: 3441 (99.623%)	• **species: 1266 (36.653%)**<br>• genus: 360 (10.422%)<br>• family: 575 (16.647%)<br>• order: 0 (0.0%)<br>• class: 271 (7.845%)<br>• phylum: 2 (0.057%)<br>• superkingdom: 6 (0.173%)<br>• root: 902 (26.114%)	• Halopiger aswanensis [taxid 148449]: 8 (0.231%)<br>• Natrinema ejinorense [taxid 373386]: 2 (0.057%)<br>• Natronorubrum texcoconense [taxid 1095776]: 2 (0.057%)<br>• Natrarchaeobius halalkaliphilus [taxid 1679091]: 2 (0.057%)<br>• Haloplanus vescus [taxid 555874]: 2 (0.057%)<br>• Natrarchaeobius chitinivorans [taxid 1679083]: 2 (0.057%)<br>• Natrarchaeobaculum aegyptiacum [taxid 745377]: 2 (0.057%)<br>• Natrinema versiforme [taxid 88724]: 2 (0.057%)<br>• other: 17 (0.492%)
Benchmark OTU ID: `FR746099-_Euryarchaeota`<br>OTU taxon: Haloquadratum walsbyi C23 [taxid 768065]<br>Expected: Haloquadratum walsbyi [taxid 293091] (species)<br>Number of reads: 2908<br>Number of identified reads: 2898 (99.656%)	• **species: 1518 (52.2%)**<br>• genus: 9 (0.309%)<br>• family: 43 (1.478%)<br>• order: 54 (1.856%)<br>• class: 343 (11.795%)<br>• phylum: 4 (0.137%)<br>• superkingdom: 4 (0.137%)<br>• root: 886 (30.467%)	• Halomicrobium zhouii [taxid 767519]: 1 (0.034%)<br>• Marininema mesophilum [taxid 1048340]: 1 (0.034%)<br>• Haloplanus vescus [taxid 555874]: 1 (0.034%)<br>• Syntrophus sp. (in: Bacteria) [taxid 48412]: 1 (0.034%)<br>• Novipirellula galeiformis [taxid 2528004]: 1 (0.034%)<br>• Halorubrum vacuolatum [taxid 63740]: 1 (0.034%)<br>• Halobaculum gomorrense [taxid 43928]: 1 (0.034%)<br>• Halogeometricum rufum [taxid 553469]: 1 (0.034%)<br>• other: 3 (0.103%)

Operational Taxonomic Unit (OTU)	Correct identifications	Wrong or overspecific identifications at species rank
Benchmark OTU ID: `AM180088-_Euryarchaeota`<br>OTU taxon: Haloquadratum walsbyi DSM 16790 [taxid 362976]<br>Expected: Haloquadratum walsbyi [taxid 293091] (species)<br>Number of reads: 2892<br>Number of identified reads: 2878 (99.515%)	• **species: 1490 (51.521%)**<br>• genus: 6 (0.207%)<br>• family: 52 (1.798%)<br>• order: 44 (1.521%)<br>• class: 310 (10.719%)<br>• phylum: 5 (0.172%)<br>• superkingdom: 2 (0.069%)<br>• root: 930 (32.157%)	• Halovenus aranensis [taxid 890420]: 2 (0.069%)<br>• Salmonella enterica [taxid 28901]: 1 (0.034%)<br>• Halanaeroarchaeum sulfurireducens [taxid 1604004]: 1 (0.034%)<br>• Halorubrum chaoviator [taxid 335819]: 1 (0.034%)<br>• Halobellus limi [taxid 699433]: 1 (0.034%)<br>• Halorubrum aquaticum [taxid 387340]: 1 (0.034%)<br>• Natronorubrum thiooxidans [taxid 308853]: 1 (0.034%)<br>• Vagococcus carniphilus [taxid 218144]: 1 (0.034%)<br>• other: 14 (0.484%)
Benchmark OTU ID: `CP000544-_Proteobacteria`<br>OTU taxon: Halorhodospira halophila SL1 [taxid 349124]<br>Expected: Halorhodospira halophila [taxid 1053] (species)<br>Number of reads: 5142<br>Number of identified reads: 5112 (99.416%)	• **species: 3686 (71.684%)**<br>• genus: 71 (1.38%)<br>• family: 28 (0.544%)<br>• order: 12 (0.233%)<br>• class: 237 (4.609%)<br>• phylum: 273 (5.309%)<br>• superkingdom: 291 (5.659%)<br>• root: 507 (9.859%)	• Salmonella enterica [taxid 28901]: 2 (0.038%)<br>• Methylomicrobium buryatense [taxid 95641]: 1 (0.019%)<br>• Halalkalicoccus paucihalophilus [taxid 1008153]: 1 (0.019%)<br>• Pararobbsia silviterrae [taxid 1792498]: 1 (0.019%)<br>• Thiothrix nivea [taxid 1031]: 1 (0.019%)<br>• Maridesulfovibrio hydrothermalis [taxid 191026]: 1 (0.019%)<br>• Archangium gephyra [taxid 48]: 1 (0.019%)<br>• Methylobacterium dankookense [taxid 560405]: 1 (0.019%)<br>• other: 12 (0.233%)
Benchmark OTU ID: `CP001365-_Euryarchaeota`<br>OTU taxon: Halorubrum lacusprofundi ATCC 49239 [taxid 416348]<br>Expected: Halorubrum lacusprofundi [taxid 2247] (species)<br>Number of reads: 2475<br>Number of identified reads: 2459 (99.353%)	• **species: 802 (32.404%)**<br>• genus: 694 (28.04%)<br>• family: 14 (0.565%)<br>• order: 48 (1.939%)<br>• class: 183 (7.393%)<br>• phylum: 2 (0.08%)<br>• superkingdom: 1 (0.04%)<br>• root: 680 (27.474%)	• Haloquadratum walsbyi [taxid 293091]: 2 (0.08%)<br>• Nakamurella multipartita [taxid 53461]: 2 (0.08%)<br>• Halorubrum ezzemoulense [taxid 337243]: 2 (0.08%)<br>• Halorubrum saccharovorum [taxid 2248]: 2 (0.08%)<br>• Halorubrum vacuolatum [taxid 63740]: 2 (0.08%)<br>• Halorubrum lipolyticum [taxid 368624]: 1 (0.04%)<br>• Halorubrum persicum [taxid 1383844]: 1 (0.04%)<br>• Halolamina pelagica [taxid 699431]: 1 (0.04%)<br>• other: 5 (0.202%)
Benchmark OTU ID: `CP001860-_Euryarchaeota`<br>OTU taxon: Haloterrigena turkmenica DSM 5511 [taxid 543526]<br>Expected: Haloterrigena turkmenica [taxid 62320] (species)<br>Number of reads: 3686<br>Number of identified reads: 3665 (99.43%)	• **species: 1313 (35.621%)**<br>• genus: 350 (9.495%)<br>• family: 656 (17.797%)<br>• order: 0 (0.0%)<br>• class: 296 (8.03%)<br>• phylum: 5 (0.135%)<br>• superkingdom: 3 (0.081%)<br>• root: 969 (26.288%)	• Natrarchaeobius chitinivorans [taxid 1679083]: 2 (0.054%)<br>• Natronolimnohabitans innermongolicus [taxid 253107]: 2 (0.054%)<br>• Natronomonas salsuginis [taxid 2217661]: 2 (0.054%)<br>• Mola mola [taxid 94237]: 1 (0.027%)<br>• Halopiger salifodinae [taxid 1202768]: 1 (0.027%)<br>• Halalkaliarchaeum desulfuricum [taxid 2055893]: 1 (0.027%)<br>• Haloterrigena hispanica [taxid 392421]: 1 (0.027%)<br>• Natronorubrum sulfidifaciens [taxid 388259]: 1 (0.027%)<br>• other: 24 (0.651%)

Operational Taxonomic Unit (OTU)	Correct identifications	Wrong or overspecific identifications at species rank
Benchmark OTU ID: `CP003945-_Cyanobacteria`<br>OTU taxon: Halothece sp. PCC 7418 [taxid 65093]<br>Expected: Halothece [taxid 76023] (genus)<br>Number of reads: 23722<br>Number of identified reads: 23509 (99.102%)	• **genus: 15337 (64.653%)**<br>• family: 705 (2.971%)<br>• order: 125 (0.526%)<br>• phylum: 2897 (12.212%)<br>• superkingdom: 1485 (6.26%)<br>• root: 2903 (12.237%)	• Euhalothece natronophila [taxid 577489]: 10 (0.042%)<br>• Dactylococcopsis salina [taxid 292566]: 9 (0.037%)<br>• Microcystis aeruginosa [taxid 1126]: 3 (0.012%)<br>• Rubidibacter lacunae [taxid 582514]: 3 (0.012%)<br>• Limnospira maxima [taxid 129910]: 2 (0.008%)<br>• Oscillatoria acuminata [taxid 118323]: 2 (0.008%)<br>• other: 71 (0.299%)
Benchmark OTU ID: `CP001098-_Firmicutes`<br>OTU taxon: Halothermothrix orenii H 168 [taxid 373903]<br>Expected: Halothermothrix orenii [taxid 31909] (species)<br>Number of reads: 3356<br>Number of identified reads: 3285 (97.884%)	• **species: 2424 (72.228%)**<br>• genus: 0 (0.0%)<br>• family: 21 (0.625%)<br>• order: 7 (0.208%)<br>• class: 37 (1.102%)<br>• phylum: 81 (2.413%)<br>• superkingdom: 253 (7.538%)<br>• root: 460 (13.706%)	• Natranaerobius trueperi [taxid 759412]: 2 (0.059%)<br>• Paenibacillus polymyxa [taxid 1406]: 1 (0.029%)<br>• Enterococcus mundtii [taxid 53346]: 1 (0.029%)<br>• Muribacter muris [taxid 67855]: 1 (0.029%)<br>• Clostridium collagenovorans [taxid 29357]: 1 (0.029%)<br>• Lupinus albus [taxid 3870]: 1 (0.029%)<br>• Lactococcus fujiensis [taxid 610251]: 1 (0.029%)<br>• Oceanotoga teriensis [taxid 515440]: 1 (0.029%)<br>• other: 2 (0.059%)
Benchmark OTU ID: `FR871757-_Proteobacteria`<br>OTU taxon: Helicobacter bizzozeronii CIII-1 [taxid 1002804]<br>Expected: Helicobacter bizzozeronii [taxid 56877] (species)<br>Number of reads: 3050<br>Number of identified reads: 3026 (99.213%)	• **species: 1538 (50.426%)**<br>• genus: 977 (32.032%)<br>• family: 1 (0.032%)<br>• order: 39 (1.278%)<br>• class: 1 (0.032%)<br>• phylum: 46 (1.508%)<br>• superkingdom: 85 (2.786%)<br>• root: 337 (11.049%)	• **Helicobacter pylori [taxid 210]: 153 (5.016%)**<br>• Helicobacter felis [taxid 214]: 5 (0.163%)<br>• Helicobacter ailurogastricus [taxid 1578720]: 2 (0.065%)<br>• Helicobacter heilmannii [taxid 35817]: 2 (0.065%)<br>• Helicobacter mustelae [taxid 217]: 2 (0.065%)<br>• Helicobacter salomonis [taxid 56878]: 2 (0.065%)<br>• Sulfurospirillum halorespirans [taxid 194424]: 1 (0.032%)<br>• Wolinella succinogenes [taxid 844]: 1 (0.032%)<br>• other: 6 (0.196%)
Benchmark OTU ID: `CP003479-_Proteobacteria`<br>OTU taxon: Helicobacter cetorum MIT 00-7128 [taxid 182217]<br>Expected: Helicobacter cetorum [taxid 138563] (species)<br>Number of reads: 3498<br>Number of identified reads: 3422 (97.827%)	• species: 1354 (38.707%)<br>• **genus: 1584 (45.283%)**<br>• family: 1 (0.028%)<br>• order: 30 (0.857%)<br>• class: 1 (0.028%)<br>• phylum: 54 (1.543%)<br>• superkingdom: 80 (2.287%)<br>• root: 316 (9.033%)	• **Helicobacter pylori [taxid 210]: 396 (11.32%)**<br>• Moraxella lacunata [taxid 477]: 1 (0.028%)<br>• Wolinella succinogenes [taxid 844]: 1 (0.028%)<br>• Strongylus vulgaris [taxid 40348]: 1 (0.028%)<br>• Sipha flava [taxid 143950]: 1 (0.028%)<br>• Sulfurospirillum halorespirans [taxid 194424]: 1 (0.028%)<br>• Methylobacterium soli [taxid 553447]: 1 (0.028%)<br>• Streptomyces griseocarneus [taxid 51201]: 1 (0.028%)
Benchmark OTU ID: `CP003481-_Proteobacteria`<br>OTU taxon: Helicobacter cetorum MIT 99-5656 [taxid 1163745]<br>Expected: Helicobacter cetorum [taxid 138563] (species)<br>Number of reads: 3242<br>Number of identified reads: 3183 (98.18%)	• species: 940 (28.994%)<br>• **genus: 1804 (55.644%)**<br>• family: 0 (0.0%)<br>• order: 28 (0.863%)<br>• class: 2 (0.061%)<br>• phylum: 35 (1.079%)<br>• superkingdom: 72 (2.22%)<br>• root: 301 (9.284%)	• **Helicobacter pylori [taxid 210]: 487 (15.021%)**<br>• Nyssa sinensis [taxid 561372]: 1 (0.03%)<br>• Candidatus Rickettsiella viridis [taxid 676208]: 1 (0.03%)<br>• Parashewanella spongiae [taxid 342950]: 1 (0.03%)<br>• Oscillibacter valericigenes [taxid 351091]: 1 (0.03%)<br>• Helicobacter felis [taxid 214]: 1 (0.03%)

Operational Taxonomic Unit (OTU)	Correct identifications	Wrong or overspecific identifications at species rank
Benchmark OTU ID: `CP002571-_Pathogens`<br>OTU taxon: Helicobacter pylori 2017 [taxid 985081]<br>Expected: Helicobacter pylori [taxid 210] (species)<br>Number of reads: 2657<br>Number of identified reads: 2636 (99.209%)	• **species: 2360 (88.821%)**<br>• genus: 19 (0.715%)<br>• family: 0 (0.0%)<br>• order: 14 (0.526%)<br>• class: 0 (0.0%)<br>• phylum: 13 (0.489%)<br>• superkingdom: 59 (2.22%)<br>• root: 170 (6.398%)	• Capsicum chinense [taxid 80379]: 1 (0.037%)<br>• Helicobacter pullorum [taxid 35818]: 1 (0.037%)
Benchmark OTU ID: `CP002184-_Pathogens`<br>OTU taxon: Helicobacter pylori 908 [taxid 869727]<br>Expected: Helicobacter pylori [taxid 210] (species)<br>Number of reads: 2660<br>Number of identified reads: 2632 (98.947%)	• **species: 2322 (87.293%)**<br>• genus: 28 (1.052%)<br>• family: 0 (0.0%)<br>• order: 16 (0.601%)<br>• class: 0 (0.0%)<br>• phylum: 34 (1.278%)<br>• superkingdom: 44 (1.654%)<br>• root: 187 (7.03%)	• Salmonella enterica [taxid 28901]: 1 (0.037%)<br>• Loktanella salsilacus [taxid 195913]: 1 (0.037%)<br>• Helicobacter heilmannii [taxid 35817]: 1 (0.037%)
Benchmark OTU ID: `CP002953-_Pathogens`<br>OTU taxon: Helicobacter pylori ELS37 [taxid 1055527]<br>Expected: Helicobacter pylori [taxid 210] (species)<br>Number of reads: 2947<br>Number of identified reads: 2922 (99.151%)	• **species: 2579 (87.512%)**<br>• genus: 23 (0.78%)<br>• family: 0 (0.0%)<br>• order: 18 (0.61%)<br>• class: 1 (0.033%)<br>• phylum: 35 (1.187%)<br>• superkingdom: 52 (1.764%)<br>• root: 213 (7.227%)	• Syphacia muris [taxid 451379]: 1 (0.033%)
Benchmark OTU ID: `AP011943-_Pathogens`<br>OTU taxon: Helicobacter pylori F32 [taxid 102608]<br>Expected: Helicobacter pylori [taxid 210] (species)<br>Number of reads: 2733<br>Number of identified reads: 2697 (98.682%)	• **species: 2349 (85.949%)**<br>• genus: 31 (1.134%)<br>• family: 2 (0.073%)<br>• order: 12 (0.439%)<br>• class: 3 (0.109%)<br>• phylum: 30 (1.097%)<br>• superkingdom: 69 (2.524%)<br>• root: 201 (7.354%)	• Wolinella succinogenes [taxid 844]: 1 (0.036%)<br>• Helicobacter pullorum [taxid 35818]: 1 (0.036%)<br>• Streptomyces griseocarneus [taxid 51201]: 1 (0.036%)<br>• Nocardia cyriacigeorgica [taxid 135487]: 1 (0.036%)
Benchmark OTU ID: `CP002331-_Pathogens`<br>OTU taxon: Helicobacter pylori India7 [taxid 907238]<br>Expected: Helicobacter pylori [taxid 210] (species)<br>Number of reads: 2976<br>Number of identified reads: 2951 (99.159%)	• **species: 2591 (87.063%)**<br>• genus: 28 (0.94%)<br>• family: 0 (0.0%)<br>• order: 18 (0.604%)<br>• class: 0 (0.0%)<br>• phylum: 30 (1.008%)<br>• superkingdom: 73 (2.452%)<br>• root: 208 (6.989%)	• Helicobacter pullorum [taxid 35818]: 1 (0.033%)<br>• Limosa lapponica [taxid 161683]: 1 (0.033%)<br>• Sipha flava [taxid 143950]: 1 (0.033%)

Operational Taxonomic Unit (OTU)	Correct identifications	Wrong or overspecific identifications at species rank
Benchmark OTU ID: `AP012601-_Pathogens`<br>OTU taxon: Helicobacter pylori OK310 [taxid 1248726]<br>Expected: Helicobacter pylori [taxid 210] (species)<br>Number of reads: 2764<br>Number of identified reads: 2745 (99.312%)	• **species: 2434 (88.06%)**<br>• genus: 31 (1.121%)<br>• family: 0 (0.0%)<br>• order: 12 (0.434%)<br>• class: 0 (0.0%)<br>• phylum: 24 (0.868%)<br>• superkingdom: 49 (1.772%)<br>• root: 194 (7.018%)	• Syphacia muris [taxid 451379]: 1 (0.036%)<br>• Helicobacter cetorum [taxid 138563]: 1 (0.036%)
Benchmark OTU ID: `CP002983-_Pathogens`<br>OTU taxon: Helicobacter pylori SNT49 [taxid 1055530]<br>Expected: Helicobacter pylori [taxid 210] (species)<br>Number of reads: 2805<br>Number of identified reads: 2776 (98.966%)	• **species: 2442 (87.058%)**<br>• genus: 28 (0.998%)<br>• family: 0 (0.0%)<br>• order: 12 (0.427%)<br>• class: 0 (0.0%)<br>• phylum: 28 (0.998%)<br>• superkingdom: 58 (2.067%)<br>• root: 206 (7.344%)	• Streptomyces prasinus [taxid 67345]: 1 (0.035%)<br>• Sipha flava [taxid 143950]: 1 (0.035%)<br>• Streptomyces griseocarneus [taxid 51201]: 1 (0.035%)
Benchmark OTU ID: `CP001072-_Pathogens`<br>OTU taxon: Helicobacter pylori Shi470 [taxid 512562]<br>Expected: Helicobacter pylori [taxid 210] (species)<br>Number of reads: 2807<br>Number of identified reads: 2788 (99.323%)	• **species: 2477 (88.243%)**<br>• genus: 25 (0.89%)<br>• family: 0 (0.0%)<br>• order: 15 (0.534%)<br>• class: 4 (0.142%)<br>• phylum: 32 (1.14%)<br>• superkingdom: 45 (1.603%)<br>• root: 189 (6.733%)	• Rhodovulum euryhalinum [taxid 35805]: 1 (0.035%)<br>• Halanaerobium saccharolyticum [taxid 43595]: 1 (0.035%)
Benchmark OTU ID: `CP003419-_Pathogens`<br>OTU taxon: Helicobacter pylori XZ274 [taxid 1127122]<br>Expected: Helicobacter pylori [taxid 210] (species)<br>Number of reads: 2871<br>Number of identified reads: 2845 (99.094%)	• **species: 2532 (88.192%)**<br>• genus: 17 (0.592%)<br>• family: 0 (0.0%)<br>• order: 12 (0.417%)<br>• class: 0 (0.0%)<br>• phylum: 23 (0.801%)<br>• superkingdom: 65 (2.264%)<br>• root: 196 (6.826%)	• Agrococcus baldri [taxid 153730]: 1 (0.034%)<br>• Helicobacter pullorum [taxid 35818]: 1 (0.034%)
Benchmark OTU ID: `CP002039-_Proteobacteria`<br>OTU taxon: Herbaspirillum seropedicae SmR1 [taxid 757424]<br>Expected: Herbaspirillum seropedicae [taxid 964] (species)<br>Number of reads: 11522<br>Number of identified reads: 11502 (99.826%)	• **species: 4995 (43.351%)**<br>• genus: 2693 (23.372%)<br>• family: 606 (5.259%)<br>• order: 663 (5.754%)<br>• class: 186 (1.614%)<br>• phylum: 862 (7.481%)<br>• superkingdom: 486 (4.218%)<br>• root: 988 (8.574%)	• Herbaspirillum rubrisubalbicans [taxid 80842]: 12 (0.104%)<br>• Lupinus albus [taxid 3870]: 9 (0.078%)<br>• Herbaspirillum robiniae [taxid 2014887]: 3 (0.026%)<br>• Herbaspirillum aquaticum [taxid 568783]: 3 (0.026%)<br>• Schizaphis graminum [taxid 13262]: 3 (0.026%)<br>• Oxalobacter formigenes [taxid 847]: 2 (0.017%)<br>• Ricinus communis [taxid 3988]: 2 (0.017%)<br>• Herbaspirillum huttiense [taxid 863372]: 2 (0.017%)<br>• other: 33 (0.286%)

Operational Taxonomic Unit (OTU)	Correct identifications	Wrong or overspecific identifications at species rank
Benchmark OTU ID: `CU207211-_Proteobacteria`<br>OTU taxon: Herminiimonas arsenicoxydans [taxid 204773]<br>Expected: Herminiimonas arsenicoxydans [taxid 204773] (species)<br>Number of reads: 6821<br>Number of identified reads: 6811 (99.853%)	• **species: 3340 (48.966%)**<br>• genus: 338 (4.955%)<br>• family: 791 (11.596%)<br>• order: 415 (6.084%)<br>• class: 167 (2.448%)<br>• phylum: 678 (9.939%)<br>• superkingdom: 251 (3.679%)<br>• root: 825 (12.095%)	• Janthinobacterium sp. Marseille [taxid 375286]: 6 (0.087%)<br>• Herminiimonas fonticola [taxid 303380]: 6 (0.087%)<br>• Lupinus albus [taxid 3870]: 3 (0.043%)<br>• Pseudomonas aeruginosa [taxid 287]: 2 (0.029%)<br>• Deinococcus hopiensis [taxid 309885]: 1 (0.014%)<br>• Massilia timonae [taxid 47229]: 1 (0.014%)<br>• Delftia acidovorans [taxid 80866]: 1 (0.014%)<br>• Ralstonia mannitolilytica [taxid 105219]: 1 (0.014%)<br>• other: 35 (0.513%)
Benchmark OTU ID: `CP000875-_Chloroflexi`<br>OTU taxon: Herpetosiphon aurantiacus DSM 785 [taxid 316274]<br>Expected: Herpetosiphon aurantiacus [taxid 65] (species)<br>Number of reads: 163897<br>Number of identified reads: 163354 (99.668%)	• **species: 104412 (63.705%)**<br>• genus: 21426 (13.072%)<br>• family: 0 (0.0%)<br>• order: 0 (0.0%)<br>• class: 181 (0.11%)<br>• phylum: 1034 (0.63%)<br>• superkingdom: 13912 (8.488%)<br>• root: 22257 (13.579%)	• Herpetosiphon geysericola [taxid 70996]: 436 (0.266%)<br>• Psychrosphaera saromensis [taxid 716813]: 5 (0.003%)<br>• Staphylococcus aureus [taxid 1280]: 5 (0.003%)<br>• Oscillochloris trichoides [taxid 104176]: 4 (0.002%)<br>• Actinomyces urogenitalis [taxid 103621]: 4 (0.002%)<br>• bacterium [taxid 1869227]: 3 (0.001%)<br>• Antarcticibacterium flavum [taxid 2058175]: 3 (0.001%)<br>• Bacteroidetes/Chlorobi group bacterium ChocPot_Mid [taxid 2382230]: 2 (0.001%)<br>• other: 228 (0.139%)
Benchmark OTU ID: `chr22-_Eukaryotes`<br>OTU taxon: Homo sapiens [taxid 9606]<br>Expected: Homo sapiens [taxid 9606] (species)<br>Number of reads: 242105<br>Number of identified reads: 219238 (90.554%)	• species: 2006 (0.828%)<br>• genus: 0 (0.0%)<br>• family: 3394 (1.401%)<br>• order: 37213 (15.37%)<br>• class: 3761 (1.553%)<br>• phylum: 1745 (0.72%)<br>• superkingdom: 5574 (2.302%)<br>• **root: 161795 (66.828%)**	• **Macaca mulatta [taxid 9544]: 4689 (1.936%)**<br>• Callithrix jacchus [taxid 9483]: 1166 (0.481%)<br>• Plasmodium ovale [taxid 36330]: 780 (0.322%)<br>• Pongo abelii [taxid 9601]: 569 (0.235%)<br>• Macaca fascicularis [taxid 9541]: 490 (0.202%)<br>• Pan troglodytes [taxid 9598]: 451 (0.186%)<br>• Nomascus leucogenys [taxid 61853]: 382 (0.157%)<br>• Gorilla gorilla [taxid 9593]: 323 (0.133%)<br>• other: 3827 (1.58%)
Benchmark OTU ID: `CP002786-_Actinobacteria`<br>OTU taxon: Hoyosella subflava DQS3-9A1 [taxid 443218]<br>Expected: Hoyosella subflava [taxid 639313] (species)<br>Number of reads: 20817<br>Number of identified reads: 20652 (99.207%)	• **species: 12460 (59.854%)**<br>• genus: 0 (0.0%)<br>• family: 228 (1.095%)<br>• order: 1324 (6.36%)<br>• class: 1537 (7.383%)<br>• phylum: 23 (0.11%)<br>• superkingdom: 1235 (5.932%)<br>• root: 3826 (18.379%)	• Mycobacteroides abscessus [taxid 36809]: 4 (0.019%)<br>• Mycobacterium tuberculosis [taxid 1773]: 4 (0.019%)<br>• Corynebacterium rouxii [taxid 2719119]: 3 (0.014%)<br>• Mycobacteroides salmoniphilum [taxid 404941]: 3 (0.014%)<br>• Nocardia stercoris [taxid 2483361]: 2 (0.009%)<br>• Varibaculum cambriense [taxid 184870]: 2 (0.009%)<br>• Nocardia amikacinitolerans [taxid 756689]: 1 (0.004%)<br>• Actinokineospora bangkokensis [taxid 1193682]: 1 (0.004%)<br>• other: 76 (0.365%)

Operational Taxonomic Unit (OTU)	Correct identifications	Wrong or overspecific identifications at species rank
Benchmark OTU ID: `CP000493-_Crenarchaeota`<br>OTU taxon: Hyperthermus butylicus DSM 5456 [taxid 415426]<br>Expected: Hyperthermus butylicus [taxid 54248] (species)<br>Number of reads: 3293<br>Number of identified reads: 3197 (97.084%)	• **species: 1792 (54.418%)**<br>• genus: 1 (0.03%)<br>• family: 53 (1.609%)<br>• order: 8 (0.242%)<br>• class: 33 (1.002%)<br>• phylum: 0 (0.0%)<br>• superkingdom: 34 (1.032%)<br>• root: 1203 (36.532%)	• Thermoprotei archaeon [taxid 2250277]: 2 (0.06%)<br>• Pyrolobus fumarii [taxid 54252]: 2 (0.06%)<br>• Pseudomonas stutzeri [taxid 316]: 1 (0.03%)<br>• Nonomuraea solani [taxid 1144553]: 1 (0.03%)<br>• Ereboglobus luteus [taxid 1796921]: 1 (0.03%)<br>• Gordonia terrae [taxid 2055]: 1 (0.03%)<br>• Pyrodictium delaneyi [taxid 1273541]: 1 (0.03%)<br>• Lysobacter aestuarii [taxid 1706195]: 1 (0.03%)<br>• other: 6 (0.182%)
Benchmark OTU ID: `CP005587-_Proteobacteria`<br>OTU taxon: Hyphomicrobium denitrificans 1NES1 [taxid 670307]<br>Expected: Hyphomicrobium denitrificans [taxid 53399] (species)<br>Number of reads: 7685<br>Number of identified reads: 7643 (99.453%)	• **species: 4088 (53.194%)**<br>• genus: 949 (12.348%)<br>• family: 19 (0.247%)<br>• order: 277 (3.604%)<br>• class: 362 (4.71%)<br>• phylum: 265 (3.448%)<br>• superkingdom: 361 (4.697%)<br>• root: 1310 (17.046%)	• Mycobacterium innocens [taxid 2341083]: 1 (0.013%)<br>• Anopheles darlingi [taxid 43151]: 1 (0.013%)<br>• Fopius arisanus [taxid 64838]: 1 (0.013%)<br>• Aplosporella prunicola [taxid 462254]: 1 (0.013%)<br>• Paludibacterium purpuratum [taxid 1144873]: 1 (0.013%)<br>• Hyphomicrobium nitrativorans [taxid 1427356]: 1 (0.013%)<br>• Chlamydomonas leiostraca [taxid 1034604]: 1 (0.013%)<br>• Fibroporia radiculosa [taxid 599839]: 1 (0.013%)<br>• other: 16 (0.208%)
Benchmark OTU ID: `CP002083-_Proteobacteria`<br>OTU taxon: Hyphomicrobium denitrificans ATCC 51888 [taxid 582899]<br>Expected: Hyphomicrobium denitrificans [taxid 53399] (species)<br>Number of reads: 7304<br>Number of identified reads: 7271 (99.548%)	• species: 2108 (28.86%)<br>• **genus: 3036 (41.566%)**<br>• family: 14 (0.191%)<br>• order: 227 (3.107%)<br>• class: 278 (3.806%)<br>• phylum: 201 (2.751%)<br>• superkingdom: 317 (4.34%)<br>• root: 1084 (14.841%)	• Hyphomicrobium sp. xq [taxid 2665159]: 1 (0.013%)<br>• Aplanochytrium stocchinoi [taxid 215587]: 1 (0.013%)<br>• Tychonema bourrellyi [taxid 54313]: 1 (0.013%)<br>• Hyphomicrobium sulfonivorans [taxid 121290]: 1 (0.013%)<br>• Brevundimonas abyssalis [taxid 1125965]: 1 (0.013%)<br>• Hyphomicrobium facile [taxid 51670]: 1 (0.013%)<br>• Methyloceanibacter methanicus [taxid 1774968]: 1 (0.013%)<br>• Bartonella quintana [taxid 803]: 1 (0.013%)<br>• other: 5 (0.068%)
Benchmark OTU ID: `FQ859181-_Proteobacteria`<br>OTU taxon: Hyphomicrobium sp. MC1 [taxid 717785]<br>Expected: Hyphomicrobium [taxid 81] (genus)<br>Number of reads: 9820<br>Number of identified reads: 9788 (99.674%)	• **genus: 6821 (69.46%)**<br>• family: 16 (0.162%)<br>• order: 331 (3.37%)<br>• class: 351 (3.574%)<br>• phylum: 372 (3.788%)<br>• superkingdom: 422 (4.297%)<br>• root: 1462 (14.887%)	• Hyphomicrobium sp. xq [taxid 2665159]: 20 (0.203%)<br>• Hyphomicrobium denitrificans [taxid 53399]: 12 (0.122%)<br>• Hyphomicrobium sulfonivorans [taxid 121290]: 2 (0.02%)<br>• Pelotomaculum propionicicum [taxid 258475]: 1 (0.01%)<br>• Actinoalloteichus hoggarensis [taxid 1470176]: 1 (0.01%)<br>• Aliidiomarina sanyensis [taxid 1249555]: 1 (0.01%)<br>• Phreatobacter stygius [taxid 1940610]: 1 (0.01%)<br>• other: 26 (0.264%)

Operational Taxonomic Unit (OTU)	Correct identifications	Wrong or overspecific identifications at species rank
Benchmark OTU ID: `CP000158-_Proteobacteria`<br>OTU taxon: Hyphomonas neptunium ATCC 15444 [taxid 228405]<br>Expected: Hyphomonas neptunium [taxid 81032] (species)<br>Number of reads: 7452<br>Number of identified reads: 7434 (99.758%)	• species: 85 (1.14%)<br>• **genus: 4398 (59.017%)**<br>• family: 258 (3.462%)<br>• order: 0 (0.0%)<br>• class: 1349 (18.102%)<br>• phylum: 197 (2.643%)<br>• superkingdom: 311 (4.173%)<br>• root: 829 (11.124%)	• Hyphomonas hirschiana [taxid 81030]: 91 (1.221%)<br>• Hyphomonas beringensis [taxid 1280946]: 3 (0.04%)<br>• Hyphomonas oceanitis [taxid 81033]: 2 (0.026%)<br>• Hyphomonas adhaerens [taxid 81029]: 2 (0.026%)<br>• Phaffia rhodozyma [taxid 264483]: 1 (0.013%)<br>• Hyphomonas jannaschiana [taxid 86]: 1 (0.013%)<br>• Lupinus albus [taxid 3870]: 1 (0.013%)<br>• Botryotinia narcissicola [taxid 278944]: 1 (0.013%)<br>• other: 23 (0.308%)
Benchmark OTU ID: `CP000816-_Crenarchaeota`<br>OTU taxon: Ignicoccus hospitalis KIN4/I [taxid 453591]<br>Expected: Ignicoccus hospitalis [taxid 160233] (species)<br>Number of reads: 2369<br>Number of identified reads: 2318 (97.847%)	• **species: 1403 (59.223%)**<br>• genus: 21 (0.886%)<br>• family: 1 (0.042%)<br>• order: 2 (0.084%)<br>• class: 10 (0.422%)<br>• phylum: 0 (0.0%)<br>• superkingdom: 21 (0.886%)<br>• root: 809 (34.149%)	• Ignicoccus islandicus [taxid 54259]: 2 (0.084%)<br>• Pseudomassariella vexata [taxid 1141098]: 1 (0.042%)<br>• Chlamydomonas reinhardtii [taxid 3055]: 1 (0.042%)
Benchmark OTU ID: `CP002098-_Crenarchaeota`<br>OTU taxon: Ignisphaera aggregans DSM 17230 [taxid 583356]<br>Expected: Ignisphaera aggregans [taxid 334771] (species)<br>Number of reads: 3815<br>Number of identified reads: 3736 (97.929%)	• **species: 2660 (69.724%)**<br>• genus: 0 (0.0%)<br>• family: 8 (0.209%)<br>• order: 1 (0.026%)<br>• class: 30 (0.786%)<br>• phylum: 0 (0.0%)<br>• superkingdom: 29 (0.76%)<br>• root: 965 (25.294%)	• Thermoprotei archaeon [taxid 2250277]: 2 (0.052%)<br>• Davidia involucrata [taxid 16924]: 1 (0.026%)<br>• Zestosphaera tikiterensis [taxid 1973259]: 1 (0.026%)<br>• Candidatus Bathyarchaeota archaeon [taxid 2026714]: 1 (0.026%)<br>• Chryseobacterium angstadtii [taxid 558151]: 1 (0.026%)<br>• Valsa malicola [taxid 356882]: 1 (0.026%)
Benchmark OTU ID: `AP012057-_Actinobacteria`<br>OTU taxon: Ilumatobacter coccineus YM16-304 [taxid 1313172]<br>Expected: Ilumatobacter coccineus [taxid 467094] (species)<br>Number of reads: 21264<br>Number of identified reads: 21211 (99.75%)	• **species: 15481 (72.803%)**<br>• genus: 102 (0.479%)<br>• family: 0 (0.0%)<br>• order: 149 (0.7%)<br>• class: 7 (0.032%)<br>• phylum: 1288 (6.057%)<br>• superkingdom: 1792 (8.427%)<br>• root: 2357 (11.084%)	• Ilumatobacter fluminis [taxid 467091]: 1 (0.004%)<br>• Rosenbergiella nectarea [taxid 988801]: 1 (0.004%)<br>• Clostridium acetireducens [taxid 76489]: 1 (0.004%)<br>• Pseudonocardia autotrophica [taxid 2074]: 1 (0.004%)<br>• Methylobacterium currus [taxid 2051553]: 1 (0.004%)<br>• Salmonella enterica [taxid 28901]: 1 (0.004%)<br>• Mycobacteroides abscessus [taxid 36809]: 1 (0.004%)<br>• Aspergillus sclerotiicarbonarius [taxid 487660]: 1 (0.004%)<br>• other: 34 (0.159%)

Operational Taxonomic Unit (OTU)	Correct identifications	Wrong or overspecific identifications at species rank
Benchmark OTU ID: `CP002353-_Planctomycetes`<br>OTU taxon: Isosphaera pallida ATCC 43644 [taxid 575540]<br>Expected: Isosphaera pallida [taxid 128] (species)<br>Number of reads: 198873<br>Number of identified reads: 196110 (98.61%)	• **species: 139550 (70.17%)**<br>• genus: 0 (0.0%)<br>• family: 598 (0.3%)<br>• order: 0 (0.0%)<br>• class: 1071 (0.538%)<br>• phylum: 1475 (0.741%)<br>• superkingdom: 16892 (8.493%)<br>• root: 36251 (18.228%)	• Beta vulgaris [taxid 161934]: 13 (0.006%)<br>• Aquisphaera giovannonii [taxid 406548]: 12 (0.006%)<br>• Tautonia sociabilis [taxid 2080755]: 8 (0.004%)<br>• Tautonia plasticadhaerens [taxid 2527974]: 7 (0.003%)<br>• Paludisphaera borealis [taxid 1387353]: 5 (0.002%)<br>• Escherichia coli [taxid 562]: 4 (0.002%)<br>• Gemmata massiliana [taxid 1210884]: 4 (0.002%)<br>• Hordeum vulgare [taxid 4513]: 3 (0.001%)<br>• other: 339 (0.17%)
Benchmark OTU ID: `CP000269-_Proteobacteria`<br>OTU taxon: Janthinobacterium sp. Marseille [taxid 375286]<br>Expected: Janthinobacterium sp. Marseille [taxid 375286] (species)<br>Number of reads: 8364<br>Number of identified reads: 8354 (99.88%)	• **species: 3883 (46.425%)**<br>• genus: 9 (0.107%)<br>• family: 1922 (22.979%)<br>• order: 500 (5.978%)<br>• class: 178 (2.128%)<br>• phylum: 694 (8.297%)<br>• superkingdom: 271 (3.24%)<br>• root: 892 (10.664%)	• Herminiimonas arsenicoxydans [taxid 204773]: 6 (0.071%)<br>• Lupinus albus [taxid 3870]: 3 (0.035%)<br>• Herminiimonas fonticola [taxid 303380]: 2 (0.023%)<br>• Collimonas arenae [taxid 279058]: 2 (0.023%)<br>• Salmonella enterica [taxid 28901]: 2 (0.023%)<br>• Geobacter argillaceus [taxid 345631]: 1 (0.011%)<br>• Ancylobacter pratisalsi [taxid 1745854]: 1 (0.011%)<br>• Bacillus gobiensis [taxid 1441095]: 1 (0.011%)<br>• other: 33 (0.394%)
Benchmark OTU ID: `CP002018-_Proteobacteria`<br>OTU taxon: Ketogulonicigenium vulgare WSH-001 [taxid 759362]<br>Expected: Ketogulonicigenium vulgare [taxid 92945] (species)<br>Number of reads: 5340<br>Number of identified reads: 5326 (99.737%)	• **species: 3191 (59.756%)**<br>• genus: 228 (4.269%)<br>• family: 534 (10.0%)<br>• order: 15 (0.28%)<br>• class: 351 (6.573%)<br>• phylum: 170 (3.183%)<br>• superkingdom: 292 (5.468%)<br>• root: 533 (9.981%)	• Ketogulonicigenium robustum [taxid 92947]: 43 (0.805%)<br>• Pontibaca methylaminivorans [taxid 515897]: 2 (0.037%)<br>• Rhodovulum imhoffii [taxid 365340]: 2 (0.037%)<br>• Roseisalinus antarcticus [taxid 254357]: 2 (0.037%)<br>• Rhodobaca barguzinensis [taxid 441209]: 2 (0.037%)<br>• Pseudoruegeria haliotis [taxid 1280846]: 2 (0.037%)<br>• Rhodobacter aestuarii [taxid 453582]: 1 (0.018%)<br>• Hevea brasiliensis [taxid 3981]: 1 (0.018%)<br>• other: 38 (0.711%)
Benchmark OTU ID: `CP002224-_Proteobacteria`<br>OTU taxon: Ketogulonicigenium vulgare Y25 [taxid 880591]<br>Expected: Ketogulonicigenium vulgare [taxid 92945] (species)<br>Number of reads: 5362<br>Number of identified reads: 5350 (99.776%)	• **species: 3206 (59.791%)**<br>• genus: 251 (4.681%)<br>• family: 489 (9.119%)<br>• order: 14 (0.261%)<br>• class: 341 (6.359%)<br>• phylum: 192 (3.58%)<br>• superkingdom: 264 (4.923%)<br>• root: 586 (10.928%)	• Ketogulonicigenium robustum [taxid 92947]: 42 (0.783%)<br>• Rhodovulum sulfidophilum [taxid 35806]: 2 (0.037%)<br>• Actibacterium mucosum [taxid 1087332]: 2 (0.037%)<br>• Sulfitobacter guttiformis [taxid 74349]: 2 (0.037%)<br>• Palleronia marisminoris [taxid 315423]: 1 (0.018%)<br>• Salinihabitans flavidus [taxid 569882]: 1 (0.018%)<br>• Paracoccus alcaliphilus [taxid 34002]: 1 (0.018%)<br>• Methylocystis heyeri [taxid 391905]: 1 (0.018%)<br>• other: 25 (0.466%)

Operational Taxonomic Unit (OTU)	Correct identifications	Wrong or overspecific identifications at species rank
Benchmark OTU ID: `AP010968-_Actinobacteria`<br>OTU taxon: Kitasatospora setae KM-6054 [taxid 452652]<br>Expected: Kitasatospora setae [taxid 2066] (species)<br>Number of reads: 40635<br>Number of identified reads: 40459 (99.566%)	• **species: 18556 (45.665%)**<br>• genus: 1592 (3.917%)<br>• family: 11217 (27.604%)<br>• order: 0 (0.0%)<br>• class: 2786 (6.856%)<br>• phylum: 37 (0.091%)<br>• superkingdom: 2683 (6.602%)<br>• root: 3515 (8.65%)	• Kitasatospora cheerisanensis [taxid 81942]: 15 (0.036%)<br>• Streptacidiphilus bronchialis [taxid 2126346]: 6 (0.014%)<br>• Kitasatospora cineracea [taxid 88074]: 6 (0.014%)<br>• Kitasatospora atroaurantiaca [taxid 285545]: 5 (0.012%)<br>• Globodera pallida [taxid 36090]: 4 (0.009%)<br>• Streptomyces sp. C [taxid 253839]: 4 (0.009%)<br>• Microcella alkaliphila [taxid 279828]: 4 (0.009%)<br>• Streptomyces albidoflavus [taxid 1886]: 4 (0.009%)<br>• other: 180 (0.442%)
Benchmark OTU ID: `CP002824-_Proteobacteria`<br>OTU taxon: Klebsiella aerogenes KCTC 2190 [taxid 1028307]<br>Expected: Klebsiella aerogenes [taxid 548] (species)<br>Number of reads: 10995<br>Number of identified reads: 10961 (99.69%)	• species: 3349 (30.459%)<br>• genus: 797 (7.248%)<br>• **family: 4139 (37.644%)**<br>• order: 911 (8.285%)<br>• class: 306 (2.783%)<br>• phylum: 223 (2.028%)<br>• superkingdom: 327 (2.974%)<br>• root: 902 (8.203%)	• Salmonella enterica [taxid 28901]: 147 (1.336%)<br>• Klebsiella pneumoniae [taxid 573]: 85 (0.773%)<br>• Escherichia coli [taxid 562]: 79 (0.718%)<br>• Klebsiella variicola [taxid 244366]: 8 (0.072%)<br>• Klebsiella oxytoca [taxid 571]: 7 (0.063%)<br>• Klebsiella michiganensis [taxid 1134687]: 6 (0.054%)<br>• Klebsiella grimontii [taxid 2058152]: 5 (0.045%)<br>• Enterobacter ludwigii [taxid 299767]: 5 (0.045%)<br>• other: 141 (1.282%)
Benchmark OTU ID: `CP003683-_Proteobacteria`<br>OTU taxon: Klebsiella michiganensis E718 [taxid 1191061]<br>Expected: Klebsiella michiganensis [taxid 1134687] (species)<br>Number of reads: 12834<br>Number of identified reads: 12790 (99.657%)	• species: 223 (1.737%)<br>• **genus: 4778 (37.229%)**<br>• family: 4730 (36.855%)<br>• order: 903 (7.035%)<br>• class: 422 (3.288%)<br>• phylum: 247 (1.924%)<br>• superkingdom: 381 (2.968%)<br>• root: 1096 (8.539%)	• Salmonella enterica [taxid 28901]: 143 (1.114%)<br>• Klebsiella pneumoniae [taxid 573]: 110 (0.857%)<br>• Escherichia coli [taxid 562]: 96 (0.748%)<br>• Klebsiella oxytoca [taxid 571]: 66 (0.514%)<br>• Klebsiella grimontii [taxid 2058152]: 37 (0.288%)<br>• Klebsiella indica [taxid 2582917]: 18 (0.14%)<br>• Klebsiella aerogenes [taxid 548]: 18 (0.14%)<br>• Klebsiella variicola [taxid 244366]: 15 (0.116%)<br>• other: 175 (1.363%)
Benchmark OTU ID: `CP003218-_Proteobacteria`<br>OTU taxon: Klebsiella michiganensis KCTC 1686 [taxid 1006551]<br>Expected: Klebsiella michiganensis [taxid 1134687] (species)<br>Number of reads: 12558<br>Number of identified reads: 12506 (99.585%)	• species: 433 (3.448%)<br>• genus: 4491 (35.762%)<br>• **family: 4621 (36.797%)**<br>• order: 998 (7.947%)<br>• class: 325 (2.587%)<br>• phylum: 220 (1.751%)<br>• superkingdom: 382 (3.041%)<br>• root: 1023 (8.146%)	• Salmonella enterica [taxid 28901]: 166 (1.321%)<br>• Escherichia coli [taxid 562]: 70 (0.557%)<br>• Klebsiella pneumoniae [taxid 573]: 65 (0.517%)<br>• Klebsiella oxytoca [taxid 571]: 42 (0.334%)<br>• Klebsiella grimontii [taxid 2058152]: 18 (0.143%)<br>• Klebsiella quasipneumoniae [taxid 1463165]: 14 (0.111%)<br>• Klebsiella indica [taxid 2582917]: 13 (0.103%)<br>• Raoultella terrigena [taxid 577]: 11 (0.087%)<br>• other: 157 (1.25%)

Operational Taxonomic Unit (OTU)	Correct identifications	Wrong or overspecific identifications at species rank
Benchmark OTU ID: `CP003200-_Proteobacteria`<br>OTU taxon: Klebsiella pneumoniae subsp. pneumoniae HS11286 [taxid 1125630]<br>Expected: Klebsiella pneumoniae [taxid 573] (species)<br>Number of reads: 11117<br>Number of identified reads: 11085 (99.712%)	• species: 1433 (12.89%)<br>• genus: 2547 (22.91%)<br>• **family: 4850 (43.626%)**<br>• order: 716 (6.44%)<br>• class: 309 (2.779%)<br>• phylum: 198 (1.781%)<br>• superkingdom: 283 (2.545%)<br>• root: 737 (6.629%)	• Salmonella enterica [taxid 28901]: 101 (0.908%)<br>• Escherichia coli [taxid 562]: 80 (0.719%)<br>• Klebsiella quasipneumoniae [taxid 1463165]: 38 (0.341%)<br>• Klebsiella quasivariicola [taxid 2026240]: 12 (0.107%)<br>• Klebsiella aerogenes [taxid 548]: 11 (0.098%)<br>• Klebsiella variicola [taxid 244366]: 10 (0.089%)<br>• Klebsiella oxytoca [taxid 571]: 9 (0.08%)<br>• Klebsiella grimontii [taxid 2058152]: 8 (0.071%)<br>• other: 122 (1.097%)
Benchmark OTU ID: `AP006725-_Proteobacteria`<br>OTU taxon: Klebsiella pneumoniae subsp. pneumoniae NTUH-K2044 [taxid 484021]<br>Expected: Klebsiella pneumoniae [taxid 573] (species)<br>Number of reads: 10925<br>Number of identified reads: 10887 (99.652%)	• species: 1270 (11.624%)<br>• genus: 2954 (27.038%)<br>• **family: 4521 (41.382%)**<br>• order: 743 (6.8%)<br>• class: 269 (2.462%)<br>• phylum: 204 (1.867%)<br>• superkingdom: 298 (2.727%)<br>• root: 625 (5.72%)	• Salmonella enterica [taxid 28901]: 107 (0.979%)<br>• Escherichia coli [taxid 562]: 79 (0.723%)<br>• Klebsiella quasipneumoniae [taxid 1463165]: 36 (0.329%)<br>• Klebsiella variicola [taxid 244366]: 19 (0.173%)<br>• Klebsiella aerogenes [taxid 548]: 17 (0.155%)<br>• Klebsiella quasivariicola [taxid 2026240]: 8 (0.073%)<br>• Enterobacter cloacae [taxid 550]: 6 (0.054%)<br>• Pluralibacter gergoviae [taxid 61647]: 5 (0.045%)<br>• other: 123 (1.125%)
Benchmark OTU ID: `AP012159-_Proteobacteria`<br>OTU taxon: Komagataeibacter medellinensis NBRC 3288 [taxid 634177]<br>Expected: Komagataeibacter medellinensis [taxid 1177712] (species)<br>Number of reads: 6174<br>Number of identified reads: 6147 (99.562%)	• **species: 2549 (41.286%)**<br>• genus: 631 (10.22%)<br>• family: 1447 (23.436%)<br>• order: 26 (0.421%)<br>• class: 190 (3.077%)<br>• phylum: 158 (2.559%)<br>• superkingdom: 246 (3.984%)<br>• root: 897 (14.528%)	• Komagataeibacter nataicola [taxid 265960]: 6 (0.097%)<br>• Komagataeibacter xylinus [taxid 28448]: 6 (0.097%)<br>• Acetobacter pasteurianus [taxid 438]: 4 (0.064%)<br>• Komagataeibacter intermedius [taxid 66229]: 4 (0.064%)<br>• Gluconobacter frateurii [taxid 38308]: 3 (0.048%)<br>• Komagataeibacter melaceti [taxid 2766577]: 3 (0.048%)<br>• Acetobacter aceti [taxid 435]: 3 (0.048%)<br>• Granulibacter bethesdensis [taxid 364410]: 2 (0.032%)<br>• other: 42 (0.68%)
Benchmark OTU ID: `CP002017-_Firmicutes`<br>OTU taxon: Kyrpidia tusciae DSM 2912 [taxid 562970]<br>Expected: Kyrpidia tusciae [taxid 33943] (species)<br>Number of reads: 4647<br>Number of identified reads: 4603 (99.053%)	• **species: 1718 (36.97%)**<br>• genus: 1415 (30.449%)<br>• family: 15 (0.322%)<br>• order: 145 (3.12%)<br>• class: 14 (0.301%)<br>• phylum: 80 (1.721%)<br>• superkingdom: 434 (9.339%)<br>• root: 781 (16.806%)	• Kyrpidia spormannii [taxid 2055160]: 87 (1.872%)<br>• Candidatus Bipolaricaulis anaerobius [taxid 2026885]: 1 (0.021%)<br>• Azospirillum brasilense [taxid 192]: 1 (0.021%)<br>• Edaphobacillus lindanitolerans [taxid 550447]: 1 (0.021%)<br>• Brevibacillus panacihumi [taxid 497735]: 1 (0.021%)<br>• Phocaeicola vulgatus [taxid 821]: 1 (0.021%)<br>• Macrostomum lignano [taxid 282301]: 1 (0.021%)<br>• Sulfobacillus benefaciens [taxid 453960]: 1 (0.021%)<br>• other: 6 (0.129%)

Operational Taxonomic Unit (OTU)	Correct identifications	Wrong or overspecific identifications at species rank
Benchmark OTU ID: `CP001686-_Actinobacteria`<br>OTU taxon: Kytococcus sedentarius DSM 20547 [taxid 478801]<br>Expected: Kytococcus sedentarius [taxid 1276] (species)<br>Number of reads: 11244<br>Number of identified reads: 11187 (99.493%)	• **species: 6723 (59.791%)**<br>• genus: 945 (8.404%)<br>• family: 0 (0.0%)<br>• order: 567 (5.042%)<br>• class: 1199 (10.663%)<br>• phylum: 10 (0.088%)<br>• superkingdom: 815 (7.248%)<br>• root: 916 (8.146%)	• Kytococcus aerolatus [taxid 592308]: 15 (0.133%)<br>• Brevibacterium aurantiacum [taxid 273384]: 13 (0.115%)<br>• Propionibacterium cyclohexanicum [taxid 64702]: 9 (0.08%)<br>• Dermacoccus nishinomiyaensis [taxid 1274]: 3 (0.026%)<br>• Salmonella enterica [taxid 28901]: 2 (0.017%)<br>• Kocuria soli [taxid 2485125]: 2 (0.017%)<br>• Mycobacteroides abscessus [taxid 36809]: 2 (0.017%)<br>• Microbacterium lacticum [taxid 33885]: 1 (0.008%)<br>• other: 49 (0.435%)
Benchmark OTU ID: `CP000885-_Firmicutes`<br>OTU taxon: Lachnoclostridium phytofermentans ISDg [taxid 357809]<br>Expected: Lachnoclostridium phytofermentans [taxid 66219] (species)<br>Number of reads: 6987<br>Number of identified reads: 6863 (98.225%)	• **species: 5126 (73.364%)**<br>• genus: 9 (0.128%)<br>• family: 148 (2.118%)<br>• order: 308 (4.408%)<br>• class: 4 (0.057%)<br>• phylum: 179 (2.561%)<br>• superkingdom: 272 (3.892%)<br>• root: 815 (11.664%)	• [Ruminococcus] torques [taxid 33039]: 1 (0.014%)<br>• Herbinix luporum [taxid 1679721]: 1 (0.014%)<br>• Saccharothrix syringae [taxid 103733]: 1 (0.014%)<br>• Eubacterium ventriosum [taxid 39496]: 1 (0.014%)<br>• Anaerocolumna xylanovorans [taxid 100134]: 1 (0.014%)<br>• Glutamicibacter nicotianae [taxid 37929]: 1 (0.014%)<br>• Colletotrichum shisoi [taxid 2078593]: 1 (0.014%)<br>• Aliicoccus persicus [taxid 930138]: 1 (0.014%)<br>• other: 13 (0.186%)
Benchmark OTU ID: `HE970764-_Firmicutes`<br>OTU taxon: Lacticaseibacillus paracasei [taxid 1597]<br>Expected: Lacticaseibacillus paracasei [taxid 1597] (species)<br>Number of reads: 4152<br>Number of identified reads: 4139 (99.686%)	• species: 153 (3.684%)<br>• **genus: 2125 (51.18%)**<br>• family: 1047 (25.216%)<br>• order: 83 (1.999%)<br>• class: 30 (0.722%)<br>• phylum: 28 (0.674%)<br>• superkingdom: 120 (2.89%)<br>• root: 550 (13.246%)	• Lacticaseibacillus casei [taxid 1582]: 29 (0.698%)<br>• Lacticaseibacillus rhamnosus [taxid 47715]: 8 (0.192%)<br>• Lacticaseibacillus chiayiensis [taxid 2100821]: 5 (0.12%)<br>• Weissella thailandensis [taxid 89061]: 3 (0.072%)<br>• Lacticaseibacillus saniviri [taxid 931533]: 2 (0.048%)<br>• Lacticaseibacillus sharpeae [taxid 1626]: 2 (0.048%)<br>• Weissella hellenica [taxid 46256]: 1 (0.024%)<br>• Weissella cibaria [taxid 137591]: 1 (0.024%)<br>• other: 16 (0.385%)
Benchmark OTU ID: `AP009333-_Firmicutes`<br>OTU taxon: Lactococcus garvieae Lg2 [taxid 420890]<br>Expected: Lactococcus garvieae [taxid 1363] (species)<br>Number of reads: 2373<br>Number of identified reads: 2355 (99.241%)	• species: 811 (34.176%)<br>• **genus: 982 (41.382%)**<br>• family: 97 (4.087%)<br>• order: 67 (2.823%)<br>• class: 22 (0.927%)<br>• phylum: 20 (0.842%)<br>• superkingdom: 99 (4.171%)<br>• root: 252 (10.619%)	• Lactococcus lactis [taxid 1358]: 8 (0.337%)<br>• Listeria fleischmannii [taxid 1069827]: 2 (0.084%)<br>• Lactococcus piscium [taxid 1364]: 2 (0.084%)<br>• Lactococcus termiticola [taxid 2169526]: 2 (0.084%)<br>• Trichuris trichiura [taxid 36087]: 2 (0.084%)<br>• Lactococcus petauri [taxid 1940789]: 1 (0.042%)<br>• Lactococcus reticulitermitis [taxid 2025039]: 1 (0.042%)<br>• Syntrophaceticus schinkii [taxid 499207]: 1 (0.042%)<br>• other: 3 (0.126%)

Operational Taxonomic Unit (OTU)	Correct identifications	Wrong or overspecific identifications at species rank
Benchmark OTU ID: `AM406671-_Firmicutes`<br>OTU taxon: Lactococcus lactis subsp. cremoris MG1363 [taxid 416870]<br>Expected: Lactococcus lactis [taxid 1358] (species)<br>Number of reads: 3278<br>Number of identified reads: 3224 (98.352%)	• **species: 2345 (71.537%)**<br>• genus: 178 (5.43%)<br>• family: 110 (3.355%)<br>• order: 75 (2.287%)<br>• class: 39 (1.189%)<br>• phylum: 37 (1.128%)<br>• superkingdom: 123 (3.752%)<br>• root: 312 (9.517%)	• Streptococcus mitis [taxid 28037]: 2 (0.061%)<br>• Streptococcus anginosus [taxid 1328]: 2 (0.061%)<br>• Streptococcus sobrinus [taxid 1310]: 2 (0.061%)<br>• Anaerosphaera multitolerans [taxid 2487351]: 1 (0.03%)<br>• Streptococcus thermophilus [taxid 1308]: 1 (0.03%)<br>• Lactococcus garvieae [taxid 1363]: 1 (0.03%)<br>• Enterococcus saigonensis [taxid 1805431]: 1 (0.03%)<br>• Thermosinus carboxydivorans [taxid 261685]: 1 (0.03%)<br>• other: 10 (0.305%)
Benchmark OTU ID: `CP002365-_Firmicutes`<br>OTU taxon: Lactococcus lactis subsp. lactis CV56 [taxid 929102]<br>Expected: Lactococcus lactis [taxid 1358] (species)<br>Number of reads: 3070<br>Number of identified reads: 3035 (98.859%)	• **species: 2252 (73.355%)**<br>• genus: 155 (5.048%)<br>• family: 58 (1.889%)<br>• order: 70 (2.28%)<br>• class: 38 (1.237%)<br>• phylum: 20 (0.651%)<br>• superkingdom: 109 (3.55%)<br>• root: 332 (10.814%)	• Lactococcus allomyrinae [taxid 2419773]: 1 (0.032%)<br>• Staphylococcus aureus [taxid 1280]: 1 (0.032%)<br>• Aphanomyces euteiches [taxid 100861]: 1 (0.032%)<br>• Lactococcus fujiensis [taxid 610251]: 1 (0.032%)<br>• Ligilactobacillus salivarius [taxid 1624]: 1 (0.032%)<br>• Listeria monocytogenes [taxid 1639]: 1 (0.032%)<br>• Amedibacillus dolichus [taxid 31971]: 1 (0.032%)<br>• Rickettsiales endosymbiont of Stachyamoeba lipophora [taxid 2486578]: 1 (0.032%)<br>• other: 2 (0.065%)
Benchmark OTU ID: `AP012281-_Firmicutes`<br>OTU taxon: Lactococcus lactis subsp. lactis IO-1 [taxid 1046624]<br>Expected: Lactococcus lactis [taxid 1358] (species)<br>Number of reads: 3105<br>Number of identified reads: 3064 (98.679%)	• **species: 2326 (74.911%)**<br>• genus: 190 (6.119%)<br>• family: 50 (1.61%)<br>• order: 61 (1.964%)<br>• class: 30 (0.966%)<br>• phylum: 21 (0.676%)<br>• superkingdom: 122 (3.929%)<br>• root: 260 (8.373%)	• Lactococcus garvieae [taxid 1363]: 2 (0.064%)<br>• Streptococcus equi [taxid 1336]: 2 (0.064%)<br>• Lupinus albus [taxid 3870]: 1 (0.032%)<br>• Salinicoccus kekensis [taxid 714307]: 1 (0.032%)<br>• Lactobacillus pontis [taxid 35787]: 1 (0.032%)<br>• Azospirillum brasilense [taxid 192]: 1 (0.032%)<br>• Neobacillus bataviensis [taxid 220685]: 1 (0.032%)<br>• Weissella cryptocerci [taxid 2506420]: 1 (0.032%)<br>• other: 4 (0.128%)
Benchmark OTU ID: `AE005176-_Firmicutes`<br>OTU taxon: Lactococcus lactis subsp. lactis Il1403 [taxid 272623]<br>Expected: Lactococcus lactis [taxid 1358] (species)<br>Number of reads: 3016<br>Number of identified reads: 2974 (98.607%)	• **species: 2102 (69.694%)**<br>• genus: 208 (6.896%)<br>• family: 85 (2.818%)<br>• order: 69 (2.287%)<br>• class: 28 (0.928%)<br>• phylum: 23 (0.762%)<br>• superkingdom: 105 (3.481%)<br>• root: 344 (11.405%)	• [Clostridium] innocuum [taxid 1522]: 1 (0.033%)<br>• Streptococcus pneumoniae [taxid 1313]: 1 (0.033%)<br>• Granulicatella balaenopterae [taxid 137733]: 1 (0.033%)<br>• Weissella soli [taxid 155866]: 1 (0.033%)<br>• Streptococcus sanguinis [taxid 1305]: 1 (0.033%)<br>• Lactococcus fujiensis [taxid 610251]: 1 (0.033%)<br>• Streptococcus mitis [taxid 28037]: 1 (0.033%)<br>• Streptococcus dysgalactiae [taxid 1334]: 1 (0.033%)

Operational Taxonomic Unit (OTU)	Correct identifications	Wrong or overspecific identifications at species rank
Benchmark OTU ID: `CP001834-_Firmicutes`<br>OTU taxon: Lactococcus lactis subsp. lactis KF147 [taxid 684738]<br>Expected: Lactococcus lactis [taxid 1358] (species)<br>Number of reads: 3388<br>Number of identified reads: 3341 (98.612%)	• **species: 2492 (73.553%)**<br>• genus: 206 (6.08%)<br>• family: 71 (2.095%)<br>• order: 76 (2.243%)<br>• class: 37 (1.092%)<br>• phylum: 26 (0.767%)<br>• superkingdom: 105 (3.099%)<br>• root: 325 (9.592%)	• Lactococcus raffinolactis [taxid 1366]: 4 (0.118%)<br>• Lactococcus fujiensis [taxid 610251]: 2 (0.059%)<br>• Pseudoalteromonas luteoviolacea [taxid 43657]: 1 (0.029%)<br>• Streptococcus equi [taxid 1336]: 1 (0.029%)<br>• Apilactobacillus kunkeei [taxid 148814]: 1 (0.029%)<br>• Ligilactobacillus animalis [taxid 1605]: 1 (0.029%)
Benchmark OTU ID: `CP001154-_Proteobacteria`<br>OTU taxon: Laribacter hongkongensis HLHK9 [taxid 557598]<br>Expected: Laribacter hongkongensis [taxid 168471] (species)<br>Number of reads: 6247<br>Number of identified reads: 6231 (99.743%)	• **species: 4031 (64.526%)**<br>• genus: 0 (0.0%)<br>• family: 96 (1.536%)<br>• order: 99 (1.584%)<br>• class: 305 (4.882%)<br>• phylum: 707 (11.317%)<br>• superkingdom: 293 (4.69%)<br>• root: 690 (11.045%)	• Crenobacter sedimenti [taxid 2705474]: 2 (0.032%)<br>• Chromobacterium haemolyticum [taxid 394935]: 2 (0.032%)<br>• Thiorhodospira sibirica [taxid 154347]: 1 (0.016%)<br>• Cupriavidus oxalaticus [taxid 96344]: 1 (0.016%)<br>• Monosiga brevicollis [taxid 81824]: 1 (0.016%)<br>• Aspergillus novoparasiticus [taxid 986946]: 1 (0.016%)<br>• Kinneretia asaccharophila [taxid 582607]: 1 (0.016%)<br>• Pseudomonas argentinensis [taxid 289370]: 1 (0.016%)<br>• other: 30 (0.48%)
Benchmark OTU ID: `CP004029-_Proteobacteria`<br>OTU taxon: Lawsonia intracellularis N343 [taxid 1234378]<br>Expected: Lawsonia intracellularis [taxid 29546] (species)<br>Number of reads: 2395<br>Number of identified reads: 2340 (97.703%)	• **species: 1851 (77.286%)**<br>• genus: 0 (0.0%)<br>• family: 37 (1.544%)<br>• order: 5 (0.208%)<br>• class: 8 (0.334%)<br>• phylum: 54 (2.254%)<br>• superkingdom: 115 (4.801%)<br>• root: 267 (11.148%)	• Rickettsiales endosymbiont of Peranema trichophorum [taxid 2486577]: 1 (0.041%)<br>• Desulfocurvibacter africanus [taxid 873]: 1 (0.041%)<br>• Durinskia baltica [taxid 400756]: 1 (0.041%)
Benchmark OTU ID: `AM180252-_Proteobacteria`<br>OTU taxon: Lawsonia intracellularis PHE/MN1-00 [taxid 363253]<br>Expected: Lawsonia intracellularis [taxid 29546] (species)<br>Number of reads: 2396<br>Number of identified reads: 2340 (97.662%)	• **species: 1856 (77.462%)**<br>• genus: 0 (0.0%)<br>• family: 31 (1.293%)<br>• order: 9 (0.375%)<br>• class: 12 (0.5%)<br>• phylum: 50 (2.086%)<br>• superkingdom: 116 (4.841%)<br>• root: 266 (11.101%)	• Desulfovibrio litoralis [taxid 466107]: 1 (0.041%)<br>• Deinococcus peraridilitoris [taxid 432329]: 1 (0.041%)<br>• Paraperlucidibaca baekdonensis [taxid 748120]: 1 (0.041%)
Benchmark OTU ID: `CP002305-_Bacteroidetes`<br>OTU taxon: Leadbetterella byssophila DSM 17132 [taxid 649349]<br>Expected: Leadbetterella byssophila [taxid 316068] (species)<br>Number of reads: 27042<br>Number of identified reads: 26825 (99.197%)	• **species: 19518 (72.176%)**<br>• genus: 0 (0.0%)<br>• family: 383 (1.416%)<br>• order: 253 (0.935%)<br>• class: 0 (0.0%)<br>• phylum: 2376 (8.786%)<br>• superkingdom: 1323 (4.892%)<br>• root: 2948 (10.901%)	• Avrilella dinanensis [taxid 2008672]: 11 (0.04%)<br>• Arachidicoccus soli [taxid 2341117]: 4 (0.014%)<br>• Ephemera danica [taxid 1049336]: 3 (0.011%)<br>• Chryseobacterium lacus [taxid 2058346]: 3 (0.011%)<br>• Aquirufa nivalisilvae [taxid 2516557]: 2 (0.007%)<br>• Solitalea koreensis [taxid 543615]: 2 (0.007%)<br>• Sphingobacterium spiritivorum [taxid 258]: 2 (0.007%)<br>• Pontibacter mucosus [taxid 1649266]: 2 (0.007%)<br>• other: 90 (0.332%)

Operational Taxonomic Unit (OTU)	Correct identifications	Wrong or overspecific identifications at species rank
Benchmark OTU ID: `CP000675-_Pathogens`<br>OTU taxon: Legionella pneumophila str. Corby [taxid 400673]<br>Expected: Legionella pneumophila [taxid 446] (species)<br>Number of reads: 7727<br>Number of identified reads: 7667 (99.223%)	• **species: 4713 (60.993%)**<br>• genus: 1101 (14.248%)<br>• family: 257 (3.325%)<br>• order: 16 (0.207%)<br>• class: 168 (2.174%)<br>• phylum: 142 (1.837%)<br>• superkingdom: 282 (3.649%)<br>• root: 982 (12.708%)	• Legionella parisiensis [taxid 45071]: 40 (0.517%)<br>• Legionella norrlandica [taxid 1498499]: 23 (0.297%)<br>• Legionella jordanis [taxid 456]: 11 (0.142%)<br>• Legionella gratiana [taxid 45066]: 8 (0.103%)<br>• Legionella waltersii [taxid 66969]: 6 (0.077%)<br>• Legionella drancourtii [taxid 168933]: 4 (0.051%)<br>• Legionella fallonii [taxid 96230]: 3 (0.038%)<br>• Legionella tucsonensis [taxid 40335]: 3 (0.038%)<br>• other: 46 (0.595%)
Benchmark OTU ID: `CP003885-_Pathogens`<br>OTU taxon: Legionella pneumophila subsp. pneumophila LPE509 [taxid 1312904]<br>Expected: Legionella pneumophila [taxid 446] (species)<br>Number of reads: 7372<br>Number of identified reads: 7314 (99.213%)	• **species: 4760 (64.568%)**<br>• genus: 976 (13.239%)<br>• family: 251 (3.404%)<br>• order: 28 (0.379%)<br>• class: 157 (2.129%)<br>• phylum: 143 (1.939%)<br>• superkingdom: 190 (2.577%)<br>• root: 806 (10.933%)	• Legionella waltersii [taxid 66969]: 3 (0.04%)<br>• Legionella worsleiensis [taxid 45076]: 3 (0.04%)<br>• Legionella quateirensis [taxid 45072]: 3 (0.04%)<br>• Legionella qingyii [taxid 2184757]: 2 (0.027%)<br>• Legionella sainthelensi [taxid 28087]: 2 (0.027%)<br>• Legionella lansingensis [taxid 45067]: 2 (0.027%)<br>• Legionella fallonii [taxid 96230]: 2 (0.027%)<br>• Legionella massiliensis [taxid 1034943]: 1 (0.013%)<br>• other: 26 (0.352%)
Benchmark OTU ID: `AE016822-_Actinobacteria`<br>OTU taxon: Leifsonia xyli subsp. xyli str. CTCB07 [taxid 281090]<br>Expected: Leifsonia xyli [taxid 1575] (species)<br>Number of reads: 10142<br>Number of identified reads: 10093 (99.516%)	• **species: 5493 (54.16%)**<br>• genus: 441 (4.348%)<br>• family: 1113 (10.974%)<br>• order: 196 (1.932%)<br>• class: 647 (6.379%)<br>• phylum: 8 (0.078%)<br>• superkingdom: 696 (6.862%)<br>• root: 1485 (14.642%)	• Pseudoclavibacter endophyticus [taxid 1778590]: 4 (0.039%)<br>• Corynebacterium variabile [taxid 1727]: 3 (0.029%)<br>• Glaciihabitans tibetensis [taxid 1266600]: 2 (0.019%)<br>• Subtercola vilae [taxid 2056433]: 2 (0.019%)<br>• Rathayibacter toxicus [taxid 145458]: 2 (0.019%)<br>• Klugiella xanthotipulae [taxid 244735]: 2 (0.019%)<br>• Micromonospora zingiberis [taxid 2053011]: 1 (0.009%)<br>• Brevilactibacter flavus [taxid 2072026]: 1 (0.009%)<br>• other: 52 (0.512%)
Benchmark OTU ID: `CP003946-_Cyanobacteria`<br>OTU taxon: Leptolyngbya sp. PCC 7376 [taxid 111781]<br>Expected: Leptolyngbya [taxid 47251] (genus)<br>Number of reads: 29971<br>Number of identified reads: 29806 (99.449%)	• **genus: 20274 (67.645%)**<br>• family: 23 (0.076%)<br>• order: 1968 (6.566%)<br>• phylum: 1508 (5.031%)<br>• superkingdom: 1733 (5.782%)<br>• root: 4233 (14.123%)	• Limnothrix rosea [taxid 71188]: 55 (0.183%)<br>• Leptolyngbya sp. SIOISBB [taxid 2607771]: 50 (0.166%)<br>• Leptolyngbya sp. Heron Island J [taxid 1385935]: 20 (0.066%)<br>• Leptolyngbya sp. 'hensonii' [taxid 1922337]: 11 (0.036%)<br>• Merismopedia glauca [taxid 292586]: 2 (0.006%)<br>• Medicago truncatula [taxid 3880]: 2 (0.006%)<br>• other: 81 (0.27%)

Operational Taxonomic Unit (OTU)	Correct identifications	Wrong or overspecific identifications at species rank
Benchmark OTU ID: `CP002919-_Nitrospira`<br>OTU taxon: Leptospirillum ferriphilum ML-04 [taxid 1048260]<br>Expected: Leptospirillum ferriphilum [taxid 178606] (species)<br>Number of reads: 20853<br>Number of identified reads: 20784 (99.669%)	• species: 4001 (19.186%)<br>• **genus: 11213 (53.771%)**<br>• family: 8 (0.038%)<br>• order: 1 (0.004%)<br>• class: 0 (0.0%)<br>• phylum: 24 (0.115%)<br>• superkingdom: 1776 (8.516%)<br>• root: 3729 (17.882%)	• Leptospirillum ferrodiazotrophum [taxid 412449]: 14 (0.067%)<br>• Leptospirillum ferrooxidans [taxid 180]: 13 (0.062%)<br>• Leptospirillum sp. Group IV 'UBA BS' [taxid 1260983]: 3 (0.014%)<br>• Citrobacter koseri [taxid 545]: 2 (0.009%)<br>• [Ruminococcus] torques [taxid 33039]: 2 (0.009%)<br>• Helicobacter pylori [taxid 210]: 2 (0.009%)<br>• Thalassiosira rotula [taxid 49265]: 2 (0.009%)<br>• Thermodesulfatator indicus [taxid 171695]: 1 (0.004%)<br>• other: 43 (0.206%)
Benchmark OTU ID: `AP012342-_Nitrospira`<br>OTU taxon: Leptospirillum ferrooxidans C2-3 [taxid 1162668]<br>Expected: Leptospirillum ferrooxidans [taxid 180] (species)<br>Number of reads: 22492<br>Number of identified reads: 22420 (99.679%)	• **species: 14403 (64.036%)**<br>• genus: 476 (2.116%)<br>• family: 10 (0.044%)<br>• order: 0 (0.0%)<br>• class: 0 (0.0%)<br>• phylum: 19 (0.084%)<br>• superkingdom: 2217 (9.856%)<br>• root: 5256 (23.368%)	• Leptospirillum ferriphilum [taxid 178606]: 16 (0.071%)<br>• Leptospirillum ferrodiazotrophum [taxid 412449]: 8 (0.035%)<br>• Maridesulfovibrio hydrothermalis [taxid 191026]: 4 (0.017%)<br>• bacterium [taxid 1869227]: 3 (0.013%)<br>• Skeletonema menzelii [taxid 216823]: 2 (0.008%)<br>• Streptomyces qinglanensis [taxid 943816]: 1 (0.004%)<br>• Methanococcoides vulcani [taxid 1353158]: 1 (0.004%)<br>• Anditalea andensis [taxid 1048983]: 1 (0.004%)<br>• other: 46 (0.204%)
Benchmark OTU ID: `CP001013-_Proteobacteria`<br>OTU taxon: Leptothrix cholodnii SP-6 [taxid 395495]<br>Expected: Leptothrix cholodnii [taxid 34029] (species)<br>Number of reads: 10162<br>Number of identified reads: 10139 (99.773%)	• **species: 6593 (64.878%)**<br>• genus: 107 (1.052%)<br>• order: 1274 (12.536%)<br>• class: 263 (2.588%)<br>• phylum: 724 (7.124%)<br>• superkingdom: 485 (4.772%)<br>• root: 690 (6.79%)	• Sphaerotilus hippei [taxid 744406]: 2 (0.019%)<br>• Ideonella dechloratans [taxid 36863]: 2 (0.019%)<br>• Aquabacterium pictum [taxid 2315236]: 2 (0.019%)<br>• Sphaerotilus natans [taxid 34103]: 2 (0.019%)<br>• Pseudomonas kirkiae [taxid 2211392]: 1 (0.009%)<br>• Gimesia alba [taxid 2527973]: 1 (0.009%)<br>• Serratia symbiotica [taxid 138074]: 1 (0.009%)<br>• other: 37 (0.364%)
Benchmark OTU ID: `CP003851-_Firmicutes`<br>OTU taxon: Leuconostoc carnosum JB16 [taxid 1229758]<br>Expected: Leuconostoc carnosum [taxid 1252] (species)<br>Number of reads: 1863<br>Number of identified reads: 1851 (99.355%)	• **species: 1272 (68.276%)**<br>• genus: 189 (10.144%)<br>• family: 55 (2.952%)<br>• order: 77 (4.133%)<br>• class: 30 (1.61%)<br>• phylum: 13 (0.697%)<br>• superkingdom: 86 (4.616%)<br>• root: 128 (6.87%)	• Leuconostoc mesenteroides [taxid 1245]: 2 (0.107%)<br>• Leptospirillum sp. Group IV 'UBA BS' [taxid 1260983]: 1 (0.053%)<br>• Lactiplantibacillus plantarum [taxid 1590]: 1 (0.053%)<br>• Vagococcus entomophilus [taxid 1160095]: 1 (0.053%)<br>• Mycoplasma canadense [taxid 29554]: 1 (0.053%)<br>• Pacificimonas flava [taxid 1234595]: 1 (0.053%)<br>• Lacticaseibacillus casei [taxid 1582]: 1 (0.053%)<br>• Streptococcus suis [taxid 1307]: 1 (0.053%)

Operational Taxonomic Unit (OTU)	Correct identifications	Wrong or overspecific identifications at species rank
Benchmark OTU ID: `CP003839-_Firmicutes`<br>OTU taxon: Leuconostoc gelidum JB7 [taxid 1229756]<br>Expected: Leuconostoc gelidum [taxid 1244] (species)<br>Number of reads: 2260<br>Number of identified reads: 2235 (98.893%)	• species: 809 (35.796%)<br>• **genus: 944 (41.769%)**<br>• family: 41 (1.814%)<br>• order: 93 (4.115%)<br>• class: 20 (0.884%)<br>• phylum: 17 (0.752%)<br>• superkingdom: 100 (4.424%)<br>• root: 207 (9.159%)	• Leuconostoc inhae [taxid 178001]: 11 (0.486%)<br>• Leuconostoc pseudomesenteroides [taxid 33968]: 5 (0.221%)<br>• Leuconostoc citreum [taxid 33964]: 4 (0.176%)<br>• Leuconostoc kimchii [taxid 136609]: 3 (0.132%)<br>• Leuconostoc lactis [taxid 1246]: 2 (0.088%)<br>• Leuconostoc fallax [taxid 1251]: 2 (0.088%)<br>• Leuconostoc mesenteroides [taxid 1245]: 1 (0.044%)<br>• Weissella soli [taxid 155866]: 1 (0.044%)<br>• other: 4 (0.176%)
Benchmark OTU ID: `CP003101-_Firmicutes`<br>OTU taxon: Leuconostoc mesenteroides subsp. mesenteroides J18 [taxid 1107880]<br>Expected: Leuconostoc mesenteroides [taxid 1245] (species)<br>Number of reads: 2265<br>Number of identified reads: 2250 (99.337%)	• species: 772 (34.083%)<br>• **genus: 983 (43.399%)**<br>• family: 82 (3.62%)<br>• order: 85 (3.752%)<br>• class: 26 (1.147%)<br>• phylum: 8 (0.353%)<br>• superkingdom: 78 (3.443%)<br>• root: 212 (9.359%)	• Leuconostoc suionicum [taxid 1511761]: 13 (0.573%)<br>• Leuconostoc citreum [taxid 33964]: 6 (0.264%)<br>• Leuconostoc litchii [taxid 1981069]: 4 (0.176%)<br>• Leuconostoc carnosum [taxid 1252]: 3 (0.132%)<br>• Weissella koreensis [taxid 165096]: 1 (0.044%)<br>• Lupinus albus [taxid 3870]: 1 (0.044%)<br>• Enterobius vermicularis [taxid 51028]: 1 (0.044%)<br>• Leuconostoc gelidum [taxid 1244]: 1 (0.044%)<br>• other: 12 (0.529%)
Benchmark OTU ID: `CP002898-_Firmicutes`<br>OTU taxon: Leuconostoc sp. C2 [taxid 979982]<br>Expected: Leuconostoc [taxid 1243] (genus)<br>Number of reads: 2235<br>Number of identified reads: 2217 (99.194%)	• **genus: 1701 (76.107%)**<br>• family: 55 (2.46%)<br>• order: 80 (3.579%)<br>• class: 18 (0.805%)<br>• phylum: 11 (0.492%)<br>• superkingdom: 102 (4.563%)<br>• root: 244 (10.917%)	• **Leuconostoc kimchii [taxid 136609]: 1418 (63.445%)**<br>• Leuconostoc pseudomesenteroides [taxid 33968]: 10 (0.447%)<br>• Leuconostoc carnosum [taxid 1252]: 7 (0.313%)<br>• Leuconostoc mesenteroides [taxid 1245]: 5 (0.223%)<br>• Fructilactobacillus sanfranciscensis [taxid 1625]: 2 (0.089%)<br>• Leuconostoc lactis [taxid 1246]: 2 (0.089%)<br>• Fructobacillus tropaeoli [taxid 709323]: 1 (0.044%)<br>• other: 10 (0.447%)
Benchmark OTU ID: `AE017262-_Firmicutes`<br>OTU taxon: Listeria monocytogenes serotype 4b str. F2365 [taxid 265669]<br>Expected: Listeria monocytogenes [taxid 1639] (species)<br>Number of reads: 3879<br>Number of identified reads: 3852 (99.303%)	• **species: 1797 (46.326%)**<br>• genus: 1461 (37.664%)<br>• family: 2 (0.051%)<br>• order: 99 (2.552%)<br>• class: 74 (1.907%)<br>• phylum: 42 (1.082%)<br>• superkingdom: 129 (3.325%)<br>• root: 245 (6.316%)	• Listeria innocua [taxid 1642]: 3 (0.077%)<br>• Listeria ivanovii [taxid 1638]: 2 (0.051%)<br>• Listeria welshimeri [taxid 1643]: 2 (0.051%)<br>• Listeria grayi [taxid 1641]: 2 (0.051%)<br>• Listeria seeligeri [taxid 1640]: 1 (0.025%)<br>• Pediococcus acidilactici [taxid 1254]: 1 (0.025%)<br>• Listeria booriae [taxid 1552123]: 1 (0.025%)<br>• Streptococcus anginosus [taxid 1328]: 1 (0.025%)<br>• other: 7 (0.18%)

Operational Taxonomic Unit (OTU)	Correct identifications	Wrong or overspecific identifications at species rank
Benchmark OTU ID: `AP009484-_Firmicutes`<br>OTU taxon: Macrococcus caseolyticus JCSC5402 [taxid 458233]<br>Expected: Macrococcus caseolyticus [taxid 69966] (species)<br>Number of reads: 2595<br>Number of identified reads: 2562 (98.728%)	• **species: 1274 (49.094%)**<br>• genus: 566 (21.811%)<br>• family: 208 (8.015%)<br>• order: 105 (4.046%)<br>• class: 40 (1.541%)<br>• phylum: 27 (1.04%)<br>• superkingdom: 124 (4.778%)<br>• root: 216 (8.323%)	• Macrococcus canis [taxid 1855823]: 9 (0.346%)<br>• Macrococcus brunensis [taxid 198483]: 2 (0.077%)<br>• Lupinus albus [taxid 3870]: 1 (0.038%)<br>• Aquabacterium commune [taxid 70586]: 1 (0.038%)<br>• Carnobacterium maltaromaticum [taxid 2751]: 1 (0.038%)<br>• Oceanibacterium hippocampi [taxid 745714]: 1 (0.038%)<br>• Lactobacillus amylolyticus [taxid 83683]: 1 (0.038%)<br>• Staphylococcus haemolyticus [taxid 1283]: 1 (0.038%)<br>• other: 4 (0.154%)
Benchmark OTU ID: `CP000471-_Proteobacteria`<br>OTU taxon: Magnetococcus marinus MC-1 [taxid 156889]<br>Expected: Magnetococcus marinus [taxid 1124597] (species)<br>Number of reads: 9735<br>Number of identified reads: 9661 (99.239%)	• **species: 7302 (75.007%)**<br>• genus: 131 (1.345%)<br>• family: 50 (0.513%)<br>• order: 0 (0.0%)<br>• class: 111 (1.14%)<br>• phylum: 384 (3.944%)<br>• superkingdom: 359 (3.687%)<br>• root: 1312 (13.477%)	• Candidatus Magnetococcus massalia [taxid 451514]: 8 (0.082%)<br>• Magnetofaba australis [taxid 1472297]: 1 (0.01%)<br>• Scrippsiella trochoidea [taxid 71861]: 1 (0.01%)<br>• Paracoccus chinensis [taxid 525640]: 1 (0.01%)<br>• Lupinus albus [taxid 3870]: 1 (0.01%)<br>• Auraticoccus cholistanensis [taxid 2656650]: 1 (0.01%)<br>• Symbiodinium microadriaticum [taxid 2951]: 1 (0.01%)<br>• Methylomonas methanica [taxid 421]: 1 (0.01%)<br>• other: 4 (0.041%)
Benchmark OTU ID: `CP005383-_Proteobacteria`<br>OTU taxon: Mannheimia haemolytica M42548 [taxid 1316932]<br>Expected: Mannheimia haemolytica [taxid 75985] (species)<br>Number of reads: 5263<br>Number of identified reads: 5223 (99.239%)	• species: 172 (3.268%)<br>• genus: 51 (0.969%)<br>• **family: 3617 (68.725%)**<br>• order: 2 (0.038%)<br>• class: 396 (7.524%)<br>• phylum: 159 (3.021%)<br>• superkingdom: 230 (4.37%)<br>• root: 585 (11.115%)	• Mannheimia granulomatis [taxid 85402]: 7 (0.133%)<br>• Mannheimia varigena [taxid 85404]: 7 (0.133%)<br>• Pasteurella multocida [taxid 747]: 6 (0.114%)<br>• Haemophilus influenzae [taxid 727]: 5 (0.095%)<br>• Actinobacillus ureae [taxid 723]: 4 (0.076%)<br>• Nicoletella semolina [taxid 271160]: 4 (0.076%)<br>• Haemophilus sputorum [taxid 1078480]: 3 (0.057%)<br>• Actinobacillus indolicus [taxid 51049]: 3 (0.057%)<br>• other: 67 (1.273%)
Benchmark OTU ID: `CP004752-_Proteobacteria`<br>OTU taxon: Mannheimia haemolytica USDA-ARS-USMARC-183 [taxid 1249531]<br>Expected: Mannheimia haemolytica [taxid 75985] (species)<br>Number of reads: 5097<br>Number of identified reads: 5063 (99.332%)	• species: 245 (4.806%)<br>• genus: 61 (1.196%)<br>• **family: 3484 (68.353%)**<br>• order: 0 (0.0%)<br>• class: 377 (7.396%)<br>• phylum: 141 (2.766%)<br>• superkingdom: 189 (3.708%)<br>• root: 559 (10.967%)	• Mannheimia varigena [taxid 85404]: 12 (0.235%)<br>• Mannheimia granulomatis [taxid 85402]: 7 (0.137%)<br>• Bibersteinia trehalosi [taxid 47735]: 5 (0.098%)<br>• Glaesserella parasuis [taxid 738]: 4 (0.078%)<br>• Pararhodospirillum photometricum [taxid 1084]: 4 (0.078%)<br>• Rodentibacter myodis [taxid 1907939]: 3 (0.058%)<br>• Salmonella enterica [taxid 28901]: 3 (0.058%)<br>• Otariodibacter oris [taxid 1032623]: 3 (0.058%)<br>• other: 65 (1.275%)

<table>
<tr><th>Operational Taxonomic Unit (OTU)</th><th>Correct identifications</th><th>Wrong or overspecific identifications at species rank</th></tr>
<tr><td>Benchmark OTU ID: <code>CP004753-_Proteobacteria</code>
OTU taxon: Mannheimia haemolytica USDA-ARS-USMARC-185 [taxid 1249526]
Expected: Mannheimia haemolytica [taxid 75985] (species)
Number of reads: 4841
Number of identified reads: 4809 (99.338%)</td><td>• species: 244 (5.04%)
• genus: 58 (1.198%)
• family: 3391 (70.047%)
• order: 0 (0.0%)
• class: 385 (7.952%)
• phylum: 113 (2.334%)
• superkingdom: 178 (3.676%)
• root: 430 (8.882%)</td><td>• Mannheimia granulomatis [taxid 85402]: 14 (0.289%)
• Bibersteinia trehalosi [taxid 47735]: 5 (0.103%)
• Avibacterium paragallinarum [taxid 728]: 5 (0.103%)
• Salmonella enterica [taxid 28901]: 4 (0.082%)
• Pasteurella bettyae [taxid 752]: 4 (0.082%)
• Aggregatibacter aphrophilus [taxid 732]: 3 (0.061%)
• Mannheimia varigena [taxid 85404]: 3 (0.061%)
• Haemophilus haemolyticus [taxid 726]: 3 (0.061%)
• other: 64 (1.322%)</td></tr>
<tr><td>Benchmark OTU ID: <code>CP000514-_Proteobacteria</code>
OTU taxon: Marinobacter hydrocarbonoclasticus VT8 [taxid 351348]
Expected: Marinobacter hydrocarbonoclasticus [taxid 2743] (species)
Number of reads: 8851
Number of identified reads: 8843 (99.909%)</td><td>• species: 929 (10.495%)
• genus: 5185 (58.58%)
• family: 28 (0.316%)
• order: 18 (0.203%)
• class: 1077 (12.168%)
• phylum: 373 (4.214%)
• superkingdom: 324 (3.66%)
• root: 903 (10.202%)</td><td>• Marinobacter fuscus [taxid 2109942]: 8 (0.09%)
• Marinobacter salarius [taxid 1420917]: 6 (0.067%)
• Marinobacter halophilus [taxid 1323740]: 5 (0.056%)
• Marinobacter excellens [taxid 218670]: 5 (0.056%)
• Marinobacter persicus [taxid 930118]: 4 (0.045%)
• Marinobacter mobilis [taxid 488533]: 3 (0.033%)
• Marinobacter vinifirmus [taxid 355591]: 3 (0.033%)
• Marinobacter zhejiangensis [taxid 488535]: 3 (0.033%)
• other: 65 (0.734%)</td></tr>
<tr><td>Benchmark OTU ID: <code>CP003735-_Proteobacteria</code>
OTU taxon: Marinobacter sp. BSs20148 [taxid 490759]
Expected: Marinobacter [taxid 2742] (genus)
Number of reads: 8260
Number of identified reads: 8253 (99.915%)</td><td>• genus: 5721 (69.261%)
• family: 26 (0.314%)
• order: 13 (0.157%)
• class: 753 (9.116%)
• phylum: 363 (4.394%)
• superkingdom: 324 (3.922%)
• root: 1040 (12.59%)</td><td>• Marinobacter psychrophilus [taxid 330734]: 59 (0.714%)
• Marinobacter algicola [taxid 236100]: 15 (0.181%)
• Marinobacter santoriniensis [taxid 523742]: 3 (0.036%)
• Marinobacter antarcticus [taxid 564117]: 3 (0.036%)
• Marinobacter segnicrescens [taxid 430453]: 3 (0.036%)
• Marinobacter confluentis [taxid 1697557]: 2 (0.024%)
• Marinobacter daqiaonensis [taxid 650891]: 2 (0.024%)
• other: 46 (0.556%)</td></tr>
<tr><td>Benchmark OTU ID: <code>CP002583-_Proteobacteria</code>
OTU taxon: Marinomonas mediterranea MMB-1 [taxid 717774]
Expected: Marinomonas mediterranea [taxid 119864] (species)
Number of reads: 9656
Number of identified reads: 9612 (99.544%)</td><td>• species: 5613 (58.129%)
• genus: 1391 (14.405%)
• family: 34 (0.352%)
• order: 33 (0.341%)
• class: 683 (7.073%)
• phylum: 327 (3.386%)
• superkingdom: 340 (3.521%)
• root: 1183 (12.251%)</td><td>• Marinomonas balearica [taxid 491947]: 19 (0.196%)
• Marinomonas spartinae [taxid 1792290]: 4 (0.041%)
• Marinomonas aquiplantarum [taxid 491951]: 2 (0.02%)
• Marinomonas ushuaiensis [taxid 263818]: 2 (0.02%)
• Marinomonas profundimaris [taxid 1208321]: 2 (0.02%)
• Salmonella enterica [taxid 28901]: 2 (0.02%)
• Pseudomonas cedrina [taxid 651740]: 1 (0.01%)
• Lysinibacillus acetophenoni [taxid 614649]: 1 (0.01%)
• other: 36 (0.372%)</td></tr>
</table>

Operational Taxonomic Unit (OTU)	Correct identifications	Wrong or overspecific identifications at species rank
Benchmark OTU ID: `CP000749-_Proteobacteria`<br>OTU taxon: Marinomonas sp. MWYL1 [taxid 400668]<br>Expected: Marinomonas [taxid 28253] (genus)<br>Number of reads: 10592<br>Number of identified reads: 10535 (99.461%)	• **genus: 7965 (75.198%)**<br>• family: 65 (0.613%)<br>• order: 42 (0.396%)<br>• class: 713 (6.731%)<br>• phylum: 372 (3.512%)<br>• superkingdom: 348 (3.285%)<br>• root: 1022 (9.648%)	• Marinomonas rhizomae [taxid 491948]: 21 (0.198%)<br>• Marinomonas polaris [taxid 293552]: 19 (0.179%)<br>• Marinomonas primoryensis [taxid 178399]: 18 (0.169%)<br>• Marinomonas foliarum [taxid 491950]: 13 (0.122%)<br>• Marinomonas spartinae [taxid 1792290]: 13 (0.122%)<br>• Marinomonas alcarazii [taxid 491949]: 12 (0.113%)<br>• Marinomonas profundimaris [taxid 1208321]: 11 (0.103%)<br>• other: 73 (0.689%)
Benchmark OTU ID: `CP003358-_Proteobacteria`<br>OTU taxon: Mesorhizobium australicum WSM2073 [taxid 754035]<br>Expected: Mesorhizobium australicum [taxid 536018] (species)<br>Number of reads: 13067<br>Number of identified reads: 13026 (99.686%)	• species: 996 (7.622%)<br>• **genus: 9051 (69.266%)**<br>• family: 73 (0.558%)<br>• order: 654 (5.004%)<br>• class: 266 (2.035%)<br>• phylum: 264 (2.02%)<br>• superkingdom: 485 (3.711%)<br>• root: 1227 (9.39%)	• Mesorhizobium loti [taxid 381]: 13 (0.099%)<br>• Mesorhizobium plurifarium [taxid 69974]: 11 (0.084%)<br>• Mesorhizobium qingshengii [taxid 1165689]: 7 (0.053%)<br>• Mesorhizobium japonicum [taxid 2066070]: 7 (0.053%)<br>• Mesorhizobium albiziae [taxid 335020]: 6 (0.045%)<br>• Mesorhizobium opportunistum [taxid 593909]: 6 (0.045%)<br>• Mesorhizobium tamadayense [taxid 425306]: 5 (0.038%)<br>• Mesorhizobium metallidurans [taxid 489722]: 4 (0.03%)<br>• other: 74 (0.566%)
Benchmark OTU ID: `CP002656-_Crenarchaeota`<br>OTU taxon: Metallosphaera cuprina Ar-4 [taxid 1006006]<br>Expected: Metallosphaera cuprina [taxid 1006005] (species)<br>Number of reads: 3726<br>Number of identified reads: 3643 (97.772%)	• **species: 2307 (61.916%)**<br>• genus: 181 (4.857%)<br>• family: 125 (3.354%)<br>• order: 4 (0.107%)<br>• class: 10 (0.268%)<br>• phylum: 0 (0.0%)<br>• superkingdom: 18 (0.483%)<br>• root: 958 (25.711%)	• Hyperthermus butylicus [taxid 54248]: 1 (0.026%)<br>• Steinernema carpocapsae [taxid 34508]: 1 (0.026%)<br>• Metallosphaera tengchongensis [taxid 1532350]: 1 (0.026%)<br>• Stauroneis constricta [taxid 265584]: 1 (0.026%)<br>• Metallosphaera hakonensis [taxid 79601]: 1 (0.026%)<br>• Helicobacter pylori [taxid 210]: 1 (0.026%)<br>• Acidianus manzaensis [taxid 282676]: 1 (0.026%)<br>• Metallosphaera sedula [taxid 43687]: 1 (0.026%)<br>• other: 8 (0.214%)
Benchmark OTU ID: `CP000682-_Crenarchaeota`<br>OTU taxon: Metallosphaera sedula DSM 5348 [taxid 399549]<br>Expected: Metallosphaera sedula [taxid 43687] (species)<br>Number of reads: 4604<br>Number of identified reads: 4511 (97.98%)	• species: 90 (1.954%)<br>• **genus: 2943 (63.922%)**<br>• family: 167 (3.627%)<br>• order: 4 (0.086%)<br>• class: 16 (0.347%)<br>• phylum: 0 (0.0%)<br>• superkingdom: 27 (0.586%)<br>• root: 1219 (26.476%)	• Metallosphaera prunae [taxid 47304]: 14 (0.304%)<br>• Metallosphaera hakonensis [taxid 79601]: 11 (0.238%)<br>• Metallosphaera cuprina [taxid 1006005]: 5 (0.108%)<br>• Metallosphaera tengchongensis [taxid 1532350]: 4 (0.086%)<br>• Sulfurisphaera tokodaii [taxid 111955]: 2 (0.043%)<br>• Metallosphaera yellowstonensis [taxid 1111107]: 2 (0.043%)<br>• Sulfuracidifex tepidarius [taxid 1294262]: 1 (0.021%)<br>• Rubripirellula reticaptiva [taxid 2528013]: 1 (0.021%)<br>• other: 9 (0.195%)

Operational Taxonomic Unit (OTU)	Correct identifications	Wrong or overspecific identifications at species rank
Benchmark OTU ID: `CP002551-_Euryarchaeota`<br>OTU taxon: Methanobacterium lacus [taxid 877455]<br>Expected: Methanobacterium lacus [taxid 877455] (species)<br>Number of reads: 2316<br>Number of identified reads: 2267 (97.884%)	• **species: 1236 (53.367%)**<br>• genus: 349 (15.069%)<br>• family: 37 (1.597%)<br>• order: 16 (0.69%)<br>• class: 0 (0.0%)<br>• phylum: 18 (0.777%)<br>• superkingdom: 7 (0.302%)<br>• root: 571 (24.654%)	• Candidatus Bathyarchaeota archaeon [taxid 2026714]: 1 (0.043%)<br>• Amorphochlora amoebiformis [taxid 1561963]: 1 (0.043%)<br>• Sporomusa acidovorans [taxid 112900]: 1 (0.043%)<br>• Geobacter uraniireducens [taxid 351604]: 1 (0.043%)<br>• Methanobacterium congolense [taxid 118062]: 1 (0.043%)<br>• Nyssa sinensis [taxid 561372]: 1 (0.043%)<br>• Ruegeria sp. ANG-R [taxid 1577903]: 1 (0.043%)
Benchmark OTU ID: `CP002772-_Euryarchaeota`<br>OTU taxon: Methanobacterium paludis [taxid 868131]<br>Expected: Methanobacterium paludis [taxid 868131] (species)<br>Number of reads: 2277<br>Number of identified reads: 2218 (97.408%)	• **species: 1437 (63.109%)**<br>• genus: 67 (2.942%)<br>• family: 43 (1.888%)<br>• order: 19 (0.834%)<br>• class: 0 (0.0%)<br>• phylum: 7 (0.307%)<br>• superkingdom: 14 (0.614%)<br>• root: 609 (26.745%)	• Methanobacterium congolense [taxid 118062]: 2 (0.087%)<br>• Gossypium tomentosum [taxid 34277]: 1 (0.043%)<br>• Hwanghaeella grinnelliae [taxid 2500179]: 1 (0.043%)<br>• Eutreptiella gymnastica [taxid 73025]: 1 (0.043%)<br>• Methanosphaera sp. BMS [taxid 1789762]: 1 (0.043%)<br>• Cupriavidus basilensis [taxid 68895]: 1 (0.043%)
Benchmark OTU ID: `CP001696-_Euryarchaeota`<br>OTU taxon: Methanocaldococcus fervens AG86 [taxid 573064]<br>Expected: Methanocaldococcus fervens [taxid 83171] (species)<br>Number of reads: 1162<br>Number of identified reads: 1138 (97.934%)	• **species: 633 (54.475%)**<br>• genus: 205 (17.641%)<br>• family: 20 (1.721%)<br>• order: 21 (1.807%)<br>• class: 0 (0.0%)<br>• phylum: 14 (1.204%)<br>• superkingdom: 15 (1.29%)<br>• root: 215 (18.502%)	• Methanocaldococcus bathoardescens [taxid 1301915]: 4 (0.344%)<br>• Methanocaldococcus infernus [taxid 67760]: 2 (0.172%)<br>• Methanocaldococcus villosus [taxid 667126]: 2 (0.172%)<br>• Methanocaldococcus jannaschii [taxid 2190]: 1 (0.086%)<br>• Methanophagales archaeon [taxid 2056316]: 1 (0.086%)<br>• Methanococcus maripaludis [taxid 39152]: 1 (0.086%)<br>• Candidatus Korarchaeota archaeon [taxid 2056630]: 1 (0.086%)<br>• Methanocaldococcus vulcanius [taxid 73913]: 1 (0.086%)
Benchmark OTU ID: `L77117-_Euryarchaeota`<br>OTU taxon: Methanocaldococcus jannaschii DSM 2661 [taxid 243232]<br>Expected: Methanocaldococcus jannaschii [taxid 2190] (species)<br>Number of reads: 1351<br>Number of identified reads: 1315 (97.335%)	• **species: 574 (42.487%)**<br>• genus: 391 (28.941%)<br>• family: 31 (2.294%)<br>• order: 24 (1.776%)<br>• class: 0 (0.0%)<br>• phylum: 11 (0.814%)<br>• superkingdom: 18 (1.332%)<br>• root: 257 (19.022%)	• Methanocaldococcus vulcanius [taxid 73913]: 5 (0.37%)<br>• Methanocaldococcus bathoardescens [taxid 1301915]: 3 (0.222%)<br>• Methanotorris igneus [taxid 2189]: 2 (0.148%)<br>• Methanocaldococcus fervens [taxid 83171]: 2 (0.148%)<br>• Methanotorris formicicus [taxid 213185]: 1 (0.074%)<br>• Pygocentrus nattereri [taxid 42514]: 1 (0.074%)
Benchmark OTU ID: `AM114193-_Euryarchaeota`<br>OTU taxon: Methanocella arvoryzae MRE50 [taxid 351160]<br>Expected: Methanocella arvoryzae [taxid 1175445] (species)<br>Number of reads: 2942<br>Number of identified reads: 2931 (99.626%)	• **species: 1928 (65.533%)**<br>• genus: 58 (1.971%)<br>• family: 0 (0.0%)<br>• order: 0 (0.0%)<br>• class: 15 (0.509%)<br>• phylum: 14 (0.475%)<br>• superkingdom: 9 (0.305%)<br>• root: 838 (28.484%)	• Calorimonas adulescens [taxid 2606906]: 1 (0.033%)<br>• Pichia kudriavzevii [taxid 4909]: 1 (0.033%)

Operational Taxonomic Unit (OTU)	Correct identifications	Wrong or overspecific identifications at species rank
Benchmark OTU ID: `CP003243-_Euryarchaeota`<br>OTU taxon: Methanocella conradii HZ254 [taxid 1041930]<br>Expected: Methanocella conradii [taxid 1175444] (species)<br>Number of reads: 2100<br>Number of identified reads: 2087 (99.38%)	• **species: 1263 (60.142%)**<br>• genus: 80 (3.809%)<br>• family: 0 (0.0%)<br>• order: 0 (0.0%)<br>• class: 12 (0.571%)<br>• phylum: 15 (0.714%)<br>• superkingdom: 14 (0.666%)<br>• root: 659 (31.38%)	• Filamoeba nolandi [taxid 136452]: 1 (0.047%)<br>• Methanosarcina acetivorans [taxid 2214]: 1 (0.047%)<br>• Candidatus Thorarchaeota archaeon [taxid 2053491]: 1 (0.047%)<br>• Cryptococcus depauperatus [taxid 5208]: 1 (0.047%)<br>• Neolecta irregularis [taxid 48691]: 1 (0.047%)<br>• Desulfomonile tiedjei [taxid 2358]: 1 (0.047%)<br>• Filimonas effusa [taxid 2508721]: 1 (0.047%)<br>• Dictyostelium discoideum [taxid 44689]: 1 (0.047%)<br>• other: 3 (0.142%)
Benchmark OTU ID: `AP011532-_Euryarchaeota`<br>OTU taxon: Methanocella paludicola SANAE [taxid 304371]<br>Expected: Methanocella paludicola [taxid 570267] (species)<br>Number of reads: 2708<br>Number of identified reads: 2690 (99.335%)	• **species: 1736 (64.106%)**<br>• genus: 87 (3.212%)<br>• family: 0 (0.0%)<br>• order: 0 (0.0%)<br>• class: 18 (0.664%)<br>• phylum: 19 (0.701%)<br>• superkingdom: 14 (0.516%)<br>• root: 768 (28.36%)	• archaeon [taxid 1906665]: 1 (0.036%)<br>• Eutrema salsugineum [taxid 72664]: 1 (0.036%)<br>• Hadesarchaea archaeon [taxid 2250276]: 1 (0.036%)
Benchmark OTU ID: `CP000743-_Euryarchaeota`<br>OTU taxon: Methanococcus aeolicus Nankai-3 [taxid 419665]<br>Expected: Methanococcus aeolicus [taxid 42879] (species)<br>Number of reads: 1251<br>Number of identified reads: 1222 (97.681%)	• **species: 857 (68.505%)**<br>• genus: 10 (0.799%)<br>• family: 8 (0.639%)<br>• order: 18 (1.438%)<br>• class: 0 (0.0%)<br>• phylum: 15 (1.199%)<br>• superkingdom: 6 (0.479%)<br>• root: 290 (23.181%)	• Methanothermococcus okinawensis [taxid 155863]: 2 (0.159%)<br>• Methanocaldococcus bathoardescens [taxid 1301915]: 1 (0.079%)<br>• Methanotorris igneus [taxid 2189]: 1 (0.079%)
Benchmark OTU ID: `CP002913-_Euryarchaeota`<br>OTU taxon: Methanococcus maripaludis X1 [taxid 1053692]<br>Expected: Methanococcus maripaludis [taxid 39152] (species)<br>Number of reads: 1437<br>Number of identified reads: 1410 (98.121%)	• **species: 1010 (70.285%)**<br>• genus: 49 (3.409%)<br>• family: 4 (0.278%)<br>• order: 14 (0.974%)<br>• class: 0 (0.0%)<br>• phylum: 11 (0.765%)<br>• superkingdom: 7 (0.487%)<br>• root: 304 (21.155%)	• Methanococcus aeolicus [taxid 42879]: 1 (0.069%)<br>• Methanocella arvoryzae [taxid 1175445]: 1 (0.069%)<br>• Methanococcus vannielii [taxid 2187]: 1 (0.069%)<br>• Latimeria chalumnae [taxid 7897]: 1 (0.069%)<br>• Methanocaldococcus bathoardescens [taxid 1301915]: 1 (0.069%)
Benchmark OTU ID: `CP000559-_Euryarchaeota`<br>OTU taxon: Methanocorpusculum labreanum Z [taxid 410358]<br>Expected: Methanocorpusculum labreanum [taxid 83984] (species)<br>Number of reads: 1498<br>Number of identified reads: 1491 (99.532%)	• **species: 656 (43.791%)**<br>• genus: 205 (13.684%)<br>• family: 0 (0.0%)<br>• order: 11 (0.734%)<br>• class: 6 (0.4%)<br>• phylum: 28 (1.869%)<br>• superkingdom: 19 (1.268%)<br>• root: 506 (33.778%)	• Methanocorpusculum sp. MCE [taxid 2006183]: 14 (0.934%)<br>• Methanocorpusculum parvum [taxid 2193]: 3 (0.2%)<br>• Methanocaldococcus bathoardescens [taxid 1301915]: 1 (0.066%)<br>• Insolitispirillum peregrinum [taxid 80876]: 1 (0.066%)<br>• Bacillus sp. SJS [taxid 1423321]: 1 (0.066%)<br>• Acetonema longum [taxid 2374]: 1 (0.066%)<br>• Rothia mucilaginosa [taxid 43675]: 1 (0.066%)<br>• Glaciihabitans arcticus [taxid 2668039]: 1 (0.066%)<br>• other: 2 (0.133%)

Operational Taxonomic Unit (OTU)	Correct identifications	Wrong or overspecific identifications at species rank
Benchmark OTU ID: `CP000780-_Euryarchaeota`<br>OTU taxon: Methanoregula boonei 6A8 [taxid 456442]<br>Expected: Methanoregula boonei [taxid 358766] (species)<br>Number of reads: 2273<br>Number of identified reads: 2268 (99.78%)	• **species: 1392 (61.24%)**<br>• genus: 21 (0.923%)<br>• family: 15 (0.659%)<br>• order: 52 (2.287%)<br>• class: 12 (0.527%)<br>• phylum: 15 (0.659%)<br>• superkingdom: 17 (0.747%)<br>• root: 701 (30.84%)	• Gilliamella apicola [taxid 1196095]: 1 (0.043%)<br>• Prunus armeniaca [taxid 36596]: 1 (0.043%)<br>• Fontibacillus phaseoli [taxid 1416533]: 1 (0.043%)<br>• Candidatus Woesearchaeota archaeon [taxid 2026803]: 1 (0.043%)<br>• Thermoplasmata archaeon [taxid 1906666]: 1 (0.043%)<br>• Methanoculleus thermophilus [taxid 2200]: 1 (0.043%)<br>• Archaeoglobi archaeon [taxid 2250530]: 1 (0.043%)<br>• Escherichia coli [taxid 562]: 1 (0.043%)<br>• other: 4 (0.175%)
Benchmark OTU ID: `CP003117-_Euryarchaeota`<br>OTU taxon: Methanosaeta harundinacea 6Ac [taxid 1110509]<br>Expected: Methanosaeta harundinacea [taxid 301375] (species)<br>Number of reads: 2290<br>Number of identified reads: 2265 (98.908%)	• **species: 1424 (62.183%)**<br>• genus: 53 (2.314%)<br>• family: 0 (0.0%)<br>• order: 11 (0.48%)<br>• class: 8 (0.349%)<br>• phylum: 17 (0.742%)<br>• superkingdom: 11 (0.48%)<br>• root: 676 (29.519%)	• Schizosaccharomyces japonicus [taxid 4897]: 1 (0.043%)<br>• Tetrapisispora phaffii [taxid 113608]: 1 (0.043%)<br>• Methanothrix thermoacetophila [taxid 2224]: 1 (0.043%)
Benchmark OTU ID: `CP002101-_Euryarchaeota`<br>OTU taxon: Methanosalsum zhilinae DSM 4017 [taxid 679901]<br>Expected: Methanosalsum zhilinae [taxid 39669] (species)<br>Number of reads: 1848<br>Number of identified reads: 1827 (98.863%)	• **species: 1175 (63.582%)**<br>• genus: 22 (1.19%)<br>• family: 63 (3.409%)<br>• order: 13 (0.703%)<br>• class: 3 (0.162%)<br>• phylum: 14 (0.757%)<br>• superkingdom: 4 (0.216%)<br>• root: 505 (27.326%)	• Coprococcus comes [taxid 410072]: 1 (0.054%)<br>• Pseudodesulfovibrio profundus [taxid 57320]: 1 (0.054%)<br>• Branchiostoma floridae [taxid 7739]: 1 (0.054%)<br>• Methanolobus profundi [taxid 487685]: 1 (0.054%)<br>• Methanococcoides vulcani [taxid 1353158]: 1 (0.054%)
Benchmark OTU ID: `AE010299-_Euryarchaeota`<br>OTU taxon: Methanosarcina acetivorans C2A [taxid 188937]<br>Expected: Methanosarcina acetivorans [taxid 2214] (species)<br>Number of reads: 5642<br>Number of identified reads: 5533 (98.068%)	• **species: 2123 (37.628%)**<br>• genus: 1462 (25.912%)<br>• family: 53 (0.939%)<br>• order: 9 (0.159%)<br>• class: 15 (0.265%)<br>• phylum: 18 (0.319%)<br>• superkingdom: 14 (0.248%)<br>• root: 1749 (30.999%)	• Methanosarcina siciliae [taxid 38027]: 39 (0.691%)<br>• Methanosarcina mazei [taxid 2209]: 11 (0.194%)<br>• Methanosarcina horonobensis [taxid 418008]: 7 (0.124%)<br>• Methanosarcina barkeri [taxid 2208]: 6 (0.106%)<br>• Methanocaldococcus bathoardescens [taxid 1301915]: 1 (0.017%)<br>• Methanohalophilus halophilus [taxid 2177]: 1 (0.017%)<br>• Methanosarcina spelaei [taxid 1036679]: 1 (0.017%)<br>• Clostridium botulinum [taxid 1491]: 1 (0.017%)<br>• other: 14 (0.248%)

Operational Taxonomic Unit (OTU)	Correct identifications	Wrong or overspecific identifications at species rank
Benchmark OTU ID: `CP000099-_Euryarchaeota`<br>OTU taxon: Methanosarcina barkeri str. Fusaro [taxid 269797]<br>Expected: Methanosarcina barkeri [taxid 2208] (species)<br>Number of reads: 4682<br>Number of identified reads: 4562 (97.436%)	• **species: 1685 (35.988%)**<br>• genus: 1281 (27.36%)<br>• family: 21 (0.448%)<br>• order: 15 (0.32%)<br>• class: 3 (0.064%)<br>• phylum: 17 (0.363%)<br>• superkingdom: 12 (0.256%)<br>• root: 1477 (31.546%)	• Methanosarcina flavescens [taxid 1715806]: 4 (0.085%)<br>• Methanosarcina lacustris [taxid 170861]: 4 (0.085%)<br>• Methanosarcina acetivorans [taxid 2214]: 4 (0.085%)<br>• Methanosarcina mazei [taxid 2209]: 4 (0.085%)<br>• Methanosarcina spelaei [taxid 1036679]: 3 (0.064%)<br>• Methanosarcina thermophila [taxid 2210]: 2 (0.042%)<br>• Methanosarcina vacuolata [taxid 2215]: 1 (0.021%)<br>• Methanosarcina siciliae [taxid 38027]: 1 (0.021%)<br>• other: 13 (0.277%)
Benchmark OTU ID: `CP000254-_Euryarchaeota`<br>OTU taxon: Methanospirillum hungatei JF-1 [taxid 323259]<br>Expected: Methanospirillum hungatei [taxid 2203] (species)<br>Number of reads: 3325<br>Number of identified reads: 3305 (99.398%)	• species: 1126 (33.864%)<br>• genus: 41 (1.233%)<br>• family: 0 (0.0%)<br>• order: 25 (0.751%)<br>• class: 16 (0.481%)<br>• **phylum: 1205 (36.24%)**<br>• superkingdom: 8 (0.24%)<br>• root: 843 (25.353%)	• Methanospirillum stamsii [taxid 1277351]: 4 (0.12%)<br>• Methanolacinia petrolearia [taxid 54120]: 2 (0.06%)<br>• Methanospirillum lacunae [taxid 668570]: 2 (0.06%)<br>• Hadesarchaea archaeon [taxid 2250276]: 1 (0.03%)<br>• Bacillus oleivorans [taxid 1448271]: 1 (0.03%)<br>• Paenibacillus borealis [taxid 160799]: 1 (0.03%)<br>• Peptoanaerobacter stomatis [taxid 796937]: 1 (0.03%)<br>• Euryarchaeota archaeon [taxid 2026739]: 1 (0.03%)<br>• other: 4 (0.12%)
Benchmark OTU ID: `CP001710-_Euryarchaeota`<br>OTU taxon: Methanothermobacter marburgensis str. Marburg [taxid 79929]<br>Expected: Methanothermobacter marburgensis [taxid 145263] (species)<br>Number of reads: 1319<br>Number of identified reads: 1311 (99.393%)	• species: 170 (12.888%)<br>• **genus: 631 (47.839%)**<br>• family: 28 (2.122%)<br>• order: 9 (0.682%)<br>• class: 0 (0.0%)<br>• phylum: 14 (1.061%)<br>• superkingdom: 8 (0.606%)<br>• root: 424 (32.145%)	• Neotoma lepida [taxid 56216]: 1 (0.075%)<br>• Pseudobacter ginsenosidimutans [taxid 661488]: 1 (0.075%)<br>• Undibacterium parvum [taxid 401471]: 1 (0.075%)<br>• Methanothermobacter thermautotrophicus [taxid 145262]: 1 (0.075%)
Benchmark OTU ID: `AE000666-_Euryarchaeota`<br>OTU taxon: Methanothermobacter thermautotrophicus str. Delta H [taxid 187420]<br>Expected: Methanothermobacter thermautotrophicus [taxid 145262] (species)<br>Number of reads: 1433<br>Number of identified reads: 1425 (99.441%)	• species: 144 (10.048%)<br>• **genus: 708 (49.406%)**<br>• family: 45 (3.14%)<br>• order: 11 (0.767%)<br>• class: 0 (0.0%)<br>• phylum: 6 (0.418%)<br>• superkingdom: 7 (0.488%)<br>• root: 479 (33.426%)	• Methanothermobacter defluvii [taxid 49339]: 3 (0.209%)<br>• Psimunavirus psiM2 [taxid 2734265]: 1 (0.069%)<br>• Pseudobacter ginsenosidimutans [taxid 661488]: 1 (0.069%)<br>• Pelosinus fermentans [taxid 365349]: 1 (0.069%)<br>• Methanothermobacter marburgensis [taxid 145263]: 1 (0.069%)<br>• Corynebacterium pilosum [taxid 35756]: 1 (0.069%)
Benchmark OTU ID: `CP002278-_Euryarchaeota`<br>OTU taxon: Methanothermus fervidus DSM 2088 [taxid 523846]<br>Expected: Methanothermus fervidus [taxid 2180] (species)<br>Number of reads: 908<br>Number of identified reads: 903 (99.449%)	• **species: 633 (69.713%)**<br>• genus: 1 (0.11%)<br>• family: 0 (0.0%)<br>• order: 31 (3.414%)<br>• class: 0 (0.0%)<br>• phylum: 14 (1.541%)<br>• superkingdom: 14 (1.541%)<br>• root: 206 (22.687%)	• Achlya hypogyna [taxid 1202772]: 1 (0.11%)

Operational Taxonomic Unit (OTU)	Correct identifications	Wrong or overspecific identifications at species rank
Benchmark OTU ID: `CP000975-_Verrucomicrobia`<br>OTU taxon: Methylacidiphilum infernorum V4 [taxid 481448]<br>Expected: Methylacidiphilum infernorum [taxid 511746] (species)<br>Number of reads: 148963<br>Number of identified reads: 147843 (99.248%)	• **species: 87870 (58.987%)**<br>• genus: 18507 (12.423%)<br>• family: 0 (0.0%)<br>• order: 0 (0.0%)<br>• class: 0 (0.0%)<br>• phylum: 1193 (0.8%)<br>• superkingdom: 11037 (7.409%)<br>• root: 29017 (19.479%)	• Methylacidiphilum sp. Phi [taxid 1847729]: 224 (0.15%)<br>• Methylacidiphilum kamchatkense [taxid 431057]: 27 (0.018%)<br>• Methylacidiphilum sp. Yel [taxid 1847730]: 17 (0.011%)<br>• Methylacidiphilum fumariolicum [taxid 591154]: 15 (0.01%)<br>• Peribacillus simplex [taxid 1478]: 5 (0.003%)<br>• Chloroflexus aggregans [taxid 152260]: 5 (0.003%)<br>• Oikopleura dioica [taxid 34765]: 4 (0.002%)<br>• bacterium [taxid 1869227]: 4 (0.002%)<br>• other: 261 (0.175%)
Benchmark OTU ID: `CP000555-_Proteobacteria`<br>OTU taxon: Methylibium petroleiphilum PM1 [taxid 420662]<br>Expected: Methylibium petroleiphilum [taxid 105560] (species)<br>Number of reads: 8215<br>Number of identified reads: 8191 (99.707%)	• species: 1172 (14.266%)<br>• **genus: 4134 (50.322%)**<br>• order: 1095 (13.329%)<br>• class: 227 (2.763%)<br>• phylum: 659 (8.021%)<br>• superkingdom: 381 (4.637%)<br>• root: 521 (6.342%)	• Methylibium sp. Pch-M [taxid 2082386]: 78 (0.949%)<br>• Piscinibacter aquaticus [taxid 392597]: 2 (0.024%)<br>• Aquabacterium pictum [taxid 2315236]: 2 (0.024%)<br>• Pseudomonas stutzeri [taxid 316]: 2 (0.024%)<br>• Ideonella dechloratans [taxid 36863]: 2 (0.024%)<br>• Saezia sanguinis [taxid 1965230]: 1 (0.012%)<br>• Chitinimonas arctica [taxid 2594795]: 1 (0.012%)<br>• other: 31 (0.377%)
Benchmark OTU ID: `CP001001-_Proteobacteria`<br>OTU taxon: Methylobacterium radiotolerans JCM 2831 [taxid 426355]<br>Expected: Methylobacterium radiotolerans [taxid 31998] (species)<br>Number of reads: 12791<br>Number of identified reads: 12722 (99.46%)	• species: 3272 (25.58%)<br>• **genus: 5621 (43.944%)**<br>• family: 467 (3.651%)<br>• order: 429 (3.353%)<br>• class: 448 (3.502%)<br>• phylum: 493 (3.854%)<br>• superkingdom: 764 (5.972%)<br>• root: 1203 (9.405%)	• Methylobacterium oryzae [taxid 334852]: 17 (0.132%)<br>• Methylobacterium phyllostachyos [taxid 582672]: 9 (0.07%)<br>• Methylobacterium mesophilicum [taxid 39956]: 9 (0.07%)<br>• Methylobacterium symbioticum [taxid 2584084]: 7 (0.054%)<br>• Lupinus albus [taxid 3870]: 7 (0.054%)<br>• Methylobacterium pseudosasicola [taxid 582667]: 5 (0.039%)<br>• Methylobacterium brachiatum [taxid 269660]: 5 (0.039%)<br>• Methylobacterium oxalidis [taxid 944322]: 3 (0.023%)<br>• other: 58 (0.453%)
Benchmark OTU ID: `CP000943-_Proteobacteria`<br>OTU taxon: Methylobacterium sp. 4-46 [taxid 426117]<br>Expected: Methylobacterium [taxid 407] (genus)<br>Number of reads: 16349<br>Number of identified reads: 16230 (99.272%)	• **genus: 10666 (65.239%)**<br>• family: 321 (1.963%)<br>• order: 756 (4.624%)<br>• class: 629 (3.847%)<br>• phylum: 692 (4.232%)<br>• superkingdom: 1115 (6.819%)<br>• root: 2024 (12.379%)	• Methylobacterium nodulans [taxid 114616]: 33 (0.201%)<br>• Methylobacterium aquaticum [taxid 270351]: 6 (0.036%)<br>• Methylobacterium tarhaniae [taxid 1187852]: 6 (0.036%)<br>• Methylobacterium sp. CLZ [taxid 2696060]: 5 (0.03%)<br>• Methylobacterium gnaphalii [taxid 1010610]: 4 (0.024%)<br>• Methylobacterium dankookense [taxid 560405]: 4 (0.024%)<br>• Methylobacterium currus [taxid 2051553]: 4 (0.024%)<br>• other: 98 (0.599%)

Operational Taxonomic Unit (OTU)	Correct identifications	Wrong or overspecific identifications at species rank
Benchmark OTU ID: `AE017282-_Proteobacteria`<br>OTU taxon: Methylococcus capsulatus str. Bath [taxid 243233]<br>Expected: Methylococcus capsulatus [taxid 414] (species)<br>Number of reads: 6551<br>Number of identified reads: 6536 (99.771%)	• **species: 4403 (67.211%)**<br>• genus: 6 (0.091%)<br>• family: 91 (1.389%)<br>• order: 22 (0.335%)<br>• class: 295 (4.503%)<br>• phylum: 544 (8.304%)<br>• superkingdom: 405 (6.182%)<br>• root: 764 (11.662%)	• Tepidicella xavieri [taxid 360241]: 3 (0.045%)<br>• Brugia timori [taxid 42155]: 1 (0.015%)<br>• Limisphaera ngatamarikiensis [taxid 1324935]: 1 (0.015%)<br>• Tetrabaena socialis [taxid 47790]: 1 (0.015%)<br>• Ramlibacter tataouinensis [taxid 94132]: 1 (0.015%)<br>• Cellulosimicrobium cellulans [taxid 1710]: 1 (0.015%)<br>• Tepidimonas fonticaldi [taxid 1101373]: 1 (0.015%)<br>• Vibrio crassostreae [taxid 246167]: 1 (0.015%)<br>• other: 20 (0.305%)
Benchmark OTU ID: `HE956757-_Proteobacteria`<br>OTU taxon: Methylocystis sp. SC2 [taxid 187303]<br>Expected: Methylocystis [taxid 133] (genus)<br>Number of reads: 7606<br>Number of identified reads: 7570 (99.526%)	• **genus: 3822 (50.249%)**<br>• family: 1379 (18.13%)<br>• order: 489 (6.429%)<br>• class: 287 (3.773%)<br>• phylum: 225 (2.958%)<br>• superkingdom: 412 (5.416%)<br>• root: 947 (12.45%)	• Methylocystis hirsuta [taxid 369798]: 24 (0.315%)<br>• Methylocystis rosea [taxid 173366]: 12 (0.157%)<br>• Methylocystis heyeri [taxid 391905]: 4 (0.052%)<br>• Methylocystis parvus [taxid 134]: 4 (0.052%)<br>• Methylocystis bryophila [taxid 655015]: 3 (0.039%)<br>• Salmonella enterica [taxid 28901]: 2 (0.026%)<br>• Tannerella forsythia [taxid 28112]: 1 (0.013%)<br>• other: 25 (0.328%)
Benchmark OTU ID: `FO082060-_Proteobacteria`<br>OTU taxon: Methylomicrobium alcaliphilum 20Z [taxid 1091494]<br>Expected: Methylomicrobium alcaliphilum [taxid 271065] (species)<br>Number of reads: 9619<br>Number of identified reads: 9547 (99.251%)	• species: 2525 (26.25%)<br>• genus: 1844 (19.17%)<br>• family: 321 (3.337%)<br>• order: 67 (0.696%)<br>• **class: 2541 (26.416%)**<br>• phylum: 357 (3.711%)<br>• superkingdom: 398 (4.137%)<br>• root: 1485 (15.438%)	• Methylomicrobium buryatense [taxid 95641]: 57 (0.592%)<br>• Methylomicrobium kenyense [taxid 269709]: 40 (0.415%)<br>• Methylocaldum marinum [taxid 1432792]: 2 (0.02%)<br>• Candidatus Kentron sp. LFY [taxid 2126342]: 2 (0.02%)<br>• Salmonella enterica [taxid 28901]: 2 (0.02%)<br>• Methylomonas methanica [taxid 421]: 2 (0.02%)<br>• Thalassotalea euphylliae [taxid 1655234]: 1 (0.01%)<br>• Bauldia litoralis [taxid 665467]: 1 (0.01%)<br>• other: 34 (0.353%)
Benchmark OTU ID: `CP003380-_Proteobacteria`<br>OTU taxon: Methylophaga frappieri [taxid 754477]<br>Expected: Methylophaga frappieri [taxid 754477] (species)<br>Number of reads: 5185<br>Number of identified reads: 5176 (99.826%)	• **species: 3436 (66.268%)**<br>• genus: 93 (1.793%)<br>• family: 4 (0.077%)<br>• order: 7 (0.135%)<br>• class: 581 (11.205%)<br>• phylum: 309 (5.959%)<br>• superkingdom: 201 (3.876%)<br>• root: 542 (10.453%)	• Aliiglaciecola lipolytica [taxid 477689]: 4 (0.077%)<br>• Pseudomonas stutzeri [taxid 316]: 2 (0.038%)<br>• Vibrio cholerae [taxid 666]: 1 (0.019%)<br>• Salinisphaera japonica [taxid 1304270]: 1 (0.019%)<br>• Edwardsiella tarda [taxid 636]: 1 (0.019%)<br>• Schizaphis graminum [taxid 13262]: 1 (0.019%)<br>• Cupriavidus metallidurans [taxid 119219]: 1 (0.019%)<br>• Novimethylophilus kurashikiensis [taxid 1825523]: 1 (0.019%)<br>• other: 15 (0.289%)

Operational Taxonomic Unit (OTU)	Correct identifications	Wrong or overspecific identifications at species rank
Benchmark OTU ID: `CP001511-_Proteobacteria`<br>OTU taxon: Methylorubrum extorquens AM1 [taxid 272630]<br>Expected: Methylorubrum extorquens [taxid 408] (species)<br>Number of reads: 1954<br>Number of identified reads: 1938 (99.181%)	• **species: 631 (32.292%)**<br>• genus: 584 (29.887%)<br>• family: 235 (12.026%)<br>• order: 26 (1.33%)<br>• class: 58 (2.968%)<br>• phylum: 35 (1.791%)<br>• superkingdom: 81 (4.145%)<br>• root: 285 (14.585%)	• Methylorubrum populi [taxid 223967]: 7 (0.358%)<br>• Methylobacterium aquaticum [taxid 270351]: 2 (0.102%)<br>• Rhodobacter ovatus [taxid 439529]: 1 (0.051%)<br>• Hartmannibacter diazotrophicus [taxid 1482074]: 1 (0.051%)<br>• Kozakia baliensis [taxid 153496]: 1 (0.051%)<br>• Selaginella moellendorffii [taxid 88036]: 1 (0.051%)<br>• Isosphaera pallida [taxid 128]: 1 (0.051%)<br>• Ruegeria lacuscaerulensis [taxid 55218]: 1 (0.051%)<br>• other: 8 (0.409%)
Benchmark OTU ID: `CP001298-_Proteobacteria`<br>OTU taxon: Methylorubrum extorquens CM4 [taxid 440085]<br>Expected: Methylorubrum extorquens [taxid 408] (species)<br>Number of reads: 12116<br>Number of identified reads: 12033 (99.314%)	• species: 2168 (17.893%)<br>• genus: 1268 (10.465%)<br>• **family: 5606 (46.269%)**<br>• order: 359 (2.963%)<br>• class: 329 (2.715%)<br>• phylum: 358 (2.954%)<br>• superkingdom: 572 (4.721%)<br>• root: 1359 (11.216%)	• Methylorubrum populi [taxid 223967]: 35 (0.288%)<br>• Methylobacterium sp. CLZ [taxid 2696060]: 8 (0.066%)<br>• Methylobacterium haplocladii [taxid 1176176]: 5 (0.041%)<br>• Lupinus albus [taxid 3870]: 5 (0.041%)<br>• Methylobacterium durans [taxid 2202825]: 4 (0.033%)<br>• Methylobacterium pseudosasicola [taxid 582667]: 3 (0.024%)<br>• Methylobacterium gossipiicola [taxid 582675]: 3 (0.024%)<br>• Methylobacterium aquaticum [taxid 270351]: 2 (0.016%)<br>• other: 37 (0.305%)
Benchmark OTU ID: `CP001029-_Proteobacteria`<br>OTU taxon: Methylorubrum populi BJ001 [taxid 441620]<br>Expected: Methylorubrum populi [taxid 223967] (species)<br>Number of reads: 12167<br>Number of identified reads: 12069 (99.194%)	• species: 4243 (34.873%)<br>• genus: 251 (2.062%)<br>• **family: 4506 (37.034%)**<br>• order: 423 (3.476%)<br>• class: 481 (3.953%)<br>• phylum: 395 (3.246%)<br>• superkingdom: 593 (4.873%)<br>• root: 1158 (9.517%)	• Methylorubrum extorquens [taxid 408]: 33 (0.271%)<br>• Methylorubrum salsuginis [taxid 414703]: 7 (0.057%)<br>• Methylobacterium sp. CLZ [taxid 2696060]: 7 (0.057%)<br>• Methylobacterium soli [taxid 553447]: 5 (0.041%)<br>• Methylobacterium gnaphalii [taxid 1010610]: 5 (0.041%)<br>• Methylobacterium durans [taxid 2202825]: 4 (0.032%)<br>• Lupinus albus [taxid 3870]: 4 (0.032%)<br>• Methylobacterium platani [taxid 427683]: 2 (0.016%)<br>• other: 52 (0.427%)
Benchmark OTU ID: `CP001672-_Proteobacteria`<br>OTU taxon: Methylotenera mobilis JLW8 [taxid 583345]<br>Expected: Methylotenera mobilis [taxid 359408] (species)<br>Number of reads: 4848<br>Number of identified reads: 4813 (99.278%)	• **species: 2184 (45.049%)**<br>• genus: 853 (17.594%)<br>• family: 82 (1.691%)<br>• order: 117 (2.413%)<br>• class: 480 (9.9%)<br>• phylum: 475 (9.797%)<br>• superkingdom: 187 (3.857%)<br>• root: 432 (8.91%)	• Novimethylophilus kurashikiensis [taxid 1825523]: 2 (0.041%)<br>• Shimia haliotis [taxid 1280847]: 1 (0.02%)<br>• Escherichia coli [taxid 562]: 1 (0.02%)<br>• Methylotenera versatilis [taxid 1055487]: 1 (0.02%)<br>• Castellaniella defragrans [taxid 75697]: 1 (0.02%)<br>• Marinimicrobium koreense [taxid 306545]: 1 (0.02%)<br>• Lupinus albus [taxid 3870]: 1 (0.02%)<br>• Candidatus Methylopumilus turicensis [taxid 1581680]: 1 (0.02%)<br>• other: 16 (0.33%)

Operational Taxonomic Unit (OTU)	Correct identifications	Wrong or overspecific identifications at species rank
Benchmark OTU ID: `CP001674-_Proteobacteria`<br>OTU taxon: Methylovorus glucosetrophus SIP3-4 [taxid 582744]<br>Expected: Methylovorus glucosotrophus [taxid 266009] (species)<br>Number of reads: 5856<br>Number of identified reads: 5846 (99.829%)	• **species: 3872 (66.12%)**<br>• genus: 50 (0.853%)<br>• family: 129 (2.202%)<br>• order: 39 (0.665%)<br>• class: 373 (6.369%)<br>• phylum: 606 (10.348%)<br>• superkingdom: 222 (3.79%)<br>• root: 553 (9.443%)	• Novimethylophilus kurashikiensis [taxid 1825523]: 2 (0.034%)<br>• Methylocaldum marinum [taxid 1432792]: 1 (0.017%)<br>• Pragia fontium [taxid 82985]: 1 (0.017%)<br>• Komagataeibacter nataicola [taxid 265960]: 1 (0.017%)<br>• Zobellella endophytica [taxid 2116700]: 1 (0.017%)<br>• Adlercreutzia caecimuris [taxid 671266]: 1 (0.017%)<br>• Methylotenera versatilis [taxid 1055487]: 1 (0.017%)<br>• Paramesorhizobium deserti [taxid 1494590]: 1 (0.017%)<br>• other: 14 (0.239%)
Benchmark OTU ID: `CP002252-_Proteobacteria`<br>OTU taxon: Methylovorus sp. MP688 [taxid 887061]<br>Expected: Methylovorus [taxid 81682] (genus)<br>Number of reads: 5556<br>Number of identified reads: 5549 (99.874%)	• **genus: 3129 (56.317%)**<br>• family: 189 (3.401%)<br>• order: 55 (0.989%)<br>• class: 422 (7.595%)<br>• phylum: 600 (10.799%)<br>• superkingdom: 283 (5.093%)<br>• root: 866 (15.586%)	• **Methylovorus glucosotrophus [taxid 266009]: 3087 (55.561%)**<br>• Pseudomethylobacillus aquaticus [taxid 2676064]: 4 (0.071%)<br>• Candidatus Methylopumilus turicensis [taxid 1581680]: 2 (0.035%)<br>• Methylobacillus flagellatus [taxid 405]: 2 (0.035%)<br>• Methylotenera versatilis [taxid 1055487]: 2 (0.035%)<br>• Candidatus Thiodictyon syntrophicum [taxid 1166950]: 1 (0.017%)<br>• Pirellulimonas nuda [taxid 2528009]: 1 (0.017%)<br>• other: 23 (0.413%)
Benchmark OTU ID: `CP002382-_Proteobacteria`<br>OTU taxon: Micavibrio aeruginosavorus ARL-13 [taxid 856793]<br>Expected: Micavibrio aeruginosavorus [taxid 349221] (species)<br>Number of reads: 4700<br>Number of identified reads: 4690 (99.787%)	• **species: 3650 (77.659%)**<br>• genus: 10 (0.212%)<br>• class: 235 (5.0%)<br>• phylum: 126 (2.68%)<br>• superkingdom: 152 (3.234%)<br>• root: 511 (10.872%)	• labyrinthulid quahog parasite QPX [taxid 96639]: 1 (0.021%)<br>• Lupinus albus [taxid 3870]: 1 (0.021%)<br>• Bradyrhizobium lablabi [taxid 722472]: 1 (0.021%)<br>• Erythrobacter aureus [taxid 2182384]: 1 (0.021%)<br>• Melanaphis sacchari [taxid 742174]: 1 (0.021%)<br>• Salmonella enterica [taxid 28901]: 1 (0.021%)
Benchmark OTU ID: `CP003630-_Cyanobacteria`<br>OTU taxon: Microcoleus sp. PCC 7113 [taxid 1173027]<br>Expected: Microcoleus [taxid 44471] (genus)<br>Number of reads: 45445<br>Number of identified reads: 45024 (99.073%)	• **genus: 28678 (63.104%)**<br>• family: 233 (0.512%)<br>• order: 722 (1.588%)<br>• phylum: 5557 (12.227%)<br>• superkingdom: 2559 (5.63%)<br>• root: 7189 (15.819%)	• Coleofasciculus chthonoplastes [taxid 64178]: 21 (0.046%)<br>• Crinalium epipsammum [taxid 241425]: 7 (0.015%)<br>• Crocosphaera watsonii [taxid 263511]: 4 (0.008%)<br>• Neosynechococcus sphagnicola [taxid 1501145]: 4 (0.008%)<br>• Nostoc sphaeroides [taxid 446679]: 4 (0.008%)<br>• Roseofilum reptotaenium [taxid 1233427]: 3 (0.006%)<br>• other: 129 (0.283%)
Benchmark OTU ID: `AP009552-_Cyanobacteria`<br>OTU taxon: Microcystis aeruginosa NIES-843 [taxid 449447]<br>Expected: Microcystis aeruginosa [taxid 1126] (species)<br>Number of reads: 34702<br>Number of identified reads: 34135 (98.366%)	• species: 3970 (11.44%)<br>• **genus: 23077 (66.5%)**<br>• family: 0 (0.0%)<br>• order: 396 (1.141%)<br>• phylum: 1569 (4.521%)<br>• superkingdom: 1409 (4.06%)<br>• root: 3669 (10.572%)	• Microcystis viridis [taxid 44822]: 159 (0.458%)<br>• Microcystis panniformis [taxid 513223]: 102 (0.293%)<br>• Microcystis flos-aquae [taxid 109615]: 57 (0.164%)<br>• Microcystis wesenbergii [taxid 44823]: 24 (0.069%)<br>• Microcystis novacekii [taxid 75560]: 19 (0.054%)<br>• Aphanothece hegewaldii [taxid 1521625]: 2 (0.005%)<br>• Candidatus Atelocyanobacterium thalassa [taxid 713887]: 2 (0.005%)<br>• other: 52 (0.149%)

Operational Taxonomic Unit (OTU)	Correct identifications	Wrong or overspecific identifications at species rank
Benchmark OTU ID: `AP012204-_Actinobacteria`<br>OTU taxon: Microlunatus phosphovorus NM-1 [taxid 1032480]<br>Expected: Microlunatus phosphovorus [taxid 29405] (species)<br>Number of reads: 25444<br>Number of identified reads: 25303 (99.445%)	• **species: 17373 (68.279%)**<br>• genus: 82 (0.322%)<br>• family: 175 (0.687%)<br>• order: 300 (1.179%)<br>• class: 2690 (10.572%)<br>• phylum: 38 (0.149%)<br>• superkingdom: 1628 (6.398%)<br>• root: 2997 (11.778%)	• Mycobacteroides abscessus [taxid 36809]: 2 (0.007%)<br>• Actinopolymorpha cephalotaxi [taxid 504797]: 2 (0.007%)<br>• Brevibacterium casei [taxid 33889]: 2 (0.007%)<br>• Intrasporangium calvum [taxid 53358]: 1 (0.003%)<br>• Pseudopedobacter saltans [taxid 151895]: 1 (0.003%)<br>• Mycolicibacterium thermoresistibile [taxid 1797]: 1 (0.003%)<br>• Streptomyces gilvosporeus [taxid 553510]: 1 (0.003%)<br>• Azotobacter beijerinckii [taxid 170623]: 1 (0.003%)<br>• other: 72 (0.282%)
Benchmark OTU ID: `CP002638-_Actinobacteria`<br>OTU taxon: Micromonospora maris AB-18-032 [taxid 263358]<br>Expected: Micromonospora maris [taxid 1003110] (species)<br>Number of reads: 30299<br>Number of identified reads: 30184 (99.62%)	• species: 2282 (7.531%)<br>• genus: 2561 (8.452%)<br>• **family: 18423 (60.803%)**<br>• order: 0 (0.0%)<br>• class: 2582 (8.521%)<br>• phylum: 25 (0.082%)<br>• superkingdom: 1673 (5.521%)<br>• root: 2614 (8.627%)	• Micromonospora zingiberis [taxid 2053011]: 27 (0.089%)<br>• Micromonospora radicis [taxid 1894971]: 17 (0.056%)<br>• Micromonospora craterilacus [taxid 1655439]: 15 (0.049%)<br>• Micromonospora globbae [taxid 1894969]: 14 (0.046%)<br>• Micromonospora phaseoli [taxid 1144548]: 11 (0.036%)<br>• Micromonospora olivasterospora [taxid 1880]: 9 (0.029%)<br>• Micromonospora rosaria [taxid 47874]: 8 (0.026%)<br>• Micromonospora sediminimaris [taxid 547162]: 8 (0.026%)<br>• other: 287 (0.947%)
Benchmark OTU ID: `CP001992-_Actinobacteria`<br>OTU taxon: Mobiluncus curtisii ATCC 43063 [taxid 548479]<br>Expected: Mobiluncus curtisii [taxid 2051] (species)<br>Number of reads: 8114<br>Number of identified reads: 8073 (99.494%)	• species: 1465 (18.055%)<br>• **genus: 4478 (55.188%)**<br>• family: 143 (1.762%)<br>• order: 0 (0.0%)<br>• class: 444 (5.472%)<br>• phylum: 36 (0.443%)<br>• superkingdom: 506 (6.236%)<br>• root: 993 (12.238%)	• Mobiluncus mulieris [taxid 2052]: 9 (0.11%)<br>• Mobiluncus holmesii [taxid 144178]: 7 (0.086%)<br>• Varibaculum cambriense [taxid 184870]: 3 (0.036%)<br>• Pseudopropionibacterium propionicum [taxid 1750]: 1 (0.012%)<br>• Gardnerella vaginalis [taxid 2702]: 1 (0.012%)<br>• Mobilicoccus pelagius [taxid 746032]: 1 (0.012%)<br>• Vibrio gigantis [taxid 296199]: 1 (0.012%)<br>• Flavonifractor plautii [taxid 292800]: 1 (0.012%)<br>• other: 17 (0.209%)
Benchmark OTU ID: `FO203431-_Actinobacteria`<br>OTU taxon: Modestobacter marinus [taxid 477641]<br>Expected: Modestobacter marinus [taxid 477641] (species)<br>Number of reads: 24917<br>Number of identified reads: 24819 (99.606%)	• **species: 12966 (52.036%)**<br>• genus: 1773 (7.115%)<br>• family: 2894 (11.614%)<br>• order: 0 (0.0%)<br>• class: 3273 (13.135%)<br>• phylum: 33 (0.132%)<br>• superkingdom: 1833 (7.356%)<br>• root: 2026 (8.13%)	• Modestobacter versicolor [taxid 429133]: 18 (0.072%)<br>• Modestobacter muralis [taxid 1608614]: 13 (0.052%)<br>• Modestobacter roseus [taxid 1181884]: 12 (0.048%)<br>• Blastococcus saxobsidens [taxid 138336]: 9 (0.036%)<br>• Modestobacter multiseptatus [taxid 88139]: 8 (0.032%)<br>• Modestobacter caceresii [taxid 1522368]: 8 (0.032%)<br>• Geodermatophilus obscurus [taxid 1861]: 7 (0.028%)<br>• Geodermatophilus sabuli [taxid 1564158]: 3 (0.012%)<br>• other: 102 (0.409%)

Operational Taxonomic Unit (OTU)	Correct identifications	Wrong or overspecific identifications at species rank
Benchmark OTU ID: `CP004345-_Proteobacteria`<br>OTU taxon: Morganella morganii subsp. morganii KT [taxid 1124991]<br>Expected: Morganella morganii [taxid 582] (species)<br>Number of reads: 7663<br>Number of identified reads: 7639 (99.686%)	• **species: 2974 (38.809%)**<br>• genus: 543 (7.085%)<br>• family: 523 (6.825%)<br>• order: 2167 (28.278%)<br>• class: 378 (4.932%)<br>• phylum: 153 (1.996%)<br>• superkingdom: 222 (2.897%)<br>• root: 678 (8.847%)	• Salmonella enterica [taxid 28901]: 19 (0.247%)<br>• Escherichia coli [taxid 562]: 15 (0.195%)<br>• Xenorhabdus bovienii [taxid 40576]: 4 (0.052%)<br>• Morganella sp. (in: Bacteria) [taxid 1912315]: 3 (0.039%)<br>• Proteus penneri [taxid 102862]: 3 (0.039%)<br>• Morganella psychrotolerans [taxid 368603]: 3 (0.039%)<br>• Xenorhabdus mauleonii [taxid 351675]: 2 (0.026%)<br>• Buchnera aphidicola [taxid 9]: 2 (0.026%)<br>• other: 34 (0.443%)
Benchmark OTU ID: `FR687359-_Proteobacteria`<br>OTU taxon: Mycetohabitans rhizoxinica HKI 454 [taxid 882378]<br>Expected: Mycetohabitans rhizoxinica [taxid 412963] (species)<br>Number of reads: 5313<br>Number of identified reads: 5291 (99.585%)	• **species: 2046 (38.509%)**<br>• genus: 220 (4.14%)<br>• family: 1527 (28.74%)<br>• order: 159 (2.992%)<br>• class: 78 (1.468%)<br>• phylum: 351 (6.606%)<br>• superkingdom: 245 (4.611%)<br>• root: 660 (12.422%)	• Mycetohabitans endofungorum [taxid 417203]: 14 (0.263%)<br>• Burkholderia multivorans [taxid 87883]: 2 (0.037%)<br>• Candidatus Pandoraea novymonadis [taxid 1808959]: 2 (0.037%)<br>• Burkholderia cepacia [taxid 292]: 2 (0.037%)<br>• Mycoavidus cysteinexigens [taxid 1553431]: 2 (0.037%)<br>• Candidatus Paraburkholderia kirkii [taxid 198822]: 2 (0.037%)<br>• Caballeronia glathei [taxid 60547]: 2 (0.037%)<br>• Clostridium novyi [taxid 1542]: 1 (0.018%)<br>• other: 35 (0.658%)
Benchmark OTU ID: `CP000479-_Actinobacteria`<br>OTU taxon: Mycobacterium avium 104 [taxid 243243]<br>Expected: Mycobacterium avium [taxid 1764] (species)<br>Number of reads: 24426<br>Number of identified reads: 24340 (99.647%)	• species: 2125 (8.699%)<br>• **genus: 16506 (67.575%)**<br>• family: 1982 (8.114%)<br>• order: 446 (1.825%)<br>• class: 826 (3.381%)<br>• phylum: 14 (0.057%)<br>• superkingdom: 1005 (4.114%)<br>• root: 1431 (5.858%)	• Mycobacterium colombiense [taxid 339268]: 42 (0.171%)<br>• Mycobacterium lepraemurium [taxid 64667]: 34 (0.139%)<br>• Mycobacterium intracellulare [taxid 1767]: 17 (0.069%)<br>• Mycobacterium mantenii [taxid 560555]: 14 (0.057%)<br>• Mycobacterium asiaticum [taxid 1790]: 11 (0.045%)<br>• Mycobacterium tuberculosis [taxid 1773]: 11 (0.045%)<br>• Mycobacterium simiae [taxid 1784]: 11 (0.045%)<br>• Mycobacterium arosiense [taxid 425468]: 10 (0.04%)<br>• other: 272 (1.113%)
Benchmark OTU ID: `AE016958-_Actinobacteria`<br>OTU taxon: Mycobacterium avium subsp. paratuberculosis K-10 [taxid 262316]<br>Expected: Mycobacterium avium [taxid 1764] (species)<br>Number of reads: 21263<br>Number of identified reads: 21209 (99.746%)	• species: 2455 (11.545%)<br>• **genus: 14233 (66.937%)**<br>• family: 1443 (6.786%)<br>• order: 366 (1.721%)<br>• class: 669 (3.146%)<br>• phylum: 14 (0.065%)<br>• superkingdom: 813 (3.823%)<br>• root: 1209 (5.685%)	• Mycobacterium lepraemurium [taxid 64667]: 33 (0.155%)<br>• Mycobacterium colombiense [taxid 339268]: 31 (0.145%)<br>• Mycobacterium intracellulare [taxid 1767]: 15 (0.07%)<br>• Mycobacterium tuberculosis [taxid 1773]: 14 (0.065%)<br>• Mycobacterium bohemicum [taxid 56425]: 11 (0.051%)<br>• Mycobacterium conspicuum [taxid 44010]: 8 (0.037%)<br>• Mycobacterium gordonae [taxid 1778]: 7 (0.032%)<br>• Mycobacterium mantenii [taxid 560555]: 7 (0.032%)<br>• other: 185 (0.87%)

Operational Taxonomic Unit (OTU)	Correct identifications	Wrong or overspecific identifications at species rank
Benchmark OTU ID: `CP005928-_Actinobacteria`<br>OTU taxon: Mycobacterium avium subsp. paratuberculosis MAP4 [taxid 1199187]<br>Expected: Mycobacterium avium [taxid 1764] (species)<br>Number of reads: 21261<br>Number of identified reads: 21192 (99.675%)	• species: 2472 (11.626%)<br>• **genus: 14084 (66.243%)**<br>• family: 1458 (6.857%)<br>• order: 368 (1.73%)<br>• class: 706 (3.32%)<br>• phylum: 11 (0.051%)<br>• superkingdom: 903 (4.247%)<br>• root: 1176 (5.531%)	• Mycobacterium lepraemurium [taxid 64667]: 41 (0.192%)<br>• Mycobacterium colombiense [taxid 339268]: 30 (0.141%)<br>• Mycobacterium intracellulare [taxid 1767]: 20 (0.094%)<br>• Mycobacterium mantenii [taxid 560555]: 18 (0.084%)<br>• Mycobacterium tuberculosis [taxid 1773]: 18 (0.084%)<br>• Mycobacterium marseillense [taxid 701042]: 13 (0.061%)<br>• Mycobacterium arosiense [taxid 425468]: 13 (0.061%)<br>• Mycobacterium gordonae [taxid 1778]: 9 (0.042%)<br>• other: 211 (0.992%)
Benchmark OTU ID: `HE572590-_Actinobacteria`<br>OTU taxon: Mycobacterium canettii CIPT 140010059 [taxid 1048245]<br>Expected: Mycobacterium canettii [taxid 78331] (species)<br>Number of reads: 19559<br>Number of identified reads: 19448 (99.432%)	• species: 1234 (6.309%)<br>• **genus: 13337 (68.188%)**<br>• family: 1193 (6.099%)<br>• order: 347 (1.774%)<br>• class: 741 (3.788%)<br>• phylum: 13 (0.066%)<br>• superkingdom: 931 (4.759%)<br>• root: 1644 (8.405%)	• **Mycobacterium tuberculosis [taxid 1773]: 1852 (9.468%)**<br>• Mycobacterium decipiens [taxid 1430326]: 16 (0.081%)<br>• Mycobacterium kansasii [taxid 1768]: 12 (0.061%)<br>• Mycobacterium shinjukuense [taxid 398694]: 11 (0.056%)<br>• Mycobacterium simiae [taxid 1784]: 9 (0.046%)<br>• Mycobacterium riyadhense [taxid 486698]: 8 (0.04%)<br>• Mycobacterium asiaticum [taxid 1790]: 8 (0.04%)<br>• Mycobacterium lacus [taxid 169765]: 7 (0.035%)<br>• other: 166 (0.848%)
Benchmark OTU ID: `FO203507-_Actinobacteria`<br>OTU taxon: Mycobacterium canettii CIPT 140060008 [taxid 1205676]<br>Expected: Mycobacterium canettii [taxid 78331] (species)<br>Number of reads: 19295<br>Number of identified reads: 19200 (99.507%)	• species: 787 (4.078%)<br>• **genus: 13606 (70.515%)**<br>• family: 1188 (6.157%)<br>• order: 348 (1.803%)<br>• class: 743 (3.85%)<br>• phylum: 9 (0.046%)<br>• superkingdom: 907 (4.7%)<br>• root: 1599 (8.287%)	• **Mycobacterium tuberculosis [taxid 1773]: 1911 (9.904%)**<br>• Mycobacterium decipiens [taxid 1430326]: 22 (0.114%)<br>• Mycobacterium shinjukuense [taxid 398694]: 15 (0.077%)<br>• Mycobacterium ulcerans [taxid 1809]: 9 (0.046%)<br>• Mycobacterium heckeshornense [taxid 110505]: 8 (0.041%)<br>• Mycobacterium simiae [taxid 1784]: 8 (0.041%)<br>• Mycobacterium asiaticum [taxid 1790]: 7 (0.036%)<br>• Mycobacterium lacus [taxid 169765]: 6 (0.031%)<br>• other: 156 (0.808%)
Benchmark OTU ID: `FO203509-_Actinobacteria`<br>OTU taxon: Mycobacterium canettii CIPT 140070010 [taxid 1205674]<br>Expected: Mycobacterium canettii [taxid 78331] (species)<br>Number of reads: 19740<br>Number of identified reads: 19631 (99.447%)	• species: 545 (2.76%)<br>• **genus: 13013 (65.921%)**<br>• family: 1451 (7.35%)<br>• order: 433 (2.193%)<br>• class: 813 (4.118%)<br>• phylum: 22 (0.111%)<br>• superkingdom: 1102 (5.582%)<br>• root: 2234 (11.317%)	• **Mycobacterium tuberculosis [taxid 1773]: 1652 (8.368%)**<br>• Mycobacterium decipiens [taxid 1430326]: 74 (0.374%)<br>• Mycobacterium shinjukuense [taxid 398694]: 46 (0.233%)<br>• Mycobacterium riyadhense [taxid 486698]: 20 (0.101%)<br>• Mycobacterium innocens [taxid 2341083]: 14 (0.07%)<br>• Mycobacterium xenopi [taxid 1789]: 14 (0.07%)<br>• Mycobacterium parascrofulaceum [taxid 240125]: 13 (0.065%)<br>• Mycobacterium basiliense [taxid 2094119]: 13 (0.065%)<br>• other: 292 (1.479%)

Operational Taxonomic Unit (OTU)	Correct identifications	Wrong or overspecific identifications at species rank
Benchmark OTU ID: `CP003322-_Actinobacteria`<br>OTU taxon: Mycobacterium intracellulare ATCC 13950 [taxid 487521]<br>Expected: Mycobacterium intracellulare [taxid 1767] (species)<br>Number of reads: 24069<br>Number of identified reads: 24003 (99.725%)	• species: 1426 (5.924%)<br>• **genus: 16927 (70.326%)**<br>• family: 1859 (7.723%)<br>• order: 410 (1.703%)<br>• class: 779 (3.236%)<br>• phylum: 18 (0.074%)<br>• superkingdom: 946 (3.93%)<br>• root: 1624 (6.747%)	• Mycobacterium colombiense [taxid 339268]: 45 (0.186%)<br>• Mycobacterium avium [taxid 1764]: 31 (0.128%)<br>• Mycobacterium tuberculosis [taxid 1773]: 30 (0.124%)<br>• Mycobacterium asiaticum [taxid 1790]: 19 (0.078%)<br>• Mycobacterium xenopi [taxid 1789]: 16 (0.066%)<br>• Mycobacterium mantenii [taxid 560555]: 16 (0.066%)<br>• Mycobacterium vulneris [taxid 547163]: 15 (0.062%)<br>• Mycobacterium saskatchewanense [taxid 220927]: 14 (0.058%)<br>• other: 399 (1.657%)
Benchmark OTU ID: `CP003323-_Actinobacteria`<br>OTU taxon: Mycobacterium intracellulare MOTT-02 [taxid 1138382]<br>Expected: Mycobacterium intracellulare [taxid 1767] (species)<br>Number of reads: 24103<br>Number of identified reads: 24006 (99.597%)	• species: 1226 (5.086%)<br>• **genus: 16741 (69.456%)**<br>• family: 2161 (8.965%)<br>• order: 382 (1.584%)<br>• class: 741 (3.074%)<br>• phylum: 15 (0.062%)<br>• superkingdom: 1018 (4.223%)<br>• root: 1714 (7.111%)	• Mycobacterium colombiense [taxid 339268]: 53 (0.219%)<br>• Mycobacterium avium [taxid 1764]: 47 (0.194%)<br>• Mycobacterium tuberculosis [taxid 1773]: 33 (0.136%)<br>• Mycobacterium marseillense [taxid 701042]: 25 (0.103%)<br>• Mycobacterium asiaticum [taxid 1790]: 15 (0.062%)<br>• Mycobacterium gordonae [taxid 1778]: 15 (0.062%)<br>• Mycobacterium lepraemurium [taxid 64667]: 13 (0.053%)<br>• Mycobacterium rhizamassiliense [taxid 1841860]: 13 (0.053%)<br>• other: 382 (1.584%)
Benchmark OTU ID: `CP002275-_Actinobacteria`<br>OTU taxon: Mycobacterium intracellulare subsp. intracellulare MTCC 9506 [taxid 1232724]<br>Expected: Mycobacterium intracellulare [taxid 1767] (species)<br>Number of reads: 24983<br>Number of identified reads: 24880 (99.587%)	• species: 1491 (5.968%)<br>• **genus: 17499 (70.043%)**<br>• family: 1858 (7.437%)<br>• order: 376 (1.505%)<br>• class: 828 (3.314%)<br>• phylum: 11 (0.044%)<br>• superkingdom: 1029 (4.118%)<br>• root: 1771 (7.088%)	• Mycobacterium avium [taxid 1764]: 48 (0.192%)<br>• Mycobacterium colombiense [taxid 339268]: 46 (0.184%)<br>• Mycobacterium tuberculosis [taxid 1773]: 31 (0.124%)<br>• Mycobacterium marseillense [taxid 701042]: 20 (0.08%)<br>• Mycobacterium asiaticum [taxid 1790]: 17 (0.068%)<br>• Mycobacterium mantenii [taxid 560555]: 17 (0.068%)<br>• Mycobacterium simiae [taxid 1784]: 15 (0.06%)<br>• Mycobacterium parascrofulaceum [taxid 240125]: 15 (0.06%)<br>• other: 368 (1.473%)
Benchmark OTU ID: `ENA|CM000636|CM000636.1-_Actinobacteria`<br>OTU taxon: Mycobacterium kansasii [taxid 1768]<br>Expected: Mycobacterium kansasii [taxid 1768] (species)<br>Number of reads: 26503<br>Number of identified reads: 26353 (99.434%)	• species: 6756 (25.491%)<br>• **genus: 12815 (48.353%)**<br>• family: 1606 (6.059%)<br>• order: 461 (1.739%)<br>• class: 870 (3.282%)<br>• phylum: 16 (0.06%)<br>• superkingdom: 1239 (4.674%)<br>• root: 2566 (9.681%)	• Mycobacterium tuberculosis [taxid 1773]: 46 (0.173%)<br>• Mycobacterium persicum [taxid 1487726]: 37 (0.139%)<br>• Mycobacterium attenuatum [taxid 2341086]: 34 (0.128%)<br>• Mycobacterium pseudokansasii [taxid 2341080]: 32 (0.12%)<br>• Mycobacterium innocens [taxid 2341083]: 29 (0.109%)<br>• Mycobacterium simiae [taxid 1784]: 26 (0.098%)<br>• Mycobacterium basiliense [taxid 2094119]: 20 (0.075%)<br>• Mycobacterium gastri [taxid 1777]: 16 (0.06%)<br>• other: 305 (1.15%)

Operational Taxonomic Unit (OTU)	Correct identifications	Wrong or overspecific identifications at species rank
Benchmark OTU ID: `FM211192-_Actinobacteria`<br>OTU taxon: Mycobacterium leprae Br4923 [taxid 561304]<br>Expected: Mycobacterium leprae [taxid 1769] (species)<br>Number of reads: 13610<br>Number of identified reads: 13498 (99.177%)	• species: 4261 (31.307%)<br>• genus: 2203 (16.186%)<br>• family: 610 (4.481%)<br>• order: 236 (1.734%)<br>• class: 419 (3.078%)<br>• phylum: 10 (0.073%)<br>• superkingdom: 591 (4.342%)<br>• **root: 5132 (37.707%)**	• Mycobacterium haemophilum [taxid 29311]: 40 (0.293%)<br>• Mycobacterium tuberculosis [taxid 1773]: 21 (0.154%)<br>• Mycobacterium lepromatosis [taxid 480418]: 18 (0.132%)<br>• Mycobacterium uberis [taxid 2162698]: 5 (0.036%)<br>• Mycobacterium gordonae [taxid 1778]: 5 (0.036%)<br>• Mycobacterium ulcerans [taxid 1809]: 4 (0.029%)<br>• Mycobacterium helveticum [taxid 2592811]: 4 (0.029%)<br>• Mycobacterium asiaticum [taxid 1790]: 3 (0.022%)<br>• other: 89 (0.653%)
Benchmark OTU ID: `ENA|AL450380|AL450380.1-_Actinobacteria`<br>OTU taxon: Mycobacterium leprae TN [taxid 272631]<br>Expected: Mycobacterium leprae [taxid 1769] (species)<br>Number of reads: 13611<br>Number of identified reads: 13482 (99.052%)	• species: 4244 (31.18%)<br>• genus: 2271 (16.685%)<br>• family: 573 (4.209%)<br>• order: 249 (1.829%)<br>• class: 443 (3.254%)<br>• phylum: 5 (0.036%)<br>• superkingdom: 608 (4.466%)<br>• **root: 5066 (37.219%)**	• Mycobacterium haemophilum [taxid 29311]: 35 (0.257%)<br>• Mycobacterium tuberculosis [taxid 1773]: 30 (0.22%)<br>• Mycobacterium uberis [taxid 2162698]: 15 (0.11%)<br>• Mycobacterium lepromatosis [taxid 480418]: 12 (0.088%)<br>• Mycobacterium saskatchewanense [taxid 220927]: 4 (0.029%)<br>• Mycobacterium gordonae [taxid 1778]: 3 (0.022%)<br>• Mycobacterium basiliense [taxid 2094119]: 3 (0.022%)<br>• Mycobacterium shigaense [taxid 722731]: 3 (0.022%)<br>• other: 92 (0.675%)
Benchmark OTU ID: `CP003324-_Actinobacteria`<br>OTU taxon: Mycobacterium paraintracellulare [taxid 1138383]<br>Expected: Mycobacterium paraintracellulare [taxid 1138383] (species)<br>Number of reads: 24552<br>Number of identified reads: 24459 (99.621%)	• species: 1149 (4.679%)<br>• **genus: 17575 (71.582%)**<br>• family: 1904 (7.754%)<br>• order: 393 (1.6%)<br>• class: 753 (3.066%)<br>• phylum: 11 (0.044%)<br>• superkingdom: 1021 (4.158%)<br>• root: 1639 (6.675%)	• Mycobacterium intracellulare [taxid 1767]: 287 (1.168%)<br>• Mycobacterium colombiense [taxid 339268]: 43 (0.175%)<br>• Mycobacterium avium [taxid 1764]: 39 (0.158%)<br>• Mycobacterium tuberculosis [taxid 1773]: 35 (0.142%)<br>• Mycobacterium sherrisii [taxid 243061]: 23 (0.093%)<br>• Mycobacterium marseillense [taxid 701042]: 22 (0.089%)<br>• Mycobacterium asiaticum [taxid 1790]: 19 (0.077%)<br>• Mycobacterium mantenii [taxid 560555]: 17 (0.069%)<br>• other: 356 (1.449%)
Benchmark OTU ID: `CP000580-_Actinobacteria`<br>OTU taxon: Mycobacterium sp. JLS [taxid 164757]<br>Expected: Mycobacterium sp. JLS [taxid 164757] (species)<br>Number of reads: 27234<br>Number of identified reads: 27166 (99.75%)	• species: 1211 (4.446%)<br>• genus: 1409 (5.173%)<br>• **family: 19196 (70.485%)**<br>• order: 705 (2.588%)<br>• class: 1288 (4.729%)<br>• phylum: 16 (0.058%)<br>• superkingdom: 1305 (4.791%)<br>• root: 2019 (7.413%)	• Mycolicibacterium litorale [taxid 758802]: 50 (0.183%)<br>• Mycolicibacterium monacense [taxid 85693]: 25 (0.091%)<br>• Mycolicibacterium doricum [taxid 126673]: 21 (0.077%)<br>• Mycobacterium sp. MCS [taxid 164756]: 20 (0.073%)<br>• Mycolicibacterium moriokaense [taxid 39691]: 17 (0.062%)<br>• Mycobacteroides abscessus [taxid 36809]: 12 (0.044%)<br>• Mycobacterium sp. KMS [taxid 189918]: 12 (0.044%)<br>• Mycolicibacterium vanbaalenii [taxid 110539]: 11 (0.04%)<br>• other: 350 (1.285%)

Operational Taxonomic Unit (OTU)	Correct identifications	Wrong or overspecific identifications at species rank
Benchmark OTU ID: `CP000518-_Actinobacteria`<br>OTU taxon: Mycobacterium sp. KMS [taxid 189918]<br>Expected: Mycobacterium sp. KMS [taxid 189918] (species)<br>Number of reads: 25709<br>Number of identified reads: 25612 (99.622%)	• species: 69 (0.268%)<br>• genus: 3418 (13.294%)<br>• **family: 17136 (66.653%)**<br>• order: 641 (2.493%)<br>• class: 1311 (5.099%)<br>• phylum: 15 (0.058%)<br>• superkingdom: 1238 (4.815%)<br>• root: 1761 (6.849%)	• Mycolicibacterium litorale [taxid 758802]: 61 (0.237%)<br>• Mycobacterium sp. MCS [taxid 164756]: 56 (0.217%)<br>• Mycobacterium sp. JLS [taxid 164757]: 24 (0.093%)<br>• Mycolicibacterium doricum [taxid 126673]: 23 (0.089%)<br>• Mycobacteroides abscessus [taxid 36809]: 16 (0.062%)<br>• Mycolicibacterium monacense [taxid 85693]: 16 (0.062%)<br>• Mycolicibacterium gilvum [taxid 1804]: 9 (0.035%)<br>• Mycolicibacterium hassiacum [taxid 46351]: 8 (0.031%)<br>• other: 326 (1.268%)
Benchmark OTU ID: `CP000384-_Actinobacteria`<br>OTU taxon: Mycobacterium sp. MCS [taxid 164756]<br>Expected: Mycobacterium sp. MCS [taxid 164756] (species)<br>Number of reads: 25553<br>Number of identified reads: 25465 (99.655%)	• species: 78 (0.305%)<br>• genus: 3341 (13.074%)<br>• **family: 16993 (66.5%)**<br>• order: 690 (2.7%)<br>• class: 1304 (5.103%)<br>• phylum: 24 (0.093%)<br>• superkingdom: 1164 (4.555%)<br>• root: 1852 (7.247%)	• Mycolicibacterium litorale [taxid 758802]: 50 (0.195%)<br>• Mycobacterium sp. JLS [taxid 164757]: 32 (0.125%)<br>• Mycobacterium sp. KMS [taxid 189918]: 31 (0.121%)<br>• Mycolicibacterium doricum [taxid 126673]: 30 (0.117%)<br>• Mycobacteroides abscessus [taxid 36809]: 22 (0.086%)<br>• Mycolicibacterium monacense [taxid 85693]: 19 (0.074%)<br>• Mycolicibacterium moriokaense [taxid 39691]: 11 (0.043%)<br>• Mycobacterium gordonae [taxid 1778]: 10 (0.039%)<br>• other: 360 (1.408%)
Benchmark OTU ID: `ENA|CM000789|CM000789.2-_Actinobacteria`<br>OTU taxon: Mycobacterium tuberculosis [taxid 1773]<br>Expected: Mycobacterium tuberculosis [taxid 1773] (species)<br>Number of reads: 18849<br>Number of identified reads: 18750 (99.474%)	• species: 2420 (12.838%)<br>• **genus: 11779 (62.491%)**<br>• family: 1158 (6.143%)<br>• order: 349 (1.851%)<br>• class: 688 (3.65%)<br>• phylum: 8 (0.042%)<br>• superkingdom: 858 (4.551%)<br>• root: 1478 (7.841%)	• Mycobacterium decipiens [taxid 1430326]: 28 (0.148%)<br>• Mycobacterium shinjukuense [taxid 398694]: 14 (0.074%)<br>• Mycobacterium basiliense [taxid 2094119]: 9 (0.047%)<br>• Mycobacterium canettii [taxid 78331]: 8 (0.042%)<br>• Mycobacterium riyadhense [taxid 486698]: 8 (0.042%)<br>• Mycobacterium gordonae [taxid 1778]: 7 (0.037%)<br>• Mycobacterium asiaticum [taxid 1790]: 7 (0.037%)<br>• Mycobacterium lacus [taxid 169765]: 5 (0.026%)<br>• other: 132 (0.7%)
Benchmark OTU ID: `CP005386-_Actinobacteria`<br>OTU taxon: Mycobacterium tuberculosis CAS/NITR204 [taxid 1310114]<br>Expected: Mycobacterium tuberculosis [taxid 1773] (species)<br>Number of reads: 19122<br>Number of identified reads: 19004 (99.382%)	• species: 2837 (14.836%)<br>• **genus: 11248 (58.822%)**<br>• family: 1178 (6.16%)<br>• order: 356 (1.861%)<br>• class: 703 (3.676%)<br>• phylum: 14 (0.073%)<br>• superkingdom: 1035 (5.412%)<br>• root: 1620 (8.471%)	• Mycobacterium decipiens [taxid 1430326]: 31 (0.162%)<br>• Mycobacterium canettii [taxid 78331]: 13 (0.067%)<br>• Mycobacterium shinjukuense [taxid 398694]: 9 (0.047%)<br>• Mycobacterium simiae [taxid 1784]: 6 (0.031%)<br>• Mycobacterium uberis [taxid 2162698]: 6 (0.031%)<br>• Mycobacterium riyadhense [taxid 486698]: 6 (0.031%)<br>• Mycobacterium orygis [taxid 1305738]: 6 (0.031%)<br>• Mycobacterium kansasii [taxid 1768]: 5 (0.026%)<br>• other: 137 (0.716%)

Operational Taxonomic Unit (OTU)	Correct identifications	Wrong or overspecific identifications at species rank
Benchmark OTU ID: `CP002992-_Actinobacteria`<br>OTU taxon: Mycobacterium tuberculosis CTRI-2 [taxid 707235]<br>Expected: Mycobacterium tuberculosis [taxid 1773] (species)<br>Number of reads: 19150<br>Number of identified reads: 19039 (99.42%)	• species: 2519 (13.154%)<br>• **genus: 11837 (61.812%)**<br>• family: 1214 (6.339%)<br>• order: 337 (1.759%)<br>• class: 632 (3.3%)<br>• phylum: 16 (0.083%)<br>• superkingdom: 901 (4.704%)<br>• root: 1570 (8.198%)	• Mycobacterium decipiens [taxid 1430326]: 25 (0.13%)<br>• Mycobacterium simiae [taxid 1784]: 10 (0.052%)<br>• Mycobacterium canettii [taxid 78331]: 7 (0.036%)<br>• Mycobacterium gordonae [taxid 1778]: 7 (0.036%)<br>• Mycobacterium kyorinense [taxid 487514]: 5 (0.026%)<br>• Mycobacterium riyadhense [taxid 486698]: 5 (0.026%)<br>• Mycobacterium orygis [taxid 1305738]: 5 (0.026%)<br>• Mycobacterium bohemicum [taxid 56425]: 5 (0.026%)<br>• other: 146 (0.762%)
Benchmark OTU ID: `CP000611-_Actinobacteria`<br>OTU taxon: Mycobacterium tuberculosis H37Ra [taxid 419947]<br>Expected: Mycobacterium tuberculosis [taxid 1773] (species)<br>Number of reads: 19255<br>Number of identified reads: 19160 (99.506%)	• species: 2653 (13.778%)<br>• **genus: 11897 (61.786%)**<br>• family: 1200 (6.232%)<br>• order: 353 (1.833%)<br>• class: 648 (3.365%)<br>• phylum: 10 (0.051%)<br>• superkingdom: 874 (4.539%)<br>• root: 1519 (7.888%)	• Mycobacterium decipiens [taxid 1430326]: 19 (0.098%)<br>• Mycobacterium asiaticum [taxid 1790]: 9 (0.046%)<br>• Mycobacterium simiae [taxid 1784]: 7 (0.036%)<br>• Mycobacterium canettii [taxid 78331]: 7 (0.036%)<br>• Mycobacterium riyadhense [taxid 486698]: 6 (0.031%)<br>• Mycobacterium kansasii [taxid 1768]: 5 (0.025%)<br>• Mycobacterium lepraemurium [taxid 64667]: 5 (0.025%)<br>• Mycobacterium conspicuum [taxid 44010]: 5 (0.025%)<br>• other: 144 (0.747%)
Benchmark OTU ID: `CP001976-_Actinobacteria`<br>OTU taxon: Mycobacterium tuberculosis KZN 605 [taxid 478435]<br>Expected: Mycobacterium tuberculosis [taxid 1773] (species)<br>Number of reads: 19153<br>Number of identified reads: 19063 (99.53%)	• species: 2538 (13.251%)<br>• **genus: 11833 (61.781%)**<br>• family: 1169 (6.103%)<br>• order: 376 (1.963%)<br>• class: 674 (3.519%)<br>• phylum: 8 (0.041%)<br>• superkingdom: 888 (4.636%)<br>• root: 1564 (8.165%)	• Mycobacterium decipiens [taxid 1430326]: 24 (0.125%)<br>• Mycobacterium canettii [taxid 78331]: 12 (0.062%)<br>• Mycobacterium gordonae [taxid 1778]: 7 (0.036%)<br>• Mycobacterium asiaticum [taxid 1790]: 6 (0.031%)<br>• Mycobacterium simiae [taxid 1784]: 5 (0.026%)<br>• Mycobacterium kansasii [taxid 1768]: 5 (0.026%)<br>• Mycobacterium attenuatum [taxid 2341086]: 4 (0.02%)<br>• Mycobacterium lepromatosis [taxid 480418]: 4 (0.02%)<br>• other: 128 (0.668%)
Benchmark OTU ID: `CP003233-_Actinobacteria`<br>OTU taxon: Mycobacterium tuberculosis RGTB327 [taxid 1091500]<br>Expected: Mycobacterium tuberculosis [taxid 1773] (species)<br>Number of reads: 19059<br>Number of identified reads: 18959 (99.475%)	• species: 2562 (13.442%)<br>• **genus: 11681 (61.288%)**<br>• family: 1167 (6.123%)<br>• order: 325 (1.705%)<br>• class: 658 (3.452%)<br>• phylum: 10 (0.052%)<br>• superkingdom: 932 (4.89%)<br>• root: 1608 (8.436%)	• Mycobacterium decipiens [taxid 1430326]: 20 (0.104%)<br>• Mycobacterium canettii [taxid 78331]: 11 (0.057%)<br>• Mycobacterium kansasii [taxid 1768]: 8 (0.041%)<br>• Mycobacterium shinjukuense [taxid 398694]: 8 (0.041%)<br>• Mycobacterium gordonae [taxid 1778]: 7 (0.036%)<br>• Mycobacterium riyadhense [taxid 486698]: 6 (0.031%)<br>• Mycobacterium uberis [taxid 2162698]: 5 (0.026%)<br>• Mycobacterium simiae [taxid 1784]: 5 (0.026%)<br>• other: 122 (0.64%)

Operational Taxonomic Unit (OTU)	Correct identifications	Wrong or overspecific identifications at species rank
Benchmark OTU ID: `HE608151-_Actinobacteria`<br>OTU taxon: Mycobacterium tuberculosis UT205 [taxid 1097669]<br>Expected: Mycobacterium tuberculosis [taxid 1773] (species)<br>Number of reads: 18246<br>Number of identified reads: 18152 (99.484%)	• species: 2247 (12.315%)<br>• **genus: 11447 (62.737%)**<br>• family: 1128 (6.182%)<br>• order: 343 (1.879%)<br>• class: 610 (3.343%)<br>• phylum: 17 (0.093%)<br>• superkingdom: 851 (4.664%)<br>• root: 1501 (8.226%)	• Mycobacterium decipiens [taxid 1430326]: 21 (0.115%)<br>• Mycobacterium canettii [taxid 78331]: 11 (0.06%)<br>• Mycobacterium kansasii [taxid 1768]: 8 (0.043%)<br>• Mycobacterium riyadhense [taxid 486698]: 7 (0.038%)<br>• Mycobacterium asiaticum [taxid 1790]: 5 (0.027%)<br>• Mycobacterium lacus [taxid 169765]: 4 (0.021%)<br>• Mycobacterium simiae [taxid 1784]: 4 (0.021%)<br>• Mycobacterium attenuatum [taxid 2341086]: 4 (0.021%)<br>• other: 99 (0.542%)
Benchmark OTU ID: `CP005082-_Actinobacteria`<br>OTU taxon: Mycobacterium tuberculosis str. Beijing/NITR203 [taxid 1306400]<br>Expected: Mycobacterium tuberculosis [taxid 1773] (species)<br>Number of reads: 19211<br>Number of identified reads: 19105 (99.448%)	• species: 2478 (12.898%)<br>• **genus: 11990 (62.412%)**<br>• family: 1095 (5.699%)<br>• order: 352 (1.832%)<br>• class: 684 (3.56%)<br>• phylum: 10 (0.052%)<br>• superkingdom: 916 (4.768%)<br>• root: 1572 (8.182%)	• Mycobacterium decipiens [taxid 1430326]: 24 (0.124%)<br>• Mycobacterium asiaticum [taxid 1790]: 12 (0.062%)<br>• Mycobacterium canettii [taxid 78331]: 10 (0.052%)<br>• Mycobacterium kansasii [taxid 1768]: 7 (0.036%)<br>• Mycobacterium orygis [taxid 1305738]: 6 (0.031%)<br>• Mycobacterium ulcerans [taxid 1809]: 6 (0.031%)<br>• Mycobacterium basiliense [taxid 2094119]: 5 (0.026%)<br>• Mycobacterium lacus [taxid 169765]: 4 (0.02%)<br>• other: 119 (0.619%)
Benchmark OTU ID: `AP012340-_Actinobacteria`<br>OTU taxon: Mycobacterium tuberculosis str. Erdman = ATCC 35801 [taxid 652616]<br>Expected: Mycobacterium tuberculosis [taxid 1773] (species)<br>Number of reads: 19119<br>Number of identified reads: 19007 (99.414%)	• species: 2520 (13.18%)<br>• **genus: 11875 (62.11%)**<br>• family: 1090 (5.701%)<br>• order: 365 (1.909%)<br>• class: 635 (3.321%)<br>• phylum: 20 (0.104%)<br>• superkingdom: 873 (4.566%)<br>• root: 1622 (8.483%)	• Mycobacterium decipiens [taxid 1430326]: 20 (0.104%)<br>• Mycobacterium canettii [taxid 78331]: 11 (0.057%)<br>• Mycobacterium basiliense [taxid 2094119]: 8 (0.041%)<br>• Mycobacterium riyadhense [taxid 486698]: 8 (0.041%)<br>• Mycobacterium intermedium [taxid 28445]: 7 (0.036%)<br>• Mycobacterium orygis [taxid 1305738]: 5 (0.026%)<br>• Mycobacterium xenopi [taxid 1789]: 5 (0.026%)<br>• Mycobacterium shinjukuense [taxid 398694]: 5 (0.026%)<br>• other: 156 (0.815%)
Benchmark OTU ID: `CP004886-_Actinobacteria`<br>OTU taxon: Mycobacterium tuberculosis str. Haarlem/NITR202 [taxid 1304279]<br>Expected: Mycobacterium tuberculosis [taxid 1773] (species)<br>Number of reads: 19171<br>Number of identified reads: 19080 (99.525%)	• species: 2365 (12.336%)<br>• **genus: 11419 (59.563%)**<br>• family: 1247 (6.504%)<br>• order: 373 (1.945%)<br>• class: 665 (3.468%)<br>• phylum: 16 (0.083%)<br>• superkingdom: 908 (4.736%)<br>• root: 2077 (10.834%)	• Mycobacterium decipiens [taxid 1430326]: 17 (0.088%)<br>• Mycobacterium canettii [taxid 78331]: 14 (0.073%)<br>• Mycobacterium riyadhense [taxid 486698]: 9 (0.046%)<br>• Mycobacterium shinjukuense [taxid 398694]: 9 (0.046%)<br>• Mycobacterium basiliense [taxid 2094119]: 8 (0.041%)<br>• Mycobacterium uberis [taxid 2162698]: 6 (0.031%)<br>• Mycobacterium lacus [taxid 169765]: 5 (0.026%)<br>• Mycobacterium simiae [taxid 1784]: 5 (0.026%)<br>• other: 105 (0.547%)

Operational Taxonomic Unit (OTU)	Correct identifications	Wrong or overspecific identifications at species rank
Benchmark OTU ID: `FR878060-_Actinobacteria`<br>OTU taxon: Mycobacterium tuberculosis variant africanum GM041182 [taxid 572418]<br>Expected: Mycobacterium tuberculosis [taxid 1773] (species)<br>Number of reads: 19105<br>Number of identified reads: 19000 (99.45%)	• species: 2535 (13.268%)<br>• **genus: 11803 (61.779%)**<br>• family: 1148 (6.008%)<br>• order: 334 (1.748%)<br>• class: 662 (3.465%)<br>• phylum: 13 (0.068%)<br>• superkingdom: 847 (4.433%)<br>• root: 1646 (8.615%)	• Mycobacterium decipiens [taxid 1430326]: 23 (0.12%)<br>• Mycobacterium canettii [taxid 78331]: 16 (0.083%)<br>• Mycobacterium kansasii [taxid 1768]: 11 (0.057%)<br>• Mycobacterium asiaticum [taxid 1790]: 7 (0.036%)<br>• Mycobacterium shinjukuense [taxid 398694]: 7 (0.036%)<br>• Mycobacterium simiae [taxid 1784]: 7 (0.036%)<br>• Mycobacterium colombiense [taxid 339268]: 5 (0.026%)<br>• Mycobacterium basiliense [taxid 2094119]: 5 (0.026%)<br>• other: 127 (0.664%)
Benchmark OTU ID: `CP003900-_Actinobacteria`<br>OTU taxon: Mycobacterium tuberculosis variant bovis BCG str. Korea 1168P [taxid 1206780]<br>Expected: Mycobacterium tuberculosis [taxid 1773] (species)<br>Number of reads: 19043<br>Number of identified reads: 18950 (99.511%)	• species: 2491 (13.08%)<br>• **genus: 11785 (61.886%)**<br>• family: 1159 (6.086%)<br>• order: 378 (1.984%)<br>• class: 672 (3.528%)<br>• phylum: 20 (0.105%)<br>• superkingdom: 888 (4.663%)<br>• root: 1543 (8.102%)	• Mycobacterium decipiens [taxid 1430326]: 20 (0.105%)<br>• Mycobacterium canettii [taxid 78331]: 11 (0.057%)<br>• Mycobacterium simiae [taxid 1784]: 6 (0.031%)<br>• Mycobacterium shinjukuense [taxid 398694]: 6 (0.031%)<br>• Mycobacterium riyadhense [taxid 486698]: 6 (0.031%)<br>• Mycobacterium gordonae [taxid 1778]: 5 (0.026%)<br>• Mycobacterium ulcerans [taxid 1809]: 5 (0.026%)<br>• Mycobacterium malmoense [taxid 1780]: 5 (0.026%)<br>• other: 127 (0.666%)
Benchmark OTU ID: `CP002095-_Actinobacteria`<br>OTU taxon: Mycobacterium tuberculosis variant bovis BCG str. Mexico [taxid 717522]<br>Expected: Mycobacterium tuberculosis [taxid 1773] (species)<br>Number of reads: 18912<br>Number of identified reads: 18800 (99.407%)	• species: 2534 (13.398%)<br>• **genus: 11779 (62.283%)**<br>• family: 1125 (5.948%)<br>• order: 340 (1.797%)<br>• class: 639 (3.378%)<br>• phylum: 18 (0.095%)<br>• superkingdom: 848 (4.483%)<br>• root: 1507 (7.968%)	• Mycobacterium decipiens [taxid 1430326]: 25 (0.132%)<br>• Mycobacterium shinjukuense [taxid 398694]: 10 (0.052%)<br>• Mycobacterium canettii [taxid 78331]: 9 (0.047%)<br>• Mycobacterium asiaticum [taxid 1790]: 8 (0.042%)<br>• Mycobacterium kansasii [taxid 1768]: 8 (0.042%)<br>• Mycobacterium riyadhense [taxid 486698]: 6 (0.031%)<br>• Mycobacterium uberis [taxid 2162698]: 6 (0.031%)<br>• Mycobacterium simiae [taxid 1784]: 6 (0.031%)<br>• other: 119 (0.629%)
Benchmark OTU ID: `AM408590-_Actinobacteria`<br>OTU taxon: Mycobacterium tuberculosis variant bovis BCG str. Pasteur 1173P2 [taxid 410289]<br>Expected: Mycobacterium tuberculosis [taxid 1773] (species)<br>Number of reads: 19032<br>Number of identified reads: 18933 (99.479%)	• species: 2557 (13.435%)<br>• **genus: 11800 (62.0%)**<br>• family: 1145 (6.016%)<br>• order: 342 (1.796%)<br>• class: 663 (3.483%)<br>• phylum: 10 (0.052%)<br>• superkingdom: 895 (4.702%)<br>• root: 1509 (7.928%)	• Mycobacterium decipiens [taxid 1430326]: 23 (0.12%)<br>• Mycobacterium canettii [taxid 78331]: 12 (0.063%)<br>• Mycobacterium shinjukuense [taxid 398694]: 9 (0.047%)<br>• Mycobacterium simiae [taxid 1784]: 7 (0.036%)<br>• Mycobacterium kansasii [taxid 1768]: 7 (0.036%)<br>• Mycobacterium xenopi [taxid 1789]: 6 (0.031%)<br>• Mycobacterium asiaticum [taxid 1790]: 6 (0.031%)<br>• Mycobacterium avium [taxid 1764]: 6 (0.031%)<br>• other: 144 (0.756%)

Operational Taxonomic Unit (OTU)	Correct identifications	Wrong or overspecific identifications at species rank
Benchmark OTU ID: `AP010918-_Actinobacteria`<br>OTU taxon: Mycobacterium tuberculosis variant bovis BCG str. Tokyo 172 [taxid 561275]<br>Expected: Mycobacterium tuberculosis [taxid 1773] (species)<br>Number of reads: 19018<br>Number of identified reads: 18923 (99.5%)	• species: 2493 (13.108%)<br>• **genus: 11761 (61.841%)**<br>• family: 1167 (6.136%)<br>• order: 356 (1.871%)<br>• class: 678 (3.565%)<br>• phylum: 17 (0.089%)<br>• superkingdom: 877 (4.611%)<br>• root: 1568 (8.244%)	• Mycobacterium decipiens [taxid 1430326]: 16 (0.084%)<br>• Mycobacterium canettii [taxid 78331]: 10 (0.052%)<br>• Mycobacterium riyadhense [taxid 486698]: 8 (0.042%)<br>• Mycobacterium gordonae [taxid 1778]: 6 (0.031%)<br>• Mycobacterium shinjukuense [taxid 398694]: 6 (0.031%)<br>• Mycobacterium kansasii [taxid 1768]: 6 (0.031%)<br>• Mycobacterium lacus [taxid 169765]: 5 (0.026%)<br>• Mycobacterium basiliense [taxid 2094119]: 5 (0.026%)<br>• other: 136 (0.715%)
Benchmark OTU ID: `CP000325-_Actinobacteria`<br>OTU taxon: Mycobacterium ulcerans Agy99 [taxid 362242]<br>Expected: Mycobacterium ulcerans [taxid 1809] (species)<br>Number of reads: 25192<br>Number of identified reads: 25083 (99.567%)	• species: 3853 (15.294%)<br>• **genus: 14896 (59.129%)**<br>• family: 1532 (6.081%)<br>• order: 473 (1.877%)<br>• class: 827 (3.282%)<br>• phylum: 9 (0.035%)<br>• superkingdom: 1162 (4.612%)<br>• root: 2311 (9.173%)	• Mycobacterium marinum [taxid 1781]: 187 (0.742%)<br>• Mycobacterium pseudoshottsii [taxid 265949]: 169 (0.67%)<br>• Mycobacterium liflandii [taxid 261524]: 80 (0.317%)<br>• Mycobacterium tuberculosis [taxid 1773]: 33 (0.13%)<br>• Mycobacterium asiaticum [taxid 1790]: 15 (0.059%)<br>• Mycobacterium kansasii [taxid 1768]: 13 (0.051%)<br>• Mycobacterium basiliense [taxid 2094119]: 13 (0.051%)<br>• Mycobacterium haemophilum [taxid 29311]: 10 (0.039%)<br>• other: 263 (1.043%)
Benchmark OTU ID: `CU458896-_Actinobacteria`<br>OTU taxon: Mycobacteroides abscessus ATCC 19977 [taxid 561007]<br>Expected: Mycobacteroides abscessus [taxid 36809] (species)<br>Number of reads: 22426<br>Number of identified reads: 22338 (99.607%)	• **species: 9635 (42.963%)**<br>• genus: 6627 (29.55%)<br>• family: 1716 (7.651%)<br>• order: 654 (2.916%)<br>• class: 861 (3.839%)<br>• phylum: 21 (0.093%)<br>• superkingdom: 938 (4.182%)<br>• root: 1880 (8.383%)	• Mycobacteroides franklinii [taxid 948102]: 26 (0.115%)<br>• Mycobacteroides salmoniphilum [taxid 404941]: 25 (0.111%)<br>• Mycobacteroides immunogenum [taxid 83262]: 15 (0.066%)<br>• Mycobacteroides saopaulense [taxid 1578165]: 7 (0.031%)<br>• Mycobacteroides chelonae [taxid 1774]: 4 (0.017%)<br>• [Mycobacterium] stephanolepidis [taxid 1520670]: 4 (0.017%)<br>• Mycobacterium tuberculosis [taxid 1773]: 3 (0.013%)<br>• Gordonia rhizosphera [taxid 83341]: 2 (0.008%)<br>• other: 59 (0.263%)
Benchmark OTU ID: `CP003699-_Actinobacteria`<br>OTU taxon: Mycobacteroides abscessus subsp. massiliense str. GO 06 [taxid 1198627]<br>Expected: Mycobacteroides abscessus [taxid 36809] (species)<br>Number of reads: 22434<br>Number of identified reads: 22363 (99.683%)	• **species: 9493 (42.315%)**<br>• genus: 6812 (30.364%)<br>• family: 1755 (7.822%)<br>• order: 603 (2.687%)<br>• class: 890 (3.967%)<br>• phylum: 13 (0.057%)<br>• superkingdom: 926 (4.127%)<br>• root: 1855 (8.268%)	• Mycobacteroides salmoniphilum [taxid 404941]: 23 (0.102%)<br>• Mycobacteroides franklinii [taxid 948102]: 17 (0.075%)<br>• Mycobacteroides saopaulense [taxid 1578165]: 17 (0.075%)<br>• Mycobacteroides immunogenum [taxid 83262]: 10 (0.044%)<br>• [Mycobacterium] stephanolepidis [taxid 1520670]: 6 (0.026%)<br>• Mycobacterium tuberculosis [taxid 1773]: 4 (0.017%)<br>• Corynebacterium rouxii [taxid 2719119]: 3 (0.013%)<br>• Mycobacterium ulcerans [taxid 1809]: 3 (0.013%)<br>• other: 73 (0.325%)

Operational Taxonomic Unit (OTU)	Correct identifications	Wrong or overspecific identifications at species rank
Benchmark OTU ID: `CP002329-_Actinobacteria`<br>OTU taxon: Mycolicibacter sinensis [taxid 875328]<br>Expected: Mycolicibacter sinensis [taxid 875328] (species)<br>Number of reads: 20351<br>Number of identified reads: 20294 (99.719%)	• species: 2814 (13.827%)<br>• **genus: 9810 (48.204%)**<br>• family: 3601 (17.694%)<br>• order: 477 (2.343%)<br>• class: 897 (4.407%)<br>• phylum: 19 (0.093%)<br>• superkingdom: 1060 (5.208%)<br>• root: 1601 (7.866%)	• Mycolicibacter algericus [taxid 1288388]: 89 (0.437%)<br>• Mycolicibacter senuensis [taxid 386913]: 84 (0.412%)<br>• Mycolicibacter heraklionensis [taxid 512402]: 25 (0.122%)<br>• Mycolicibacter kumamotonensis [taxid 354243]: 20 (0.098%)<br>• Mycolicibacter terrae [taxid 1788]: 20 (0.098%)<br>• Mycolicibacter arupensis [taxid 342002]: 7 (0.034%)<br>• Mycolicibacter longobardus [taxid 1108812]: 7 (0.034%)<br>• Mycobacterium tuberculosis [taxid 1773]: 6 (0.029%)<br>• other: 135 (0.663%)
Benchmark OTU ID: `CP003053-_Actinobacteria`<br>OTU taxon: Mycolicibacterium chubuense NBB4 [taxid 710421]<br>Expected: Mycolicibacterium chubuense [taxid 1800] (species)<br>Number of reads: 24957<br>Number of identified reads: 24874 (99.667%)	• **species: 12269 (49.16%)**<br>• genus: 1053 (4.219%)<br>• family: 6478 (25.956%)<br>• order: 637 (2.552%)<br>• class: 1226 (4.912%)<br>• phylum: 25 (0.1%)<br>• superkingdom: 1233 (4.94%)<br>• root: 1940 (7.773%)	• Mycolicibacterium rhodesiae [taxid 36814]: 10 (0.04%)<br>• Mycolicibacterium obuense [taxid 1807]: 9 (0.036%)<br>• Mycolicibacterium moriokaense [taxid 39691]: 8 (0.032%)<br>• Mycolicibacterium duvalii [taxid 39688]: 8 (0.032%)<br>• Mycobacterium uberis [taxid 2162698]: 6 (0.024%)<br>• Mycolicibacterium vanbaalenii [taxid 110539]: 5 (0.02%)<br>• Mycolicibacterium gilvum [taxid 1804]: 5 (0.02%)<br>• Mycolicibacterium hodleri [taxid 49897]: 4 (0.016%)<br>• other: 194 (0.777%)
Benchmark OTU ID: `CP000656-_Actinobacteria`<br>OTU taxon: Mycolicibacterium gilvum PYR-GCK [taxid 350054]<br>Expected: Mycolicibacterium gilvum [taxid 1804] (species)<br>Number of reads: 25133<br>Number of identified reads: 25060 (99.709%)	• **species: 10861 (43.214%)**<br>• genus: 1497 (5.956%)<br>• family: 7495 (29.821%)<br>• order: 689 (2.741%)<br>• class: 1232 (4.901%)<br>• phylum: 24 (0.095%)<br>• superkingdom: 1289 (5.128%)<br>• root: 1956 (7.782%)	• Mycolicibacterium moriokaense [taxid 39691]: 12 (0.047%)<br>• Mycolicibacterium vanbaalenii [taxid 110539]: 10 (0.039%)<br>• Mycolicibacterium iranicum [taxid 912594]: 10 (0.039%)<br>• Mycolicibacterium duvalii [taxid 39688]: 10 (0.039%)<br>• Mycolicibacterium obuense [taxid 1807]: 8 (0.031%)<br>• Mycolicibacterium elephantis [taxid 81858]: 8 (0.031%)<br>• Mycobacterium tuberculosis [taxid 1773]: 6 (0.023%)<br>• Mycobacteroides abscessus [taxid 36809]: 6 (0.023%)<br>• other: 228 (0.907%)
Benchmark OTU ID: `CP002385-_Actinobacteria`<br>OTU taxon: Mycolicibacterium gilvum Spyr1 [taxid 278137]<br>Expected: Mycolicibacterium gilvum [taxid 1804] (species)<br>Number of reads: 24781<br>Number of identified reads: 24732 (99.802%)	• **species: 10730 (43.299%)**<br>• genus: 1367 (5.516%)<br>• family: 7451 (30.067%)<br>• order: 708 (2.857%)<br>• class: 1265 (5.104%)<br>• phylum: 15 (0.06%)<br>• superkingdom: 1232 (4.971%)<br>• root: 1947 (7.856%)	• Mycolicibacterium iranicum [taxid 912594]: 12 (0.048%)<br>• Mycolicibacterium smegmatis [taxid 1772]: 12 (0.048%)<br>• Mycolicibacterium vanbaalenii [taxid 110539]: 10 (0.04%)<br>• Mycolicibacterium moriokaense [taxid 39691]: 10 (0.04%)<br>• Mycolicibacterium obuense [taxid 1807]: 8 (0.032%)<br>• Mycolicibacterium aurum [taxid 1791]: 7 (0.028%)<br>• Mycolicibacterium duvalii [taxid 39688]: 7 (0.028%)<br>• Mycolicibacterium aromaticivorans [taxid 318425]: 6 (0.024%)<br>• other: 202 (0.815%)

Operational Taxonomic Unit (OTU)	Correct identifications	Wrong or overspecific identifications at species rank
Benchmark OTU ID: `CP003169-_Actinobacteria`<br>OTU taxon: Mycolicibacterium rhodesiae NBB3 [taxid 710685]<br>Expected: Mycolicibacterium rhodesiae [taxid 36814] (species)<br>Number of reads: 29034<br>Number of identified reads: 28937 (99.665%)	• **species: 13207 (45.488%)**<br>• genus: 2580 (8.886%)<br>• family: 6962 (23.978%)<br>• order: 660 (2.273%)<br>• class: 1182 (4.071%)<br>• phylum: 38 (0.13%)<br>• superkingdom: 1344 (4.629%)<br>• root: 2938 (10.119%)	• Mycolicibacterium moriokaense [taxid 39691]: 36 (0.123%)<br>• Mycolicibacterium tusciae [taxid 75922]: 24 (0.082%)<br>• Mycobacterium gordonae [taxid 1778]: 13 (0.044%)<br>• Mycobacterium tuberculosis [taxid 1773]: 13 (0.044%)<br>• Mycobacteroides abscessus [taxid 36809]: 9 (0.03%)<br>• Mycolicibacterium chubuense [taxid 1800]: 7 (0.024%)<br>• Mycolicibacterium novocastrense [taxid 59813]: 7 (0.024%)<br>• Mycolicibacterium elephantis [taxid 81858]: 6 (0.02%)<br>• other: 244 (0.84%)
Benchmark OTU ID: `CP001034-_Firmicutes`<br>OTU taxon: Natranaerobius thermophilus JW/NM-WN-LF [taxid 457570]<br>Expected: Natranaerobius thermophilus [taxid 375929] (species)<br>Number of reads: 4296<br>Number of identified reads: 4241 (98.719%)	• **species: 3298 (76.769%)**<br>• genus: 38 (0.884%)<br>• family: 0 (0.0%)<br>• order: 0 (0.0%)<br>• class: 63 (1.466%)<br>• phylum: 108 (2.513%)<br>• superkingdom: 264 (6.145%)<br>• root: 469 (10.917%)	• Natranaerobius trueperi [taxid 759412]: 2 (0.046%)<br>• Lacticaseibacillus rhamnosus [taxid 47715]: 1 (0.023%)<br>• Candidatus Kentron sp. G [taxid 2126341]: 1 (0.023%)<br>• Acrocarpospora macrocephala [taxid 150177]: 1 (0.023%)<br>• Poriferisphaera corsica [taxid 2528020]: 1 (0.023%)<br>• Alkalihalobacillus pseudofirmus [taxid 79885]: 1 (0.023%)<br>• Paenibacillus methanolicus [taxid 582686]: 1 (0.023%)<br>• [Eubacterium] cellulosolvens [taxid 29322]: 1 (0.023%)<br>• other: 3 (0.069%)
Benchmark OTU ID: `CP001932-_Euryarchaeota`<br>OTU taxon: Natrialba magadii ATCC 43099 [taxid 547559]<br>Expected: Natrialba magadii [taxid 13769] (species)<br>Number of reads: 3542<br>Number of identified reads: 3528 (99.604%)	• **species: 1133 (31.987%)**<br>• genus: 607 (17.137%)<br>• family: 475 (13.41%)<br>• order: 0 (0.0%)<br>• class: 260 (7.34%)<br>• phylum: 3 (0.084%)<br>• superkingdom: 5 (0.141%)<br>• root: 985 (27.809%)	• Natrialba chahannaoensis [taxid 68911]: 5 (0.141%)<br>• Natrialba hulunbeirensis [taxid 123783]: 3 (0.084%)<br>• Haloterrigena hispanica [taxid 392421]: 2 (0.056%)<br>• Natronococcus occultus [taxid 29288]: 2 (0.056%)<br>• Natrialba asiatica [taxid 64602]: 1 (0.028%)<br>• Haloarchaeobius iranensis [taxid 996166]: 1 (0.028%)<br>• Natrarchaeobius halalkaliphilus [taxid 1679091]: 1 (0.028%)<br>• Natronolimnohabitans innermongolicus [taxid 253107]: 1 (0.028%)<br>• other: 12 (0.338%)
Benchmark OTU ID: `CP003372-_Euryarchaeota`<br>OTU taxon: Natrinema pellirubrum DSM 15624 [taxid 797303]<br>Expected: Natrinema pellirubrum [taxid 69525] (species)<br>Number of reads: 3583<br>Number of identified reads: 3567 (99.553%)	• species: 780 (21.769%)<br>• genus: 75 (2.093%)<br>• **family: 1401 (39.101%)**<br>• order: 0 (0.0%)<br>• class: 346 (9.656%)<br>• phylum: 3 (0.083%)<br>• superkingdom: 6 (0.167%)<br>• root: 906 (25.286%)	• Haloterrigena saccharevitans [taxid 301967]: 7 (0.195%)<br>• Haloterrigena thermotolerans [taxid 121872]: 7 (0.195%)<br>• Natrinema versiforme [taxid 88724]: 7 (0.195%)<br>• Haloterrigena hispanica [taxid 392421]: 4 (0.111%)<br>• Halolamina pelagica [taxid 699431]: 3 (0.083%)<br>• Natrinema altunense [taxid 222984]: 3 (0.083%)<br>• Natrinema ejinorense [taxid 373386]: 2 (0.055%)<br>• Natronomonas salsuginis [taxid 2217661]: 2 (0.055%)<br>• other: 33 (0.921%)

Operational Taxonomic Unit (OTU)	Correct identifications	Wrong or overspecific identifications at species rank
Benchmark OTU ID: `CP003412-_Euryarchaeota`<br>OTU taxon: Natrinema sp. J7-2 [taxid 406552]<br>Expected: Natrinema [taxid 88723] (genus)<br>Number of reads: 3485<br>Number of identified reads: 3462 (99.34%)	• **genus: 1624 (46.599%)**<br>• family: 558 (16.011%)<br>• order: 0 (0.0%)<br>• class: 270 (7.747%)<br>• phylum: 4 (0.114%)<br>• superkingdom: 2 (0.057%)<br>• root: 953 (27.345%)	• Natrinema gari [taxid 419186]: 17 (0.487%)<br>• Natrinema altunense [taxid 222984]: 8 (0.229%)<br>• Natrinema pallidum [taxid 69527]: 7 (0.2%)<br>• Natrinema versiforme [taxid 88724]: 4 (0.114%)<br>• Natrinema salaciae [taxid 1186196]: 3 (0.086%)<br>• Natrinema pellirubrum [taxid 69525]: 2 (0.057%)<br>• Natronococcus jeotgali [taxid 413812]: 2 (0.057%)<br>• other: 18 (0.516%)
Benchmark OTU ID: `CP003377-_Euryarchaeota`<br>OTU taxon: Natronobacterium gregoryi SP2 [taxid 797304]<br>Expected: Natronobacterium gregoryi [taxid 44930] (species)<br>Number of reads: 3581<br>Number of identified reads: 3568 (99.636%)	• **species: 1677 (46.83%)**<br>• genus: 86 (2.401%)<br>• family: 477 (13.32%)<br>• order: 0 (0.0%)<br>• class: 272 (7.595%)<br>• phylum: 2 (0.055%)<br>• superkingdom: 1 (0.027%)<br>• root: 989 (27.617%)	• Natrarchaeobius chitinivorans [taxid 1679083]: 2 (0.055%)<br>• Halobiforma nitratireducens [taxid 130048]: 2 (0.055%)<br>• Natronorubrum tibetense [taxid 63128]: 2 (0.055%)<br>• Haloarcula sinaiiensis [taxid 35742]: 1 (0.027%)<br>• Neolamprologus brichardi [taxid 32507]: 1 (0.027%)<br>• Natrarchaeobaculum sulfurireducens [taxid 2044521]: 1 (0.027%)<br>• Halorubrum aquaticum [taxid 387340]: 1 (0.027%)<br>• Natronorubrum aibiense [taxid 348826]: 1 (0.027%)<br>• other: 15 (0.418%)
Benchmark OTU ID: `CR936257-_Euryarchaeota`<br>OTU taxon: Natronomonas pharaonis DSM 2160 [taxid 348780]<br>Expected: Natronomonas pharaonis [taxid 2257] (species)<br>Number of reads: 2328<br>Number of identified reads: 2319 (99.613%)	• **species: 1257 (53.994%)**<br>• genus: 34 (1.46%)<br>• family: 12 (0.515%)<br>• order: 98 (4.209%)<br>• class: 183 (7.86%)<br>• phylum: 1 (0.042%)<br>• superkingdom: 3 (0.128%)<br>• root: 691 (29.682%)	• Oryza glumipatula [taxid 40148]: 1 (0.042%)<br>• Halogeometricum pallidum [taxid 411361]: 1 (0.042%)<br>• Cyclospora cayetanensis [taxid 88456]: 1 (0.042%)<br>• Oidiodendron maius [taxid 78148]: 1 (0.042%)<br>• Novipirellula galeiformis [taxid 2528004]: 1 (0.042%)<br>• Hortaea thailandica [taxid 706561]: 1 (0.042%)
Benchmark OTU ID: `CP001279-_Proteobacteria`<br>OTU taxon: Nautilia profundicola AmH [taxid 598659]<br>Expected: Nautilia profundicola [taxid 244787] (species)<br>Number of reads: 2888<br>Number of identified reads: 2847 (98.58%)	• **species: 1699 (58.829%)**<br>• genus: 282 (9.764%)<br>• family: 158 (5.47%)<br>• order: 3 (0.103%)<br>• class: 198 (6.855%)<br>• phylum: 67 (2.319%)<br>• superkingdom: 145 (5.02%)<br>• root: 293 (10.145%)	• Helicobacter pylori [taxid 210]: 3 (0.103%)<br>• Cetia pacifica [taxid 1424653]: 3 (0.103%)<br>• Lebetimonas natsushimae [taxid 1936991]: 2 (0.069%)<br>• Caminibacter mediatlanticus [taxid 291048]: 2 (0.069%)<br>• Microbispora hainanensis [taxid 568844]: 1 (0.034%)<br>• Enterococcus casseliflavus [taxid 37734]: 1 (0.034%)<br>• Phorcysia thermohydrogeniphila [taxid 936138]: 1 (0.034%)<br>• Limosilactobacillus fermentum [taxid 1613]: 1 (0.034%)<br>• other: 3 (0.103%)

Operational Taxonomic Unit (OTU)	Correct identifications	Wrong or overspecific identifications at species rank
Benchmark OTU ID: `CP002440-_Pathogens`<br>OTU taxon: Neisseria gonorrhoeae TCDC-NG08107 [taxid 940296]<br>Expected: Neisseria gonorrhoeae [taxid 485] (species)<br>Number of reads: 4173<br>Number of identified reads: 4157 (99.616%)	• species: 1172 (28.085%)<br>• **genus: 1954 (46.824%)**<br>• family: 173 (4.145%)<br>• order: 8 (0.191%)<br>• class: 59 (1.413%)<br>• phylum: 253 (6.062%)<br>• superkingdom: 133 (3.187%)<br>• root: 400 (9.585%)	• **Neisseria meningitidis [taxid 487]: 90 (2.156%)**<br>• Neisseria lactamica [taxid 486]: 17 (0.407%)<br>• Neisseria polysaccharea [taxid 489]: 11 (0.263%)<br>• Neisseria cinerea [taxid 483]: 4 (0.095%)<br>• Neisseria animalis [taxid 492]: 2 (0.047%)<br>• Alcanivorax dieselolei [taxid 285091]: 1 (0.023%)<br>• Simonsiella muelleri [taxid 72]: 1 (0.023%)<br>• Suttonella indologenes [taxid 13276]: 1 (0.023%)<br>• other: 14 (0.335%)
Benchmark OTU ID: `FM999788-_Pathogens`<br>OTU taxon: Neisseria meningitidis 8013 [taxid 604162]<br>Expected: Neisseria meningitidis [taxid 487] (species)<br>Number of reads: 4480<br>Number of identified reads: 4463 (99.62%)	• species: 1172 (26.16%)<br>• **genus: 2256 (50.357%)**<br>• family: 173 (3.861%)<br>• order: 13 (0.29%)<br>• class: 59 (1.316%)<br>• phylum: 260 (5.803%)<br>• superkingdom: 165 (3.683%)<br>• root: 363 (8.102%)	• Neisseria gonorrhoeae [taxid 485]: 57 (1.272%)<br>• Neisseria lactamica [taxid 486]: 29 (0.647%)<br>• Neisseria polysaccharea [taxid 489]: 18 (0.401%)<br>• Neisseria cinerea [taxid 483]: 7 (0.156%)<br>• Neisseria flavescens [taxid 484]: 6 (0.133%)<br>• Clostridioides difficile [taxid 1496]: 4 (0.089%)<br>• Neisseria mucosa [taxid 488]: 2 (0.044%)<br>• Neisseria animaloris [taxid 326522]: 2 (0.044%)<br>• other: 15 (0.334%)
Benchmark OTU ID: `AM421808-_Pathogens`<br>OTU taxon: Neisseria meningitidis FAM18 [taxid 272831]<br>Expected: Neisseria meningitidis [taxid 487] (species)<br>Number of reads: 4273<br>Number of identified reads: 4250 (99.461%)	• species: 1173 (27.451%)<br>• **genus: 2065 (48.326%)**<br>• family: 189 (4.423%)<br>• order: 15 (0.351%)<br>• class: 59 (1.38%)<br>• phylum: 229 (5.359%)<br>• superkingdom: 137 (3.206%)<br>• root: 378 (8.846%)	• Neisseria gonorrhoeae [taxid 485]: 43 (1.006%)<br>• Neisseria lactamica [taxid 486]: 17 (0.397%)<br>• Neisseria polysaccharea [taxid 489]: 12 (0.28%)<br>• Clostridioides difficile [taxid 1496]: 3 (0.07%)<br>• Neisseria weaveri [taxid 28091]: 3 (0.07%)<br>• Neisseria flavescens [taxid 484]: 3 (0.07%)<br>• Neisseria sicca [taxid 490]: 2 (0.046%)<br>• Neisseria elongata [taxid 495]: 2 (0.046%)<br>• other: 28 (0.655%)
Benchmark OTU ID: `CP002419-_Pathogens`<br>OTU taxon: Neisseria meningitidis G2136 [taxid 935599]<br>Expected: Neisseria meningitidis [taxid 487] (species)<br>Number of reads: 4248<br>Number of identified reads: 4235 (99.693%)	• species: 1102 (25.941%)<br>• **genus: 2092 (49.246%)**<br>• family: 167 (3.931%)<br>• order: 16 (0.376%)<br>• class: 55 (1.294%)<br>• phylum: 264 (6.214%)<br>• superkingdom: 150 (3.531%)<br>• root: 388 (9.133%)	• Neisseria gonorrhoeae [taxid 485]: 47 (1.106%)<br>• Neisseria lactamica [taxid 486]: 15 (0.353%)<br>• Neisseria polysaccharea [taxid 489]: 10 (0.235%)<br>• Neisseria sicca [taxid 490]: 8 (0.188%)<br>• Neisseria cinerea [taxid 483]: 6 (0.141%)<br>• Clostridioides difficile [taxid 1496]: 5 (0.117%)<br>• Neisseria flavescens [taxid 484]: 3 (0.07%)<br>• Neisseria arctica [taxid 1470200]: 3 (0.07%)<br>• other: 20 (0.47%)

Operational Taxonomic Unit (OTU)	Correct identifications	Wrong or overspecific identifications at species rank
Benchmark OTU ID: `CP001561-_Pathogens`<br>OTU taxon: Neisseria meningitidis alpha710 [taxid 630588]<br>Expected: Neisseria meningitidis [taxid 487] (species)<br>Number of reads: 4393<br>Number of identified reads: 4379 (99.681%)	• species: 1196 (27.225%)<br>• **genus: 2188 (49.806%)**<br>• family: 198 (4.507%)<br>• order: 15 (0.341%)<br>• class: 62 (1.411%)<br>• phylum: 232 (5.281%)<br>• superkingdom: 142 (3.232%)<br>• root: 345 (7.853%)	• Neisseria gonorrhoeae [taxid 485]: 46 (1.047%)<br>• Neisseria lactamica [taxid 486]: 26 (0.591%)<br>• Neisseria polysaccharea [taxid 489]: 13 (0.295%)<br>• Neisseria flavescens [taxid 484]: 5 (0.113%)<br>• Neisseria sicca [taxid 490]: 5 (0.113%)<br>• Neisseria cinerea [taxid 483]: 5 (0.113%)<br>• Neisseria subflava [taxid 28449]: 2 (0.045%)<br>• Cupriavidus taiwanensis [taxid 164546]: 1 (0.022%)<br>• other: 11 (0.25%)
Benchmark OTU ID: `CP000115-_Proteobacteria`<br>OTU taxon: Nitrobacter winogradskyi Nb-255 [taxid 323098]<br>Expected: Nitrobacter winogradskyi [taxid 913] (species)<br>Number of reads: 6771<br>Number of identified reads: 6744 (99.601%)	• **species: 2910 (42.977%)**<br>• genus: 842 (12.435%)<br>• family: 734 (10.84%)<br>• order: 346 (5.11%)<br>• class: 381 (5.626%)<br>• phylum: 194 (2.865%)<br>• superkingdom: 303 (4.474%)<br>• root: 1029 (15.197%)	• Nitrobacter vulgaris [taxid 29421]: 5 (0.073%)<br>• Nitrobacter hamburgensis [taxid 912]: 4 (0.059%)<br>• Bradyrhizobium elkanii [taxid 29448]: 1 (0.014%)<br>• Afipia felis [taxid 1035]: 1 (0.014%)<br>• Rhodobium orientis [taxid 34017]: 1 (0.014%)<br>• Maricaulis salignorans [taxid 144026]: 1 (0.014%)<br>• Bradyrhizobium erythrophlei [taxid 1437360]: 1 (0.014%)<br>• Blastochloris viridis [taxid 1079]: 1 (0.014%)<br>• other: 24 (0.354%)
Benchmark OTU ID: `CP000127-_Proteobacteria`<br>OTU taxon: Nitrosococcus oceani ATCC 19707 [taxid 323261]<br>Expected: Nitrosococcus oceani [taxid 1229] (species)<br>Number of reads: 6950<br>Number of identified reads: 6878 (98.964%)	• **species: 3605 (51.87%)**<br>• genus: 1021 (14.69%)<br>• family: 42 (0.604%)<br>• order: 12 (0.172%)<br>• class: 342 (4.92%)<br>• phylum: 339 (4.877%)<br>• superkingdom: 362 (5.208%)<br>• root: 1154 (16.604%)	• Nitrosococcus watsonii [taxid 473531]: 22 (0.316%)<br>• Nitrosococcus halophilus [taxid 133539]: 4 (0.057%)<br>• Nitrosococcus wardiae [taxid 1814290]: 3 (0.043%)<br>• Eikenella corrodens [taxid 539]: 1 (0.014%)<br>• Actinoplanes philippinensis [taxid 35752]: 1 (0.014%)<br>• Methylomonas koyamae [taxid 702114]: 1 (0.014%)<br>• Salmonella enterica [taxid 28901]: 1 (0.014%)<br>• Thiobaca trueperi [taxid 127458]: 1 (0.014%)<br>• other: 16 (0.23%)
Benchmark OTU ID: `CP002086-_Proteobacteria`<br>OTU taxon: Nitrosococcus watsonii C-113 [taxid 105559]<br>Expected: Nitrosococcus watsonii [taxid 473531] (species)<br>Number of reads: 6605<br>Number of identified reads: 6559 (99.303%)	• **species: 3346 (50.658%)**<br>• genus: 1066 (16.139%)<br>• family: 35 (0.529%)<br>• order: 14 (0.211%)<br>• class: 295 (4.466%)<br>• phylum: 309 (4.678%)<br>• superkingdom: 371 (5.616%)<br>• root: 1117 (16.911%)	• Nitrosococcus oceani [taxid 1229]: 56 (0.847%)<br>• Nitrosococcus wardiae [taxid 1814290]: 9 (0.136%)<br>• Nitrosococcus halophilus [taxid 133539]: 6 (0.09%)<br>• endosymbiont of Ridgeia piscesae [taxid 54398]: 1 (0.015%)<br>• Posidoniimonas corsicana [taxid 1938618]: 1 (0.015%)<br>• Brugia timori [taxid 42155]: 1 (0.015%)<br>• Lingulodinium polyedra [taxid 160621]: 1 (0.015%)<br>• Nocardioides psychrotolerans [taxid 1005945]: 1 (0.015%)<br>• other: 15 (0.227%)

Operational Taxonomic Unit (OTU)	Correct identifications	Wrong or overspecific identifications at species rank
Benchmark OTU ID: `AL954747-_Proteobacteria`<br>OTU taxon: Nitrosomonas europaea ATCC 19718 [taxid 228410]<br>Expected: Nitrosomonas europaea [taxid 915] (species)<br>Number of reads: 5443<br>Number of identified reads: 5434 (99.834%)	• **species: 3246 (59.636%)**<br>• genus: 398 (7.312%)<br>• family: 43 (0.79%)<br>• order: 61 (1.12%)<br>• class: 193 (3.545%)<br>• phylum: 565 (10.38%)<br>• superkingdom: 217 (3.986%)<br>• root: 708 (13.007%)	• Nitrosomonas eutropha [taxid 916]: 8 (0.146%)<br>• Pseudomonas oleovorans [taxid 301]: 3 (0.055%)<br>• Nitrosomonas mobilis [taxid 51642]: 2 (0.036%)<br>• Tistlia consotensis [taxid 1321365]: 1 (0.018%)<br>• Caballeronia temeraria [taxid 1777137]: 1 (0.018%)<br>• Cupriavidus pauculus [taxid 82633]: 1 (0.018%)<br>• Candidatus Desulfofervidus auxilii [taxid 1621989]: 1 (0.018%)<br>• Nitrosomonas communis [taxid 44574]: 1 (0.018%)<br>• other: 16 (0.293%)
Benchmark OTU ID: `CP000450-_Proteobacteria`<br>OTU taxon: Nitrosomonas eutropha C91 [taxid 335283]<br>Expected: Nitrosomonas eutropha [taxid 916] (species)<br>Number of reads: 5103<br>Number of identified reads: 5088 (99.706%)	• **species: 3016 (59.102%)**<br>• genus: 429 (8.406%)<br>• family: 43 (0.842%)<br>• order: 18 (0.352%)<br>• class: 223 (4.369%)<br>• phylum: 501 (9.817%)<br>• superkingdom: 194 (3.801%)<br>• root: 659 (12.913%)	• Nitrosomonas stercoris [taxid 1444684]: 3 (0.058%)<br>• Nitrosomonas mobilis [taxid 51642]: 2 (0.039%)<br>• Shigella dysenteriae [taxid 622]: 2 (0.039%)<br>• Salmonella enterica [taxid 28901]: 2 (0.039%)<br>• Nitrosomonas halophila [taxid 44576]: 2 (0.039%)<br>• Nitrosomonas ureae [taxid 44577]: 2 (0.039%)<br>• Francisella endosymbiont of Amblyomma maculatum [taxid 255919]: 1 (0.019%)<br>• Falsochrobactrum ovis [taxid 1293442]: 1 (0.019%)<br>• other: 20 (0.391%)
Benchmark OTU ID: `CP002552-_Proteobacteria`<br>OTU taxon: Nitrosomonas sp. AL212 [taxid 153948]<br>Expected: Nitrosomonas [taxid 914] (genus)<br>Number of reads: 6272<br>Number of identified reads: 6234 (99.394%)	• **genus: 4352 (69.387%)**<br>• family: 69 (1.1%)<br>• order: 22 (0.35%)<br>• class: 188 (2.997%)<br>• phylum: 595 (9.486%)<br>• superkingdom: 239 (3.81%)<br>• root: 768 (12.244%)	• **Nitrosomonas ureae [taxid 44577]: 128 (2.04%)**<br>• Nitrosomonas cryotolerans [taxid 44575]: 3 (0.047%)<br>• Nitrosomonas communis [taxid 44574]: 2 (0.031%)<br>• Candidatus Nitrotoga sp. MKT [taxid 2184311]: 2 (0.031%)<br>• Nitrosomonas marina [taxid 917]: 2 (0.031%)<br>• Nitrosomonas oligotropha [taxid 42354]: 2 (0.031%)<br>• Agrobacterium rosae [taxid 1972867]: 1 (0.015%)<br>• other: 18 (0.286%)
Benchmark OTU ID: `CP000103-_Proteobacteria`<br>OTU taxon: Nitrosospira multiformis ATCC 25196 [taxid 323848]<br>Expected: Nitrosospira multiformis [taxid 1231] (species)<br>Number of reads: 6280<br>Number of identified reads: 6267 (99.792%)	• **species: 2646 (42.133%)**<br>• genus: 89 (1.417%)<br>• family: 317 (5.047%)<br>• order: 1208 (19.235%)<br>• class: 259 (4.124%)<br>• phylum: 435 (6.926%)<br>• superkingdom: 239 (3.805%)<br>• root: 1071 (17.054%)	• Candidatus Nitrotoga fabula [taxid 2182327]: 3 (0.047%)<br>• Nitrosovibrio tenuis [taxid 1233]: 3 (0.047%)<br>• Gallionella capsiferriformans [taxid 370405]: 1 (0.015%)<br>• Burkholderia vietnamiensis [taxid 60552]: 1 (0.015%)<br>• Nitrosomonas nitrosa [taxid 52442]: 1 (0.015%)<br>• Citrobacter koseri [taxid 545]: 1 (0.015%)<br>• Legionella birminghamensis [taxid 28083]: 1 (0.015%)<br>• Calditerrivibrio nitroreducens [taxid 477976]: 1 (0.015%)<br>• other: 15 (0.238%)

Operational Taxonomic Unit (OTU)	Correct identifications	Wrong or overspecific identifications at species rank
Benchmark OTU ID: `FP929003-_Nitrospira`<br>OTU taxon: Nitrospira defluvii [taxid 330214]<br>Expected: Nitrospira defluvii [taxid 330214] (species)<br>Number of reads: 41300<br>Number of identified reads: 41142 (99.617%)	• **species: 16523 (40.007%)**<br>• genus: 13158 (31.859%)<br>• family: 65 (0.157%)<br>• order: 11 (0.026%)<br>• class: 2 (0.004%)<br>• phylum: 1314 (3.181%)<br>• superkingdom: 4613 (11.169%)<br>• root: 5421 (13.125%)	• Nitrospira moscoviensis [taxid 42253]: 10 (0.024%)<br>• Candidatus Nitrospira inopinata [taxid 1715989]: 7 (0.016%)<br>• Candidatus Nitrospira nitrificans [taxid 1742973]: 5 (0.012%)<br>• Nitrospira japonica [taxid 1325564]: 5 (0.012%)<br>• Tepidicella xavieri [taxid 360241]: 2 (0.004%)<br>• Stenotrophomonas maltophilia [taxid 40324]: 2 (0.004%)<br>• Serpentinomonas mccroryi [taxid 1458426]: 2 (0.004%)<br>• Salmonella enterica [taxid 28901]: 2 (0.004%)<br>• other: 84 (0.203%)
Benchmark OTU ID: `AP006618-_Actinobacteria`<br>OTU taxon: Nocardia farcinica IFM 10152 [taxid 247156]<br>Expected: Nocardia farcinica [taxid 37329] (species)<br>Number of reads: 27101<br>Number of identified reads: 27011 (99.667%)	• species: 4241 (15.648%)<br>• **genus: 14728 (54.344%)**<br>• family: 789 (2.911%)<br>• order: 1250 (4.612%)<br>• class: 2111 (7.789%)<br>• phylum: 20 (0.073%)<br>• superkingdom: 1624 (5.992%)<br>• root: 2227 (8.217%)	• Nocardia puris [taxid 208602]: 19 (0.07%)<br>• Nocardia cyriacigeorgica [taxid 135487]: 19 (0.07%)<br>• Nocardia arthritidis [taxid 228602]: 13 (0.047%)<br>• Nocardia stercoris [taxid 2483361]: 13 (0.047%)<br>• Nocardia pseudobrasiliensis [taxid 45979]: 11 (0.04%)<br>• Nocardia terpenica [taxid 455432]: 9 (0.033%)<br>• Nocardia panacis [taxid 2340916]: 9 (0.033%)<br>• Nocardia donostiensis [taxid 1538463]: 7 (0.025%)<br>• other: 161 (0.594%)
Benchmark OTU ID: `CP000509-_Actinobacteria`<br>OTU taxon: Nocardioides sp. JS614 [taxid 196162]<br>Expected: Nocardioides [taxid 1839] (genus)<br>Number of reads: 22027<br>Number of identified reads: 21936 (99.586%)	• **genus: 15412 (69.968%)**<br>• family: 451 (2.047%)<br>• order: 118 (0.535%)<br>• class: 2511 (11.399%)<br>• phylum: 37 (0.167%)<br>• superkingdom: 1625 (7.377%)<br>• root: 1771 (8.04%)	• Nocardioides szechwanensis [taxid 1005944]: 8 (0.036%)<br>• Nocardioides anomalus [taxid 2712223]: 6 (0.027%)<br>• Nocardioides euryhalodurans [taxid 2518370]: 6 (0.027%)<br>• Nocardioides guangzhouensis [taxid 2497878]: 5 (0.022%)<br>• Nocardioides baekrokdamisoli [taxid 1804624]: 5 (0.022%)<br>• Nocardioides jejuensis [taxid 2502782]: 4 (0.018%)<br>• Nocardioides gansuensis [taxid 2138300]: 4 (0.018%)<br>• other: 121 (0.549%)
Benchmark OTU ID: `CP003788-_Actinobacteria`<br>OTU taxon: Nocardiopsis alba ATCC BAA-2165 [taxid 1205910]<br>Expected: Nocardiopsis alba [taxid 53437] (species)<br>Number of reads: 26253<br>Number of identified reads: 26011 (99.078%)	• **species: 15493 (59.014%)**<br>• genus: 2037 (7.759%)<br>• family: 464 (1.767%)<br>• order: 216 (0.822%)<br>• class: 3301 (12.573%)<br>• phylum: 26 (0.099%)<br>• superkingdom: 1624 (6.185%)<br>• root: 2826 (10.764%)	• Streptomonospora alba [taxid 183763]: 7 (0.026%)<br>• Nocardiopsis flavescens [taxid 758803]: 7 (0.026%)<br>• Actinorugispora endophytica [taxid 1605990]: 4 (0.015%)<br>• Buchananella hordeovulneris [taxid 52770]: 4 (0.015%)<br>• Marinactinospora thermotolerans [taxid 531310]: 3 (0.011%)<br>• Haloactinospora alba [taxid 405555]: 3 (0.011%)<br>• Pseudoflavonifractor capillosus [taxid 106588]: 2 (0.007%)<br>• Propioniferax innocua [taxid 1753]: 2 (0.007%)<br>• other: 93 (0.354%)

Operational Taxonomic Unit (OTU)	Correct identifications	Wrong or overspecific identifications at species rank
Benchmark OTU ID: `CP001037-_Cyanobacteria`<br>OTU taxon: Nostoc punctiforme PCC 73102 [taxid 63737]<br>Expected: Nostoc punctiforme [taxid 272131] (species)<br>Number of reads: 50486<br>Number of identified reads: 49825 (98.69%)	• species: 13330 (26.403%)<br>• **genus: 16355 (32.395%)**<br>• family: 774 (1.533%)<br>• order: 5490 (10.874%)<br>• phylum: 3302 (6.54%)<br>• superkingdom: 2756 (5.458%)<br>• root: 7700 (15.251%)	• Nostoc flagelliforme [taxid 1306274]: 105 (0.207%)<br>• Nostoc sphaeroides [taxid 446679]: 82 (0.162%)<br>• Nostoc commune [taxid 1178]: 41 (0.081%)<br>• Nostoc linckia [taxid 92942]: 39 (0.077%)<br>• Nostoc calcicola [taxid 212354]: 30 (0.059%)<br>• Nostoc minutum [taxid 1841509]: 29 (0.057%)<br>• Nostoc carneum [taxid 374162]: 9 (0.017%)<br>• other: 201 (0.398%)
Benchmark OTU ID: `CP003548-_Cyanobacteria`<br>OTU taxon: Nostoc sp. PCC 7107 [taxid 317936]<br>Expected: Nostoc [taxid 1177] (genus)<br>Number of reads: 37917<br>Number of identified reads: 37438 (98.736%)	• **genus: 17157 (45.248%)**<br>• family: 466 (1.229%)<br>• order: 10324 (27.227%)<br>• phylum: 2744 (7.236%)<br>• superkingdom: 1796 (4.736%)<br>• root: 4879 (12.867%)	• Nostoc cycadae [taxid 246795]: 45 (0.118%)<br>• Nostoc piscinale [taxid 224012]: 28 (0.073%)<br>• Fischerella thermalis [taxid 372787]: 21 (0.055%)<br>• Nostoc linckia [taxid 92942]: 19 (0.05%)<br>• Nostoc minutum [taxid 1841509]: 18 (0.047%)<br>• Mastigocoleus testarum [taxid 996925]: 16 (0.042%)<br>• other: 252 (0.664%)
Benchmark OTU ID: `BA000019-_Cyanobacteria`<br>OTU taxon: Nostoc sp. PCC 7120 = FACHB-418 [taxid 103690]<br>Expected: Nostoc [taxid 1177] (genus)<br>Number of reads: 38470<br>Number of identified reads: 37979 (98.723%)	• genus: 832 (2.162%)<br>• **family: 23072 (59.974%)**<br>• order: 4091 (10.634%)<br>• phylum: 2988 (7.767%)<br>• superkingdom: 1852 (4.814%)<br>• root: 5070 (13.179%)	• Trichormus variabilis [taxid 264691]: 381 (0.99%)<br>• Nostoc linckia [taxid 92942]: 17 (0.044%)<br>• Trichormus azollae [taxid 1164]: 16 (0.041%)<br>• Nostoc minutum [taxid 1841509]: 16 (0.041%)<br>• Nostoc punctiforme [taxid 272131]: 10 (0.025%)<br>• Anabaena cylindrica [taxid 1165]: 10 (0.025%)<br>• other: 186 (0.483%)
Benchmark OTU ID: `CP003552-_Cyanobacteria`<br>OTU taxon: Nostoc sp. PCC 7524 [taxid 28072]<br>Expected: Nostoc [taxid 1177] (genus)<br>Number of reads: 39931<br>Number of identified reads: 39425 (98.732%)	• **genus: 23402 (58.606%)**<br>• family: 934 (2.339%)<br>• order: 4334 (10.853%)<br>• phylum: 3140 (7.863%)<br>• superkingdom: 2140 (5.359%)<br>• root: 5383 (13.48%)	• Nostoc minutum [taxid 1841509]: 18 (0.045%)<br>• Nostoc linckia [taxid 92942]: 17 (0.042%)<br>• Nostoc flagelliforme [taxid 1306274]: 16 (0.04%)<br>• Fischerella thermalis [taxid 372787]: 13 (0.032%)<br>• Nostoc sphaeroides [taxid 446679]: 12 (0.03%)<br>• Nostoc calcicola [taxid 212354]: 11 (0.027%)<br>• other: 223 (0.558%)
Benchmark OTU ID: `FR856861-_Proteobacteria`<br>OTU taxon: Novosphingobium sp. PP1Y [taxid 702113]<br>Expected: Novosphingobium [taxid 165696] (genus)<br>Number of reads: 1730<br>Number of identified reads: 1728 (99.884%)	• **genus: 1071 (61.907%)**<br>• family: 83 (4.797%)<br>• order: 41 (2.369%)<br>• class: 189 (10.924%)<br>• phylum: 73 (4.219%)<br>• superkingdom: 62 (3.583%)<br>• root: 209 (12.08%)	• Novosphingobium pentaromativorans [taxid 205844]: 6 (0.346%)<br>• Novosphingobium lindaniclasticum [taxid 1329895]: 2 (0.115%)<br>• Paludifilum halophilum [taxid 1642702]: 1 (0.057%)<br>• Novosphingobium sp. PCY [taxid 1248720]: 1 (0.057%)<br>• Caulobacter segnis [taxid 88688]: 1 (0.057%)<br>• Novosphingobium tardaugens [taxid 169176]: 1 (0.057%)<br>• Novosphingobium aromaticivorans [taxid 48935]: 1 (0.057%)<br>• other: 2 (0.115%)

Operational Taxonomic Unit (OTU)	Correct identifications	Wrong or overspecific identifications at species rank
Benchmark OTU ID: `FR856862-_Proteobacteria`<br>OTU taxon: Novosphingobium sp. PP1Y [taxid 702113]<br>Expected: Novosphingobium [taxid 165696] (genus)<br>Number of reads: 7917<br>Number of identified reads: 7892 (99.684%)	• **genus: 5038 (63.635%)**<br>• family: 533 (6.732%)<br>• order: 603 (7.616%)<br>• class: 300 (3.789%)<br>• phylum: 189 (2.387%)<br>• superkingdom: 320 (4.041%)<br>• root: 895 (11.304%)	• Novosphingobium pentaromativorans [taxid 205844]: 105 (1.326%)<br>• Novosphingobium mathurense [taxid 428990]: 11 (0.138%)<br>• Novosphingobium malaysiense [taxid 1348853]: 4 (0.05%)<br>• Sphingobium japonicum [taxid 332056]: 2 (0.025%)<br>• Lupinus albus [taxid 3870]: 2 (0.025%)<br>• Novosphingobium nitrogenifigens [taxid 378548]: 2 (0.025%)<br>• Novosphingobium sediminis [taxid 707214]: 2 (0.025%)<br>• other: 38 (0.479%)
Benchmark OTU ID: `CP003171-_Proteobacteria`<br>OTU taxon: Oceanimonas sp. GK1 [taxid 511062]<br>Expected: Oceanimonas [taxid 129577] (genus)<br>Number of reads: 7024<br>Number of identified reads: 7001 (99.672%)	• **genus: 3975 (56.591%)**<br>• family: 781 (11.119%)<br>• order: 0 (0.0%)<br>• class: 995 (14.165%)<br>• phylum: 303 (4.313%)<br>• superkingdom: 273 (3.886%)<br>• root: 669 (9.524%)	• Oceanimonas baumannii [taxid 129578]: 20 (0.284%)<br>• Oceanimonas doudoroffii [taxid 84158]: 12 (0.17%)<br>• Zobellella endophytica [taxid 2116700]: 8 (0.113%)<br>• Escherichia coli [taxid 562]: 3 (0.042%)<br>• Salmonella enterica [taxid 28901]: 2 (0.028%)<br>• Zobellella denitrificans [taxid 347534]: 2 (0.028%)<br>• Oceanisphaera psychrotolerans [taxid 1414654]: 2 (0.028%)<br>• other: 38 (0.541%)
Benchmark OTU ID: `CP000758-_Proteobacteria`<br>OTU taxon: Ochrobactrum anthropi ATCC 49188 [taxid 439375]<br>Expected: Ochrobactrum anthropi [taxid 529] (species)<br>Number of reads: 5612<br>Number of identified reads: 5603 (99.839%)	• species: 525 (9.354%)<br>• **genus: 1665 (29.668%)**<br>• family: 1520 (27.084%)<br>• order: 712 (12.687%)<br>• class: 172 (3.064%)<br>• phylum: 126 (2.245%)<br>• superkingdom: 199 (3.545%)<br>• root: 677 (12.063%)	• Ochrobactrum lupini [taxid 255457]: 55 (0.98%)<br>• Brucella intermedia [taxid 94625]: 18 (0.32%)<br>• Ochrobactrum oryzae [taxid 335286]: 7 (0.124%)<br>• Ochrobactrum soli [taxid 2448455]: 4 (0.071%)<br>• Ochrobactrum pecoris [taxid 867683]: 4 (0.071%)<br>• Falsochrobactrum shanghaiense [taxid 2201899]: 4 (0.071%)<br>• Ochrobactrum rhizosphaerae [taxid 571254]: 3 (0.053%)<br>• Ochrobactrum tritici [taxid 94626]: 3 (0.053%)<br>• other: 42 (0.748%)
Benchmark OTU ID: `CP000759-_Proteobacteria`<br>OTU taxon: Ochrobactrum anthropi ATCC 49188 [taxid 439375]<br>Expected: Ochrobactrum anthropi [taxid 529] (species)<br>Number of reads: 3382<br>Number of identified reads: 3377 (99.852%)	• species: 268 (7.924%)<br>• **genus: 1121 (33.146%)**<br>• family: 781 (23.092%)<br>• order: 347 (10.26%)<br>• class: 250 (7.392%)<br>• phylum: 104 (3.075%)<br>• superkingdom: 119 (3.518%)<br>• root: 384 (11.354%)	• Ochrobactrum lupini [taxid 255457]: 17 (0.502%)<br>• Ochrobactrum cytisi [taxid 407152]: 6 (0.177%)<br>• Brucella intermedia [taxid 94625]: 4 (0.118%)<br>• Ochrobactrum thiophenivorans [taxid 571255]: 3 (0.088%)<br>• Ochrobactrum pseudintermedium [taxid 370111]: 3 (0.088%)<br>• Ochrobactrum oryzae [taxid 335286]: 2 (0.059%)<br>• Ochrobactrum haematophilum [taxid 419474]: 2 (0.059%)<br>• Lupinus albus [taxid 3870]: 2 (0.059%)<br>• other: 27 (0.798%)

Operational Taxonomic Unit (OTU)	Correct identifications	Wrong or overspecific identifications at species rank
Benchmark OTU ID: `CP003740-_Proteobacteria`<br>OTU taxon: Octadecabacter antarcticus 307 [taxid 391626]<br>Expected: Octadecabacter antarcticus [taxid 1217908] (species)<br>Number of reads: 9944<br>Number of identified reads: 9900 (99.557%)	• **species: 4922 (49.497%)**<br>• genus: 664 (6.677%)<br>• family: 1643 (16.522%)<br>• order: 35 (0.351%)<br>• class: 385 (3.871%)<br>• phylum: 252 (2.534%)<br>• superkingdom: 352 (3.539%)<br>• root: 1639 (16.482%)	• Octadecabacter arcticus [taxid 53946]: 36 (0.362%)<br>• Lentibacter algarum [taxid 576131]: 4 (0.04%)<br>• Cognatiyoonia sediminum [taxid 1508389]: 3 (0.03%)<br>• Pseudorhodobacter antarcticus [taxid 1077947]: 3 (0.03%)<br>• Halocynthiibacter arcticus [taxid 1579316]: 3 (0.03%)<br>• Sulfitobacter guttiformis [taxid 74349]: 3 (0.03%)<br>• Rhodovulum sulfidophilum [taxid 35806]: 3 (0.03%)<br>• Octadecabacter ascidiaceicola [taxid 1655543]: 2 (0.02%)<br>• other: 59 (0.593%)
Benchmark OTU ID: `CP003742-_Proteobacteria`<br>OTU taxon: Octadecabacter arcticus 238 [taxid 391616]<br>Expected: Octadecabacter arcticus [taxid 53946] (species)<br>Number of reads: 10816<br>Number of identified reads: 10778 (99.648%)	• **species: 5219 (48.252%)**<br>• genus: 783 (7.239%)<br>• family: 1812 (16.752%)<br>• order: 29 (0.268%)<br>• class: 447 (4.132%)<br>• phylum: 281 (2.598%)<br>• superkingdom: 358 (3.309%)<br>• root: 1844 (17.048%)	• Octadecabacter antarcticus [taxid 1217908]: 73 (0.674%)<br>• Octadecabacter temperatus [taxid 1458307]: 4 (0.036%)<br>• Halocynthiibacter arcticus [taxid 1579316]: 3 (0.027%)<br>• Flavimaricola marinus [taxid 1819565]: 2 (0.018%)<br>• Pseudooctadecabacter jejudonensis [taxid 1391910]: 2 (0.018%)<br>• Litoreibacter ponti [taxid 1510457]: 2 (0.018%)<br>• Planktotalea frisia [taxid 696762]: 2 (0.018%)<br>• Octadecabacter ascidiaceicola [taxid 1655543]: 2 (0.018%)<br>• other: 83 (0.767%)
Benchmark OTU ID: `CP002544-_Bacteroidetes`<br>OTU taxon: Odoribacter splanchnicus DSM 20712 [taxid 709991]<br>Expected: Odoribacter splanchnicus [taxid 28118] (species)<br>Number of reads: 29570<br>Number of identified reads: 29406 (99.445%)	• species: 7950 (26.885%)<br>• **genus: 12870 (43.523%)**<br>• family: 208 (0.703%)<br>• order: 2674 (9.042%)<br>• class: 118 (0.399%)<br>• phylum: 785 (2.654%)<br>• superkingdom: 1372 (4.639%)<br>• root: 3419 (11.562%)	• Bacteroides fragilis [taxid 817]: 24 (0.081%)<br>• Phocaeicola vulgatus [taxid 821]: 18 (0.06%)<br>• Odoribacter laneus [taxid 626933]: 14 (0.047%)<br>• Bacteroides stercoris [taxid 46506]: 10 (0.033%)<br>• Bacteroides pyogenes [taxid 310300]: 6 (0.02%)<br>• Parabacteroides merdae [taxid 46503]: 4 (0.013%)<br>• Alistipes shahii [taxid 328814]: 3 (0.01%)<br>• Bacteroides uniformis [taxid 820]: 3 (0.01%)<br>• other: 72 (0.243%)
Benchmark OTU ID: `CP002106-_Actinobacteria`<br>OTU taxon: Olsenella uli DSM 7084 [taxid 633147]<br>Expected: Olsenella uli [taxid 133926] (species)<br>Number of reads: 7651<br>Number of identified reads: 7579 (99.058%)	• **species: 4974 (65.011%)**<br>• genus: 286 (3.738%)<br>• family: 75 (0.98%)<br>• order: 337 (4.404%)<br>• class: 129 (1.686%)<br>• phylum: 174 (2.274%)<br>• superkingdom: 666 (8.704%)<br>• root: 931 (12.168%)	• Lancefieldella rimae [taxid 1383]: 3 (0.039%)<br>• Slackia exigua [taxid 84109]: 2 (0.026%)<br>• Methylomicrobium album [taxid 39775]: 1 (0.013%)<br>• Janibacter indicus [taxid 857417]: 1 (0.013%)<br>• Collinsella aerofaciens [taxid 74426]: 1 (0.013%)<br>• Borrelia miyamotoi [taxid 47466]: 1 (0.013%)<br>• Campylobacter ureolyticus [taxid 827]: 1 (0.013%)<br>• Lactobacillus hokkaidonensis [taxid 1193095]: 1 (0.013%)<br>• other: 6 (0.078%)

Operational Taxonomic Unit (OTU)	Correct identifications	Wrong or overspecific identifications at species rank
Benchmark OTU ID: `CP001032-_Verrucomicrobia`<br>OTU taxon: Opitutus terrae PB90-1 [taxid 452637]<br>Expected: Opitutus terrae [taxid 107709] (species)<br>Number of reads: 426088<br>Number of identified reads: 424269 (99.573%)	• **species: 317051 (74.409%)**<br>• genus: 1216 (0.285%)<br>• family: 3689 (0.865%)<br>• order: 11 (0.002%)<br>• class: 2974 (0.697%)<br>• phylum: 8416 (1.975%)<br>• superkingdom: 46341 (10.875%)<br>• root: 44176 (10.367%)	• Nibricoccus aquaticus [taxid 2576891]: 23 (0.005%)<br>• Oleiharenicola lentus [taxid 2508720]: 16 (0.003%)<br>• Salmonella enterica [taxid 28901]: 16 (0.003%)<br>• Ereboglobus luteus [taxid 1796921]: 13 (0.003%)<br>• Lacunisphaera limnophila [taxid 1838286]: 13 (0.003%)<br>• Cephaloticoccus capnophilus [taxid 1548208]: 12 (0.002%)<br>• bacterium [taxid 1869227]: 11 (0.002%)<br>• Rariglobus hedericola [taxid 2597822]: 11 (0.002%)<br>• other: 754 (0.176%)
Benchmark OTU ID: `AP008981-_Proteobacteria`<br>OTU taxon: Orientia tsutsugamushi str. Ikeda [taxid 334380]<br>Expected: Orientia tsutsugamushi [taxid 784] (species)<br>Number of reads: 3636<br>Number of identified reads: 3489 (95.957%)	• **species: 2813 (77.365%)**<br>• genus: 58 (1.595%)<br>• family: 15 (0.412%)<br>• order: 3 (0.082%)<br>• class: 15 (0.412%)<br>• phylum: 34 (0.935%)<br>• superkingdom: 69 (1.897%)<br>• root: 477 (13.118%)	• Orientia chuto [taxid 911112]: 1 (0.027%)<br>• Rickettsia endosymbiont of Ixodes scapularis [taxid 444612]: 1 (0.027%)<br>• Fistulifera solaris [taxid 1519565]: 1 (0.027%)<br>• Helicobacter felis [taxid 214]: 1 (0.027%)
Benchmark OTU ID: `CP003283-_Bacteroidetes`<br>OTU taxon: Ornithobacterium rhinotracheale DSM 15997 [taxid 867902]<br>Expected: Ornithobacterium rhinotracheale [taxid 28251] (species)<br>Number of reads: 14422<br>Number of identified reads: 14268 (98.932%)	• **species: 10616 (73.609%)**<br>• genus: 95 (0.658%)<br>• family: 201 (1.393%)<br>• order: 645 (4.472%)<br>• class: 7 (0.048%)<br>• phylum: 601 (4.167%)<br>• superkingdom: 623 (4.319%)<br>• root: 1462 (10.137%)	• Lupinus albus [taxid 3870]: 6 (0.041%)<br>• Candidatus Walczuchella monophlebidarum [taxid 1415657]: 2 (0.013%)<br>• Spinacia oleracea [taxid 3562]: 2 (0.013%)<br>• Moheibacter sediminis [taxid 1434700]: 2 (0.013%)<br>• Saprospira grandis [taxid 1008]: 2 (0.013%)<br>• Pedobacter psychrotolerans [taxid 1843235]: 1 (0.006%)<br>• Segetibacter aerophilus [taxid 670293]: 1 (0.006%)<br>• Jiulongibacter sediminis [taxid 1605367]: 1 (0.006%)<br>• other: 34 (0.235%)
Benchmark OTU ID: `CP003607-_Cyanobacteria`<br>OTU taxon: Oscillatoria acuminata PCC 6304 [taxid 56110]<br>Expected: Oscillatoria acuminata [taxid 118323] (species)<br>Number of reads: 46890<br>Number of identified reads: 46283 (98.705%)	• **species: 31289 (66.728%)**<br>• genus: 4 (0.008%)<br>• family: 145 (0.309%)<br>• order: 754 (1.608%)<br>• phylum: 3255 (6.941%)<br>• superkingdom: 2598 (5.54%)<br>• root: 8124 (17.325%)	• Arthrospira platensis [taxid 118562]: 9 (0.019%)<br>• Limnospira fusiformis [taxid 54297]: 4 (0.008%)<br>• Limnospira indica [taxid 147322]: 3 (0.006%)<br>• Synechococcus elongatus [taxid 32046]: 3 (0.006%)<br>• Microcystis aeruginosa [taxid 1126]: 3 (0.006%)<br>• Acaryochloris marina [taxid 155978]: 3 (0.006%)<br>• Cylindrospermopsis raciborskii [taxid 77022]: 2 (0.004%)<br>• other: 105 (0.223%)

Operational Taxonomic Unit (OTU)	Correct identifications	Wrong or overspecific identifications at species rank
Benchmark OTU ID: `CP003614-_Cyanobacteria`<br>OTU taxon: Oscillatoria nigro-viridis PCC 7112 [taxid 179408]<br>Expected: Oscillatoria nigro-viridis [taxid 482564] (species)<br>Number of reads: 45501<br>Number of identified reads: 45114 (99.149%)	• **species: 19193 (42.181%)**<br>• genus: 16 (0.035%)<br>• family: 153 (0.336%)<br>• order: 7892 (17.344%)<br>• phylum: 6746 (14.826%)<br>• superkingdom: 2832 (6.224%)<br>• root: 8170 (17.955%)	• Microcoleus vaginatus [taxid 119532]: 247 (0.542%)<br>• Tychonema bourrellyi [taxid 54313]: 42 (0.092%)<br>• Oscillatoria acuminata [taxid 118323]: 9 (0.019%)<br>• Chamaesiphon minutus [taxid 1173032]: 7 (0.015%)<br>• Phormidium ambiguum [taxid 71191]: 7 (0.015%)<br>• Scytonema hofmannii [taxid 34078]: 6 (0.013%)<br>• Gloeothece verrucosa [taxid 2546359]: 4 (0.008%)<br>• other: 184 (0.404%)
Benchmark OTU ID: `CP003235-_Firmicutes`<br>OTU taxon: Paenibacillus mucilaginosus 3016 [taxid 1116391]<br>Expected: Paenibacillus mucilaginosus [taxid 61624] (species)<br>Number of reads: 13213<br>Number of identified reads: 13156 (99.568%)	• **species: 9112 (68.962%)**<br>• genus: 842 (6.372%)<br>• family: 172 (1.301%)<br>• order: 367 (2.777%)<br>• class: 22 (0.166%)<br>• phylum: 132 (0.999%)<br>• superkingdom: 867 (6.561%)<br>• root: 1627 (12.313%)	• Carboxydothermus pertinax [taxid 870242]: 3 (0.022%)<br>• Mycobacterium tuberculosis [taxid 1773]: 2 (0.015%)<br>• Paenibacillus zeisoli [taxid 2496267]: 1 (0.007%)<br>• Paenibacillus larvae [taxid 1464]: 1 (0.007%)<br>• Alexandrium monilatum [taxid 311494]: 1 (0.007%)<br>• Acidisphaera rubrifaciens [taxid 50715]: 1 (0.007%)<br>• Tetrahymena thermophila [taxid 5911]: 1 (0.007%)<br>• Paenibacillus popilliae [taxid 78057]: 1 (0.007%)<br>• other: 41 (0.31%)
Benchmark OTU ID: `CP003422-_Firmicutes`<br>OTU taxon: Paenibacillus mucilaginosus K02 [taxid 997761]<br>Expected: Paenibacillus mucilaginosus [taxid 61624] (species)<br>Number of reads: 13263<br>Number of identified reads: 13210 (99.6%)	• **species: 9192 (69.305%)**<br>• genus: 839 (6.325%)<br>• family: 198 (1.492%)<br>• order: 336 (2.533%)<br>• class: 35 (0.263%)<br>• phylum: 132 (0.995%)<br>• superkingdom: 844 (6.363%)<br>• root: 1612 (12.154%)	• Paenibacillus thalictri [taxid 2527873]: 3 (0.022%)<br>• Paenibacillus darwinianus [taxid 1380763]: 3 (0.022%)<br>• Mycobacterium tuberculosis [taxid 1773]: 2 (0.015%)<br>• Lupinus albus [taxid 3870]: 2 (0.015%)<br>• Karenia brevis [taxid 156230]: 1 (0.007%)<br>• Bos indicus x Bos taurus [taxid 30522]: 1 (0.007%)<br>• Paenibacillus borealis [taxid 160799]: 1 (0.007%)<br>• Rickenella mellea [taxid 50990]: 1 (0.007%)<br>• other: 34 (0.256%)
Benchmark OTU ID: `CP000154-_Firmicutes`<br>OTU taxon: Paenibacillus polymyxa E681 [taxid 349520]<br>Expected: Paenibacillus polymyxa [taxid 1406] (species)<br>Number of reads: 7863<br>Number of identified reads: 7812 (99.351%)	• species: 1488 (18.924%)<br>• **genus: 4592 (58.4%)**<br>• family: 88 (1.119%)<br>• order: 209 (2.658%)<br>• class: 35 (0.445%)<br>• phylum: 79 (1.004%)<br>• superkingdom: 395 (5.023%)<br>• root: 921 (11.713%)	• Paenibacillus terrae [taxid 159743]: 17 (0.216%)<br>• Paenibacillus peoriae [taxid 59893]: 7 (0.089%)<br>• Paenibacillus ottowii [taxid 2315729]: 4 (0.05%)<br>• Paenibacillus kribbensis [taxid 172713]: 3 (0.038%)<br>• Paenibacillus zeisoli [taxid 2496267]: 3 (0.038%)<br>• Leptotrichia hofstadii [taxid 157688]: 2 (0.025%)<br>• Paenibacillus faecis [taxid 862114]: 2 (0.025%)<br>• Paenibacillus anaericanus [taxid 170367]: 2 (0.025%)<br>• other: 51 (0.648%)

Operational Taxonomic Unit (OTU)	Correct identifications	Wrong or overspecific identifications at species rank
Benchmark OTU ID: `HE577054-_Firmicutes`<br>OTU taxon: Paenibacillus polymyxa M1 [taxid 1052684]<br>Expected: Paenibacillus polymyxa [taxid 1406] (species)<br>Number of reads: 8614<br>Number of identified reads: 8574 (99.535%)	• species: 1130 (13.118%)<br>• **genus: 5478 (63.594%)**<br>• family: 93 (1.079%)<br>• order: 243 (2.82%)<br>• class: 37 (0.429%)<br>• phylum: 101 (1.172%)<br>• superkingdom: 374 (4.341%)<br>• root: 1107 (12.851%)	• Paenibacillus terrae [taxid 159743]: 14 (0.162%)<br>• Paenibacillus ottowii [taxid 2315729]: 13 (0.15%)<br>• Paenibacillus peoriae [taxid 59893]: 4 (0.046%)<br>• Paenibacillus kribbensis [taxid 172713]: 3 (0.034%)<br>• Mucor ambiguus [taxid 91626]: 2 (0.023%)<br>• Escherichia coli [taxid 562]: 2 (0.023%)<br>• Paenibacillus elgii [taxid 189691]: 2 (0.023%)<br>• Paenibacillus cellulosilyticus [taxid 375489]: 2 (0.023%)<br>• other: 53 (0.615%)
Benchmark OTU ID: `CP002213-_Firmicutes`<br>OTU taxon: Paenibacillus polymyxa SC2 [taxid 886882]<br>Expected: Paenibacillus polymyxa [taxid 1406] (species)<br>Number of reads: 8402<br>Number of identified reads: 8351 (99.393%)	• species: 1090 (12.973%)<br>• **genus: 5387 (64.115%)**<br>• family: 106 (1.261%)<br>• order: 210 (2.499%)<br>• class: 36 (0.428%)<br>• phylum: 108 (1.285%)<br>• superkingdom: 374 (4.451%)<br>• root: 1033 (12.294%)	• Paenibacillus terrae [taxid 159743]: 21 (0.249%)<br>• Paenibacillus ottowii [taxid 2315729]: 10 (0.119%)<br>• Paenibacillus macerans [taxid 44252]: 7 (0.083%)<br>• Paenibacillus alvei [taxid 44250]: 4 (0.047%)<br>• Paenibacillus polysaccharolyticus [taxid 582692]: 3 (0.035%)<br>• Paenibacillus crassostreae [taxid 1763538]: 3 (0.035%)<br>• Paenibacillus zeisoli [taxid 2496267]: 3 (0.035%)<br>• Lupinus albus [taxid 3870]: 3 (0.035%)<br>• other: 52 (0.618%)
Benchmark OTU ID: `CP003107-_Firmicutes`<br>OTU taxon: Paenibacillus terrae HPL-003 [taxid 985665]<br>Expected: Paenibacillus terrae [taxid 159743] (species)<br>Number of reads: 8964<br>Number of identified reads: 8913 (99.431%)	• species: 3292 (36.724%)<br>• **genus: 3562 (39.736%)**<br>• family: 106 (1.182%)<br>• order: 257 (2.867%)<br>• class: 42 (0.468%)<br>• phylum: 91 (1.015%)<br>• superkingdom: 431 (4.808%)<br>• root: 1121 (12.505%)	• Paenibacillus polymyxa [taxid 1406]: 21 (0.234%)<br>• Paenibacillus crassostreae [taxid 1763538]: 4 (0.044%)<br>• Paenibacillus kribbensis [taxid 172713]: 3 (0.033%)<br>• Paenibacillus lutimineralis [taxid 2707005]: 2 (0.022%)<br>• Paenibacillus lautus [taxid 1401]: 2 (0.022%)<br>• Paenibacillus peoriae [taxid 59893]: 2 (0.022%)<br>• Paenibacillus durus [taxid 44251]: 2 (0.022%)<br>• Paenibacillus macerans [taxid 44252]: 2 (0.022%)<br>• other: 41 (0.457%)
Benchmark OTU ID: `Pan_troglo-_Eukaryotes`<br>OTU taxon: Pan troglodytes [taxid 9598]<br>Expected: Pan troglodytes [taxid 9598] (species)<br>Number of reads: 200656<br>Number of identified reads: 175163 (87.295%)	• species: 366 (0.182%)<br>• genus: 280 (0.139%)<br>• family: 1731 (0.862%)<br>• order: 15813 (7.88%)<br>• class: 2063 (1.028%)<br>• phylum: 1226 (0.61%)<br>• superkingdom: 3559 (1.773%)<br>• **root: 146430 (72.975%)**	• Macaca mulatta [taxid 9544]: 1946 (0.969%)<br>• Homo sapiens [taxid 9606]: 520 (0.259%)<br>• Callithrix jacchus [taxid 9483]: 502 (0.25%)<br>• Macaca fascicularis [taxid 9541]: 237 (0.118%)<br>• Pongo abelii [taxid 9601]: 213 (0.106%)<br>• Nomascus leucogenys [taxid 61853]: 140 (0.069%)<br>• Gorilla gorilla [taxid 9593]: 121 (0.06%)<br>• Colobus angolensis [taxid 54131]: 102 (0.05%)<br>• other: 2136 (1.064%)

Operational Taxonomic Unit (OTU)	Correct identifications	Wrong or overspecific identifications at species rank
Benchmark OTU ID: `AP012032-_Proteobacteria`<br>OTU taxon: Pantoea ananatis AJ13355 [taxid 932677]<br>Expected: Pantoea ananatis [taxid 553] (species)<br>Number of reads: 9366<br>Number of identified reads: 9326 (99.572%)	• **species: 2887 (30.824%)**<br>• genus: 1858 (19.837%)<br>• family: 387 (4.131%)<br>• order: 2321 (24.781%)<br>• class: 364 (3.886%)<br>• phylum: 207 (2.21%)<br>• superkingdom: 288 (3.074%)<br>• root: 1009 (10.773%)	• Salmonella enterica [taxid 28901]: 30 (0.32%)<br>• Escherichia coli [taxid 562]: 18 (0.192%)<br>• Pantoea allii [taxid 574096]: 11 (0.117%)<br>• Pantoea stewartii [taxid 66269]: 7 (0.074%)<br>• Pluralibacter gergoviae [taxid 61647]: 4 (0.042%)<br>• Pantoea deleyi [taxid 470932]: 4 (0.042%)<br>• Klebsiella aerogenes [taxid 548]: 3 (0.032%)<br>• Erwinia iniecta [taxid 1560201]: 3 (0.032%)<br>• other: 95 (1.014%)
Benchmark OTU ID: `CP001875-_Proteobacteria`<br>OTU taxon: Pantoea ananatis LMG 20103 [taxid 706191]<br>Expected: Pantoea ananatis [taxid 553] (species)<br>Number of reads: 9698<br>Number of identified reads: 9671 (99.721%)	• **species: 3102 (31.985%)**<br>• genus: 1902 (19.612%)<br>• family: 383 (3.949%)<br>• order: 2329 (24.015%)<br>• class: 307 (3.165%)<br>• phylum: 246 (2.536%)<br>• superkingdom: 296 (3.052%)<br>• root: 1094 (11.28%)	• Salmonella enterica [taxid 28901]: 33 (0.34%)<br>• Escherichia coli [taxid 562]: 20 (0.206%)<br>• Pantoea stewartii [taxid 66269]: 8 (0.082%)<br>• Pantoea allii [taxid 574096]: 7 (0.072%)<br>• Candidatus Fukatsuia symbiotica [taxid 1878942]: 4 (0.041%)<br>• Erwinia gerundensis [taxid 1619313]: 3 (0.03%)<br>• Klebsiella oxytoca [taxid 571]: 3 (0.03%)<br>• Candidatus Regiella insecticola [taxid 138073]: 2 (0.02%)<br>• other: 95 (0.979%)
Benchmark OTU ID: `CP003085-_Proteobacteria`<br>OTU taxon: Pantoea ananatis PA13 [taxid 1095774]<br>Expected: Pantoea ananatis [taxid 553] (species)<br>Number of reads: 9435<br>Number of identified reads: 9398 (99.607%)	• **species: 2490 (26.391%)**<br>• genus: 2028 (21.494%)<br>• family: 352 (3.73%)<br>• order: 2377 (25.193%)<br>• class: 355 (3.762%)<br>• phylum: 239 (2.533%)<br>• superkingdom: 291 (3.084%)<br>• root: 1252 (13.269%)	• Salmonella enterica [taxid 28901]: 43 (0.455%)<br>• Pantoea stewartii [taxid 66269]: 26 (0.275%)<br>• Escherichia coli [taxid 562]: 22 (0.233%)<br>• Pantoea allii [taxid 574096]: 17 (0.18%)<br>• Pectobacterium betavasculorum [taxid 55207]: 6 (0.063%)<br>• Pantoea deleyi [taxid 470932]: 6 (0.063%)<br>• Enterobacter cloacae [taxid 550]: 5 (0.052%)<br>• Pantoea agglomerans [taxid 549]: 5 (0.052%)<br>• other: 130 (1.377%)
Benchmark OTU ID: `CP002433-_Proteobacteria`<br>OTU taxon: Pantoea sp. At-9b [taxid 592316]<br>Expected: Pantoea [taxid 53335] (genus)<br>Number of reads: 8946<br>Number of identified reads: 8926 (99.776%)	• **genus: 3883 (43.404%)**<br>• family: 402 (4.493%)<br>• order: 2981 (33.322%)<br>• class: 367 (4.102%)<br>• phylum: 245 (2.738%)<br>• superkingdom: 272 (3.04%)<br>• root: 766 (8.562%)	• Salmonella enterica [taxid 28901]: 36 (0.402%)<br>• Pantoea cypripedii [taxid 55209]: 25 (0.279%)<br>• Escherichia coli [taxid 562]: 24 (0.268%)<br>• Pantoea ananatis [taxid 553]: 8 (0.089%)<br>• Anopheles aquasalis [taxid 42839]: 5 (0.055%)<br>• Klebsiella pneumoniae [taxid 573]: 5 (0.055%)<br>• Photorhabdus namnaonensis [taxid 1851568]: 5 (0.055%)<br>• other: 124 (1.386%)

Operational Taxonomic Unit (OTU)	Correct identifications	Wrong or overspecific identifications at species rank
Benchmark OTU ID: `CP002206-_Proteobacteria`<br>OTU taxon: Pantoea vagans C9-1 [taxid 712898]<br>Expected: Pantoea vagans [taxid 470934] (species)<br>Number of reads: 8172<br>Number of identified reads: 8150 (99.73%)	• species: 534 (6.534%)<br>• **genus: 3610 (44.175%)**<br>• family: 397 (4.858%)<br>• order: 2021 (24.73%)<br>• class: 348 (4.258%)<br>• phylum: 216 (2.643%)<br>• superkingdom: 236 (2.887%)<br>• root: 781 (9.557%)	• Salmonella enterica [taxid 28901]: 27 (0.33%)<br>• Pantoea ananatis [taxid 553]: 18 (0.22%)<br>• Escherichia coli [taxid 562]: 17 (0.208%)<br>• Pantoea deleyi [taxid 470932]: 15 (0.183%)<br>• Pantoea agglomerans [taxid 549]: 13 (0.159%)<br>• Plautia stali symbiont [taxid 891974]: 5 (0.061%)<br>• Pantoea sp. Eser [taxid 2576758]: 5 (0.061%)<br>• Klebsiella pneumoniae [taxid 573]: 4 (0.048%)<br>• other: 113 (1.382%)
Benchmark OTU ID: `CP000490-_Proteobacteria`<br>OTU taxon: Paracoccus denitrificans PD1222 [taxid 318586]<br>Expected: Paracoccus denitrificans [taxid 266] (species)<br>Number of reads: 3009<br>Number of identified reads: 2999 (99.667%)	• **species: 1035 (34.396%)**<br>• genus: 952 (31.638%)<br>• family: 253 (8.408%)<br>• order: 6 (0.199%)<br>• class: 202 (6.713%)<br>• phylum: 123 (4.087%)<br>• superkingdom: 150 (4.985%)<br>• root: 273 (9.072%)	• Paracoccus versutus [taxid 34007]: 4 (0.132%)<br>• Paracoccus sulfuroxidans [taxid 384678]: 2 (0.066%)<br>• Rhodovulum sulfidophilum [taxid 35806]: 2 (0.066%)<br>• Paracoccus pantotrophus [taxid 82367]: 2 (0.066%)<br>• Paracoccus thiocyanatus [taxid 34006]: 2 (0.066%)<br>• Paracoccus halophilus [taxid 376733]: 2 (0.066%)<br>• Celeribacter indicus [taxid 1208324]: 1 (0.033%)<br>• Lepeophtheirus salmonis [taxid 72036]: 1 (0.033%)<br>• other: 21 (0.697%)
Benchmark OTU ID: `CP002897-_Proteobacteria`<br>OTU taxon: Paracoccus denitrificans SD1 [taxid 1051075]<br>Expected: Paracoccus denitrificans [taxid 266] (species)<br>Number of reads: 5833<br>Number of identified reads: 5805 (99.519%)	• species: 38 (0.651%)<br>• **genus: 2370 (40.63%)**<br>• family: 866 (14.846%)<br>• order: 11 (0.188%)<br>• class: 681 (11.674%)<br>• phylum: 323 (5.537%)<br>• superkingdom: 365 (6.257%)<br>• root: 1138 (19.509%)	• Paracoccus aminovorans [taxid 34004]: 80 (1.371%)<br>• Paracoccus thiocyanatus [taxid 34006]: 74 (1.268%)<br>• Paracoccus sp. DMF [taxid 400837]: 71 (1.217%)<br>• Paracoccus yeei [taxid 147645]: 37 (0.634%)<br>• Paracoccus pantotrophus [taxid 82367]: 33 (0.565%)<br>• Paracoccus versutus [taxid 34007]: 32 (0.548%)<br>• Paracoccus halophilus [taxid 376733]: 28 (0.48%)<br>• Paracoccus aestuariivivens [taxid 1820333]: 25 (0.428%)<br>• other: 241 (4.131%)
Benchmark OTU ID: `CP002835-_Firmicutes`<br>OTU taxon: Parageobacillus thermoglucosidasius C56-YS93 [taxid 634956]<br>Expected: Parageobacillus thermoglucosidasius [taxid 1426] (species)<br>Number of reads: 5460<br>Number of identified reads: 5421 (99.285%)	• species: 529 (9.688%)<br>• genus: 127 (2.326%)<br>• **family: 3385 (61.996%)**<br>• order: 245 (4.487%)<br>• class: 57 (1.043%)<br>• phylum: 65 (1.19%)<br>• superkingdom: 276 (5.054%)<br>• root: 732 (13.406%)	• Parageobacillus thermantarcticus [taxid 186116]: 7 (0.128%)<br>• Parageobacillus caldoxylosilyticus [taxid 81408]: 7 (0.128%)<br>• Bacillus methanolicus [taxid 1471]: 4 (0.073%)<br>• Lupinus albus [taxid 3870]: 4 (0.073%)<br>• Parageobacillus toebii [taxid 153151]: 3 (0.054%)<br>• Caldibacillus debilis [taxid 301148]: 3 (0.054%)<br>• Thermolongibacillus altinsuensis [taxid 575256]: 2 (0.036%)<br>• Anoxybacillus flavithermus [taxid 33934]: 2 (0.036%)<br>• other: 56 (1.025%)

Operational Taxonomic Unit (OTU)	Correct identifications	Wrong or overspecific identifications at species rank
Benchmark OTU ID: `CP003837-_Proteobacteria`<br>OTU taxon: Paraglaciecola psychrophila 170 [taxid 1129794]<br>Expected: Paraglaciecola psychrophila [taxid 326544] (species)<br>Number of reads: 11297<br>Number of identified reads: 11165 (98.831%)	• **species: 6567 (58.13%)**<br>• genus: 1203 (10.648%)<br>• family: 301 (2.664%)<br>• order: 250 (2.212%)<br>• class: 634 (5.612%)<br>• phylum: 302 (2.673%)<br>• superkingdom: 341 (3.018%)<br>• root: 1563 (13.835%)	• Paraglaciecola arctica [taxid 1128911]: 18 (0.159%)<br>• Escherichia coli [taxid 562]: 3 (0.026%)<br>• Paraglaciecola hydrolytica [taxid 1799789]: 3 (0.026%)<br>• Moraxella catarrhalis [taxid 480]: 1 (0.008%)<br>• Pseudidiomarina atlantica [taxid 1517416]: 1 (0.008%)<br>• Chitinophaga flava [taxid 2259036]: 1 (0.008%)<br>• Pasteurella multocida [taxid 747]: 1 (0.008%)<br>• Alcanivorax hongdengensis [taxid 519051]: 1 (0.008%)<br>• other: 28 (0.247%)
Benchmark OTU ID: `CP000774-_Proteobacteria`<br>OTU taxon: Parvibaculum lavamentivorans DS-1 [taxid 402881]<br>Expected: Parvibaculum lavamentivorans [taxid 256618] (species)<br>Number of reads: 7924<br>Number of identified reads: 7909 (99.81%)	• **species: 4282 (54.038%)**<br>• genus: 60 (0.757%)<br>• family: 150 (1.892%)<br>• order: 169 (2.132%)<br>• class: 1365 (17.226%)<br>• phylum: 540 (6.814%)<br>• superkingdom: 386 (4.871%)<br>• root: 949 (11.976%)	• Parvibaculum sedimenti [taxid 2608632]: 3 (0.037%)<br>• Nitrospirillum amazonense [taxid 28077]: 2 (0.025%)<br>• Tepidicaulis marinus [taxid 1333998]: 2 (0.025%)<br>• Novosphingobium kunmingense [taxid 1211806]: 1 (0.012%)<br>• Legionella feeleii [taxid 453]: 1 (0.012%)<br>• Aspergillus thermomutatus [taxid 41047]: 1 (0.012%)<br>• Bartonella schoenbuchensis [taxid 165694]: 1 (0.012%)<br>• Trebonia kvetii [taxid 2480626]: 1 (0.012%)<br>• other: 45 (0.567%)
Benchmark OTU ID: `CP002156-_Proteobacteria`<br>OTU taxon: Parvularcula bermudensis HTCC2503 [taxid 314260]<br>Expected: Parvularcula bermudensis [taxid 208216] (species)<br>Number of reads: 5647<br>Number of identified reads: 5605 (99.256%)	• **species: 3798 (67.256%)**<br>• genus: 41 (0.726%)<br>• family: 11 (0.194%)<br>• order: 0 (0.0%)<br>• class: 489 (8.659%)<br>• phylum: 172 (3.045%)<br>• superkingdom: 270 (4.781%)<br>• root: 819 (14.503%)	• Parvularcula flava [taxid 1566827]: 4 (0.07%)<br>• Hyphomonas polymorpha [taxid 74319]: 2 (0.035%)<br>• Sphingorhabdus lacus [taxid 392610]: 2 (0.035%)<br>• Rhizobium leguminosarum [taxid 384]: 1 (0.017%)<br>• Acidithiobacillus ferrivorans [taxid 160808]: 1 (0.017%)<br>• Marinicaulis flavus [taxid 2058213]: 1 (0.017%)<br>• Ochrobactrum gallinifaecis [taxid 215590]: 1 (0.017%)<br>• Aestuariivirga litoralis [taxid 2650924]: 1 (0.017%)<br>• other: 13 (0.23%)
Benchmark OTU ID: `AE004439-_Proteobacteria`<br>OTU taxon: Pasteurella multocida subsp. multocida str. Pm70 [taxid 272843]<br>Expected: Pasteurella multocida [taxid 747] (species)<br>Number of reads: 4195<br>Number of identified reads: 4173 (99.475%)	• species: 669 (15.947%)<br>• genus: 49 (1.168%)<br>• **family: 2491 (59.38%)**<br>• order: 1 (0.023%)<br>• class: 412 (9.821%)<br>• phylum: 100 (2.383%)<br>• superkingdom: 138 (3.289%)<br>• root: 311 (7.413%)	• Haemophilus influenzae [taxid 727]: 6 (0.143%)<br>• Pasteurella canis [taxid 753]: 4 (0.095%)<br>• Conservatibacter flavescens [taxid 28161]: 3 (0.071%)<br>• Avibacterium paragallinarum [taxid 728]: 3 (0.071%)<br>• Pasteurella langaaensis [taxid 756]: 3 (0.071%)<br>• Escherichia coli [taxid 562]: 3 (0.071%)<br>• Pasteurella dagmatis [taxid 754]: 3 (0.071%)<br>• Bibersteinia trehalosi [taxid 47735]: 2 (0.047%)<br>• other: 43 (1.025%)

Operational Taxonomic Unit (OTU)	Correct identifications	Wrong or overspecific identifications at species rank
Benchmark OTU ID: `BX950851-_Proteobacteria`<br>OTU taxon: Pectobacterium atrosepticum SCRI1043 [taxid 218491]<br>Expected: Pectobacterium atrosepticum [taxid 29471] (species)<br>Number of reads: 10510<br>Number of identified reads: 10476 (99.676%)	• species: 1970 (18.744%)<br>• **genus: 3511 (33.406%)**<br>• family: 925 (8.801%)<br>• order: 1940 (18.458%)<br>• class: 423 (4.024%)<br>• phylum: 241 (2.293%)<br>• superkingdom: 330 (3.139%)<br>• root: 1132 (10.77%)	• Pectobacterium carotovorum [taxid 554]: 22 (0.209%)<br>• Salmonella enterica [taxid 28901]: 21 (0.199%)<br>• Pectobacterium parmentieri [taxid 1905730]: 16 (0.152%)<br>• Escherichia coli [taxid 562]: 16 (0.152%)<br>• Pectobacterium betavasculorum [taxid 55207]: 12 (0.114%)<br>• Pectobacterium polaris [taxid 2042057]: 11 (0.104%)<br>• Pectobacterium peruviense [taxid 2066479]: 10 (0.095%)<br>• Pectobacterium brasiliense [taxid 180957]: 9 (0.085%)<br>• other: 103 (0.98%)
Benchmark OTU ID: `CP001657-_Proteobacteria`<br>OTU taxon: Pectobacterium carotovorum subsp. carotovorum PC1 [taxid 561230]<br>Expected: Pectobacterium carotovorum [taxid 554] (species)<br>Number of reads: 10058<br>Number of identified reads: 10030 (99.721%)	• species: 1984 (19.725%)<br>• **genus: 3117 (30.99%)**<br>• family: 1051 (10.449%)<br>• order: 1833 (18.224%)<br>• class: 389 (3.867%)<br>• phylum: 257 (2.555%)<br>• superkingdom: 321 (3.191%)<br>• root: 1069 (10.628%)	• Salmonella enterica [taxid 28901]: 23 (0.228%)<br>• Escherichia coli [taxid 562]: 19 (0.188%)<br>• Pectobacterium parmentieri [taxid 1905730]: 12 (0.119%)<br>• Pectobacterium brasiliense [taxid 180957]: 12 (0.119%)<br>• Pectobacterium polaris [taxid 2042057]: 12 (0.119%)<br>• Pectobacterium actinidiae [taxid 1507808]: 6 (0.059%)<br>• Pectobacterium odoriferum [taxid 78398]: 6 (0.059%)<br>• Samsonia erythrinae [taxid 160434]: 5 (0.049%)<br>• other: 120 (1.193%)
Benchmark OTU ID: `CP003776-_Proteobacteria`<br>OTU taxon: Pectobacterium carotovorum subsp. carotovorum PCC21 [taxid 1218933]<br>Expected: Pectobacterium carotovorum [taxid 554] (species)<br>Number of reads: 10012<br>Number of identified reads: 9983 (99.71%)	• species: 150 (1.498%)<br>• **genus: 4688 (46.823%)**<br>• family: 953 (9.518%)<br>• order: 2023 (20.205%)<br>• class: 430 (4.294%)<br>• phylum: 261 (2.606%)<br>• superkingdom: 294 (2.936%)<br>• root: 1175 (11.735%)	• Pectobacterium brasiliense [taxid 180957]: 125 (1.248%)<br>• Pectobacterium polaris [taxid 2042057]: 69 (0.689%)<br>• Salmonella enterica [taxid 28901]: 49 (0.489%)<br>• Escherichia coli [taxid 562]: 18 (0.179%)<br>• Pectobacterium odoriferum [taxid 78398]: 18 (0.179%)<br>• Pectobacterium aquaticum [taxid 2204145]: 15 (0.149%)<br>• Pectobacterium betavasculorum [taxid 55207]: 11 (0.109%)<br>• Pectobacterium atrosepticum [taxid 29471]: 10 (0.099%)<br>• other: 155 (1.548%)
Benchmark OTU ID: `CP003415-_Proteobacteria`<br>OTU taxon: Pectobacterium parmentieri [taxid 1905730]<br>Expected: Pectobacterium parmentieri [taxid 1905730] (species)<br>Number of reads: 10736<br>Number of identified reads: 10675 (99.431%)	• species: 2168 (20.193%)<br>• **genus: 3495 (32.554%)**<br>• family: 843 (7.852%)<br>• order: 2004 (18.666%)<br>• class: 390 (3.632%)<br>• phylum: 270 (2.514%)<br>• superkingdom: 310 (2.887%)<br>• root: 1184 (11.028%)	• Salmonella enterica [taxid 28901]: 23 (0.214%)<br>• Escherichia coli [taxid 562]: 17 (0.158%)<br>• Pectobacterium carotovorum [taxid 554]: 12 (0.111%)<br>• Pectobacterium polaris [taxid 2042057]: 10 (0.093%)<br>• Pectobacterium atrosepticum [taxid 29471]: 9 (0.083%)<br>• Pectobacterium wasabiae [taxid 55208]: 9 (0.083%)<br>• Pectobacterium betavasculorum [taxid 55207]: 7 (0.065%)<br>• Pectobacterium punjabense [taxid 2108399]: 7 (0.065%)<br>• other: 121 (1.127%)

Operational Taxonomic Unit (OTU)	Correct identifications	Wrong or overspecific identifications at species rank
Benchmark OTU ID: `CP000422-_Firmicutes`<br>OTU taxon: Pediococcus pentosaceus ATCC 25745 [taxid 278197]<br>Expected: Pediococcus pentosaceus [taxid 1255] (species)<br>Number of reads: 2163<br>Number of identified reads: 2144 (99.121%)	• **species: 1407 (65.048%)**<br>• genus: 177 (8.183%)<br>• family: 141 (6.518%)<br>• order: 67 (3.097%)<br>• class: 34 (1.571%)<br>• phylum: 22 (1.017%)<br>• superkingdom: 89 (4.114%)<br>• root: 204 (9.431%)	• Pediococcus argentinicus [taxid 480391]: 2 (0.092%)<br>• Latilactobacillus curvatus [taxid 28038]: 1 (0.046%)<br>• Weissella cryptocerci [taxid 2506420]: 1 (0.046%)<br>• Lupinus albus [taxid 3870]: 1 (0.046%)<br>• Ligilactobacillus salivarius [taxid 1624]: 1 (0.046%)<br>• Lactobacillus gasseri [taxid 1596]: 1 (0.046%)<br>• Lactiplantibacillus plantarum [taxid 1590]: 1 (0.046%)<br>• Extubocellulus spinifer [taxid 265572]: 1 (0.046%)<br>• other: 7 (0.323%)
Benchmark OTU ID: `CP001681-_Bacteroidetes`<br>OTU taxon: Pedobacter heparinus DSM 2366 [taxid 485917]<br>Expected: Pedobacter heparinus [taxid 984] (species)<br>Number of reads: 35460<br>Number of identified reads: 35314 (99.588%)	• **species: 22048 (62.177%)**<br>• genus: 4821 (13.595%)<br>• family: 968 (2.729%)<br>• order: 93 (0.262%)<br>• class: 3 (0.008%)<br>• phylum: 2574 (7.258%)<br>• superkingdom: 1431 (4.035%)<br>• root: 3352 (9.452%)	• Pedobacter africanus [taxid 151894]: 47 (0.132%)<br>• Pedobacter nyackensis [taxid 475255]: 12 (0.033%)<br>• Pedobacter metabolipauper [taxid 425513]: 8 (0.022%)<br>• Pedobacter nutrimenti [taxid 1241337]: 6 (0.016%)<br>• Lupinus albus [taxid 3870]: 5 (0.014%)<br>• Pedobacter steynii [taxid 430522]: 4 (0.011%)<br>• Pedobacter hartonius [taxid 425514]: 4 (0.011%)<br>• Pedobacter psychrophilus [taxid 1826909]: 3 (0.008%)<br>• other: 114 (0.321%)
Benchmark OTU ID: `CP003075-_Proteobacteria`<br>OTU taxon: Pelagibacterium halotolerans B2 [taxid 1082931]<br>Expected: Pelagibacterium halotolerans [taxid 531813] (species)<br>Number of reads: 7992<br>Number of identified reads: 7951 (99.486%)	• **species: 3867 (48.385%)**<br>• genus: 769 (9.622%)<br>• family: 137 (1.714%)<br>• order: 1184 (14.814%)<br>• class: 445 (5.568%)<br>• phylum: 248 (3.103%)<br>• superkingdom: 376 (4.704%)<br>• root: 918 (11.486%)	• Pelagibacterium luteolum [taxid 440168]: 5 (0.062%)<br>• Pelagibacterium lacus [taxid 2282655]: 2 (0.025%)<br>• Bartonella queenslandensis [taxid 481138]: 1 (0.012%)<br>• Devosia enhydra [taxid 665118]: 1 (0.012%)<br>• Devosia epidermidihirudinis [taxid 1293439]: 1 (0.012%)<br>• Oceanimonas doudoroffii [taxid 84158]: 1 (0.012%)<br>• Breoghania corrubedonensis [taxid 665038]: 1 (0.012%)<br>• Bauldia litoralis [taxid 665467]: 1 (0.012%)<br>• other: 31 (0.387%)
Benchmark OTU ID: `CP000142-_Proteobacteria`<br>OTU taxon: Pelobacter carbinolicus DSM 2380 [taxid 338963]<br>Expected: Pelobacter carbinolicus [taxid 19] (species)<br>Number of reads: 7364<br>Number of identified reads: 7346 (99.755%)	• **species: 4954 (67.273%)**<br>• genus: 196 (2.661%)<br>• family: 48 (0.651%)<br>• order: 76 (1.032%)<br>• class: 111 (1.507%)<br>• phylum: 267 (3.625%)<br>• superkingdom: 498 (6.762%)<br>• root: 1190 (16.159%)	• Pelobacter acetylenicus [taxid 29542]: 6 (0.081%)<br>• Metallibacterium scheffleri [taxid 993689]: 1 (0.013%)<br>• Candidatus Accumulibacter phosphatis [taxid 327160]: 1 (0.013%)<br>• Lupinus albus [taxid 3870]: 1 (0.013%)<br>• Tessaracoccus oleiagri [taxid 686624]: 1 (0.013%)<br>• Giardia intestinalis [taxid 5741]: 1 (0.013%)<br>• Medicago truncatula [taxid 3880]: 1 (0.013%)<br>• Jonquetella anthropi [taxid 428712]: 1 (0.013%)<br>• other: 14 (0.19%)

Operational Taxonomic Unit (OTU)	Correct identifications	Wrong or overspecific identifications at species rank
Benchmark OTU ID: `CP000482-_Proteobacteria`<br>OTU taxon: Pelobacter propionicus DSM 2379 [taxid 338966]<br>Expected: Pelobacter propionicus [taxid 29543] (species)<br>Number of reads: 8134<br>Number of identified reads: 8104 (99.631%)	• **species: 5745 (70.629%)**<br>• genus: 4 (0.049%)<br>• family: 6 (0.073%)<br>• order: 443 (5.446%)<br>• class: 94 (1.155%)<br>• phylum: 308 (3.786%)<br>• superkingdom: 551 (6.774%)<br>• root: 948 (11.654%)	• Desulfovibrio ferrophilus [taxid 241368]: 3 (0.036%)<br>• Geobacter sp. SVR [taxid 2495594]: 3 (0.036%)<br>• Salmonella enterica [taxid 28901]: 1 (0.012%)<br>• Staphylococcus aureus [taxid 1280]: 1 (0.012%)<br>• Rhodopseudomonas pentothenatexigens [taxid 999699]: 1 (0.012%)<br>• Methylococcus capsulatus [taxid 414]: 1 (0.012%)<br>• Rhizophora mucronata [taxid 61149]: 1 (0.012%)<br>• Tetranychus urticae [taxid 32264]: 1 (0.012%)<br>• other: 10 (0.122%)
Benchmark OTU ID: `CP000096-_Chlorobi`<br>OTU taxon: Pelodictyon luteolum DSM 273 [taxid 319225]<br>Expected: Pelodictyon luteolum [taxid 1100] (species)<br>Number of reads: 28707<br>Number of identified reads: 28655 (99.818%)	• **species: 16570 (57.721%)**<br>• genus: 28 (0.097%)<br>• family: 4662 (16.239%)<br>• order: 16 (0.055%)<br>• class: 0 (0.0%)<br>• phylum: 1 (0.003%)<br>• superkingdom: 3153 (10.983%)<br>• root: 4183 (14.571%)	• Chlorobium phaeovibrioides [taxid 1094]: 37 (0.128%)<br>• Chlorobium phaeobacteroides [taxid 1096]: 7 (0.024%)<br>• Chlorobaculum tepidum [taxid 1097]: 6 (0.02%)<br>• Prosthecochloris vibrioformis [taxid 1098]: 5 (0.017%)<br>• Chlorobium limicola [taxid 1092]: 5 (0.017%)<br>• Chlorobaculum parvum [taxid 274539]: 4 (0.013%)<br>• Pelodictyon phaeoclathratiforme [taxid 34090]: 4 (0.013%)<br>• Bifidobacterium longum [taxid 216816]: 2 (0.006%)<br>• other: 79 (0.275%)
Benchmark OTU ID: `CP001110-_Chlorobi`<br>OTU taxon: Pelodictyon phaeoclathratiforme BU-1 [taxid 324925]<br>Expected: Pelodictyon phaeoclathratiforme [taxid 34090] (species)<br>Number of reads: 38214<br>Number of identified reads: 38146 (99.822%)	• **species: 22646 (59.261%)**<br>• genus: 58 (0.151%)<br>• family: 5008 (13.105%)<br>• order: 18 (0.047%)<br>• class: 0 (0.0%)<br>• phylum: 1 (0.002%)<br>• superkingdom: 4173 (10.92%)<br>• root: 6204 (16.234%)	• Chlorobium phaeobacteroides [taxid 1096]: 71 (0.185%)<br>• Chlorobium limicola [taxid 1092]: 11 (0.028%)<br>• Chlorobium ferrooxidans [taxid 84205]: 11 (0.028%)<br>• Chlorobium chlorochromatii [taxid 337090]: 9 (0.023%)<br>• Chlorobaculum tepidum [taxid 1097]: 7 (0.018%)<br>• Pelodictyon luteolum [taxid 1100]: 6 (0.015%)<br>• Chlorobaculum parvum [taxid 274539]: 4 (0.01%)<br>• Chlorobium phaeovibrioides [taxid 1094]: 4 (0.01%)<br>• other: 106 (0.277%)
Benchmark OTU ID: `CP002972-_Proteobacteria`<br>OTU taxon: Phaeobacter inhibens 2.10 [taxid 383629]<br>Expected: Phaeobacter inhibens [taxid 221822] (species)<br>Number of reads: 7572<br>Number of identified reads: 7547 (99.669%)	• species: 1327 (17.525%)<br>• **genus: 2211 (29.199%)**<br>• family: 2148 (28.367%)<br>• order: 56 (0.739%)<br>• class: 309 (4.08%)<br>• phylum: 199 (2.628%)<br>• superkingdom: 287 (3.79%)<br>• root: 999 (13.193%)	• Phaeobacter porticola [taxid 1844006]: 28 (0.369%)<br>• Phaeobacter gallaeciensis [taxid 60890]: 26 (0.343%)<br>• Phaeobacter piscinae [taxid 1580596]: 17 (0.224%)<br>• Phaeobacter italicus [taxid 481446]: 15 (0.198%)<br>• Ascidiaceihabitans donghaensis [taxid 1510460]: 7 (0.092%)<br>• Epibacterium ulvae [taxid 1156985]: 4 (0.052%)<br>• Flavimaricola marinus [taxid 1819565]: 4 (0.052%)<br>• Leisingera sp. ANG-Vp [taxid 1577896]: 3 (0.039%)<br>• other: 79 (1.043%)

Operational Taxonomic Unit (OTU)	Correct identifications	Wrong or overspecific identifications at species rank
Benchmark OTU ID: `CP002530-_Bacteroidetes`<br>OTU taxon: Phocaeicola salanitronis DSM 18170 [taxid 667015]<br>Expected: Phocaeicola salanitronis [taxid 376805] (species)<br>Number of reads: 28434<br>Number of identified reads: 28293 (99.504%)	• species: 11014 (38.735%)<br>• genus: 123 (0.432%)<br>• **order: 12021 (42.276%)**<br>• class: 37 (0.13%)<br>• phylum: 445 (1.565%)<br>• superkingdom: 1211 (4.258%)<br>• root: 3424 (12.041%)	• Phocaeicola vulgatus [taxid 821]: 22 (0.077%)<br>• Bacteroides fragilis [taxid 817]: 13 (0.045%)<br>• Phocaeicola coprocola [taxid 310298]: 12 (0.042%)<br>• Phocaeicola plebeius [taxid 310297]: 9 (0.031%)<br>• Bacteroides uniformis [taxid 820]: 8 (0.028%)<br>• Bacteroides intestinalis [taxid 329854]: 7 (0.024%)<br>• Bacteroides stercoris [taxid 46506]: 6 (0.021%)<br>• other: 116 (0.407%)
Benchmark OTU ID: `ENA|CR354532|CR354532.1-_Proteobacteria`<br>OTU taxon: Photobacterium profundum [taxid 74109]<br>Expected: Photobacterium profundum [taxid 74109] (species)<br>Number of reads: 4148<br>Number of identified reads: 4103 (98.915%)	• species: 1463 (35.27%)<br>• **genus: 1525 (36.764%)**<br>• family: 189 (4.556%)<br>• order: 0 (0.0%)<br>• class: 237 (5.713%)<br>• phylum: 65 (1.567%)<br>• superkingdom: 91 (2.193%)<br>• root: 533 (12.849%)	• Photobacterium indicum [taxid 81447]: 14 (0.337%)<br>• Photobacterium frigidiphilum [taxid 264736]: 13 (0.313%)<br>• Photobacterium halotolerans [taxid 265726]: 2 (0.048%)<br>• Vibrio splendidus [taxid 29497]: 2 (0.048%)<br>• Photobacterium jeanii [taxid 858640]: 2 (0.048%)<br>• Photobacterium leiognathi [taxid 553611]: 2 (0.048%)<br>• Vibrio thalassae [taxid 1243014]: 1 (0.024%)<br>• Photobacterium sanctipauli [taxid 1342794]: 1 (0.024%)<br>• other: 21 (0.506%)
Benchmark OTU ID: `AP012338-_Planctomycetes`<br>OTU taxon: Phycisphaera mikurensis NBRC 102666 [taxid 1142394]<br>Expected: Phycisphaera mikurensis [taxid 547188] (species)<br>Number of reads: 128243<br>Number of identified reads: 127628 (99.52%)	• **species: 100132 (78.079%)**<br>• genus: 5 (0.003%)<br>• family: 100 (0.077%)<br>• order: 69 (0.053%)<br>• class: 119 (0.092%)<br>• phylum: 760 (0.592%)<br>• superkingdom: 15644 (12.198%)<br>• root: 10644 (8.299%)	• Rhodoblastus acidophilus [taxid 1074]: 3 (0.002%)<br>• Lingulodinium polyedra [taxid 160621]: 3 (0.002%)<br>• Bacillus subtilis [taxid 1423]: 2 (0.001%)<br>• Roseospira marina [taxid 140057]: 2 (0.001%)<br>• Porphyra umbilicalis [taxid 2786]: 2 (0.001%)<br>• Pseudomonas alcaligenes [taxid 43263]: 2 (0.001%)<br>• Chlamydomonas reinhardtii [taxid 3055]: 2 (0.001%)<br>• Mycobacterium interjectum [taxid 33895]: 2 (0.001%)<br>• other: 199 (0.155%)
Benchmark OTU ID: `AE017261-_Euryarchaeota`<br>OTU taxon: Picrophilus torridus DSM 9790 [taxid 263820]<br>Expected: Picrophilus torridus [taxid 82076] (species)<br>Number of reads: 1226<br>Number of identified reads: 1195 (97.471%)	• species: 155 (12.642%)<br>• **genus: 624 (50.897%)**<br>• family: 0 (0.0%)<br>• order: 22 (1.794%)<br>• class: 0 (0.0%)<br>• phylum: 0 (0.0%)<br>• superkingdom: 9 (0.734%)<br>• root: 373 (30.424%)	• Picrophilus oshimae [taxid 46632]: 9 (0.734%)<br>• Acidiplasma aeolicum [taxid 507754]: 2 (0.163%)<br>• Yasminevirus sp. GU-2018 [taxid 2420051]: 1 (0.081%)

Operational Taxonomic Unit (OTU)	Correct identifications	Wrong or overspecific identifications at species rank
Benchmark OTU ID: `CP001848-_Planctomycetes`<br>OTU taxon: Pirellula staleyi DSM 6068 [taxid 530564]<br>Expected: Pirellula staleyi [taxid 125] (species)<br>Number of reads: 229464<br>Number of identified reads: 228795 (99.708%)	• **species: 166011 (72.347%)**<br>• genus: 46 (0.02%)<br>• family: 608 (0.264%)<br>• order: 204 (0.088%)<br>• class: 2408 (1.049%)<br>• phylum: 4989 (2.174%)<br>• superkingdom: 16343 (7.122%)<br>• root: 37907 (16.519%)	• Lignipirellula cremea [taxid 2528010]: 13 (0.005%)<br>• Paludisphaera borealis [taxid 1387353]: 5 (0.002%)<br>• Novipirellula artificiosorum [taxid 2528016]: 5 (0.002%)<br>• Dracunculus medinensis [taxid 318479]: 5 (0.002%)<br>• Rubripirellula reticaptiva [taxid 2528013]: 5 (0.002%)<br>• Bremerella volcania [taxid 2527984]: 5 (0.002%)<br>• Blastopirellula marina [taxid 124]: 5 (0.002%)<br>• Heliobacterium modesticaldum [taxid 35701]: 5 (0.002%)<br>• other: 379 (0.165%)
Benchmark OTU ID: `CP001744-_Planctomycetes`<br>OTU taxon: Planctopirus limnophila DSM 3776 [taxid 521674]<br>Expected: Planctopirus limnophila [taxid 120] (species)<br>Number of reads: 196762<br>Number of identified reads: 196491 (99.862%)	• species: 56376 (28.651%)<br>• **genus: 85981 (43.697%)**<br>• family: 1456 (0.739%)<br>• order: 167 (0.084%)<br>• class: 676 (0.343%)<br>• phylum: 2701 (1.372%)<br>• superkingdom: 12803 (6.506%)<br>• root: 36107 (18.35%)	• Planctopirus hydrillae [taxid 1841610]: 1247 (0.633%)<br>• Planctopirus ephydatiae [taxid 2528019]: 757 (0.384%)<br>• Schlesneria paludicola [taxid 360056]: 8 (0.004%)<br>• Fimbriiglobus ruber [taxid 1908690]: 7 (0.003%)<br>• bacterium [taxid 1869227]: 5 (0.002%)<br>• Polystyrenella longa [taxid 2528007]: 4 (0.002%)<br>• Oxyrrhis marina [taxid 2969]: 4 (0.002%)<br>• Gemmata massiliana [taxid 1210884]: 3 (0.001%)<br>• other: 331 (0.168%)
Benchmark OTU ID: `CP003590-_Cyanobacteria`<br>OTU taxon: Pleurocapsa sp. PCC 7327 [taxid 118163]<br>Expected: Pleurocapsa minor [taxid 54308] (species)<br>Number of reads: 29053<br>Number of identified reads: 28816 (99.184%)	• **species: 10260 (35.314%)**<br>• genus: 6 (0.02%)<br>• family: 10 (0.034%)<br>• order: 7423 (25.549%)<br>• phylum: 4347 (14.962%)<br>• superkingdom: 1971 (6.784%)<br>• root: 4729 (16.277%)	• Hydrococcus rivularis [taxid 1616834]: 287 (0.987%)<br>• Microcystis aeruginosa [taxid 1126]: 7 (0.024%)<br>• Hyella patelloides [taxid 1982969]: 6 (0.02%)<br>• Gloeothece verrucosa [taxid 2546359]: 5 (0.017%)<br>• Stanieria cyanosphaera [taxid 102116]: 5 (0.017%)<br>• Rippkaea orientalis [taxid 2546366]: 3 (0.01%)<br>• Aphanothece sacrum [taxid 1122]: 3 (0.01%)<br>• other: 106 (0.364%)
Benchmark OTU ID: `CP000529-_Proteobacteria`<br>OTU taxon: Polaromonas naphthalenivorans CJ2 [taxid 365044]<br>Expected: Polaromonas naphthalenivorans [taxid 216465] (species)<br>Number of reads: 9039<br>Number of identified reads: 9020 (99.789%)	• **species: 4805 (53.158%)**<br>• genus: 674 (7.456%)<br>• family: 555 (6.14%)<br>• order: 1050 (11.616%)<br>• class: 157 (1.736%)<br>• phylum: 632 (6.991%)<br>• superkingdom: 349 (3.861%)<br>• root: 789 (8.728%)	• Pseudoxanthomonas spadix [taxid 415229]: 5 (0.055%)<br>• Polaromonas sp. Pch-P [taxid 2082385]: 4 (0.044%)<br>• Azotobacter chroococcum [taxid 353]: 2 (0.022%)<br>• Polaromonas vacuolata [taxid 37448]: 2 (0.022%)<br>• Burkholderia vietnamiensis [taxid 60552]: 2 (0.022%)<br>• Rhodoferax antarcticus [taxid 81479]: 1 (0.011%)<br>• Durinskia baltica [taxid 400756]: 1 (0.011%)<br>• Jimgerdemannia flammicorona [taxid 994334]: 1 (0.011%)<br>• other: 32 (0.354%)

Operational Taxonomic Unit (OTU)	Correct identifications	Wrong or overspecific identifications at species rank
Benchmark OTU ID: `CP000316-_Proteobacteria`<br>OTU taxon: Polaromonas sp. JS666 [taxid 296591]<br>Expected: Polaromonas [taxid 52972] (genus)<br>Number of reads: 10816<br>Number of identified reads: 10784 (99.704%)	• **genus: 6188 (57.211%)**<br>• family: 773 (7.146%)<br>• order: 1345 (12.435%)<br>• class: 208 (1.923%)<br>• phylum: 782 (7.23%)<br>• superkingdom: 420 (3.883%)<br>• root: 1062 (9.818%)	• **Polaromonas sp. Pch-P [taxid 2082385]: 555 (5.131%)**<br>• Variovorax paradoxus [taxid 34073]: 5 (0.046%)<br>• Polaromonas vacuolata [taxid 37448]: 5 (0.046%)<br>• Polaromonas naphthalenivorans [taxid 216465]: 3 (0.027%)<br>• Rivibacter subsaxonicus [taxid 457575]: 2 (0.018%)<br>• Salmonella enterica [taxid 28901]: 2 (0.018%)<br>• Roseateles depolymerans [taxid 76731]: 1 (0.009%)<br>• other: 49 (0.453%)
Benchmark OTU ID: `CP002568-_Proteobacteria`<br>OTU taxon: Polymorphum gilvum SL003B-26A1 [taxid 991905]<br>Expected: Polymorphum gilvum [taxid 991904] (species)<br>Number of reads: 9577<br>Number of identified reads: 9539 (99.603%)	• **species: 5474 (57.157%)**<br>• genus: 0 (0.0%)<br>• class: 2364 (24.684%)<br>• phylum: 372 (3.884%)<br>• superkingdom: 469 (4.897%)<br>• root: 851 (8.885%)	• Lupinus albus [taxid 3870]: 3 (0.031%)<br>• Labrenzia alexandrii [taxid 388408]: 2 (0.02%)<br>• Lutibaculum baratangense [taxid 1358440]: 1 (0.01%)<br>• Lichenibacterium ramalinae [taxid 2316527]: 1 (0.01%)<br>• Bradyrhizobium oligotrophicum [taxid 44255]: 1 (0.01%)<br>• Notoacmeibacter marinus [taxid 1876515]: 1 (0.01%)<br>• other: 36 (0.375%)
Benchmark OTU ID: `CP000655-_Proteobacteria`<br>OTU taxon: Polynucleobacter asymbioticus QLW-P1DMWA-1 [taxid 312153]<br>Expected: Polynucleobacter asymbioticus [taxid 576611] (species)<br>Number of reads: 3975<br>Number of identified reads: 3958 (99.572%)	• **species: 1970 (49.559%)**<br>• genus: 959 (24.125%)<br>• family: 63 (1.584%)<br>• order: 139 (3.496%)<br>• class: 113 (2.842%)<br>• phylum: 229 (5.761%)<br>• superkingdom: 129 (3.245%)<br>• root: 351 (8.83%)	• Polynucleobacter aenigmaticus [taxid 1743164]: 2 (0.05%)<br>• Polynucleobacter wuianus [taxid 1743168]: 2 (0.05%)<br>• Methylobacter tundripaludum [taxid 173365]: 1 (0.025%)<br>• Cupriavidus taiwanensis [taxid 164546]: 1 (0.025%)<br>• Polynucleobacter campilacus [taxid 1743163]: 1 (0.025%)<br>• Helianthus annuus [taxid 4232]: 1 (0.025%)<br>• Leptothrix cholodnii [taxid 34029]: 1 (0.025%)<br>• Rhodobacter sphaeroides [taxid 1063]: 1 (0.025%)<br>• other: 9 (0.226%)
Benchmark OTU ID: `CP001010-_Proteobacteria`<br>OTU taxon: Polynucleobacter necessarius STIR1 [taxid 452638]<br>Expected: Polynucleobacter necessarius [taxid 576610] (species)<br>Number of reads: 2627<br>Number of identified reads: 2615 (99.543%)	• **species: 1076 (40.959%)**<br>• genus: 709 (26.988%)<br>• family: 42 (1.598%)<br>• order: 90 (3.425%)<br>• class: 101 (3.844%)<br>• phylum: 166 (6.318%)<br>• superkingdom: 94 (3.578%)<br>• root: 332 (12.637%)	• Polynucleobacter asymbioticus [taxid 576611]: 6 (0.228%)<br>• Polynucleobacter duraquae [taxid 1835254]: 4 (0.152%)<br>• Polynucleobacter wuianus [taxid 1743168]: 2 (0.076%)<br>• Polynucleobacter campilacus [taxid 1743163]: 2 (0.076%)<br>• beta proteobacterium CB [taxid 543913]: 2 (0.076%)<br>• Polynucleobacter paneuropaeus [taxid 2527775]: 2 (0.076%)<br>• Moniliophthora roreri [taxid 221103]: 1 (0.038%)<br>• Polynucleobacter hirudinilacicola [taxid 1743166]: 1 (0.038%)<br>• other: 9 (0.342%)
Benchmark OTU ID: `CP002589-_Bacteroidetes`<br>OTU taxon: Prevotella denticola F0289 [taxid 767031]<br>Expected: Prevotella denticola [taxid 28129] (species)<br>Number of reads: 18514<br>Number of identified reads: 18462 (99.719%)	• **species: 7261 (39.218%)**<br>• genus: 6979 (37.695%)<br>• family: 110 (0.594%)<br>• order: 599 (3.235%)<br>• class: 18 (0.097%)<br>• phylum: 216 (1.166%)<br>• superkingdom: 724 (3.91%)<br>• root: 2528 (13.654%)	• Prevotella multiformis [taxid 282402]: 58 (0.313%)<br>• Prevotella histicola [taxid 470565]: 19 (0.102%)<br>• Prevotella intermedia [taxid 28131]: 19 (0.102%)<br>• Prevotella disiens [taxid 28130]: 16 (0.086%)<br>• Prevotella nigrescens [taxid 28133]: 14 (0.075%)<br>• Prevotella melaninogenica [taxid 28132]: 13 (0.07%)<br>• Prevotella veroralis [taxid 28137]: 12 (0.064%)<br>• Prevotella corporis [taxid 28128]: 8 (0.043%)<br>• other: 111 (0.599%)

Operational Taxonomic Unit (OTU)	Correct identifications	Wrong or overspecific identifications at species rank
Benchmark OTU ID: `CP002122-_Bacteroidetes`<br>OTU taxon: Prevotella melaninogenica ATCC 25845 [taxid 553174]<br>Expected: Prevotella melaninogenica [taxid 28132] (species)<br>Number of reads: 9842<br>Number of identified reads: 9696 (98.516%)	• species: 1690 (17.171%)<br>• **genus: 6027 (61.237%)**<br>• family: 47 (0.477%)<br>• order: 325 (3.302%)<br>• class: 8 (0.081%)<br>• phylum: 109 (1.107%)<br>• superkingdom: 344 (3.495%)<br>• root: 1140 (11.583%)	• Prevotella histicola [taxid 470565]: 23 (0.233%)<br>• Prevotella scopos [taxid 589437]: 20 (0.203%)<br>• Prevotella fusca [taxid 589436]: 10 (0.101%)<br>• Prevotella oulorum [taxid 28136]: 9 (0.091%)<br>• Prevotella bivia [taxid 28125]: 6 (0.06%)<br>• Prevotella copri [taxid 165179]: 5 (0.05%)<br>• Prevotella amnii [taxid 419005]: 5 (0.05%)<br>• Prevotella multiformis [taxid 282402]: 5 (0.05%)<br>• other: 66 (0.67%)
Benchmark OTU ID: `CP002123-_Bacteroidetes`<br>OTU taxon: Prevotella melaninogenica ATCC 25845 [taxid 553174]<br>Expected: Prevotella melaninogenica [taxid 28132] (species)<br>Number of reads: 6615<br>Number of identified reads: 6529 (98.699%)	• species: 1226 (18.533%)<br>• **genus: 3929 (59.395%)**<br>• family: 27 (0.408%)<br>• order: 203 (3.068%)<br>• class: 3 (0.045%)<br>• phylum: 58 (0.876%)<br>• superkingdom: 203 (3.068%)<br>• root: 878 (13.272%)	• Prevotella scopos [taxid 589437]: 18 (0.272%)<br>• Prevotella multiformis [taxid 282402]: 17 (0.256%)<br>• Prevotella denticola [taxid 28129]: 15 (0.226%)<br>• Prevotella histicola [taxid 470565]: 10 (0.151%)<br>• Prevotella fusca [taxid 589436]: 9 (0.136%)<br>• Prevotella veroralis [taxid 28137]: 9 (0.136%)<br>• Prevotella intermedia [taxid 28131]: 8 (0.12%)<br>• Prevotella salivae [taxid 228604]: 7 (0.105%)<br>• other: 56 (0.846%)
Benchmark OTU ID: `CP002006-_Bacteroidetes`<br>OTU taxon: Prevotella ruminicola 23 [taxid 264731]<br>Expected: Prevotella ruminicola [taxid 839] (species)<br>Number of reads: 23697<br>Number of identified reads: 23609 (99.628%)	• species: 7113 (30.016%)<br>• **genus: 11991 (50.601%)**<br>• family: 436 (1.839%)<br>• order: 727 (3.067%)<br>• class: 19 (0.08%)<br>• phylum: 288 (1.215%)<br>• superkingdom: 899 (3.793%)<br>• root: 2122 (8.954%)	• Prevotella copri [taxid 165179]: 9 (0.037%)<br>• Prevotella marshii [taxid 189722]: 8 (0.033%)<br>• Prevotella pectinovora [taxid 1602169]: 7 (0.029%)<br>• Prevotella micans [taxid 189723]: 5 (0.021%)<br>• Prevotella multisaccharivorax [taxid 310514]: 4 (0.016%)<br>• Prevotella buccae [taxid 28126]: 4 (0.016%)<br>• Prevotella stercorea [taxid 363265]: 3 (0.012%)<br>• Prevotella dentalis [taxid 52227]: 3 (0.012%)<br>• other: 65 (0.274%)
Benchmark OTU ID: `CP000551-_Cyanobacteria`<br>OTU taxon: Prochlorococcus marinus str. AS9601 [taxid 146891]<br>Expected: Prochlorococcus marinus [taxid 1219] (species)<br>Number of reads: 7161<br>Number of identified reads: 7021 (98.044%)	• **species: 2941 (41.069%)**<br>• genus: 2578 (36.0%)<br>• family: 0 (0.0%)<br>• order: 151 (2.108%)<br>• phylum: 95 (1.326%)<br>• superkingdom: 410 (5.725%)<br>• root: 835 (11.66%)	• cyanobacterium endosymbiont of Rhopalodia gibberula [taxid 1763363]: 1 (0.013%)<br>• Xerophyta humilis [taxid 211604]: 1 (0.013%)<br>• Pinus koraiensis [taxid 88728]: 1 (0.013%)<br>• cyanobiont of Ornithocercus magnificus [taxid 2496102]: 1 (0.013%)<br>• Candidatus Synechococcus spongiarum [taxid 431041]: 1 (0.013%)<br>• Shewanella maritima [taxid 2520507]: 1 (0.013%)<br>• Legionella erythra [taxid 448]: 1 (0.013%)<br>• other: 6 (0.083%)

Operational Taxonomic Unit (OTU)	Correct identifications	Wrong or overspecific identifications at species rank
Benchmark OTU ID: `CP000878-_Cyanobacteria`<br>OTU taxon: Prochlorococcus marinus str. MIT 9211 [taxid 93059]<br>Expected: Prochlorococcus marinus [taxid 1219] (species)<br>Number of reads: 7283<br>Number of identified reads: 7195 (98.791%)	• **species: 4899 (67.266%)**<br>• genus: 293 (4.023%)<br>• family: 0 (0.0%)<br>• order: 262 (3.597%)<br>• phylum: 135 (1.853%)<br>• superkingdom: 498 (6.837%)<br>• root: 1097 (15.062%)	• Tissierella creatinini [taxid 43143]: 1 (0.013%)<br>• Aureimonas flava [taxid 2320271]: 1 (0.013%)<br>• Corethron pennatum [taxid 218684]: 1 (0.013%)<br>• Nocardioides guangzhouensis [taxid 2497878]: 1 (0.013%)<br>• Lupinus albus [taxid 3870]: 1 (0.013%)<br>• Candidatus Erwinia haradaeae [taxid 1922217]: 1 (0.013%)<br>• Heterorhabditis bacteriophora [taxid 37862]: 1 (0.013%)<br>• other: 8 (0.109%)
Benchmark OTU ID: `CP000825-_Cyanobacteria`<br>OTU taxon: Prochlorococcus marinus str. MIT 9215 [taxid 93060]<br>Expected: Prochlorococcus marinus [taxid 1219] (species)<br>Number of reads: 7615<br>Number of identified reads: 7424 (97.491%)	• **species: 3914 (51.398%)**<br>• genus: 1811 (23.782%)<br>• family: 0 (0.0%)<br>• order: 174 (2.284%)<br>• phylum: 96 (1.26%)<br>• superkingdom: 476 (6.25%)<br>• root: 933 (12.252%)	• Medicago truncatula [taxid 3880]: 3 (0.039%)<br>• Paulinella micropora [taxid 1928728]: 2 (0.026%)<br>• Solanum chacoense [taxid 4108]: 2 (0.026%)<br>• Candidatus Thermokryptus mobilis [taxid 1643428]: 1 (0.013%)<br>• Microcystis aeruginosa [taxid 1126]: 1 (0.013%)<br>• Lupinus albus [taxid 3870]: 1 (0.013%)<br>• Gloeothece verrucosa [taxid 2546359]: 1 (0.013%)<br>• other: 8 (0.105%)
Benchmark OTU ID: `CP000576-_Cyanobacteria`<br>OTU taxon: Prochlorococcus marinus str. MIT 9301 [taxid 167546]<br>Expected: Prochlorococcus marinus [taxid 1219] (species)<br>Number of reads: 6976<br>Number of identified reads: 6803 (97.52%)	• **species: 2918 (41.829%)**<br>• genus: 2389 (34.245%)<br>• family: 0 (0.0%)<br>• order: 172 (2.465%)<br>• phylum: 88 (1.261%)<br>• superkingdom: 428 (6.135%)<br>• root: 803 (11.51%)	• Staphylococcus hominis [taxid 1290]: 1 (0.014%)<br>• Planktothrix tepida [taxid 1678309]: 1 (0.014%)<br>• Capsicum annuum [taxid 4072]: 1 (0.014%)<br>• Trypanosoma rangeli [taxid 5698]: 1 (0.014%)<br>• Novipirellula aureliae [taxid 2527966]: 1 (0.014%)<br>• Aphanocapsa feldmannii [taxid 192050]: 1 (0.014%)<br>• Escherichia coli [taxid 562]: 1 (0.014%)<br>• other: 2 (0.028%)
Benchmark OTU ID: `CP000554-_Cyanobacteria`<br>OTU taxon: Prochlorococcus marinus str. MIT 9303 [taxid 59922]<br>Expected: Prochlorococcus marinus [taxid 1219] (species)<br>Number of reads: 13845<br>Number of identified reads: 13769 (99.451%)	• species: 3069 (22.166%)<br>• **genus: 5812 (41.979%)**<br>• family: 0 (0.0%)<br>• order: 946 (6.832%)<br>• phylum: 208 (1.502%)<br>• superkingdom: 929 (6.71%)<br>• root: 2779 (20.072%)	• Candidatus Synechococcus spongiarum [taxid 431041]: 3 (0.021%)<br>• Puccinia sorghi [taxid 27349]: 1 (0.007%)<br>• Gloeothece verrucosa [taxid 2546359]: 1 (0.007%)<br>• Pedobacter psychrophilus [taxid 1826909]: 1 (0.007%)<br>• Punica granatum [taxid 22663]: 1 (0.007%)<br>• Capsulimonas corticalis [taxid 2219043]: 1 (0.007%)<br>• Durio zibethinus [taxid 66656]: 1 (0.007%)<br>• other: 17 (0.122%)
Benchmark OTU ID: `CP000111-_Cyanobacteria`<br>OTU taxon: Prochlorococcus marinus str. MIT 9312 [taxid 74546]<br>Expected: Prochlorococcus marinus [taxid 1219] (species)<br>Number of reads: 7421<br>Number of identified reads: 7260 (97.83%)	• **species: 4048 (54.547%)**<br>• genus: 1558 (20.994%)<br>• family: 1 (0.013%)<br>• order: 150 (2.021%)<br>• phylum: 97 (1.307%)<br>• superkingdom: 475 (6.4%)<br>• root: 913 (12.302%)	• Acartia pacifica [taxid 335913]: 1 (0.013%)<br>• Leptolyngbya valderiana [taxid 322865]: 1 (0.013%)<br>• Panstrongylus lignarius [taxid 156445]: 1 (0.013%)<br>• Protopolystoma xenopodis [taxid 117903]: 1 (0.013%)<br>• Lupinus albus [taxid 3870]: 1 (0.013%)<br>• Auxenochlorella protothecoides [taxid 3075]: 1 (0.013%)<br>• Salipaludibacillus keqinensis [taxid 2045207]: 1 (0.013%)<br>• other: 5 (0.067%)

Operational Taxonomic Unit (OTU)	Correct identifications	Wrong or overspecific identifications at species rank
Benchmark OTU ID: `BX548175-_Cyanobacteria`<br>OTU taxon: Prochlorococcus marinus str. MIT 9313 [taxid 74547]<br>Expected: Prochlorococcus marinus [taxid 1219] (species)<br>Number of reads: 12049<br>Number of identified reads: 11951 (99.186%)	• species: 3152 (26.159%)<br>• **genus: 4484 (37.214%)**<br>• family: 0 (0.0%)<br>• order: 910 (7.552%)<br>• phylum: 176 (1.46%)<br>• superkingdom: 753 (6.249%)<br>• root: 2451 (20.341%)	• Solanum chacoense [taxid 4108]: 2 (0.016%)<br>• Medicago truncatula [taxid 3880]: 2 (0.016%)<br>• Aphanocapsa feldmannii [taxid 192050]: 2 (0.016%)<br>• Elsinoe australis [taxid 40998]: 1 (0.008%)<br>• Treponema primitia [taxid 88058]: 1 (0.008%)<br>• Azospirillum brasilense [taxid 192]: 1 (0.008%)<br>• Bipolaris sorokiniana [taxid 45130]: 1 (0.008%)<br>• other: 30 (0.248%)
Benchmark OTU ID: `CP000552-_Cyanobacteria`<br>OTU taxon: Prochlorococcus marinus str. MIT 9515 [taxid 167542]<br>Expected: Prochlorococcus marinus [taxid 1219] (species)<br>Number of reads: 7387<br>Number of identified reads: 7177 (97.157%)	• **species: 5031 (68.106%)**<br>• genus: 457 (6.186%)<br>• family: 0 (0.0%)<br>• order: 165 (2.233%)<br>• phylum: 93 (1.258%)<br>• superkingdom: 422 (5.712%)<br>• root: 1002 (13.564%)	• Thiohalophilus thiocyanatoxydans [taxid 381308]: 1 (0.013%)<br>• Pseudomonas aeruginosa [taxid 287]: 1 (0.013%)<br>• Amycolatopsis methanolica [taxid 1814]: 1 (0.013%)<br>• Salmonella enterica [taxid 28901]: 1 (0.013%)<br>• Scrippsiella hangoei [taxid 268821]: 1 (0.013%)<br>• Dokdonella immobilis [taxid 578942]: 1 (0.013%)<br>• Campanula carpatica [taxid 171910]: 1 (0.013%)<br>• other: 2 (0.027%)
Benchmark OTU ID: `CP000553-_Cyanobacteria`<br>OTU taxon: Prochlorococcus marinus str. NATL1A [taxid 167555]<br>Expected: Prochlorococcus marinus [taxid 1219] (species)<br>Number of reads: 8447<br>Number of identified reads: 8261 (97.798%)	• **species: 4006 (47.425%)**<br>• genus: 2152 (25.476%)<br>• family: 0 (0.0%)<br>• order: 227 (2.687%)<br>• phylum: 127 (1.503%)<br>• superkingdom: 493 (5.836%)<br>• root: 1238 (14.656%)	• Richelia intracellularis [taxid 1164990]: 1 (0.011%)<br>• Thermobaculum terrenum [taxid 166501]: 1 (0.011%)<br>• Daphnia magna [taxid 35525]: 1 (0.011%)<br>• Gloeomargarita lithophora [taxid 1188228]: 1 (0.011%)<br>• Aliidiomarina taiwanensis [taxid 946228]: 1 (0.011%)<br>• Trifolium medium [taxid 97028]: 1 (0.011%)<br>• Arthrospira platensis [taxid 118562]: 1 (0.011%)<br>• other: 14 (0.165%)
Benchmark OTU ID: `CP000095-_Cyanobacteria`<br>OTU taxon: Prochlorococcus marinus str. NATL2A [taxid 59920]<br>Expected: Prochlorococcus marinus [taxid 1219] (species)<br>Number of reads: 8302<br>Number of identified reads: 8146 (98.12%)	• **species: 4003 (48.217%)**<br>• genus: 2021 (24.343%)<br>• family: 0 (0.0%)<br>• order: 283 (3.408%)<br>• phylum: 111 (1.337%)<br>• superkingdom: 521 (6.275%)<br>• root: 1189 (14.321%)	• Paulinella longichromatophora [taxid 1708747]: 1 (0.012%)<br>• Euhalothece natronophila [taxid 577489]: 1 (0.012%)<br>• Aerococcus suis [taxid 371602]: 1 (0.012%)<br>• Salix viminalis [taxid 40686]: 1 (0.012%)<br>• Medicago truncatula [taxid 3880]: 1 (0.012%)<br>• Aspergillus terreus [taxid 33178]: 1 (0.012%)<br>• Petrohua bernabei [taxid 320783]: 1 (0.012%)<br>• other: 10 (0.12%)
Benchmark OTU ID: `AE017126-_Cyanobacteria`<br>OTU taxon: Prochlorococcus marinus subsp. marinus str. CCMP1375 [taxid 167539]<br>Expected: Prochlorococcus marinus [taxid 1219] (species)<br>Number of reads: 7647<br>Number of identified reads: 7529 (98.456%)	• **species: 5305 (69.373%)**<br>• genus: 316 (4.132%)<br>• family: 0 (0.0%)<br>• order: 246 (3.216%)<br>• phylum: 110 (1.438%)<br>• superkingdom: 476 (6.224%)<br>• root: 1053 (13.77%)	• Ancylostoma caninum [taxid 29170]: 1 (0.013%)<br>• Tetrahymena thermophila [taxid 5911]: 1 (0.013%)<br>• Pseudomonas amygdali [taxid 47877]: 1 (0.013%)<br>• Pseudomonas syringae [taxid 317]: 1 (0.013%)<br>• Candidatus Synechococcus spongiarum [taxid 431041]: 1 (0.013%)<br>• Nesidiocoris tenuis [taxid 355587]: 1 (0.013%)<br>• Kerstersia gyiorum [taxid 206506]: 1 (0.013%)<br>• other: 15 (0.196%)

Operational Taxonomic Unit (OTU)	Correct identifications	Wrong or overspecific identifications at species rank
Benchmark OTU ID: `BX548174-_Cyanobacteria`<br>OTU taxon: Prochlorococcus marinus subsp. pastoris str. CCMP1986 [taxid 59919]<br>Expected: Prochlorococcus marinus [taxid 1219] (species)<br>Number of reads: 7082<br>Number of identified reads: 6911 (97.585%)	• **species: 4818 (68.031%)**<br>• genus: 464 (6.551%)<br>• family: 0 (0.0%)<br>• order: 167 (2.358%)<br>• phylum: 88 (1.242%)<br>• superkingdom: 460 (6.495%)<br>• root: 902 (12.736%)	• Crocosphaera watsonii [taxid 263511]: 1 (0.014%)<br>• Peptoniphilus asaccharolyticus [taxid 1258]: 1 (0.014%)<br>• Paraburkholderia phenazinium [taxid 60549]: 1 (0.014%)<br>• Pseudidiomarina aestuarii [taxid 624146]: 1 (0.014%)<br>• Udotea argentea [taxid 189445]: 1 (0.014%)<br>• Malaciobacter mytili [taxid 603050]: 1 (0.014%)<br>• Artemisia annua [taxid 35608]: 1 (0.014%)<br>• other: 3 (0.042%)
Benchmark OTU ID: `FN806773-_Actinobacteria`<br>OTU taxon: Propionibacterium freudenreichii subsp. shermanii CIRM-BIA1 [taxid 754252]<br>Expected: Propionibacterium freudenreichii [taxid 1744] (species)<br>Number of reads: 10417<br>Number of identified reads: 10361 (99.462%)	• **species: 7492 (71.92%)**<br>• genus: 137 (1.315%)<br>• family: 227 (2.179%)<br>• order: 34 (0.326%)<br>• class: 850 (8.159%)<br>• phylum: 18 (0.172%)<br>• superkingdom: 682 (6.546%)<br>• root: 914 (8.774%)	• Acidipropionibacterium jensenii [taxid 1749]: 6 (0.057%)<br>• Kocuria marina [taxid 223184]: 2 (0.019%)<br>• Microbacterium esteraromaticum [taxid 57043]: 2 (0.019%)<br>• Schlegelella thermodepolymerans [taxid 215580]: 1 (0.009%)<br>• Gordonia amarae [taxid 36821]: 1 (0.009%)<br>• Enterobius vermicularis [taxid 51028]: 1 (0.009%)<br>• Sandaracinus amylolyticus [taxid 927083]: 1 (0.009%)<br>• Brevibacterium aurantiacum [taxid 273384]: 1 (0.009%)<br>• other: 35 (0.335%)
Benchmark OTU ID: `CP001108-_Chlorobi`<br>OTU taxon: Prosthecochloris aestuarii DSM 271 [taxid 290512]<br>Expected: Prosthecochloris aestuarii [taxid 1102] (species)<br>Number of reads: 30862<br>Number of identified reads: 30816 (99.85%)	• species: 5329 (17.267%)<br>• **genus: 15960 (51.714%)**<br>• family: 1537 (4.98%)<br>• order: 20 (0.064%)<br>• class: 0 (0.0%)<br>• phylum: 1 (0.003%)<br>• superkingdom: 3235 (10.482%)<br>• root: 4705 (15.245%)	• Prosthecochloris sp. ZM [taxid 2283143]: 195 (0.631%)<br>• Chlorobium phaeobacteroides [taxid 1096]: 23 (0.074%)<br>• Prosthecochloris marina [taxid 2017681]: 23 (0.074%)<br>• Chlorobaculum limnaeum [taxid 274537]: 8 (0.025%)<br>• Chlorobaculum parvum [taxid 274539]: 7 (0.022%)<br>• Chlorobium phaeovibrioides [taxid 1094]: 3 (0.009%)<br>• Salmonella enterica [taxid 28901]: 3 (0.009%)<br>• Chlorobium limicola [taxid 1092]: 2 (0.006%)<br>• other: 51 (0.165%)
Benchmark OTU ID: `AM942759-_Proteobacteria`<br>OTU taxon: Proteus mirabilis HI4320 [taxid 529507]<br>Expected: Proteus mirabilis [taxid 584] (species)<br>Number of reads: 8259<br>Number of identified reads: 8128 (98.413%)	• species: 897 (10.86%)<br>• **genus: 2853 (34.544%)**<br>• family: 760 (9.202%)<br>• order: 2004 (24.264%)<br>• class: 466 (5.642%)<br>• phylum: 138 (1.67%)<br>• superkingdom: 227 (2.748%)<br>• root: 779 (9.432%)	• Salmonella enterica [taxid 28901]: 23 (0.278%)<br>• Escherichia coli [taxid 562]: 17 (0.205%)<br>• Proteus penneri [taxid 102862]: 10 (0.121%)<br>• Proteus vulgaris [taxid 585]: 6 (0.072%)<br>• Providencia stuartii [taxid 588]: 4 (0.048%)<br>• Klebsiella pneumoniae [taxid 573]: 4 (0.048%)<br>• Proteus columbae [taxid 1987580]: 4 (0.048%)<br>• Photorhabdus asymbiotica [taxid 291112]: 3 (0.036%)<br>• other: 62 (0.75%)
Benchmark OTU ID: `CP003592-_Cyanobacteria`<br>OTU taxon: Pseudanabaena sp. PCC 7367 [taxid 82654]<br>Expected: Pseudanabaena [taxid 1152] (genus)<br>Number of reads: 26216<br>Number of identified reads: 25836 (98.55%)	• **genus: 18235 (69.556%)**<br>• family: 5 (0.019%)<br>• order: 176 (0.671%)<br>• phylum: 1027 (3.917%)<br>• superkingdom: 1627 (6.206%)<br>• root: 4716 (17.989%)	• Pseudanabaena frigida [taxid 945775]: 3 (0.011%)<br>• Aphanizomenon flos-aquae [taxid 1176]: 3 (0.011%)<br>• Prochlorococcus marinus [taxid 1219]: 2 (0.007%)<br>• Limnothrix rosea [taxid 71188]: 2 (0.007%)<br>• Scleroderma citrinum [taxid 68788]: 2 (0.007%)<br>• Stemphylium lycopersici [taxid 183478]: 1 (0.003%)<br>• other: 35 (0.133%)

Operational Taxonomic Unit (OTU)	Correct identifications	Wrong or overspecific identifications at species rank
Benchmark OTU ID: `CP000388-_Proteobacteria`<br>OTU taxon: Pseudoalteromonas atlantica T6c [taxid 342610]<br>Expected: Pseudoalteromonas atlantica [taxid 288] (species)<br>Number of reads: 10787<br>Number of identified reads: 10722 (99.397%)	• **species: 4302 (39.881%)**<br>• genus: 16 (0.148%)<br>• family: 1 (0.009%)<br>• order: 2280 (21.136%)<br>• class: 964 (8.936%)<br>• phylum: 1673 (15.509%)<br>• superkingdom: 328 (3.04%)<br>• root: 1154 (10.698%)	• Paraglaciecola mesophila [taxid 197222]: 27 (0.25%)<br>• Paraglaciecola polaris [taxid 222814]: 9 (0.083%)<br>• Paraglaciecola hydrolytica [taxid 1799789]: 4 (0.037%)<br>• Alteromonas sediminis [taxid 2259342]: 4 (0.037%)<br>• Paraglaciecola psychrophila [taxid 326544]: 3 (0.027%)<br>• Escherichia coli [taxid 562]: 3 (0.027%)<br>• Parashewanella curva [taxid 2338552]: 2 (0.018%)<br>• Salmonella enterica [taxid 28901]: 2 (0.018%)<br>• other: 62 (0.574%)
Benchmark OTU ID: `CP001796-_Proteobacteria`<br>OTU taxon: Pseudoalteromonas sp. SM9913 [taxid 234831]<br>Expected: Pseudoalteromonas [taxid 53246] (genus)<br>Number of reads: 6615<br>Number of identified reads: 6554 (99.077%)	• **genus: 4800 (72.562%)**<br>• family: 40 (0.604%)<br>• order: 309 (4.671%)<br>• class: 416 (6.288%)<br>• phylum: 141 (2.131%)<br>• superkingdom: 210 (3.174%)<br>• root: 635 (9.599%)	• Pseudoalteromonas tetraodonis [taxid 43659]: 81 (1.224%)<br>• Pseudoalteromonas issachenkonii [taxid 152297]: 38 (0.574%)<br>• Pseudoalteromonas neustonica [taxid 1840331]: 17 (0.256%)<br>• Pseudoalteromonas fuliginea [taxid 1872678]: 8 (0.12%)<br>• Pseudoalteromonas arctica [taxid 394751]: 8 (0.12%)<br>• Pseudoalteromonas sp. JSTW [taxid 2752475]: 5 (0.075%)<br>• Pseudoalteromonas sp. GCY [taxid 2003316]: 4 (0.06%)<br>• other: 59 (0.891%)
Benchmark OTU ID: `CR954246-_Proteobacteria`<br>OTU taxon: Pseudoalteromonas translucida [taxid 166935]<br>Expected: Pseudoalteromonas translucida [taxid 166935] (species)<br>Number of reads: 6350<br>Number of identified reads: 6277 (98.85%)	• species: 594 (9.354%)<br>• **genus: 4117 (64.834%)**<br>• family: 19 (0.299%)<br>• order: 248 (3.905%)<br>• class: 387 (6.094%)<br>• phylum: 118 (1.858%)<br>• superkingdom: 228 (3.59%)<br>• root: 560 (8.818%)	• Pseudoalteromonas nigrifaciens [taxid 28109]: 10 (0.157%)<br>• Pseudoalteromonas phenolica [taxid 161398]: 3 (0.047%)<br>• Pseudoalteromonas tunicata [taxid 314281]: 3 (0.047%)<br>• Pseudoalteromonas arctica [taxid 394751]: 2 (0.031%)<br>• Shewanella piezotolerans [taxid 404011]: 2 (0.031%)<br>• Pseudoalteromonas luteoviolacea [taxid 43657]: 2 (0.031%)<br>• Pseudoalteromonas espejiana [taxid 28107]: 2 (0.031%)<br>• Glaciecola pallidula [taxid 56807]: 1 (0.015%)<br>• other: 21 (0.33%)
Benchmark OTU ID: `AP012224-_Proteobacteria`<br>OTU taxon: Pseudogulbenkiania sp. NH8B [taxid 748280]<br>Expected: Pseudogulbenkiania [taxid 568394] (genus)<br>Number of reads: 8865<br>Number of identified reads: 8826 (99.56%)	• **genus: 5498 (62.019%)**<br>• family: 253 (2.853%)<br>• order: 212 (2.391%)<br>• class: 629 (7.095%)<br>• phylum: 1023 (11.539%)<br>• superkingdom: 391 (4.41%)<br>• root: 815 (9.193%)	• Pseudogulbenkiania subflava [taxid 451637]: 27 (0.304%)<br>• Pseudogulbenkiania ferrooxidans [taxid 549169]: 16 (0.18%)<br>• Nitrosomonas mobilis [taxid 51642]: 3 (0.033%)<br>• Chromobacterium sphagni [taxid 1903179]: 2 (0.022%)<br>• Gulbenkiania indica [taxid 375574]: 2 (0.022%)<br>• Anaerotruncus colihominis [taxid 169435]: 2 (0.022%)<br>• Bathymodiolus azoricus thioautotrophic gill symbiont [taxid 235205]: 2 (0.022%)<br>• other: 43 (0.485%)

Operational Taxonomic Unit (OTU)	Correct identifications	Wrong or overspecific identifications at species rank
Benchmark OTU ID: `CP003149-_Pathogens`<br>OTU taxon: Pseudomonas aeruginosa DK2 [taxid 1093787]<br>Expected: Pseudomonas aeruginosa [taxid 287] (species)<br>Number of reads: 14793<br>Number of identified reads: 14765 (99.81%)	• species: 986 (6.665%)<br>• **genus: 10950 (74.021%)**<br>• family: 51 (0.344%)<br>• order: 43 (0.29%)<br>• class: 652 (4.407%)<br>• phylum: 710 (4.799%)<br>• superkingdom: 511 (3.454%)<br>• root: 848 (5.732%)	• Pseudomonas fluorescens [taxid 294]: 12 (0.081%)<br>• Pseudomonas putida [taxid 303]: 11 (0.074%)<br>• Klebsiella pneumoniae [taxid 573]: 11 (0.074%)<br>• Pseudomonas citronellolis [taxid 53408]: 5 (0.033%)<br>• Pseudomonas syringae [taxid 317]: 5 (0.033%)<br>• Lupinus albus [taxid 3870]: 4 (0.027%)<br>• Pseudomonas kirkiae [taxid 2211392]: 3 (0.02%)<br>• Pseudomonas knackmussii [taxid 65741]: 3 (0.02%)<br>• other: 137 (0.926%)
Benchmark OTU ID: `CP002496-_Pathogens`<br>OTU taxon: Pseudomonas aeruginosa M18 [taxid 941193]<br>Expected: Pseudomonas aeruginosa [taxid 287] (species)<br>Number of reads: 14605<br>Number of identified reads: 14564 (99.719%)	• species: 979 (6.703%)<br>• **genus: 10903 (74.652%)**<br>• family: 29 (0.198%)<br>• order: 31 (0.212%)<br>• class: 642 (4.395%)<br>• phylum: 554 (3.793%)<br>• superkingdom: 532 (3.642%)<br>• root: 869 (5.95%)	• Pseudomonas fluorescens [taxid 294]: 25 (0.171%)<br>• Klebsiella pneumoniae [taxid 573]: 16 (0.109%)<br>• Pseudomonas chlororaphis [taxid 587753]: 6 (0.041%)<br>• Pseudomonas panipatensis [taxid 428992]: 6 (0.041%)<br>• Pseudomonas knackmussii [taxid 65741]: 5 (0.034%)<br>• Pseudomonas putida [taxid 303]: 5 (0.034%)<br>• Pseudomonas resinovorans [taxid 53412]: 4 (0.027%)<br>• Pseudomonas savastanoi [taxid 29438]: 3 (0.02%)<br>• other: 117 (0.801%)
Benchmark OTU ID: `CT573326-_Proteobacteria`<br>OTU taxon: Pseudomonas entomophila L48 [taxid 384676]<br>Expected: Pseudomonas entomophila [taxid 312306] (species)<br>Number of reads: 12366<br>Number of identified reads: 12335 (99.749%)	• species: 4198 (33.947%)<br>• **genus: 6172 (49.911%)**<br>• family: 12 (0.097%)<br>• order: 19 (0.153%)<br>• class: 302 (2.442%)<br>• phylum: 361 (2.919%)<br>• superkingdom: 357 (2.886%)<br>• root: 903 (7.302%)	• Pseudomonas putida [taxid 303]: 60 (0.485%)<br>• Pseudomonas fluorescens [taxid 294]: 22 (0.177%)<br>• Pseudomonas mosselii [taxid 78327]: 6 (0.048%)<br>• Pseudomonas syringae [taxid 317]: 5 (0.04%)<br>• Pseudomonas monteilii [taxid 76759]: 5 (0.04%)<br>• Pseudomonas aeruginosa [taxid 287]: 4 (0.032%)<br>• Lupinus albus [taxid 3870]: 3 (0.024%)<br>• Pseudomonas amygdali [taxid 47877]: 3 (0.024%)<br>• other: 117 (0.946%)
Benchmark OTU ID: `CP003734-_Proteobacteria`<br>OTU taxon: Pseudomonas putida DOT-T1E [taxid 1196325]<br>Expected: Pseudomonas putida [taxid 303] (species)<br>Number of reads: 13202<br>Number of identified reads: 13182 (99.848%)	• species: 1093 (8.279%)<br>• **genus: 9991 (75.677%)**<br>• family: 11 (0.083%)<br>• order: 24 (0.181%)<br>• class: 330 (2.499%)<br>• phylum: 495 (3.749%)<br>• superkingdom: 341 (2.582%)<br>• root: 889 (6.733%)	• Pseudomonas fluorescens [taxid 294]: 19 (0.143%)<br>• Pseudomonas plecoglossicida [taxid 70775]: 18 (0.136%)<br>• Pseudomonas monteilii [taxid 76759]: 11 (0.083%)<br>• Pseudomonas syringae [taxid 317]: 7 (0.053%)<br>• Pseudomonas cremoricolorata [taxid 157783]: 6 (0.045%)<br>• Pseudomonas entomophila [taxid 312306]: 5 (0.037%)<br>• Pseudomonas aeruginosa [taxid 287]: 5 (0.037%)<br>• Pseudomonas amygdali [taxid 47877]: 4 (0.03%)<br>• other: 121 (0.916%)

Operational Taxonomic Unit (OTU)	Correct identifications	Wrong or overspecific identifications at species rank
Benchmark OTU ID: `CP003677-_Proteobacteria`<br>OTU taxon: Pseudomonas stutzeri CCUG 29243 [taxid 1196835]<br>Expected: Pseudomonas stutzeri [taxid 316] (species)<br>Number of reads: 9711<br>Number of identified reads: 9691 (99.794%)	• species: 624 (6.425%)<br>• **genus: 6679 (68.777%)**<br>• family: 66 (0.679%)<br>• order: 235 (2.419%)<br>• class: 547 (5.632%)<br>• phylum: 480 (4.942%)<br>• superkingdom: 309 (3.181%)<br>• root: 750 (7.723%)	• Pseudomonas xanthomarina [taxid 271420]: 50 (0.514%)<br>• Pseudomonas chloritidismutans [taxid 203192]: 9 (0.092%)<br>• Pseudomonas kunmingensis [taxid 1211807]: 7 (0.072%)<br>• Pseudomonas aeruginosa [taxid 287]: 6 (0.061%)<br>• Pseudomonas syringae [taxid 317]: 5 (0.051%)<br>• Pseudomonas kirkiae [taxid 2211392]: 5 (0.051%)<br>• Pseudomonas putida [taxid 303]: 4 (0.041%)<br>• Pseudomonas savastanoi [taxid 29438]: 3 (0.03%)<br>• other: 90 (0.926%)
Benchmark OTU ID: `CP002593-_Actinobacteria`<br>OTU taxon: Pseudonocardia dioxanivorans CB1190 [taxid 675635]<br>Expected: Pseudonocardia dioxanivorans [taxid 240495] (species)<br>Number of reads: 32370<br>Number of identified reads: 32284 (99.734%)	• **species: 17037 (52.632%)**<br>• genus: 2349 (7.256%)<br>• family: 3292 (10.169%)<br>• order: 31 (0.095%)<br>• class: 3807 (11.76%)<br>• phylum: 50 (0.154%)<br>• superkingdom: 2596 (8.019%)<br>• root: 3091 (9.548%)	• Pseudonocardia sulfidoxydans [taxid 54011]: 17 (0.052%)<br>• Amycolatopsis rubida [taxid 112413]: 6 (0.018%)<br>• Pseudonocardia oroxyli [taxid 366584]: 5 (0.015%)<br>• Pseudonocardia asaccharolytica [taxid 54010]: 4 (0.012%)<br>• Pseudonocardia thermophila [taxid 1848]: 4 (0.012%)<br>• Pseudonocardia autotrophica [taxid 2074]: 4 (0.012%)<br>• Actinomycetospora succinea [taxid 663603]: 3 (0.009%)<br>• Pseudonocardia hydrocarbonoxydans [taxid 76726]: 3 (0.009%)<br>• other: 153 (0.472%)
Benchmark OTU ID: `CP002545-_Bacteroidetes`<br>OTU taxon: Pseudopedobacter saltans DSM 12145 [taxid 762903]<br>Expected: Pseudopedobacter saltans [taxid 151895] (species)<br>Number of reads: 31417<br>Number of identified reads: 31055 (98.847%)	• **species: 21646 (68.899%)**<br>• genus: 0 (0.0%)<br>• family: 1694 (5.391%)<br>• order: 31 (0.098%)<br>• class: 9 (0.028%)<br>• phylum: 2761 (8.788%)<br>• superkingdom: 1566 (4.984%)<br>• root: 3332 (10.605%)	• Mucilaginibacter paludis [taxid 423351]: 4 (0.012%)<br>• Pedobacter heparinus [taxid 984]: 3 (0.009%)<br>• Pararcticibacter tournemirensis [taxid 699437]: 3 (0.009%)<br>• Chryseobacterium lacus [taxid 2058346]: 2 (0.006%)<br>• Mucilaginibacter pedocola [taxid 1792845]: 2 (0.006%)<br>• Spinacia oleracea [taxid 3562]: 2 (0.006%)<br>• Mucilaginibacter frigoritolerans [taxid 652788]: 2 (0.006%)<br>• Pseudomonas savastanoi [taxid 29438]: 2 (0.006%)<br>• other: 88 (0.28%)
Benchmark OTU ID: `FO082820-_Rhizobium_Bradyrhizobium`<br>OTU taxon: Pseudorhizobium banfieldiae [taxid 1125847]<br>Expected: Pseudorhizobium banfieldiae [taxid 1125847] (species)<br>Number of reads: 114350<br>Number of identified reads: 114025 (99.715%)	• **species: 58083 (50.794%)**<br>• genus: 1556 (1.36%)<br>• family: 18362 (16.057%)<br>• order: 9059 (7.922%)<br>• class: 5388 (4.711%)<br>• phylum: 3358 (2.936%)<br>• superkingdom: 4571 (3.997%)<br>• root: 13488 (11.795%)	• Lupinus albus [taxid 3870]: 52 (0.045%)<br>• Rhizobium leguminosarum [taxid 384]: 44 (0.038%)<br>• Pseudorhizobium pelagicum [taxid 1509405]: 38 (0.033%)<br>• Rhizobium etli [taxid 29449]: 20 (0.017%)<br>• Agrobacterium tumefaciens [taxid 358]: 20 (0.017%)<br>• Neorhizobium galegae [taxid 399]: 19 (0.016%)<br>• Rhizobium oryzae [taxid 464029]: 17 (0.014%)<br>• Rhizobium tibeticum [taxid 501024]: 10 (0.008%)<br>• other: 568 (0.496%)

Operational Taxonomic Unit (OTU)	Correct identifications	Wrong or overspecific identifications at species rank
Benchmark OTU ID: `CP003147-_Proteobacteria`<br>OTU taxon: Pseudovibrio sp. FO-BEG1 [taxid 911045]<br>Expected: Pseudovibrio [taxid 258255] (genus)<br>Number of reads: 11436<br>Number of identified reads: 11410 (99.772%)	• **genus: 8099 (70.82%)**<br>• family: 219 (1.915%)<br>• order: 7 (0.061%)<br>• class: 881 (7.703%)<br>• phylum: 349 (3.051%)<br>• superkingdom: 374 (3.27%)<br>• root: 1457 (12.74%)	• Pseudovibrio denitrificans [taxid 258256]: 82 (0.717%)<br>• Pseudovibrio axinellae [taxid 989403]: 15 (0.131%)<br>• Pseudomonas meliae [taxid 86176]: 3 (0.026%)<br>• Mikania micrantha [taxid 192012]: 2 (0.017%)<br>• Roseibium hamelinense [taxid 150831]: 2 (0.017%)<br>• Pararhodospirillum photometricum [taxid 1084]: 2 (0.017%)<br>• Nesiotobacter exalbescens [taxid 197461]: 2 (0.017%)<br>• other: 46 (0.402%)
Benchmark OTU ID: `CP002446-_Proteobacteria`<br>OTU taxon: Pseudoxanthomonas suwonensis 11-1 [taxid 743721]<br>Expected: Pseudoxanthomonas suwonensis [taxid 314722] (species)<br>Number of reads: 6809<br>Number of identified reads: 6771 (99.441%)	• **species: 3581 (52.592%)**<br>• genus: 642 (9.428%)<br>• family: 992 (14.568%)<br>• order: 119 (1.747%)<br>• class: 193 (2.834%)<br>• phylum: 349 (5.125%)<br>• superkingdom: 387 (5.683%)<br>• root: 500 (7.343%)	• Stenotrophomonas maltophilia [taxid 40324]: 5 (0.073%)<br>• Stenotrophomonas panacihumi [taxid 676599]: 2 (0.029%)<br>• Pseudolysobacter antarcticus [taxid 2511995]: 2 (0.029%)<br>• Xylella fastidiosa [taxid 2371]: 2 (0.029%)<br>• Brugia timori [taxid 42155]: 2 (0.029%)<br>• Pseudoxanthomonas taiwanensis [taxid 176598]: 2 (0.029%)<br>• Xanthomonas oryzae [taxid 347]: 1 (0.014%)<br>• Haemophilus haemolyticus [taxid 726]: 1 (0.014%)<br>• other: 24 (0.352%)
Benchmark OTU ID: `CP000323-_Proteobacteria`<br>OTU taxon: Psychrobacter cryohalolentis K5 [taxid 335284]<br>Expected: Psychrobacter cryohalolentis [taxid 330922] (species)<br>Number of reads: 6001<br>Number of identified reads: 5938 (98.95%)	• species: 843 (14.047%)<br>• **genus: 3839 (63.972%)**<br>• family: 146 (2.432%)<br>• order: 27 (0.449%)<br>• class: 162 (2.699%)<br>• phylum: 164 (2.732%)<br>• superkingdom: 177 (2.949%)<br>• root: 576 (9.598%)	• Psychrobacter sp. G [taxid 571800]: 33 (0.549%)<br>• Psychrobacter arcticus [taxid 334543]: 6 (0.099%)<br>• Psychrobacter frigidicola [taxid 45611]: 5 (0.083%)<br>• Psychrobacter aquaticus [taxid 248452]: 4 (0.066%)<br>• Psychrobacter fozii [taxid 198480]: 3 (0.049%)<br>• Psychrobacter urativorans [taxid 45610]: 3 (0.049%)<br>• Psychrobacter phenylpyruvicus [taxid 29432]: 2 (0.033%)<br>• Escherichia coli [taxid 562]: 2 (0.033%)<br>• other: 16 (0.266%)
Benchmark OTU ID: `CP003879-_Bacteroidetes`<br>OTU taxon: Psychroflexus torquis ATCC 700755 [taxid 313595]<br>Expected: Psychroflexus torquis [taxid 57029] (species)<br>Number of reads: 29035<br>Number of identified reads: 28406 (97.833%)	• **species: 12173 (41.925%)**<br>• genus: 7952 (27.387%)<br>• family: 1933 (6.657%)<br>• order: 301 (1.036%)<br>• class: 8 (0.027%)<br>• phylum: 865 (2.979%)<br>• superkingdom: 1119 (3.853%)<br>• root: 4035 (13.897%)	• Psychroflexus gondwanensis [taxid 251]: 101 (0.347%)<br>• Psychroflexus sediminis [taxid 470826]: 15 (0.051%)<br>• Psychroserpens burtonensis [taxid 49278]: 7 (0.024%)<br>• Subsaximicrobium wynnwilliamsii [taxid 291179]: 3 (0.01%)<br>• Lupinus albus [taxid 3870]: 3 (0.01%)<br>• Psychroflexus salarius [taxid 1155689]: 3 (0.01%)<br>• Flavobacterium sandaracinum [taxid 2541733]: 2 (0.006%)<br>• Cyclobacterium qasimii [taxid 1350429]: 2 (0.006%)<br>• other: 86 (0.296%)

Operational Taxonomic Unit (OTU)	Correct identifications	Wrong or overspecific identifications at species rank
Benchmark OTU ID: `CP000510-_Proteobacteria`<br>OTU taxon: Psychromonas ingrahamii 37 [taxid 357804]<br>Expected: Psychromonas ingrahamii [taxid 357794] (species)<br>Number of reads: 9375<br>Number of identified reads: 9214 (98.282%)	• **species: 4795 (51.146%)**<br>• genus: 1307 (13.941%)<br>• family: 0 (0.0%)<br>• order: 189 (2.016%)<br>• class: 795 (8.48%)<br>• phylum: 254 (2.709%)<br>• superkingdom: 300 (3.2%)<br>• root: 1568 (16.725%)	• Candidatus Photodesmus katoptron [taxid 28176]: 2 (0.021%)<br>• Tepidiphilus thermophilus [taxid 876478]: 1 (0.01%)<br>• Enterocloster aldensis [taxid 358742]: 1 (0.01%)<br>• Enterobacter kobei [taxid 208224]: 1 (0.01%)<br>• Candidatus Hakubanella thermoalkaliphilus [taxid 2754717]: 1 (0.01%)<br>• Muribacter muris [taxid 67855]: 1 (0.01%)<br>• Candidatus Enterovibrio escacola [taxid 1927127]: 1 (0.01%)<br>• Desulfobacula phenolica [taxid 90732]: 1 (0.01%)<br>• other: 22 (0.234%)
Benchmark OTU ID: `CP002663-_Proteobacteria`<br>OTU taxon: Pusillimonas sp. T7-7 [taxid 1007105]<br>Expected: Pusillimonas [taxid 305976] (genus)<br>Number of reads: 7854<br>Number of identified reads: 7843 (99.859%)	• **genus: 4762 (60.631%)**<br>• family: 693 (8.823%)<br>• order: 292 (3.717%)<br>• class: 97 (1.235%)<br>• phylum: 716 (9.116%)<br>• superkingdom: 341 (4.341%)<br>• root: 940 (11.968%)	• Pusillimonas thiosulfatoxidans [taxid 2028345]: 11 (0.14%)<br>• Pusillimonas ginsengisoli [taxid 453575]: 6 (0.076%)<br>• Alcaligenes aquatilis [taxid 323284]: 2 (0.025%)<br>• Eoetvoesia caeni [taxid 645616]: 2 (0.025%)<br>• Pseudomonas fluorescens [taxid 294]: 2 (0.025%)<br>• Ramlibacter tataouinensis [taxid 94132]: 1 (0.012%)<br>• Advenella kashmirensis [taxid 310575]: 1 (0.012%)<br>• other: 25 (0.318%)
Benchmark OTU ID: `AE009441-_Crenarchaeota`<br>OTU taxon: Pyrobaculum aerophilum str. IM2 [taxid 178306]<br>Expected: Pyrobaculum aerophilum [taxid 13773] (species)<br>Number of reads: 4681<br>Number of identified reads: 4577 (97.778%)	• **species: 2654 (56.697%)**<br>• genus: 390 (8.331%)<br>• family: 50 (1.068%)<br>• order: 3 (0.064%)<br>• class: 22 (0.469%)<br>• phylum: 0 (0.0%)<br>• superkingdom: 23 (0.491%)<br>• root: 1383 (29.544%)	• Pyrobaculum ferrireducens [taxid 1104324]: 3 (0.064%)<br>• Pyrobaculum islandicum [taxid 2277]: 2 (0.042%)<br>• Fusarium oxysporum [taxid 5507]: 1 (0.021%)<br>• Gregarina niphandrodes [taxid 110365]: 1 (0.021%)<br>• Pyrobaculum calidifontis [taxid 181486]: 1 (0.021%)<br>• Salinivirga cyanobacteriivorans [taxid 1307839]: 1 (0.021%)<br>• Thermoproteus tenax [taxid 2271]: 1 (0.021%)<br>• Thermoprotei archaeon [taxid 2250277]: 1 (0.021%)<br>• other: 5 (0.106%)
Benchmark OTU ID: `CP000660-_Crenarchaeota`<br>OTU taxon: Pyrobaculum arsenaticum DSM 13514 [taxid 340102]<br>Expected: Pyrobaculum arsenaticum [taxid 121277] (species)<br>Number of reads: 4428<br>Number of identified reads: 4336 (97.922%)	• species: 1199 (27.077%)<br>• **genus: 1618 (36.54%)**<br>• family: 54 (1.219%)<br>• order: 2 (0.045%)<br>• class: 20 (0.451%)<br>• phylum: 0 (0.0%)<br>• superkingdom: 22 (0.496%)<br>• root: 1343 (30.329%)	• Pyrobaculum oguniense [taxid 99007]: 50 (1.129%)<br>• Pyrobaculum aerophilum [taxid 13773]: 9 (0.203%)<br>• Pyrobaculum ferrireducens [taxid 1104324]: 4 (0.09%)<br>• Pyrobaculum islandicum [taxid 2277]: 2 (0.045%)<br>• Abditibacterium utsteinense [taxid 1960156]: 1 (0.022%)<br>• Scyliorhinus torazame [taxid 75743]: 1 (0.022%)<br>• Altererythrobacter atlanticus [taxid 1267766]: 1 (0.022%)<br>• Komagataeibacter intermedius [taxid 66229]: 1 (0.022%)<br>• other: 7 (0.158%)

Operational Taxonomic Unit (OTU)	Correct identifications	Wrong or overspecific identifications at species rank
Benchmark OTU ID: `CP000561-_Crenarchaeota`<br>OTU taxon: Pyrobaculum calidifontis JCM 11548 [taxid 410359]<br>Expected: Pyrobaculum calidifontis [taxid 181486] (species)<br>Number of reads: 4148<br>Number of identified reads: 4040 (97.396%)	• **species: 2335 (56.292%)**<br>• genus: 252 (6.075%)<br>• family: 55 (1.325%)<br>• order: 1 (0.024%)<br>• class: 13 (0.313%)<br>• phylum: 0 (0.0%)<br>• superkingdom: 26 (0.626%)<br>• root: 1293 (31.171%)	• Ordospora colligata [taxid 174685]: 1 (0.024%)<br>• Pyrobaculum aerophilum [taxid 13773]: 1 (0.024%)<br>• Flavobacterium johnsoniae [taxid 986]: 1 (0.024%)<br>• Thermoprotei archaeon [taxid 2250277]: 1 (0.024%)<br>• Branchiostoma floridae [taxid 7739]: 1 (0.024%)<br>• Pyrobaculum oguniense [taxid 99007]: 1 (0.024%)<br>• Methanonatronarchaeia archaeon [taxid 2099682]: 1 (0.024%)<br>• Candidatus Korarchaeota archaeon [taxid 2056630]: 1 (0.024%)<br>• other: 5 (0.12%)
Benchmark OTU ID: `CP003098-_Crenarchaeota`<br>OTU taxon: Pyrobaculum ferrireducens [taxid 1104324]<br>Expected: Pyrobaculum ferrireducens [taxid 1104324] (species)<br>Number of reads: 5295<br>Number of identified reads: 5172 (97.677%)	• **species: 2924 (55.221%)**<br>• genus: 347 (6.553%)<br>• family: 89 (1.68%)<br>• order: 3 (0.056%)<br>• class: 17 (0.321%)<br>• phylum: 0 (0.0%)<br>• superkingdom: 30 (0.566%)<br>• root: 1657 (31.293%)	• Pyrobaculum neutrophilum [taxid 70771]: 6 (0.113%)<br>• Thermoproteus uzoniensis [taxid 184117]: 4 (0.075%)<br>• Pyrobaculum aerophilum [taxid 13773]: 4 (0.075%)<br>• Pyrobaculum islandicum [taxid 2277]: 2 (0.037%)<br>• Paramuribaculum intestinale [taxid 2094151]: 1 (0.018%)<br>• actinobacterium acAcidi [taxid 1504320]: 1 (0.018%)<br>• Euryarchaeota archaeon [taxid 2026739]: 1 (0.018%)<br>• Minutocellus polymorphus [taxid 265543]: 1 (0.018%)<br>• other: 5 (0.094%)
Benchmark OTU ID: `CP000504-_Crenarchaeota`<br>OTU taxon: Pyrobaculum islandicum DSM 4184 [taxid 384616]<br>Expected: Pyrobaculum islandicum [taxid 2277] (species)<br>Number of reads: 3691<br>Number of identified reads: 3614 (97.913%)	• **species: 1935 (52.424%)**<br>• genus: 432 (11.704%)<br>• family: 62 (1.679%)<br>• order: 0 (0.0%)<br>• class: 12 (0.325%)<br>• phylum: 0 (0.0%)<br>• superkingdom: 14 (0.379%)<br>• root: 1110 (30.073%)	• Pyrobaculum neutrophilum [taxid 70771]: 7 (0.189%)<br>• Pyrobaculum ferrireducens [taxid 1104324]: 3 (0.081%)<br>• Pyrobaculum aerophilum [taxid 13773]: 2 (0.054%)<br>• Pyrobaculum calidifontis [taxid 181486]: 2 (0.054%)<br>• Polarella glacialis [taxid 89957]: 1 (0.027%)<br>• Photobacterium profundum [taxid 74109]: 1 (0.027%)<br>• Strongyloides ratti [taxid 34506]: 1 (0.027%)<br>• Thermoproteus tenax [taxid 2271]: 1 (0.027%)
Benchmark OTU ID: `CP001014-_Crenarchaeota`<br>OTU taxon: Pyrobaculum neutrophilum V24Sta [taxid 444157]<br>Expected: Pyrobaculum neutrophilum [taxid 70771] (species)<br>Number of reads: 3550<br>Number of identified reads: 3463 (97.549%)	• **species: 1682 (47.38%)**<br>• genus: 419 (11.802%)<br>• family: 90 (2.535%)<br>• order: 0 (0.0%)<br>• class: 15 (0.422%)<br>• phylum: 0 (0.0%)<br>• superkingdom: 18 (0.507%)<br>• root: 1139 (32.084%)	• Pyrobaculum islandicum [taxid 2277]: 13 (0.366%)<br>• Thermoproteus tenax [taxid 2271]: 3 (0.084%)<br>• Pyrobaculum ferrireducens [taxid 1104324]: 2 (0.056%)<br>• Thermoprotei archaeon [taxid 2250277]: 1 (0.028%)<br>• Haemonchus contortus [taxid 6289]: 1 (0.028%)<br>• Corynebacterium marinum [taxid 349751]: 1 (0.028%)<br>• Chondromyces apiculatus [taxid 51]: 1 (0.028%)<br>• Chelonus inanitus bracovirus [taxid 36344]: 1 (0.028%)<br>• other: 5 (0.14%)

Operational Taxonomic Unit (OTU)	Correct identifications	Wrong or overspecific identifications at species rank
Benchmark OTU ID: `CP003316-_Crenarchaeota`<br>OTU taxon: Pyrobaculum oguniense TE7 [taxid 698757]<br>Expected: Pyrobaculum oguniense [taxid 99007] (species)<br>Number of reads: 5215<br>Number of identified reads: 5107 (97.929%)	• **species: 1681 (32.233%)**<br>• genus: 1641 (31.466%)<br>• family: 71 (1.361%)<br>• order: 1 (0.019%)<br>• class: 22 (0.421%)<br>• phylum: 1 (0.019%)<br>• superkingdom: 24 (0.46%)<br>• root: 1586 (30.412%)	• Pyrobaculum arsenaticum [taxid 121277]: 30 (0.575%)<br>• Pyrobaculum aerophilum [taxid 13773]: 7 (0.134%)<br>• Pyrobaculum neutrophilum [taxid 70771]: 5 (0.095%)<br>• Pyrobaculum calidifontis [taxid 181486]: 2 (0.038%)<br>• Pyrobaculum islandicum [taxid 2277]: 1 (0.019%)<br>• Jiangella anatolica [taxid 2670374]: 1 (0.019%)<br>• Thalassoglobus polymorphus [taxid 2527994]: 1 (0.019%)<br>• Stentor coeruleus [taxid 5963]: 1 (0.019%)<br>• other: 6 (0.115%)
Benchmark OTU ID: `CP003685-_Euryarchaeota`<br>OTU taxon: Pyrococcus furiosus COM1 [taxid 1185654]<br>Expected: Pyrococcus furiosus [taxid 2261] (species)<br>Number of reads: 1608<br>Number of identified reads: 1584 (98.507%)	• **species: 877 (54.539%)**<br>• genus: 79 (4.912%)<br>• family: 127 (7.898%)<br>• order: 10 (0.621%)<br>• class: 61 (3.793%)<br>• phylum: 3 (0.186%)<br>• superkingdom: 16 (0.995%)<br>• root: 395 (24.564%)	• Thermococcus celer [taxid 2264]: 1 (0.062%)<br>• Thermococcus paralvinellae [taxid 582419]: 1 (0.062%)<br>• Pyrococcus horikoshii [taxid 53953]: 1 (0.062%)<br>• Thermococcus thioreducens [taxid 277988]: 1 (0.062%)<br>• Pyrococcus kukulkanii [taxid 1609559]: 1 (0.062%)
Benchmark OTU ID: `AE009950-_Euryarchaeota`<br>OTU taxon: Pyrococcus furiosus DSM 3638 [taxid 186497]<br>Expected: Pyrococcus furiosus [taxid 2261] (species)<br>Number of reads: 1607<br>Number of identified reads: 1588 (98.817%)	• **species: 896 (55.756%)**<br>• genus: 80 (4.978%)<br>• family: 125 (7.778%)<br>• order: 4 (0.248%)<br>• class: 63 (3.92%)<br>• phylum: 11 (0.684%)<br>• superkingdom: 27 (1.68%)<br>• root: 368 (22.899%)	• Thermococci archaeon [taxid 2250254]: 2 (0.124%)<br>• Thermoplasma acidophilum [taxid 2303]: 1 (0.062%)<br>• Methanosalsum zhilinae [taxid 39669]: 1 (0.062%)<br>• Pyrococcus horikoshii [taxid 53953]: 1 (0.062%)<br>• Thermococcus gorgonarius [taxid 71997]: 1 (0.062%)<br>• Thermococcus profundus [taxid 49899]: 1 (0.062%)<br>• Flavivirga eckloniae [taxid 1803846]: 1 (0.062%)<br>• Thermococcus litoralis [taxid 2265]: 1 (0.062%)
Benchmark OTU ID: `BA000001-_Euryarchaeota`<br>OTU taxon: Pyrococcus horikoshii OT3 [taxid 70601]<br>Expected: Pyrococcus horikoshii [taxid 53953] (species)<br>Number of reads: 1428<br>Number of identified reads: 1404 (98.319%)	• **species: 788 (55.182%)**<br>• genus: 73 (5.112%)<br>• family: 96 (6.722%)<br>• order: 3 (0.21%)<br>• class: 61 (4.271%)<br>• phylum: 8 (0.56%)<br>• superkingdom: 14 (0.98%)<br>• root: 344 (24.089%)	• Thermococcus siculi [taxid 72803]: 1 (0.07%)<br>• Zestosphaera tikiterensis [taxid 1973259]: 1 (0.07%)<br>• Cajanus cajan [taxid 3821]: 1 (0.07%)<br>• Pyrococcus abyssi [taxid 29292]: 1 (0.07%)<br>• Thermococcus eurythermalis [taxid 1505907]: 1 (0.07%)<br>• Caenorhabditis briggsae [taxid 6238]: 1 (0.07%)
Benchmark OTU ID: `CP003534-_Euryarchaeota`<br>OTU taxon: Pyrococcus sp. ST04 [taxid 1183377]<br>Expected: Pyrococcus [taxid 2260] (genus)<br>Number of reads: 1427<br>Number of identified reads: 1409 (98.738%)	• **genus: 741 (51.927%)**<br>• family: 142 (9.95%)<br>• order: 2 (0.14%)<br>• class: 89 (6.236%)<br>• phylum: 18 (1.261%)<br>• superkingdom: 19 (1.331%)<br>• root: 369 (25.858%)	• Pyrococcus horikoshii [taxid 53953]: 4 (0.28%)<br>• Thermococcus cleftensis [taxid 163003]: 1 (0.07%)<br>• Euryarchaeota archaeon [taxid 2026739]: 1 (0.07%)<br>• Thermococcus chitonophagus [taxid 54262]: 1 (0.07%)<br>• Thermococcus paralvinellae [taxid 582419]: 1 (0.07%)<br>• Thermococcus guaymasensis [taxid 110164]: 1 (0.07%)<br>• Pleurotus ostreatus [taxid 5322]: 1 (0.07%)<br>• other: 3 (0.21%)

Operational Taxonomic Unit (OTU)	Correct identifications	Wrong or overspecific identifications at species rank
Benchmark OTU ID: `CP002838-_Crenarchaeota`<br>OTU taxon: Pyrolobus fumarii 1A [taxid 694429]<br>Expected: Pyrolobus fumarii [taxid 54252] (species)<br>Number of reads: 3733<br>Number of identified reads: 3635 (97.374%)	• **species: 2271 (60.835%)**<br>• genus: 0 (0.0%)<br>• family: 17 (0.455%)<br>• order: 11 (0.294%)<br>• class: 34 (0.91%)<br>• phylum: 0 (0.0%)<br>• superkingdom: 32 (0.857%)<br>• root: 1206 (32.306%)	• Candidatus Aegiribacteria sp. MLS_C [taxid 1775674]: 1 (0.026%)<br>• Candidatus Bathyarchaeota archaeon [taxid 2026714]: 1 (0.026%)<br>• Cervus elaphus [taxid 9860]: 1 (0.026%)<br>• Thermoprotei archaeon [taxid 2250277]: 1 (0.026%)<br>• Salibacterium qingdaonense [taxid 266892]: 1 (0.026%)<br>• Ignicoccus islandicus [taxid 54259]: 1 (0.026%)<br>• Nocardioides jejuensis [taxid 2502782]: 1 (0.026%)<br>• Frankia canadensis [taxid 1836972]: 1 (0.026%)<br>• other: 2 (0.053%)
Benchmark OTU ID: `CP002505-_Proteobacteria`<br>OTU taxon: Rahnella sp. Y9602 [taxid 741091]<br>Expected: Rahnella [taxid 34037] (genus)<br>Number of reads: 10060<br>Number of identified reads: 10043 (99.831%)	• **genus: 4130 (41.053%)**<br>• family: 614 (6.103%)<br>• order: 3312 (32.922%)<br>• class: 431 (4.284%)<br>• phylum: 265 (2.634%)<br>• superkingdom: 283 (2.813%)<br>• root: 1003 (9.97%)	• Rahnella aquatilis [taxid 34038]: 60 (0.596%)<br>• Salmonella enterica [taxid 28901]: 45 (0.447%)<br>• Escherichia coli [taxid 562]: 19 (0.188%)<br>• Rahnella sp. AA [taxid 2057180]: 18 (0.178%)<br>• Ewingella americana [taxid 41202]: 9 (0.089%)<br>• Serratia symbiotica [taxid 138074]: 7 (0.069%)<br>• Rahnella woolbedingensis [taxid 1510574]: 7 (0.069%)<br>• other: 110 (1.093%)
Benchmark OTU ID: `FP885895-_Proteobacteria`<br>OTU taxon: Ralstonia solanacearum CMR15 [taxid 859655]<br>Expected: Ralstonia solanacearum [taxid 305] (species)<br>Number of reads: 7203<br>Number of identified reads: 7180 (99.68%)	• species: 2131 (29.584%)<br>• **genus: 2880 (39.983%)**<br>• family: 513 (7.122%)<br>• order: 315 (4.373%)<br>• class: 100 (1.388%)<br>• phylum: 414 (5.747%)<br>• superkingdom: 241 (3.345%)<br>• root: 579 (8.038%)	• Ralstonia syzygii [taxid 28097]: 5 (0.069%)<br>• Cupriavidus taiwanensis [taxid 164546]: 5 (0.069%)<br>• Ralstonia insidiosa [taxid 190721]: 2 (0.027%)<br>• Psychromonas ingrahamii [taxid 357794]: 1 (0.013%)<br>• Pseudoxanthomonas indica [taxid 428993]: 1 (0.013%)<br>• Nitrosospira lacus [taxid 1288494]: 1 (0.013%)<br>• Ralstonia pickettii [taxid 329]: 1 (0.013%)<br>• Variovorax paradoxus [taxid 34073]: 1 (0.013%)<br>• other: 15 (0.208%)
Benchmark OTU ID: `CP000245-_Proteobacteria`<br>OTU taxon: Ramlibacter tataouinensis TTB310 [taxid 365046]<br>Expected: Ramlibacter tataouinensis [taxid 94132] (species)<br>Number of reads: 8274<br>Number of identified reads: 8243 (99.625%)	• **species: 4987 (60.273%)**<br>• genus: 138 (1.667%)<br>• family: 661 (7.988%)<br>• order: 817 (9.874%)<br>• class: 141 (1.704%)<br>• phylum: 507 (6.127%)<br>• superkingdom: 429 (5.184%)<br>• root: 557 (6.731%)	• Limnohabitans planktonicus [taxid 540060]: 2 (0.024%)<br>• Giesbergeria anulus [taxid 180197]: 1 (0.012%)<br>• Clostridium amylolyticum [taxid 1121298]: 1 (0.012%)<br>• Denitromonas halophilus [taxid 1629404]: 1 (0.012%)<br>• Mesorhizobium amorphae [taxid 71433]: 1 (0.012%)<br>• Pristionchus pacificus [taxid 54126]: 1 (0.012%)<br>• Xylophilus ampelinus [taxid 54067]: 1 (0.012%)<br>• Saezia sanguinis [taxid 1965230]: 1 (0.012%)<br>• other: 22 (0.265%)

Operational Taxonomic Unit (OTU)	Correct identifications	Wrong or overspecific identifications at species rank
Benchmark OTU ID: `CP004142-_Proteobacteria`<br>OTU taxon: Raoultella ornithinolytica B6 [taxid 1286170]<br>Expected: Raoultella ornithinolytica [taxid 54291] (species)<br>Number of reads: 11262<br>Number of identified reads: 11225 (99.671%)	• species: 1139 (10.113%)<br>• genus: 2530 (22.464%)<br>• **family: 4747 (42.15%)**<br>• order: 928 (8.24%)<br>• class: 338 (3.001%)<br>• phylum: 244 (2.166%)<br>• superkingdom: 326 (2.894%)<br>• root: 966 (8.577%)	• Salmonella enterica [taxid 28901]: 144 (1.278%)<br>• Escherichia coli [taxid 562]: 87 (0.772%)<br>• Klebsiella pneumoniae [taxid 573]: 80 (0.71%)<br>• Raoultella planticola [taxid 575]: 56 (0.497%)<br>• Raoultella terrigena [taxid 577]: 12 (0.106%)<br>• Klebsiella variicola [taxid 244366]: 9 (0.079%)<br>• Klebsiella michiganensis [taxid 1134687]: 9 (0.079%)<br>• Klebsiella quasipneumoniae [taxid 1463165]: 7 (0.062%)<br>• other: 145 (1.287%)
Benchmark OTU ID: `CP000133-_Rhizobium_Bradyrhizobium`<br>OTU taxon: Rhizobium etli CFN 42 [taxid 347834]<br>Expected: Rhizobium etli [taxid 29449] (species)<br>Number of reads: 118607<br>Number of identified reads: 118320 (99.758%)	• species: 21038 (17.737%)<br>• **genus: 62535 (52.724%)**<br>• family: 7603 (6.41%)<br>• order: 6100 (5.143%)<br>• class: 2668 (2.249%)<br>• phylum: 2586 (2.18%)<br>• superkingdom: 3900 (3.288%)<br>• root: 11738 (9.896%)	• Rhizobium leguminosarum [taxid 384]: 803 (0.677%)<br>• Rhizobium phaseoli [taxid 396]: 122 (0.102%)<br>• Rhizobium aethiopicum [taxid 1138170]: 86 (0.072%)<br>• Rhizobium laguerreae [taxid 1076926]: 77 (0.064%)<br>• Rhizobium esperanzae [taxid 1967781]: 59 (0.049%)<br>• Lupinus albus [taxid 3870]: 52 (0.043%)<br>• Rhizobium sullae [taxid 50338]: 46 (0.038%)<br>• Rhizobium pisi [taxid 574561]: 42 (0.035%)<br>• other: 784 (0.661%)
Benchmark OTU ID: `CP001074-_Rhizobium_Bradyrhizobium`<br>OTU taxon: Rhizobium etli CIAT 652 [taxid 491916]<br>Expected: Rhizobium etli [taxid 29449] (species)<br>Number of reads: 122558<br>Number of identified reads: 122248 (99.747%)	• species: 8036 (6.556%)<br>• **genus: 80446 (65.639%)**<br>• family: 7296 (5.953%)<br>• order: 5995 (4.891%)<br>• class: 2684 (2.189%)<br>• phylum: 2557 (2.086%)<br>• superkingdom: 4031 (3.289%)<br>• root: 11054 (9.019%)	• Rhizobium leguminosarum [taxid 384]: 1080 (0.881%)<br>• Rhizobium phaseoli [taxid 396]: 766 (0.625%)<br>• Rhizobium laguerreae [taxid 1076926]: 82 (0.066%)<br>• Rhizobium aethiopicum [taxid 1138170]: 65 (0.053%)<br>• Rhizobium sophoriradicis [taxid 1535245]: 52 (0.042%)<br>• Lupinus albus [taxid 3870]: 52 (0.042%)<br>• Agrobacterium vitis [taxid 373]: 50 (0.04%)<br>• Rhizobium esperanzae [taxid 1967781]: 48 (0.039%)<br>• other: 919 (0.749%)
Benchmark OTU ID: `CP001622-_Rhizobium_Bradyrhizobium`<br>OTU taxon: Rhizobium leguminosarum bv. trifolii WSM1325 [taxid 395491]<br>Expected: Rhizobium leguminosarum [taxid 384] (species)<br>Number of reads: 130169<br>Number of identified reads: 129873 (99.772%)	• species: 25893 (19.891%)<br>• **genus: 68878 (52.914%)**<br>• family: 6684 (5.134%)<br>• order: 5950 (4.57%)<br>• class: 2671 (2.051%)<br>• phylum: 2815 (2.162%)<br>• superkingdom: 4265 (3.276%)<br>• root: 12590 (9.672%)	• Rhizobium etli [taxid 29449]: 409 (0.314%)<br>• Rhizobium laguerreae [taxid 1076926]: 243 (0.186%)<br>• Rhizobium phaseoli [taxid 396]: 74 (0.056%)<br>• Rhizobium vallis [taxid 634290]: 44 (0.033%)<br>• Rhizobium pisi [taxid 574561]: 42 (0.032%)<br>• Lupinus albus [taxid 3870]: 42 (0.032%)<br>• Rhizobium sullae [taxid 50338]: 39 (0.029%)<br>• Rhizobium grahamii [taxid 1120045]: 38 (0.029%)<br>• other: 786 (0.603%)

Operational Taxonomic Unit (OTU)	Correct identifications	Wrong or overspecific identifications at species rank
Benchmark OTU ID: `CP001191-_Rhizobium_Bradyrhizobium`<br>OTU taxon: Rhizobium leguminosarum bv. trifolii WSM2304 [taxid 395492]<br>Expected: Rhizobium leguminosarum [taxid 384] (species)<br>Number of reads: 123296<br>Number of identified reads: 123006 (99.764%)	• species: 14341 (11.631%)<br>• **genus: 74900 (60.748%)**<br>• family: 7260 (5.888%)<br>• order: 5682 (4.608%)<br>• class: 2651 (2.15%)<br>• phylum: 2543 (2.062%)<br>• superkingdom: 3996 (3.24%)<br>• root: 11476 (9.307%)	• Rhizobium etli [taxid 29449]: 559 (0.453%)<br>• Rhizobium acidisoli [taxid 1538158]: 137 (0.111%)<br>• Rhizobium laguerreae [taxid 1076926]: 90 (0.072%)<br>• Rhizobium phaseoli [taxid 396]: 83 (0.067%)<br>• Rhizobium pisi [taxid 574561]: 75 (0.06%)<br>• Rhizobium aethiopicum [taxid 1138170]: 51 (0.041%)<br>• Lupinus albus [taxid 3870]: 48 (0.038%)<br>• Rhizobium sullae [taxid 50338]: 43 (0.034%)<br>• other: 874 (0.708%)
Benchmark OTU ID: `AM236080-_Rhizobium_Bradyrhizobium`<br>OTU taxon: Rhizobium leguminosarum bv. viciae 3841 [taxid 216596]<br>Expected: Rhizobium leguminosarum [taxid 384] (species)<br>Number of reads: 138870<br>Number of identified reads: 138559 (99.776%)	• species: 26843 (19.329%)<br>• **genus: 73656 (53.039%)**<br>• family: 7551 (5.437%)<br>• order: 6647 (4.786%)<br>• class: 2790 (2.009%)<br>• phylum: 2973 (2.14%)<br>• superkingdom: 4363 (3.141%)<br>• root: 13592 (9.787%)	• Rhizobium etli [taxid 29449]: 422 (0.303%)<br>• Rhizobium laguerreae [taxid 1076926]: 261 (0.187%)<br>• Rhizobium phaseoli [taxid 396]: 60 (0.043%)<br>• Rhizobium aethiopicum [taxid 1138170]: 51 (0.036%)<br>• Rhizobium pisi [taxid 574561]: 49 (0.035%)<br>• Rhizobium sullae [taxid 50338]: 45 (0.032%)<br>• Rhizobium vallis [taxid 634290]: 45 (0.032%)<br>• Lupinus albus [taxid 3870]: 43 (0.03%)<br>• other: 864 (0.622%)
Benchmark OTU ID: `CP004015-_Rhizobium_Bradyrhizobium`<br>OTU taxon: Rhizobium tropici CIAT 899 [taxid 698761]<br>Expected: Rhizobium tropici [taxid 398] (species)<br>Number of reads: 102269<br>Number of identified reads: 102098 (99.832%)	• species: 324 (0.316%)<br>• **genus: 62605 (61.216%)**<br>• family: 13041 (12.751%)<br>• order: 6197 (6.059%)<br>• class: 2749 (2.688%)<br>• phylum: 2544 (2.487%)<br>• superkingdom: 3369 (3.294%)<br>• root: 11130 (10.883%)	• Rhizobium freirei [taxid 1353277]: 303 (0.296%)<br>• Rhizobium lusitanum [taxid 293958]: 212 (0.207%)<br>• Rhizobium leguminosarum [taxid 384]: 143 (0.139%)<br>• Rhizobium hainanense [taxid 52131]: 74 (0.072%)<br>• Rhizobium multihospitium [taxid 410764]: 72 (0.07%)<br>• Rhizobium miluonense [taxid 411945]: 61 (0.059%)<br>• Rhizobium etli [taxid 29449]: 58 (0.056%)<br>• Lupinus albus [taxid 3870]: 47 (0.045%)<br>• other: 744 (0.727%)
Benchmark OTU ID: `CP003470-_Proteobacteria`<br>OTU taxon: Rhodanobacter denitrificans [taxid 666685]<br>Expected: Rhodanobacter denitrificans [taxid 666685] (species)<br>Number of reads: 8623<br>Number of identified reads: 8588 (99.594%)	• **species: 3297 (38.234%)**<br>• genus: 2174 (25.211%)<br>• family: 781 (9.057%)<br>• order: 340 (3.942%)<br>• class: 284 (3.293%)<br>• phylum: 498 (5.775%)<br>• superkingdom: 473 (5.485%)<br>• root: 729 (8.454%)	• Rhodanobacter thiooxydans [taxid 416169]: 6 (0.069%)<br>• Mizugakiibacter sediminis [taxid 1475481]: 4 (0.046%)<br>• Rhodanobacter lindaniclasticus [taxid 75310]: 4 (0.046%)<br>• Rhodanobacter glycinis [taxid 582702]: 3 (0.034%)<br>• Brugia timori [taxid 42155]: 3 (0.034%)<br>• Dyella tabacisoli [taxid 2282381]: 2 (0.023%)<br>• Fulvimonas soli [taxid 155197]: 2 (0.023%)<br>• Massilia eurypsychrophila [taxid 1485217]: 1 (0.011%)<br>• other: 31 (0.359%)

Operational Taxonomic Unit (OTU)	Correct identifications	Wrong or overspecific identifications at species rank
Benchmark OTU ID: `CP000143-_Proteobacteria`<br>OTU taxon: Rhodobacter sphaeroides 2.4.1 [taxid 272943]<br>Expected: Rhodobacter sphaeroides [taxid 1063] (species)<br>Number of reads: 6288<br>Number of identified reads: 6251 (99.411%)	• species: 686 (10.909%)<br>• **genus: 3163 (50.302%)**<br>• family: 1089 (17.318%)<br>• order: 32 (0.508%)<br>• class: 346 (5.502%)<br>• phylum: 169 (2.687%)<br>• superkingdom: 277 (4.405%)<br>• root: 486 (7.729%)	• Rhodobacter ovatus [taxid 439529]: 9 (0.143%)<br>• Rhodobacter megalophilus [taxid 418630]: 7 (0.111%)<br>• Rhodobacter johrii [taxid 445629]: 5 (0.079%)<br>• Rhodobacter blasticus [taxid 1075]: 3 (0.047%)<br>• Roseicitreum antarcticum [taxid 564137]: 2 (0.031%)<br>• Devosia geojensis [taxid 443610]: 1 (0.015%)<br>• Paracoccus laeviglucosivorans [taxid 1197861]: 1 (0.015%)<br>• Aeromonas salmonicida [taxid 645]: 1 (0.015%)<br>• other: 27 (0.429%)
Benchmark OTU ID: `CP000578-_Proteobacteria`<br>OTU taxon: Rhodobacter sphaeroides ATCC 17029 [taxid 349101]<br>Expected: Rhodobacter sphaeroides [taxid 1063] (species)<br>Number of reads: 1859<br>Number of identified reads: 1845 (99.246%)	• species: 310 (16.675%)<br>• **genus: 935 (50.295%)**<br>• family: 129 (6.939%)<br>• order: 2 (0.107%)<br>• class: 118 (6.347%)<br>• phylum: 61 (3.281%)<br>• superkingdom: 109 (5.863%)<br>• root: 175 (9.413%)	• Rhodobacter megalophilus [taxid 418630]: 7 (0.376%)<br>• Rhodobacter johrii [taxid 445629]: 3 (0.161%)<br>• Cordyceps fumosorosea [taxid 114497]: 1 (0.053%)<br>• Lentibacter algarum [taxid 576131]: 1 (0.053%)<br>• Tranquillimonas alkanivorans [taxid 441119]: 1 (0.053%)<br>• Hoyosella subflava [taxid 639313]: 1 (0.053%)<br>• Desulfallas geothermicus [taxid 39060]: 1 (0.053%)<br>• Prasinococcus capsulatus [taxid 156131]: 1 (0.053%)<br>• other: 2 (0.107%)
Benchmark OTU ID: `CP001150-_Proteobacteria`<br>OTU taxon: Rhodobacter sphaeroides KD131 [taxid 557760]<br>Expected: Rhodobacter sphaeroides [taxid 1063] (species)<br>Number of reads: 6210<br>Number of identified reads: 6169 (99.339%)	• species: 674 (10.853%)<br>• **genus: 3037 (48.904%)**<br>• family: 1104 (17.777%)<br>• order: 41 (0.66%)<br>• class: 343 (5.523%)<br>• phylum: 175 (2.818%)<br>• superkingdom: 324 (5.217%)<br>• root: 464 (7.471%)	• Rhodobacter johrii [taxid 445629]: 9 (0.144%)<br>• Rhodobacter ovatus [taxid 439529]: 6 (0.096%)<br>• Rhodobacter megalophilus [taxid 418630]: 4 (0.064%)<br>• Cereibacter changlensis [taxid 402884]: 4 (0.064%)<br>• Celeribacter neptunius [taxid 588602]: 2 (0.032%)<br>• Haematobacter massiliensis [taxid 195105]: 2 (0.032%)<br>• Roseobacter denitrificans [taxid 2434]: 1 (0.016%)<br>• Agrobacterium tumefaciens [taxid 358]: 1 (0.016%)<br>• other: 34 (0.547%)
Benchmark OTU ID: `CP001151-_Proteobacteria`<br>OTU taxon: Rhodobacter sphaeroides KD131 [taxid 557760]<br>Expected: Rhodobacter sphaeroides [taxid 1063] (species)<br>Number of reads: 2036<br>Number of identified reads: 2032 (99.803%)	• species: 456 (22.396%)<br>• **genus: 841 (41.306%)**<br>• family: 194 (9.528%)<br>• order: 3 (0.147%)<br>• class: 114 (5.599%)<br>• phylum: 76 (3.732%)<br>• superkingdom: 109 (5.353%)<br>• root: 235 (11.542%)	• Rhodobacter johrii [taxid 445629]: 5 (0.245%)<br>• Rhodobacter megalophilus [taxid 418630]: 4 (0.196%)<br>• Lupinus albus [taxid 3870]: 2 (0.098%)<br>• Roseovarius tolerans [taxid 74031]: 1 (0.049%)<br>• Variibacter gotjawalensis [taxid 1333996]: 1 (0.049%)<br>• Candidatus Chloroploca asiatica [taxid 1506545]: 1 (0.049%)<br>• Gemmobacter intermedius [taxid 1553448]: 1 (0.049%)<br>• Cereibacter changlensis [taxid 402884]: 1 (0.049%)<br>• other: 8 (0.392%)

Operational Taxonomic Unit (OTU)	Correct identifications	Wrong or overspecific identifications at species rank
Benchmark OTU ID: `AP008957-_Actinobacteria`<br>OTU taxon: Rhodococcus erythropolis PR4 [taxid 234621]<br>Expected: Rhodococcus erythropolis [taxid 1833] (species)<br>Number of reads: 29527<br>Number of identified reads: 29422 (99.644%)	• species: 2425 (8.212%)<br>• **genus: 18278 (61.902%)**<br>• family: 648 (2.194%)<br>• order: 1061 (3.593%)<br>• class: 1596 (5.405%)<br>• phylum: 23 (0.077%)<br>• superkingdom: 1488 (5.039%)<br>• root: 3877 (13.13%)	• Rhodococcus qingshengii [taxid 334542]: 21 (0.071%)<br>• Rhodococcus enclensis [taxid 1049584]: 18 (0.06%)<br>• Rhodococcus triatomae [taxid 300028]: 10 (0.033%)<br>• Rhodococcus tukisamuensis [taxid 168276]: 8 (0.027%)<br>• Ascochyta rabiei [taxid 5454]: 6 (0.02%)<br>• Rhodococcus coprophilus [taxid 38310]: 6 (0.02%)<br>• Rhodococcus rhodnii [taxid 38312]: 5 (0.016%)<br>• Rhodococcus ruber [taxid 1830]: 4 (0.013%)<br>• other: 107 (0.362%)
Benchmark OTU ID: `FN563149-_Actinobacteria`<br>OTU taxon: Rhodococcus hoagii 103S [taxid 685727]<br>Expected: Rhodococcus hoagii [taxid 43767] (species)<br>Number of reads: 22308<br>Number of identified reads: 22241 (99.699%)	• species: 1958 (8.777%)<br>• **genus: 13571 (60.834%)**<br>• family: 489 (2.192%)<br>• order: 1212 (5.433%)<br>• class: 1642 (7.36%)<br>• phylum: 15 (0.067%)<br>• superkingdom: 1301 (5.831%)<br>• root: 2033 (9.113%)	• Rhodococcus agglutinans [taxid 1644129]: 13 (0.058%)<br>• Rhodococcus triatomae [taxid 300028]: 9 (0.04%)<br>• Rhodococcus erythropolis [taxid 1833]: 6 (0.026%)<br>• Rhodococcus gordoniae [taxid 223392]: 4 (0.017%)<br>• Rhodococcus pyridinivorans [taxid 103816]: 4 (0.017%)<br>• Rhodococcus tukisamuensis [taxid 168276]: 3 (0.013%)<br>• Rhodococcus coprophilus [taxid 38310]: 3 (0.013%)<br>• Rhodococcus rhodnii [taxid 38312]: 3 (0.013%)<br>• other: 111 (0.497%)
Benchmark OTU ID: `AP011115-_Actinobacteria`<br>OTU taxon: Rhodococcus opacus B4 [taxid 632772]<br>Expected: Rhodococcus opacus [taxid 37919] (species)<br>Number of reads: 36373<br>Number of identified reads: 36245 (99.648%)	• species: 9729 (26.747%)<br>• **genus: 16408 (45.11%)**<br>• family: 560 (1.539%)<br>• order: 1587 (4.363%)<br>• class: 2384 (6.554%)<br>• phylum: 30 (0.082%)<br>• superkingdom: 2151 (5.913%)<br>• root: 3371 (9.267%)	• Rhodococcus wratislaviensis [taxid 44752]: 78 (0.214%)<br>• Rhodococcus jostii [taxid 132919]: 35 (0.096%)<br>• Rhodococcus triatomae [taxid 300028]: 17 (0.046%)<br>• Rhodococcus koreensis [taxid 99653]: 13 (0.035%)<br>• Rhodococcus imtechensis [taxid 262776]: 7 (0.019%)<br>• Rhodococcus hoagii [taxid 43767]: 4 (0.01%)<br>• Mycobacteroides abscessus [taxid 36809]: 4 (0.01%)<br>• Rhodococcus rhodnii [taxid 38312]: 4 (0.01%)<br>• other: 131 (0.36%)
Benchmark OTU ID: `CP000267-_Proteobacteria`<br>OTU taxon: Rhodoferax ferrireducens T118 [taxid 338969]<br>Expected: Rhodoferax ferrireducens [taxid 192843] (species)<br>Number of reads: 9719<br>Number of identified reads: 9704 (99.845%)	• **species: 4168 (42.885%)**<br>• genus: 1668 (17.162%)<br>• family: 597 (6.142%)<br>• order: 897 (9.229%)<br>• class: 251 (2.582%)<br>• phylum: 697 (7.171%)<br>• superkingdom: 400 (4.115%)<br>• root: 1021 (10.505%)	• Rhodoferax antarcticus [taxid 81479]: 3 (0.03%)<br>• Rhodoferax koreense [taxid 1842727]: 2 (0.02%)<br>• Rubrivivax albus [taxid 2499835]: 1 (0.01%)<br>• Acidovorax temperans [taxid 80878]: 1 (0.01%)<br>• Bacillus albus [taxid 2026189]: 1 (0.01%)<br>• Candidatus Symbiobacter mobilis [taxid 1436290]: 1 (0.01%)<br>• Calditerrivibrio nitroreducens [taxid 477976]: 1 (0.01%)<br>• Candidatus Electrothrix aarhusiensis [taxid 1859131]: 1 (0.01%)<br>• other: 37 (0.38%)

Operational Taxonomic Unit (OTU)	Correct identifications	Wrong or overspecific identifications at species rank
Benchmark OTU ID: `ENA|BX119912|BX119912.1-_Planctomycetes`<br>OTU taxon: Rhodopirellula baltica SH 1 [taxid 243090]<br>Expected: Rhodopirellula baltica [taxid 265606] (species)<br>Number of reads: 269624<br>Number of identified reads: 269247 (99.86%)	• **species: 118747 (44.041%)**<br>• genus: 81688 (30.297%)<br>• family: 2893 (1.072%)<br>• order: 178 (0.066%)<br>• class: 1665 (0.617%)<br>• phylum: 9723 (3.606%)<br>• superkingdom: 15275 (5.665%)<br>• root: 38825 (14.399%)	• Rhodopirellula europaea [taxid 1263866]: 630 (0.233%)<br>• Rhodopirellula islandica [taxid 595434]: 526 (0.195%)<br>• Rhodopirellula bahusiensis [taxid 2014065]: 278 (0.103%)<br>• Rhodopirellula sallentina [taxid 1263869]: 51 (0.018%)<br>• Rhodopirellula solitaria [taxid 2527987]: 48 (0.017%)<br>• Rhodopirellula sp. MGV [taxid 2023130]: 41 (0.015%)<br>• Rubripirellula tenax [taxid 2528015]: 25 (0.009%)<br>• Rhodopirellula maiorica [taxid 1265734]: 23 (0.008%)<br>• other: 494 (0.183%)
Benchmark OTU ID: `CP000283-_Proteobacteria`<br>OTU taxon: Rhodopseudomonas palustris BisB5 [taxid 316057]<br>Expected: Rhodopseudomonas palustris [taxid 1076] (species)<br>Number of reads: 10125<br>Number of identified reads: 10092 (99.674%)	• species: 1251 (12.355%)<br>• **genus: 4927 (48.661%)**<br>• family: 1153 (11.387%)<br>• order: 392 (3.871%)<br>• class: 491 (4.849%)<br>• phylum: 364 (3.595%)<br>• superkingdom: 447 (4.414%)<br>• root: 1053 (10.4%)	• Rhodopseudomonas pseudopalustris [taxid 1513892]: 44 (0.434%)<br>• Rhodopseudomonas pentothenatexigens [taxid 999699]: 4 (0.039%)<br>• Rhodopseudomonas faecalis [taxid 99655]: 4 (0.039%)<br>• Bradyrhizobium erythrophlei [taxid 1437360]: 3 (0.029%)<br>• Parasteatoda tepidariorum [taxid 114398]: 3 (0.029%)<br>• Bradyrhizobium valentinum [taxid 1518501]: 2 (0.019%)<br>• Nitrobacter hamburgensis [taxid 912]: 2 (0.019%)<br>• Bradyrhizobium lablabi [taxid 722472]: 2 (0.019%)<br>• other: 31 (0.306%)
Benchmark OTU ID: `ENA|BX571963|BX571963.1-_Proteobacteria`<br>OTU taxon: Rhodopseudomonas palustris CGA009 [taxid 258594]<br>Expected: Rhodopseudomonas palustris [taxid 1076] (species)<br>Number of reads: 11399<br>Number of identified reads: 11355 (99.614%)	• **species: 5122 (44.933%)**<br>• genus: 1993 (17.483%)<br>• family: 1181 (10.36%)<br>• order: 435 (3.816%)<br>• class: 583 (5.114%)<br>• phylum: 409 (3.588%)<br>• superkingdom: 476 (4.175%)<br>• root: 1150 (10.088%)	• Rhodopseudomonas pentothenatexigens [taxid 999699]: 6 (0.052%)<br>• Rhodopseudomonas faecalis [taxid 99655]: 2 (0.017%)<br>• Azospirillum lipoferum [taxid 193]: 2 (0.017%)<br>• Nitrobacter vulgaris [taxid 29421]: 1 (0.008%)<br>• Rhodoblastus acidophilus [taxid 1074]: 1 (0.008%)<br>• Bradyrhizobium centrolobii [taxid 1505087]: 1 (0.008%)<br>• Conexibacter woesei [taxid 191495]: 1 (0.008%)<br>• Pseudorhodoplanes sinuspersici [taxid 1235591]: 1 (0.008%)<br>• other: 38 (0.333%)
Benchmark OTU ID: `CP002418-_Proteobacteria`<br>OTU taxon: Rhodopseudomonas palustris DX-1 [taxid 652103]<br>Expected: Rhodopseudomonas palustris [taxid 1076] (species)<br>Number of reads: 11275<br>Number of identified reads: 11236 (99.654%)	• **species: 4673 (41.445%)**<br>• genus: 2272 (20.15%)<br>• family: 1074 (9.525%)<br>• order: 491 (4.354%)<br>• class: 577 (5.117%)<br>• phylum: 444 (3.937%)<br>• superkingdom: 545 (4.833%)<br>• root: 1141 (10.119%)	• Rhodopseudomonas pentothenatexigens [taxid 999699]: 12 (0.106%)<br>• Rhodopseudomonas faecalis [taxid 99655]: 7 (0.062%)<br>• Bradyrhizobium neotropicale [taxid 1497615]: 2 (0.017%)<br>• Bradyrhizobium icense [taxid 1274631]: 2 (0.017%)<br>• Tanacetum cinerariifolium [taxid 118510]: 1 (0.008%)<br>• Salipiger profundus [taxid 1229727]: 1 (0.008%)<br>• Rhodoplanes elegans [taxid 29408]: 1 (0.008%)<br>• Pusillimonas noertemannii [taxid 305977]: 1 (0.008%)<br>• other: 41 (0.363%)

Operational Taxonomic Unit (OTU)	Correct identifications	Wrong or overspecific identifications at species rank
Benchmark OTU ID: `CP000250-_Proteobacteria`<br>OTU taxon: Rhodopseudomonas palustris HaA2 [taxid 316058]<br>Expected: Rhodopseudomonas palustris [taxid 1076] (species)<br>Number of reads: 11112<br>Number of identified reads: 11069 (99.613%)	• **species: 5581 (50.224%)**<br>• genus: 1261 (11.348%)<br>• family: 1213 (10.916%)<br>• order: 439 (3.95%)<br>• class: 514 (4.625%)<br>• phylum: 370 (3.329%)<br>• superkingdom: 566 (5.093%)<br>• root: 1120 (10.079%)	• Bradyrhizobium nanningense [taxid 1325118]: 2 (0.017%)<br>• Lupinus albus [taxid 3870]: 2 (0.017%)<br>• Rhodopseudomonas pentothenatexigens [taxid 999699]: 2 (0.017%)<br>• Rhodopseudomonas faecalis [taxid 99655]: 2 (0.017%)<br>• Variibacter gotjawalensis [taxid 1333996]: 1 (0.008%)<br>• Limosilactobacillus reuteri [taxid 1598]: 1 (0.008%)<br>• Bradyrhizobium japonicum [taxid 375]: 1 (0.008%)<br>• Camelimonas lactis [taxid 659006]: 1 (0.008%)<br>• other: 33 (0.296%)
Benchmark OTU ID: `CP001096-_Proteobacteria`<br>OTU taxon: Rhodopseudomonas palustris TIE-1 [taxid 395960]<br>Expected: Rhodopseudomonas palustris [taxid 1076] (species)<br>Number of reads: 12040<br>Number of identified reads: 11984 (99.534%)	• **species: 5475 (45.473%)**<br>• genus: 2104 (17.475%)<br>• family: 1192 (9.9%)<br>• order: 465 (3.862%)<br>• class: 490 (4.069%)<br>• phylum: 447 (3.712%)<br>• superkingdom: 507 (4.21%)<br>• root: 1291 (10.722%)	• Rhodopseudomonas faecalis [taxid 99655]: 5 (0.041%)<br>• Rhodopseudomonas pentothenatexigens [taxid 999699]: 5 (0.041%)<br>• Bradyrhizobium icense [taxid 1274631]: 3 (0.024%)<br>• Bradyrhizobium zhanjiangense [taxid 1325107]: 2 (0.016%)<br>• Bradyrhizobium daqingense [taxid 993502]: 2 (0.016%)<br>• Bradyrhizobium shewense [taxid 1761772]: 2 (0.016%)<br>• Rhizobium leguminosarum [taxid 384]: 2 (0.016%)<br>• Phreatobacter stygius [taxid 1940610]: 1 (0.008%)<br>• other: 36 (0.299%)
Benchmark OTU ID: `CP000613-_Proteobacteria`<br>OTU taxon: Rhodospirillum centenum SW [taxid 414684]<br>Expected: Rhodospirillum centenum [taxid 34018] (species)<br>Number of reads: 8916<br>Number of identified reads: 8872 (99.506%)	• **species: 6011 (67.418%)**<br>• genus: 1 (0.011%)<br>• family: 238 (2.669%)<br>• order: 29 (0.325%)<br>• class: 616 (6.908%)<br>• phylum: 480 (5.383%)<br>• superkingdom: 625 (7.009%)<br>• root: 865 (9.701%)	• Magnetospirillum marisnigri [taxid 1285242]: 2 (0.022%)<br>• Aurantimonas aggregata [taxid 2047720]: 2 (0.022%)<br>• Nitrospirillum amazonense [taxid 28077]: 2 (0.022%)<br>• Candidatus Kentron sp. H [taxid 2126337]: 1 (0.011%)<br>• Chamaesiphon polymorphus [taxid 2107691]: 1 (0.011%)<br>• Niveispirillum lacus [taxid 1981099]: 1 (0.011%)<br>• Rhodocytophaga rosea [taxid 2704465]: 1 (0.011%)<br>• Chanos chanos [taxid 29144]: 1 (0.011%)<br>• other: 22 (0.246%)
Benchmark OTU ID: `CP003046-_Proteobacteria`<br>OTU taxon: Rhodospirillum rubrum F11 [taxid 1036743]<br>Expected: Rhodospirillum rubrum [taxid 1085] (species)<br>Number of reads: 8906<br>Number of identified reads: 8862 (99.505%)	• **species: 6196 (69.571%)**<br>• genus: 4 (0.044%)<br>• family: 178 (1.998%)<br>• order: 32 (0.359%)<br>• class: 517 (5.805%)<br>• phylum: 457 (5.131%)<br>• superkingdom: 522 (5.861%)<br>• root: 948 (10.644%)	• Pararhodospirillum photometricum [taxid 1084]: 6 (0.067%)<br>• Rhizobium tubonense [taxid 484088]: 2 (0.022%)<br>• cyanobacterium endosymbiont of Rhopalodia gibberula [taxid 1763363]: 1 (0.011%)<br>• Aestuariivirga litoralis [taxid 2650924]: 1 (0.011%)<br>• Isobaculum melis [taxid 142588]: 1 (0.011%)<br>• Candidimonas bauzanensis [taxid 658167]: 1 (0.011%)<br>• Neokomagataea tanensis [taxid 661191]: 1 (0.011%)<br>• Hibiscus syriacus [taxid 106335]: 1 (0.011%)<br>• other: 19 (0.213%)

Operational Taxonomic Unit (OTU)	Correct identifications	Wrong or overspecific identifications at species rank
Benchmark OTU ID: `CP001807-_Bacteroidetes`<br>OTU taxon: Rhodothermus marinus DSM 4252 [taxid 518766]<br>Expected: Rhodothermus marinus [taxid 29549] (species)<br>Number of reads: 20977<br>Number of identified reads: 20937 (99.809%)	• **species: 13525 (64.475%)**<br>• genus: 1772 (8.447%)<br>• family: 35 (0.166%)<br>• order: 0 (0.0%)<br>• phylum: 457 (2.178%)<br>• superkingdom: 3561 (16.975%)<br>• root: 1571 (7.489%)	• Rhodothermus profundi [taxid 633813]: 20 (0.095%)<br>• bacterium [taxid 1869227]: 2 (0.009%)<br>• Halocynthiibacter arcticus [taxid 1579316]: 1 (0.004%)<br>• Streptomyces himastatinicus [taxid 998084]: 1 (0.004%)<br>• Bradyrhizobium japonicum [taxid 375]: 1 (0.004%)<br>• Paraburkholderia bryophila [taxid 420952]: 1 (0.004%)<br>• Gemmata obscuriglobus [taxid 114]: 1 (0.004%)<br>• other: 56 (0.266%)
Benchmark OTU ID: `CP001612-_Pathogens`<br>OTU taxon: Rickettsia africae ESF-5 [taxid 347255]<br>Expected: Rickettsia africae [taxid 35788] (species)<br>Number of reads: 1981<br>Number of identified reads: 1950 (98.435%)	• species: 165 (8.329%)<br>• **genus: 1450 (73.195%)**<br>• family: 3 (0.151%)<br>• order: 8 (0.403%)<br>• class: 20 (1.009%)<br>• phylum: 27 (1.362%)<br>• superkingdom: 60 (3.028%)<br>• root: 217 (10.954%)	• Rickettsia rhipicephali [taxid 33992]: 16 (0.807%)<br>• Rickettsia amblyommatis [taxid 33989]: 14 (0.706%)<br>• Rickettsia parkeri [taxid 35792]: 14 (0.706%)<br>• Rickettsia felis [taxid 42862]: 11 (0.555%)<br>• Rickettsia conorii [taxid 781]: 10 (0.504%)<br>• Rickettsia rickettsii [taxid 783]: 9 (0.454%)<br>• Rickettsia montanensis [taxid 33991]: 5 (0.252%)<br>• Rickettsia canadensis [taxid 788]: 5 (0.252%)<br>• other: 45 (2.271%)
Benchmark OTU ID: `CP003338-_Pathogens`<br>OTU taxon: Rickettsia australis str. Cutlack [taxid 1105110]<br>Expected: Rickettsia australis [taxid 787] (species)<br>Number of reads: 2028<br>Number of identified reads: 1976 (97.435%)	• species: 589 (29.043%)<br>• **genus: 973 (47.978%)**<br>• family: 5 (0.246%)<br>• order: 3 (0.147%)<br>• class: 26 (1.282%)<br>• phylum: 23 (1.134%)<br>• superkingdom: 63 (3.106%)<br>• root: 293 (14.447%)	• Rickettsia felis [taxid 42862]: 37 (1.824%)<br>• Rickettsia akari [taxid 786]: 18 (0.887%)<br>• Rickettsia asiatica [taxid 238800]: 8 (0.394%)<br>• Rickettsia massiliae [taxid 35791]: 4 (0.197%)<br>• Rickettsia amblyommatis [taxid 33989]: 3 (0.147%)<br>• Rickettsia endosymbiont of Ixodes pacificus [taxid 1133329]: 3 (0.147%)<br>• Rickettsia asembonensis [taxid 1068590]: 3 (0.147%)<br>• Rickettsia bellii [taxid 33990]: 3 (0.147%)<br>• other: 17 (0.838%)
Benchmark OTU ID: `CP003304-_Pathogens`<br>OTU taxon: Rickettsia canadensis str. CA410 [taxid 1105107]<br>Expected: Rickettsia canadensis [taxid 788] (species)<br>Number of reads: 1662<br>Number of identified reads: 1613 (97.051%)	• **species: 716 (43.08%)**<br>• genus: 460 (27.677%)<br>• family: 0 (0.0%)<br>• order: 5 (0.3%)<br>• class: 28 (1.684%)<br>• phylum: 23 (1.383%)<br>• superkingdom: 48 (2.888%)<br>• root: 331 (19.915%)	• Rickettsia asiatica [taxid 238800]: 6 (0.361%)<br>• Rickettsia akari [taxid 786]: 4 (0.24%)<br>• Rickettsia felis [taxid 42862]: 3 (0.18%)<br>• Rickettsia typhi [taxid 785]: 2 (0.12%)<br>• Rickettsia amblyommatis [taxid 33989]: 2 (0.12%)<br>• Rickettsia bellii [taxid 33990]: 2 (0.12%)<br>• Rickettsia endosymbiont of Culicoides newsteadi [taxid 1961830]: 1 (0.06%)<br>• Rickettsia montanensis [taxid 33991]: 1 (0.06%)<br>• other: 7 (0.421%)

Operational Taxonomic Unit (OTU)	Correct identifications	Wrong or overspecific identifications at species rank
Benchmark OTU ID: `CP000409-_Pathogens`<br>OTU taxon: Rickettsia canadensis str. McKiel [taxid 293613]<br>Expected: Rickettsia canadensis [taxid 788] (species)<br>Number of reads: 1685<br>Number of identified reads: 1629 (96.676%)	• **species: 831 (49.317%)**<br>• genus: 412 (24.451%)<br>• family: 3 (0.178%)<br>• order: 3 (0.178%)<br>• class: 24 (1.424%)<br>• phylum: 21 (1.246%)<br>• superkingdom: 59 (3.501%)<br>• root: 274 (16.261%)	• Rickettsia asiatica [taxid 238800]: 3 (0.178%)<br>• Rickettsia massiliae [taxid 35791]: 3 (0.178%)<br>• Rickettsia felis [taxid 42862]: 3 (0.178%)<br>• Rickettsia amblyommatis [taxid 33989]: 3 (0.178%)<br>• Mucuna pruriens [taxid 157652]: 1 (0.059%)<br>• Rickettsia rhipicephali [taxid 33992]: 1 (0.059%)<br>• Rickettsia argasii [taxid 1441385]: 1 (0.059%)<br>• Magnetovibrio blakemorei [taxid 28181]: 1 (0.059%)<br>• other: 9 (0.534%)
Benchmark OTU ID: `CP002912-_Pathogens`<br>OTU taxon: Rickettsia heilongjiangensis 054 [taxid 1032845]<br>Expected: Rickettsia heilongjiangensis [taxid 226665] (species)<br>Number of reads: 1982<br>Number of identified reads: 1933 (97.527%)	• species: 103 (5.196%)<br>• **genus: 1529 (77.144%)**<br>• family: 2 (0.1%)<br>• order: 2 (0.1%)<br>• class: 22 (1.109%)<br>• phylum: 28 (1.412%)<br>• superkingdom: 57 (2.875%)<br>• root: 187 (9.434%)	• **Rickettsia argasii [taxid 1441385]: 46 (2.32%)**<br>• Rickettsia felis [taxid 42862]: 14 (0.706%)<br>• Rickettsia amblyommatis [taxid 33989]: 11 (0.554%)<br>• Rickettsia massiliae [taxid 35791]: 9 (0.454%)<br>• Rickettsia japonica [taxid 35790]: 9 (0.454%)<br>• Rickettsia montanensis [taxid 33991]: 7 (0.353%)<br>• Rickettsia rhipicephali [taxid 33992]: 5 (0.252%)<br>• Rickettsia canadensis [taxid 788]: 5 (0.252%)<br>• other: 25 (1.261%)
Benchmark OTU ID: `AP011533-_Pathogens`<br>OTU taxon: Rickettsia japonica YH [taxid 652620]<br>Expected: Rickettsia japonica [taxid 35790] (species)<br>Number of reads: 1994<br>Number of identified reads: 1952 (97.893%)	• species: 129 (6.469%)<br>• **genus: 1508 (75.626%)**<br>• family: 3 (0.15%)<br>• order: 4 (0.2%)<br>• class: 25 (1.253%)<br>• phylum: 22 (1.103%)<br>• superkingdom: 51 (2.557%)<br>• root: 210 (10.531%)	• Rickettsia argasii [taxid 1441385]: 35 (1.755%)<br>• Rickettsia rhipicephali [taxid 33992]: 12 (0.601%)<br>• Rickettsia heilongjiangensis [taxid 226665]: 11 (0.551%)<br>• Rickettsia amblyommatis [taxid 33989]: 10 (0.501%)<br>• Rickettsia montanensis [taxid 33991]: 6 (0.3%)<br>• Rickettsia rickettsii [taxid 783]: 6 (0.3%)<br>• Rickettsia conorii [taxid 781]: 6 (0.3%)<br>• Rickettsia canadensis [taxid 788]: 6 (0.3%)<br>• other: 37 (1.855%)
Benchmark OTU ID: `CP003308-_Pathogens`<br>OTU taxon: Rickettsia philipii str. 364D [taxid 481009]<br>Expected: Rickettsia philipii [taxid 1105106] (species)<br>Number of reads: 2005<br>Number of identified reads: 1962 (97.855%)	• species: 106 (5.286%)<br>• **genus: 1536 (76.608%)**<br>• family: 3 (0.149%)<br>• order: 5 (0.249%)<br>• class: 21 (1.047%)<br>• phylum: 26 (1.296%)<br>• superkingdom: 60 (2.992%)<br>• root: 203 (10.124%)	• Rickettsia rickettsii [taxid 783]: 22 (1.097%)<br>• Rickettsia amblyommatis [taxid 33989]: 12 (0.598%)<br>• Rickettsia massiliae [taxid 35791]: 9 (0.448%)<br>• Rickettsia parkeri [taxid 35792]: 8 (0.399%)<br>• Rickettsia endosymbiont of Proechinophthirus fluctus [taxid 1462733]: 6 (0.299%)<br>• Rickettsia rhipicephali [taxid 33992]: 6 (0.299%)<br>• Rickettsia africae [taxid 35788]: 6 (0.299%)<br>• Rickettsia slovaca [taxid 35794]: 5 (0.249%)<br>• other: 57 (2.842%)

Operational Taxonomic Unit (OTU)	Correct identifications	Wrong or overspecific identifications at species rank
Benchmark OTU ID: `CP004889-_Pathogens`<br>OTU taxon: Rickettsia prowazekii str. Breinl [taxid 1290428]<br>Expected: Rickettsia prowazekii [taxid 782] (species)<br>Number of reads: 1559<br>Number of identified reads: 1495 (95.894%)	• **species: 677 (43.425%)**<br>• genus: 466 (29.89%)<br>• family: 1 (0.064%)<br>• order: 5 (0.32%)<br>• class: 24 (1.539%)<br>• phylum: 18 (1.154%)<br>• superkingdom: 44 (2.822%)<br>• root: 260 (16.677%)	• Rickettsia typhi [taxid 785]: 2 (0.128%)<br>• Rickettsia asiatica [taxid 238800]: 1 (0.064%)<br>• Rickettsia peacockii [taxid 47589]: 1 (0.064%)<br>• Rickettsia rhipicephali [taxid 33992]: 1 (0.064%)<br>• Rickettsia akari [taxid 786]: 1 (0.064%)<br>• Rickettsia amblyommatis [taxid 33989]: 1 (0.064%)<br>• Rickettsia felis [taxid 42862]: 1 (0.064%)
Benchmark OTU ID: `CP003393-_Pathogens`<br>OTU taxon: Rickettsia prowazekii str. BuV67-CWPP [taxid 1105096]<br>Expected: Rickettsia prowazekii [taxid 782] (species)<br>Number of reads: 1565<br>Number of identified reads: 1469 (93.865%)	• **species: 702 (44.856%)**<br>• genus: 459 (29.329%)<br>• family: 6 (0.383%)<br>• order: 4 (0.255%)<br>• class: 19 (1.214%)<br>• phylum: 10 (0.638%)<br>• superkingdom: 47 (3.003%)<br>• root: 216 (13.801%)	• Rickettsia typhi [taxid 785]: 3 (0.191%)<br>• Rickettsia endosymbiont of Culicoides newsteadi [taxid 1961830]: 2 (0.127%)<br>• Rickettsia amblyommatis [taxid 33989]: 2 (0.127%)<br>• Aspergillus ellipticus [taxid 41066]: 1 (0.063%)<br>• Buchnera aphidicola [taxid 9]: 1 (0.063%)<br>• Protopolystoma xenopodis [taxid 117903]: 1 (0.063%)<br>• Rickettsia akari [taxid 786]: 1 (0.063%)
Benchmark OTU ID: `CP003392-_Pathogens`<br>OTU taxon: Rickettsia prowazekii str. Katsinyian [taxid 1105095]<br>Expected: Rickettsia prowazekii [taxid 782] (species)<br>Number of reads: 1565<br>Number of identified reads: 1475 (94.249%)	• **species: 647 (41.341%)**<br>• genus: 463 (29.584%)<br>• family: 0 (0.0%)<br>• order: 7 (0.447%)<br>• class: 16 (1.022%)<br>• phylum: 22 (1.405%)<br>• superkingdom: 58 (3.706%)<br>• root: 259 (16.549%)	• Rickettsia typhi [taxid 785]: 3 (0.191%)<br>• Rickettsia raoultii [taxid 369822]: 2 (0.127%)<br>• Chimaeribacter californicus [taxid 2060067]: 1 (0.063%)<br>• Rickettsia felis [taxid 42862]: 1 (0.063%)<br>• Rickettsia amblyommatis [taxid 33989]: 1 (0.063%)<br>• Pararhodospirillum photometricum [taxid 1084]: 1 (0.063%)<br>• Paraburkholderia hospita [taxid 169430]: 1 (0.063%)<br>• Trichechus manatus [taxid 9778]: 1 (0.063%)<br>• other: 4 (0.255%)
Benchmark OTU ID: `CP003305-_Pathogens`<br>OTU taxon: Rickettsia rickettsii str. Brazil [taxid 1105104]<br>Expected: Rickettsia rickettsii [taxid 783] (species)<br>Number of reads: 1925<br>Number of identified reads: 1882 (97.766%)	• species: 131 (6.805%)<br>• **genus: 1418 (73.662%)**<br>• family: 3 (0.155%)<br>• order: 7 (0.363%)<br>• class: 20 (1.038%)<br>• phylum: 28 (1.454%)<br>• superkingdom: 59 (3.064%)<br>• root: 215 (11.168%)	• Rickettsia felis [taxid 42862]: 14 (0.727%)<br>• Rickettsia amblyommatis [taxid 33989]: 8 (0.415%)<br>• Rickettsia asiatica [taxid 238800]: 7 (0.363%)<br>• Rickettsia philipii [taxid 1105106]: 7 (0.363%)<br>• Rickettsia massiliae [taxid 35791]: 6 (0.311%)<br>• Rickettsia rhipicephali [taxid 33992]: 6 (0.311%)<br>• Rickettsia akari [taxid 786]: 5 (0.259%)<br>• Rickettsia australis [taxid 787]: 4 (0.207%)<br>• other: 37 (1.922%)

Operational Taxonomic Unit (OTU)	Correct identifications	Wrong or overspecific identifications at species rank
Benchmark OTU ID: `CP003306-_Pathogens`<br>OTU taxon: Rickettsia rickettsii str. Colombia [taxid 1105102]<br>Expected: Rickettsia rickettsii [taxid 783] (species)<br>Number of reads: 1961<br>Number of identified reads: 1924 (98.113%)	• species: 132 (6.731%)<br>• **genus: 1469 (74.91%)**<br>• family: 2 (0.101%)<br>• order: 1 (0.05%)<br>• class: 15 (0.764%)<br>• phylum: 25 (1.274%)<br>• superkingdom: 56 (2.855%)<br>• root: 221 (11.269%)	• Rickettsia amblyommatis [taxid 33989]: 10 (0.509%)<br>• Rickettsia rhipicephali [taxid 33992]: 9 (0.458%)<br>• Rickettsia massiliae [taxid 35791]: 9 (0.458%)<br>• Rickettsia philipii [taxid 1105106]: 7 (0.356%)<br>• Rickettsia felis [taxid 42862]: 7 (0.356%)<br>• Rickettsia sibirica [taxid 35793]: 6 (0.305%)<br>• Rickettsia parkeri [taxid 35792]: 6 (0.305%)<br>• Rickettsia argasii [taxid 1441385]: 5 (0.254%)<br>• other: 49 (2.498%)
Benchmark OTU ID: `AE017197-_Pathogens`<br>OTU taxon: Rickettsia typhi str. Wilmington [taxid 257363]<br>Expected: Rickettsia typhi [taxid 785] (species)<br>Number of reads: 1565<br>Number of identified reads: 1483 (94.76%)	• **species: 690 (44.089%)**<br>• genus: 450 (28.753%)<br>• family: 1 (0.063%)<br>• order: 3 (0.191%)<br>• class: 19 (1.214%)<br>• phylum: 16 (1.022%)<br>• superkingdom: 53 (3.386%)<br>• root: 250 (15.974%)	• Rickettsia prowazekii [taxid 782]: 6 (0.383%)<br>• Clostridium homopropionicum [taxid 36844]: 1 (0.063%)<br>• Rickettsia massiliae [taxid 35791]: 1 (0.063%)<br>• Sulfurihydrogenibium azorense [taxid 309806]: 1 (0.063%)<br>• Rickettsia canadensis [taxid 788]: 1 (0.063%)<br>• Rickettsia bellii [taxid 33990]: 1 (0.063%)<br>• Bibersteinia trehalosi [taxid 47735]: 1 (0.063%)<br>• Rickettsia montanensis [taxid 33991]: 1 (0.063%)<br>• other: 2 (0.127%)
Benchmark OTU ID: `CP003388-_Bacteroidetes`<br>OTU taxon: Riemerella anatipestifer ATCC 11845 = DSM 15868 [taxid 693978]<br>Expected: Riemerella anatipestifer [taxid 34085] (species)<br>Number of reads: 12635<br>Number of identified reads: 12479 (98.765%)	• **species: 8538 (67.574%)**<br>• genus: 94 (0.743%)<br>• family: 1239 (9.806%)<br>• order: 408 (3.229%)<br>• class: 0 (0.0%)<br>• phylum: 387 (3.062%)<br>• superkingdom: 531 (4.202%)<br>• root: 1249 (9.885%)	• Lupinus albus [taxid 3870]: 11 (0.087%)<br>• Riemerella columbipharyngis [taxid 1071918]: 5 (0.039%)<br>• Chitinophaga eiseniae [taxid 634771]: 3 (0.023%)<br>• Chryseobacterium taklimakanense [taxid 536441]: 2 (0.015%)<br>• Chryseobacterium taihuense [taxid 1141221]: 1 (0.007%)<br>• Persephonella marina [taxid 309805]: 1 (0.007%)<br>• Crustomastix stigmatica [taxid 195967]: 1 (0.007%)<br>• Elizabethkingia anophelis [taxid 1117645]: 1 (0.007%)<br>• other: 34 (0.269%)
Benchmark OTU ID: `CP003787-_Bacteroidetes`<br>OTU taxon: Riemerella anatipestifer RA-CH-1 [taxid 1228997]<br>Expected: Riemerella anatipestifer [taxid 34085] (species)<br>Number of reads: 13741<br>Number of identified reads: 13563 (98.704%)	• **species: 9398 (68.393%)**<br>• genus: 84 (0.611%)<br>• family: 1345 (9.788%)<br>• order: 449 (3.267%)<br>• class: 3 (0.021%)<br>• phylum: 376 (2.736%)<br>• superkingdom: 587 (4.271%)<br>• root: 1291 (9.395%)	• Lupinus albus [taxid 3870]: 19 (0.138%)<br>• Riemerella columbipharyngis [taxid 1071918]: 3 (0.021%)<br>• Ornithobacterium rhinotracheale [taxid 28251]: 2 (0.014%)<br>• Chryseobacterium defluvii [taxid 160396]: 1 (0.007%)<br>• Salegentibacter agarivorans [taxid 345907]: 1 (0.007%)<br>• Chryseobacterium taihuense [taxid 1141221]: 1 (0.007%)<br>• Cyclobacterium qasimii [taxid 1350429]: 1 (0.007%)<br>• Haloechinothrix alba [taxid 664784]: 1 (0.007%)<br>• other: 37 (0.269%)

Operational Taxonomic Unit (OTU)	Correct identifications	Wrong or overspecific identifications at species rank
Benchmark OTU ID: `CP001287-_Cyanobacteria`<br>OTU taxon: Rippkaea orientalis PCC 8801 [taxid 41431]<br>Expected: Rippkaea orientalis [taxid 2546366] (species)<br>Number of reads: 27024<br>Number of identified reads: 26641 (98.582%)	• **species: 17534 (64.883%)**<br>• genus: 0 (0.0%)<br>• family: 505 (1.868%)<br>• order: 644 (2.383%)<br>• phylum: 3227 (11.941%)<br>• superkingdom: 1579 (5.842%)<br>• root: 3112 (11.515%)	• Aphanothece sacrum [taxid 1122]: 14 (0.051%)<br>• Gloeothece citriformis [taxid 2546356]: 10 (0.037%)<br>• Microcystis aeruginosa [taxid 1126]: 9 (0.033%)<br>• Crocosphaera watsonii [taxid 263511]: 9 (0.033%)<br>• Crocosphaera chwakensis [taxid 2546361]: 5 (0.018%)<br>• Crocosphaera subtropica [taxid 2546360]: 4 (0.014%)<br>• Merismopedia glauca [taxid 292586]: 4 (0.014%)<br>• other: 67 (0.247%)
Benchmark OTU ID: `CP001701-_Cyanobacteria`<br>OTU taxon: Rippkaea orientalis PCC 8802 [taxid 395962]<br>Expected: Rippkaea orientalis [taxid 2546366] (species)<br>Number of reads: 26961<br>Number of identified reads: 26638 (98.801%)	• **species: 15861 (58.829%)**<br>• genus: 0 (0.0%)<br>• family: 593 (2.199%)<br>• order: 699 (2.592%)<br>• phylum: 3683 (13.66%)<br>• superkingdom: 1688 (6.26%)<br>• root: 4059 (15.055%)	• Crocosphaera watsonii [taxid 263511]: 36 (0.133%)<br>• Aphanothece sacrum [taxid 1122]: 22 (0.081%)<br>• Microcystis aeruginosa [taxid 1126]: 16 (0.059%)<br>• Aphanothece hegewaldii [taxid 1521625]: 12 (0.044%)<br>• Crocosphaera subtropica [taxid 2546360]: 11 (0.04%)<br>• Crocosphaera chwakensis [taxid 2546361]: 10 (0.037%)<br>• Gloeothece verrucosa [taxid 2546359]: 9 (0.033%)<br>• other: 108 (0.4%)
Benchmark OTU ID: `CP003549-_Cyanobacteria`<br>OTU taxon: Rivularia sp. PCC 7116 [taxid 373994]<br>Expected: Rivularia [taxid 373984] (genus)<br>Number of reads: 53549<br>Number of identified reads: 52348 (97.757%)	• **genus: 32857 (61.358%)**<br>• family: 0 (0.0%)<br>• order: 5513 (10.295%)<br>• phylum: 2912 (5.438%)<br>• superkingdom: 2597 (4.849%)<br>• root: 8368 (15.626%)	• Calothrix parasitica [taxid 1973486]: 126 (0.235%)<br>• Mastigocoleus testarum [taxid 996925]: 16 (0.029%)<br>• Calothrix parietina [taxid 32054]: 11 (0.02%)<br>• Nostoc linckia [taxid 92942]: 6 (0.011%)<br>• Tolypothrix bouteillei [taxid 1246981]: 6 (0.011%)<br>• Nostoc minutum [taxid 1841509]: 5 (0.009%)<br>• other: 135 (0.252%)
Benchmark OTU ID: `CP003040-_Firmicutes`<br>OTU taxon: Roseburia hominis A2-183 [taxid 585394]<br>Expected: Roseburia hominis [taxid 301301] (species)<br>Number of reads: 4978<br>Number of identified reads: 4965 (99.738%)	• species: 888 (17.838%)<br>• **genus: 2046 (41.1%)**<br>• family: 239 (4.801%)<br>• order: 842 (16.914%)<br>• class: 1 (0.02%)<br>• phylum: 197 (3.957%)<br>• superkingdom: 236 (4.74%)<br>• root: 513 (10.305%)	• Roseburia inulinivorans [taxid 360807]: 4 (0.08%)<br>• [Bacteroides] pectinophilus [taxid 384638]: 3 (0.06%)<br>• Roseburia intestinalis [taxid 166486]: 3 (0.06%)<br>• Blautia obeum [taxid 40520]: 2 (0.04%)<br>• Clostridium botulinum [taxid 1491]: 2 (0.04%)<br>• Cuneatibacter caecimuris [taxid 1796618]: 2 (0.04%)<br>• Blautia wexlerae [taxid 418240]: 2 (0.04%)<br>• Blautia faecicola [taxid 2509240]: 1 (0.02%)<br>• other: 24 (0.482%)
Benchmark OTU ID: `FP929050-_Firmicutes`<br>OTU taxon: Roseburia intestinalis XB6B4 [taxid 718255]<br>Expected: Roseburia intestinalis [taxid 166486] (species)<br>Number of reads: 5818<br>Number of identified reads: 5803 (99.742%)	• species: 1814 (31.179%)<br>• **genus: 1893 (32.536%)**<br>• family: 285 (4.898%)<br>• order: 786 (13.509%)<br>• class: 7 (0.12%)<br>• phylum: 224 (3.85%)<br>• superkingdom: 239 (4.107%)<br>• root: 552 (9.487%)	• [Eubacterium] rectale [taxid 39491]: 3 (0.051%)<br>• Roseburia inulinivorans [taxid 360807]: 3 (0.051%)<br>• [Bacteroides] pectinophilus [taxid 384638]: 2 (0.034%)<br>• Coprococcus comes [taxid 410072]: 2 (0.034%)<br>• Phormidium ambiguum [taxid 71191]: 1 (0.017%)<br>• Dorea formicigenerans [taxid 39486]: 1 (0.017%)<br>• Lacrimispora algidixylanolytica [taxid 94868]: 1 (0.017%)<br>• Lederbergia galactosidilyticus [taxid 217031]: 1 (0.017%)<br>• other: 19 (0.326%)

Operational Taxonomic Unit (OTU)	Correct identifications	Wrong or overspecific identifications at species rank
Benchmark OTU ID: `CP000804-_Chloroflexi`<br>OTU taxon: Roseiflexus castenholzii DSM 13941 [taxid 383372]<br>Expected: Roseiflexus castenholzii [taxid 120962] (species)<br>Number of reads: 146632<br>Number of identified reads: 144419 (98.49%)	• **species: 85416 (58.251%)**<br>• genus: 10321 (7.038%)<br>• family: 255 (0.173%)<br>• order: 1170 (0.797%)<br>• class: 36 (0.024%)<br>• phylum: 5188 (3.538%)<br>• superkingdom: 15051 (10.264%)<br>• root: 26809 (18.283%)	• Chloroflexus aggregans [taxid 152260]: 41 (0.027%)<br>• Kouleothrix aurantiaca [taxid 186479]: 10 (0.006%)<br>• Chloroflexus islandicus [taxid 1707952]: 8 (0.005%)<br>• Candidatus Viridilinea halotolerans [taxid 2491704]: 8 (0.005%)<br>• Caldilinea aerophila [taxid 133453]: 5 (0.003%)<br>• Oscillochloris trichoides [taxid 104176]: 4 (0.002%)<br>• Candidatus Viridilinea mediisalina [taxid 2024553]: 4 (0.002%)<br>• Ophiocordyceps camponoti-rufipedis [taxid 2004952]: 3 (0.002%)<br>• other: 288 (0.196%)
Benchmark OTU ID: `CP000686-_Chloroflexi`<br>OTU taxon: Roseiflexus sp. RS-1 [taxid 357808]<br>Expected: Roseiflexus [taxid 120961] (genus)<br>Number of reads: 148801<br>Number of identified reads: 147955 (99.431%)	• **genus: 103070 (69.267%)**<br>• family: 247 (0.165%)<br>• order: 559 (0.375%)<br>• class: 78 (0.052%)<br>• phylum: 3454 (2.321%)<br>• superkingdom: 15315 (10.292%)<br>• root: 25040 (16.827%)	• Roseiflexus castenholzii [taxid 120962]: 630 (0.423%)<br>• Caldilinea aerophila [taxid 133453]: 16 (0.01%)<br>• Chloroflexus islandicus [taxid 1707952]: 14 (0.009%)<br>• Candidatus Viridilinea halotolerans [taxid 2491704]: 9 (0.006%)<br>• Kouleothrix aurantiaca [taxid 186479]: 7 (0.004%)<br>• bacterium [taxid 1869227]: 5 (0.003%)<br>• Candidatus Viridilinea mediisalina [taxid 2024553]: 5 (0.003%)<br>• other: 373 (0.25%)
Benchmark OTU ID: `CP000362-_Proteobacteria`<br>OTU taxon: Roseobacter denitrificans OCh 114 [taxid 375451]<br>Expected: Roseobacter denitrificans [taxid 2434] (species)<br>Number of reads: 8415<br>Number of identified reads: 8407 (99.904%)	• **species: 3449 (40.986%)**<br>• genus: 1370 (16.28%)<br>• family: 1696 (20.154%)<br>• order: 23 (0.273%)<br>• class: 392 (4.658%)<br>• phylum: 241 (2.863%)<br>• superkingdom: 286 (3.398%)<br>• root: 947 (11.253%)	• Roseobacter litoralis [taxid 42443]: 42 (0.499%)<br>• Sulfitobacter sabulilitoris [taxid 2562655]: 2 (0.023%)<br>• Nereida ignava [taxid 282199]: 2 (0.023%)<br>• Eilatimonas milleporae [taxid 911205]: 2 (0.023%)<br>• Lutimaribacter pacificus [taxid 391948]: 2 (0.023%)<br>• Sulfitobacter delicatus [taxid 218672]: 2 (0.023%)<br>• Roseovarius aestuarii [taxid 475083]: 2 (0.023%)<br>• Crocosphaera watsonii [taxid 263511]: 1 (0.011%)<br>• other: 42 (0.499%)
Benchmark OTU ID: `CP002623-_Proteobacteria`<br>OTU taxon: Roseobacter litoralis Och 149 [taxid 391595]<br>Expected: Roseobacter litoralis [taxid 42443] (species)<br>Number of reads: 9253<br>Number of identified reads: 9237 (99.827%)	• **species: 4076 (44.05%)**<br>• genus: 1238 (13.379%)<br>• family: 1686 (18.221%)<br>• order: 26 (0.28%)<br>• class: 431 (4.657%)<br>• phylum: 260 (2.809%)<br>• superkingdom: 333 (3.598%)<br>• root: 1180 (12.752%)	• Roseobacter denitrificans [taxid 2434]: 29 (0.313%)<br>• Sulfitobacter pseudonitzschiae [taxid 1402135]: 3 (0.032%)<br>• Shimia abyssi [taxid 1662395]: 2 (0.021%)<br>• Celeribacter halophilus [taxid 576117]: 2 (0.021%)<br>• Jannaschia helgolandensis [taxid 188906]: 2 (0.021%)<br>• Ketogulonicigenium vulgare [taxid 92945]: 2 (0.021%)<br>• Ruegeria halocynthiae [taxid 985054]: 2 (0.021%)<br>• Tritonibacter horizontis [taxid 1768241]: 1 (0.01%)<br>• other: 60 (0.648%)

Operational Taxonomic Unit (OTU)	Correct identifications	Wrong or overspecific identifications at species rank
Benchmark OTU ID: `CP002280-_Actinobacteria`<br>OTU taxon: Rothia dentocariosa ATCC 17931 [taxid 762948]<br>Expected: Rothia dentocariosa [taxid 2047] (species)<br>Number of reads: 9876<br>Number of identified reads: 9817 (99.402%)	• species: 1668 (16.889%)<br>• **genus: 5746 (58.181%)**<br>• family: 171 (1.731%)<br>• order: 103 (1.042%)<br>• class: 313 (3.169%)<br>• phylum: 8 (0.081%)<br>• superkingdom: 463 (4.688%)<br>• root: 1330 (13.466%)	• Rothia aeria [taxid 172042]: 14 (0.141%)<br>• Rothia mucilaginosa [taxid 43675]: 5 (0.05%)<br>• Candidatus Hakubanella thermoalkaliphilus [taxid 2754717]: 2 (0.02%)<br>• Alkalihalobacillus pseudofirmus [taxid 79885]: 2 (0.02%)<br>• Rothia nasimurium [taxid 85336]: 2 (0.02%)<br>• Aneurinibacillus migulanus [taxid 47500]: 1 (0.01%)<br>• Schaalia odontolytica [taxid 1660]: 1 (0.01%)<br>• Corethrella appendiculata [taxid 1370023]: 1 (0.01%)<br>• other: 18 (0.182%)
Benchmark OTU ID: `AP011540-_Actinobacteria`<br>OTU taxon: Rothia mucilaginosa DY-18 [taxid 680646]<br>Expected: Rothia mucilaginosa [taxid 43675] (species)<br>Number of reads: 8694<br>Number of identified reads: 8654 (99.539%)	• species: 848 (9.753%)<br>• **genus: 5918 (68.069%)**<br>• family: 115 (1.322%)<br>• order: 107 (1.23%)<br>• class: 291 (3.347%)<br>• phylum: 8 (0.092%)<br>• superkingdom: 399 (4.589%)<br>• root: 961 (11.053%)	• Rothia dentocariosa [taxid 2047]: 4 (0.046%)<br>• Rothia nasimurium [taxid 85336]: 2 (0.023%)<br>• Propionibacterium freudenreichii [taxid 1744]: 1 (0.011%)<br>• Panicum hallii [taxid 206008]: 1 (0.011%)<br>• Parasteatoda tepidariorum [taxid 114398]: 1 (0.011%)<br>• Bifidobacterium biavatii [taxid 762212]: 1 (0.011%)<br>• Allokutzneria albata [taxid 211114]: 1 (0.011%)<br>• Symbiodinium microadriaticum [taxid 2951]: 1 (0.011%)<br>• other: 14 (0.161%)
Benchmark OTU ID: `CP002546-_Planctomycetes`<br>OTU taxon: Rubinisphaera brasiliensis DSM 5305 [taxid 756272]<br>Expected: Rubinisphaera brasiliensis [taxid 119] (species)<br>Number of reads: 221448<br>Number of identified reads: 221088 (99.837%)	• **species: 96433 (43.546%)**<br>• genus: 41 (0.018%)<br>• family: 72139 (32.576%)<br>• order: 93 (0.041%)<br>• class: 1360 (0.614%)<br>• phylum: 2734 (1.234%)<br>• superkingdom: 15111 (6.823%)<br>• root: 32952 (14.88%)	• Planctomicrobium piriforme [taxid 1576369]: 20 (0.009%)<br>• Thalassoglobus neptunius [taxid 1938619]: 17 (0.007%)<br>• Fuerstia marisgermanicae [taxid 1891926]: 16 (0.007%)<br>• Thalassoglobus polymorphus [taxid 2527994]: 15 (0.006%)<br>• Calycomorphotria hydatis [taxid 2528027]: 14 (0.006%)<br>• Caulifigura coniformis [taxid 2527983]: 12 (0.005%)<br>• Maioricimonas rarisocia [taxid 2528026]: 11 (0.004%)<br>• Rubinisphaera italica [taxid 2527969]: 10 (0.004%)<br>• other: 438 (0.197%)
Benchmark OTU ID: `AP012320-_Proteobacteria`<br>OTU taxon: Rubrivivax gelatinosus IL144 [taxid 983917]<br>Expected: Rubrivivax gelatinosus [taxid 28068] (species)<br>Number of reads: 10463<br>Number of identified reads: 10443 (99.808%)	• **species: 5628 (53.789%)**<br>• genus: 1372 (13.112%)<br>• order: 988 (9.442%)<br>• class: 258 (2.465%)<br>• phylum: 832 (7.951%)<br>• superkingdom: 634 (6.059%)<br>• root: 727 (6.948%)	• Rubrivivax benzoatilyticus [taxid 316997]: 6 (0.057%)<br>• Pararhodospirillum photometricum [taxid 1084]: 2 (0.019%)<br>• Rubrivivax albus [taxid 2499835]: 2 (0.019%)<br>• Marivita hallyeonensis [taxid 996342]: 1 (0.009%)<br>• Escherichia coli [taxid 562]: 1 (0.009%)<br>• Micromonospora citrea [taxid 47855]: 1 (0.009%)<br>• Serpentinomonas mccroryi [taxid 1458426]: 1 (0.009%)<br>• other: 42 (0.401%)

Operational Taxonomic Unit (OTU)	Correct identifications	Wrong or overspecific identifications at species rank
Benchmark OTU ID: `CP000386-_Actinobacteria`<br>OTU taxon: Rubrobacter xylanophilus DSM 9941 [taxid 266117]<br>Expected: Rubrobacter xylanophilus [taxid 49319] (species)<br>Number of reads: 13403<br>Number of identified reads: 13334 (99.485%)	• **species: 9279 (69.23%)**<br>• genus: 286 (2.133%)<br>• family: 8 (0.059%)<br>• order: 0 (0.0%)<br>• class: 0 (0.0%)<br>• phylum: 866 (6.461%)<br>• superkingdom: 1596 (11.907%)<br>• root: 1278 (9.535%)	• Rubrobacter radiotolerans [taxid 42256]: 5 (0.037%)<br>• Rubrobacter taiwanensis [taxid 185139]: 4 (0.029%)<br>• Novibacillus thermophilus [taxid 1471761]: 1 (0.007%)<br>• Amycolatopsis vastitatis [taxid 1905142]: 1 (0.007%)<br>• Serratia marcescens [taxid 615]: 1 (0.007%)<br>• Sediminispirochaeta smaragdinae [taxid 55206]: 1 (0.007%)<br>• Bacillus thuringiensis [taxid 1428]: 1 (0.007%)<br>• Candidatus Hamiltonella defensa [taxid 138072]: 1 (0.007%)<br>• other: 25 (0.186%)
Benchmark OTU ID: `CP000031-_Proteobacteria`<br>OTU taxon: Ruegeria pomeroyi DSS-3 [taxid 246200]<br>Expected: Ruegeria pomeroyi [taxid 89184] (species)<br>Number of reads: 8362<br>Number of identified reads: 8327 (99.581%)	• **species: 4142 (49.533%)**<br>• genus: 637 (7.617%)<br>• family: 1948 (23.295%)<br>• order: 45 (0.538%)<br>• class: 339 (4.054%)<br>• phylum: 203 (2.427%)<br>• superkingdom: 324 (3.874%)<br>• root: 679 (8.12%)	• Ruegeria meonggei [taxid 1446476]: 3 (0.035%)<br>• Sulfitobacter mediterraneus [taxid 83219]: 2 (0.023%)<br>• Celeribacter baekdonensis [taxid 875171]: 2 (0.023%)<br>• Ruegeria marina [taxid 639004]: 2 (0.023%)<br>• Octadecabacter arcticus [taxid 53946]: 2 (0.023%)<br>• Roseovarius nubinhibens [taxid 314263]: 2 (0.023%)<br>• Ruegeria marisrubri [taxid 1685379]: 2 (0.023%)<br>• Phreatobacter stygius [taxid 1940610]: 1 (0.011%)<br>• other: 62 (0.741%)
Benchmark OTU ID: `CP000377-_Proteobacteria`<br>OTU taxon: Ruegeria sp. TM1040 [taxid 292414]<br>Expected: Ruegeria [taxid 97050] (genus)<br>Number of reads: 6318<br>Number of identified reads: 6293 (99.604%)	• **genus: 2798 (44.286%)**<br>• family: 2023 (32.019%)<br>• order: 36 (0.569%)<br>• class: 335 (5.302%)<br>• phylum: 155 (2.453%)<br>• superkingdom: 226 (3.577%)<br>• root: 714 (11.301%)	• Epibacterium mobile [taxid 379347]: 9 (0.142%)<br>• Phaeobacter gallaeciensis [taxid 60890]: 3 (0.047%)<br>• Tritonibacter horizontis [taxid 1768241]: 3 (0.047%)<br>• Ruegeria profundi [taxid 1685378]: 2 (0.031%)<br>• Shimia aestuarii [taxid 254406]: 2 (0.031%)<br>• Octadecabacter antarcticus [taxid 1217908]: 2 (0.031%)<br>• Pelagivirga sediminicola [taxid 2170575]: 2 (0.031%)<br>• other: 48 (0.759%)
Benchmark OTU ID: `CP002403-_Firmicutes`<br>OTU taxon: Ruminococcus albus 7 = DSM 20455 [taxid 697329]<br>Expected: Ruminococcus albus [taxid 1264] (species)<br>Number of reads: 5128<br>Number of identified reads: 5104 (99.531%)	• **species: 3505 (68.35%)**<br>• genus: 258 (5.031%)<br>• family: 37 (0.721%)<br>• order: 106 (2.067%)<br>• class: 2 (0.039%)<br>• phylum: 95 (1.852%)<br>• superkingdom: 230 (4.485%)<br>• root: 867 (16.907%)	• Ruminococcus flavefaciens [taxid 1265]: 3 (0.058%)<br>• Butyrivibrio hungatei [taxid 185008]: 1 (0.019%)<br>• Aphanomyces euteiches [taxid 100861]: 1 (0.019%)<br>• Staphylococcus aureus [taxid 1280]: 1 (0.019%)<br>• Amedibacillus dolichus [taxid 31971]: 1 (0.019%)<br>• [Clostridium] cellulosi [taxid 29343]: 1 (0.019%)<br>• Hespellia stercorisuis [taxid 180311]: 1 (0.019%)<br>• Cottoperca gobio [taxid 56716]: 1 (0.019%)

Operational Taxonomic Unit (OTU)	Correct identifications	Wrong or overspecific identifications at species rank
Benchmark OTU ID: `FP929051-_Firmicutes`<br>OTU taxon: Ruminococcus bromii L2-63 [taxid 657321]<br>Expected: Ruminococcus bromii [taxid 40518] (species)<br>Number of reads: 2797<br>Number of identified reads: 2778 (99.32%)	• species: 297 (10.618%)<br>• **genus: 1501 (53.664%)**<br>• family: 72 (2.574%)<br>• order: 274 (9.796%)<br>• class: 1 (0.035%)<br>• phylum: 136 (4.862%)<br>• superkingdom: 145 (5.184%)<br>• root: 349 (12.477%)	• Desulfonema ishimotonii [taxid 45657]: 1 (0.035%)<br>• Hungatella xylanolytica [taxid 384636]: 1 (0.035%)<br>• Paenibacillus methanolicus [taxid 582686]: 1 (0.035%)<br>• Dialister micraerophilus [taxid 309120]: 1 (0.035%)<br>• Enterocloster bolteae [taxid 208479]: 1 (0.035%)<br>• Paracoccus gahaiensis [taxid 1706839]: 1 (0.035%)<br>• Vagococcus acidifermentans [taxid 564710]: 1 (0.035%)<br>• Enterocloster clostridioformis [taxid 1531]: 1 (0.035%)<br>• other: 6 (0.214%)
Benchmark OTU ID: `CP002859-_Bacteroidetes`<br>OTU taxon: Runella slithyformis DSM 19594 [taxid 761193]<br>Expected: Runella slithyformis [taxid 106] (species)<br>Number of reads: 46111<br>Number of identified reads: 45964 (99.681%)	• species: 0 (0.0%)<br>• genus: 15668 (33.978%)<br>• family: 3534 (7.664%)<br>• order: 1073 (2.326%)<br>• class: 2 (0.004%)<br>• phylum: 4571 (9.913%)<br>• superkingdom: 4452 (9.654%)<br>• **root: 16578 (35.952%)**	• Arundinibacter roseus [taxid 2070510]: 150 (0.325%)<br>• Arcicella aurantiaca [taxid 591202]: 37 (0.08%)<br>• Arsenicibacter rosenii [taxid 1750698]: 25 (0.054%)<br>• Spirosoma montaniterrae [taxid 1178516]: 25 (0.054%)<br>• Spirosoma sordidisoli [taxid 2502893]: 24 (0.052%)<br>• Spirosoma fluviale [taxid 1597977]: 23 (0.049%)<br>• Haliscomenobacter hydrossis [taxid 2350]: 19 (0.041%)<br>• Bacteroides luti [taxid 1297750]: 18 (0.039%)<br>• other: 716 (1.552%)
Benchmark OTU ID: `CP001800-_Crenarchaeota`<br>OTU taxon: Saccharolobus solfataricus 98/2 [taxid 555311]<br>Expected: Saccharolobus solfataricus [taxid 2287] (species)<br>Number of reads: 5797<br>Number of identified reads: 5616 (96.877%)	• **species: 2777 (47.904%)**<br>• genus: 0 (0.0%)<br>• family: 1488 (25.668%)<br>• order: 7 (0.12%)<br>• class: 26 (0.448%)<br>• phylum: 0 (0.0%)<br>• superkingdom: 24 (0.414%)<br>• root: 1262 (21.769%)	• Sulfolobus islandicus [taxid 43080]: 28 (0.483%)<br>• Sulfuracidifex tepidarius [taxid 1294262]: 2 (0.034%)<br>• Sulfolobus acidocaldarius [taxid 2285]: 2 (0.034%)<br>• Sulfuracidifex metallicus [taxid 47303]: 2 (0.034%)<br>• Sulfolobales archaeon [taxid 2268200]: 1 (0.017%)<br>• Acidianus manzaensis [taxid 282676]: 1 (0.017%)<br>• Acidianus brierleyi [taxid 41673]: 1 (0.017%)<br>• Acidianus hospitalis [taxid 563177]: 1 (0.017%)<br>• other: 11 (0.189%)
Benchmark OTU ID: `AE006641-_Crenarchaeota`<br>OTU taxon: Saccharolobus solfataricus P2 [taxid 273057]<br>Expected: Saccharolobus solfataricus [taxid 2287] (species)<br>Number of reads: 6567<br>Number of identified reads: 6368 (96.969%)	• **species: 2978 (45.347%)**<br>• genus: 2 (0.03%)<br>• family: 1825 (27.79%)<br>• order: 6 (0.091%)<br>• class: 18 (0.274%)<br>• phylum: 0 (0.0%)<br>• superkingdom: 35 (0.532%)<br>• root: 1466 (22.323%)	• Sulfolobus islandicus [taxid 43080]: 34 (0.517%)<br>• Metallosphaera hakonensis [taxid 79601]: 3 (0.045%)<br>• Acidianus brierleyi [taxid 41673]: 3 (0.045%)<br>• Metallosphaera yellowstonensis [taxid 1111107]: 3 (0.045%)<br>• Sulfolobus acidocaldarius [taxid 2285]: 2 (0.03%)<br>• Sulfodiicoccus acidiphilus [taxid 1670455]: 1 (0.015%)<br>• Stygiolobus azoricus [taxid 41675]: 1 (0.015%)<br>• Pseudozyma hubeiensis [taxid 327079]: 1 (0.015%)<br>• other: 8 (0.121%)

Operational Taxonomic Unit (OTU)	Correct identifications	Wrong or overspecific identifications at species rank
Benchmark OTU ID: `tpg|BK006938.2|-_Eukaryotes`<br>OTU taxon: Saccharomyces cerevisiae S288C [taxid 559292]<br>Expected: Saccharomyces cerevisiae [taxid 4932] (species)<br>Number of reads: 58066<br>Number of identified reads: 56972 (98.115%)	• species: 4066 (7.002%)<br>• **genus: 32667 (56.258%)**<br>• family: 2618 (4.508%)<br>• order: 808 (1.391%)<br>• class: 0 (0.0%)<br>• phylum: 446 (0.768%)<br>• superkingdom: 1056 (1.818%)<br>• root: 15026 (25.877%)	• Saccharomyces arboricola [taxid 706196]: 63 (0.108%)<br>• Saccharomyces cerevisiae x Saccharomyces kudriavzevii [taxid 332112]: 57 (0.098%)<br>• Saccharomyces pastorianus [taxid 27292]: 33 (0.056%)<br>• Saccharomyces kudriavzevii [taxid 114524]: 29 (0.049%)<br>• Saccharomyces boulardii (nom. inval.) [taxid 252598]: 15 (0.025%)<br>• Trichococcus shcherbakoviae [taxid 2094020]: 14 (0.024%)<br>• Kazachstania africana [taxid 432096]: 11 (0.018%)<br>• Saccharomyces eubayanus [taxid 1080349]: 10 (0.017%)<br>• other: 154 (0.265%)
Benchmark OTU ID: `tpg|BK006948.2|-_Eukaryotes`<br>OTU taxon: Saccharomyces cerevisiae S288C [taxid 559292]<br>Expected: Saccharomyces cerevisiae [taxid 4932] (species)<br>Number of reads: 44957<br>Number of identified reads: 44086 (98.062%)	• species: 3379 (7.516%)<br>• **genus: 24469 (54.427%)**<br>• family: 1850 (4.115%)<br>• order: 584 (1.299%)<br>• class: 0 (0.0%)<br>• phylum: 377 (0.838%)<br>• superkingdom: 824 (1.832%)<br>• root: 12346 (27.461%)	• Saccharomyces arboricola [taxid 706196]: 55 (0.122%)<br>• Saccharomyces pastorianus [taxid 27292]: 35 (0.077%)<br>• Saccharomyces cerevisiae x Saccharomyces kudriavzevii [taxid 332112]: 34 (0.075%)<br>• Saccharomyces kudriavzevii [taxid 114524]: 25 (0.055%)<br>• Kazachstania naganishii [taxid 588726]: 6 (0.013%)<br>• Kazachstania africana [taxid 432096]: 6 (0.013%)<br>• [Candida] glabrata [taxid 5478]: 5 (0.011%)<br>• Kazachstania saulgeensis [taxid 1789683]: 5 (0.011%)<br>• other: 138 (0.306%)
Benchmark OTU ID: `CP000282-_Proteobacteria`<br>OTU taxon: Saccharophagus degradans 2-40 [taxid 203122]<br>Expected: Saccharophagus degradans [taxid 86304] (species)<br>Number of reads: 10495<br>Number of identified reads: 10439 (99.466%)	• **species: 7700 (73.368%)**<br>• genus: 19 (0.181%)<br>• family: 30 (0.285%)<br>• order: 29 (0.276%)<br>• class: 743 (7.079%)<br>• phylum: 290 (2.763%)<br>• superkingdom: 360 (3.43%)<br>• root: 1258 (11.986%)	• Salmonella enterica [taxid 28901]: 2 (0.019%)<br>• Buchnera aphidicola [taxid 9]: 2 (0.019%)<br>• Rhodotorula graminis [taxid 29898]: 1 (0.009%)<br>• Grimontia celer [taxid 1796497]: 1 (0.009%)<br>• Filimonas effusa [taxid 2508721]: 1 (0.009%)<br>• Methyloglobulus morosus [taxid 1410681]: 1 (0.009%)<br>• Psychrosphaera saromensis [taxid 716813]: 1 (0.009%)<br>• Marinomonas spartinae [taxid 1792290]: 1 (0.009%)<br>• other: 20 (0.19%)
Benchmark OTU ID: `CP000667-_Actinobacteria`<br>OTU taxon: Salinispora tropica CNB-440 [taxid 369723]<br>Expected: Salinispora tropica [taxid 168695] (species)<br>Number of reads: 22995<br>Number of identified reads: 22884 (99.517%)	• **species: 10431 (45.362%)**<br>• genus: 1236 (5.375%)<br>• family: 4870 (21.178%)<br>• order: 0 (0.0%)<br>• class: 2092 (9.097%)<br>• phylum: 21 (0.091%)<br>• superkingdom: 1344 (5.844%)<br>• root: 2859 (12.433%)	• Salinispora arenicola [taxid 168697]: 50 (0.217%)<br>• Micromonospora pallida [taxid 145854]: 7 (0.03%)<br>• Micromonospora pattaloongensis [taxid 405436]: 6 (0.026%)<br>• Micromonospora pisi [taxid 589240]: 5 (0.021%)<br>• Micromonospora rhizosphaerae [taxid 568872]: 5 (0.021%)<br>• Micromonospora aurantiaca [taxid 47850]: 3 (0.013%)<br>• Micromonospora matsumotoense [taxid 121616]: 3 (0.013%)<br>• Micromonospora nigra [taxid 145857]: 3 (0.013%)<br>• other: 133 (0.578%)

Operational Taxonomic Unit (OTU)	Correct identifications	Wrong or overspecific identifications at species rank
Benchmark OTU ID: `AE017220-_Pathogens`<br>OTU taxon: Salmonella enterica subsp. enterica serovar Choleraesuis str. SC-B67 [taxid 321314]<br>Expected: Salmonella enterica [taxid 28901] (species)<br>Number of reads: 10675<br>Number of identified reads: 10645 (99.718%)	• **species: 6765 (63.372%)**<br>• genus: 37 (0.346%)<br>• family: 2056 (19.259%)<br>• order: 429 (4.018%)<br>• class: 265 (2.482%)<br>• phylum: 167 (1.564%)<br>• superkingdom: 249 (2.332%)<br>• root: 669 (6.266%)	• Escherichia coli [taxid 562]: 56 (0.524%)<br>• Klebsiella pneumoniae [taxid 573]: 11 (0.103%)<br>• Shigella flexneri [taxid 623]: 7 (0.065%)<br>• Salmonella bongori [taxid 54736]: 6 (0.056%)<br>• Citrobacter freundii [taxid 546]: 5 (0.046%)<br>• Enterobacter cloacae [taxid 550]: 4 (0.037%)<br>• Shigella sonnei [taxid 624]: 3 (0.028%)<br>• Photorhabdus namnaonensis [taxid 1851568]: 3 (0.028%)<br>• other: 62 (0.58%)
Benchmark OTU ID: `CP001144-_Pathogens`<br>OTU taxon: Salmonella enterica subsp. enterica serovar Dublin str. CT_02021853 [taxid 439851]<br>Expected: Salmonella enterica [taxid 28901] (species)<br>Number of reads: 10893<br>Number of identified reads: 10849 (99.596%)	• **species: 6883 (63.187%)**<br>• genus: 40 (0.367%)<br>• family: 2109 (19.361%)<br>• order: 433 (3.975%)<br>• class: 242 (2.221%)<br>• phylum: 162 (1.487%)<br>• superkingdom: 258 (2.368%)<br>• root: 721 (6.618%)	• Escherichia coli [taxid 562]: 73 (0.67%)<br>• Klebsiella pneumoniae [taxid 573]: 11 (0.1%)<br>• Citrobacter koseri [taxid 545]: 5 (0.045%)<br>• Enterobacter cloacae [taxid 550]: 4 (0.036%)<br>• Klebsiella indica [taxid 2582917]: 2 (0.018%)<br>• Photorhabdus namnaonensis [taxid 1851568]: 2 (0.018%)<br>• Shigella sonnei [taxid 624]: 2 (0.018%)<br>• Enterobacter cancerogenus [taxid 69218]: 2 (0.018%)<br>• other: 53 (0.486%)
Benchmark OTU ID: `FM200053-_Pathogens`<br>OTU taxon: Salmonella enterica subsp. enterica serovar Paratyphi A str. AKU_12601 [taxid 554290]<br>Expected: Salmonella enterica [taxid 28901] (species)<br>Number of reads: 10240<br>Number of identified reads: 10203 (99.638%)	• **species: 6358 (62.089%)**<br>• genus: 43 (0.419%)<br>• family: 2029 (19.814%)<br>• order: 441 (4.306%)<br>• class: 213 (2.08%)<br>• phylum: 156 (1.523%)<br>• superkingdom: 217 (2.119%)<br>• root: 740 (7.226%)	• Escherichia coli [taxid 562]: 57 (0.556%)<br>• Klebsiella pneumoniae [taxid 573]: 12 (0.117%)<br>• Citrobacter freundii [taxid 546]: 5 (0.048%)<br>• Citrobacter koseri [taxid 545]: 5 (0.048%)<br>• Enterobacter cloacae [taxid 550]: 4 (0.039%)<br>• Shigella flexneri [taxid 623]: 4 (0.039%)<br>• Salmonella bongori [taxid 54736]: 4 (0.039%)<br>• Enterobacter cancerogenus [taxid 69218]: 4 (0.039%)<br>• other: 49 (0.478%)
Benchmark OTU ID: `CP000886-_Pathogens`<br>OTU taxon: Salmonella enterica subsp. enterica serovar Paratyphi B str. SPB7 [taxid 1016998]<br>Expected: Salmonella enterica [taxid 28901] (species)<br>Number of reads: 10933<br>Number of identified reads: 10898 (99.679%)	• **species: 6823 (62.407%)**<br>• genus: 33 (0.301%)<br>• family: 2283 (20.881%)<br>• order: 455 (4.161%)<br>• class: 226 (2.067%)<br>• phylum: 141 (1.289%)<br>• superkingdom: 239 (2.186%)<br>• root: 691 (6.32%)	• Escherichia coli [taxid 562]: 64 (0.585%)<br>• Klebsiella pneumoniae [taxid 573]: 6 (0.054%)<br>• Enterobacter cloacae [taxid 550]: 5 (0.045%)<br>• Citrobacter koseri [taxid 545]: 5 (0.045%)<br>• Shigella flexneri [taxid 623]: 4 (0.036%)<br>• Escherichia alba [taxid 2562891]: 3 (0.027%)<br>• Shigella sonnei [taxid 624]: 2 (0.018%)<br>• Cronobacter sakazakii [taxid 28141]: 2 (0.018%)<br>• other: 56 (0.512%)

Operational Taxonomic Unit (OTU)	Correct identifications	Wrong or overspecific identifications at species rank
Benchmark OTU ID: `CP000857-_Pathogens`<br>OTU taxon: Salmonella enterica subsp. enterica serovar Paratyphi C str. RKS4594 [taxid 476213]<br>Expected: Salmonella enterica [taxid 28901] (species)<br>Number of reads: 10869<br>Number of identified reads: 10818 (99.53%)	• **species: 6906 (63.538%)**<br>• genus: 35 (0.322%)<br>• family: 2135 (19.643%)<br>• order: 455 (4.186%)<br>• class: 245 (2.254%)<br>• phylum: 149 (1.37%)<br>• superkingdom: 225 (2.07%)<br>• root: 661 (6.081%)	• Escherichia coli [taxid 562]: 64 (0.588%)<br>• Klebsiella pneumoniae [taxid 573]: 12 (0.11%)<br>• Shigella flexneri [taxid 623]: 5 (0.046%)<br>• Salmonella bongori [taxid 54736]: 4 (0.036%)<br>• Citrobacter koseri [taxid 545]: 4 (0.036%)<br>• Enterobacter cloacae [taxid 550]: 4 (0.036%)<br>• Citrobacter freundii [taxid 546]: 4 (0.036%)<br>• Proteus penneri [taxid 102862]: 3 (0.027%)<br>• other: 69 (0.634%)
Benchmark OTU ID: `CP002099-_Pathogens`<br>OTU taxon: Salmonella enterica subsp. enterica serovar Typhi str. Ty21a [taxid 527001]<br>Expected: Salmonella enterica [taxid 28901] (species)<br>Number of reads: 10766<br>Number of identified reads: 10734 (99.702%)	• **species: 6656 (61.824%)**<br>• genus: 46 (0.427%)<br>• family: 2232 (20.731%)<br>• order: 443 (4.114%)<br>• class: 233 (2.164%)<br>• phylum: 135 (1.253%)<br>• superkingdom: 245 (2.275%)<br>• root: 738 (6.854%)	• Escherichia coli [taxid 562]: 89 (0.826%)<br>• Klebsiella pneumoniae [taxid 573]: 9 (0.083%)<br>• Citrobacter freundii [taxid 546]: 4 (0.037%)<br>• Klebsiella michiganensis [taxid 1134687]: 4 (0.037%)<br>• Citrobacter koseri [taxid 545]: 4 (0.037%)<br>• Salmonella bongori [taxid 54736]: 3 (0.027%)<br>• Shigella flexneri [taxid 623]: 3 (0.027%)<br>• Escherichia alba [taxid 2562891]: 3 (0.027%)<br>• other: 61 (0.566%)
Benchmark OTU ID: `CP003386-_Pathogens`<br>OTU taxon: Salmonella enterica subsp. enterica serovar Typhimurium str. 798 [taxid 1008297]<br>Expected: Salmonella enterica [taxid 28901] (species)<br>Number of reads: 10977<br>Number of identified reads: 10950 (99.754%)	• **species: 6935 (63.177%)**<br>• genus: 46 (0.419%)<br>• family: 2192 (19.969%)<br>• order: 440 (4.008%)<br>• class: 238 (2.168%)<br>• phylum: 140 (1.275%)<br>• superkingdom: 249 (2.268%)<br>• root: 704 (6.413%)	• Escherichia coli [taxid 562]: 61 (0.555%)<br>• Klebsiella pneumoniae [taxid 573]: 10 (0.091%)<br>• Citrobacter koseri [taxid 545]: 8 (0.072%)<br>• Shigella flexneri [taxid 623]: 4 (0.036%)<br>• Enterobacter kobei [taxid 208224]: 3 (0.027%)<br>• Enterobacter hormaechei [taxid 158836]: 3 (0.027%)<br>• Mycobacterium ahvazicum [taxid 1964395]: 2 (0.018%)<br>• Buchnera aphidicola [taxid 9]: 2 (0.018%)<br>• other: 68 (0.619%)
Benchmark OTU ID: `CP002614-_Pathogens`<br>OTU taxon: Salmonella enterica subsp. enterica serovar Typhimurium str. UK-1 [taxid 990282]<br>Expected: Salmonella enterica [taxid 28901] (species)<br>Number of reads: 10831<br>Number of identified reads: 10791 (99.63%)	• **species: 6836 (63.115%)**<br>• genus: 27 (0.249%)<br>• family: 2135 (19.711%)<br>• order: 442 (4.08%)<br>• class: 248 (2.289%)<br>• phylum: 157 (1.449%)<br>• superkingdom: 259 (2.391%)<br>• root: 681 (6.287%)	• Escherichia coli [taxid 562]: 57 (0.526%)<br>• Klebsiella pneumoniae [taxid 573]: 10 (0.092%)<br>• Citrobacter koseri [taxid 545]: 7 (0.064%)<br>• Salmonella bongori [taxid 54736]: 5 (0.046%)<br>• Shigella flexneri [taxid 623]: 4 (0.036%)<br>• Shigella sonnei [taxid 624]: 4 (0.036%)<br>• Klebsiella grimontii [taxid 2058152]: 3 (0.027%)<br>• Serratia symbiotica [taxid 138074]: 3 (0.027%)<br>• other: 52 (0.48%)

Operational Taxonomic Unit (OTU)	Correct identifications	Wrong or overspecific identifications at species rank
Benchmark OTU ID: `CP001819-_Actinobacteria`<br>OTU taxon: Sanguibacter keddieii DSM 10542 [taxid 446469]<br>Expected: Sanguibacter keddieii [taxid 60920] (species)<br>Number of reads: 18439<br>Number of identified reads: 18358 (99.56%)	• **species: 10118 (54.872%)**<br>• genus: 2129 (11.546%)<br>• family: 0 (0.0%)<br>• order: 1502 (8.145%)<br>• class: 1651 (8.953%)<br>• phylum: 18 (0.097%)<br>• superkingdom: 1253 (6.795%)<br>• root: 1665 (9.029%)	• Sanguibacter gelidistatuariae [taxid 1814289]: 6 (0.032%)<br>• Sanguibacter antarcticus [taxid 372484]: 5 (0.027%)<br>• Subtercola vilae [taxid 2056433]: 2 (0.01%)<br>• Bogoriella caseilytica [taxid 56055]: 2 (0.01%)<br>• Knoellia sinensis [taxid 136100]: 2 (0.01%)<br>• Paraoerskovia marina [taxid 545619]: 2 (0.01%)<br>• Coccidioides immitis [taxid 5501]: 2 (0.01%)<br>• Intrasporangium chromatireducens [taxid 1386088]: 2 (0.01%)<br>• other: 72 (0.39%)
Benchmark OTU ID: `CP002637-_Firmicutes`<br>OTU taxon: Selenomonas sputigena ATCC 35185 [taxid 546271]<br>Expected: Selenomonas sputigena [taxid 69823] (species)<br>Number of reads: 3340<br>Number of identified reads: 3341 (100.029%)	• **species: 1260 (37.724%)**<br>• genus: 1213 (36.317%)<br>• family: 100 (2.994%)<br>• order: 4 (0.119%)<br>• class: 26 (0.778%)<br>• phylum: 120 (3.592%)<br>• superkingdom: 239 (7.155%)<br>• root: 373 (11.167%)	• Selenomonas ruminantium [taxid 971]: 2 (0.059%)<br>• Hanusia phi [taxid 3032]: 1 (0.029%)<br>• Halobacteroides halobius [taxid 42422]: 1 (0.029%)<br>• Streptococcus pneumoniae [taxid 1313]: 1 (0.029%)<br>• Schaalia georgiae [taxid 52768]: 1 (0.029%)<br>• Sellimonas intestinalis [taxid 1653434]: 1 (0.029%)<br>• Gymnoxanthella radiolariae [taxid 1798043]: 1 (0.029%)<br>• Anaerococcus nagyae [taxid 1755241]: 1 (0.029%)<br>• other: 5 (0.149%)
Benchmark OTU ID: `CP003959-_Proteobacteria`<br>OTU taxon: Serratia marcescens WW4 [taxid 435998]<br>Expected: Serratia marcescens [taxid 615] (species)<br>Number of reads: 10909<br>Number of identified reads: 10881 (99.743%)	• species: 665 (6.095%)<br>• **genus: 5092 (46.677%)**<br>• family: 374 (3.428%)<br>• order: 2703 (24.777%)<br>• class: 439 (4.024%)<br>• phylum: 313 (2.869%)<br>• superkingdom: 353 (3.235%)<br>• root: 931 (8.534%)	• Salmonella enterica [taxid 28901]: 34 (0.311%)<br>• Serratia nematodiphila [taxid 458197]: 30 (0.275%)<br>• Serratia surfactantfaciens [taxid 2741499]: 25 (0.229%)<br>• Escherichia coli [taxid 562]: 23 (0.21%)<br>• Serratia symbiotica [taxid 138074]: 18 (0.165%)<br>• Serratia plymuthica [taxid 82996]: 14 (0.128%)<br>• Serratia liquefaciens [taxid 614]: 11 (0.1%)<br>• Serratia ficaria [taxid 61651]: 10 (0.091%)<br>• other: 139 (1.274%)
Benchmark OTU ID: `CP002773-_Proteobacteria`<br>OTU taxon: Serratia plymuthica AS9 [taxid 768492]<br>Expected: Serratia plymuthica [taxid 82996] (species)<br>Number of reads: 11362<br>Number of identified reads: 11320 (99.63%)	• **species: 2991 (26.324%)**<br>• genus: 2733 (24.053%)<br>• family: 473 (4.162%)<br>• order: 2566 (22.584%)<br>• class: 472 (4.154%)<br>• phylum: 338 (2.974%)<br>• superkingdom: 390 (3.432%)<br>• root: 1344 (11.828%)	• Serratia marcescens [taxid 615]: 51 (0.448%)<br>• Serratia proteamaculans [taxid 28151]: 50 (0.44%)<br>• Salmonella enterica [taxid 28901]: 28 (0.246%)<br>• Serratia liquefaciens [taxid 614]: 26 (0.228%)<br>• Escherichia coli [taxid 562]: 25 (0.22%)<br>• Serratia quinivorans [taxid 137545]: 23 (0.202%)<br>• Serratia fonticola [taxid 47917]: 16 (0.14%)<br>• Serratia symbiotica [taxid 138074]: 15 (0.132%)<br>• other: 148 (1.302%)

Operational Taxonomic Unit (OTU)	Correct identifications	Wrong or overspecific identifications at species rank
Benchmark OTU ID: `CP000826-_Proteobacteria`<br>OTU taxon: Serratia proteamaculans 568 [taxid 399741]<br>Expected: Serratia proteamaculans [taxid 28151] (species)<br>Number of reads: 11376<br>Number of identified reads: 11344 (99.718%)	• species: 899 (7.902%)<br>• **genus: 5343 (46.967%)**<br>• family: 425 (3.735%)<br>• order: 2494 (21.923%)<br>• class: 465 (4.087%)<br>• phylum: 293 (2.575%)<br>• superkingdom: 363 (3.19%)<br>• root: 1059 (9.309%)	• Serratia quinivorans [taxid 137545]: 50 (0.439%)<br>• Salmonella enterica [taxid 28901]: 39 (0.342%)<br>• Serratia plymuthica [taxid 82996]: 38 (0.334%)<br>• Escherichia coli [taxid 562]: 20 (0.175%)<br>• Serratia fonticola [taxid 47917]: 18 (0.158%)<br>• Serratia liquefaciens [taxid 614]: 16 (0.14%)<br>• Serratia marcescens [taxid 615]: 16 (0.14%)<br>• Serratia symbiotica [taxid 138074]: 14 (0.123%)<br>• other: 128 (1.125%)
Benchmark OTU ID: `CP002775-_Proteobacteria`<br>OTU taxon: Serratia sp. AS13 [taxid 768493]<br>Expected: Serratia [taxid 613] (genus)<br>Number of reads: 11362<br>Number of identified reads: 11331 (99.727%)	• **genus: 5575 (49.067%)**<br>• family: 503 (4.427%)<br>• order: 2621 (23.068%)<br>• class: 481 (4.233%)<br>• phylum: 335 (2.948%)<br>• superkingdom: 389 (3.423%)<br>• root: 1421 (12.506%)	• **Serratia plymuthica [taxid 82996]: 2918 (25.682%)**<br>• Serratia proteamaculans [taxid 28151]: 54 (0.475%)<br>• Serratia marcescens [taxid 615]: 41 (0.36%)<br>• Salmonella enterica [taxid 28901]: 39 (0.343%)<br>• Escherichia coli [taxid 562]: 23 (0.202%)<br>• Serratia quinivorans [taxid 137545]: 22 (0.193%)<br>• Serratia liquefaciens [taxid 614]: 18 (0.158%)<br>• other: 151 (1.328%)
Benchmark OTU ID: `CP002295-_Proteobacteria`<br>OTU taxon: Serratia symbiotica str. 'Cinara cedri' [taxid 568817]<br>Expected: Serratia symbiotica [taxid 138074] (species)<br>Number of reads: 3082<br>Number of identified reads: 2351 (76.281%)	• species: 981 (31.829%)<br>• genus: 6 (0.194%)<br>• family: 7 (0.227%)<br>• order: 141 (4.574%)<br>• class: 103 (3.341%)<br>• phylum: 31 (1.005%)<br>• superkingdom: 58 (1.881%)<br>• **root: 1016 (32.965%)**	• Photobacterium lutimaris [taxid 388278]: 1 (0.032%)<br>• secondary endosymbiont of Ctenarytaina eucalypti [taxid 1199245]: 1 (0.032%)<br>• Legionella feeleii [taxid 453]: 1 (0.032%)<br>• Blochmannia endosymbiont of Polyrhachis (Hedomyrma) turneri [taxid 1505596]: 1 (0.032%)<br>• Arsenophonus endosymbiont of Trialeurodes vaporariorum [taxid 235567]: 1 (0.032%)<br>• Arthrobotrys oligospora [taxid 13349]: 1 (0.032%)<br>• Pasteurella canis [taxid 753]: 1 (0.032%)<br>• Gemmobacter intermedius [taxid 1553448]: 1 (0.032%)<br>• other: 3 (0.097%)
Benchmark OTU ID: `AE014299-_Proteobacteria`<br>OTU taxon: Shewanella oneidensis MR-1 [taxid 211586]<br>Expected: Shewanella oneidensis [taxid 70863] (species)<br>Number of reads: 10298<br>Number of identified reads: 10236 (99.397%)	• species: 3390 (32.919%)<br>• **genus: 4229 (41.066%)**<br>• family: 13 (0.126%)<br>• order: 155 (1.505%)<br>• class: 773 (7.506%)<br>• phylum: 219 (2.126%)<br>• superkingdom: 287 (2.786%)<br>• root: 1164 (11.303%)	• Shewanella xiamenensis [taxid 332186]: 27 (0.262%)<br>• Shewanella baltica [taxid 62322]: 18 (0.174%)<br>• Shewanella decolorationis [taxid 256839]: 15 (0.145%)<br>• Shewanella putrefaciens [taxid 24]: 14 (0.135%)<br>• Shewanella morhuae [taxid 365591]: 13 (0.126%)<br>• Shewanella hanedai [taxid 25]: 6 (0.058%)<br>• Shewanella fodinae [taxid 552357]: 4 (0.038%)<br>• Shewanella maritima [taxid 2520507]: 4 (0.038%)<br>• other: 74 (0.718%)

Operational Taxonomic Unit (OTU)	Correct identifications	Wrong or overspecific identifications at species rank
Benchmark OTU ID: `CP000034-_Proteobacteria`<br>OTU taxon: Shigella dysenteriae Sd197 [taxid 300267]<br>Expected: Shigella dysenteriae [taxid 622] (species)<br>Number of reads: 8947<br>Number of identified reads: 8925 (99.754%)	• species: 889 (9.936%)<br>• genus: 236 (2.637%)<br>• **family: 6282 (70.213%)**<br>• order: 468 (5.23%)<br>• class: 207 (2.313%)<br>• phylum: 129 (1.441%)<br>• superkingdom: 209 (2.335%)<br>• root: 502 (5.61%)	• **Escherichia coli [taxid 562]: 928 (10.372%)**<br>• Salmonella enterica [taxid 28901]: 125 (1.397%)<br>• Shigella flexneri [taxid 623]: 64 (0.715%)<br>• Shigella sonnei [taxid 624]: 62 (0.692%)<br>• Klebsiella pneumoniae [taxid 573]: 16 (0.178%)<br>• Shigella boydii [taxid 621]: 12 (0.134%)<br>• Citrobacter amalonaticus [taxid 35703]: 7 (0.078%)<br>• Escherichia albertii [taxid 208962]: 6 (0.067%)<br>• other: 90 (1.005%)
Benchmark OTU ID: `CP001383-_Proteobacteria`<br>OTU taxon: Shigella flexneri 2002017 [taxid 591020]<br>Expected: Shigella flexneri [taxid 623] (species)<br>Number of reads: 9579<br>Number of identified reads: 9557 (99.77%)	• species: 235 (2.453%)<br>• genus: 530 (5.532%)<br>• **family: 7183 (74.986%)**<br>• order: 439 (4.582%)<br>• class: 241 (2.515%)<br>• phylum: 146 (1.524%)<br>• superkingdom: 210 (2.192%)<br>• root: 570 (5.95%)	• **Escherichia coli [taxid 562]: 1063 (11.097%)**<br>• Salmonella enterica [taxid 28901]: 151 (1.576%)<br>• Shigella sonnei [taxid 624]: 64 (0.668%)<br>• Shigella dysenteriae [taxid 622]: 30 (0.313%)<br>• Klebsiella pneumoniae [taxid 573]: 18 (0.187%)<br>• Shigella boydii [taxid 621]: 14 (0.146%)<br>• Escherichia fergusonii [taxid 564]: 11 (0.114%)<br>• Escherichia albertii [taxid 208962]: 8 (0.083%)<br>• other: 112 (1.169%)
Benchmark OTU ID: `CP000266-_Proteobacteria`<br>OTU taxon: Shigella flexneri 5 str. 8401 [taxid 373384]<br>Expected: Shigella flexneri [taxid 623] (species)<br>Number of reads: 9408<br>Number of identified reads: 9382 (99.723%)	• species: 346 (3.677%)<br>• genus: 498 (5.293%)<br>• **family: 7000 (74.404%)**<br>• order: 434 (4.613%)<br>• class: 196 (2.083%)<br>• phylum: 145 (1.541%)<br>• superkingdom: 199 (2.115%)<br>• root: 560 (5.952%)	• **Escherichia coli [taxid 562]: 1071 (11.383%)**<br>• Salmonella enterica [taxid 28901]: 148 (1.573%)<br>• Shigella sonnei [taxid 624]: 69 (0.733%)<br>• Shigella dysenteriae [taxid 622]: 39 (0.414%)<br>• Klebsiella pneumoniae [taxid 573]: 21 (0.223%)<br>• Shigella boydii [taxid 621]: 20 (0.212%)<br>• Escherichia fergusonii [taxid 564]: 9 (0.095%)<br>• Enterobacter cloacae [taxid 550]: 8 (0.085%)<br>• other: 101 (1.073%)
Benchmark OTU ID: `HE616528-_Proteobacteria`<br>OTU taxon: Shigella sonnei 53G [taxid 216599]<br>Expected: Shigella sonnei [taxid 624] (species)<br>Number of reads: 10340<br>Number of identified reads: 10310 (99.709%)	• species: 327 (3.162%)<br>• genus: 264 (2.553%)<br>• **family: 8166 (78.974%)**<br>• order: 434 (4.197%)<br>• class: 184 (1.779%)<br>• phylum: 153 (1.479%)<br>• superkingdom: 222 (2.147%)<br>• root: 556 (5.377%)	• **Escherichia coli [taxid 562]: 1444 (13.965%)**<br>• Salmonella enterica [taxid 28901]: 150 (1.45%)<br>• Shigella flexneri [taxid 623]: 85 (0.822%)<br>• Shigella dysenteriae [taxid 622]: 46 (0.444%)<br>• Shigella boydii [taxid 621]: 16 (0.154%)<br>• Klebsiella pneumoniae [taxid 573]: 15 (0.145%)<br>• Citrobacter freundii [taxid 546]: 7 (0.067%)<br>• Citrobacter koseri [taxid 545]: 5 (0.048%)<br>• other: 126 (1.218%)

Operational Taxonomic Unit (OTU)	Correct identifications	Wrong or overspecific identifications at species rank
Benchmark OTU ID: `CP001560-_Proteobacteria`<br>OTU taxon: Shimwellia blattae DSM 4481 = NBRC 105725 [taxid 630626]<br>Expected: Shimwellia blattae [taxid 563] (species)<br>Number of reads: 8473<br>Number of identified reads: 8438 (99.586%)	• **species: 4392 (51.835%)**<br>• genus: 0 (0.0%)<br>• family: 1446 (17.065%)<br>• order: 923 (10.893%)<br>• class: 364 (4.295%)<br>• phylum: 210 (2.478%)<br>• superkingdom: 254 (2.997%)<br>• root: 841 (9.925%)	• Salmonella enterica [taxid 28901]: 73 (0.861%)<br>• Escherichia coli [taxid 562]: 39 (0.46%)<br>• Klebsiella pneumoniae [taxid 573]: 8 (0.094%)<br>• Pararhodospirillum photometricum [taxid 1084]: 3 (0.035%)<br>• Azospirillum brasilense [taxid 192]: 3 (0.035%)<br>• Klebsiella quasipneumoniae [taxid 1463165]: 3 (0.035%)<br>• Escherichia fergusonii [taxid 564]: 2 (0.023%)<br>• Photorhabdus namnaonensis [taxid 1851568]: 2 (0.023%)<br>• other: 62 (0.731%)
Benchmark OTU ID: `CP001965-_Proteobacteria`<br>OTU taxon: Sideroxydans lithotrophicus ES-1 [taxid 580332]<br>Expected: Sideroxydans lithotrophicus [taxid 63745] (species)<br>Number of reads: 5874<br>Number of identified reads: 5865 (99.846%)	• **species: 3449 (58.716%)**<br>• genus: 13 (0.221%)<br>• family: 117 (1.991%)<br>• order: 106 (1.804%)<br>• class: 372 (6.332%)<br>• phylum: 680 (11.576%)<br>• superkingdom: 332 (5.652%)<br>• root: 788 (13.415%)	• Candidatus Gallionella acididurans [taxid 1796491]: 2 (0.034%)<br>• Stenotrophomonas maltophilia [taxid 40324]: 2 (0.034%)<br>• Alexandrium catenella [taxid 2925]: 1 (0.017%)<br>• Halioglobus lutimaris [taxid 1737061]: 1 (0.017%)<br>• Paralcaligenes ureilyticus [taxid 627131]: 1 (0.017%)<br>• Salmonella enterica [taxid 28901]: 1 (0.017%)<br>• Thiomonas delicata [taxid 364030]: 1 (0.017%)<br>• Candidatus Cloacimonas acidaminovorans [taxid 456827]: 1 (0.017%)<br>• other: 20 (0.34%)
Benchmark OTU ID: `CP003746-_Proteobacteria`<br>OTU taxon: Simiduia agarivorans SA1 = DSM 21679 [taxid 1117647]<br>Expected: Simiduia agarivorans [taxid 447471] (species)<br>Number of reads: 8813<br>Number of identified reads: 8791 (99.75%)	• **species: 6473 (73.448%)**<br>• genus: 0 (0.0%)<br>• family: 28 (0.317%)<br>• order: 63 (0.714%)<br>• class: 725 (8.226%)<br>• phylum: 321 (3.642%)<br>• superkingdom: 328 (3.721%)<br>• root: 848 (9.622%)	• Wenzhouxiangella marina [taxid 1579979]: 1 (0.011%)<br>• Umboniibacter marinipuniceus [taxid 569599]: 1 (0.011%)<br>• Sinobacterium caligoides [taxid 933926]: 1 (0.011%)<br>• Alcanivorax hongdengensis [taxid 519051]: 1 (0.011%)<br>• Salmonella enterica [taxid 28901]: 1 (0.011%)<br>• Candidatus Rickettsiella viridis [taxid 676208]: 1 (0.011%)<br>• Microbulbifer yueqingensis [taxid 658219]: 1 (0.011%)<br>• Hwanghaeicola aestuarii [taxid 568105]: 1 (0.011%)<br>• other: 19 (0.215%)
Benchmark OTU ID: `CP003364-_Planctomycetes`<br>OTU taxon: Singulisphaera acidiphila DSM 18658 [taxid 886293]<br>Expected: Singulisphaera acidiphila [taxid 466153] (species)<br>Number of reads: 374701<br>Number of identified reads: 371633 (99.181%)	• **species: 215054 (57.393%)**<br>• genus: 43841 (11.7%)<br>• family: 2699 (0.72%)<br>• order: 0 (0.0%)<br>• class: 4145 (1.106%)<br>• phylum: 3331 (0.888%)<br>• superkingdom: 31407 (8.381%)<br>• root: 70626 (18.848%)	• Tautonia plasticadhaerens [taxid 2527974]: 62 (0.016%)<br>• Aquisphaera giovannonii [taxid 406548]: 53 (0.014%)<br>• Paludisphaera borealis [taxid 1387353]: 35 (0.009%)<br>• Beta vulgaris [taxid 161934]: 27 (0.007%)<br>• [Clostridium] spiroforme [taxid 29348]: 18 (0.004%)<br>• bacterium [taxid 1869227]: 10 (0.002%)<br>• Tautonia sociabilis [taxid 2080755]: 10 (0.002%)<br>• Blastopirellula marina [taxid 124]: 9 (0.002%)<br>• other: 812 (0.216%)

Operational Taxonomic Unit (OTU)	Correct identifications	Wrong or overspecific identifications at species rank
Benchmark OTU ID: `AL591688-_Proteobacteria`<br>OTU taxon: Sinorhizobium meliloti 1021 [taxid 266834]<br>Expected: Sinorhizobium meliloti [taxid 382] (species)<br>Number of reads: 7338<br>Number of identified reads: 7307 (99.577%)	• species: 372 (5.069%)<br>• genus: 526 (7.168%)<br>• **family: 2902 (39.547%)**<br>• order: 1996 (27.2%)<br>• class: 271 (3.693%)<br>• phylum: 183 (2.493%)<br>• superkingdom: 280 (3.815%)<br>• root: 771 (10.506%)	• Sinorhizobium medicae [taxid 110321]: 43 (0.585%)<br>• Sinorhizobium fredii [taxid 380]: 15 (0.204%)<br>• Sinorhizobium saheli [taxid 36856]: 5 (0.068%)<br>• Ensifer sojae [taxid 716925]: 4 (0.054%)<br>• Ensifer glycinis [taxid 1472378]: 4 (0.054%)<br>• Rhizobium oryzae [taxid 464029]: 3 (0.04%)<br>• Ensifer adhaerens [taxid 106592]: 3 (0.04%)<br>• Lupinus albus [taxid 3870]: 3 (0.04%)<br>• other: 53 (0.722%)
Benchmark OTU ID: `CP002740-_Proteobacteria`<br>OTU taxon: Sinorhizobium meliloti BL225C [taxid 698936]<br>Expected: Sinorhizobium meliloti [taxid 382] (species)<br>Number of reads: 7378<br>Number of identified reads: 7348 (99.593%)	• species: 373 (5.055%)<br>• genus: 544 (7.373%)<br>• **family: 2915 (39.509%)**<br>• order: 2076 (28.137%)<br>• class: 238 (3.225%)<br>• phylum: 156 (2.114%)<br>• superkingdom: 264 (3.578%)<br>• root: 775 (10.504%)	• Sinorhizobium medicae [taxid 110321]: 32 (0.433%)<br>• Sinorhizobium fredii [taxid 380]: 16 (0.216%)<br>• Sinorhizobium saheli [taxid 36856]: 6 (0.081%)<br>• Ensifer alkalisoli [taxid 1752398]: 5 (0.067%)<br>• Agrobacterium tumefaciens [taxid 358]: 4 (0.054%)<br>• Pararhizobium antarcticum [taxid 1798805]: 3 (0.04%)<br>• Sinorhizobium americanum [taxid 194963]: 3 (0.04%)<br>• Neorhizobium galegae [taxid 399]: 3 (0.04%)<br>• other: 60 (0.813%)
Benchmark OTU ID: `CP003933-_Proteobacteria`<br>OTU taxon: Sinorhizobium meliloti GR4 [taxid 1235461]<br>Expected: Sinorhizobium meliloti [taxid 382] (species)<br>Number of reads: 7254<br>Number of identified reads: 7234 (99.724%)	• species: 231 (3.184%)<br>• genus: 547 (7.54%)<br>• **family: 2884 (39.757%)**<br>• order: 2039 (28.108%)<br>• class: 259 (3.57%)<br>• phylum: 176 (2.426%)<br>• superkingdom: 277 (3.818%)<br>• root: 807 (11.124%)	• Sinorhizobium medicae [taxid 110321]: 28 (0.385%)<br>• Sinorhizobium fredii [taxid 380]: 15 (0.206%)<br>• Ensifer glycinis [taxid 1472378]: 7 (0.096%)<br>• Sinorhizobium americanum [taxid 194963]: 5 (0.068%)<br>• Lupinus albus [taxid 3870]: 4 (0.055%)<br>• Rhizobium leguminosarum [taxid 384]: 4 (0.055%)<br>• Ensifer sojae [taxid 716925]: 4 (0.055%)<br>• Ensifer adhaerens [taxid 106592]: 3 (0.041%)<br>• other: 63 (0.868%)
Benchmark OTU ID: `HE995405-_Proteobacteria`<br>OTU taxon: Sinorhizobium meliloti Rm41 [taxid 1230587]<br>Expected: Sinorhizobium meliloti [taxid 382] (species)<br>Number of reads: 7394<br>Number of identified reads: 7372 (99.702%)	• species: 409 (5.531%)<br>• genus: 581 (7.857%)<br>• **family: 2931 (39.64%)**<br>• order: 1939 (26.223%)<br>• class: 230 (3.11%)<br>• phylum: 152 (2.055%)<br>• superkingdom: 272 (3.678%)<br>• root: 843 (11.401%)	• Sinorhizobium medicae [taxid 110321]: 38 (0.513%)<br>• Sinorhizobium fredii [taxid 380]: 11 (0.148%)<br>• Sinorhizobium americanum [taxid 194963]: 6 (0.081%)<br>• Sinorhizobium saheli [taxid 36856]: 6 (0.081%)<br>• Lupinus albus [taxid 3870]: 5 (0.067%)<br>• Rhizobium leguminosarum [taxid 384]: 5 (0.067%)<br>• Sinorhizobium terangae [taxid 110322]: 4 (0.054%)<br>• Mesorhizobium loti [taxid 381]: 4 (0.054%)<br>• other: 69 (0.933%)

Operational Taxonomic Unit (OTU)	Correct identifications	Wrong or overspecific identifications at species rank
Benchmark OTU ID: `CP001830-_Proteobacteria`<br>OTU taxon: Sinorhizobium meliloti SM11 [taxid 707241]<br>Expected: Sinorhizobium meliloti [taxid 382] (species)<br>Number of reads: 7909<br>Number of identified reads: 7878 (99.608%)	• species: 388 (4.905%)<br>• genus: 628 (7.94%)<br>• **family: 2933 (37.084%)**<br>• order: 2300 (29.08%)<br>• class: 246 (3.11%)<br>• phylum: 167 (2.111%)<br>• superkingdom: 285 (3.603%)<br>• root: 922 (11.657%)	• Sinorhizobium medicae [taxid 110321]: 28 (0.354%)<br>• Sinorhizobium fredii [taxid 380]: 14 (0.177%)<br>• Mesorhizobium loti [taxid 381]: 9 (0.113%)<br>• Rhizobium leguminosarum [taxid 384]: 7 (0.088%)<br>• Lupinus albus [taxid 3870]: 6 (0.075%)<br>• Ensifer alkalisoli [taxid 1752398]: 4 (0.05%)<br>• Agrobacterium salinitolerans [taxid 1183413]: 3 (0.037%)<br>• Sinorhizobium americanum [taxid 194963]: 3 (0.037%)<br>• other: 52 (0.657%)
Benchmark OTU ID: `CP001684-_Actinobacteria`<br>OTU taxon: Slackia heliotrinireducens DSM 20476 [taxid 471855]<br>Expected: Slackia heliotrinireducens [taxid 84110] (species)<br>Number of reads: 13106<br>Number of identified reads: 13069 (99.717%)	• **species: 9608 (73.309%)**<br>• genus: 80 (0.61%)<br>• family: 101 (0.77%)<br>• order: 2 (0.015%)<br>• class: 246 (1.877%)<br>• phylum: 207 (1.579%)<br>• superkingdom: 1284 (9.797%)<br>• root: 1534 (11.704%)	• Collinsella aerofaciens [taxid 74426]: 4 (0.03%)<br>• Aeromonas hydrophila [taxid 644]: 2 (0.015%)<br>• Lysobacter dokdonensis [taxid 414050]: 1 (0.007%)<br>• Anaerococcus tetradius [taxid 33036]: 1 (0.007%)<br>• Adlercreutzia caecicola [taxid 747645]: 1 (0.007%)<br>• Acanthochromis polyacanthus [taxid 80966]: 1 (0.007%)<br>• Rhodovulum robiginosum [taxid 68292]: 1 (0.007%)<br>• Candidatus Ishikawaella capsulata [taxid 168169]: 1 (0.007%)<br>• other: 21 (0.16%)
Benchmark OTU ID: `AP008232-_Proteobacteria`<br>OTU taxon: Sodalis glossinidius str. 'morsitans' [taxid 343509]<br>Expected: Sodalis glossinidius [taxid 63612] (species)<br>Number of reads: 8501<br>Number of identified reads: 8441 (99.294%)	• **species: 2666 (31.361%)**<br>• genus: 1017 (11.963%)<br>• family: 135 (1.588%)<br>• order: 1628 (19.15%)<br>• class: 376 (4.423%)<br>• phylum: 224 (2.634%)<br>• superkingdom: 355 (4.175%)<br>• root: 1989 (23.397%)	• **Sodalis praecaptivus [taxid 1239307]: 230 (2.705%)**<br>• Salmonella enterica [taxid 28901]: 21 (0.247%)<br>• Glossina pallidipes [taxid 7398]: 17 (0.199%)<br>• Candidatus Sodalis pierantonius [taxid 1486991]: 16 (0.188%)<br>• Glossina brevipalpis [taxid 37001]: 15 (0.176%)<br>• Plautia stali symbiont [taxid 891974]: 12 (0.141%)<br>• Escherichia coli [taxid 562]: 10 (0.117%)<br>• Serratia symbiotica [taxid 138074]: 6 (0.07%)<br>• other: 133 (1.564%)
Benchmark OTU ID: `CP003349-_Bacteroidetes`<br>OTU taxon: Solitalea canadensis DSM 3403 [taxid 929556]<br>Expected: Solitalea canadensis [taxid 995] (species)<br>Number of reads: 35724<br>Number of identified reads: 35351 (98.955%)	• **species: 25589 (71.629%)**<br>• genus: 1002 (2.804%)<br>• family: 657 (1.839%)<br>• order: 42 (0.117%)<br>• class: 2 (0.005%)<br>• phylum: 2673 (7.482%)<br>• superkingdom: 1652 (4.624%)<br>• root: 3700 (10.357%)	• Solitalea longa [taxid 2079460]: 38 (0.106%)<br>• Solitalea koreensis [taxid 543615]: 10 (0.027%)<br>• Lupinus albus [taxid 3870]: 5 (0.013%)<br>• Prevotella pallens [taxid 60133]: 4 (0.011%)<br>• Leptomonas pyrrhocoris [taxid 157538]: 3 (0.008%)<br>• Ephemera danica [taxid 1049336]: 3 (0.008%)<br>• Parapedobacter luteus [taxid 623280]: 2 (0.005%)<br>• Pseudopedobacter saltans [taxid 151895]: 2 (0.005%)<br>• other: 86 (0.24%)

Operational Taxonomic Unit (OTU)	Correct identifications	Wrong or overspecific identifications at species rank
Benchmark OTU ID: `CP001823-_Chloroflexi`<br>OTU taxon: Sphaerobacter thermophilus DSM 20745 [taxid 479434]<br>Expected: Sphaerobacter thermophilus [taxid 2057] (species)<br>Number of reads: 64025<br>Number of identified reads: 63374 (98.983%)	• **species: 45236 (70.653%)**<br>• genus: 0 (0.0%)<br>• family: 232 (0.362%)<br>• order: 0 (0.0%)<br>• class: 135 (0.21%)<br>• phylum: 718 (1.121%)<br>• superkingdom: 9813 (15.326%)<br>• root: 7180 (11.214%)	• Nitrolancea hollandica [taxid 1206749]: 9 (0.014%)<br>• Litorilinea aerophila [taxid 1204385]: 4 (0.006%)<br>• bacterium [taxid 1869227]: 3 (0.004%)<br>• Candidatus Koribacter versatilis [taxid 658062]: 3 (0.004%)<br>• Aureliella helgolandensis [taxid 2527968]: 2 (0.003%)<br>• Acidisphaera rubrifaciens [taxid 50715]: 2 (0.003%)<br>• Chondromyces apiculatus [taxid 51]: 2 (0.003%)<br>• Ditylum brightwellii [taxid 49249]: 2 (0.003%)<br>• other: 157 (0.245%)
Benchmark OTU ID: `CP001824-_Chloroflexi`<br>OTU taxon: Sphaerobacter thermophilus DSM 20745 [taxid 479434]<br>Expected: Sphaerobacter thermophilus [taxid 2057] (species)<br>Number of reads: 22799<br>Number of identified reads: 22520 (98.776%)	• **species: 16341 (71.674%)**<br>• genus: 0 (0.0%)<br>• family: 39 (0.171%)<br>• order: 0 (0.0%)<br>• class: 31 (0.135%)<br>• phylum: 212 (0.929%)<br>• superkingdom: 3303 (14.487%)<br>• root: 2568 (11.263%)	• bacterium [taxid 1869227]: 7 (0.03%)<br>• Chondromyces crocatus [taxid 52]: 3 (0.013%)<br>• Nitrolancea hollandica [taxid 1206749]: 2 (0.008%)<br>• Nitrospira moscoviensis [taxid 42253]: 2 (0.008%)<br>• Kouleothrix aurantiaca [taxid 186479]: 1 (0.004%)<br>• Plesiomonas shigelloides [taxid 703]: 1 (0.004%)<br>• Micromonospora phaseoli [taxid 1144548]: 1 (0.004%)<br>• Morganella morganii [taxid 582]: 1 (0.004%)<br>• other: 48 (0.21%)
Benchmark OTU ID: `CP002798-_Proteobacteria`<br>OTU taxon: Sphingobium chlorophenolicum L-1 [taxid 690566]<br>Expected: Sphingobium chlorophenolicum [taxid 46429] (species)<br>Number of reads: 6048<br>Number of identified reads: 6021 (99.553%)	• **species: 2247 (37.152%)**<br>• genus: 1568 (25.925%)<br>• family: 837 (13.839%)<br>• order: 156 (2.579%)<br>• class: 204 (3.373%)<br>• phylum: 166 (2.744%)<br>• superkingdom: 240 (3.968%)<br>• root: 595 (9.837%)	• Sphingobium faniae [taxid 570446]: 4 (0.066%)<br>• Sphingobium japonicum [taxid 332056]: 2 (0.033%)<br>• Sphingobium fluviale [taxid 2506423]: 2 (0.033%)<br>• Caballeronia arationis [taxid 1777142]: 1 (0.016%)<br>• Haemophilus pittmaniae [taxid 249188]: 1 (0.016%)<br>• Corchorus olitorius [taxid 93759]: 1 (0.016%)<br>• Solitalea koreensis [taxid 543615]: 1 (0.016%)<br>• Sphingomonas indica [taxid 941907]: 1 (0.016%)<br>• other: 27 (0.446%)
Benchmark OTU ID: `AP012222-_Proteobacteria`<br>OTU taxon: Sphingobium sp. SYK-6 [taxid 627192]<br>Expected: Sphingobium [taxid 165695] (genus)<br>Number of reads: 8564<br>Number of identified reads: 8533 (99.638%)	• **genus: 5898 (68.869%)**<br>• family: 513 (5.99%)<br>• order: 241 (2.814%)<br>• class: 372 (4.343%)<br>• phylum: 256 (2.989%)<br>• superkingdom: 386 (4.507%)<br>• root: 860 (10.042%)	• Sphingobium algorifonticola [taxid 2008318]: 3 (0.035%)<br>• Lupinus albus [taxid 3870]: 2 (0.023%)<br>• Novosphingobium mathurense [taxid 428990]: 2 (0.023%)<br>• Sphingobium yanoikuyae [taxid 13690]: 2 (0.023%)<br>• Sphingopyxis sp. HIX [taxid 1759074]: 1 (0.011%)<br>• Algoriphagus boseongensis [taxid 1442587]: 1 (0.011%)<br>• Sphingomonas sanxanigenens [taxid 397260]: 1 (0.011%)<br>• other: 28 (0.326%)

Operational Taxonomic Unit (OTU)	Correct identifications	Wrong or overspecific identifications at species rank
Benchmark OTU ID: `CP000699-_Proteobacteria`<br>OTU taxon: Sphingomonas wittichii RW1 [taxid 392499]<br>Expected: Sphingomonas wittichii [taxid 160791] (species)<br>Number of reads: 11226<br>Number of identified reads: 11201 (99.777%)	• **species: 4779 (42.57%)**<br>• genus: 2910 (25.921%)<br>• family: 967 (8.613%)<br>• order: 258 (2.298%)<br>• class: 481 (4.284%)<br>• phylum: 383 (3.411%)<br>• superkingdom: 558 (4.97%)<br>• root: 857 (7.634%)	• Sphingomonas histidinilytica [taxid 439228]: 13 (0.115%)<br>• Rhizorhabdus dicambivorans [taxid 1850238]: 4 (0.035%)<br>• Sphingomonas sanxanigenens [taxid 397260]: 2 (0.017%)<br>• Sphingomonas laterariae [taxid 861865]: 2 (0.017%)<br>• Sphingobium ummariense [taxid 420994]: 1 (0.008%)<br>• Sphingobium herbicidovorans [taxid 76947]: 1 (0.008%)<br>• Aestuariisphingobium litorale [taxid 2339262]: 1 (0.008%)<br>• Leucothrix pacifica [taxid 1247513]: 1 (0.008%)<br>• other: 33 (0.293%)
Benchmark OTU ID: `CP003653-_Cyanobacteria`<br>OTU taxon: Stanieria cyanosphaera PCC 7437 [taxid 111780]<br>Expected: Stanieria cyanosphaera [taxid 102116] (species)<br>Number of reads: 29412<br>Number of identified reads: 28917 (98.317%)	• **species: 16413 (55.803%)**<br>• genus: 3686 (12.532%)<br>• family: 0 (0.0%)<br>• order: 292 (0.992%)<br>• phylum: 3478 (11.825%)<br>• superkingdom: 1658 (5.637%)<br>• root: 3330 (11.321%)	• Hyella patelloides [taxid 1982969]: 9 (0.03%)<br>• Crocosphaera subtropica [taxid 2546360]: 5 (0.016%)<br>• Hydrococcus rivularis [taxid 1616834]: 3 (0.01%)<br>• Lupinus albus [taxid 3870]: 3 (0.01%)<br>• Prochlorococcus marinus [taxid 1219]: 3 (0.01%)<br>• Rubidibacter lacunae [taxid 582514]: 3 (0.01%)<br>• Gloeothece verrucosa [taxid 2546359]: 2 (0.006%)<br>• other: 85 (0.288%)
Benchmark OTU ID: `CP003668-_Firmicutes`<br>OTU taxon: Staphylococcus warneri SG1 [taxid 1194526]<br>Expected: Staphylococcus warneri [taxid 1292] (species)<br>Number of reads: 3209<br>Number of identified reads: 3170 (98.784%)	• species: 59 (1.838%)<br>• **genus: 2544 (79.277%)**<br>• family: 49 (1.526%)<br>• order: 63 (1.963%)<br>• class: 33 (1.028%)<br>• phylum: 28 (0.872%)<br>• superkingdom: 130 (4.051%)<br>• root: 258 (8.039%)	• Staphylococcus aureus [taxid 1280]: 27 (0.841%)<br>• Staphylococcus pasteuri [taxid 45972]: 4 (0.124%)<br>• Staphylococcus devriesei [taxid 586733]: 2 (0.062%)<br>• Staphylococcus pragensis [taxid 1611836]: 2 (0.062%)<br>• Staphylococcus auricularis [taxid 29379]: 2 (0.062%)<br>• Staphylococcus saccharolyticus [taxid 33028]: 2 (0.062%)<br>• Staphylococcus rostri [taxid 522262]: 2 (0.062%)<br>• Megasphaera cerevisiae [taxid 39029]: 1 (0.031%)<br>• other: 19 (0.592%)
Benchmark OTU ID: `CP002051-_Crenarchaeota`<br>OTU taxon: Staphylothermus hellenicus DSM 12710 [taxid 591019]<br>Expected: Staphylothermus hellenicus [taxid 84599] (species)<br>Number of reads: 3076<br>Number of identified reads: 3015 (98.016%)	• **species: 1761 (57.249%)**<br>• genus: 380 (12.353%)<br>• family: 29 (0.942%)<br>• order: 7 (0.227%)<br>• class: 54 (1.755%)<br>• phylum: 1 (0.032%)<br>• superkingdom: 40 (1.3%)<br>• root: 713 (23.179%)	• Staphylothermus marinus [taxid 2280]: 20 (0.65%)<br>• Thermoprotei archaeon [taxid 2250277]: 3 (0.097%)<br>• Dracunculus medinensis [taxid 318479]: 1 (0.032%)<br>• Plasmodium falciparum [taxid 5833]: 1 (0.032%)
Benchmark OTU ID: `CP000575-_Crenarchaeota`<br>OTU taxon: Staphylothermus marinus F1 [taxid 399550]<br>Expected: Staphylothermus marinus [taxid 2280] (species)<br>Number of reads: 3051<br>Number of identified reads: 2976 (97.541%)	• **species: 1792 (58.734%)**<br>• genus: 332 (10.881%)<br>• family: 14 (0.458%)<br>• order: 7 (0.229%)<br>• class: 86 (2.818%)<br>• phylum: 0 (0.0%)<br>• superkingdom: 38 (1.245%)<br>• root: 692 (22.681%)	• Staphylothermus hellenicus [taxid 84599]: 16 (0.524%)<br>• Thermoprotei archaeon [taxid 2250277]: 2 (0.065%)<br>• Desulfurococcus amylolyticus [taxid 94694]: 1 (0.032%)<br>• Aeropyrum pernix [taxid 56636]: 1 (0.032%)<br>• Candidatus Bathyarchaeota archaeon [taxid 2026714]: 1 (0.032%)<br>• Leptothrix cholodnii [taxid 34029]: 1 (0.032%)

Operational Taxonomic Unit (OTU)	Correct identifications	Wrong or overspecific identifications at species rank
Benchmark OTU ID: `CP002026-_Proteobacteria`<br>OTU taxon: Starkeya novella DSM 506 [taxid 639283]<br>Expected: Starkeya novella [taxid 921] (species)<br>Number of reads: 9837<br>Number of identified reads: 9795 (99.573%)	• **species: 5591 (56.836%)**<br>• genus: 293 (2.978%)<br>• family: 841 (8.549%)<br>• order: 781 (7.939%)<br>• class: 530 (5.387%)<br>• phylum: 409 (4.157%)<br>• superkingdom: 537 (5.458%)<br>• root: 812 (8.254%)	• Ancylobacter aquaticus [taxid 100]: 7 (0.071%)<br>• Ancylobacter pratisalsi [taxid 1745854]: 6 (0.06%)<br>• Pseudolabrys sp. GY_H [taxid 2292256]: 2 (0.02%)<br>• Bosea lathyri [taxid 1036778]: 2 (0.02%)<br>• Bradyrhizobium cytisi [taxid 515489]: 2 (0.02%)<br>• Rhodopseudomonas palustris [taxid 1076]: 1 (0.01%)<br>• Sphingomonas hengshuiensis [taxid 1609977]: 1 (0.01%)<br>• Rhodoferax saidenbachensis [taxid 1484693]: 1 (0.01%)<br>• other: 29 (0.294%)
Benchmark OTU ID: `HE798556-_Proteobacteria`<br>OTU taxon: Stenotrophomonas maltophilia D457 [taxid 1163399]<br>Expected: Stenotrophomonas maltophilia [taxid 40324] (species)<br>Number of reads: 9846<br>Number of identified reads: 9799 (99.522%)	• species: 494 (5.017%)<br>• **genus: 5552 (56.388%)**<br>• family: 1184 (12.025%)<br>• order: 96 (0.975%)<br>• class: 257 (2.61%)<br>• phylum: 686 (6.967%)<br>• superkingdom: 722 (7.332%)<br>• root: 797 (8.094%)	• Stenotrophomonas pavanii [taxid 487698]: 38 (0.385%)<br>• Brugia timori [taxid 42155]: 5 (0.05%)<br>• Stenotrophomonas rhizophila [taxid 216778]: 4 (0.04%)<br>• Xanthomonas citri [taxid 346]: 4 (0.04%)<br>• Pseudomonas aeruginosa [taxid 287]: 4 (0.04%)<br>• Stenotrophomonas koreensis [taxid 266128]: 3 (0.03%)<br>• Stenotrophomonas humi [taxid 405444]: 2 (0.02%)<br>• Xanthomonas populi [taxid 53414]: 2 (0.02%)<br>• other: 60 (0.609%)
Benchmark OTU ID: `AM743169-_Proteobacteria`<br>OTU taxon: Stenotrophomonas maltophilia K279a [taxid 522373]<br>Expected: Stenotrophomonas maltophilia [taxid 40324] (species)<br>Number of reads: 10031<br>Number of identified reads: 9995 (99.641%)	• species: 2020 (20.137%)<br>• **genus: 4659 (46.446%)**<br>• family: 1127 (11.235%)<br>• order: 89 (0.887%)<br>• class: 287 (2.861%)<br>• phylum: 546 (5.443%)<br>• superkingdom: 562 (5.602%)<br>• root: 689 (6.868%)	• Brugia timori [taxid 42155]: 5 (0.049%)<br>• Xanthomonas arboricola [taxid 56448]: 5 (0.049%)<br>• Xanthomonas axonopodis [taxid 53413]: 2 (0.019%)<br>• Salmonella enterica [taxid 28901]: 2 (0.019%)<br>• Stenotrophomonas rhizophila [taxid 216778]: 2 (0.019%)<br>• Beta vulgaris [taxid 161934]: 2 (0.019%)<br>• Stenotrophomonas koreensis [taxid 266128]: 2 (0.019%)<br>• Stenotrophomonas humi [taxid 405444]: 2 (0.019%)<br>• other: 37 (0.368%)
Benchmark OTU ID: `CP002271-_Proteobacteria`<br>OTU taxon: Stigmatella aurantiaca DW4/3-1 [taxid 378806]<br>Expected: Stigmatella aurantiaca [taxid 41] (species)<br>Number of reads: 22203<br>Number of identified reads: 21960 (98.905%)	• **species: 14333 (64.554%)**<br>• genus: 1637 (7.372%)<br>• family: 636 (2.864%)<br>• order: 1243 (5.598%)<br>• class: 52 (0.234%)<br>• phylum: 616 (2.774%)<br>• superkingdom: 1218 (5.485%)<br>• root: 2195 (9.886%)	• Stigmatella erecta [taxid 83460]: 10 (0.045%)<br>• Hyalangium minutum [taxid 394096]: 9 (0.04%)<br>• Cystobacter ferrugineus [taxid 83449]: 3 (0.013%)<br>• Myxococcus llanfairpwllgwyngyllgogerychwyrndrobwllllantysiliogogogochensis [taxid 2590453]: 2 (0.009%)<br>• Corallococcus llansteffanensis [taxid 2316731]: 2 (0.009%)<br>• Melittangium boletus [taxid 83453]: 2 (0.009%)<br>• Archangium gephyra [taxid 48]: 2 (0.009%)<br>• Cystobacter fuscus [taxid 43]: 2 (0.009%)<br>• other: 33 (0.148%)

Operational Taxonomic Unit (OTU)	Correct identifications	Wrong or overspecific identifications at species rank
Benchmark OTU ID: `CP001845-_Firmicutes`<br>OTU taxon: Streptococcus pneumoniae gamPNI0373 [taxid 697283]<br>Expected: Streptococcus pneumoniae [taxid 1313] (species)<br>Number of reads: 2534<br>Number of identified reads: 2501 (98.697%)	• species: 728 (28.729%)<br>• **genus: 1406 (55.485%)**<br>• family: 14 (0.552%)<br>• order: 31 (1.223%)<br>• class: 20 (0.789%)<br>• phylum: 30 (1.183%)<br>• superkingdom: 84 (3.314%)<br>• root: 186 (7.34%)	• Streptococcus mitis [taxid 28037]: 24 (0.947%)<br>• Streptococcus oralis [taxid 1303]: 13 (0.513%)<br>• Streptococcus pseudopneumoniae [taxid 257758]: 5 (0.197%)<br>• Streptococcus infantis [taxid 68892]: 3 (0.118%)<br>• Streptococcus merionis [taxid 400065]: 2 (0.078%)<br>• Streptococcus downei [taxid 1317]: 1 (0.039%)<br>• Streptococcus constellatus [taxid 76860]: 1 (0.039%)<br>• Streptococcus chenjunshii [taxid 2173853]: 1 (0.039%)<br>• other: 10 (0.394%)
Benchmark OTU ID: `AE004092-_Firmicutes`<br>OTU taxon: Streptococcus pyogenes M1 GAS [taxid 160490]<br>Expected: Streptococcus pyogenes [taxid 1314] (species)<br>Number of reads: 2169<br>Number of identified reads: 2144 (98.847%)	• species: 825 (38.035%)<br>• **genus: 827 (38.128%)**<br>• family: 14 (0.645%)<br>• order: 46 (2.12%)<br>• class: 26 (1.198%)<br>• phylum: 17 (0.783%)<br>• superkingdom: 76 (3.503%)<br>• root: 309 (14.246%)	• Streptococcus dysgalactiae [taxid 1334]: 8 (0.368%)<br>• Streptococcus suis [taxid 1307]: 3 (0.138%)<br>• Paenibacillus polymyxa [taxid 1406]: 2 (0.092%)<br>• Streptococcus parauberis [taxid 1348]: 2 (0.092%)<br>• Streptococcus sanguinis [taxid 1305]: 1 (0.046%)<br>• Streptococcus equinus [taxid 1335]: 1 (0.046%)<br>• Streptococcus castoreus [taxid 254786]: 1 (0.046%)<br>• Streptococcus ictaluri [taxid 380397]: 1 (0.046%)<br>• other: 22 (1.014%)
Benchmark OTU ID: `CP003219-_Actinobacteria`<br>OTU taxon: Streptomyces cattleya NRRL 8057 = DSM 46488 [taxid 1003195]<br>Expected: Streptomyces cattleya [taxid 29303] (species)<br>Number of reads: 28383<br>Number of identified reads: 28201 (99.358%)	• **species: 15979 (56.297%)**<br>• genus: 5813 (20.48%)<br>• family: 169 (0.595%)<br>• order: 0 (0.0%)<br>• class: 1974 (6.954%)<br>• phylum: 18 (0.063%)<br>• superkingdom: 1646 (5.799%)<br>• root: 2563 (9.03%)	• Streptomyces angustmyceticus [taxid 285578]: 6 (0.021%)<br>• Streptomyces albus [taxid 1888]: 6 (0.021%)<br>• Beta vulgaris [taxid 161934]: 5 (0.017%)<br>• Streptomyces violaceusniger [taxid 68280]: 5 (0.017%)<br>• Streptomyces viridosporus [taxid 67581]: 4 (0.014%)<br>• Streptomyces sp. C [taxid 253839]: 4 (0.014%)<br>• Streptomyces rapamycinicus [taxid 1226757]: 4 (0.014%)<br>• Streptomyces avermitilis [taxid 33903]: 3 (0.01%)<br>• other: 186 (0.655%)
Benchmark OTU ID: `ENA|AL645882|AL645882.2-_Actinobacteria`<br>OTU taxon: Streptomyces coelicolor [taxid 1902]<br>Expected: Streptomyces coelicolor [taxid 1902] (species)<br>Number of reads: 40068<br>Number of identified reads: 39875 (99.518%)	• species: 1509 (3.766%)<br>• **genus: 31891 (79.592%)**<br>• family: 46 (0.114%)<br>• order: 0 (0.0%)<br>• class: 1880 (4.692%)<br>• phylum: 27 (0.067%)<br>• superkingdom: 1727 (4.31%)<br>• root: 2753 (6.87%)	• Streptomyces ambofaciens [taxid 1889]: 32 (0.079%)<br>• Streptomyces rubrogriseus [taxid 194673]: 22 (0.054%)<br>• Streptomyces coelicoflavus [taxid 285562]: 21 (0.052%)<br>• Streptomyces parvulus [taxid 146923]: 13 (0.032%)<br>• Streptomyces pactum [taxid 68249]: 12 (0.029%)<br>• Streptomyces lividans [taxid 1916]: 11 (0.027%)<br>• Streptomyces viridosporus [taxid 67581]: 9 (0.022%)<br>• Streptomyces avermitilis [taxid 33903]: 8 (0.019%)<br>• other: 559 (1.395%)

Operational Taxonomic Unit (OTU)	Correct identifications	Wrong or overspecific identifications at species rank
Benchmark OTU ID: `HE971709-_Actinobacteria`<br>OTU taxon: Streptomyces davaonensis JCM 4913 [taxid 1214101]<br>Expected: Streptomyces davaonensis [taxid 348043] (species)<br>Number of reads: 43973<br>Number of identified reads: 43791 (99.586%)	• species: 18004 (40.943%)<br>• **genus: 18208 (41.407%)**<br>• family: 90 (0.204%)<br>• order: 0 (0.0%)<br>• class: 2112 (4.802%)<br>• phylum: 25 (0.056%)<br>• superkingdom: 2010 (4.57%)<br>• root: 3302 (7.509%)	• Streptomyces ipomoeae [taxid 103232]: 13 (0.029%)<br>• Streptomyces viridochromogenes [taxid 1938]: 13 (0.029%)<br>• Streptomyces scabiei [taxid 1930]: 11 (0.025%)<br>• Streptomyces venezuelae [taxid 54571]: 9 (0.02%)<br>• Streptomyces shenzhenensis [taxid 943815]: 8 (0.018%)<br>• Streptomyces spinoverrucosus [taxid 284043]: 8 (0.018%)<br>• Streptomyces clavuligerus [taxid 1901]: 7 (0.015%)<br>• Streptomyces afghaniensis [taxid 66865]: 7 (0.015%)<br>• other: 427 (0.971%)
Benchmark OTU ID: `CP003720-_Actinobacteria`<br>OTU taxon: Streptomyces hygroscopicus subsp. jinggangensis TL01 [taxid 1203460]<br>Expected: Streptomyces hygroscopicus [taxid 1912] (species)<br>Number of reads: 45813<br>Number of identified reads: 45541 (99.406%)	• species: 4352 (9.499%)<br>• **genus: 32591 (71.139%)**<br>• family: 97 (0.211%)<br>• order: 0 (0.0%)<br>• class: 2392 (5.221%)<br>• phylum: 26 (0.056%)<br>• superkingdom: 2235 (4.878%)<br>• root: 3803 (8.301%)	• Streptomyces corchorusii [taxid 1903]: 125 (0.272%)<br>• Streptomyces pluripotens [taxid 1355015]: 16 (0.034%)<br>• Streptomyces misionensis [taxid 67331]: 11 (0.024%)<br>• Streptomyces roseochromogenus [taxid 285450]: 11 (0.024%)<br>• Streptomyces mangrovisoli [taxid 1428628]: 10 (0.021%)<br>• Streptomyces yokosukanensis [taxid 67386]: 10 (0.021%)<br>• Streptomyces fodineus [taxid 1904616]: 9 (0.019%)<br>• Streptomyces puniciscabiei [taxid 164348]: 9 (0.019%)<br>• other: 616 (1.344%)
Benchmark OTU ID: `CP003990-_Actinobacteria`<br>OTU taxon: Streptomyces sp. PAMC 26508 [taxid 1265601]<br>Expected: Streptomyces [taxid 1883] (genus)<br>Number of reads: 34475<br>Number of identified reads: 34288 (99.457%)	• **genus: 22952 (66.575%)**<br>• family: 106 (0.307%)<br>• order: 0 (0.0%)<br>• class: 2875 (8.339%)<br>• phylum: 16 (0.046%)<br>• superkingdom: 5426 (15.738%)<br>• root: 2871 (8.327%)	• Streptomyces pratensis [taxid 1169025]: 34 (0.098%)<br>• Streptomyces argenteolus [taxid 67274]: 26 (0.075%)<br>• Streptomyces avidinii [taxid 1895]: 24 (0.069%)<br>• Streptomyces sp. SirexAA-E [taxid 862751]: 23 (0.066%)<br>• Streptomyces nitrosporeus [taxid 28894]: 13 (0.037%)<br>• Streptomyces filamentosus [taxid 67294]: 11 (0.031%)<br>• Streptomyces lunaelactis [taxid 1535768]: 10 (0.029%)<br>• other: 459 (1.331%)
Benchmark OTU ID: `CP002993-_Actinobacteria`<br>OTU taxon: Streptomyces sp. SirexAA-E [taxid 862751]<br>Expected: Streptomyces sp. SirexAA-E [taxid 862751] (species)<br>Number of reads: 33928<br>Number of identified reads: 33741 (99.448%)	• **species: 14447 (42.581%)**<br>• genus: 13018 (38.369%)<br>• family: 61 (0.179%)<br>• order: 0 (0.0%)<br>• class: 1738 (5.122%)<br>• phylum: 20 (0.058%)<br>• superkingdom: 1722 (5.075%)<br>• root: 2701 (7.96%)	• Streptomyces nitrosporeus [taxid 28894]: 11 (0.032%)<br>• Streptomyces argenteolus [taxid 67274]: 9 (0.026%)<br>• Streptomyces cyaneofuscatus [taxid 66883]: 7 (0.02%)<br>• Streptomyces venezuelae [taxid 54571]: 7 (0.02%)<br>• Streptomyces phyllanthi [taxid 1803180]: 7 (0.02%)<br>• Globodera pallida [taxid 36090]: 6 (0.017%)<br>• Streptomyces vitaminophilus [taxid 76728]: 5 (0.014%)<br>• Streptomyces sp. C [taxid 253839]: 5 (0.014%)<br>• other: 277 (0.816%)

Operational Taxonomic Unit (OTU)	Correct identifications	Wrong or overspecific identifications at species rank
Benchmark OTU ID: `CP002994-_Actinobacteria`<br>OTU taxon: Streptomyces violaceusniger Tu 4113 [taxid 653045]<br>Expected: Streptomyces violaceusniger [taxid 68280] (species)<br>Number of reads: 49817<br>Number of identified reads: 49517 (99.397%)	• species: 10814 (21.707%)<br>• **genus: 28532 (57.273%)**<br>• family: 111 (0.222%)<br>• order: 0 (0.0%)<br>• class: 3203 (6.429%)<br>• phylum: 26 (0.052%)<br>• superkingdom: 2424 (4.865%)<br>• root: 4355 (8.741%)	• Streptomyces antimycoticus [taxid 68175]: 79 (0.158%)<br>• Streptomyces melanosporofaciens [taxid 67327]: 44 (0.088%)<br>• Streptomyces hygroscopicus [taxid 1912]: 37 (0.074%)<br>• Streptomyces rapamycinicus [taxid 1226757]: 31 (0.062%)<br>• Streptomyces himastatinicus [taxid 998084]: 29 (0.058%)<br>• Streptomyces iranensis [taxid 576784]: 29 (0.058%)<br>• Streptomyces malaysiensis [taxid 92644]: 29 (0.058%)<br>• Streptomyces antioxidans [taxid 1507734]: 26 (0.052%)<br>• other: 475 (0.953%)
Benchmark OTU ID: `CP000077-_Crenarchaeota`<br>OTU taxon: Sulfolobus acidocaldarius DSM 639 [taxid 330779]<br>Expected: Sulfolobus acidocaldarius [taxid 2285] (species)<br>Number of reads: 4690<br>Number of identified reads: 4576 (97.569%)	• **species: 3245 (69.189%)**<br>• genus: 21 (0.447%)<br>• family: 144 (3.07%)<br>• order: 5 (0.106%)<br>• class: 24 (0.511%)<br>• phylum: 1 (0.021%)<br>• superkingdom: 32 (0.682%)<br>• root: 1068 (22.771%)	• Bizionia saleffrena [taxid 291189]: 1 (0.021%)<br>• bacterium [taxid 1869227]: 1 (0.021%)<br>• Sulfurisphaera tokodaii [taxid 111955]: 1 (0.021%)<br>• Candidatus Bathyarchaeota archaeon [taxid 2026714]: 1 (0.021%)<br>• Stygiolobus azoricus [taxid 41675]: 1 (0.021%)<br>• Metallosphaera cuprina [taxid 1006005]: 1 (0.021%)<br>• Bursaphelenchus xylophilus [taxid 6326]: 1 (0.021%)
Benchmark OTU ID: `CP002817-_Crenarchaeota`<br>OTU taxon: Sulfolobus acidocaldarius N8 [taxid 1028566]<br>Expected: Sulfolobus acidocaldarius [taxid 2285] (species)<br>Number of reads: 4566<br>Number of identified reads: 4446 (97.371%)	• **species: 3233 (70.805%)**<br>• genus: 14 (0.306%)<br>• family: 128 (2.803%)<br>• order: 2 (0.043%)<br>• class: 16 (0.35%)<br>• phylum: 0 (0.0%)<br>• superkingdom: 26 (0.569%)<br>• root: 998 (21.857%)	• Oesophagostomum dentatum [taxid 61180]: 1 (0.021%)<br>• Bursaphelenchus xylophilus [taxid 6326]: 1 (0.021%)<br>• Pteridomonas danica [taxid 38822]: 1 (0.021%)<br>• Naegleria gruberi [taxid 5762]: 1 (0.021%)<br>• Acidianus infernus [taxid 12915]: 1 (0.021%)<br>• Pseudoalteromonas luteoviolacea [taxid 43657]: 1 (0.021%)<br>• Portunus trituberculatus [taxid 210409]: 1 (0.021%)<br>• Trema orientale [taxid 63057]: 1 (0.021%)
Benchmark OTU ID: `CP002818-_Crenarchaeota`<br>OTU taxon: Sulfolobus acidocaldarius Ron12/I [taxid 1028567]<br>Expected: Sulfolobus acidocaldarius [taxid 2285] (species)<br>Number of reads: 4685<br>Number of identified reads: 4552 (97.161%)	• **species: 3303 (70.501%)**<br>• genus: 21 (0.448%)<br>• family: 139 (2.966%)<br>• order: 4 (0.085%)<br>• class: 19 (0.405%)<br>• phylum: 0 (0.0%)<br>• superkingdom: 23 (0.49%)<br>• root: 1016 (21.686%)	• Metallosphaera hakonensis [taxid 79601]: 1 (0.021%)<br>• Clostridium gasigenes [taxid 94869]: 1 (0.021%)<br>• Thermoprotei archaeon [taxid 2250277]: 1 (0.021%)<br>• Candidatus Mancarchaeum acidiphilum [taxid 1920749]: 1 (0.021%)<br>• Metallosphaera tengchongensis [taxid 1532350]: 1 (0.021%)<br>• Sulfolobus spindle-shaped virus [taxid 2491899]: 1 (0.021%)<br>• Amorphotheca resinae [taxid 5101]: 1 (0.021%)<br>• Caldivirga maquilingensis [taxid 76887]: 1 (0.021%)
Benchmark OTU ID: `CP002426-_Crenarchaeota`<br>OTU taxon: Sulfolobus islandicus HVE10/4 [taxid 930943]<br>Expected: Sulfolobus islandicus [taxid 43080] (species)<br>Number of reads: 5761<br>Number of identified reads: 5602 (97.24%)	• **species: 2626 (45.582%)**<br>• genus: 364 (6.318%)<br>• family: 1333 (23.138%)<br>• order: 8 (0.138%)<br>• class: 34 (0.59%)<br>• phylum: 0 (0.0%)<br>• superkingdom: 40 (0.694%)<br>• root: 1167 (20.256%)	• Saccharolobus solfataricus [taxid 2287]: 17 (0.295%)<br>• Sulfuracidifex metallicus [taxid 47303]: 2 (0.034%)<br>• Metallosphaera yellowstonensis [taxid 1111107]: 2 (0.034%)<br>• Acidianus sulfidivorans [taxid 312539]: 2 (0.034%)<br>• Acidianus hospitalis [taxid 563177]: 2 (0.034%)<br>• Sulfuracidifex tepidarius [taxid 1294262]: 2 (0.034%)<br>• Stygiolobus azoricus [taxid 41675]: 1 (0.017%)<br>• Candidatus Acidianus copahuensis [taxid 1160895]: 1 (0.017%)<br>• other: 12 (0.208%)

Operational Taxonomic Unit (OTU)	Correct identifications	Wrong or overspecific identifications at species rank
Benchmark OTU ID: `CP001731-_Crenarchaeota`<br>OTU taxon: Sulfolobus islandicus L.D.8.5 [taxid 425944]<br>Expected: Sulfolobus islandicus [taxid 43080] (species)<br>Number of reads: 5929<br>Number of identified reads: 5741 (96.829%)	• **species: 2580 (43.514%)**<br>• genus: 357 (6.021%)<br>• family: 1442 (24.321%)<br>• order: 23 (0.387%)<br>• class: 26 (0.438%)<br>• phylum: 0 (0.0%)<br>• superkingdom: 35 (0.59%)<br>• root: 1238 (20.88%)	• Saccharolobus solfataricus [taxid 2287]: 29 (0.489%)<br>• Metallosphaera yellowstonensis [taxid 1111107]: 4 (0.067%)<br>• Sulfolobus acidocaldarius [taxid 2285]: 4 (0.067%)<br>• Sulfuracidifex tepidarius [taxid 1294262]: 3 (0.05%)<br>• Acidianus hospitalis [taxid 563177]: 2 (0.033%)<br>• Sulfuracidifex metallicus [taxid 47303]: 2 (0.033%)<br>• Sulfodiicoccus acidiphilus [taxid 1670455]: 2 (0.033%)<br>• Acidianus brierleyi [taxid 41673]: 1 (0.016%)<br>• other: 9 (0.151%)
Benchmark OTU ID: `CP001399-_Crenarchaeota`<br>OTU taxon: Sulfolobus islandicus L.S.2.15 [taxid 429572]<br>Expected: Sulfolobus islandicus [taxid 43080] (species)<br>Number of reads: 5966<br>Number of identified reads: 5791 (97.066%)	• **species: 2614 (43.814%)**<br>• genus: 388 (6.503%)<br>• family: 1414 (23.7%)<br>• order: 13 (0.217%)<br>• class: 28 (0.469%)<br>• phylum: 0 (0.0%)<br>• superkingdom: 31 (0.519%)<br>• root: 1257 (21.069%)	• Saccharolobus solfataricus [taxid 2287]: 21 (0.351%)<br>• Metallosphaera yellowstonensis [taxid 1111107]: 5 (0.083%)<br>• Acidianus brierleyi [taxid 41673]: 3 (0.05%)<br>• Sulfolobus acidocaldarius [taxid 2285]: 3 (0.05%)<br>• Acidianus manzaensis [taxid 282676]: 2 (0.033%)<br>• Metallosphaera cuprina [taxid 1006005]: 2 (0.033%)<br>• Saccharolobus shibatae [taxid 2286]: 2 (0.033%)<br>• Sulfolobus spindle-shaped virus Lassen [taxid 1959008]: 2 (0.033%)<br>• other: 11 (0.184%)
Benchmark OTU ID: `CP003928-_Crenarchaeota`<br>OTU taxon: Sulfolobus islandicus LAL14/1 [taxid 1241935]<br>Expected: Sulfolobus islandicus [taxid 43080] (species)<br>Number of reads: 5288<br>Number of identified reads: 5166 (97.692%)	• **species: 2354 (44.515%)**<br>• genus: 332 (6.278%)<br>• family: 1275 (24.111%)<br>• order: 15 (0.283%)<br>• class: 28 (0.529%)<br>• phylum: 0 (0.0%)<br>• superkingdom: 41 (0.775%)<br>• root: 1082 (20.461%)	• Saccharolobus solfataricus [taxid 2287]: 25 (0.472%)<br>• Sulfuracidifex metallicus [taxid 47303]: 4 (0.075%)<br>• Acidianus hospitalis [taxid 563177]: 3 (0.056%)<br>• Metallosphaera hakonensis [taxid 79601]: 2 (0.037%)<br>• Acidianus ambivalens [taxid 2283]: 2 (0.037%)<br>• Penicilliopsis zonata [taxid 41063]: 1 (0.018%)<br>• Celerinatantimonas diazotrophica [taxid 412034]: 1 (0.018%)<br>• Sulfolobus acidocaldarius [taxid 2285]: 1 (0.018%)<br>• other: 9 (0.17%)
Benchmark OTU ID: `CP001400-_Crenarchaeota`<br>OTU taxon: Sulfolobus islandicus M.14.25 [taxid 427317]<br>Expected: Sulfolobus islandicus [taxid 43080] (species)<br>Number of reads: 5647<br>Number of identified reads: 5494 (97.29%)	• **species: 2458 (43.527%)**<br>• genus: 395 (6.994%)<br>• family: 1309 (23.18%)<br>• order: 6 (0.106%)<br>• class: 33 (0.584%)<br>• phylum: 0 (0.0%)<br>• superkingdom: 39 (0.69%)<br>• root: 1212 (21.462%)	• Saccharolobus solfataricus [taxid 2287]: 11 (0.194%)<br>• Sulfolobus spindle-shaped virus [taxid 2491899]: 5 (0.088%)<br>• Sulfuracidifex metallicus [taxid 47303]: 2 (0.035%)<br>• Acidianus manzaensis [taxid 282676]: 2 (0.035%)<br>• Helicobacter canis [taxid 29419]: 1 (0.017%)<br>• Candidatus Verstraetearchaeota archaeon [taxid 2250257]: 1 (0.017%)<br>• Acidianus sulfidivorans [taxid 312539]: 1 (0.017%)<br>• Saccharolobus shibatae [taxid 2286]: 1 (0.017%)<br>• other: 11 (0.194%)

Operational Taxonomic Unit (OTU)	Correct identifications	Wrong or overspecific identifications at species rank
Benchmark OTU ID: `CP001401-_Crenarchaeota`<br>OTU taxon: Sulfolobus islandicus M.16.27 [taxid 427318]<br>Expected: Sulfolobus islandicus [taxid 43080] (species)<br>Number of reads: 5856<br>Number of identified reads: 5700 (97.336%)	• **species: 2611 (44.586%)**<br>• genus: 411 (7.018%)<br>• family: 1319 (22.523%)<br>• order: 11 (0.187%)<br>• class: 29 (0.495%)<br>• phylum: 1 (0.017%)<br>• superkingdom: 25 (0.426%)<br>• root: 1247 (21.294%)	• Saccharolobus solfataricus [taxid 2287]: 27 (0.461%)<br>• Metallosphaera yellowstonensis [taxid 1111107]: 2 (0.034%)<br>• Desulfovibrio gracilis [taxid 47158]: 1 (0.017%)<br>• Metallosphaera tengchongensis [taxid 1532350]: 1 (0.017%)<br>• Octopus bimaculoides [taxid 37653]: 1 (0.017%)<br>• Crustomastix stigmatica [taxid 195967]: 1 (0.017%)<br>• Metallosphaera hakonensis [taxid 79601]: 1 (0.017%)<br>• Acidianus sulfidivorans [taxid 312539]: 1 (0.017%)<br>• other: 9 (0.153%)
Benchmark OTU ID: `CP001402-_Crenarchaeota`<br>OTU taxon: Sulfolobus islandicus M.16.4 [taxid 426118]<br>Expected: Sulfolobus islandicus [taxid 43080] (species)<br>Number of reads: 5592<br>Number of identified reads: 5433 (97.156%)	• **species: 2501 (44.724%)**<br>• genus: 375 (6.706%)<br>• family: 1324 (23.676%)<br>• order: 7 (0.125%)<br>• class: 19 (0.339%)<br>• phylum: 0 (0.0%)<br>• superkingdom: 35 (0.625%)<br>• root: 1137 (20.332%)	• Saccharolobus solfataricus [taxid 2287]: 16 (0.286%)<br>• Sulfolobus acidocaldarius [taxid 2285]: 5 (0.089%)<br>• Acidianus sulfidivorans [taxid 312539]: 2 (0.035%)<br>• Sulfodiicoccus acidiphilus [taxid 1670455]: 2 (0.035%)<br>• Sulfuracidifex metallicus [taxid 47303]: 2 (0.035%)<br>• Alkalihalobacillus macyae [taxid 157733]: 1 (0.017%)<br>• Acidianus manzaensis [taxid 282676]: 1 (0.017%)<br>• Companilactobacillus nodensis [taxid 460870]: 1 (0.017%)<br>• other: 3 (0.053%)
Benchmark OTU ID: `CP002425-_Crenarchaeota`<br>OTU taxon: Sulfolobus islandicus REY15A [taxid 930945]<br>Expected: Sulfolobus islandicus [taxid 43080] (species)<br>Number of reads: 5432<br>Number of identified reads: 5276 (97.128%)	• **species: 2359 (43.427%)**<br>• genus: 374 (6.885%)<br>• family: 1306 (24.042%)<br>• order: 7 (0.128%)<br>• class: 22 (0.405%)<br>• phylum: 0 (0.0%)<br>• superkingdom: 34 (0.625%)<br>• root: 1133 (20.857%)	• Saccharolobus solfataricus [taxid 2287]: 23 (0.423%)<br>• Sulfolobus acidocaldarius [taxid 2285]: 6 (0.11%)<br>• Metallosphaera tengchongensis [taxid 1532350]: 2 (0.036%)<br>• Metallosphaera cuprina [taxid 1006005]: 2 (0.036%)<br>• Acidianus hospitalis [taxid 563177]: 2 (0.036%)<br>• Candidatus Acidianus copahuensis [taxid 1160895]: 1 (0.018%)<br>• Pseudoloma neurophilia [taxid 146866]: 1 (0.018%)<br>• Sulfodiicoccus acidiphilus [taxid 1670455]: 1 (0.018%)<br>• other: 12 (0.22%)
Benchmark OTU ID: `CP001403-_Crenarchaeota`<br>OTU taxon: Sulfolobus islandicus Y.G.57.14 [taxid 439386]<br>Expected: Sulfolobus islandicus [taxid 43080] (species)<br>Number of reads: 5880<br>Number of identified reads: 5715 (97.193%)	• **species: 2466 (41.938%)**<br>• genus: 380 (6.462%)<br>• family: 1527 (25.969%)<br>• order: 12 (0.204%)<br>• class: 24 (0.408%)<br>• phylum: 0 (0.0%)<br>• superkingdom: 39 (0.663%)<br>• root: 1234 (20.986%)	• Saccharolobus solfataricus [taxid 2287]: 26 (0.442%)<br>• Metallosphaera yellowstonensis [taxid 1111107]: 5 (0.085%)<br>• Acidianus manzaensis [taxid 282676]: 2 (0.034%)<br>• Acidianus hospitalis [taxid 563177]: 2 (0.034%)<br>• Saccharolobus shibatae [taxid 2286]: 2 (0.034%)<br>• Acidianus ambivalens [taxid 2283]: 1 (0.017%)<br>• Sulfurisphaera tokodaii [taxid 111955]: 1 (0.017%)<br>• Sulfurisphaera ohwakuensis [taxid 69656]: 1 (0.017%)<br>• other: 12 (0.204%)

Operational Taxonomic Unit (OTU)	Correct identifications	Wrong or overspecific identifications at species rank
Benchmark OTU ID: `CP001404-_Crenarchaeota`<br>OTU taxon: Sulfolobus islandicus Y.N.15.51 [taxid 419942]<br>Expected: Sulfolobus islandicus [taxid 43080] (species)<br>Number of reads: 6155<br>Number of identified reads: 5980 (97.156%)	• **species: 2619 (42.55%)**<br>• genus: 387 (6.287%)<br>• family: 1556 (25.28%)<br>• order: 14 (0.227%)<br>• class: 35 (0.568%)<br>• phylum: 0 (0.0%)<br>• superkingdom: 47 (0.763%)<br>• root: 1278 (20.763%)	• Saccharolobus solfataricus [taxid 2287]: 29 (0.471%)<br>• Metallosphaera yellowstonensis [taxid 1111107]: 18 (0.292%)<br>• Acidianus hospitalis [taxid 563177]: 5 (0.081%)<br>• Sulfolobus acidocaldarius [taxid 2285]: 3 (0.048%)<br>• Acidianus manzaensis [taxid 282676]: 2 (0.032%)<br>• Sulfurisphaera tokodaii [taxid 111955]: 2 (0.032%)<br>• Acidianus brierleyi [taxid 41673]: 2 (0.032%)<br>• Sulfuracidifex metallicus [taxid 47303]: 2 (0.032%)<br>• other: 12 (0.194%)
Benchmark OTU ID: `CP002205-_Proteobacteria`<br>OTU taxon: Sulfurimonas autotrophica DSM 16294 [taxid 563040]<br>Expected: Sulfurimonas autotrophica [taxid 202747] (species)<br>Number of reads: 3960<br>Number of identified reads: 3936 (99.393%)	• **species: 2819 (71.186%)**<br>• genus: 80 (2.02%)<br>• family: 6 (0.151%)<br>• order: 187 (4.722%)<br>• class: 53 (1.338%)<br>• phylum: 119 (3.005%)<br>• superkingdom: 243 (6.136%)<br>• root: 423 (10.681%)	• Helicobacter pylori [taxid 210]: 8 (0.202%)<br>• Helicobacter sanguini [taxid 1548018]: 1 (0.025%)<br>• Campylobacter jejuni [taxid 197]: 1 (0.025%)<br>• Candidatus Liberibacter asiaticus [taxid 34021]: 1 (0.025%)<br>• Planococcus donghaensis [taxid 414778]: 1 (0.025%)<br>• Campylobacter lari [taxid 201]: 1 (0.025%)<br>• Cyberlindnera fabianii [taxid 36022]: 1 (0.025%)<br>• Desulfurobacterium atlanticum [taxid 240169]: 1 (0.025%)<br>• other: 8 (0.202%)
Benchmark OTU ID: `CP000153-_Proteobacteria`<br>OTU taxon: Sulfurimonas denitrificans DSM 1251 [taxid 326298]<br>Expected: Sulfurimonas denitrificans [taxid 39766] (species)<br>Number of reads: 4069<br>Number of identified reads: 4025 (98.918%)	• **species: 2778 (68.272%)**<br>• genus: 296 (7.274%)<br>• family: 7 (0.172%)<br>• order: 241 (5.922%)<br>• class: 30 (0.737%)<br>• phylum: 97 (2.383%)<br>• superkingdom: 180 (4.423%)<br>• root: 392 (9.633%)	• Helicobacter pylori [taxid 210]: 4 (0.098%)<br>• Sulfurimonas crateris [taxid 2574727]: 2 (0.049%)<br>• Sulfurimonas hongkongensis [taxid 1172190]: 2 (0.049%)<br>• Campylobacter concisus [taxid 199]: 2 (0.049%)<br>• Diaphanoeca grandis [taxid 28014]: 1 (0.024%)<br>• Corethrella appendiculata [taxid 1370023]: 1 (0.024%)<br>• Thiovulum sp. ES [taxid 1177931]: 1 (0.024%)
Benchmark OTU ID: `BA000023-_Crenarchaeota`<br>OTU taxon: Sulfurisphaera tokodaii str. 7 [taxid 273063]<br>Expected: Sulfurisphaera tokodaii [taxid 111955] (species)<br>Number of reads: 5862<br>Number of identified reads: 5674 (96.792%)	• **species: 1751 (29.87%)**<br>• genus: 1139 (19.43%)<br>• family: 1394 (23.78%)<br>• order: 11 (0.187%)<br>• class: 31 (0.528%)<br>• phylum: 0 (0.0%)<br>• superkingdom: 31 (0.528%)<br>• root: 1284 (21.903%)	• Sulfurisphaera ohwakuensis [taxid 69656]: 24 (0.409%)<br>• Sulfolobus acidocaldarius [taxid 2285]: 5 (0.085%)<br>• Sulfolobus islandicus [taxid 43080]: 5 (0.085%)<br>• Acidianus brierleyi [taxid 41673]: 2 (0.034%)<br>• Saccharolobus solfataricus [taxid 2287]: 2 (0.034%)<br>• Metallosphaera yellowstonensis [taxid 1111107]: 2 (0.034%)<br>• Acidianus manzaensis [taxid 282676]: 1 (0.017%)<br>• Acidianus ambivalens [taxid 2283]: 1 (0.017%)<br>• other: 8 (0.136%)

Operational Taxonomic Unit (OTU)	Correct identifications	Wrong or overspecific identifications at species rank
Benchmark OTU ID: `CP003333-_Proteobacteria`<br>OTU taxon: Sulfurospirillum barnesii SES-3 [taxid 760154]<br>Expected: Sulfurospirillum barnesii [taxid 44674] (species)<br>Number of reads: 4764<br>Number of identified reads: 4746 (99.622%)	• **species: 3138 (65.869%)**<br>• genus: 652 (13.685%)<br>• family: 161 (3.379%)<br>• order: 138 (2.896%)<br>• class: 9 (0.188%)<br>• phylum: 132 (2.77%)<br>• superkingdom: 176 (3.694%)<br>• root: 338 (7.094%)	• Sulfurospirillum deleyianum [taxid 65553]: 5 (0.104%)<br>• Sulfurospirillum halorespirans [taxid 194424]: 4 (0.083%)<br>• Helicobacter pylori [taxid 210]: 2 (0.041%)<br>• Sulfurospirillum sp. MES [taxid 1565314]: 2 (0.041%)<br>• Helicobacter ailurogastricus [taxid 1578720]: 1 (0.02%)<br>• Nitrincola tapanii [taxid 1708751]: 1 (0.02%)<br>• Campylobacter jejuni [taxid 197]: 1 (0.02%)<br>• Nautilia profundicola [taxid 244787]: 1 (0.02%)
Benchmark OTU ID: `AP006840-_Firmicutes`<br>OTU taxon: Symbiobacterium thermophilum IAM 14863 [taxid 292459]<br>Expected: Symbiobacterium thermophilum [taxid 2734] (species)<br>Number of reads: 4937<br>Number of identified reads: 4887 (98.987%)	• **species: 3242 (65.667%)**<br>• genus: 2 (0.04%)<br>• family: 0 (0.0%)<br>• order: 19 (0.384%)<br>• class: 9 (0.182%)<br>• phylum: 592 (11.991%)<br>• superkingdom: 548 (11.099%)<br>• root: 470 (9.519%)	• Streptococcus suis [taxid 1307]: 1 (0.02%)<br>• Paenibacillus typhae [taxid 1174501]: 1 (0.02%)<br>• Methylocapsa palsarum [taxid 1612308]: 1 (0.02%)<br>• Clostridium intestinale [taxid 36845]: 1 (0.02%)<br>• Isosphaera pallida [taxid 128]: 1 (0.02%)<br>• Arthrobacter subterraneus [taxid 335973]: 1 (0.02%)<br>• Helobdella robusta [taxid 6412]: 1 (0.02%)<br>• Lacrimispora algidixylanolytica [taxid 94868]: 1 (0.02%)<br>• other: 5 (0.101%)
Benchmark OTU ID: `AP008231-_Cyanobacteria`<br>OTU taxon: Synechococcus elongatus PCC 6301 [taxid 269084]<br>Expected: Synechococcus elongatus [taxid 32046] (species)<br>Number of reads: 13935<br>Number of identified reads: 13866 (99.504%)	• **species: 9401 (67.463%)**<br>• genus: 130 (0.932%)<br>• family: 23 (0.165%)<br>• order: 151 (1.083%)<br>• phylum: 781 (5.604%)<br>• superkingdom: 1156 (8.295%)<br>• root: 2175 (15.608%)	• Solanum chacoense [taxid 4108]: 3 (0.021%)<br>• Sphaerochaeta pleomorpha [taxid 1131707]: 2 (0.014%)<br>• Heliobacterium modesticaldum [taxid 35701]: 2 (0.014%)<br>• Rosa chinensis [taxid 74649]: 1 (0.007%)<br>• Ferrimicrobium acidiphilum [taxid 121039]: 1 (0.007%)<br>• cyanobacterium endosymbiont of Epithemia turgida [taxid 718217]: 1 (0.007%)<br>• Branchiostoma floridae [taxid 7739]: 1 (0.007%)<br>• other: 29 (0.208%)
Benchmark OTU ID: `CP000100-_Cyanobacteria`<br>OTU taxon: Synechococcus elongatus PCC 7942 = FACHB-805 [taxid 1140]<br>Expected: Synechococcus elongatus [taxid 32046] (species)<br>Number of reads: 13933<br>Number of identified reads: 13869 (99.54%)	• **species: 9384 (67.35%)**<br>• genus: 144 (1.033%)<br>• family: 20 (0.143%)<br>• order: 148 (1.062%)<br>• phylum: 809 (5.806%)<br>• superkingdom: 1230 (8.827%)<br>• root: 2096 (15.043%)	• Solanum chacoense [taxid 4108]: 2 (0.014%)<br>• Stenomitos frigidus [taxid 1886765]: 2 (0.014%)<br>• Porphyra purpurea [taxid 2787]: 1 (0.007%)<br>• Capsicum annuum [taxid 4072]: 1 (0.007%)<br>• Trichodesmium erythraeum [taxid 1206]: 1 (0.007%)<br>• Oidiodendron maius [taxid 78148]: 1 (0.007%)<br>• Halomicronema hongdechloris [taxid 1209493]: 1 (0.007%)<br>• other: 34 (0.244%)
Benchmark OTU ID: `CP000435-_Cyanobacteria`<br>OTU taxon: Synechococcus sp. CC9311 [taxid 64471]<br>Expected: Synechococcus [taxid 1129] (genus)<br>Number of reads: 13344<br>Number of identified reads: 13275 (99.482%)	• **genus: 6522 (48.875%)**<br>• family: 644 (4.826%)<br>• order: 929 (6.961%)<br>• phylum: 388 (2.907%)<br>• superkingdom: 1530 (11.465%)<br>• root: 3183 (23.853%)	• Goniomonas pacifica [taxid 195067]: 25 (0.187%)<br>• Synechococcus sp. Ace-Pa [taxid 2572902]: 14 (0.104%)<br>• Synechococcus sp. Lanier [taxid 1910957]: 5 (0.037%)<br>• Synechococcus sp. Dim [taxid 221337]: 5 (0.037%)<br>• Prochlorococcus marinus [taxid 1219]: 4 (0.029%)<br>• Lupinus albus [taxid 3870]: 3 (0.022%)<br>• other: 52 (0.389%)

Operational Taxonomic Unit (OTU)	Correct identifications	Wrong or overspecific identifications at species rank
Benchmark OTU ID: `CP000110-_Cyanobacteria`<br>OTU taxon: Synechococcus sp. CC9605 [taxid 110662]<br>Expected: Synechococcus [taxid 1129] (genus)<br>Number of reads: 12710<br>Number of identified reads: 12650 (99.527%)	• **genus: 7790 (61.29%)**<br>• family: 478 (3.76%)<br>• order: 696 (5.476%)<br>• phylum: 447 (3.516%)<br>• superkingdom: 1113 (8.756%)<br>• root: 2078 (16.349%)	• Synechococcus sp. Ace-Pa [taxid 2572902]: 34 (0.267%)<br>• Synechococcus sp. Lanier [taxid 1910957]: 4 (0.031%)<br>• Prochlorococcus marinus [taxid 1219]: 4 (0.031%)<br>• Synechococcus elongatus [taxid 32046]: 2 (0.015%)<br>• bacterium [taxid 1869227]: 2 (0.015%)<br>• Synechococcus sp. Dim [taxid 221337]: 2 (0.015%)<br>• other: 40 (0.314%)
Benchmark OTU ID: `CP000097-_Cyanobacteria`<br>OTU taxon: Synechococcus sp. CC9902 [taxid 316279]<br>Expected: Synechococcus [taxid 1129] (genus)<br>Number of reads: 10890<br>Number of identified reads: 10837 (99.513%)	• **genus: 6180 (56.749%)**<br>• family: 755 (6.932%)<br>• order: 567 (5.206%)<br>• phylum: 341 (3.131%)<br>• superkingdom: 881 (8.089%)<br>• root: 2081 (19.109%)	• Synechococcus sp. Ace-Pa [taxid 2572902]: 11 (0.101%)<br>• Synechococcus sp. Lanier [taxid 1910957]: 4 (0.036%)<br>• Prochlorococcus marinus [taxid 1219]: 3 (0.027%)<br>• Lupinus albus [taxid 3870]: 2 (0.018%)<br>• Solanum chacoense [taxid 4108]: 2 (0.018%)<br>• Selenomonas sputigena [taxid 69823]: 1 (0.009%)<br>• other: 22 (0.202%)
Benchmark OTU ID: `CP000240-_Cyanobacteria`<br>OTU taxon: Synechococcus sp. JA-2-3B'a(2-13) [taxid 321332]<br>Expected: Synechococcus [taxid 1129] (genus)<br>Number of reads: 16248<br>Number of identified reads: 16106 (99.126%)	• **genus: 11401 (70.168%)**<br>• family: 7 (0.043%)<br>• order: 65 (0.4%)<br>• phylum: 529 (3.255%)<br>• superkingdom: 1432 (8.813%)<br>• root: 2622 (16.137%)	• Synechococcus lividus [taxid 33070]: 4 (0.024%)<br>• Synechococcus sp. Ace-Pa [taxid 2572902]: 3 (0.018%)<br>• Microcystis aeruginosa [taxid 1126]: 2 (0.012%)<br>• Helicobacter pylori [taxid 210]: 2 (0.012%)<br>• Medicago truncatula [taxid 3880]: 2 (0.012%)<br>• Enterococcus faecium [taxid 1352]: 1 (0.006%)<br>• other: 41 (0.252%)
Benchmark OTU ID: `CP000239-_Cyanobacteria`<br>OTU taxon: Synechococcus sp. JA-3-3Ab [taxid 321327]<br>Expected: Synechococcus [taxid 1129] (genus)<br>Number of reads: 15496<br>Number of identified reads: 15336 (98.967%)	• **genus: 11139 (71.883%)**<br>• family: 3 (0.019%)<br>• order: 57 (0.367%)<br>• phylum: 526 (3.394%)<br>• superkingdom: 1474 (9.512%)<br>• root: 2101 (13.558%)	• Chloracidobacterium thermophilum [taxid 458033]: 6 (0.038%)<br>• Thermus aquaticus [taxid 271]: 6 (0.038%)<br>• Synechococcus lividus [taxid 33070]: 3 (0.019%)<br>• Synechococcus sp. Ace-Pa [taxid 2572902]: 2 (0.012%)<br>• Microcystis aeruginosa [taxid 1126]: 2 (0.012%)<br>• Klebsormidium nitens [taxid 105231]: 1 (0.006%)<br>• other: 31 (0.2%)
Benchmark OTU ID: `CP003558-_Cyanobacteria`<br>OTU taxon: Synechococcus sp. PCC 6312 [taxid 195253]<br>Expected: Synechococcus [taxid 1129] (genus)<br>Number of reads: 20542<br>Number of identified reads: 20360 (99.114%)	• **genus: 14165 (68.956%)**<br>• family: 39 (0.189%)<br>• order: 184 (0.895%)<br>• phylum: 1297 (6.313%)<br>• superkingdom: 1495 (7.277%)<br>• root: 3136 (15.266%)	• Synechococcus lividus [taxid 33070]: 7 (0.034%)<br>• Synechococcus sp. Ace-Pa [taxid 2572902]: 3 (0.014%)<br>• Thermosynechococcus elongatus [taxid 146786]: 3 (0.014%)<br>• Crocosphaera watsonii [taxid 263511]: 2 (0.009%)<br>• Heliobacterium modesticaldum [taxid 35701]: 2 (0.009%)<br>• Phormidium tenue [taxid 126344]: 2 (0.009%)<br>• other: 48 (0.233%)

Operational Taxonomic Unit (OTU)	Correct identifications	Wrong or overspecific identifications at species rank
Benchmark OTU ID: `CP000951-_Cyanobacteria`<br>OTU taxon: Synechococcus sp. PCC 7002 [taxid 32049]<br>Expected: Synechococcus [taxid 1129] (genus)<br>Number of reads: 15993<br>Number of identified reads: 15913 (99.499%)	• **genus: 10818 (67.642%)**<br>• family: 5 (0.031%)<br>• order: 572 (3.576%)<br>• phylum: 1241 (7.759%)<br>• superkingdom: 1017 (6.359%)<br>• root: 2218 (13.868%)	• Synechococcus elongatus [taxid 32046]: 4 (0.025%)<br>• Prochlorococcus marinus [taxid 1219]: 3 (0.018%)<br>• Scytonema hofmannii [taxid 34078]: 2 (0.012%)<br>• Microcystis aeruginosa [taxid 1126]: 2 (0.012%)<br>• Westiellopsis prolifica [taxid 221298]: 2 (0.012%)<br>• Nymphaea colorata [taxid 210225]: 2 (0.012%)<br>• other: 54 (0.337%)
Benchmark OTU ID: `CP003594-_Cyanobacteria`<br>OTU taxon: Synechococcus sp. PCC 7502 [taxid 1173263]<br>Expected: Synechococcus [taxid 1129] (genus)<br>Number of reads: 19307<br>Number of identified reads: 19115 (99.005%)	• **genus: 13880 (71.891%)**<br>• family: 6 (0.031%)<br>• order: 389 (2.014%)<br>• phylum: 1094 (5.666%)<br>• superkingdom: 1316 (6.816%)<br>• root: 2382 (12.337%)	• Gloeomargarita lithophora [taxid 1188228]: 3 (0.015%)<br>• Neosynechococcus sphagnicola [taxid 1501145]: 3 (0.015%)<br>• Helicobacter pylori [taxid 210]: 2 (0.01%)<br>• Merismopedia glauca [taxid 292586]: 2 (0.01%)<br>• Medicago truncatula [taxid 3880]: 2 (0.01%)<br>• Chamaesiphon polymorphus [taxid 2107691]: 2 (0.01%)<br>• other: 50 (0.258%)
Benchmark OTU ID: `CT978603-_Cyanobacteria`<br>OTU taxon: Synechococcus sp. RCC307 [taxid 316278]<br>Expected: Synechococcus [taxid 1129] (genus)<br>Number of reads: 10820<br>Number of identified reads: 10769 (99.528%)	• genus: 2743 (25.351%)<br>• family: 128 (1.182%)<br>• **order: 5306 (49.038%)**<br>• phylum: 240 (2.218%)<br>• superkingdom: 966 (8.927%)<br>• root: 1354 (12.513%)	• Synechococcus sp. Ace-Pa [taxid 2572902]: 11 (0.101%)<br>• Synechococcus sp. Lanier [taxid 1910957]: 11 (0.101%)<br>• Lupinus albus [taxid 3870]: 2 (0.018%)<br>• Candidatus Synechococcus spongiarum [taxid 431041]: 2 (0.018%)<br>• Merismopedia glauca [taxid 292586]: 1 (0.009%)<br>• Aphanocapsa feldmannii [taxid 192050]: 1 (0.009%)<br>• other: 31 (0.286%)
Benchmark OTU ID: `CT971583-_Cyanobacteria`<br>OTU taxon: Synechococcus sp. WH 7803 [taxid 32051]<br>Expected: Synechococcus [taxid 1129] (genus)<br>Number of reads: 11762<br>Number of identified reads: 11717 (99.617%)	• **genus: 7473 (63.535%)**<br>• family: 440 (3.74%)<br>• order: 689 (5.857%)<br>• phylum: 299 (2.542%)<br>• superkingdom: 1024 (8.706%)<br>• root: 1755 (14.92%)	• Synechococcus sp. Ace-Pa [taxid 2572902]: 19 (0.161%)<br>• Prochlorococcus marinus [taxid 1219]: 10 (0.085%)<br>• Synechococcus sp. Lanier [taxid 1910957]: 9 (0.076%)<br>• Synechococcus sp. Dim [taxid 221337]: 7 (0.059%)<br>• Candidatus Synechococcus spongiarum [taxid 431041]: 5 (0.042%)<br>• Solanum chacoense [taxid 4108]: 2 (0.017%)<br>• other: 31 (0.263%)
Benchmark OTU ID: `ENA|BX548020|BX548020.1-_Cyanobacteria`<br>OTU taxon: Synechococcus sp. WH 8102 [taxid 84588]<br>Expected: Synechococcus [taxid 1129] (genus)<br>Number of reads: 12207<br>Number of identified reads: 12158 (99.598%)	• **genus: 7269 (59.547%)**<br>• family: 733 (6.004%)<br>• order: 723 (5.922%)<br>• phylum: 426 (3.489%)<br>• superkingdom: 1101 (9.019%)<br>• root: 1861 (15.245%)	• Synechococcus sp. Ace-Pa [taxid 2572902]: 31 (0.253%)<br>• Synechococcus sp. Lanier [taxid 1910957]: 9 (0.073%)<br>• Synechococcus sp. Dim [taxid 221337]: 5 (0.04%)<br>• cyanobiont of Ornithocercus magnificus [taxid 2496102]: 3 (0.024%)<br>• Aphanocapsa feldmannii [taxid 192050]: 2 (0.016%)<br>• Tychonema bourrellyi [taxid 54313]: 2 (0.016%)<br>• other: 32 (0.262%)

Operational Taxonomic Unit (OTU)	Correct identifications	Wrong or overspecific identifications at species rank
Benchmark OTU ID: `AP012205-_Cyanobacteria`<br>OTU taxon: Synechocystis sp. PCC 6803 [taxid 1148]<br>Expected: Synechocystis [taxid 1142] (genus)<br>Number of reads: 19709<br>Number of identified reads: 19554 (99.213%)	• **genus: 13913 (70.592%)**<br>• family: 5 (0.025%)<br>• order: 219 (1.111%)<br>• phylum: 1513 (7.676%)<br>• superkingdom: 1300 (6.595%)<br>• root: 2557 (12.973%)	• Synechocystis sp. AHZ-HB-MK [taxid 372073]: 7 (0.035%)<br>• Mastigocoleus testarum [taxid 996925]: 2 (0.01%)<br>• Phormidium sp. OSCR [taxid 1666905]: 2 (0.01%)<br>• Microcystis aeruginosa [taxid 1126]: 2 (0.01%)<br>• Calypogeia fissa [taxid 362796]: 1 (0.005%)<br>• Odontella aurita [taxid 265563]: 1 (0.005%)<br>• other: 33 (0.167%)
Benchmark OTU ID: `BA000022-_Cyanobacteria`<br>OTU taxon: Synechocystis sp. PCC 6803 [taxid 1148]<br>Expected: Synechocystis [taxid 1142] (genus)<br>Number of reads: 19724<br>Number of identified reads: 19593 (99.335%)	• **genus: 13767 (69.798%)**<br>• family: 5 (0.025%)<br>• order: 249 (1.262%)<br>• phylum: 1650 (8.365%)<br>• superkingdom: 1292 (6.55%)<br>• root: 2582 (13.09%)	• Trichodesmium erythraeum [taxid 1206]: 3 (0.015%)<br>• Synechocystis sp. AHZ-HB-MK [taxid 372073]: 3 (0.015%)<br>• Microcystis aeruginosa [taxid 1126]: 2 (0.01%)<br>• Leptolyngbya sp. 'hensonii' [taxid 1922337]: 2 (0.01%)<br>• Chamaesiphon minutus [taxid 1173032]: 2 (0.01%)<br>• cyanobacterium endosymbiont of Rhopalodia gibberula [taxid 1763363]: 2 (0.01%)<br>• other: 55 (0.278%)
Benchmark OTU ID: `CP003265-_Cyanobacteria`<br>OTU taxon: Synechocystis sp. PCC 6803 [taxid 1148]<br>Expected: Synechocystis [taxid 1142] (genus)<br>Number of reads: 19699<br>Number of identified reads: 19549 (99.238%)	• **genus: 13830 (70.206%)**<br>• family: 5 (0.025%)<br>• order: 222 (1.126%)<br>• phylum: 1550 (7.868%)<br>• superkingdom: 1262 (6.406%)<br>• root: 2631 (13.356%)	• Synechocystis sp. AHZ-HB-MK [taxid 372073]: 4 (0.02%)<br>• Microcystis aeruginosa [taxid 1126]: 3 (0.015%)<br>• Trichodesmium erythraeum [taxid 1206]: 2 (0.01%)<br>• Candidatus Atelocyanobacterium thalassa [taxid 713887]: 2 (0.01%)<br>• Gloeothece citriformis [taxid 2546356]: 2 (0.01%)<br>• Mycobacteroides abscessus [taxid 36809]: 1 (0.005%)<br>• other: 43 (0.218%)
Benchmark OTU ID: `AP012276-_Cyanobacteria`<br>OTU taxon: Synechocystis sp. PCC 6803 substr. GT-I [taxid 1080228]<br>Expected: Synechocystis [taxid 1142] (genus)<br>Number of reads: 19702<br>Number of identified reads: 19584 (99.401%)	• **genus: 13751 (69.794%)**<br>• family: 7 (0.035%)<br>• order: 224 (1.136%)<br>• phylum: 1692 (8.587%)<br>• superkingdom: 1247 (6.329%)<br>• root: 2605 (13.222%)	• Dorcoceras hygrometricum [taxid 472368]: 3 (0.015%)<br>• Candidatus Atelocyanobacterium thalassa [taxid 713887]: 2 (0.01%)<br>• Merismopedia glauca [taxid 292586]: 2 (0.01%)<br>• Lupinus albus [taxid 3870]: 2 (0.01%)<br>• Limnospira indica [taxid 147322]: 1 (0.005%)<br>• Listeria fleischmannii [taxid 1069827]: 1 (0.005%)<br>• other: 51 (0.258%)
Benchmark OTU ID: `AP012277-_Cyanobacteria`<br>OTU taxon: Synechocystis sp. PCC 6803 substr. PCC-N [taxid 1080229]<br>Expected: Synechocystis [taxid 1142] (genus)<br>Number of reads: 19703<br>Number of identified reads: 19560 (99.274%)	• **genus: 13806 (70.07%)**<br>• family: 3 (0.015%)<br>• order: 233 (1.182%)<br>• phylum: 1594 (8.09%)<br>• superkingdom: 1261 (6.4%)<br>• root: 2620 (13.297%)	• Lyngbya confervoides [taxid 207921]: 3 (0.015%)<br>• Cellulomonas soli [taxid 931535]: 2 (0.01%)<br>• Synechocystis sp. AHZ-HB-MK [taxid 372073]: 2 (0.01%)<br>• Leptolyngbya boryana [taxid 1184]: 2 (0.01%)<br>• Lupinus albus [taxid 3870]: 2 (0.01%)<br>• Moraxella osloensis [taxid 34062]: 2 (0.01%)<br>• other: 44 (0.223%)

Operational Taxonomic Unit (OTU)	Correct identifications	Wrong or overspecific identifications at species rank
Benchmark OTU ID: `AP012278-_Cyanobacteria`<br>OTU taxon: Synechocystis sp. PCC 6803 substr. PCC-P [taxid 1080230]<br>Expected: Synechocystis [taxid 1142] (genus)<br>Number of reads: 19703<br>Number of identified reads: 19579 (99.37%)	• **genus: 13832 (70.202%)**<br>• family: 1 (0.005%)<br>• order: 221 (1.121%)<br>• phylum: 1600 (8.12%)<br>• superkingdom: 1225 (6.217%)<br>• root: 2652 (13.459%)	• Synechocystis sp. AHZ-HB-MK [taxid 372073]: 6 (0.03%)<br>• Synechococcus elongatus [taxid 32046]: 3 (0.015%)<br>• Dorcoceras hygrometricum [taxid 472368]: 2 (0.01%)<br>• Gloeomargarita lithophora [taxid 1188228]: 2 (0.01%)<br>• cyanobacterium endosymbiont of Epithemia turgida [taxid 718217]: 2 (0.01%)<br>• Leptolyngbya sp. 'hensonii' [taxid 1922337]: 1 (0.005%)<br>• other: 36 (0.182%)
Benchmark OTU ID: `CP000478-_Proteobacteria`<br>OTU taxon: Syntrophobacter fumaroxidans MPOB [taxid 335543]<br>Expected: Syntrophobacter fumaroxidans [taxid 119484] (species)<br>Number of reads: 10344<br>Number of identified reads: 10262 (99.207%)	• **species: 7020 (67.865%)**<br>• genus: 6 (0.058%)<br>• family: 45 (0.435%)<br>• order: 9 (0.087%)<br>• class: 249 (2.407%)<br>• phylum: 237 (2.291%)<br>• superkingdom: 741 (7.163%)<br>• root: 1939 (18.745%)	• Pseudodesulfovibrio profundus [taxid 57320]: 2 (0.019%)<br>• Thermodesulfobacterium geofontis [taxid 1295609]: 1 (0.009%)<br>• Kushneria phosphatilytica [taxid 657387]: 1 (0.009%)<br>• Chlorobaculum parvum [taxid 274539]: 1 (0.009%)<br>• Coleofasciculus chthonoplastes [taxid 64178]: 1 (0.009%)<br>• Desulfoplanes formicivorans [taxid 1592317]: 1 (0.009%)<br>• Leucoagaricus sp. SymC.cos [taxid 1714833]: 1 (0.009%)<br>• Desulfoglaeba alkanexedens [taxid 361111]: 1 (0.009%)<br>• other: 26 (0.251%)
Benchmark OTU ID: `CP002547-_Firmicutes`<br>OTU taxon: Syntrophobotulus glycolicus DSM 8271 [taxid 645991]<br>Expected: Syntrophobotulus glycolicus [taxid 51197] (species)<br>Number of reads: 4682<br>Number of identified reads: 4659 (99.508%)	• **species: 3297 (70.418%)**<br>• genus: 0 (0.0%)<br>• family: 61 (1.302%)<br>• order: 151 (3.225%)<br>• class: 7 (0.149%)<br>• phylum: 130 (2.776%)<br>• superkingdom: 310 (6.621%)<br>• root: 696 (14.865%)	• Oxobacter pfennigii [taxid 36849]: 1 (0.021%)<br>• Hordeum vulgare [taxid 4513]: 1 (0.021%)<br>• Candidatus Bathyarchaeota archaeon [taxid 2026714]: 1 (0.021%)<br>• Peribacillus simplex [taxid 1478]: 1 (0.021%)<br>• Chryseobacterium oranimense [taxid 421058]: 1 (0.021%)<br>• Lutispora thermophila [taxid 288966]: 1 (0.021%)<br>• Syntrophomonas wolfei [taxid 863]: 1 (0.021%)<br>• Desulfitobacterium hafniense [taxid 49338]: 1 (0.021%)<br>• other: 14 (0.299%)
Benchmark OTU ID: `CP002048-_Firmicutes`<br>OTU taxon: Syntrophothermus lipocalidus DSM 12680 [taxid 643648]<br>Expected: Syntrophothermus lipocalidus [taxid 86170] (species)<br>Number of reads: 3080<br>Number of identified reads: 3066 (99.545%)	• **species: 2039 (66.201%)**<br>• genus: 0 (0.0%)<br>• family: 10 (0.324%)<br>• order: 21 (0.681%)<br>• class: 16 (0.519%)<br>• phylum: 61 (1.98%)<br>• superkingdom: 295 (9.577%)<br>• root: 620 (20.129%)	• Adhaeribacter arboris [taxid 2072846]: 1 (0.032%)<br>• Micromonospora inositola [taxid 47865]: 1 (0.032%)<br>• Latilactobacillus sakei [taxid 1599]: 1 (0.032%)<br>• Ralstonia solanacearum [taxid 305]: 1 (0.032%)<br>• Caulifigura coniformis [taxid 2527983]: 1 (0.032%)<br>• Agrilactobacillus composti [taxid 398555]: 1 (0.032%)<br>• Paxillus rubicundulus [taxid 463315]: 1 (0.032%)
Benchmark OTU ID: `CP003264-_Proteobacteria`<br>OTU taxon: Taylorella equigenitalis ATCC 35865 [taxid 743973]<br>Expected: Taylorella equigenitalis [taxid 29575] (species)<br>Number of reads: 3013<br>Number of identified reads: 2984 (99.037%)	• **species: 1965 (65.217%)**<br>• genus: 263 (8.728%)<br>• family: 44 (1.46%)<br>• order: 52 (1.725%)<br>• class: 38 (1.261%)<br>• phylum: 194 (6.438%)<br>• superkingdom: 134 (4.447%)<br>• root: 291 (9.658%)	• Taylorella asinigenitalis [taxid 84590]: 38 (1.261%)<br>• Poeciliopsis prolifica [taxid 188132]: 2 (0.066%)<br>• Nitrosomonas aestuarii [taxid 52441]: 1 (0.033%)<br>• Enterococcus mundtii [taxid 53346]: 1 (0.033%)<br>• Candidatus Phytoplasma phoenicium [taxid 198422]: 1 (0.033%)<br>• Mesorhizobium tamadayense [taxid 425306]: 1 (0.033%)<br>• Cupriavidus necator [taxid 106590]: 1 (0.033%)<br>• Burkholderia multivorans [taxid 87883]: 1 (0.033%)

Operational Taxonomic Unit (OTU)	Correct identifications	Wrong or overspecific identifications at species rank
Benchmark OTU ID: `CP002456-_Proteobacteria`<br>OTU taxon: Taylorella equigenitalis MCE9 [taxid 937774]<br>Expected: Taylorella equigenitalis [taxid 29575] (species)<br>Number of reads: 2932<br>Number of identified reads: 2901 (98.942%)	• **species: 1993 (67.974%)**<br>• genus: 223 (7.605%)<br>• family: 33 (1.125%)<br>• order: 48 (1.637%)<br>• class: 35 (1.193%)<br>• phylum: 187 (6.377%)<br>• superkingdom: 129 (4.399%)<br>• root: 253 (8.628%)	• Taylorella asinigenitalis [taxid 84590]: 5 (0.17%)<br>• Streptococcus salivarius [taxid 1304]: 1 (0.034%)<br>• Dyadobacter jejuensis [taxid 1082580]: 1 (0.034%)<br>• Alcanivorax hongdengensis [taxid 519051]: 1 (0.034%)<br>• Ottowia oryzae [taxid 2109914]: 1 (0.034%)<br>• Acinetobacter sichuanensis [taxid 2136183]: 1 (0.034%)<br>• [Polyangium] brachysporum [taxid 413882]: 1 (0.034%)<br>• Pararhodospirillum photometricum [taxid 1084]: 1 (0.034%)
Benchmark OTU ID: `CP003379-_Acidobacteria`<br>OTU taxon: Terriglobus roseus DSM 18391 [taxid 926566]<br>Expected: Terriglobus roseus [taxid 392734] (species)<br>Number of reads: 413535<br>Number of identified reads: 412173 (99.67%)	• **species: 303284 (73.339%)**<br>• genus: 2861 (0.691%)<br>• family: 9036 (2.185%)<br>• order: 1119 (0.27%)<br>• class: 376 (0.09%)<br>• phylum: 3762 (0.909%)<br>• superkingdom: 37689 (9.113%)<br>• root: 53627 (12.967%)	• Terriglobus saanensis [taxid 870903]: 137 (0.033%)<br>• Terriglobus albidus [taxid 1592106]: 49 (0.011%)<br>• Edaphobacter modestus [taxid 388466]: 36 (0.008%)<br>• Bryocella elongata [taxid 863522]: 28 (0.006%)<br>• Candidatus Solibacter usitatus [taxid 332163]: 28 (0.006%)<br>• Acidisarcina polymorpha [taxid 2211140]: 24 (0.005%)<br>• Granulicella rosea [taxid 474952]: 20 (0.004%)<br>• Edaphobacter dinghuensis [taxid 1560005]: 16 (0.003%)<br>• other: 776 (0.187%)
Benchmark OTU ID: `CP002467-_Acidobacteria`<br>OTU taxon: Terriglobus saanensis SP1PR4 [taxid 401053]<br>Expected: Terriglobus saanensis [taxid 870903] (species)<br>Number of reads: 401628<br>Number of identified reads: 400930 (99.826%)	• **species: 285064 (70.977%)**<br>• genus: 3480 (0.866%)<br>• family: 12793 (3.185%)<br>• order: 1790 (0.445%)<br>• class: 311 (0.077%)<br>• phylum: 3872 (0.964%)<br>• superkingdom: 36274 (9.031%)<br>• root: 56956 (14.181%)	• Terriglobus roseus [taxid 392734]: 101 (0.025%)<br>• Terriglobus albidus [taxid 1592106]: 63 (0.015%)<br>• Granulicella mallensis [taxid 940614]: 47 (0.011%)<br>• Granulicella pectinivorans [taxid 474950]: 45 (0.011%)<br>• Granulicella rosea [taxid 474952]: 38 (0.009%)<br>• Granulicella sibirica [taxid 2479048]: 36 (0.008%)<br>• Edaphobacter dinghuensis [taxid 1560005]: 33 (0.008%)<br>• Bryocella elongata [taxid 863522]: 31 (0.007%)<br>• other: 815 (0.202%)
Benchmark OTU ID: `CP003732-_Firmicutes`<br>OTU taxon: Thermacetogenium phaeum DSM 12270 [taxid 1089553]<br>Expected: Thermacetogenium phaeum [taxid 85874] (species)<br>Number of reads: 3933<br>Number of identified reads: 3909 (99.389%)	• **species: 2415 (61.403%)**<br>• genus: 0 (0.0%)<br>• family: 25 (0.635%)<br>• order: 12 (0.305%)<br>• class: 287 (7.297%)<br>• phylum: 69 (1.754%)<br>• superkingdom: 334 (8.492%)<br>• root: 763 (19.399%)	• Aeromonas lusitana [taxid 931529]: 1 (0.025%)<br>• Venturia effusa [taxid 50376]: 1 (0.025%)<br>• Moorella thermoacetica [taxid 1525]: 1 (0.025%)<br>• Thermosediminibacter litoriperuensis [taxid 291989]: 1 (0.025%)<br>• Phascolarctobacterium succinatutens [taxid 626940]: 1 (0.025%)<br>• Eumeta japonica [taxid 151549]: 1 (0.025%)<br>• Heliobacterium modesticaldum [taxid 35701]: 1 (0.025%)<br>• Carboxydothermus islandicus [taxid 661089]: 1 (0.025%)<br>• other: 3 (0.076%)

Operational Taxonomic Unit (OTU)	Correct identifications	Wrong or overspecific identifications at species rank
Benchmark OTU ID: `CP002344-_Firmicutes`<br>OTU taxon: Thermaerobacter marianensis DSM 12885 [taxid 644966]<br>Expected: Thermaerobacter marianensis [taxid 73919] (species)<br>Number of reads: 3782<br>Number of identified reads: 3748 (99.101%)	• **species: 2002 (52.934%)**<br>• genus: 248 (6.557%)<br>• family: 0 (0.0%)<br>• order: 9 (0.237%)<br>• class: 5 (0.132%)<br>• phylum: 409 (10.814%)<br>• superkingdom: 602 (15.917%)<br>• root: 468 (12.374%)	• Thermaerobacter subterraneus [taxid 175696]: 9 (0.237%)<br>• bacterium [taxid 1869227]: 1 (0.026%)<br>• Calderihabitans maritimus [taxid 1246530]: 1 (0.026%)<br>• Arthrobacter woluwensis [taxid 156980]: 1 (0.026%)<br>• Geobacter argillaceus [taxid 345631]: 1 (0.026%)<br>• Conexibacter woesei [taxid 191495]: 1 (0.026%)<br>• Streptococcus sobrinus [taxid 1310]: 1 (0.026%)<br>• Natranaerobius trueperi [taxid 759412]: 1 (0.026%)<br>• other: 8 (0.211%)
Benchmark OTU ID: `CP002028-_Firmicutes`<br>OTU taxon: Thermincola potens JR [taxid 635013]<br>Expected: Thermincola potens [taxid 863643] (species)<br>Number of reads: 4283<br>Number of identified reads: 4241 (99.019%)	• species: 1005 (23.464%)<br>• **genus: 1935 (45.178%)**<br>• family: 55 (1.284%)<br>• order: 38 (0.887%)<br>• class: 28 (0.653%)<br>• phylum: 177 (4.132%)<br>• superkingdom: 328 (7.658%)<br>• root: 669 (15.619%)	• Thermincola ferriacetica [taxid 281456]: 30 (0.7%)<br>• Pelotomaculum schinkii [taxid 78350]: 1 (0.023%)<br>• Brandtodinium nutricula [taxid 1333877]: 1 (0.023%)<br>• Chromobacterium violaceum [taxid 536]: 1 (0.023%)<br>• Aquisalimonas asiatica [taxid 406100]: 1 (0.023%)<br>• Maledivibacter halophilus [taxid 36842]: 1 (0.023%)<br>• Thiocapsa roseopersicina [taxid 1058]: 1 (0.023%)<br>• [Ruminococcus] torques [taxid 33039]: 1 (0.023%)<br>• other: 17 (0.396%)
Benchmark OTU ID: `CP002210-_Firmicutes`<br>OTU taxon: Thermoanaerobacter sp. X513 [taxid 573062]<br>Expected: Thermoanaerobacter [taxid 1754] (genus)<br>Number of reads: 3161<br>Number of identified reads: 3111 (98.418%)	• **genus: 1667 (52.736%)**<br>• family: 312 (9.87%)<br>• order: 38 (1.202%)<br>• class: 378 (11.958%)<br>• phylum: 108 (3.416%)<br>• superkingdom: 221 (6.991%)<br>• root: 384 (12.148%)	• Thermoanaerobacter uzonensis [taxid 447593]: 7 (0.221%)<br>• Caldanaerobacter subterraneus [taxid 911092]: 5 (0.158%)<br>• Thermoanaerobacter kivui [taxid 2325]: 4 (0.126%)<br>• Thermoanaerobacter thermocopriae [taxid 29350]: 4 (0.126%)<br>• Thermoanaerobacter ethanolicus [taxid 1757]: 3 (0.094%)<br>• Thermoanaerobacter thermohydrosulfuricus [taxid 1516]: 2 (0.063%)<br>• Thermoanaerobacter brockii [taxid 29323]: 2 (0.063%)<br>• other: 17 (0.537%)
Benchmark OTU ID: `CP002991-_Firmicutes`<br>OTU taxon: Thermoanaerobacter wiegelii Rt8.B1 [taxid 697303]<br>Expected: Thermoanaerobacter wiegelii [taxid 46354] (species)<br>Number of reads: 3687<br>Number of identified reads: 3613 (97.992%)	• species: 621 (16.842%)<br>• **genus: 1394 (37.808%)**<br>• family: 325 (8.814%)<br>• order: 58 (1.573%)<br>• class: 460 (12.476%)<br>• phylum: 95 (2.576%)<br>• superkingdom: 232 (6.292%)<br>• root: 424 (11.499%)	• Thermoanaerobacter thermohydrosulfuricus [taxid 1516]: 9 (0.244%)<br>• Thermoanaerobacter kivui [taxid 2325]: 9 (0.244%)<br>• Caldanaerobacter subterraneus [taxid 911092]: 6 (0.162%)<br>• Thermoanaerobacter uzonensis [taxid 447593]: 5 (0.135%)<br>• Thermoanaerobacter mathranii [taxid 583357]: 3 (0.081%)<br>• Thermoanaerobacter thermocopriae [taxid 29350]: 2 (0.054%)<br>• Calorimonas adulescens [taxid 2606906]: 2 (0.054%)<br>• Thermoanaerobacter italicus [taxid 108150]: 2 (0.054%)<br>• other: 19 (0.515%)

Operational Taxonomic Unit (OTU)	Correct identifications	Wrong or overspecific identifications at species rank
Benchmark OTU ID: `CP003184-_Firmicutes`<br>OTU taxon: Thermoanaerobacterium saccharolyticum JW/SL-YS485 [taxid 1094508]<br>Expected: Thermoanaerobacterium saccharolyticum [taxid 28896] (species)<br>Number of reads: 3593<br>Number of identified reads: 3544 (98.636%)	• species: 28 (0.779%)<br>• **genus: 2188 (60.896%)**<br>• family: 1 (0.027%)<br>• order: 67 (1.864%)<br>• class: 440 (12.246%)<br>• phylum: 93 (2.588%)<br>• superkingdom: 234 (6.512%)<br>• root: 486 (13.526%)	• Thermoanaerobacterium aotearoense [taxid 47490]: 12 (0.333%)<br>• Thermoanaerobacterium sp. RBIITD [taxid 1550240]: 12 (0.333%)<br>• Thermoanaerobacterium thermosaccharolyticum [taxid 1517]: 11 (0.306%)<br>• Thermoanaerobacterium xylanolyticum [taxid 29329]: 4 (0.111%)<br>• Caldanaerobacter subterraneus [taxid 911092]: 2 (0.055%)<br>• Citrobacter freundii [taxid 546]: 1 (0.027%)<br>• Hungateiclostridium thermocellum [taxid 1515]: 1 (0.027%)<br>• Clostridium grantii [taxid 40575]: 1 (0.027%)<br>• other: 6 (0.166%)
Benchmark OTU ID: `CP002739-_Firmicutes`<br>OTU taxon: Thermoanaerobacterium xylanolyticum LX-11 [taxid 858215]<br>Expected: Thermoanaerobacterium xylanolyticum [taxid 29329] (species)<br>Number of reads: 3286<br>Number of identified reads: 3236 (98.478%)	• species: 968 (29.458%)<br>• **genus: 1061 (32.288%)**<br>• family: 1 (0.03%)<br>• order: 62 (1.886%)<br>• class: 426 (12.964%)<br>• phylum: 79 (2.404%)<br>• superkingdom: 210 (6.39%)<br>• root: 426 (12.964%)	• Thermoanaerobacterium sp. RBIITD [taxid 1550240]: 8 (0.243%)<br>• Thermoanaerobacterium thermosaccharolyticum [taxid 1517]: 7 (0.213%)<br>• Thermoanaerobacter italicus [taxid 108150]: 2 (0.06%)<br>• Enterocloster clostridioformis [taxid 1531]: 2 (0.06%)<br>• Thermoanaerobacter kivui [taxid 2325]: 2 (0.06%)<br>• Maledivibacter halophilus [taxid 36842]: 1 (0.03%)<br>• Triticum turgidum [taxid 4571]: 1 (0.03%)<br>• Thermoanaerobacter wiegelii [taxid 46354]: 1 (0.03%)<br>• other: 11 (0.334%)
Benchmark OTU ID: `CP000088-_Actinobacteria`<br>OTU taxon: Thermobifida fusca YX [taxid 269800]<br>Expected: Thermobifida fusca [taxid 2021] (species)<br>Number of reads: 15444<br>Number of identified reads: 15250 (98.743%)	• **species: 8604 (55.71%)**<br>• genus: 754 (4.882%)<br>• family: 574 (3.716%)<br>• order: 227 (1.469%)<br>• class: 1739 (11.26%)<br>• phylum: 16 (0.103%)<br>• superkingdom: 1000 (6.475%)<br>• root: 2315 (14.989%)	• Thermobifida cellulosilytica [taxid 144786]: 22 (0.142%)<br>• Thermobifida halotolerans [taxid 483545]: 10 (0.064%)<br>• Nocardiopsis gilva [taxid 280236]: 3 (0.019%)<br>• Nocardiopsis alba [taxid 53437]: 2 (0.012%)<br>• Marinitenerispora sediminis [taxid 1931232]: 2 (0.012%)<br>• Sorangium cellulosum [taxid 56]: 1 (0.006%)<br>• Nocardiopsis flavescens [taxid 758803]: 1 (0.006%)<br>• Thermasporomyces composti [taxid 696763]: 1 (0.006%)<br>• other: 55 (0.356%)
Benchmark OTU ID: `CP001874-_Actinobacteria`<br>OTU taxon: Thermobispora bispora DSM 43833 [taxid 469371]<br>Expected: Thermobispora bispora [taxid 2006] (species)<br>Number of reads: 18128<br>Number of identified reads: 18010 (99.349%)	• **species: 9894 (54.578%)**<br>• genus: 0 (0.0%)<br>• class: 4728 (26.081%)<br>• phylum: 24 (0.132%)<br>• superkingdom: 1387 (7.651%)<br>• root: 1953 (10.773%)	• Thermomonospora curvata [taxid 2020]: 4 (0.022%)<br>• Desulfovibrio magneticus [taxid 184917]: 3 (0.016%)<br>• Thermostaphylospora chromogena [taxid 35622]: 3 (0.016%)<br>• Ephemera danica [taxid 1049336]: 2 (0.011%)<br>• Streptosporangium canum [taxid 324952]: 2 (0.011%)<br>• Nonomuraea jiangxiensis [taxid 633440]: 2 (0.011%)<br>• other: 109 (0.601%)

Operational Taxonomic Unit (OTU)	Correct identifications	Wrong or overspecific identifications at species rank
Benchmark OTU ID: `CP001398-_Euryarchaeota`<br>OTU taxon: Thermococcus gammatolerans EJ3 [taxid 593117]<br>Expected: Thermococcus gammatolerans [taxid 187878] (species)<br>Number of reads: 1751<br>Number of identified reads: 1743 (99.543%)	• **species: 664 (37.921%)**<br>• genus: 375 (21.416%)<br>• family: 53 (3.026%)<br>• order: 2 (0.114%)<br>• class: 34 (1.941%)<br>• phylum: 11 (0.628%)<br>• superkingdom: 19 (1.085%)<br>• root: 554 (31.639%)	• Thermococcus celer [taxid 2264]: 2 (0.114%)<br>• Thermococcus gorgonarius [taxid 71997]: 2 (0.114%)<br>• Thermococcus piezophilus [taxid 1712654]: 2 (0.114%)<br>• Rhodosorus marinus [taxid 101924]: 1 (0.057%)<br>• Candidatus Bathyarchaeota archaeon [taxid 2026714]: 1 (0.057%)<br>• Thermococcus litoralis [taxid 2265]: 1 (0.057%)<br>• Zostera marina [taxid 29655]: 1 (0.057%)<br>• Thermococcus guaymasensis [taxid 110164]: 1 (0.057%)<br>• other: 5 (0.285%)
Benchmark OTU ID: `AP006878-_Euryarchaeota`<br>OTU taxon: Thermococcus kodakarensis KOD1 [taxid 69014]<br>Expected: Thermococcus kodakarensis [taxid 311400] (species)<br>Number of reads: 1796<br>Number of identified reads: 1785 (99.387%)	• **species: 688 (38.307%)**<br>• genus: 357 (19.877%)<br>• family: 46 (2.561%)<br>• order: 3 (0.167%)<br>• class: 52 (2.895%)<br>• phylum: 9 (0.501%)<br>• superkingdom: 15 (0.835%)<br>• root: 587 (32.683%)	• Thermococcus peptonophilus [taxid 53952]: 2 (0.111%)<br>• Phocaeicola plebeius [taxid 310297]: 1 (0.055%)<br>• Thermococcus gorgonarius [taxid 71997]: 1 (0.055%)<br>• Salmonella enterica [taxid 28901]: 1 (0.055%)<br>• Thermococcus nautili [taxid 195522]: 1 (0.055%)<br>• Pyrococcus yayanosii [taxid 1008460]: 1 (0.055%)<br>• Thermococcus thioreducens [taxid 277988]: 1 (0.055%)<br>• Thermococcus litoralis [taxid 2265]: 1 (0.055%)<br>• other: 5 (0.278%)
Benchmark OTU ID: `CP000855-_Euryarchaeota`<br>OTU taxon: Thermococcus onnurineus NA1 [taxid 523850]<br>Expected: Thermococcus onnurineus [taxid 342948] (species)<br>Number of reads: 1543<br>Number of identified reads: 1533 (99.351%)	• **species: 621 (40.246%)**<br>• genus: 304 (19.701%)<br>• family: 49 (3.175%)<br>• order: 0 (0.0%)<br>• class: 31 (2.009%)<br>• phylum: 5 (0.324%)<br>• superkingdom: 18 (1.166%)<br>• root: 486 (31.497%)	• Thermococcus celericrescens [taxid 227598]: 3 (0.194%)<br>• Thermococcus profundus [taxid 49899]: 2 (0.129%)<br>• Thermococcus celer [taxid 2264]: 2 (0.129%)<br>• Thermococcus eurythermalis [taxid 1505907]: 2 (0.129%)<br>• Thermococcus piezophilus [taxid 1712654]: 2 (0.129%)<br>• Lucilia cuprina [taxid 7375]: 1 (0.064%)<br>• Thermococcus litoralis [taxid 2265]: 1 (0.064%)<br>• Thermococcus cleftensis [taxid 163003]: 1 (0.064%)<br>• other: 4 (0.259%)
Benchmark OTU ID: `CP002920-_Euryarchaeota`<br>OTU taxon: Thermococcus sp. 4557 [taxid 1042877]<br>Expected: Thermococcus [taxid 2263] (genus)<br>Number of reads: 1715<br>Number of identified reads: 1706 (99.475%)	• **genus: 1080 (62.973%)**<br>• family: 31 (1.807%)<br>• order: 1 (0.058%)<br>• class: 43 (2.507%)<br>• phylum: 5 (0.291%)<br>• superkingdom: 14 (0.816%)<br>• root: 504 (29.387%)	• Thermococcus celer [taxid 2264]: 3 (0.174%)<br>• Thermococcus gammatolerans [taxid 187878]: 2 (0.116%)<br>• Thermococci archaeon [taxid 2250254]: 2 (0.116%)<br>• Thermococcus cleftensis [taxid 163003]: 1 (0.058%)<br>• Thermococcus barophilus [taxid 55802]: 1 (0.058%)<br>• Thermococcus barossii [taxid 54077]: 1 (0.058%)<br>• Thermococcus onnurineus [taxid 342948]: 1 (0.058%)<br>• other: 7 (0.408%)

Operational Taxonomic Unit (OTU)	Correct identifications	Wrong or overspecific identifications at species rank
Benchmark OTU ID: `CP002952-_Euryarchaeota`<br>OTU taxon: Thermococcus sp. AM4 [taxid 246969]<br>Expected: Thermococcus [taxid 2263] (genus)<br>Number of reads: 1794<br>Number of identified reads: 1780 (99.219%)	• **genus: 1071 (59.698%)**<br>• family: 35 (1.95%)<br>• order: 2 (0.111%)<br>• class: 42 (2.341%)<br>• phylum: 6 (0.334%)<br>• superkingdom: 19 (1.059%)<br>• root: 573 (31.939%)	• Thermococcus gammatolerans [taxid 187878]: 14 (0.78%)<br>• Thermococcus cleftensis [taxid 163003]: 2 (0.111%)<br>• Thermococcus profundus [taxid 49899]: 2 (0.111%)<br>• Thermococcus nautili [taxid 195522]: 2 (0.111%)<br>• Thermococcus guaymasensis [taxid 110164]: 2 (0.111%)<br>• Thermococcus thioreducens [taxid 277988]: 2 (0.111%)<br>• Thermococcus chitonophagus [taxid 54262]: 1 (0.055%)<br>• other: 8 (0.445%)
Benchmark OTU ID: `CP001147-_Nitrospira`<br>OTU taxon: Thermodesulfovibrio yellowstonii DSM 11347 [taxid 289376]<br>Expected: Thermodesulfovibrio yellowstonii [taxid 28262] (species)<br>Number of reads: 16549<br>Number of identified reads: 16365 (98.888%)	• **species: 11313 (68.36%)**<br>• genus: 1263 (7.631%)<br>• family: 25 (0.151%)<br>• order: 1 (0.006%)<br>• class: 3 (0.018%)<br>• phylum: 65 (0.392%)<br>• superkingdom: 1956 (11.819%)<br>• root: 1724 (10.417%)	• Thermodesulfovibrio aggregans [taxid 86166]: 50 (0.302%)<br>• Leptospira inadai [taxid 29506]: 2 (0.012%)<br>• Finegoldia magna [taxid 1260]: 2 (0.012%)<br>• Chlorobium phaeobacteroides [taxid 1096]: 1 (0.006%)<br>• Helicobacter pylori [taxid 210]: 1 (0.006%)<br>• Yoonia litorea [taxid 1123755]: 1 (0.006%)<br>• Yersinia pestis [taxid 632]: 1 (0.006%)<br>• Sphingobacterium spiritivorum [taxid 258]: 1 (0.006%)<br>• other: 37 (0.223%)
Benchmark OTU ID: `CP000505-_Crenarchaeota`<br>OTU taxon: Thermofilum pendens Hrk 5 [taxid 368408]<br>Expected: Thermofilum pendens [taxid 2269] (species)<br>Number of reads: 3580<br>Number of identified reads: 3491 (97.513%)	• **species: 2117 (59.134%)**<br>• genus: 80 (2.234%)<br>• family: 0 (0.0%)<br>• order: 4 (0.111%)<br>• class: 33 (0.921%)<br>• phylum: 0 (0.0%)<br>• superkingdom: 46 (1.284%)<br>• root: 1149 (32.094%)	• Tanacetum cinerariifolium [taxid 118510]: 1 (0.027%)<br>• Aigarchaeota archaeon [taxid 2528703]: 1 (0.027%)<br>• Vicugna pacos [taxid 30538]: 1 (0.027%)<br>• Thermoprotei archaeon [taxid 2250277]: 1 (0.027%)<br>• Micromonas pusilla [taxid 38833]: 1 (0.027%)<br>• Euryarchaeota archaeon [taxid 2026739]: 1 (0.027%)<br>• Macleaya cordata [taxid 56857]: 1 (0.027%)<br>• Brevundimonas abyssalis [taxid 1125965]: 1 (0.027%)<br>• other: 3 (0.083%)
Benchmark OTU ID: `CP003531-_Crenarchaeota`<br>OTU taxon: Thermogladius calderae 1633 [taxid 1184251]<br>Expected: Thermogladius calderae [taxid 1200300] (species)<br>Number of reads: 2516<br>Number of identified reads: 2461 (97.813%)	• **species: 1532 (60.89%)**<br>• genus: 40 (1.589%)<br>• family: 24 (0.953%)<br>• order: 4 (0.158%)<br>• class: 22 (0.874%)<br>• phylum: 0 (0.0%)<br>• superkingdom: 20 (0.794%)<br>• root: 778 (30.922%)	• Desulfurococcus amylolyticus [taxid 94694]: 1 (0.039%)<br>• Vulcanisaeta moutnovskia [taxid 985052]: 1 (0.039%)<br>• Fomitopsis pinicola [taxid 40483]: 1 (0.039%)

Operational Taxonomic Unit (OTU)	Correct identifications	Wrong or overspecific identifications at species rank
Benchmark OTU ID: `CP001275-_Chloroflexi`<br>OTU taxon: Thermomicrobium roseum DSM 5159 [taxid 309801]<br>Expected: Thermomicrobium roseum [taxid 500] (species)<br>Number of reads: 43582<br>Number of identified reads: 43212 (99.151%)	• **species: 23647 (54.258%)**<br>• genus: 0 (0.0%)<br>• family: 0 (0.0%)<br>• order: 9 (0.02%)<br>• class: 112 (0.256%)<br>• phylum: 318 (0.729%)<br>• superkingdom: 10113 (23.204%)<br>• root: 8947 (20.529%)	• Sphaerobacter thermophilus [taxid 2057]: 6 (0.013%)<br>• bacterium [taxid 1869227]: 4 (0.009%)<br>• Electrophorus electricus [taxid 8005]: 4 (0.009%)<br>• Nitrolancea hollandica [taxid 1206749]: 3 (0.006%)<br>• Polarella glacialis [taxid 89957]: 2 (0.004%)<br>• Candidatus Ozemobacter sibiricus [taxid 2268124]: 2 (0.004%)<br>• Desulfobacca acetoxidans [taxid 60893]: 2 (0.004%)<br>• Camellia sinensis [taxid 4442]: 2 (0.004%)<br>• other: 164 (0.376%)
Benchmark OTU ID: `CP001738-_Actinobacteria`<br>OTU taxon: Thermomonospora curvata DSM 43183 [taxid 471852]<br>Expected: Thermomonospora curvata [taxid 2020] (species)<br>Number of reads: 25228<br>Number of identified reads: 25033 (99.227%)	• **species: 15046 (59.64%)**<br>• genus: 649 (2.572%)<br>• family: 1255 (4.974%)<br>• order: 428 (1.696%)<br>• class: 3122 (12.375%)<br>• phylum: 43 (0.17%)<br>• superkingdom: 1672 (6.627%)<br>• root: 2787 (11.047%)	• Thermomonospora echinospora [taxid 1992]: 16 (0.063%)<br>• Actinomadura craniellae [taxid 2231787]: 8 (0.031%)<br>• Thermomonospora umbrina [taxid 111806]: 7 (0.027%)<br>• Actinomadura harenae [taxid 2483351]: 3 (0.011%)<br>• Actinomadura viridilutea [taxid 58112]: 3 (0.011%)<br>• Trebonia kvetii [taxid 2480626]: 2 (0.007%)<br>• Corynebacterium halotolerans [taxid 225326]: 2 (0.007%)<br>• Streptomyces regalis [taxid 68262]: 2 (0.007%)<br>• other: 81 (0.321%)
Benchmark OTU ID: `CP002916-_Euryarchaeota`<br>OTU taxon: Thermoplasmatales archaeon BRNA1 [taxid 1054217]<br>Expected: Thermoplasmatales [taxid 2301] (order)<br>Number of reads: 1137<br>Number of identified reads: 1137 (100.0%)	• **order: 796 (70.008%)**<br>• class: 18 (1.583%)<br>• phylum: 0 (0.0%)<br>• superkingdom: 25 (2.198%)<br>• root: 286 (25.153%)	• Candidatus Bathyarchaeota archaeon [taxid 2026714]: 1 (0.087%)
Benchmark OTU ID: `FN869859-_Crenarchaeota`<br>OTU taxon: Thermoproteus tenax Kra 1 [taxid 768679]<br>Expected: Thermoproteus tenax [taxid 2271] (species)<br>Number of reads: 3729<br>Number of identified reads: 3647 (97.801%)	• **species: 2066 (55.403%)**<br>• genus: 87 (2.333%)<br>• family: 114 (3.057%)<br>• order: 0 (0.0%)<br>• class: 19 (0.509%)<br>• phylum: 0 (0.0%)<br>• superkingdom: 20 (0.536%)<br>• root: 1262 (33.842%)	• Pyrobaculum neutrophilum [taxid 70771]: 2 (0.053%)<br>• Marchantia polymorpha [taxid 3197]: 1 (0.026%)<br>• Porphyromonas gulae [taxid 111105]: 1 (0.026%)<br>• Candidatus Acetothermum autotrophicum [taxid 1446466]: 1 (0.026%)<br>• Pyrobaculum ferrireducens [taxid 1104324]: 1 (0.026%)<br>• Nocardia otitidiscaviarum [taxid 1823]: 1 (0.026%)
Benchmark OTU ID: `CP002590-_Crenarchaeota`<br>OTU taxon: Thermoproteus uzoniensis 768-20 [taxid 999630]<br>Expected: Thermoproteus uzoniensis [taxid 184117] (species)<br>Number of reads: 3965<br>Number of identified reads: 3889 (98.083%)	• **species: 2058 (51.904%)**<br>• genus: 315 (7.944%)<br>• family: 88 (2.219%)<br>• order: 1 (0.025%)<br>• class: 27 (0.68%)<br>• phylum: 0 (0.0%)<br>• superkingdom: 32 (0.807%)<br>• root: 1291 (32.559%)	• Pyrobaculum ferrireducens [taxid 1104324]: 3 (0.075%)<br>• Pyrobaculum aerophilum [taxid 13773]: 1 (0.025%)<br>• Desulfurococcaceae archaeon [taxid 2184738]: 1 (0.025%)<br>• Exidia glandulosa [taxid 5219]: 1 (0.025%)<br>• Pyrobaculum calidifontis [taxid 181486]: 1 (0.025%)<br>• Hucho hucho [taxid 62062]: 1 (0.025%)<br>• Stentor coeruleus [taxid 5963]: 1 (0.025%)<br>• Thermoproteus tenax [taxid 2271]: 1 (0.025%)<br>• other: 2 (0.05%)

Operational Taxonomic Unit (OTU)	Correct identifications	Wrong or overspecific identifications at species rank
Benchmark OTU ID: `CP001939-_Crenarchaeota`<br>OTU taxon: Thermosphaera aggregans DSM 11486 [taxid 633148]<br>Expected: Thermosphaera aggregans [taxid 54254] (species)<br>Number of reads: 2416<br>Number of identified reads: 2380 (98.509%)	• **species: 1621 (67.094%)**<br>• genus: 0 (0.0%)<br>• family: 32 (1.324%)<br>• order: 0 (0.0%)<br>• class: 25 (1.034%)<br>• phylum: 0 (0.0%)<br>• superkingdom: 21 (0.869%)<br>• root: 652 (26.986%)	• Thermoprotei archaeon [taxid 2250277]: 1 (0.041%)<br>• Pyrococcus horikoshii [taxid 53953]: 1 (0.041%)<br>• Fragilariopsis kerguelensis [taxid 186038]: 1 (0.041%)<br>• Nanoarchaeota archaeon [taxid 2026764]: 1 (0.041%)<br>• Musca domestica [taxid 7370]: 1 (0.041%)<br>• Thermogladius calderae [taxid 1200300]: 1 (0.041%)
Benchmark OTU ID: `BA000039-_Cyanobacteria`<br>OTU taxon: Thermosynechococcus elongatus BP-1 [taxid 197221]<br>Expected: Thermosynechococcus elongatus [taxid 146786] (species)<br>Number of reads: 13259<br>Number of identified reads: 13163 (99.275%)	• **species: 5142 (38.781%)**<br>• genus: 806 (6.078%)<br>• family: 293 (2.209%)<br>• order: 61 (0.46%)<br>• phylum: 3657 (27.581%)<br>• superkingdom: 1124 (8.477%)<br>• root: 2036 (15.355%)	• Synechococcus lividus [taxid 33070]: 12 (0.09%)<br>• Synechococcus elongatus [taxid 32046]: 3 (0.022%)<br>• Heliobacterium modesticaldum [taxid 35701]: 2 (0.015%)<br>• Thermosynechococcus vulcanus [taxid 32053]: 2 (0.015%)<br>• Solanum chacoense [taxid 4108]: 1 (0.007%)<br>• Lampropedia hyalina [taxid 198706]: 1 (0.007%)<br>• Octadecabacter arcticus [taxid 53946]: 1 (0.007%)<br>• other: 33 (0.248%)
Benchmark OTU ID: `CP001905-_Proteobacteria`<br>OTU taxon: Thioalkalivibrio sp. K90mix [taxid 396595]<br>Expected: Thioalkalivibrio [taxid 106633] (genus)<br>Number of reads: 5292<br>Number of identified reads: 5272 (99.622%)	• **genus: 3900 (73.696%)**<br>• family: 24 (0.453%)<br>• order: 20 (0.377%)<br>• class: 292 (5.517%)<br>• phylum: 284 (5.366%)<br>• superkingdom: 305 (5.763%)<br>• root: 442 (8.352%)	• Thioalkalivibrio versutus [taxid 106634]: 25 (0.472%)<br>• Thioalkalivibrio nitratireducens [taxid 186931]: 3 (0.056%)<br>• Thioflavicoccus mobilis [taxid 80679]: 1 (0.018%)<br>• Klebsiella oxytoca [taxid 571]: 1 (0.018%)<br>• Lingulodinium polyedra [taxid 160621]: 1 (0.018%)<br>• Stenotrophomonas maltophilia [taxid 40324]: 1 (0.018%)<br>• Granulosicoccus antarcticus [taxid 437505]: 1 (0.018%)<br>• other: 13 (0.245%)
Benchmark OTU ID: `CP002776-_Proteobacteria`<br>OTU taxon: Thiomicrospira cyclica ALM1 [taxid 717773]<br>Expected: Thiomicrospira cyclica [taxid 147268] (species)<br>Number of reads: 3464<br>Number of identified reads: 3452 (99.653%)	• **species: 2215 (63.943%)**<br>• genus: 412 (11.893%)<br>• family: 35 (1.01%)<br>• order: 2 (0.057%)<br>• class: 194 (5.6%)<br>• phylum: 138 (3.983%)<br>• superkingdom: 154 (4.445%)<br>• root: 302 (8.718%)	• Thiomicrospira aerophila [taxid 92245]: 6 (0.173%)<br>• Prosthecomicrobium hirschii [taxid 665126]: 1 (0.028%)<br>• Candidatus Thiomargarita nelsonii [taxid 1003181]: 1 (0.028%)<br>• gamma proteobacterium symbiont of Ctena orbiculata [taxid 1968598]: 1 (0.028%)<br>• Thiosulfatimonas sediminis [taxid 2675054]: 1 (0.028%)<br>• Candidatus Pantoea carbekii [taxid 1235990]: 1 (0.028%)<br>• Lactobacillus iners [taxid 147802]: 1 (0.028%)<br>• Pseudomonas protegens [taxid 380021]: 1 (0.028%)
Benchmark OTU ID: `FP475956-_Proteobacteria`<br>OTU taxon: Thiomonas arsenitoxydans [taxid 426114]<br>Expected: Thiomonas arsenitoxydans [taxid 426114] (species)<br>Number of reads: 7510<br>Number of identified reads: 7490 (99.733%)	• species: 1088 (14.487%)<br>• **genus: 3683 (49.041%)**<br>• order: 733 (9.76%)<br>• class: 153 (2.037%)<br>• phylum: 645 (8.588%)<br>• superkingdom: 409 (5.446%)<br>• root: 770 (10.252%)	• Thiomonas intermedia [taxid 926]: 12 (0.159%)<br>• Thiomonas bhubaneswarensis [taxid 339866]: 10 (0.133%)<br>• Thiomonas delicata [taxid 364030]: 3 (0.039%)<br>• Serratia symbiotica [taxid 138074]: 1 (0.013%)<br>• Brenneria alni [taxid 71656]: 1 (0.013%)<br>• Streptoalloteichus hindustanus [taxid 2017]: 1 (0.013%)<br>• Caballeronia sordidicola [taxid 196367]: 1 (0.013%)<br>• other: 26 (0.346%)

Operational Taxonomic Unit (OTU)	Correct identifications	Wrong or overspecific identifications at species rank
Benchmark OTU ID: `CP000393-_Cyanobacteria`<br>OTU taxon: Trichodesmium erythraeum IMS101 [taxid 203124]<br>Expected: Trichodesmium erythraeum [taxid 1206] (species)<br>Number of reads: 47291<br>Number of identified reads: 44268 (93.607%)	• **species: 23820 (50.368%)**<br>• genus: 39 (0.082%)<br>• family: 383 (0.809%)<br>• order: 3472 (7.341%)<br>• phylum: 2303 (4.869%)<br>• superkingdom: 1939 (4.1%)<br>• root: 12227 (25.854%)	• Nostoc flagelliforme [taxid 1306274]: 13 (0.027%)<br>• Crocosphaera watsonii [taxid 263511]: 10 (0.021%)<br>• Lyngbya aestuarii [taxid 118322]: 5 (0.01%)<br>• Moorea sp. SIOASIH [taxid 2607817]: 4 (0.008%)<br>• Microcystis aeruginosa [taxid 1126]: 4 (0.008%)<br>• Oscillatoria acuminata [taxid 118323]: 4 (0.008%)<br>• Moorea producens [taxid 1155739]: 4 (0.008%)<br>• other: 132 (0.279%)
Benchmark OTU ID: `CP000117-_Cyanobacteria`<br>OTU taxon: Trichormus variabilis ATCC 29413 [taxid 240292]<br>Expected: Trichormus variabilis [taxid 264691] (species)<br>Number of reads: 38154<br>Number of identified reads: 37605 (98.561%)	• species: 394 (1.032%)<br>• genus: 20 (0.052%)<br>• **family: 23083 (60.499%)**<br>• order: 4261 (11.167%)<br>• phylum: 2785 (7.299%)<br>• superkingdom: 1894 (4.964%)<br>• root: 5102 (13.372%)	• Trichormus azollae [taxid 1164]: 20 (0.052%)<br>• Nostoc minutum [taxid 1841509]: 19 (0.049%)<br>• Cylindrospermum stagnale [taxid 142864]: 16 (0.041%)<br>• Nostoc linckia [taxid 92942]: 16 (0.041%)<br>• Anabaena cylindrica [taxid 1165]: 15 (0.039%)<br>• Nostoc sphaeroides [taxid 446679]: 9 (0.023%)<br>• Nostoc punctiforme [taxid 272131]: 8 (0.02%)<br>• other: 182 (0.477%)
Benchmark OTU ID: `CP002417-_Proteobacteria`<br>OTU taxon: Variovorax paradoxus EPS [taxid 595537]<br>Expected: Variovorax paradoxus [taxid 34073] (species)<br>Number of reads: 13854<br>Number of identified reads: 13817 (99.732%)	• **species: 4590 (33.131%)**<br>• genus: 4530 (32.698%)<br>• family: 1304 (9.412%)<br>• order: 948 (6.842%)<br>• class: 96 (0.692%)<br>• phylum: 778 (5.615%)<br>• superkingdom: 566 (4.085%)<br>• root: 987 (7.124%)	• Variovorax guangxiensis [taxid 1775474]: 6 (0.043%)<br>• Variovorax boronicumulans [taxid 436515]: 5 (0.036%)<br>• Curvibacter putative symbiont of Hydra magnipapillata [taxid 667019]: 3 (0.021%)<br>• Hydrogenophaga crassostreae [taxid 1763535]: 2 (0.014%)<br>• Burkholderia pseudomallei [taxid 28450]: 2 (0.014%)<br>• Brugia timori [taxid 42155]: 2 (0.014%)<br>• Comamonas terrigena [taxid 32013]: 2 (0.014%)<br>• Terricaulis silvestris [taxid 2686094]: 1 (0.007%)<br>• other: 51 (0.368%)
Benchmark OTU ID: `CP001635-_Proteobacteria`<br>OTU taxon: Variovorax paradoxus S110 [taxid 543728]<br>Expected: Variovorax paradoxus [taxid 34073] (species)<br>Number of reads: 11775<br>Number of identified reads: 11750 (99.787%)	• **species: 3938 (33.443%)**<br>• genus: 3676 (31.218%)<br>• family: 1173 (9.961%)<br>• order: 945 (8.025%)<br>• class: 109 (0.925%)<br>• phylum: 638 (5.418%)<br>• superkingdom: 432 (3.668%)<br>• root: 832 (7.065%)	• Variovorax boronicumulans [taxid 436515]: 5 (0.042%)<br>• Ramlibacter tataouinensis [taxid 94132]: 2 (0.016%)<br>• Variovorax guangxiensis [taxid 1775474]: 2 (0.016%)<br>• Rhodoferax koreense [taxid 1842727]: 2 (0.016%)<br>• Burkholderia ambifaria [taxid 152480]: 1 (0.008%)<br>• Salmonella enterica [taxid 28901]: 1 (0.008%)<br>• Pseudomonas aeruginosa [taxid 287]: 1 (0.008%)<br>• Rhodoferax ferrireducens [taxid 192843]: 1 (0.008%)<br>• other: 32 (0.271%)

Operational Taxonomic Unit (OTU)	Correct identifications	Wrong or overspecific identifications at species rank
Benchmark OTU ID: CP001636-_Proteobacteria<br>OTU taxon: Variovorax paradoxus S110 [taxid 543728]<br>Expected: Variovorax paradoxus [taxid 34073] (species)<br>Number of reads: 1655<br>Number of identified reads: 1648 (99.577%)	• **species: 824 (49.788%)**<br>• genus: 278 (16.797%)<br>• family: 74 (4.471%)<br>• order: 102 (6.163%)<br>• class: 13 (0.785%)<br>• phylum: 123 (7.432%)<br>• superkingdom: 69 (4.169%)<br>• root: 163 (9.848%)	• Pandoraea terrigena [taxid 2508292]: 1 (0.06%)
Benchmark OTU ID: CP000542-_Proteobacteria<br>OTU taxon: Verminephrobacter eiseniae EF01-2 [taxid 391735]<br>Expected: Verminephrobacter eiseniae [taxid 364317] (species)<br>Number of reads: 11641<br>Number of identified reads: 11612 (99.75%)	• species: 3259 (27.995%)<br>• **genus: 4097 (35.194%)**<br>• family: 685 (5.884%)<br>• order: 936 (8.04%)<br>• class: 146 (1.254%)<br>• phylum: 848 (7.284%)<br>• superkingdom: 559 (4.801%)<br>• root: 1072 (9.208%)	• Simplicispira suum [taxid 2109915]: 2 (0.017%)<br>• Cupriavidus taiwanensis [taxid 164546]: 2 (0.017%)<br>• Curvibacter putative symbiont of Hydra magnipapillata [taxid 667019]: 2 (0.017%)<br>• Mola mola [taxid 94237]: 1 (0.008%)<br>• Rugamonas rubra [taxid 758825]: 1 (0.008%)<br>• Acidovorax temperans [taxid 80878]: 1 (0.008%)<br>• Burkholderia contaminans [taxid 488447]: 1 (0.008%)<br>• Roseivivax pacificus [taxid 1267769]: 1 (0.008%)<br>• other: 45 (0.386%)
Benchmark OTU ID: FM954972-_Pathogens<br>OTU taxon: Vibrio atlanticus [taxid 693153]<br>Expected: Vibrio atlanticus [taxid 693153] (species)<br>Number of reads: 7034<br>Number of identified reads: 6993 (99.417%)	• species: 541 (7.691%)<br>• **genus: 4740 (67.386%)**<br>• family: 380 (5.402%)<br>• order: 19 (0.27%)<br>• class: 479 (6.809%)<br>• phylum: 111 (1.578%)<br>• superkingdom: 171 (2.431%)<br>• root: 549 (7.804%)	• Vibrio splendidus [taxid 29497]: 21 (0.298%)<br>• Vibrio tasmaniensis [taxid 212663]: 15 (0.213%)<br>• Vibrio cholerae [taxid 666]: 8 (0.113%)<br>• Vibrio alginolyticus [taxid 663]: 7 (0.099%)<br>• Vibrio sp. MedPE-SWchi [taxid 1860091]: 5 (0.071%)<br>• Vibrio campbellii [taxid 680]: 4 (0.056%)<br>• Vibrio chagasii [taxid 170679]: 4 (0.056%)<br>• Vibrio sinensis [taxid 2302434]: 4 (0.056%)<br>• other: 81 (1.151%)
Benchmark OTU ID: CP002555-_Pathogens<br>OTU taxon: Vibrio cholerae LMA3984-4 [taxid 935297]<br>Expected: Vibrio cholerae [taxid 666] (species)<br>Number of reads: 5765<br>Number of identified reads: 5744 (99.635%)	• species: 1061 (18.404%)<br>• **genus: 3298 (57.207%)**<br>• family: 243 (4.215%)<br>• order: 2 (0.034%)<br>• class: 482 (8.36%)<br>• phylum: 113 (1.96%)<br>• superkingdom: 144 (2.497%)<br>• root: 399 (6.921%)	• Vibrio metoecus [taxid 1481663]: 14 (0.242%)<br>• Vibrio mimicus [taxid 674]: 10 (0.173%)<br>• Salmonella enterica [taxid 28901]: 5 (0.086%)<br>• Escherichia coli [taxid 562]: 4 (0.069%)<br>• Vibrio vulnificus [taxid 672]: 3 (0.052%)<br>• Vibrio scophthalmi [taxid 45658]: 3 (0.052%)<br>• Vibrio parahaemolyticus [taxid 670]: 3 (0.052%)<br>• Vibrio mediterranei [taxid 689]: 2 (0.034%)<br>• other: 47 (0.815%)

Operational Taxonomic Unit (OTU)	Correct identifications	Wrong or overspecific identifications at species rank
Benchmark OTU ID: `CP003069-_Pathogens`<br>OTU taxon: Vibrio cholerae O1 str. 2010EL-1786 [taxid 914149]<br>Expected: Vibrio cholerae [taxid 666] (species)<br>Number of reads: 6364<br>Number of identified reads: 6342 (99.654%)	• species: 1394 (21.904%)<br>• **genus: 3429 (53.881%)**<br>• family: 230 (3.614%)<br>• order: 0 (0.0%)<br>• class: 580 (9.113%)<br>• phylum: 90 (1.414%)<br>• superkingdom: 178 (2.796%)<br>• root: 436 (6.851%)	• Vibrio metoecus [taxid 1481663]: 10 (0.157%)<br>• Vibrio anguillarum [taxid 55601]: 6 (0.094%)<br>• Vibrio mimicus [taxid 674]: 6 (0.094%)<br>• Vibrio vulnificus [taxid 672]: 4 (0.062%)<br>• Vibrio parahaemolyticus [taxid 670]: 3 (0.047%)<br>• Vibrio alginolyticus [taxid 663]: 3 (0.047%)<br>• Escherichia coli [taxid 562]: 3 (0.047%)<br>• Vibrio viridaestus [taxid 2487322]: 2 (0.031%)<br>• other: 50 (0.785%)
Benchmark OTU ID: `CP001235-_Pathogens`<br>OTU taxon: Vibrio cholerae O395 [taxid 345073]<br>Expected: Vibrio cholerae [taxid 666] (species)<br>Number of reads: 6346<br>Number of identified reads: 6330 (99.747%)	• species: 1529 (24.093%)<br>• **genus: 3338 (52.6%)**<br>• family: 228 (3.592%)<br>• order: 0 (0.0%)<br>• class: 530 (8.351%)<br>• phylum: 106 (1.67%)<br>• superkingdom: 158 (2.489%)<br>• root: 439 (6.917%)	• Vibrio metoecus [taxid 1481663]: 10 (0.157%)<br>• Vibrio mimicus [taxid 674]: 9 (0.141%)<br>• Vibrio campbellii [taxid 680]: 4 (0.063%)<br>• Vibrio diazotrophicus [taxid 685]: 3 (0.047%)<br>• Vibrio vulnificus [taxid 672]: 3 (0.047%)<br>• Salmonella enterica [taxid 28901]: 3 (0.047%)<br>• Vibrio furnissii [taxid 29494]: 2 (0.031%)<br>• Vibrio thalassae [taxid 1243014]: 2 (0.031%)<br>• other: 52 (0.819%)
Benchmark OTU ID: `CP003241-_Proteobacteria`<br>OTU taxon: Vibrio sp. EJY3 [taxid 1116375]<br>Expected: Vibrio [taxid 662] (genus)<br>Number of reads: 6942<br>Number of identified reads: 6902 (99.423%)	• **genus: 4672 (67.3%)**<br>• family: 373 (5.373%)<br>• order: 3 (0.043%)<br>• class: 529 (7.62%)<br>• phylum: 137 (1.973%)<br>• superkingdom: 220 (3.169%)<br>• root: 958 (13.8%)	• **Vibrio sp. dhg [taxid 2163016]: 279 (4.019%)**<br>• **Vibrio natriegens [taxid 691]: 222 (3.197%)**<br>• Vibrio fluvialis [taxid 676]: 47 (0.677%)<br>• Vibrio campbellii [taxid 680]: 17 (0.244%)<br>• Vibrio parahaemolyticus [taxid 670]: 17 (0.244%)<br>• Vibrio splendidus [taxid 29497]: 15 (0.216%)<br>• Vibrio alginolyticus [taxid 663]: 13 (0.187%)<br>• other: 163 (2.348%)
Benchmark OTU ID: `AE016795-_Pathogens`<br>OTU taxon: Vibrio vulnificus CMCP6 [taxid 216895]<br>Expected: Vibrio vulnificus [taxid 672] (species)<br>Number of reads: 6991<br>Number of identified reads: 6961 (99.57%)	• species: 1172 (16.764%)<br>• **genus: 3922 (56.1%)**<br>• family: 355 (5.077%)<br>• order: 5 (0.071%)<br>• class: 533 (7.624%)<br>• phylum: 126 (1.802%)<br>• superkingdom: 198 (2.832%)<br>• root: 647 (9.254%)	• Vibrio fluvialis [taxid 676]: 10 (0.143%)<br>• Vibrio campbellii [taxid 680]: 10 (0.143%)<br>• Vibrio cholerae [taxid 666]: 9 (0.128%)<br>• Vibrio scophthalmi [taxid 45658]: 8 (0.114%)<br>• Vibrio cidicii [taxid 1763883]: 4 (0.057%)<br>• Vibrio alginolyticus [taxid 663]: 4 (0.057%)<br>• Vibrio rotiferianus [taxid 190895]: 3 (0.042%)<br>• Vibrio palustris [taxid 1918946]: 3 (0.042%)<br>• other: 88 (1.258%)

Operational Taxonomic Unit (OTU)	Correct identifications	Wrong or overspecific identifications at species rank
Benchmark OTU ID: `CP002100-_Crenarchaeota`<br>OTU taxon: Vulcanisaeta distributa DSM 14429 [taxid 572478]<br>Expected: Vulcanisaeta distributa [taxid 164451] (species)<br>Number of reads: 5060<br>Number of identified reads: 4951 (97.845%)	• **species: 2851 (56.343%)**<br>• genus: 408 (8.063%)<br>• family: 70 (1.383%)<br>• order: 3 (0.059%)<br>• class: 38 (0.75%)<br>• phylum: 0 (0.0%)<br>• superkingdom: 37 (0.731%)<br>• root: 1482 (29.288%)	• Vulcanisaeta moutnovskia [taxid 985052]: 12 (0.237%)<br>• Desulfurococcales archaeon [taxid 2480821]: 1 (0.019%)<br>• Candidatus Solibacter usitatus [taxid 332163]: 1 (0.019%)<br>• Metallosphaera tengchongensis [taxid 1532350]: 1 (0.019%)<br>• Candidatus Woesearchaeota archaeon [taxid 2026803]: 1 (0.019%)<br>• Minutocellus polymorphus [taxid 265543]: 1 (0.019%)<br>• Alkalicoccus halolimnae [taxid 1667239]: 1 (0.019%)<br>• Candidatus Verstraetearchaeota archaeon [taxid 2250257]: 1 (0.019%)<br>• other: 3 (0.059%)
Benchmark OTU ID: `CP002529-_Crenarchaeota`<br>OTU taxon: Vulcanisaeta moutnovskia 768-28 [taxid 985053]<br>Expected: Vulcanisaeta moutnovskia [taxid 985052] (species)<br>Number of reads: 4872<br>Number of identified reads: 4781 (98.132%)	• **species: 2839 (58.271%)**<br>• genus: 409 (8.394%)<br>• family: 41 (0.841%)<br>• order: 1 (0.02%)<br>• class: 22 (0.451%)<br>• phylum: 1 (0.02%)<br>• superkingdom: 32 (0.656%)<br>• root: 1366 (28.037%)	• Vulcanisaeta distributa [taxid 164451]: 12 (0.246%)<br>• Acidilobales archaeon [taxid 2268176]: 2 (0.041%)<br>• Branchiostoma belcheri [taxid 7741]: 1 (0.02%)<br>• Caldivirga maquilingensis [taxid 76887]: 1 (0.02%)<br>• Biomphalaria glabrata [taxid 6526]: 1 (0.02%)<br>• Schleiferia thermophila [taxid 884107]: 1 (0.02%)<br>• Macrostomum lignano [taxid 282301]: 1 (0.02%)<br>• Thermoprotei archaeon [taxid 2250277]: 1 (0.02%)<br>• other: 2 (0.041%)
Benchmark OTU ID: `CP002455-_Bacteroidetes`<br>OTU taxon: Weeksella virosa DSM 16922 [taxid 865938]<br>Expected: Weeksella virosa [taxid 1014] (species)<br>Number of reads: 13463<br>Number of identified reads: 13346 (99.13%)	• **species: 9525 (70.749%)**<br>• genus: 0 (0.0%)<br>• family: 279 (2.072%)<br>• order: 845 (6.276%)<br>• class: 3 (0.022%)<br>• phylum: 621 (4.612%)<br>• superkingdom: 652 (4.842%)<br>• root: 1393 (10.346%)	• Lupinus albus [taxid 3870]: 11 (0.081%)<br>• Oligella urethralis [taxid 90245]: 6 (0.044%)<br>• Algoriella xinjiangensis [taxid 684065]: 3 (0.022%)<br>• Moheibacter sediminis [taxid 1434700]: 3 (0.022%)<br>• Empedobacter falsenii [taxid 343874]: 2 (0.014%)<br>• Flavobacterium sediminis [taxid 2201181]: 2 (0.014%)<br>• Phocaeicola vulgatus [taxid 821]: 2 (0.014%)<br>• Sphingobacterium wenxiniae [taxid 683125]: 2 (0.014%)<br>• other: 50 (0.371%)
Benchmark OTU ID: `AM999887-_Proteobacteria`<br>OTU taxon: Wolbachia endosymbiont of Culex quinquefasciatus Pel [taxid 570417]<br>Expected: Wolbachia endosymbiont of Culex quinquefasciatus [taxid 263437] (species)<br>Number of reads: 2450<br>Number of identified reads: 2418 (98.693%)	• species: 156 (6.367%)<br>• **genus: 1568 (64.0%)**<br>• family: 8 (0.326%)<br>• order: 6 (0.244%)<br>• class: 33 (1.346%)<br>• phylum: 46 (1.877%)<br>• superkingdom: 116 (4.734%)<br>• root: 479 (19.551%)	• Wolbachia endosymbiont of Culex molestus [taxid 329647]: 14 (0.571%)<br>• Wolbachia endosymbiont of Drosophila mauritiana [taxid 109663]: 9 (0.367%)<br>• Wolbachia pipientis [taxid 955]: 6 (0.244%)<br>• Wolbachia endosymbiont of Cylisticus convexus [taxid 118728]: 5 (0.204%)<br>• Wolbachia endosymbiont of Aleurodicus floccissimus [taxid 2152762]: 4 (0.163%)<br>• Wolbachia endosymbiont of Folsomia candida [taxid 169402]: 4 (0.163%)<br>• Wolbachia endosymbiont of Cimex lectularius [taxid 246273]: 4 (0.163%)<br>• Wolbachia endosymbiont of Wuchereria bancrofti [taxid 96496]: 3 (0.122%)<br>• other: 38 (1.551%)

Operational Taxonomic Unit (OTU)	Correct identifications	Wrong or overspecific identifications at species rank
Benchmark OTU ID: `AE017196-_Proteobacteria`<br>OTU taxon: Wolbachia endosymbiont of Drosophila melanogaster [taxid 163164]<br>Expected: Wolbachia endosymbiont of Drosophila melanogaster [taxid 163164] (species)<br>Number of reads: 1968<br>Number of identified reads: 1934 (98.272%)	• species: 85 (4.319%)<br>• **genus: 1119 (56.859%)**<br>• family: 16 (0.813%)<br>• order: 6 (0.304%)<br>• class: 48 (2.439%)<br>• phylum: 40 (2.032%)<br>• superkingdom: 133 (6.758%)<br>• root: 472 (23.983%)	• Wolbachia pipientis [taxid 955]: 25 (1.27%)<br>• Wolbachia endosymbiont of Drosophila simulans [taxid 77038]: 19 (0.965%)<br>• Wolbachia endosymbiont of Drosophila ananassae [taxid 307502]: 13 (0.66%)<br>• Glossina austeni [taxid 7395]: 6 (0.304%)<br>• Wolbachia endosymbiont of Dactylopius coccus [taxid 1605993]: 5 (0.254%)<br>• Wolbachia endosymbiont of Onchocerca ochengi [taxid 100901]: 3 (0.152%)<br>• Wolbachia endosymbiont of Cimex lectularius [taxid 246273]: 2 (0.101%)<br>• Wolbachia endosymbiont of Wuchereria bancrofti [taxid 96496]: 2 (0.101%)<br>• other: 23 (1.168%)
Benchmark OTU ID: `CP003884-_Proteobacteria`<br>OTU taxon: Wolbachia endosymbiont of Drosophila simulans wHa [taxid 1236909]<br>Expected: Wolbachia endosymbiont of Drosophila simulans [taxid 77038] (species)<br>Number of reads: 2032<br>Number of identified reads: 1992 (98.031%)	• species: 90 (4.429%)<br>• **genus: 1211 (59.596%)**<br>• family: 7 (0.344%)<br>• order: 2 (0.098%)<br>• class: 39 (1.919%)<br>• phylum: 46 (2.263%)<br>• superkingdom: 134 (6.594%)<br>• root: 457 (22.49%)	• Wolbachia pipientis [taxid 955]: 28 (1.377%)<br>• Wolbachia endosymbiont of Drosophila ananassae [taxid 307502]: 20 (0.984%)<br>• Wolbachia endosymbiont of Drosophila melanogaster [taxid 163164]: 12 (0.59%)<br>• Wolbachia endosymbiont of Carposina sasakii [taxid 2591635]: 9 (0.442%)<br>• Wolbachia endosymbiont of Dactylopius coccus [taxid 1605993]: 7 (0.344%)<br>• Wolbachia endosymbiont of Onchocerca ochengi [taxid 100901]: 3 (0.147%)<br>• Wolbachia endosymbiont of Folsomia candida [taxid 169402]: 3 (0.147%)<br>• Wolbachia endosymbiont of Glossina morsitans morsitans [taxid 1150948]: 3 (0.147%)<br>• other: 25 (1.23%)

Operational Taxonomic Unit (OTU)	Correct identifications	Wrong or overspecific identifications at species rank
Benchmark OTU ID: `CP003883-_Proteobacteria`<br>OTU taxon: Wolbachia endosymbiont of Drosophila simulans wNo [taxid 1236908]<br>Expected: Wolbachia endosymbiont of Drosophila simulans [taxid 77038] (species)<br>Number of reads: 2045<br>Number of identified reads: 2022 (98.875%)	• species: 117 (5.721%)<br>• **genus: 1324 (64.743%)**<br>• family: 7 (0.342%)<br>• order: 2 (0.097%)<br>• class: 44 (2.151%)<br>• phylum: 41 (2.004%)<br>• superkingdom: 118 (5.77%)<br>• root: 359 (17.555%)	• Wolbachia endosymbiont of Drosophila mauritiana [taxid 109663]: 32 (1.564%)<br>• Wolbachia pipientis [taxid 955]: 7 (0.342%)<br>• Glossina austeni [taxid 7395]: 5 (0.244%)<br>• Wolbachia endosymbiont of Culex molestus [taxid 329647]: 5 (0.244%)<br>• Wolbachia endosymbiont of Diaphorina citri [taxid 116598]: 5 (0.244%)<br>• Wolbachia endosymbiont of Cylisticus convexus [taxid 118728]: 4 (0.195%)<br>• Wolbachia endosymbiont of Bemisia tabaci [taxid 215173]: 4 (0.195%)<br>• Wolbachia endosymbiont of Drosophila ananassae [taxid 307502]: 4 (0.195%)<br>• other: 22 (1.075%)
Benchmark OTU ID: `AE017321-_Proteobacteria`<br>OTU taxon: Wolbachia endosymbiont strain TRS of Brugia malayi [taxid 292805]<br>Expected: Wolbachia endosymbiont of Brugia malayi [taxid 80849] (species)<br>Number of reads: 1546<br>Number of identified reads: 1502 (97.153%)	• species: 101 (6.532%)<br>• **genus: 885 (57.244%)**<br>• family: 14 (0.905%)<br>• order: 5 (0.323%)<br>• class: 19 (1.228%)<br>• phylum: 30 (1.94%)<br>• superkingdom: 58 (3.751%)<br>• root: 382 (24.708%)	• **Wolbachia endosymbiont of Brugia pahangi [taxid 96495]: 64 (4.139%)**<br>• Wolbachia endosymbiont of Wuchereria bancrofti [taxid 96496]: 22 (1.423%)<br>• Wolbachia endosymbiont of Folsomia candida [taxid 169402]: 4 (0.258%)<br>• Glossina austeni [taxid 7395]: 4 (0.258%)<br>• Wolbachia endosymbiont of Onchocerca ochengi [taxid 100901]: 2 (0.129%)<br>• Armadillidium vulgare [taxid 13347]: 2 (0.129%)<br>• Wolbachia endosymbiont of Drosophila ananassae [taxid 307502]: 1 (0.064%)<br>• Wolbachia endosymbiont of Cimex lectularius [taxid 246273]: 1 (0.064%)<br>• other: 13 (0.84%)
Benchmark OTU ID: `CP002914-_Proteobacteria`<br>OTU taxon: Xanthomonas axonopodis pv. citrumelo F1 [taxid 981368]<br>Expected: Xanthomonas euvesicatoria [taxid 456327] (species)<br>Number of reads: 10293<br>Number of identified reads: 10255 (99.63%)	• species: 460 (4.469%)<br>• **genus: 7309 (71.009%)**<br>• family: 731 (7.101%)<br>• order: 51 (0.495%)<br>• class: 163 (1.583%)<br>• phylum: 379 (3.682%)<br>• superkingdom: 324 (3.147%)<br>• root: 828 (8.044%)	• Xanthomonas citri [taxid 346]: 96 (0.932%)<br>• Xanthomonas oryzae [taxid 347]: 75 (0.728%)<br>• Xanthomonas hortorum [taxid 56454]: 30 (0.291%)<br>• Xanthomonas axonopodis [taxid 53413]: 29 (0.281%)<br>• Xanthomonas arboricola [taxid 56448]: 29 (0.281%)<br>• Xanthomonas phaseoli [taxid 1985254]: 28 (0.272%)<br>• Xanthomonas vasicola [taxid 56459]: 17 (0.165%)<br>• Xanthomonas campestris [taxid 339]: 15 (0.145%)<br>• other: 123 (1.194%)

Operational Taxonomic Unit (OTU)	Correct identifications	Wrong or overspecific identifications at species rank
Benchmark OTU ID: `AE003849-_Proteobacteria`<br>OTU taxon: Xylella fastidiosa 9a5c [taxid 160492]<br>Expected: Xylella fastidiosa [taxid 2371] (species)<br>Number of reads: 4988<br>Number of identified reads: 4962 (99.478%)	• **species: 3115 (62.449%)**<br>• genus: 222 (4.45%)<br>• family: 320 (6.415%)<br>• order: 38 (0.761%)<br>• class: 96 (1.924%)<br>• phylum: 208 (4.17%)<br>• superkingdom: 174 (3.488%)<br>• root: 784 (15.717%)	• Xylella taiwanensis [taxid 1444770]: 2 (0.04%)<br>• Bordetella ansorpii [taxid 288768]: 1 (0.02%)<br>• Pararhodospirillum photometricum [taxid 1084]: 1 (0.02%)<br>• Yersinia rohdei [taxid 29485]: 1 (0.02%)<br>• Sphingobium barthaii [taxid 1054037]: 1 (0.02%)<br>• Castellaniella defragrans [taxid 75697]: 1 (0.02%)<br>• Xanthomonas citri [taxid 346]: 1 (0.02%)<br>• Brenneria rubrifaciens [taxid 55213]: 1 (0.02%)<br>• other: 11 (0.22%)
Benchmark OTU ID: `CP000941-_Proteobacteria`<br>OTU taxon: Xylella fastidiosa M12 [taxid 405440]<br>Expected: Xylella fastidiosa [taxid 2371] (species)<br>Number of reads: 4685<br>Number of identified reads: 4659 (99.445%)	• **species: 2914 (62.198%)**<br>• genus: 192 (4.098%)<br>• family: 293 (6.254%)<br>• order: 28 (0.597%)<br>• class: 107 (2.283%)<br>• phylum: 147 (3.137%)<br>• superkingdom: 182 (3.884%)<br>• root: 790 (16.862%)	• Xylella taiwanensis [taxid 1444770]: 2 (0.042%)<br>• Marinobacterium stanieri [taxid 49186]: 1 (0.021%)<br>• Luteimonas wenzhouensis [taxid 2599615]: 1 (0.021%)<br>• Tolypothrix campylonemoides [taxid 1136105]: 1 (0.021%)<br>• Humitalea rosea [taxid 990373]: 1 (0.021%)<br>• Pasteurella multocida [taxid 747]: 1 (0.021%)<br>• Lysobacter soli [taxid 453783]: 1 (0.021%)<br>• gamma proteobacterium symbiont of Ctena orbiculata [taxid 1968598]: 1 (0.021%)<br>• other: 11 (0.234%)
Benchmark OTU ID: `AE009442-_Proteobacteria`<br>OTU taxon: Xylella fastidiosa Temecula1 [taxid 183190]<br>Expected: Xylella fastidiosa [taxid 2371] (species)<br>Number of reads: 4786<br>Number of identified reads: 4760 (99.456%)	• **species: 2979 (62.244%)**<br>• genus: 245 (5.119%)<br>• family: 277 (5.787%)<br>• order: 40 (0.835%)<br>• class: 102 (2.131%)<br>• phylum: 159 (3.322%)<br>• superkingdom: 173 (3.614%)<br>• root: 775 (16.193%)	• Xylella taiwanensis [taxid 1444770]: 5 (0.104%)<br>• Brugia timori [taxid 42155]: 2 (0.041%)<br>• bacterium [taxid 1869227]: 1 (0.02%)<br>• Xanthomonas hyacinthi [taxid 56455]: 1 (0.02%)<br>• Lates calcarifer [taxid 8187]: 1 (0.02%)<br>• Hanseniaspora guilliermondii [taxid 56406]: 1 (0.02%)<br>• Xanthomonas citri [taxid 346]: 1 (0.02%)<br>• Salmonella enterica [taxid 28901]: 1 (0.02%)<br>• other: 13 (0.271%)
Benchmark OTU ID: `CP002165-_Proteobacteria`<br>OTU taxon: Xylella fastidiosa subsp. fastidiosa GB514 [taxid 788929]<br>Expected: Xylella fastidiosa [taxid 2371] (species)<br>Number of reads: 4721<br>Number of identified reads: 4692 (99.385%)	• **species: 2950 (62.486%)**<br>• genus: 221 (4.681%)<br>• family: 293 (6.206%)<br>• order: 41 (0.868%)<br>• class: 85 (1.8%)<br>• phylum: 139 (2.944%)<br>• superkingdom: 164 (3.473%)<br>• root: 793 (16.797%)	• Xylella taiwanensis [taxid 1444770]: 4 (0.084%)<br>• Brugia timori [taxid 42155]: 2 (0.042%)<br>• Bacteroides fragilis [taxid 817]: 1 (0.021%)<br>• Pulveribacter suum [taxid 2116657]: 1 (0.021%)<br>• Caballeronia temeraria [taxid 1777137]: 1 (0.021%)<br>• Pseudoxanthomonas indica [taxid 428993]: 1 (0.021%)<br>• Insolitispirillum peregrinum [taxid 80876]: 1 (0.021%)<br>• Lysobacter aestuarii [taxid 1706195]: 1 (0.021%)<br>• other: 7 (0.148%)

Operational Taxonomic Unit (OTU)	Correct identifications	Wrong or overspecific identifications at species rank
Benchmark OTU ID: `CP002246-_Proteobacteria`<br>OTU taxon: Yersinia enterocolitica subsp. palearctica 105.5R(r) [taxid 994476]<br>Expected: Yersinia enterocolitica [taxid 630] (species)<br>Number of reads: 9358<br>Number of identified reads: 9296 (99.337%)	• species: 2224 (23.765%)<br>• **genus: 3199 (34.184%)**<br>• family: 301 (3.216%)<br>• order: 1775 (18.967%)<br>• class: 372 (3.975%)<br>• phylum: 194 (2.073%)<br>• superkingdom: 252 (2.692%)<br>• root: 973 (10.397%)	• Salmonella enterica [taxid 28901]: 30 (0.32%)<br>• Escherichia coli [taxid 562]: 16 (0.17%)<br>• Yersinia frederiksenii [taxid 29484]: 15 (0.16%)<br>• Yersinia pestis [taxid 632]: 13 (0.138%)<br>• Yersinia intermedia [taxid 631]: 10 (0.106%)<br>• Yersinia kristensenii [taxid 28152]: 10 (0.106%)<br>• Yersinia mollaretii [taxid 33060]: 9 (0.096%)<br>• Yersinia aldovae [taxid 29483]: 6 (0.064%)<br>• other: 96 (1.025%)
Benchmark OTU ID: `FR729477-_Proteobacteria`<br>OTU taxon: Yersinia enterocolitica subsp. palearctica Y11 [taxid 930944]<br>Expected: Yersinia enterocolitica [taxid 630] (species)<br>Number of reads: 9361<br>Number of identified reads: 9302 (99.369%)	• species: 2268 (24.228%)<br>• **genus: 3163 (33.789%)**<br>• family: 310 (3.311%)<br>• order: 1795 (19.175%)<br>• class: 346 (3.696%)<br>• phylum: 204 (2.179%)<br>• superkingdom: 245 (2.617%)<br>• root: 964 (10.298%)	• Salmonella enterica [taxid 28901]: 19 (0.202%)<br>• Yersinia pestis [taxid 632]: 16 (0.17%)<br>• Yersinia frederiksenii [taxid 29484]: 16 (0.17%)<br>• Escherichia coli [taxid 562]: 14 (0.149%)<br>• Yersinia mollaretii [taxid 33060]: 9 (0.096%)<br>• Yersinia kristensenii [taxid 28152]: 8 (0.085%)<br>• Yersinia ruckeri [taxid 29486]: 6 (0.064%)<br>• Yersinia aldovae [taxid 29483]: 6 (0.064%)<br>• other: 96 (1.025%)
Benchmark OTU ID: `CP002956-_Pathogens`<br>OTU taxon: Yersinia pestis A1122 [taxid 1035377]<br>Expected: Yersinia pestis [taxid 632] (species)<br>Number of reads: 10170<br>Number of identified reads: 10104 (99.351%)	• species: 527 (5.181%)<br>• **genus: 5369 (52.792%)**<br>• family: 302 (2.969%)<br>• order: 1986 (19.528%)<br>• class: 390 (3.834%)<br>• phylum: 173 (1.701%)<br>• superkingdom: 256 (2.517%)<br>• root: 1091 (10.727%)	• Yersinia pseudotuberculosis [taxid 633]: 56 (0.55%)<br>• Escherichia coli [taxid 562]: 44 (0.432%)<br>• Salmonella enterica [taxid 28901]: 27 (0.265%)<br>• Yersinia enterocolitica [taxid 630]: 10 (0.098%)<br>• Yersinia frederiksenii [taxid 29484]: 6 (0.058%)<br>• Yersinia similis [taxid 367190]: 4 (0.039%)<br>• Yersinia kristensenii [taxid 28152]: 3 (0.029%)<br>• Klebsiella pneumoniae [taxid 573]: 3 (0.029%)<br>• other: 85 (0.835%)
Benchmark OTU ID: `CP000308-_Pathogens`<br>OTU taxon: Yersinia pestis Antiqua [taxid 360102]<br>Expected: Yersinia pestis [taxid 632] (species)<br>Number of reads: 10542<br>Number of identified reads: 10476 (99.373%)	• species: 495 (4.695%)<br>• **genus: 5399 (51.214%)**<br>• family: 296 (2.807%)<br>• order: 2268 (21.513%)<br>• class: 386 (3.661%)<br>• phylum: 196 (1.859%)<br>• superkingdom: 282 (2.675%)<br>• root: 1146 (10.87%)	• Escherichia coli [taxid 562]: 75 (0.711%)<br>• Yersinia pseudotuberculosis [taxid 633]: 66 (0.626%)<br>• Salmonella enterica [taxid 28901]: 18 (0.17%)<br>• Yersinia enterocolitica [taxid 630]: 7 (0.066%)<br>• Serratia symbiotica [taxid 138074]: 6 (0.056%)<br>• Yersinia kristensenii [taxid 28152]: 4 (0.037%)<br>• Citrobacter freundii [taxid 546]: 3 (0.028%)<br>• Rouxiella badensis [taxid 1646377]: 3 (0.028%)<br>• other: 107 (1.014%)

Operational Taxonomic Unit (OTU)	Correct identifications	Wrong or overspecific identifications at species rank
Benchmark OTU ID: `CP001589-_Pathogens`<br>OTU taxon: Yersinia pestis D182038 [taxid 637385]<br>Expected: Yersinia pestis [taxid 632] (species)<br>Number of reads: 10353<br>Number of identified reads: 10271 (99.207%)	• species: 522 (5.042%)<br>• **genus: 5419 (52.342%)**<br>• family: 327 (3.158%)<br>• order: 2023 (19.54%)<br>• class: 384 (3.709%)<br>• phylum: 202 (1.951%)<br>• superkingdom: 235 (2.269%)<br>• root: 1150 (11.107%)	• Escherichia coli [taxid 562]: 73 (0.705%)<br>• Yersinia pseudotuberculosis [taxid 633]: 67 (0.647%)<br>• Salmonella enterica [taxid 28901]: 21 (0.202%)<br>• Yersinia wautersii [taxid 1341643]: 10 (0.096%)<br>• Serratia symbiotica [taxid 138074]: 7 (0.067%)<br>• Yersinia enterocolitica [taxid 630]: 5 (0.048%)<br>• Klebsiella pneumoniae [taxid 573]: 4 (0.038%)<br>• Xenorhabdus nematophila [taxid 628]: 3 (0.028%)<br>• other: 96 (0.927%)
Benchmark OTU ID: `CP001593-_Pathogens`<br>OTU taxon: Yersinia pestis Z176003 [taxid 637386]<br>Expected: Yersinia pestis [taxid 632] (species)<br>Number of reads: 10170<br>Number of identified reads: 10089 (99.203%)	• species: 508 (4.995%)<br>• **genus: 5316 (52.271%)**<br>• family: 334 (3.284%)<br>• order: 2029 (19.95%)<br>• class: 401 (3.942%)<br>• phylum: 183 (1.799%)<br>• superkingdom: 247 (2.428%)<br>• root: 1063 (10.452%)	• Yersinia pseudotuberculosis [taxid 633]: 63 (0.619%)<br>• Escherichia coli [taxid 562]: 45 (0.442%)<br>• Salmonella enterica [taxid 28901]: 20 (0.196%)<br>• Erwinia tracheiphila [taxid 65700]: 12 (0.117%)<br>• Yersinia enterocolitica [taxid 630]: 8 (0.078%)<br>• Yersinia frederiksenii [taxid 29484]: 6 (0.058%)<br>• Serratia symbiotica [taxid 138074]: 6 (0.058%)<br>• Yersinia wautersii [taxid 1341643]: 6 (0.058%)<br>• other: 105 (1.032%)
Benchmark OTU ID: `AE017042-_Pathogens`<br>OTU taxon: Yersinia pestis biovar Microtus str. 91001 [taxid 229193]<br>Expected: Yersinia pestis [taxid 632] (species)<br>Number of reads: 10274<br>Number of identified reads: 10198 (99.26%)	• species: 467 (4.545%)<br>• **genus: 5474 (53.28%)**<br>• family: 308 (2.997%)<br>• order: 2049 (19.943%)<br>• class: 369 (3.591%)<br>• phylum: 185 (1.8%)<br>• superkingdom: 222 (2.16%)<br>• root: 1116 (10.862%)	• Yersinia pseudotuberculosis [taxid 633]: 63 (0.613%)<br>• Escherichia coli [taxid 562]: 53 (0.515%)<br>• Salmonella enterica [taxid 28901]: 27 (0.262%)<br>• Yersinia enterocolitica [taxid 630]: 9 (0.087%)<br>• Candidatus Erwinia haradaeae [taxid 1922217]: 6 (0.058%)<br>• Klebsiella pneumoniae [taxid 573]: 4 (0.038%)<br>• Yersinia wautersii [taxid 1341643]: 3 (0.029%)<br>• Azospirillum brasilense [taxid 192]: 3 (0.029%)<br>• other: 93 (0.905%)
Benchmark OTU ID: `BX936398-_Proteobacteria`<br>OTU taxon: Yersinia pseudotuberculosis IP 32953 [taxid 273123]<br>Expected: Yersinia pseudotuberculosis [taxid 633] (species)<br>Number of reads: 9791<br>Number of identified reads: 9739 (99.468%)	• species: 557 (5.688%)<br>• **genus: 5281 (53.937%)**<br>• family: 306 (3.125%)<br>• order: 1770 (18.077%)<br>• class: 359 (3.666%)<br>• phylum: 203 (2.073%)<br>• superkingdom: 239 (2.441%)<br>• root: 1020 (10.417%)	• Yersinia pestis [taxid 632]: 144 (1.47%)<br>• Escherichia coli [taxid 562]: 26 (0.265%)<br>• Salmonella enterica [taxid 28901]: 23 (0.234%)<br>• Yersinia frederiksenii [taxid 29484]: 6 (0.061%)<br>• Yersinia pekkanenii [taxid 1288385]: 6 (0.061%)<br>• Serratia symbiotica [taxid 138074]: 5 (0.051%)<br>• Yersinia wautersii [taxid 1341643]: 5 (0.051%)<br>• Yersinia aldovae [taxid 29483]: 5 (0.051%)<br>• other: 108 (1.103%)

Operational Taxonomic Unit (OTU)	Correct identifications	Wrong or overspecific identifications at species rank
Benchmark OTU ID: `CP001048-_Proteobacteria`<br>OTU taxon: Yersinia pseudotuberculosis PB1/+ [taxid 502801]<br>Expected: Yersinia pseudotuberculosis [taxid 633] (species)<br>Number of reads: 9681<br>Number of identified reads: 9624 (99.411%)	• species: 425 (4.39%)<br>• **genus: 5114 (52.825%)**<br>• family: 294 (3.036%)<br>• order: 1854 (19.15%)<br>• class: 391 (4.038%)<br>• phylum: 208 (2.148%)<br>• superkingdom: 260 (2.685%)<br>• root: 1074 (11.093%)	• Yersinia pestis [taxid 632]: 145 (1.497%)<br>• Yersinia enterocolitica [taxid 630]: 41 (0.423%)<br>• Escherichia coli [taxid 562]: 25 (0.258%)<br>• Salmonella enterica [taxid 28901]: 23 (0.237%)<br>• Klebsiella pneumoniae [taxid 573]: 9 (0.092%)<br>• Yersinia wautersii [taxid 1341643]: 8 (0.082%)<br>• Yersinia mollaretii [taxid 33060]: 6 (0.061%)<br>• Yersinia frederiksenii [taxid 29484]: 5 (0.051%)<br>• other: 100 (1.032%)
Benchmark OTU ID: `CP000950-_Proteobacteria`<br>OTU taxon: Yersinia pseudotuberculosis YPIII [taxid 502800]<br>Expected: Yersinia pseudotuberculosis [taxid 633] (species)<br>Number of reads: 9667<br>Number of identified reads: 9606 (99.368%)	• species: 609 (6.299%)<br>• **genus: 5104 (52.798%)**<br>• family: 286 (2.958%)<br>• order: 1749 (18.092%)<br>• class: 379 (3.92%)<br>• phylum: 193 (1.996%)<br>• superkingdom: 231 (2.389%)<br>• root: 1050 (10.861%)	• Yersinia pestis [taxid 632]: 151 (1.562%)<br>• Salmonella enterica [taxid 28901]: 23 (0.237%)<br>• Escherichia coli [taxid 562]: 20 (0.206%)<br>• Yersinia enterocolitica [taxid 630]: 11 (0.113%)<br>• Yersinia wautersii [taxid 1341643]: 6 (0.062%)<br>• Yersinia mollaretii [taxid 33060]: 4 (0.041%)<br>• Yersinia kristensenii [taxid 28152]: 4 (0.041%)<br>• Yersinia pekkanenii [taxid 1288385]: 3 (0.031%)<br>• other: 72 (0.744%)
Benchmark OTU ID: `CP002850-_Proteobacteria`<br>OTU taxon: Zymomonas mobilis subsp. mobilis ATCC 10988 [taxid 555217]<br>Expected: Zymomonas mobilis [taxid 542] (species)<br>Number of reads: 3665<br>Number of identified reads: 3639 (99.29%)	• **species: 2656 (72.469%)**<br>• genus: 0 (0.0%)<br>• family: 76 (2.073%)<br>• order: 36 (0.982%)<br>• class: 148 (4.038%)<br>• phylum: 92 (2.51%)<br>• superkingdom: 131 (3.574%)<br>• root: 492 (13.424%)	• Mycobacterium ulcerans [taxid 1809]: 1 (0.027%)<br>• Thermogutta terrifontis [taxid 1331910]: 1 (0.027%)<br>• Bradyrhizobium sp. Gha [taxid 1855318]: 1 (0.027%)<br>• Mikania micrantha [taxid 192012]: 1 (0.027%)<br>• Tanacetum cinerariifolium [taxid 118510]: 1 (0.027%)<br>• Neorhizobium alkalisoli [taxid 528178]: 1 (0.027%)<br>• Phenylobacterium hankyongense [taxid 1813876]: 1 (0.027%)<br>• Calocera cornea [taxid 29889]: 1 (0.027%)<br>• other: 3 (0.081%)
Benchmark OTU ID: `CP003704-_Proteobacteria`<br>OTU taxon: Zymomonas mobilis subsp. mobilis ATCC 29191 [taxid 627344]<br>Expected: Zymomonas mobilis [taxid 542] (species)<br>Number of reads: 3529<br>Number of identified reads: 3507 (99.376%)	• **species: 2302 (65.23%)**<br>• genus: 0 (0.0%)<br>• family: 83 (2.351%)<br>• order: 39 (1.105%)<br>• class: 196 (5.553%)<br>• phylum: 103 (2.918%)<br>• superkingdom: 150 (4.25%)<br>• root: 628 (17.795%)	• Atopobium deltae [taxid 1393034]: 1 (0.028%)<br>• Acetobacter tropicalis [taxid 104102]: 1 (0.028%)<br>• Sphingomonas dokdonensis [taxid 344880]: 1 (0.028%)<br>• Pseudolabrys sp. GY_H [taxid 2292256]: 1 (0.028%)<br>• Pseudooceanicola antarcticus [taxid 1247613]: 1 (0.028%)<br>• Acetobacter nitrogenifigens [taxid 285268]: 1 (0.028%)<br>• Gluconobacter japonicus [taxid 376620]: 1 (0.028%)<br>• Methylobacterium gnaphalii [taxid 1010610]: 1 (0.028%)<br>• other: 5 (0.141%)

Operational Taxonomic Unit (OTU)	Correct identifications	Wrong or overspecific identifications at species rank
Benchmark OTU ID: `AE008692-_Proteobacteria`<br>OTU taxon: Zymomonas mobilis subsp. mobilis ZM4 = ATCC 31821 [taxid 264203]<br>Expected: Zymomonas mobilis [taxid 542] (species)<br>Number of reads: 3743<br>Number of identified reads: 3725 (99.519%)	• **species: 2663 (71.146%)**<br>• genus: 0 (0.0%)<br>• family: 73 (1.95%)<br>• order: 36 (0.961%)<br>• class: 145 (3.873%)<br>• phylum: 130 (3.473%)<br>• superkingdom: 121 (3.232%)<br>• root: 549 (14.667%)	• Globodera pallida [taxid 36090]: 2 (0.053%)<br>• Bacterioplanes sanyensis [taxid 1249553]: 1 (0.026%)<br>• Haptolina brevifila [taxid 156173]: 1 (0.026%)<br>• Acidisphaera rubrifaciens [taxid 50715]: 1 (0.026%)<br>• Streptomyces lydicamycinicus [taxid 1546107]: 1 (0.026%)<br>• Limimaricola soesokkakensis [taxid 1343159]: 1 (0.026%)<br>• Skermanella aerolata [taxid 393310]: 1 (0.026%)<br>• Crucibulum laeve [taxid 68775]: 1 (0.026%)<br>• other: 6 (0.16%)
Benchmark OTU ID: `CP002865-_Proteobacteria`<br>OTU taxon: Zymomonas mobilis subsp. pomaceae ATCC 29192 [taxid 579138]<br>Expected: Zymomonas mobilis [taxid 542] (species)<br>Number of reads: 3593<br>Number of identified reads: 3557 (98.998%)	• **species: 2509 (69.83%)**<br>• genus: 0 (0.0%)<br>• family: 89 (2.477%)<br>• order: 38 (1.057%)<br>• class: 167 (4.647%)<br>• phylum: 107 (2.978%)<br>• superkingdom: 142 (3.952%)<br>• root: 504 (14.027%)	• Hartmannibacter diazotrophicus [taxid 1482074]: 1 (0.027%)<br>• Pseudorhizobium banfieldiae [taxid 1125847]: 1 (0.027%)<br>• Sphingomonas rubra [taxid 634430]: 1 (0.027%)<br>• Helicobacter pylori [taxid 210]: 1 (0.027%)<br>• Arcticibacterium luteifluviistationis [taxid 1784714]: 1 (0.027%)<br>• Phlebotomus kandelakii [taxid 1109342]: 1 (0.027%)<br>• Amycolatopsis vastitatis [taxid 1905142]: 1 (0.027%)<br>• Paraburkholderia kirstenboschensis [taxid 1245436]: 1 (0.027%)
Benchmark OTU ID: `CP001791-_Firmicutes`<br>OTU taxon: [Bacillus] selenitireducens MLS10 [taxid 439292]<br>Expected: [Bacillus] selenitireducens [taxid 85683] (species)<br>Number of reads: 4979<br>Number of identified reads: 4961 (99.638%)	• **species: 3197 (64.209%)**<br>• family: 1 (0.02%)<br>• order: 716 (14.38%)<br>• class: 32 (0.642%)<br>• phylum: 74 (1.486%)<br>• superkingdom: 313 (6.286%)<br>• root: 625 (12.552%)	• Bacillus beveridgei [taxid 632773]: 13 (0.261%)<br>• Nocardia terpenica [taxid 455432]: 1 (0.02%)<br>• Vulcanibacillus modesticaldus [taxid 337097]: 1 (0.02%)<br>• Bacillus urumqiensis [taxid 1548213]: 1 (0.02%)<br>• Alkalicoccus saliphilus [taxid 200989]: 1 (0.02%)<br>• Gracilibacillus halophilus [taxid 470864]: 1 (0.02%)<br>• Staphylococcus arlettae [taxid 29378]: 1 (0.02%)<br>• other: 19 (0.381%)
Benchmark OTU ID: `CP002272-_Proteobacteria`<br>OTU taxon: [Enterobacter] lignolyticus SCF1 [taxid 701347]<br>Expected: [Enterobacter] lignolyticus [taxid 1334193] (species)<br>Number of reads: 9948<br>Number of identified reads: 9913 (99.648%)	• species: 3726 (37.454%)<br>• genus: 26 (0.261%)<br>• **family: 3769 (37.887%)**<br>• order: 876 (8.805%)<br>• class: 261 (2.623%)<br>• phylum: 201 (2.02%)<br>• superkingdom: 265 (2.663%)<br>• root: 785 (7.891%)	• **Salmonella enterica [taxid 28901]: 216 (2.171%)**<br>• Escherichia coli [taxid 562]: 83 (0.834%)<br>• Klebsiella pneumoniae [taxid 573]: 17 (0.17%)<br>• Pluralibacter gergoviae [taxid 61647]: 6 (0.06%)<br>• Citrobacter youngae [taxid 133448]: 5 (0.05%)<br>• Yokenella regensburgei [taxid 158877]: 4 (0.04%)<br>• Citrobacter freundii [taxid 546]: 4 (0.04%)<br>• Siccibacter turicensis [taxid 357233]: 4 (0.04%)<br>• other: 111 (1.115%)
Benchmark OTU ID: `CP001107-_Firmicutes`<br>OTU taxon: [Eubacterium] rectale ATCC 33656 [taxid 515619]<br>Expected: [Eubacterium] rectale [taxid 39491] (species)<br>Number of reads: 4750<br>Number of identified reads: 4717 (99.305%)	• species: 703 (14.8%)<br>• family: 156 (3.284%)<br>• **order: 3069 (64.61%)**<br>• class: 3 (0.063%)<br>• phylum: 112 (2.357%)<br>• superkingdom: 175 (3.684%)<br>• root: 496 (10.442%)	• Eubacterium plexicaudatum [taxid 97253]: 2 (0.042%)<br>• Agathobacter ruminis [taxid 1712665]: 2 (0.042%)<br>• Roseburia intestinalis [taxid 166486]: 2 (0.042%)<br>• Faecalibacterium prausnitzii [taxid 853]: 2 (0.042%)<br>• Blautia obeum [taxid 40520]: 2 (0.042%)<br>• [Clostridium] scindens [taxid 29347]: 1 (0.021%)<br>• Eisenbergiella tayi [taxid 1432052]: 1 (0.021%)<br>• other: 21 (0.442%)

Operational Taxonomic Unit (OTU)	Correct identifications	Wrong or overspecific identifications at species rank
Benchmark OTU ID: `FP929042-_Firmicutes`<br>OTU taxon: [Eubacterium] rectale DSM 17629 [taxid 657318]<br>Expected: [Eubacterium] rectale [taxid 39491] (species)<br>Number of reads: 4380<br>Number of identified reads: 4343 (99.155%)	• species: 511 (11.666%)<br>• family: 170 (3.881%)<br>• **order: 2896 (66.118%)**<br>• class: 1 (0.022%)<br>• phylum: 179 (4.086%)<br>• superkingdom: 136 (3.105%)<br>• root: 448 (10.228%)	• Roseburia intestinalis [taxid 166486]: 4 (0.091%)<br>• Roseburia inulinivorans [taxid 360807]: 3 (0.068%)<br>• [Ruminococcus] torques [taxid 33039]: 3 (0.068%)<br>• Lachnospira eligens [taxid 39485]: 2 (0.045%)<br>• Lachnoclostridium phytofermentans [taxid 66219]: 2 (0.045%)<br>• Hespellia stercorisuis [taxid 180311]: 1 (0.022%)<br>• Clostridium uliginosum [taxid 119641]: 1 (0.022%)<br>• other: 17 (0.388%)
Benchmark OTU ID: `FP929043-_Firmicutes`<br>OTU taxon: [Eubacterium] rectale M104/1 [taxid 657317]<br>Expected: [Eubacterium] rectale [taxid 39491] (species)<br>Number of reads: 4862<br>Number of identified reads: 4835 (99.444%)	• species: 882 (18.14%)<br>• family: 147 (3.023%)<br>• **order: 2972 (61.127%)**<br>• class: 1 (0.02%)<br>• phylum: 123 (2.529%)<br>• superkingdom: 170 (3.496%)<br>• root: 534 (10.983%)	• Collinsella tanakaei [taxid 626935]: 5 (0.102%)<br>• Roseburia inulinivorans [taxid 360807]: 4 (0.082%)<br>• Roseburia intestinalis [taxid 166486]: 3 (0.061%)<br>• Roseburia faecis [taxid 301302]: 2 (0.041%)<br>• [Bacteroides] pectinophilus [taxid 384638]: 2 (0.041%)<br>• Blautia glucerasea [taxid 536633]: 1 (0.02%)<br>• Eubacterium oxidoreducens [taxid 1732]: 1 (0.02%)<br>• other: 24 (0.493%)
Benchmark OTU ID: `FP929059-_Firmicutes`<br>OTU taxon: [Eubacterium] siraeum V10Sc8a [taxid 717961]<br>Expected: [Eubacterium] siraeum [taxid 39492] (species)<br>Number of reads: 3605<br>Number of identified reads: 3591 (99.611%)	• species: 545 (15.117%)<br>• family: 82 (2.274%)<br>• **order: 1593 (44.188%)**<br>• class: 4 (0.11%)<br>• phylum: 151 (4.188%)<br>• superkingdom: 291 (8.072%)<br>• root: 918 (25.464%)	• Anaerotruncus colihominis [taxid 169435]: 1 (0.027%)<br>• Bacteroides graminisolvens [taxid 477666]: 1 (0.027%)<br>• Marvinbryantia formatexigens [taxid 168384]: 1 (0.027%)<br>• Mycolicibacter senuensis [taxid 386913]: 1 (0.027%)<br>• Desulfotomaculum hydrothermale [taxid 412895]: 1 (0.027%)<br>• Schistosoma bovis [taxid 6184]: 1 (0.027%)<br>• Apostichopus japonicus [taxid 307972]: 1 (0.027%)<br>• other: 13 (0.36%)
Benchmark OTU ID: `AE017143-_Proteobacteria`<br>OTU taxon: [Haemophilus] ducreyi 35000HP [taxid 233412]<br>Expected: [Haemophilus] ducreyi [taxid 730] (species)<br>Number of reads: 2939<br>Number of identified reads: 2902 (98.741%)	• **species: 1905 (64.817%)**<br>• genus: 22 (0.748%)<br>• family: 354 (12.044%)<br>• order: 0 (0.0%)<br>• class: 224 (7.621%)<br>• phylum: 60 (2.041%)<br>• superkingdom: 89 (3.028%)<br>• root: 244 (8.302%)	• Haemophilus haemolyticus [taxid 726]: 5 (0.17%)<br>• Escherichia coli [taxid 562]: 3 (0.102%)<br>• Salmonella enterica [taxid 28901]: 2 (0.068%)<br>• Gilliamella apicola [taxid 1196095]: 2 (0.068%)<br>• Azospirillum brasilense [taxid 192]: 1 (0.034%)<br>• Beggiatoa sp. SS [taxid 422288]: 1 (0.034%)<br>• Providencia stuartii [taxid 588]: 1 (0.034%)<br>• [Actinobacillus] rossii [taxid 123820]: 1 (0.034%)<br>• other: 21 (0.714%)

Operational Taxonomic Unit (OTU)	Correct identifications	Wrong or overspecific identifications at species rank
Benchmark OTU ID: `FP929055-_Firmicutes`<br>OTU taxon: [Ruminococcus] torques L2-14 [taxid 657313]<br>Expected: [Ruminococcus] torques [taxid 33039] (species)<br>Number of reads: 4374<br>Number of identified reads: 4352 (99.497%)	• species: 396 (9.053%)<br>• genus: 12 (0.274%)<br>• family: 141 (3.223%)<br>• **order: 2978 (68.084%)**<br>• class: 2 (0.045%)<br>• phylum: 266 (6.081%)<br>• superkingdom: 158 (3.612%)<br>• root: 395 (9.03%)	• [Ruminococcus] lactaris [taxid 46228]: 5 (0.114%)<br>• Dorea longicatena [taxid 88431]: 5 (0.114%)<br>• [Ruminococcus] gnavus [taxid 33038]: 5 (0.114%)<br>• Tyzzerella nexilis [taxid 29361]: 4 (0.091%)<br>• Blautia obeum [taxid 40520]: 4 (0.091%)<br>• Dorea formicigenerans [taxid 39486]: 3 (0.068%)<br>• [Eubacterium] rectale [taxid 39491]: 2 (0.045%)<br>• Hungatella hathewayi [taxid 154046]: 2 (0.045%)<br>• other: 28 (0.64%)
Benchmark OTU ID: `CP003809-_Proteobacteria`<br>OTU taxon: alpha proteobacterium HIMB5 [taxid 859653]<br>Expected: Pelagibacteraceae [taxid 1655514] (family)<br>Number of reads: 2138<br>Number of identified reads: 2118 (99.064%)	• **family: 1078 (50.42%)**<br>• order: 58 (2.712%)<br>• class: 133 (6.22%)<br>• phylum: 591 (27.642%)<br>• superkingdom: 110 (5.144%)<br>• root: 147 (6.875%)	• Candidatus Pelagibacter ubique [taxid 198252]: 4 (0.187%)<br>• Pseudomonas savastanoi [taxid 29438]: 1 (0.046%)<br>• Roseovarius faecimaris [taxid 2494550]: 1 (0.046%)<br>• Candidatus Deianiraea vastatrix [taxid 2163644]: 1 (0.046%)<br>• Candidatus Pelagibacter giovannonii [taxid 2563896]: 1 (0.046%)<br>• Pseudomonas anguilliseptica [taxid 53406]: 1 (0.046%)
Benchmark OTU ID: `CP003801-_Proteobacteria`<br>OTU taxon: alpha proteobacterium HIMB59 [taxid 744985]<br>Expected: Pelagibacteraceae [taxid 1655514] (family)<br>Number of reads: 2289<br>Number of identified reads: 2262 (98.82%)	• **family: 1849 (80.777%)**<br>• order: 2 (0.087%)<br>• class: 75 (3.276%)<br>• phylum: 78 (3.407%)<br>• superkingdom: 96 (4.193%)<br>• root: 161 (7.033%)	• Alexandrium catenella [taxid 2925]: 1 (0.043%)<br>• Agarivorans albus [taxid 182262]: 1 (0.043%)<br>• Candidatus Pelagibacter giovannonii [taxid 2563896]: 1 (0.043%)<br>• Wolinella succinogenes [taxid 844]: 1 (0.043%)
Benchmark OTU ID: `CP004348-_Proteobacteria`<br>OTU taxon: beta proteobacterium CB [taxid 543913]<br>Expected: beta proteobacterium CB [taxid 543913] (species)<br>Number of reads: 3719<br>Number of identified reads: 3699 (99.462%)	• species: 909 (24.442%)<br>• **class: 2116 (56.897%)**<br>• phylum: 197 (5.297%)<br>• superkingdom: 133 (3.576%)<br>• root: 344 (9.249%)	• Polynucleobacter duraquae [taxid 1835254]: 5 (0.134%)<br>• Polynucleobacter meluiroseus [taxid 1938814]: 4 (0.107%)<br>• Polynucleobacter asymbioticus [taxid 576611]: 3 (0.08%)<br>• Polynucleobacter paneuropaeus [taxid 2527775]: 2 (0.053%)<br>• Taylorella equigenitalis [taxid 29575]: 2 (0.053%)<br>• other: 22 (0.591%)
Benchmark OTU ID: `FP929060-_Firmicutes`<br>OTU taxon: butyrate-producing bacterium SM4/1 [taxid 245012]<br>Expected: Clostridiales [taxid 186802] (order)<br>Number of reads: 2493<br>Number of identified reads: 2489 (99.839%)	• **order: 1911 (76.654%)**<br>• class: 1 (0.04%)<br>• phylum: 104 (4.171%)<br>• superkingdom: 113 (4.532%)<br>• root: 359 (14.4%)	• Lacrimispora saccharolytica [taxid 84030]: 5 (0.2%)<br>• Hungatella hathewayi [taxid 154046]: 2 (0.08%)<br>• Clostridium indicum [taxid 759821]: 1 (0.04%)<br>• [Clostridium] polysaccharolyticum [taxid 29364]: 1 (0.04%)<br>• Holdemania filiformis [taxid 61171]: 1 (0.04%)<br>• other: 12 (0.481%)
Benchmark OTU ID: `FP929140-_Proteobacteria`<br>OTU taxon: gamma proteobacterium HdN1 [taxid 83406]<br>Expected: Gammaproteobacteria [taxid 1236] (class)<br>Number of reads: 9438<br>Number of identified reads: 9413 (99.735%)	• **class: 7432 (78.745%)**<br>• phylum: 420 (4.45%)<br>• superkingdom: 376 (3.983%)<br>• root: 1181 (12.513%)	• Escherichia coli [taxid 562]: 3 (0.031%)<br>• Acinetobacter baumannii [taxid 470]: 2 (0.021%)<br>• Oleiphilus messinensis [taxid 141451]: 2 (0.021%)<br>• Proteus mirabilis [taxid 584]: 1 (0.01%)<br>• other: 49 (0.519%)

Operational Taxonomic Unit (OTU)	Correct identifications	Wrong or overspecific identifications at species rank
Benchmark OTU ID: `CP003546-_Proteobacteria`<br>OTU taxon: secondary endosymbiont of Ctenarytaina eucalypti [taxid 1199245]<br>Expected: secondary endosymbiont of Ctenarytaina eucalypti [taxid 1199245] (species)<br>Number of reads: 2358<br>Number of identified reads: 2229 (94.529%)	• species: 875 (37.107%)<br>• family: 20 (0.848%)<br>• order: 133 (5.64%)<br>• class: 98 (4.156%)<br>• phylum: 38 (1.611%)<br>• superkingdom: 65 (2.756%)<br>• **root: 997 (42.281%)**	• Escherichia coli [taxid 562]: 2 (0.084%)<br>• Serratia symbiotica [taxid 138074]: 2 (0.084%)<br>• Enterobacillus tribolii [taxid 1487935]: 1 (0.042%)<br>• Sodalis endosymbiont of Henestaris halophilus [taxid 1929246]: 1 (0.042%)<br>• Reinekea blandensis [taxid 374838]: 1 (0.042%)<br>• Hahella chejuensis [taxid 158327]: 1 (0.042%)<br>• Salmonella enterica [taxid 28901]: 1 (0.042%)<br>• other: 9 (0.381%)

Benchmark dataset, shuffled reads

Operational Taxonomic Unit (OTU)	Correct identifications	Wrong or overspecific identifications at species rank
Benchmark OTU ID: `AE017042-_Random`<br>OTU taxon: AE017042-_Random [taxid 0]<br>Expected: unknown [taxid 1] (no rank)<br>Number of reads: 74929<br>Number of identified reads: 69971 (93.383%)	• species: 0 (0.0%)<br>• genus: 0 (0.0%)<br>• family: 0 (0.0%)<br>• order: 0 (0.0%)<br>• class: 0 (0.0%)<br>• phylum: 0 (0.0%)<br>• superkingdom: 0 (0.0%)<br>• **root: 68524 (91.451%)**	• Musca domestica [taxid 7370]: 4 (0.005%)<br>• Diabrotica virgifera [taxid 50389]: 3 (0.004%)<br>• Cotesia congregata [taxid 51543]: 3 (0.004%)<br>• Aulographum hederae [taxid 1176130]: 3 (0.004%)<br>• Sitophilus oryzae [taxid 7048]: 3 (0.004%)<br>• Empedobacter brevis [taxid 247]: 3 (0.004%)<br>• Prymnesium parvum [taxid 97485]: 3 (0.004%)<br>• Rhinopithecus roxellana [taxid 61622]: 2 (0.002%)<br>• other: 245 (0.326%)
Benchmark OTU ID: `ENA|AJ235269|AJ235269.1-_Random`<br>OTU taxon: ENA|AJ235269|AJ235269.1-_Random [taxid 0]<br>Expected: unknown [taxid 1] (no rank)<br>Number of reads: 14562<br>Number of identified reads: 10320 (70.869%)	• species: 0 (0.0%)<br>• genus: 0 (0.0%)<br>• family: 0 (0.0%)<br>• order: 0 (0.0%)<br>• class: 0 (0.0%)<br>• phylum: 0 (0.0%)<br>• superkingdom: 0 (0.0%)<br>• **root: 10223 (70.203%)**	• Gymnoxanthella radiolariae [taxid 1798043]: 2 (0.013%)<br>• Mucuna pruriens [taxid 157652]: 1 (0.006%)<br>• Pararcticibacter tournemirensis [taxid 699437]: 1 (0.006%)<br>• Yoonia sediminilitoris [taxid 1286148]: 1 (0.006%)<br>• Neotessella volvocina [taxid 2419522]: 1 (0.006%)<br>• Phytophthora megakarya [taxid 4795]: 1 (0.006%)<br>• Strongyloides stercoralis [taxid 6248]: 1 (0.006%)<br>• Paraprevotella clara [taxid 454154]: 1 (0.006%)<br>• other: 23 (0.157%)
Benchmark OTU ID: `ENA|AL732656|AL732656.1-_Random`<br>OTU taxon: ENA|AL732656|AL732656.1-_Random [taxid 0]<br>Expected: unknown [taxid 1] (no rank)<br>Number of reads: 33645<br>Number of identified reads: 28861 (85.78%)	• species: 0 (0.0%)<br>• genus: 0 (0.0%)<br>• family: 0 (0.0%)<br>• order: 0 (0.0%)<br>• class: 0 (0.0%)<br>• phylum: 0 (0.0%)<br>• superkingdom: 0 (0.0%)<br>• **root: 28541 (84.829%)**	• Plasmodium vinckei [taxid 5860]: 4 (0.011%)<br>• Achlya hypogyna [taxid 1202772]: 2 (0.005%)<br>• Spiroplasma litorale [taxid 216942]: 2 (0.005%)<br>• Penicillium roqueforti [taxid 5082]: 2 (0.005%)<br>• Cohnella lupini [taxid 1294267]: 2 (0.005%)<br>• Ilyobacter polytropus [taxid 167642]: 2 (0.005%)<br>• Tanacetum cinerariifolium [taxid 118510]: 2 (0.005%)<br>• bacterium [taxid 1869227]: 2 (0.005%)<br>• other: 64 (0.19%)
Benchmark OTU ID: `AM040265-_Random`<br>OTU taxon: AM040265-_Random [taxid 0]<br>Expected: unknown [taxid 1] (no rank)<br>Number of reads: 15349<br>Number of identified reads: 13388 (87.223%)	• species: 0 (0.0%)<br>• genus: 0 (0.0%)<br>• family: 0 (0.0%)<br>• order: 0 (0.0%)<br>• class: 0 (0.0%)<br>• phylum: 0 (0.0%)<br>• superkingdom: 0 (0.0%)<br>• **root: 12973 (84.52%)**	• Hyaloscypha variabilis [taxid 2482757]: 3 (0.019%)<br>• Tremella mesenterica [taxid 5217]: 2 (0.013%)<br>• Pseudonocardia sediminis [taxid 1397368]: 2 (0.013%)<br>• Alexandrium fundyense [taxid 2932]: 2 (0.013%)<br>• Mesorhizobium tamadayense [taxid 425306]: 1 (0.006%)<br>• Penicillium brasilianum [taxid 104259]: 1 (0.006%)<br>• Oceanimonas baumannii [taxid 129578]: 1 (0.006%)<br>• Shewanella hanedai [taxid 25]: 1 (0.006%)<br>• other: 46 (0.299%)

Operational Taxonomic Unit (OTU)	Correct identifications	Wrong or overspecific identifications at species rank
Benchmark OTU ID: `AM286280-_Random`<br>OTU taxon: AM286280-_Random [taxid 0]<br>Expected: unknown [taxid 1] (no rank)<br>Number of reads: 28114<br>Number of identified reads: 22313 (79.366%)	• species: 0 (0.0%)<br>• genus: 0 (0.0%)<br>• family: 0 (0.0%)<br>• order: 0 (0.0%)<br>• class: 0 (0.0%)<br>• phylum: 0 (0.0%)<br>• superkingdom: 0 (0.0%)<br>• **root: 22116 (78.665%)**	• Rubritalea profundi [taxid 1658618]: 2 (0.007%)<br>• Exophiala mesophila [taxid 212818]: 2 (0.007%)<br>• Apostasia shenzhenica [taxid 1088818]: 2 (0.007%)<br>• Tribolium castaneum [taxid 7070]: 2 (0.007%)<br>• Actinoallomurus bryophytorum [taxid 1490222]: 2 (0.007%)<br>• Rickettsia asiatica [taxid 238800]: 2 (0.007%)<br>• Calocera cornea [taxid 29889]: 1 (0.003%)<br>• Moritella marina [taxid 90736]: 1 (0.003%)<br>• other: 40 (0.142%)
Benchmark OTU ID: `AM747720-_Random`<br>OTU taxon: AM747720-_Random [taxid 0]<br>Expected: unknown [taxid 1] (no rank)<br>Number of reads: 62422<br>Number of identified reads: 41333 (66.215%)	• species: 0 (0.0%)<br>• genus: 0 (0.0%)<br>• family: 0 (0.0%)<br>• order: 0 (0.0%)<br>• class: 0 (0.0%)<br>• phylum: 0 (0.0%)<br>• superkingdom: 0 (0.0%)<br>• **root: 39148 (62.715%)**	• Symbiodinium microadriaticum [taxid 2951]: 5 (0.008%)<br>• Bordetella trematum [taxid 123899]: 4 (0.006%)<br>• Tremella mesenterica [taxid 5217]: 4 (0.006%)<br>• Salmo trutta [taxid 8032]: 3 (0.004%)<br>• Sphaerotilus hippei [taxid 744406]: 2 (0.003%)<br>• Oceanobacillus arenosus [taxid 1229153]: 2 (0.003%)<br>• Chilo suppressalis [taxid 168631]: 2 (0.003%)<br>• Paenibacillus silvae [taxid 1325358]: 2 (0.003%)<br>• other: 180 (0.288%)
Benchmark OTU ID: `AP010935-_Random`<br>OTU taxon: AP010935-_Random [taxid 0]<br>Expected: unknown [taxid 1] (no rank)<br>Number of reads: 31821<br>Number of identified reads: 28881 (90.76%)	• species: 0 (0.0%)<br>• genus: 0 (0.0%)<br>• family: 0 (0.0%)<br>• order: 0 (0.0%)<br>• class: 0 (0.0%)<br>• phylum: 0 (0.0%)<br>• superkingdom: 0 (0.0%)<br>• **root: 28530 (89.657%)**	• Tanacetum cinerariifolium [taxid 118510]: 3 (0.009%)<br>• Magnetospirillum kuznetsovii [taxid 2053833]: 3 (0.009%)<br>• Carassius auratus [taxid 7957]: 2 (0.006%)<br>• Acidomyces sp. ‘richmondensis’ [taxid 245562]: 2 (0.006%)<br>• Reticulomyxa filosa [taxid 46433]: 2 (0.006%)<br>• Paxillus rubicundulus [taxid 463315]: 2 (0.006%)<br>• Bactrocera latifrons [taxid 174628]: 1 (0.003%)<br>• Pseudomonas costantinii [taxid 168469]: 1 (0.003%)<br>• other: 71 (0.223%)
Benchmark OTU ID: `AP012203-_Random`<br>OTU taxon: AP012203-_Random [taxid 0]<br>Expected: unknown [taxid 1] (no rank)<br>Number of reads: 35872<br>Number of identified reads: 33570 (93.582%)	• species: 0 (0.0%)<br>• genus: 0 (0.0%)<br>• family: 0 (0.0%)<br>• order: 0 (0.0%)<br>• class: 0 (0.0%)<br>• phylum: 0 (0.0%)<br>• superkingdom: 0 (0.0%)<br>• **root: 32856 (91.592%)**	• Chanos chanos [taxid 29144]: 3 (0.008%)<br>• Pseudopedobacter saltans [taxid 151895]: 3 (0.008%)<br>• Lactobacillus mucosae [taxid 97478]: 2 (0.005%)<br>• [Candida] inconspicua [taxid 52247]: 2 (0.005%)<br>• Marinomonas hwangdonensis [taxid 1053647]: 2 (0.005%)<br>• Aspergillus tanneri [taxid 1220188]: 2 (0.005%)<br>• Campylobacter showae [taxid 204]: 2 (0.005%)<br>• Symbiodinium microadriaticum [taxid 2951]: 2 (0.005%)<br>• other: 106 (0.295%)

Operational Taxonomic Unit (OTU)	Correct identifications	Wrong or overspecific identifications at species rank
Benchmark OTU ID: `SHUFFLED_AP012340-_Random`<br>OTU taxon: SHUFFLED_AP012340-_Random [taxid 0]<br>Expected: unknown [taxid 1] (no rank)<br>Number of reads: 71483<br>Number of identified reads: 48150 (67.358%)	• species: 0 (0.0%)<br>• genus: 0 (0.0%)<br>• family: 0 (0.0%)<br>• order: 0 (0.0%)<br>• class: 0 (0.0%)<br>• phylum: 0 (0.0%)<br>• superkingdom: 0 (0.0%)<br>• **root: 45919 (64.237%)**	• Rhizoctonia solani [taxid 456999]: 3 (0.004%)<br>• Aegilops tauschii [taxid 37682]: 3 (0.004%)<br>• Schizochytrium aggregatum [taxid 4773]: 2 (0.002%)<br>• Trichoderma harzianum [taxid 5544]: 2 (0.002%)<br>• Streptomyces scabichelini [taxid 2711217]: 2 (0.002%)<br>• Steccherinum ochraceum [taxid 92696]: 2 (0.002%)<br>• Cryptosporangium aurantiacum [taxid 134849]: 2 (0.002%)<br>• Phytophthora cactorum [taxid 29920]: 2 (0.002%)<br>• other: 211 (0.295%)
Benchmark OTU ID: `BA000008-_Random`<br>OTU taxon: BA000008-_Random [taxid 0]<br>Expected: unknown [taxid 1] (no rank)<br>Number of reads: 16554<br>Number of identified reads: 15239 (92.056%)	• species: 0 (0.0%)<br>• genus: 0 (0.0%)<br>• family: 0 (0.0%)<br>• order: 0 (0.0%)<br>• class: 0 (0.0%)<br>• phylum: 0 (0.0%)<br>• superkingdom: 0 (0.0%)<br>• **root: 15016 (90.709%)**	• Mytilus coruscus [taxid 42192]: 5 (0.03%)<br>• Streptomyces hundungensis [taxid 1077946]: 4 (0.024%)<br>• Proteus penneri [taxid 102862]: 2 (0.012%)<br>• Hydatigera taeniaeformis [taxid 6205]: 2 (0.012%)<br>• Segetibacter aerophilus [taxid 670293]: 2 (0.012%)<br>• Candidatus Pacearchaeota archaeon [taxid 2026773]: 1 (0.006%)<br>• Paraflavitalea soli [taxid 2315862]: 1 (0.006%)<br>• Bactrocera dorsalis [taxid 27457]: 1 (0.006%)<br>• other: 47 (0.283%)
Benchmark OTU ID: `ENA|BX548020|BX548020.1-_Random`<br>OTU taxon: ENA|BX548020|BX548020.1-_Random [taxid 0]<br>Expected: unknown [taxid 1] (no rank)<br>Number of reads: 37514<br>Number of identified reads: 29774 (79.367%)	• species: 0 (0.0%)<br>• genus: 0 (0.0%)<br>• family: 0 (0.0%)<br>• order: 0 (0.0%)<br>• class: 0 (0.0%)<br>• phylum: 0 (0.0%)<br>• superkingdom: 0 (0.0%)<br>• **root: 28779 (76.715%)**	• Paenibacillus mucilaginosus [taxid 61624]: 3 (0.007%)<br>• Polyporus arcularius [taxid 5639]: 3 (0.007%)<br>• Graphocephala atropunctata [taxid 36148]: 2 (0.005%)<br>• Rhodococcus kyotonensis [taxid 398843]: 2 (0.005%)<br>• Actinoplanes lutulentus [taxid 1287878]: 2 (0.005%)<br>• Amborella trichopoda [taxid 13333]: 2 (0.005%)<br>• Tanacetum cinerariifolium [taxid 118510]: 2 (0.005%)<br>• Thalassiosira rotula [taxid 49265]: 2 (0.005%)<br>• other: 115 (0.306%)
Benchmark OTU ID: `ENA|BX571656|BX571656.1-_Random`<br>OTU taxon: ENA|BX571656|BX571656.1-_Random [taxid 0]<br>Expected: unknown [taxid 1] (no rank)<br>Number of reads: 31891<br>Number of identified reads: 30036 (94.183%)	• species: 0 (0.0%)<br>• genus: 0 (0.0%)<br>• family: 0 (0.0%)<br>• order: 0 (0.0%)<br>• class: 0 (0.0%)<br>• phylum: 0 (0.0%)<br>• superkingdom: 0 (0.0%)<br>• **root: 29403 (92.198%)**	• Crassostrea gigas [taxid 29159]: 3 (0.009%)<br>• Drosophila persimilis [taxid 7234]: 2 (0.006%)<br>• Bodo saltans [taxid 75058]: 2 (0.006%)<br>• Tritrichomonas foetus [taxid 1144522]: 2 (0.006%)<br>• Stenotrophomonas maltophilia [taxid 40324]: 2 (0.006%)<br>• Daphnia magna [taxid 35525]: 2 (0.006%)<br>• Alexandrium monilatum [taxid 311494]: 2 (0.006%)<br>• Peniophora sp. CONT [taxid 1314672]: 2 (0.006%)<br>• other: 90 (0.282%)

Operational Taxonomic Unit (OTU)	Correct identifications	Wrong or overspecific identifications at species rank
Benchmark OTU ID: `BX571857-_Random`<br>OTU taxon: BX571857-_Random [taxid 0]<br>Expected: unknown [taxid 1] (no rank)<br>Number of reads: 43854<br>Number of identified reads: 35247 (80.373%)	• species: 0 (0.0%)<br>• genus: 0 (0.0%)<br>• family: 0 (0.0%)<br>• order: 0 (0.0%)<br>• class: 0 (0.0%)<br>• phylum: 0 (0.0%)<br>• superkingdom: 0 (0.0%)<br>• **root: 34934 (79.659%)**	• Microbacterium telephonicum [taxid 1714841]: 2 (0.004%)<br>• Erythrura gouldiae [taxid 44316]: 2 (0.004%)<br>• Vitrella brassicaformis [taxid 1169539]: 2 (0.004%)<br>• Tetradesmus obliquus [taxid 3088]: 2 (0.004%)<br>• Geodermatophilus amargosae [taxid 1296565]: 2 (0.004%)<br>• Helicobacter pylori [taxid 210]: 2 (0.004%)<br>• bacterium [taxid 1869227]: 2 (0.004%)<br>• Roseivirga pacifica [taxid 1267423]: 2 (0.004%)<br>• other: 71 (0.161%)
Benchmark OTU ID: `ENA|CM000438|CM000438.1-_Random`<br>OTU taxon: ENA|CM000438|CM000438.1-_Random [taxid 0]<br>Expected: unknown [taxid 1] (no rank)<br>Number of reads: 54285<br>Number of identified reads: 34952 (64.386%)	• species: 0 (0.0%)<br>• genus: 0 (0.0%)<br>• family: 0 (0.0%)<br>• order: 0 (0.0%)<br>• class: 0 (0.0%)<br>• phylum: 0 (0.0%)<br>• superkingdom: 0 (0.0%)<br>• **root: 32909 (60.622%)**	• Cajanus cajan [taxid 3821]: 4 (0.007%)<br>• Dictyobacter alpinus [taxid 2014873]: 3 (0.005%)<br>• Pseudonocardia autotrophica [taxid 2074]: 3 (0.005%)<br>• Brandtodinium nutricula [taxid 1333877]: 3 (0.005%)<br>• Paenibacillus algorifonticola [taxid 684063]: 3 (0.005%)<br>• Amycolatopsis xylanica [taxid 589385]: 3 (0.005%)<br>• Lingulodinium polyedra [taxid 160621]: 2 (0.003%)<br>• Sorangium cellulosum [taxid 56]: 2 (0.003%)<br>• other: 174 (0.32%)
Benchmark OTU ID: `ENA|CM000488|CM000488.1-_Random`<br>OTU taxon: ENA|CM000488|CM000488.1-_Random [taxid 0]<br>Expected: unknown [taxid 1] (no rank)<br>Number of reads: 68268<br>Number of identified reads: 63396 (92.863%)	• species: 0 (0.0%)<br>• genus: 0 (0.0%)<br>• family: 0 (0.0%)<br>• order: 0 (0.0%)<br>• class: 0 (0.0%)<br>• phylum: 0 (0.0%)<br>• superkingdom: 0 (0.0%)<br>• **root: 62315 (91.279%)**	• Salmonella enterica [taxid 28901]: 5 (0.007%)<br>• Thalassionema nitzschioides [taxid 33649]: 3 (0.004%)<br>• Gymnoxanthella radiolariae [taxid 1798043]: 3 (0.004%)<br>• Dothistroma septosporum [taxid 64363]: 2 (0.002%)<br>• Enhygromyxa salina [taxid 215803]: 2 (0.002%)<br>• Nonlabens ponticola [taxid 2496866]: 2 (0.002%)<br>• Grammatophora oceanica [taxid 210454]: 2 (0.002%)<br>• Serendipita vermifera [taxid 109899]: 2 (0.002%)<br>• other: 195 (0.285%)
Benchmark OTU ID: `ENA|CM000657|CM000657.1-_Random`<br>OTU taxon: ENA|CM000657|CM000657.1-_Random [taxid 0]<br>Expected: unknown [taxid 1] (no rank)<br>Number of reads: 63842<br>Number of identified reads: 43637 (68.351%)	• species: 0 (0.0%)<br>• genus: 0 (0.0%)<br>• family: 0 (0.0%)<br>• order: 0 (0.0%)<br>• class: 0 (0.0%)<br>• phylum: 0 (0.0%)<br>• superkingdom: 0 (0.0%)<br>• **root: 43227 (67.709%)**	• Anaeromyces robustus [taxid 1754192]: 3 (0.004%)<br>• Amphimedon queenslandica [taxid 400682]: 2 (0.003%)<br>• Halalkalibacillus sediminis [taxid 2018042]: 2 (0.003%)<br>• Drosophila ananassae [taxid 7217]: 2 (0.003%)<br>• Naumovozyma dairenensis [taxid 27289]: 2 (0.003%)<br>• Schizosaccharomyces octosporus [taxid 4899]: 2 (0.003%)<br>• Helicobacter magdeburgensis [taxid 471858]: 2 (0.003%)<br>• Lachnospira multipara [taxid 28051]: 2 (0.003%)<br>• other: 112 (0.175%)

Operational Taxonomic Unit (OTU)	Correct identifications	Wrong or overspecific identifications at species rank
Benchmark OTU ID:<br>`ENA|CM000715|CM000715.1-_Random`<br>OTU taxon:<br>ENA|CM000715|CM000715.1-_Random [taxid 0]<br>Expected: unknown [taxid 1] (no rank)<br>Number of reads: 94496<br>Number of identified reads: 79374 (83.997%)	• species: 0 (0.0%)<br>• genus: 0 (0.0%)<br>• family: 0 (0.0%)<br>• order: 0 (0.0%)<br>• class: 0 (0.0%)<br>• phylum: 0 (0.0%)<br>• superkingdom: 0 (0.0%)<br>• **root: 78493 (83.064%)**	• Butyrivibrio fibrisolvens [taxid 831]: 4 (0.004%)<br>• Karenia brevis [taxid 156230]: 4 (0.004%)<br>• Cottoperca gobio [taxid 56716]: 3 (0.003%)<br>• Ekhidna lutea [taxid 447679]: 3 (0.003%)<br>• Streptococcus suis [taxid 1307]: 3 (0.003%)<br>• Faecalicoccus pleomorphus [taxid 1323]: 3 (0.003%)<br>• Erythranthe guttata [taxid 4155]: 2 (0.002%)<br>• Phocaeicola sartorii [taxid 671267]: 2 (0.002%)<br>• other: 202 (0.213%)
Benchmark OTU ID:<br>`ENA|CM000724|CM000724.1-_Random`<br>OTU taxon:<br>ENA|CM000724|CM000724.1-_Random [taxid 0]<br>Expected: unknown [taxid 1] (no rank)<br>Number of reads: 88712<br>Number of identified reads: 75433 (85.031%)	• species: 0 (0.0%)<br>• genus: 0 (0.0%)<br>• family: 0 (0.0%)<br>• order: 0 (0.0%)<br>• class: 0 (0.0%)<br>• phylum: 0 (0.0%)<br>• superkingdom: 0 (0.0%)<br>• **root: 74622 (84.117%)**	• Amycolatopsis coloradensis [taxid 76021]: 5 (0.005%)<br>• Lactuca saligna [taxid 75948]: 4 (0.004%)<br>• Metschnikowia bicuspidata [taxid 27322]: 4 (0.004%)<br>• Acetobacter tropicalis [taxid 104102]: 4 (0.004%)<br>• Scophthalmus maximus [taxid 52904]: 3 (0.003%)<br>• Heterocapsa arctica [taxid 192219]: 3 (0.003%)<br>• Tritrichomonas foetus [taxid 1144522]: 3 (0.003%)<br>• Strongylocentrotus purpuratus [taxid 7668]: 3 (0.003%)<br>• other: 189 (0.213%)
Benchmark OTU ID:<br>`ENA|CM000731|CM000731.1-_Random`<br>OTU taxon:<br>ENA|CM000731|CM000731.1-_Random [taxid 0]<br>Expected: unknown [taxid 1] (no rank)<br>Number of reads: 94413<br>Number of identified reads: 79440 (84.14%)	• species: 0 (0.0%)<br>• genus: 0 (0.0%)<br>• family: 0 (0.0%)<br>• order: 0 (0.0%)<br>• class: 0 (0.0%)<br>• phylum: 0 (0.0%)<br>• superkingdom: 0 (0.0%)<br>• **root: 78574 (83.223%)**	• Rotavirus A [taxid 28875]: 4 (0.004%)<br>• Tychonema bourrellyi [taxid 54313]: 3 (0.003%)<br>• Herpetosiphon geysericola [taxid 70996]: 3 (0.003%)<br>• Chara braunii [taxid 69332]: 3 (0.003%)<br>• Sparganum proliferum [taxid 64606]: 3 (0.003%)<br>• Marinobacter zhejiangensis [taxid 488535]: 3 (0.003%)<br>• Suricata suricatta [taxid 37032]: 3 (0.003%)<br>• Plasmopara halstedii [taxid 4781]: 3 (0.003%)<br>• other: 192 (0.203%)
Benchmark OTU ID:<br>`ENA|CM000750|CM000750.1-_Random`<br>OTU taxon:<br>ENA|CM000750|CM000750.1-_Random [taxid 0]<br>Expected: unknown [taxid 1] (no rank)<br>Number of reads: 90572<br>Number of identified reads: 76512 (84.476%)	• species: 0 (0.0%)<br>• genus: 0 (0.0%)<br>• family: 0 (0.0%)<br>• order: 0 (0.0%)<br>• class: 0 (0.0%)<br>• phylum: 0 (0.0%)<br>• superkingdom: 0 (0.0%)<br>• **root: 75663 (83.539%)**	• Rhodococcus jostii [taxid 132919]: 3 (0.003%)<br>• Methanobrevibacter curvatus [taxid 49547]: 3 (0.003%)<br>• Karenia brevis [taxid 156230]: 3 (0.003%)<br>• Candidatus Hepatoplasma crinochetorum [taxid 295596]: 3 (0.003%)<br>• Karlodinium veneficum [taxid 407301]: 3 (0.003%)<br>• Candidatus Woesearchaeota archaeon [taxid 2026803]: 2 (0.002%)<br>• Diaphanoeca grandis [taxid 28014]: 2 (0.002%)<br>• Lactuca sativa [taxid 4236]: 2 (0.002%)<br>• other: 182 (0.2%)

Operational Taxonomic Unit (OTU)	Correct identifications	Wrong or overspecific identifications at species rank
Benchmark OTU ID: `ENA|CM000754|CM000754.1-_Random`<br>OTU taxon: ENA|CM000754|CM000754.1-_Random [taxid 0]<br>Expected: unknown [taxid 1] (no rank)<br>Number of reads: 86816<br>Number of identified reads: 73333 (84.469%)	• species: 0 (0.0%)<br>• genus: 0 (0.0%)<br>• family: 0 (0.0%)<br>• order: 0 (0.0%)<br>• class: 0 (0.0%)<br>• phylum: 0 (0.0%)<br>• superkingdom: 0 (0.0%)<br>• **root: 72590 (83.613%)**	• Curtobacterium citreum [taxid 2036]: 4 (0.004%)<br>• Candidatus Aenigmarchaeota archaeon [taxid 2093792]: 4 (0.004%)<br>• Bartonella apis [taxid 1686310]: 3 (0.003%)<br>• Tanacetum cinerariifolium [taxid 118510]: 3 (0.003%)<br>• Brachypodium distachyon [taxid 15368]: 3 (0.003%)<br>• Anopheles darlingi [taxid 43151]: 3 (0.003%)<br>• Attheya septentrionalis [taxid 420275]: 3 (0.003%)<br>• Blastopirellula cremea [taxid 1031537]: 2 (0.002%)<br>• other: 176 (0.202%)
Benchmark OTU ID: `CP000097-_Random`<br>OTU taxon: CP000097-_Random [taxid 0]<br>Expected: unknown [taxid 1] (no rank)<br>Number of reads: 34051<br>Number of identified reads: 30571 (89.78%)	• species: 0 (0.0%)<br>• genus: 0 (0.0%)<br>• family: 0 (0.0%)<br>• order: 0 (0.0%)<br>• class: 0 (0.0%)<br>• phylum: 0 (0.0%)<br>• superkingdom: 0 (0.0%)<br>• **root: 29757 (87.389%)**	• Actinorugispora endophytica [taxid 1605990]: 5 (0.014%)<br>• Ophiocordyceps polyrhachis-furcata [taxid 1330020]: 2 (0.005%)<br>• Theileria equi [taxid 5872]: 2 (0.005%)<br>• Methylophaga frappieri [taxid 754477]: 2 (0.005%)<br>• Antricoccus suffuscus [taxid 1629062]: 2 (0.005%)<br>• Spodoptera frugiperda [taxid 7108]: 2 (0.005%)<br>• Klenkia marina [taxid 1960309]: 2 (0.005%)<br>• Camellia sinensis [taxid 4442]: 2 (0.005%)<br>• other: 102 (0.299%)
Benchmark OTU ID: `CP000108-_Random`<br>OTU taxon: CP000108-_Random [taxid 0]<br>Expected: unknown [taxid 1] (no rank)<br>Number of reads: 39901<br>Number of identified reads: 37134 (93.065%)	• species: 0 (0.0%)<br>• genus: 0 (0.0%)<br>• family: 0 (0.0%)<br>• order: 0 (0.0%)<br>• class: 0 (0.0%)<br>• phylum: 0 (0.0%)<br>• superkingdom: 0 (0.0%)<br>• **root: 36530 (91.551%)**	• Thiospirochaeta perfilievii [taxid 252967]: 4 (0.01%)<br>• Pedobacter rhizosphaerae [taxid 390241]: 3 (0.007%)<br>• Limimonas halophila [taxid 1082479]: 3 (0.007%)<br>• Algoriphagus boritolerans [taxid 308111]: 2 (0.005%)<br>• Phytophthora palmivora [taxid 4796]: 2 (0.005%)<br>• Micromonospora humi [taxid 745366]: 2 (0.005%)<br>• Aerococcus phage vB_AviM_AVP [taxid 2495576]: 2 (0.005%)<br>• Percolomonas cosmopolitus [taxid 63605]: 2 (0.005%)<br>• other: 88 (0.22%)
Benchmark OTU ID: `CP000154-_Random`<br>OTU taxon: CP000154-_Random [taxid 0]<br>Expected: unknown [taxid 1] (no rank)<br>Number of reads: 88877<br>Number of identified reads: 83021 (93.411%)	• species: 0 (0.0%)<br>• genus: 0 (0.0%)<br>• family: 0 (0.0%)<br>• order: 0 (0.0%)<br>• class: 0 (0.0%)<br>• phylum: 0 (0.0%)<br>• superkingdom: 0 (0.0%)<br>• **root: 81466 (91.661%)**	• Paracoccus sulfuroxidans [taxid 384678]: 4 (0.004%)<br>• Naasia lichenicola [taxid 2565933]: 4 (0.004%)<br>• Gaeumannomyces tritici [taxid 36779]: 4 (0.004%)<br>• Mycolicibacterium moriokaense [taxid 39691]: 3 (0.003%)<br>• Brugia pahangi [taxid 6280]: 3 (0.003%)<br>• Karlodinium veneficum [taxid 407301]: 3 (0.003%)<br>• Rhodopseudomonas faecalis [taxid 99655]: 3 (0.003%)<br>• Talaromyces islandicus [taxid 28573]: 3 (0.003%)<br>• other: 246 (0.276%)

Operational Taxonomic Unit (OTU)	Correct identifications	Wrong or overspecific identifications at species rank
Benchmark OTU ID: `CP000250-_Random`<br>OTU taxon: CP000250-_Random [taxid 0]<br>Expected: unknown [taxid 1] (no rank)<br>Number of reads: 87780<br>Number of identified reads: 57984 (66.056%)	• species: 0 (0.0%)<br>• genus: 0 (0.0%)<br>• family: 0 (0.0%)<br>• order: 0 (0.0%)<br>• class: 0 (0.0%)<br>• phylum: 0 (0.0%)<br>• superkingdom: 0 (0.0%)<br>• **root: 55274 (62.968%)**	• Lingulodinium polyedra [taxid 160621]: 4 (0.004%)<br>• Neonectria ditissima [taxid 78410]: 3 (0.003%)<br>• Nonomuraea gerenzanensis [taxid 93944]: 3 (0.003%)<br>• Ictalurus punctatus [taxid 7998]: 3 (0.003%)<br>• Aureobasidium pullulans [taxid 5580]: 3 (0.003%)<br>• Kipferlia bialata [taxid 797122]: 3 (0.003%)<br>• Armillaria solidipes [taxid 1076256]: 3 (0.003%)<br>• Danio rerio [taxid 7955]: 3 (0.003%)<br>• other: 258 (0.293%)
Benchmark OTU ID: `CP000312-_Random`<br>OTU taxon: CP000312-_Random [taxid 0]<br>Expected: unknown [taxid 1] (no rank)<br>Number of reads: 45545<br>Number of identified reads: 30770 (67.559%)	• species: 0 (0.0%)<br>• genus: 0 (0.0%)<br>• family: 0 (0.0%)<br>• order: 0 (0.0%)<br>• class: 0 (0.0%)<br>• phylum: 0 (0.0%)<br>• superkingdom: 0 (0.0%)<br>• **root: 30458 (66.874%)**	• Pyramimonas parkeae [taxid 36894]: 3 (0.006%)<br>• Phytophthora sojae [taxid 67593]: 2 (0.004%)<br>• Rhodopila globiformis [taxid 1071]: 2 (0.004%)<br>• Fusarium mangiferae [taxid 192010]: 2 (0.004%)<br>• Xenopus tropicalis [taxid 8364]: 2 (0.004%)<br>• Teratosphaeria nubilosa [taxid 161662]: 2 (0.004%)<br>• Drosophila navojoa [taxid 7232]: 2 (0.004%)<br>• Schizosaccharomyces pombe [taxid 4896]: 1 (0.002%)<br>• other: 79 (0.173%)
Benchmark OTU ID: `CP000381-_Random`<br>OTU taxon: CP000381-_Random [taxid 0]<br>Expected: unknown [taxid 1] (no rank)<br>Number of reads: 13099<br>Number of identified reads: 11980 (91.457%)	• species: 0 (0.0%)<br>• genus: 0 (0.0%)<br>• family: 0 (0.0%)<br>• order: 0 (0.0%)<br>• class: 0 (0.0%)<br>• phylum: 0 (0.0%)<br>• superkingdom: 0 (0.0%)<br>• **root: 11642 (88.877%)**	• Spinacia oleracea [taxid 3562]: 4 (0.03%)<br>• Esox lucius [taxid 8010]: 2 (0.015%)<br>• Mantoniella antarctica [taxid 81844]: 2 (0.015%)<br>• Prevotella buccae [taxid 28126]: 1 (0.007%)<br>• Nonomuraea wenchangensis [taxid 568860]: 1 (0.007%)<br>• Bracon brevicornis [taxid 1563983]: 1 (0.007%)<br>• Strongylocentrotus purpuratus [taxid 7668]: 1 (0.007%)<br>• Bdellovibrio sp. ArHS [taxid 1569284]: 1 (0.007%)<br>• other: 44 (0.335%)
Benchmark OTU ID: `CP000414-_Random`<br>OTU taxon: CP000414-_Random [taxid 0]<br>Expected: unknown [taxid 1] (no rank)<br>Number of reads: 30642<br>Number of identified reads: 27300 (89.093%)	• species: 0 (0.0%)<br>• genus: 0 (0.0%)<br>• family: 0 (0.0%)<br>• order: 0 (0.0%)<br>• class: 0 (0.0%)<br>• phylum: 0 (0.0%)<br>• superkingdom: 0 (0.0%)<br>• **root: 26987 (88.071%)**	• Ananas comosus [taxid 4615]: 3 (0.009%)<br>• Angiostrongylus costaricensis [taxid 334426]: 3 (0.009%)<br>• Azospirillum brasilense [taxid 192]: 2 (0.006%)<br>• Streptomyces niveus [taxid 193462]: 2 (0.006%)<br>• Gymnopilus dilepis [taxid 231916]: 2 (0.006%)<br>• Ensete ventricosum [taxid 4639]: 2 (0.006%)<br>• Fusobacterium varium [taxid 856]: 1 (0.003%)<br>• Rubrobacter xylanophilus [taxid 49319]: 1 (0.003%)<br>• other: 60 (0.195%)

Operational Taxonomic Unit (OTU)	Correct identifications	Wrong or overspecific identifications at species rank
Benchmark OTU ID: `CP000462-_Random`<br>OTU taxon: CP000462-_Random [taxid 0]<br>Expected: unknown [taxid 1] (no rank)<br>Number of reads: 77592<br>Number of identified reads: 58781 (75.756%)	• species: 0 (0.0%)<br>• genus: 0 (0.0%)<br>• family: 0 (0.0%)<br>• order: 0 (0.0%)<br>• class: 0 (0.0%)<br>• phylum: 0 (0.0%)<br>• superkingdom: 0 (0.0%)<br>• **root: 56614 (72.963%)**	• Galleria mellonella [taxid 7137]: 5 (0.006%)<br>• Bernardetia litoralis [taxid 999]: 3 (0.003%)<br>• Eimeria necatrix [taxid 51315]: 3 (0.003%)<br>• Emiliania huxleyi [taxid 2903]: 3 (0.003%)<br>• Actinomyces bowdenii [taxid 131109]: 3 (0.003%)<br>• Trypanosoma congolense [taxid 5692]: 3 (0.003%)<br>• Vitis vinifera [taxid 29760]: 3 (0.003%)<br>• Chrysochromulina rotalis [taxid 412157]: 2 (0.002%)<br>• other: 250 (0.322%)
Benchmark OTU ID: `CP000480-_Random`<br>OTU taxon: CP000480-_Random [taxid 0]<br>Expected: unknown [taxid 1] (no rank)<br>Number of reads: 116522<br>Number of identified reads: 72879 (62.545%)	• species: 0 (0.0%)<br>• genus: 0 (0.0%)<br>• family: 0 (0.0%)<br>• order: 0 (0.0%)<br>• class: 0 (0.0%)<br>• phylum: 0 (0.0%)<br>• superkingdom: 0 (0.0%)<br>• **root: 69181 (59.371%)**	• Dinophysis acuminata [taxid 47934]: 5 (0.004%)<br>• Enhygromyxa salina [taxid 215803]: 4 (0.003%)<br>• Karenia brevis [taxid 156230]: 4 (0.003%)<br>• Scrippsiella trochoidea [taxid 71861]: 3 (0.002%)<br>• Pseudobutyrivibrio ruminis [taxid 46206]: 3 (0.002%)<br>• Aspergillus aculeatus [taxid 5053]: 2 (0.001%)<br>• Fagus sylvatica [taxid 28930]: 2 (0.001%)<br>• Dendrothele bispora [taxid 1314803]: 2 (0.001%)<br>• other: 312 (0.267%)
Benchmark OTU ID: `CP000512-_Random`<br>OTU taxon: CP000512-_Random [taxid 0]<br>Expected: unknown [taxid 1] (no rank)<br>Number of reads: 88146<br>Number of identified reads: 56343 (63.92%)	• species: 0 (0.0%)<br>• genus: 0 (0.0%)<br>• family: 0 (0.0%)<br>• order: 0 (0.0%)<br>• class: 0 (0.0%)<br>• phylum: 0 (0.0%)<br>• superkingdom: 0 (0.0%)<br>• **root: 52504 (59.564%)**	• Alexandrium monilatum [taxid 311494]: 3 (0.003%)<br>• Daphnia pulex [taxid 6669]: 3 (0.003%)<br>• Pelagodinium beii [taxid 43686]: 3 (0.003%)<br>• Candidatus Bathyarchaeota archaeon [taxid 2026714]: 3 (0.003%)<br>• Colletotrichum simmondsii [taxid 703756]: 3 (0.003%)<br>• Afipia massiliensis [taxid 211460]: 3 (0.003%)<br>• Olsenella uli [taxid 133926]: 3 (0.003%)<br>• Oryza meyeriana [taxid 83307]: 3 (0.003%)<br>• other: 299 (0.339%)
Benchmark OTU ID: `CP000521-_Random`<br>OTU taxon: CP000521-_Random [taxid 0]<br>Expected: unknown [taxid 1] (no rank)<br>Number of reads: 64270<br>Number of identified reads: 57611 (89.639%)	• species: 0 (0.0%)<br>• genus: 0 (0.0%)<br>• family: 0 (0.0%)<br>• order: 0 (0.0%)<br>• class: 0 (0.0%)<br>• phylum: 0 (0.0%)<br>• superkingdom: 0 (0.0%)<br>• **root: 56887 (88.512%)**	• Galdieria sulphuraria [taxid 130081]: 5 (0.007%)<br>• Odontella aurita [taxid 265563]: 4 (0.006%)<br>• Prevotella copri [taxid 165179]: 4 (0.006%)<br>• Paenibacillus glucanolyticus [taxid 59843]: 3 (0.004%)<br>• Aureimonas jatrophae [taxid 1166073]: 3 (0.004%)<br>• Capronia epimyces [taxid 43228]: 3 (0.004%)<br>• Toxocara canis [taxid 6265]: 3 (0.004%)<br>• Striatella unipunctata [taxid 210618]: 2 (0.003%)<br>• other: 165 (0.256%)

Operational Taxonomic Unit (OTU)	Correct identifications	Wrong or overspecific identifications at species rank
Benchmark OTU ID: `CP000529-_Random`<br>OTU taxon: CP000529-_Random [taxid 0]<br>Expected: unknown [taxid 1] (no rank)<br>Number of reads: 71794<br>Number of identified reads: 53472 (74.479%)	• species: 0 (0.0%)<br>• genus: 0 (0.0%)<br>• family: 0 (0.0%)<br>• order: 0 (0.0%)<br>• class: 0 (0.0%)<br>• phylum: 0 (0.0%)<br>• superkingdom: 0 (0.0%)<br>• **root: 51357 (71.533%)**	• Lysinibacillus telephonicus [taxid 1714840]: 4 (0.005%)<br>• Brandtodinium nutricula [taxid 1333877]: 3 (0.004%)<br>• Nakamurella silvestris [taxid 1645681]: 3 (0.004%)<br>• Sinirhodobacter ferrireducens [taxid 1215032]: 3 (0.004%)<br>• Actinotalea fermentans [taxid 43671]: 3 (0.004%)<br>• Tanacetum cinerariifolium [taxid 118510]: 3 (0.004%)<br>• Ruminiclostridium sufflavum [taxid 396504]: 2 (0.002%)<br>• Ensete ventricosum [taxid 4639]: 2 (0.002%)<br>• other: 226 (0.314%)
Benchmark OTU ID: `CP000577-_Random`<br>OTU taxon: CP000577-_Random [taxid 0]<br>Expected: unknown [taxid 1] (no rank)<br>Number of reads: 49890<br>Number of identified reads: 30981 (62.098%)	• species: 0 (0.0%)<br>• genus: 0 (0.0%)<br>• family: 0 (0.0%)<br>• order: 0 (0.0%)<br>• class: 0 (0.0%)<br>• phylum: 0 (0.0%)<br>• superkingdom: 0 (0.0%)<br>• **root: 28834 (57.795%)**	• Pseudomonas aeruginosa [taxid 287]: 3 (0.006%)<br>• Sorangium cellulosum [taxid 56]: 3 (0.006%)<br>• Ustilaginoidea virens [taxid 1159556]: 2 (0.004%)<br>• Pelagodinium beii [taxid 43686]: 2 (0.004%)<br>• Colletotrichum higginsianum [taxid 80884]: 2 (0.004%)<br>• Symbiodinium microadriaticum [taxid 2951]: 2 (0.004%)<br>• Achlya hypogyna [taxid 1202772]: 2 (0.004%)<br>• Volvox carteri [taxid 3067]: 2 (0.004%)<br>• other: 166 (0.332%)
Benchmark OTU ID: `CP000792-_Random`<br>OTU taxon: CP000792-_Random [taxid 0]<br>Expected: unknown [taxid 1] (no rank)<br>Number of reads: 30879<br>Number of identified reads: 27865 (90.239%)	• species: 0 (0.0%)<br>• genus: 0 (0.0%)<br>• family: 0 (0.0%)<br>• order: 0 (0.0%)<br>• class: 0 (0.0%)<br>• phylum: 0 (0.0%)<br>• superkingdom: 0 (0.0%)<br>• **root: 27508 (89.083%)**	• Paramuricea clavata [taxid 317549]: 4 (0.012%)<br>• Lophiostoma macrostomum [taxid 372055]: 3 (0.009%)<br>• Penicillium digitatum [taxid 36651]: 3 (0.009%)<br>• Rheinheimera tuosuensis [taxid 1323745]: 2 (0.006%)<br>• Piloderma croceum [taxid 80663]: 2 (0.006%)<br>• Anopheles maculatus [taxid 74869]: 1 (0.003%)<br>• Taibaiella soli [taxid 1649169]: 1 (0.003%)<br>• Colletotrichum fioriniae [taxid 710243]: 1 (0.003%)<br>• other: 55 (0.178%)
Benchmark OTU ID: `CP000822-_Random`<br>OTU taxon: CP000822-_Random [taxid 0]<br>Expected: unknown [taxid 1] (no rank)<br>Number of reads: 77176<br>Number of identified reads: 70230 (90.999%)	• species: 0 (0.0%)<br>• genus: 0 (0.0%)<br>• family: 0 (0.0%)<br>• order: 0 (0.0%)<br>• class: 0 (0.0%)<br>• phylum: 0 (0.0%)<br>• superkingdom: 0 (0.0%)<br>• **root: 68363 (88.58%)**	• Planctomicrobium piriforme [taxid 1576369]: 3 (0.003%)<br>• Exidia glandulosa [taxid 5219]: 3 (0.003%)<br>• Phialocephala subalpina [taxid 576137]: 3 (0.003%)<br>• Leptomonas seymouri [taxid 5684]: 3 (0.003%)<br>• Fragilariopsis cylindrus [taxid 186039]: 3 (0.003%)<br>• Macrostomum lignano [taxid 282301]: 3 (0.003%)<br>• Aspergillus saccharolyticus [taxid 979771]: 3 (0.003%)<br>• Tetradesmus obliquus [taxid 3088]: 3 (0.003%)<br>• other: 268 (0.347%)

Operational Taxonomic Unit (OTU)	Correct identifications	Wrong or overspecific identifications at species rank
Benchmark OTU ID: `CP000825-_Random`<br>OTU taxon: CP000825-_Random [taxid 0]<br>Expected: unknown [taxid 1] (no rank)<br>Number of reads: 25443<br>Number of identified reads: 19465 (76.504%)	• species: 0 (0.0%)<br>• genus: 0 (0.0%)<br>• family: 0 (0.0%)<br>• order: 0 (0.0%)<br>• class: 0 (0.0%)<br>• phylum: 0 (0.0%)<br>• superkingdom: 0 (0.0%)<br>• **root: 19299 (75.851%)**	• Pediculus humanus [taxid 121225]: 2 (0.007%)<br>• Candidatus Carsonella ruddii [taxid 114186]: 2 (0.007%)<br>• Stylonychia lemnae [taxid 5949]: 2 (0.007%)<br>• Achlya hypogyna [taxid 1202772]: 2 (0.007%)<br>• Solanum tuberosum [taxid 4113]: 1 (0.003%)<br>• Agaricus bisporus [taxid 5341]: 1 (0.003%)<br>• Myroides odoratus [taxid 256]: 1 (0.003%)<br>• Chlorarachnion reptans [taxid 29199]: 1 (0.003%)<br>• other: 38 (0.149%)
Benchmark OTU ID: `CP000911-_Random`<br>OTU taxon: CP000911-_Random [taxid 0]<br>Expected: unknown [taxid 1] (no rank)<br>Number of reads: 28653<br>Number of identified reads: 25237 (88.078%)	• species: 0 (0.0%)<br>• genus: 0 (0.0%)<br>• family: 0 (0.0%)<br>• order: 0 (0.0%)<br>• class: 0 (0.0%)<br>• phylum: 0 (0.0%)<br>• superkingdom: 0 (0.0%)<br>• **root: 24460 (85.366%)**	• Salmonella enterica [taxid 28901]: 3 (0.01%)<br>• Embleya scabrispora [taxid 159449]: 2 (0.006%)<br>• Candidatus Methylospira mobilis [taxid 1808979]: 2 (0.006%)<br>• Tetrabaena socialis [taxid 47790]: 2 (0.006%)<br>• Natrarchaeobaculum sulfurireducens [taxid 2044521]: 2 (0.006%)<br>• Phascolarctos cinereus [taxid 38626]: 2 (0.006%)<br>• Rhodococcus erythropolis [taxid 1833]: 2 (0.006%)<br>• Tanacetum cinerariifolium [taxid 118510]: 2 (0.006%)<br>• other: 98 (0.342%)
Benchmark OTU ID: `CP000915-_Random`<br>OTU taxon: CP000915-_Random [taxid 0]<br>Expected: unknown [taxid 1] (no rank)<br>Number of reads: 28134<br>Number of identified reads: 22257 (79.11%)	• species: 0 (0.0%)<br>• genus: 0 (0.0%)<br>• family: 0 (0.0%)<br>• order: 0 (0.0%)<br>• class: 0 (0.0%)<br>• phylum: 0 (0.0%)<br>• superkingdom: 0 (0.0%)<br>• **root: 22067 (78.435%)**	• Lupinus albus [taxid 3870]: 3 (0.01%)<br>• Halogeometricum pallidum [taxid 411361]: 2 (0.007%)<br>• Karenia brevis [taxid 156230]: 2 (0.007%)<br>• Cicer arietinum [taxid 3827]: 2 (0.007%)<br>• Trichinella patagoniensis [taxid 990121]: 2 (0.007%)<br>• Solanum lycopersicum [taxid 4081]: 2 (0.007%)<br>• Eubacterium limosum [taxid 1736]: 2 (0.007%)<br>• Treponema socranskii [taxid 53419]: 1 (0.003%)<br>• other: 39 (0.138%)
Benchmark OTU ID: `CP000923-_Random`<br>OTU taxon: CP000923-_Random [taxid 0]<br>Expected: unknown [taxid 1] (no rank)<br>Number of reads: 37909<br>Number of identified reads: 31258 (82.455%)	• species: 0 (0.0%)<br>• genus: 0 (0.0%)<br>• family: 0 (0.0%)<br>• order: 0 (0.0%)<br>• class: 0 (0.0%)<br>• phylum: 0 (0.0%)<br>• superkingdom: 0 (0.0%)<br>• **root: 30948 (81.637%)**	• Stephanopyxis turris [taxid 515487]: 3 (0.007%)<br>• Silvanigrella paludirubra [taxid 2499159]: 2 (0.005%)<br>• Ignatzschineria cameli [taxid 2182793]: 2 (0.005%)<br>• Gymnoxanthella radiolariae [taxid 1798043]: 2 (0.005%)<br>• Phaseolus vulgaris [taxid 3885]: 2 (0.005%)<br>• Araucaria cunninghamii [taxid 56994]: 2 (0.005%)<br>• Human immunodeficiency virus 1 [taxid 11676]: 2 (0.005%)<br>• Paenibacillus oralis [taxid 2490856]: 2 (0.005%)<br>• other: 78 (0.205%)

Operational Taxonomic Unit (OTU)	Correct identifications	Wrong or overspecific identifications at species rank
Benchmark OTU ID: `CP000930-_Random`<br>OTU taxon: CP000930-_Random [taxid 0]<br>Expected: unknown [taxid 1] (no rank)<br>Number of reads: 48635<br>Number of identified reads: 41815 (85.977%)	• species: 0 (0.0%)<br>• genus: 0 (0.0%)<br>• family: 0 (0.0%)<br>• order: 0 (0.0%)<br>• class: 0 (0.0%)<br>• phylum: 0 (0.0%)<br>• superkingdom: 0 (0.0%)<br>• **root: 40553 (83.382%)**	• Puccinia graminis [taxid 5297]: 3 (0.006%)<br>• Nonomuraea polychroma [taxid 46176]: 3 (0.006%)<br>• Acidithiobacillus sulfuriphilus [taxid 1867749]: 3 (0.006%)<br>• Lingulodinium polyedra [taxid 160621]: 2 (0.004%)<br>• Teratosphaeria nubilosa [taxid 161662]: 2 (0.004%)<br>• Pseudomonas hussainii [taxid 1429083]: 2 (0.004%)<br>• Amphidinium massartii [taxid 160604]: 2 (0.004%)<br>• Mucilaginibacter frigoritolerans [taxid 652788]: 2 (0.004%)<br>• other: 144 (0.296%)
Benchmark OTU ID: `CP000943-_Random`<br>OTU taxon: CP000943-_Random [taxid 0]<br>Expected: unknown [taxid 1] (no rank)<br>Number of reads: 128161<br>Number of identified reads: 85824 (66.965%)	• species: 0 (0.0%)<br>• genus: 0 (0.0%)<br>• family: 0 (0.0%)<br>• order: 0 (0.0%)<br>• class: 0 (0.0%)<br>• phylum: 0 (0.0%)<br>• superkingdom: 0 (0.0%)<br>• **root: 77088 (60.149%)**	• Besnoitia besnoiti [taxid 94643]: 5 (0.003%)<br>• Lingulodinium polyedra [taxid 160621]: 5 (0.003%)<br>• Prymnesium polylepis [taxid 72548]: 5 (0.003%)<br>• Tetraselmis striata [taxid 3165]: 4 (0.003%)<br>• Heterocapsa triquetra [taxid 66468]: 4 (0.003%)<br>• Pelagodinium beii [taxid 43686]: 3 (0.002%)<br>• Sediminihabitans luteus [taxid 1138585]: 3 (0.002%)<br>• Streptomyces antibioticus [taxid 1890]: 3 (0.002%)<br>• other: 645 (0.503%)
Benchmark OTU ID: `CP000962-_Random`<br>OTU taxon: CP000962-_Random [taxid 0]<br>Expected: unknown [taxid 1] (no rank)<br>Number of reads: 64554<br>Number of identified reads: 44074 (68.274%)	• species: 0 (0.0%)<br>• genus: 0 (0.0%)<br>• family: 0 (0.0%)<br>• order: 0 (0.0%)<br>• class: 0 (0.0%)<br>• phylum: 0 (0.0%)<br>• superkingdom: 0 (0.0%)<br>• **root: 43653 (67.622%)**	• Bimuria novae-zelandiae [taxid 147497]: 3 (0.004%)<br>• Clostridium niameyense [taxid 1622073]: 3 (0.004%)<br>• Winogradskyella arenosi [taxid 533325]: 3 (0.004%)<br>• Leuconostoc lactis [taxid 1246]: 2 (0.003%)<br>• Silvanigrella aquatica [taxid 1915309]: 2 (0.003%)<br>• Pochonia chlamydosporia [taxid 280754]: 2 (0.003%)<br>• Lepeophtheirus salmonis [taxid 72036]: 2 (0.003%)<br>• Vitis vinifera [taxid 29760]: 2 (0.003%)<br>• other: 102 (0.158%)
Benchmark OTU ID: `CP001019-_Random`<br>OTU taxon: CP001019-_Random [taxid 0]<br>Expected: unknown [taxid 1] (no rank)<br>Number of reads: 30130<br>Number of identified reads: 28004 (92.943%)	• species: 0 (0.0%)<br>• genus: 0 (0.0%)<br>• family: 0 (0.0%)<br>• order: 0 (0.0%)<br>• class: 0 (0.0%)<br>• phylum: 0 (0.0%)<br>• superkingdom: 0 (0.0%)<br>• **root: 27530 (91.37%)**	• Euryarchaeota archaeon [taxid 2026739]: 3 (0.009%)<br>• Herbidospora galbida [taxid 2575442]: 3 (0.009%)<br>• Candidatus Tenderia electrophaga [taxid 1748243]: 2 (0.006%)<br>• Bacteroides finegoldii [taxid 338188]: 2 (0.006%)<br>• Folsomia candida [taxid 158441]: 2 (0.006%)<br>• Flavihumibacter petaseus [taxid 549295]: 2 (0.006%)<br>• Botrytis hyacinthi [taxid 278943]: 2 (0.006%)<br>• Campylobacter concisus [taxid 199]: 2 (0.006%)<br>• other: 85 (0.282%)

Operational Taxonomic Unit (OTU)	Correct identifications	Wrong or overspecific identifications at species rank
Benchmark OTU ID: `CP001080-_Random`<br>OTU taxon: CP001080-_Random [taxid 0]<br>Expected: unknown [taxid 1] (no rank)<br>Number of reads: 27173<br>Number of identified reads: 21363 (78.618%)	• species: 0 (0.0%)<br>• genus: 0 (0.0%)<br>• family: 0 (0.0%)<br>• order: 0 (0.0%)<br>• class: 0 (0.0%)<br>• phylum: 0 (0.0%)<br>• superkingdom: 0 (0.0%)<br>• **root: 21141 (77.801%)**	• Desulfitobacterium dichloroeliminans [taxid 233055]: 3 (0.011%)<br>• Arundo donax [taxid 35708]: 2 (0.007%)<br>• Anaerotignum lactatifermentans [taxid 160404]: 2 (0.007%)<br>• Phaseolus vulgaris [taxid 3885]: 2 (0.007%)<br>• Lasallia pustulata [taxid 136370]: 2 (0.007%)<br>• Rhizobium tumorigenes [taxid 2041385]: 1 (0.003%)<br>• Lichtheimia ramosa [taxid 688394]: 1 (0.003%)<br>• Ophiobolus disseminans [taxid 1469910]: 1 (0.003%)<br>• other: 43 (0.158%)
Benchmark OTU ID: `CP001084-_Random`<br>OTU taxon: CP001084-_Random [taxid 0]<br>Expected: unknown [taxid 1] (no rank)<br>Number of reads: 44929<br>Number of identified reads: 42381 (94.328%)	• species: 0 (0.0%)<br>• genus: 0 (0.0%)<br>• family: 0 (0.0%)<br>• order: 0 (0.0%)<br>• class: 0 (0.0%)<br>• phylum: 0 (0.0%)<br>• superkingdom: 0 (0.0%)<br>• **root: 41568 (92.519%)**	• Candidatus Caldiarchaeum subterraneum [taxid 311458]: 3 (0.006%)<br>• Octopus vulgaris [taxid 6645]: 2 (0.004%)<br>• Candidatus Bathyarchaeota archaeon [taxid 2026714]: 2 (0.004%)<br>• Oleiphilus messinensis [taxid 141451]: 2 (0.004%)<br>• Duncaniella dubosii [taxid 2518971]: 2 (0.004%)<br>• Purpureocillium lilacinum [taxid 33203]: 2 (0.004%)<br>• Physcomitrium patens [taxid 3218]: 2 (0.004%)<br>• Babesia sp. Xinjiang [taxid 462227]: 2 (0.004%)<br>• other: 130 (0.289%)
Benchmark OTU ID: `CP001227-_Random`<br>OTU taxon: CP001227-_Random [taxid 0]<br>Expected: unknown [taxid 1] (no rank)<br>Number of reads: 17631<br>Number of identified reads: 14062 (79.757%)	• species: 0 (0.0%)<br>• genus: 0 (0.0%)<br>• family: 0 (0.0%)<br>• order: 0 (0.0%)<br>• class: 0 (0.0%)<br>• phylum: 0 (0.0%)<br>• superkingdom: 0 (0.0%)<br>• **root: 13916 (78.929%)**	• Euryarchaeota archaeon [taxid 2026739]: 2 (0.011%)<br>• Seonamhaeicola maritimus [taxid 2591822]: 2 (0.011%)<br>• Schaalia turicensis [taxid 131111]: 2 (0.011%)<br>• Methanococcoides burtonii [taxid 29291]: 1 (0.005%)<br>• Microbotryum intermedium [taxid 269621]: 1 (0.005%)<br>• Armillaria ostoyae [taxid 47428]: 1 (0.005%)<br>• Arachidicoccus rhizosphaerae [taxid 551991]: 1 (0.005%)<br>• Paenibacillus wynnii [taxid 268407]: 1 (0.005%)<br>• other: 34 (0.192%)
Benchmark OTU ID: `CP001391-_Random`<br>OTU taxon: CP001391-_Random [taxid 0]<br>Expected: unknown [taxid 1] (no rank)<br>Number of reads: 20362<br>Number of identified reads: 17301 (84.967%)	• species: 0 (0.0%)<br>• genus: 0 (0.0%)<br>• family: 0 (0.0%)<br>• order: 0 (0.0%)<br>• class: 0 (0.0%)<br>• phylum: 0 (0.0%)<br>• superkingdom: 0 (0.0%)<br>• **root: 17111 (84.033%)**	• Dokdonella immobilis [taxid 578942]: 2 (0.009%)<br>• Basidiobolus meristosporus [taxid 423460]: 2 (0.009%)<br>• Colletotrichum sublineola [taxid 1173701]: 2 (0.009%)<br>• Vigna unguiculata [taxid 3917]: 2 (0.009%)<br>• Physcomitrium patens [taxid 3218]: 1 (0.004%)<br>• [Clostridium] fimetarium [taxid 99656]: 1 (0.004%)<br>• Eucampia antarctica [taxid 49252]: 1 (0.004%)<br>• Nocardia brasiliensis [taxid 37326]: 1 (0.004%)<br>• other: 37 (0.181%)

Operational Taxonomic Unit (OTU)	Correct identifications	Wrong or overspecific identifications at species rank
Benchmark OTU ID: `CP001489-_Random`<br>OTU taxon: CP001489-_Random [taxid 0]<br>Expected: unknown [taxid 1] (no rank)<br>Number of reads: 15845<br>Number of identified reads: 13909 (87.781%)	• species: 0 (0.0%)<br>• genus: 0 (0.0%)<br>• family: 0 (0.0%)<br>• order: 0 (0.0%)<br>• class: 0 (0.0%)<br>• phylum: 0 (0.0%)<br>• superkingdom: 0 (0.0%)<br>• **root: 13449 (84.878%)**	• Lasallia pustulata [taxid 136370]: 2 (0.012%)<br>• Mycena chlorophos [taxid 658473]: 2 (0.012%)<br>• Candidatus Poseidoniales archaeon [taxid 2163009]: 2 (0.012%)<br>• Marinomonas communis [taxid 28254]: 2 (0.012%)<br>• Gossypium barbadense [taxid 3634]: 2 (0.012%)<br>• Marssonina brunnea [taxid 698440]: 1 (0.006%)<br>• Eisenbergiella massiliensis [taxid 1720294]: 1 (0.006%)<br>• Nanoarchaeota archaeon [taxid 2026764]: 1 (0.006%)<br>• other: 60 (0.378%)
Benchmark OTU ID: `CP001582-_Random`<br>OTU taxon: CP001582-_Random [taxid 0]<br>Expected: unknown [taxid 1] (no rank)<br>Number of reads: 22832<br>Number of identified reads: 20553 (90.018%)	• species: 0 (0.0%)<br>• genus: 0 (0.0%)<br>• family: 0 (0.0%)<br>• order: 0 (0.0%)<br>• class: 0 (0.0%)<br>• phylum: 0 (0.0%)<br>• superkingdom: 0 (0.0%)<br>• **root: 20302 (88.919%)**	• Polytolypa hystricis [taxid 221428]: 3 (0.013%)<br>• Phakopsora pachyrhizi [taxid 170000]: 2 (0.008%)<br>• Massarina eburnea [taxid 45293]: 2 (0.008%)<br>• Methylorubrum populi [taxid 223967]: 2 (0.008%)<br>• Lingula anatina [taxid 7574]: 2 (0.008%)<br>• Pelagodinium beii [taxid 43686]: 2 (0.008%)<br>• Coleofasciculus chthonoplastes [taxid 64178]: 1 (0.004%)<br>• Salmonella enterica [taxid 28901]: 1 (0.004%)<br>• other: 46 (0.201%)
Benchmark OTU ID: `CP001712-_Random`<br>OTU taxon: CP001712-_Random [taxid 0]<br>Expected: unknown [taxid 1] (no rank)<br>Number of reads: 56528<br>Number of identified reads: 50513 (89.359%)	• species: 0 (0.0%)<br>• genus: 0 (0.0%)<br>• family: 0 (0.0%)<br>• order: 0 (0.0%)<br>• class: 0 (0.0%)<br>• phylum: 0 (0.0%)<br>• superkingdom: 0 (0.0%)<br>• **root: 49034 (86.742%)**	• Paracoccus versutus [taxid 34007]: 3 (0.005%)<br>• Lingulodinium polyedra [taxid 160621]: 3 (0.005%)<br>• Ectocarpus siliculosus [taxid 2880]: 2 (0.003%)<br>• Micromonospora olivasterospora [taxid 1880]: 2 (0.003%)<br>• Chlamydomonas eustigma [taxid 1157962]: 2 (0.003%)<br>• Candidatus Protochlamydia amoebophila [taxid 362787]: 2 (0.003%)<br>• Candidatus Bathyarchaeota archaeon [taxid 2026714]: 2 (0.003%)<br>• Nothobranchius furzeri [taxid 105023]: 2 (0.003%)<br>• other: 157 (0.277%)
Benchmark OTU ID: `CP001802-_Random`<br>OTU taxon: CP001802-_Random [taxid 0]<br>Expected: unknown [taxid 1] (no rank)<br>Number of reads: 85646<br>Number of identified reads: 53702 (62.702%)	• species: 0 (0.0%)<br>• genus: 0 (0.0%)<br>• family: 0 (0.0%)<br>• order: 0 (0.0%)<br>• class: 0 (0.0%)<br>• phylum: 0 (0.0%)<br>• superkingdom: 0 (0.0%)<br>• **root: 51013 (59.562%)**	• Microbacterium wangchenii [taxid 2541726]: 4 (0.004%)<br>• Pseudomonas jinjuensis [taxid 198616]: 2 (0.002%)<br>• Xanthomonas campestris [taxid 339]: 2 (0.002%)<br>• Taeniopygia guttata [taxid 59729]: 2 (0.002%)<br>• Janthinobacterium violaceinigrum [taxid 2654252]: 2 (0.002%)<br>• Babjeviella inositovora [taxid 45609]: 2 (0.002%)<br>• Odontella aurita [taxid 265563]: 2 (0.002%)<br>• Brandtodinium nutricula [taxid 1333877]: 2 (0.002%)<br>• other: 218 (0.254%)

Operational Taxonomic Unit (OTU)	Correct identifications	Wrong or overspecific identifications at species rank
Benchmark OTU ID: `CP001805-_Random`<br>OTU taxon: CP001805-_Random [taxid 0]<br>Expected: unknown [taxid 1] (no rank)<br>Number of reads: 51829<br>Number of identified reads: 48707 (93.976%)	• species: 0 (0.0%)<br>• genus: 0 (0.0%)<br>• family: 0 (0.0%)<br>• order: 0 (0.0%)<br>• class: 0 (0.0%)<br>• phylum: 0 (0.0%)<br>• superkingdom: 0 (0.0%)<br>• **root: 47848 (92.318%)**	• Tanacetum cinerariifolium [taxid 118510]: 4 (0.007%)<br>• Rhodococcus kyotonensis [taxid 398843]: 3 (0.005%)<br>• Dictyobacter vulcani [taxid 2607529]: 3 (0.005%)<br>• Debaryomyces fabryi [taxid 58627]: 3 (0.005%)<br>• Prasinococcus capsulatus [taxid 156131]: 3 (0.005%)<br>• Chaetoceros neogracilis [taxid 240364]: 3 (0.005%)<br>• Testicularia cyperi [taxid 1882483]: 2 (0.003%)<br>• Euryarchaeota archaeon [taxid 2026739]: 2 (0.003%)<br>• other: 152 (0.293%)
Benchmark OTU ID: `CP001806-_Random`<br>OTU taxon: CP001806-_Random [taxid 0]<br>Expected: unknown [taxid 1] (no rank)<br>Number of reads: 27017<br>Number of identified reads: 25420 (94.088%)	• species: 0 (0.0%)<br>• genus: 0 (0.0%)<br>• family: 0 (0.0%)<br>• order: 0 (0.0%)<br>• class: 0 (0.0%)<br>• phylum: 0 (0.0%)<br>• superkingdom: 0 (0.0%)<br>• **root: 25015 (92.589%)**	• Coniophora puteana [taxid 80637]: 4 (0.014%)<br>• Legionella israelensis [taxid 454]: 3 (0.011%)<br>• Nocardia tenerifensis [taxid 228006]: 2 (0.007%)<br>• Euryarchaeota archaeon [taxid 2026739]: 2 (0.007%)<br>• Mobilicoccus pelagius [taxid 746032]: 2 (0.007%)<br>• Clunio marinus [taxid 568069]: 2 (0.007%)<br>• Clostridium baratii [taxid 1561]: 2 (0.007%)<br>• Dyella japonica [taxid 231455]: 2 (0.007%)<br>• other: 61 (0.225%)
Benchmark OTU ID: `CP001818-_Random`<br>OTU taxon: CP001818-_Random [taxid 0]<br>Expected: unknown [taxid 1] (no rank)<br>Number of reads: 27347<br>Number of identified reads: 19070 (69.733%)	• species: 0 (0.0%)<br>• genus: 0 (0.0%)<br>• family: 0 (0.0%)<br>• order: 0 (0.0%)<br>• class: 0 (0.0%)<br>• phylum: 0 (0.0%)<br>• superkingdom: 0 (0.0%)<br>• **root: 18300 (66.917%)**	• Achromobacter veterisilvae [taxid 2069367]: 3 (0.01%)<br>• Lyngbya aestuarii [taxid 118322]: 2 (0.007%)<br>• Sulfitobacter mediterraneus [taxid 83219]: 2 (0.007%)<br>• Croceicoccus marinus [taxid 450378]: 2 (0.007%)<br>• Fonticula alba [taxid 691883]: 2 (0.007%)<br>• Sorangium cellulosum [taxid 56]: 2 (0.007%)<br>• Sedimenticola selenatireducens [taxid 191960]: 2 (0.007%)<br>• Thermoproteus tenax [taxid 2271]: 2 (0.007%)<br>• other: 74 (0.27%)
Benchmark OTU ID: `CP001825-_Random`<br>OTU taxon: CP001825-_Random [taxid 0]<br>Expected: unknown [taxid 1] (no rank)<br>Number of reads: 30444<br>Number of identified reads: 28572 (93.851%)	• species: 0 (0.0%)<br>• genus: 0 (0.0%)<br>• family: 0 (0.0%)<br>• order: 0 (0.0%)<br>• class: 0 (0.0%)<br>• phylum: 0 (0.0%)<br>• superkingdom: 0 (0.0%)<br>• **root: 28007 (91.995%)**	• Eucalyptus grandis [taxid 71139]: 2 (0.006%)<br>• Capsaspora owczarzaki [taxid 192875]: 2 (0.006%)<br>• Amborella trichopoda [taxid 13333]: 2 (0.006%)<br>• Nematostella vectensis [taxid 45351]: 2 (0.006%)<br>• Hirschia baltica [taxid 2724]: 2 (0.006%)<br>• Metschnikowia aff. pulcherrima [taxid 2163413]: 2 (0.006%)<br>• Anopheles maculatus [taxid 74869]: 2 (0.006%)<br>• Thermaurantimonas aggregans [taxid 2173829]: 2 (0.006%)<br>• other: 86 (0.282%)

Operational Taxonomic Unit (OTU)	Correct identifications	Wrong or overspecific identifications at species rank
Benchmark OTU ID: `CP001829-_Random`<br>OTU taxon: CP001829-_Random [taxid 0]<br>Expected: unknown [taxid 1] (no rank)<br>Number of reads: 35671<br>Number of identified reads: 33263 (93.249%)	• species: 0 (0.0%)<br>• genus: 0 (0.0%)<br>• family: 0 (0.0%)<br>• order: 0 (0.0%)<br>• class: 0 (0.0%)<br>• phylum: 0 (0.0%)<br>• superkingdom: 0 (0.0%)<br>• **root: 32402 (90.835%)**	• Eutypa lata [taxid 97096]: 4 (0.011%)<br>• Acinetobacter puyangensis [taxid 1096779]: 4 (0.011%)<br>• Scrippsiella trochoidea [taxid 71861]: 3 (0.008%)<br>• Biomphalaria glabrata [taxid 6526]: 3 (0.008%)<br>• Piromyces finnis [taxid 1754191]: 3 (0.008%)<br>• Euryarchaeota archaeon [taxid 2026739]: 2 (0.005%)<br>• Scleropages formosus [taxid 113540]: 2 (0.005%)<br>• Cynara cardunculus [taxid 4265]: 2 (0.005%)<br>• other: 130 (0.364%)
Benchmark OTU ID: `CP002026-_Random`<br>OTU taxon: CP002026-_Random [taxid 0]<br>Expected: unknown [taxid 1] (no rank)<br>Number of reads: 77950<br>Number of identified reads: 48800 (62.604%)	• species: 0 (0.0%)<br>• genus: 0 (0.0%)<br>• family: 0 (0.0%)<br>• order: 0 (0.0%)<br>• class: 0 (0.0%)<br>• phylum: 0 (0.0%)<br>• superkingdom: 0 (0.0%)<br>• **root: 46050 (59.076%)**	• Kitasatospora setae [taxid 2066]: 6 (0.007%)<br>• Gymnoxanthella radiolariae [taxid 1798043]: 4 (0.005%)<br>• Pelagococcus subviridis [taxid 35679]: 3 (0.003%)<br>• Methylobacterium indicum [taxid 1775910]: 3 (0.003%)<br>• Tigriopus californicus [taxid 6832]: 2 (0.002%)<br>• Pseudomonas fluorescens [taxid 294]: 2 (0.002%)<br>• Legionella jamestowniensis [taxid 455]: 2 (0.002%)<br>• Scleroderma citrinum [taxid 68788]: 2 (0.002%)<br>• other: 213 (0.273%)
Benchmark OTU ID: `CP002059-_Random`<br>OTU taxon: CP002059-_Random [taxid 0]<br>Expected: unknown [taxid 1] (no rank)<br>Number of reads: 88181<br>Number of identified reads: 79004 (89.592%)	• species: 0 (0.0%)<br>• genus: 0 (0.0%)<br>• family: 0 (0.0%)<br>• order: 0 (0.0%)<br>• class: 0 (0.0%)<br>• phylum: 0 (0.0%)<br>• superkingdom: 0 (0.0%)<br>• **root: 78096 (88.563%)**	• Hucho hucho [taxid 62062]: 6 (0.006%)<br>• Arthrobotrys oligospora [taxid 13349]: 4 (0.004%)<br>• Araneus ventricosus [taxid 182803]: 4 (0.004%)<br>• Buchnera aphidicola [taxid 9]: 4 (0.004%)<br>• Coprinellus micaceus [taxid 71717]: 3 (0.003%)<br>• Triplophysa tibetana [taxid 1572043]: 3 (0.003%)<br>• Branchiostoma belcheri [taxid 7741]: 2 (0.002%)<br>• Mucilaginibacter corticis [taxid 2597670]: 2 (0.002%)<br>• other: 158 (0.179%)
Benchmark OTU ID: `CP002071-_Random`<br>OTU taxon: CP002071-_Random [taxid 0]<br>Expected: unknown [taxid 1] (no rank)<br>Number of reads: 22348<br>Number of identified reads: 20174 (90.272%)	• species: 0 (0.0%)<br>• genus: 0 (0.0%)<br>• family: 0 (0.0%)<br>• order: 0 (0.0%)<br>• class: 0 (0.0%)<br>• phylum: 0 (0.0%)<br>• superkingdom: 0 (0.0%)<br>• **root: 19914 (89.108%)**	• Nannizzia gypsea [taxid 63402]: 3 (0.013%)<br>• Cellulomonas persica [taxid 76861]: 3 (0.013%)<br>• Lachnellula willkommii [taxid 215461]: 2 (0.008%)<br>• Pectobacterium carotovorum [taxid 554]: 2 (0.008%)<br>• Angomonas deanei [taxid 59799]: 2 (0.008%)<br>• Enterococcus rivorum [taxid 762845]: 2 (0.008%)<br>• Peptococcaceae bacterium DCMF [taxid 1761012]: 1 (0.004%)<br>• Stanieria cyanosphaera [taxid 102116]: 1 (0.004%)<br>• other: 46 (0.205%)

Operational Taxonomic Unit (OTU)	Correct identifications	Wrong or overspecific identifications at species rank
Benchmark OTU ID: `CP002076-_Random`<br>OTU taxon: CP002076-_Random [taxid 0]<br>Expected: unknown [taxid 1] (no rank)<br>Number of reads: 23648<br>Number of identified reads: 21293 (90.041%)	• species: 0 (0.0%)<br>• genus: 0 (0.0%)<br>• family: 0 (0.0%)<br>• order: 0 (0.0%)<br>• class: 0 (0.0%)<br>• phylum: 0 (0.0%)<br>• superkingdom: 0 (0.0%)<br>• **root: 21021 (88.891%)**	• Cryptomonas curvata [taxid 233186]: 3 (0.012%)<br>• Corynespora cassiicola [taxid 59586]: 2 (0.008%)<br>• Tetraodon nigroviridis [taxid 99883]: 2 (0.008%)<br>• Marinisporobacter balticus [taxid 2018667]: 2 (0.008%)<br>• Anaeromyces robustus [taxid 1754192]: 2 (0.008%)<br>• Gigaspora rosea [taxid 44941]: 2 (0.008%)<br>• Bacillus endophyticus [taxid 135735]: 1 (0.004%)<br>• Piedraia hortae [taxid 147573]: 1 (0.004%)<br>• other: 54 (0.228%)
Benchmark OTU ID: `CP002207-_Random`<br>OTU taxon: CP002207-_Random [taxid 0]<br>Expected: unknown [taxid 1] (no rank)<br>Number of reads: 67595<br>Number of identified reads: 62656 (92.693%)	• species: 0 (0.0%)<br>• genus: 0 (0.0%)<br>• family: 0 (0.0%)<br>• order: 0 (0.0%)<br>• class: 0 (0.0%)<br>• phylum: 0 (0.0%)<br>• superkingdom: 0 (0.0%)<br>• **root: 61580 (91.101%)**	• Rhizoctonia solani [taxid 456999]: 4 (0.005%)<br>• Crypthecodinium cohnii [taxid 2866]: 3 (0.004%)<br>• Paludifilum halophilum [taxid 1642702]: 2 (0.002%)<br>• Papilio machaon [taxid 76193]: 2 (0.002%)<br>• Saprochaete ingens [taxid 2606893]: 2 (0.002%)<br>• Phytophthora cactorum [taxid 29920]: 2 (0.002%)<br>• Pseudo-nitzschia fraudulenta [taxid 183588]: 2 (0.002%)<br>• Aspergillus oryzae [taxid 5062]: 2 (0.002%)<br>• other: 186 (0.275%)
Benchmark OTU ID: `CP002280-_Random`<br>OTU taxon: CP002280-_Random [taxid 0]<br>Expected: unknown [taxid 1] (no rank)<br>Number of reads: 38756<br>Number of identified reads: 35178 (90.767%)	• species: 0 (0.0%)<br>• genus: 0 (0.0%)<br>• family: 0 (0.0%)<br>• order: 0 (0.0%)<br>• class: 0 (0.0%)<br>• phylum: 0 (0.0%)<br>• superkingdom: 0 (0.0%)<br>• **root: 34267 (88.417%)**	• Vibrio vulnificus [taxid 672]: 4 (0.01%)<br>• Vibrio mangrovi [taxid 474394]: 3 (0.007%)<br>• Terfezia boudieri [taxid 82571]: 2 (0.005%)<br>• Sphingosinicella ginsenosidimutans [taxid 1176539]: 2 (0.005%)<br>• Flavobacterium viscosus [taxid 2488729]: 2 (0.005%)<br>• Jimgerdemannia flammicorona [taxid 994334]: 2 (0.005%)<br>• Oxytricha trifallax [taxid 1172189]: 2 (0.005%)<br>• Malus domestica [taxid 3750]: 2 (0.005%)<br>• other: 128 (0.33%)
Benchmark OTU ID: `CP002304-_Random`<br>OTU taxon: CP002304-_Random [taxid 0]<br>Expected: unknown [taxid 1] (no rank)<br>Number of reads: 40614<br>Number of identified reads: 32820 (80.809%)	• species: 0 (0.0%)<br>• genus: 0 (0.0%)<br>• family: 0 (0.0%)<br>• order: 0 (0.0%)<br>• class: 0 (0.0%)<br>• phylum: 0 (0.0%)<br>• superkingdom: 0 (0.0%)<br>• **root: 32479 (79.969%)**	• Pustulibacterium marinum [taxid 1224947]: 3 (0.007%)<br>• Parasponia andersonii [taxid 3476]: 2 (0.004%)<br>• Halostagnicola kamekurae [taxid 619731]: 2 (0.004%)<br>• Methanofollis liminatans [taxid 2201]: 2 (0.004%)<br>• Nitrososphaeria archaeon [taxid 2268198]: 2 (0.004%)<br>• Plasticicumulans lactativorans [taxid 1133106]: 2 (0.004%)<br>• Devosia geojensis [taxid 443610]: 1 (0.002%)<br>• Loxodonta africana [taxid 9785]: 1 (0.002%)<br>• other: 72 (0.177%)

Operational Taxonomic Unit (OTU)	Correct identifications	Wrong or overspecific identifications at species rank
Benchmark OTU ID: `CP002339-_Random`<br>OTU taxon: CP002339-_Random [taxid 0]<br>Expected: unknown [taxid 1] (no rank)<br>Number of reads: 81543<br>Number of identified reads: 76323 (93.598%)	• species: 0 (0.0%)<br>• genus: 0 (0.0%)<br>• family: 0 (0.0%)<br>• order: 0 (0.0%)<br>• class: 0 (0.0%)<br>• phylum: 0 (0.0%)<br>• superkingdom: 0 (0.0%)<br>• **root: 75071 (92.063%)**	• Guillardia theta [taxid 55529]: 4 (0.004%)<br>• Eumeta japonica [taxid 151549]: 4 (0.004%)<br>• Nocardioides terrae [taxid 574651]: 3 (0.003%)<br>• bacterium [taxid 1869227]: 3 (0.003%)<br>• Acidipila rosea [taxid 768535]: 3 (0.003%)<br>• Aureobasidium pullulans [taxid 5580]: 3 (0.003%)<br>• Candidatus Bathyarchaeota archaeon [taxid 2026714]: 3 (0.003%)<br>• Cavenderia fasciculata [taxid 261658]: 3 (0.003%)<br>• other: 225 (0.275%)
Benchmark OTU ID: `CP002344-_Random`<br>OTU taxon: CP002344-_Random [taxid 0]<br>Expected: unknown [taxid 1] (no rank)<br>Number of reads: 44631<br>Number of identified reads: 29569 (66.252%)	• species: 0 (0.0%)<br>• genus: 0 (0.0%)<br>• family: 0 (0.0%)<br>• order: 0 (0.0%)<br>• class: 0 (0.0%)<br>• phylum: 0 (0.0%)<br>• superkingdom: 0 (0.0%)<br>• **root: 26386 (59.12%)**	• Sorangium cellulosum [taxid 56]: 6 (0.013%)<br>• Mycolicibacterium agri [taxid 36811]: 4 (0.008%)<br>• Prymnesium polylepis [taxid 72548]: 4 (0.008%)<br>• Macrostomum lignano [taxid 282301]: 3 (0.006%)<br>• Mycobacterium asiaticum [taxid 1790]: 3 (0.006%)<br>• Capsicum chinense [taxid 80379]: 3 (0.006%)<br>• Parvibaculum sedimenti [taxid 2608632]: 2 (0.004%)<br>• Nocardia cyriacigeorgica [taxid 135487]: 2 (0.004%)<br>• other: 181 (0.405%)
Benchmark OTU ID: `CP002352-_Random`<br>OTU taxon: CP002352-_Random [taxid 0]<br>Expected: unknown [taxid 1] (no rank)<br>Number of reads: 64658<br>Number of identified reads: 59861 (92.58%)	• species: 0 (0.0%)<br>• genus: 0 (0.0%)<br>• family: 0 (0.0%)<br>• order: 0 (0.0%)<br>• class: 0 (0.0%)<br>• phylum: 0 (0.0%)<br>• superkingdom: 0 (0.0%)<br>• **root: 58774 (90.899%)**	• Karenia brevis [taxid 156230]: 3 (0.004%)<br>• Nitrolancea hollandica [taxid 1206749]: 3 (0.004%)<br>• Aphanomyces stellatus [taxid 120398]: 3 (0.004%)<br>• Helicocarpus griseus [taxid 121128]: 3 (0.004%)<br>• Paroedura picta [taxid 143630]: 2 (0.003%)<br>• Pomacea canaliculata [taxid 400727]: 2 (0.003%)<br>• Micractinium conductrix [taxid 554055]: 2 (0.003%)<br>• Mycolicibacter minnesotensis [taxid 1118379]: 2 (0.003%)<br>• other: 179 (0.276%)
Benchmark OTU ID: `CP002375-_Random`<br>OTU taxon: CP002375-_Random [taxid 0]<br>Expected: unknown [taxid 1] (no rank)<br>Number of reads: 15017<br>Number of identified reads: 13817 (92.009%)	• species: 0 (0.0%)<br>• genus: 0 (0.0%)<br>• family: 0 (0.0%)<br>• order: 0 (0.0%)<br>• class: 0 (0.0%)<br>• phylum: 0 (0.0%)<br>• superkingdom: 0 (0.0%)<br>• **root: 13427 (89.411%)**	• Williamsia sterculiae [taxid 1344003]: 3 (0.019%)<br>• Hyphomicrobium nitrativorans [taxid 1427356]: 3 (0.019%)<br>• Streptomyces glauciniger [taxid 235986]: 2 (0.013%)<br>• Oncopeltus fasciatus [taxid 7536]: 2 (0.013%)<br>• Prymnesium polylepis [taxid 72548]: 2 (0.013%)<br>• Bifidobacterium pullorum [taxid 78448]: 2 (0.013%)<br>• Paenibacillus lutimineralis [taxid 2707005]: 1 (0.006%)<br>• Trichomalopsis sarcophagae [taxid 543379]: 1 (0.006%)<br>• other: 31 (0.206%)

Operational Taxonomic Unit (OTU)	Correct identifications	Wrong or overspecific identifications at species rank
Benchmark OTU ID: `CP002395-_Random`<br>OTU taxon: CP002395-_Random [taxid 0]<br>Expected: unknown [taxid 1] (no rank)<br>Number of reads: 40182<br>Number of identified reads: 33585 (83.582%)	• species: 0 (0.0%)<br>• genus: 0 (0.0%)<br>• family: 0 (0.0%)<br>• order: 0 (0.0%)<br>• class: 0 (0.0%)<br>• phylum: 0 (0.0%)<br>• superkingdom: 0 (0.0%)<br>• **root: 32500 (80.881%)**	• Syncephalastrum racemosum [taxid 13706]: 6 (0.014%)<br>• Shewanella woodyi [taxid 60961]: 3 (0.007%)<br>• Isochrysis galbana [taxid 37099]: 2 (0.004%)<br>• Euryarchaeota archaeon [taxid 2026739]: 2 (0.004%)<br>• Antrodiella citrinella [taxid 2447956]: 2 (0.004%)<br>• Bacteroides fragilis [taxid 817]: 2 (0.004%)<br>• Karenia brevis [taxid 156230]: 2 (0.004%)<br>• Phaeomonas parva [taxid 124430]: 2 (0.004%)<br>• other: 106 (0.263%)
Benchmark OTU ID: `CP002468-_Random`<br>OTU taxon: CP002468-_Random [taxid 0]<br>Expected: unknown [taxid 1] (no rank)<br>Number of reads: 66299<br>Number of identified reads: 61842 (93.277%)	• species: 0 (0.0%)<br>• genus: 0 (0.0%)<br>• family: 0 (0.0%)<br>• order: 0 (0.0%)<br>• class: 0 (0.0%)<br>• phylum: 0 (0.0%)<br>• superkingdom: 0 (0.0%)<br>• **root: 60805 (91.713%)**	• Arachis duranensis [taxid 130453]: 3 (0.004%)<br>• Hirundo rustica [taxid 43150]: 3 (0.004%)<br>• Phallusia mammillata [taxid 59560]: 3 (0.004%)<br>• Robertkochia marina [taxid 1227945]: 2 (0.003%)<br>• Moorella glycerini [taxid 55779]: 2 (0.003%)<br>• Thalassiosira oceanica [taxid 159749]: 2 (0.003%)<br>• Xylaria hypoxylon [taxid 37992]: 2 (0.003%)<br>• Eucalyptus grandis [taxid 71139]: 2 (0.003%)<br>• other: 179 (0.269%)
Benchmark OTU ID: `CP002520-_Random`<br>OTU taxon: CP002520-_Random [taxid 0]<br>Expected: unknown [taxid 1] (no rank)<br>Number of reads: 43342<br>Number of identified reads: 31393 (72.43%)	• species: 0 (0.0%)<br>• genus: 0 (0.0%)<br>• family: 0 (0.0%)<br>• order: 0 (0.0%)<br>• class: 0 (0.0%)<br>• phylum: 0 (0.0%)<br>• superkingdom: 0 (0.0%)<br>• **root: 30170 (69.609%)**	• [Eubacterium] infirmum [taxid 56774]: 3 (0.006%)<br>• Massilia eurypsychrophila [taxid 1485217]: 3 (0.006%)<br>• Prasinoderma coloniale [taxid 156133]: 2 (0.004%)<br>• Candidatus Reconcilbacillus cellulovorans [taxid 1906605]: 2 (0.004%)<br>• Paramoeba atlantica [taxid 1077153]: 2 (0.004%)<br>• Euryarchaeota archaeon [taxid 2026739]: 2 (0.004%)<br>• Thalassiosira gravida [taxid 420259]: 2 (0.004%)<br>• Rhodoferax sediminis [taxid 2509614]: 2 (0.004%)<br>• other: 126 (0.29%)
Benchmark OTU ID: `CP002526-_Random`<br>OTU taxon: CP002526-_Random [taxid 0]<br>Expected: unknown [taxid 1] (no rank)<br>Number of reads: 82934<br>Number of identified reads: 77839 (93.856%)	• species: 0 (0.0%)<br>• genus: 0 (0.0%)<br>• family: 0 (0.0%)<br>• order: 0 (0.0%)<br>• class: 0 (0.0%)<br>• phylum: 0 (0.0%)<br>• superkingdom: 0 (0.0%)<br>• **root: 76520 (92.266%)**	• Drechmeria coniospora [taxid 98403]: 5 (0.006%)<br>• Arthrobacter subterraneus [taxid 335973]: 4 (0.004%)<br>• Gymnodraco acuticeps [taxid 8218]: 4 (0.004%)<br>• Portunus trituberculatus [taxid 210409]: 4 (0.004%)<br>• Candidatus Bathyarchaeota archaeon [taxid 2026714]: 4 (0.004%)<br>• Penicillium italicum [taxid 40296]: 4 (0.004%)<br>• Torrubiella hemipterigena [taxid 1531966]: 3 (0.003%)<br>• Streptomyces kaniharaensis [taxid 212423]: 3 (0.003%)<br>• other: 289 (0.348%)

Operational Taxonomic Unit (OTU)	Correct identifications	Wrong or overspecific identifications at species rank
Benchmark OTU ID: `CP002528-_Random`<br>OTU taxon: CP002528-_Random [taxid 0]<br>Expected: unknown [taxid 1] (no rank)<br>Number of reads: 54092<br>Number of identified reads: 47919 (88.587%)	• species: 0 (0.0%)<br>• genus: 0 (0.0%)<br>• family: 0 (0.0%)<br>• order: 0 (0.0%)<br>• class: 0 (0.0%)<br>• phylum: 0 (0.0%)<br>• superkingdom: 0 (0.0%)<br>• **root: 47355 (87.545%)**	• Tetranychus urticae [taxid 32264]: 3 (0.005%)<br>• Sphaerobolus stellatus [taxid 68786]: 3 (0.005%)<br>• Taibaiella chishuiensis [taxid 1434707]: 2 (0.003%)<br>• Perca fluviatilis [taxid 8168]: 2 (0.003%)<br>• Melipona quadrifasciata [taxid 166423]: 2 (0.003%)<br>• Culicoides sonorensis [taxid 179676]: 2 (0.003%)<br>• Rhizochromulina marina [taxid 1034831]: 2 (0.003%)<br>• Acetanaerobacterium elongatum [taxid 258515]: 2 (0.003%)<br>• other: 135 (0.249%)
Benchmark OTU ID: `CP002530-_Random`<br>OTU taxon: CP002530-_Random [taxid 0]<br>Expected: unknown [taxid 1] (no rank)<br>Number of reads: 68890<br>Number of identified reads: 63912 (92.773%)	• species: 0 (0.0%)<br>• genus: 0 (0.0%)<br>• family: 0 (0.0%)<br>• order: 0 (0.0%)<br>• class: 0 (0.0%)<br>• phylum: 0 (0.0%)<br>• superkingdom: 0 (0.0%)<br>• **root: 62591 (90.856%)**	• Macrostomum lignano [taxid 282301]: 3 (0.004%)<br>• Tropilaelaps mercedesae [taxid 418985]: 2 (0.002%)<br>• Symbiodinium microadriaticum [taxid 2951]: 2 (0.002%)<br>• Toxocara canis [taxid 6265]: 2 (0.002%)<br>• Zymomonas mobilis [taxid 542]: 2 (0.002%)<br>• Heterobasidion irregulare [taxid 984962]: 2 (0.002%)<br>• Glossina austeni [taxid 7395]: 2 (0.002%)<br>• Rodentolepis nana [taxid 102285]: 2 (0.002%)<br>• other: 190 (0.275%)
Benchmark OTU ID: `CP002547-_Random`<br>OTU taxon: CP002547-_Random [taxid 0]<br>Expected: unknown [taxid 1] (no rank)<br>Number of reads: 54383<br>Number of identified reads: 50335 (92.556%)	• species: 0 (0.0%)<br>• genus: 0 (0.0%)<br>• family: 0 (0.0%)<br>• order: 0 (0.0%)<br>• class: 0 (0.0%)<br>• phylum: 0 (0.0%)<br>• superkingdom: 0 (0.0%)<br>• **root: 49399 (90.835%)**	• Pediculus humanus [taxid 121225]: 4 (0.007%)<br>• Brandtodinium nutricula [taxid 1333877]: 4 (0.007%)<br>• Mycolicibacterium peregrinum [taxid 43304]: 3 (0.005%)<br>• Amphiprion ocellaris [taxid 80972]: 3 (0.005%)<br>• Euryarchaeota archaeon [taxid 2026739]: 3 (0.005%)<br>• Desulfotomaculum copahuensis [taxid 1838280]: 2 (0.003%)<br>• Gymnoxanthella radiolariae [taxid 1798043]: 2 (0.003%)<br>• Tetraselmis astigmatica [taxid 1074897]: 2 (0.003%)<br>• other: 149 (0.273%)
Benchmark OTU ID: `CP002568-_Random`<br>OTU taxon: CP002568-_Random [taxid 0]<br>Expected: unknown [taxid 1] (no rank)<br>Number of reads: 75942<br>Number of identified reads: 48467 (63.821%)	• species: 0 (0.0%)<br>• genus: 0 (0.0%)<br>• family: 0 (0.0%)<br>• order: 0 (0.0%)<br>• class: 0 (0.0%)<br>• phylum: 0 (0.0%)<br>• superkingdom: 0 (0.0%)<br>• **root: 45761 (60.257%)**	• Alexandrium monilatum [taxid 311494]: 4 (0.005%)<br>• Volvox carteri [taxid 3067]: 4 (0.005%)<br>• Myxococcus llanfairpwllgwyngyllgogerychwyrndrobwllllantysiliogogogochensis [taxid 2590453]: 3 (0.003%)<br>• Karenia brevis [taxid 156230]: 3 (0.003%)<br>• Nonomuraea pusilla [taxid 46177]: 3 (0.003%)<br>• Pseudopedinella elastica [taxid 35684]: 3 (0.003%)<br>• Rhodopirellula baltica [taxid 265606]: 3 (0.003%)<br>• Channa argus [taxid 215402]: 3 (0.003%)<br>• other: 247 (0.325%)

Operational Taxonomic Unit (OTU)	Correct identifications	Wrong or overspecific identifications at species rank
Benchmark OTU ID: `CP002736-_Random`<br>OTU taxon: CP002736-_Random [taxid 0]<br>Expected: unknown [taxid 1] (no rank)<br>Number of reads: 45457<br>Number of identified reads: 42387 (93.246%)	• species: 0 (0.0%)<br>• genus: 0 (0.0%)<br>• family: 0 (0.0%)<br>• order: 0 (0.0%)<br>• class: 0 (0.0%)<br>• phylum: 0 (0.0%)<br>• superkingdom: 0 (0.0%)<br>• **root: 41561 (91.429%)**	• Lactococcus lactis [taxid 1358]: 4 (0.008%)<br>• Euroglyphus maynei [taxid 6958]: 3 (0.006%)<br>• Portunus trituberculatus [taxid 210409]: 3 (0.006%)<br>• Terfezia boudieri [taxid 82571]: 3 (0.006%)<br>• Rhodosorus marinus [taxid 101924]: 3 (0.006%)<br>• Novosphingobium barchaimii [taxid 1420591]: 3 (0.006%)<br>• Pedobacter duraquae [taxid 425511]: 2 (0.004%)<br>• Rodentibacter pneumotropicus [taxid 758]: 2 (0.004%)<br>• other: 137 (0.301%)
Benchmark OTU ID: `CP002738-_Random`<br>OTU taxon: CP002738-_Random [taxid 0]<br>Expected: unknown [taxid 1] (no rank)<br>Number of reads: 82922<br>Number of identified reads: 76828 (92.65%)	• species: 0 (0.0%)<br>• genus: 0 (0.0%)<br>• family: 0 (0.0%)<br>• order: 0 (0.0%)<br>• class: 0 (0.0%)<br>• phylum: 0 (0.0%)<br>• superkingdom: 0 (0.0%)<br>• **root: 74953 (90.389%)**	• Lingula anatina [taxid 7574]: 5 (0.006%)<br>• Cucurbita moschata [taxid 3662]: 3 (0.003%)<br>• Chlamydia trachomatis [taxid 813]: 3 (0.003%)<br>• Diabrotica virgifera [taxid 50389]: 3 (0.003%)<br>• Auriculariopsis ampla [taxid 97359]: 3 (0.003%)<br>• Starkeya novella [taxid 921]: 3 (0.003%)<br>• Desulfatibacillum aliphaticivorans [taxid 218208]: 3 (0.003%)<br>• Ornithodoros erraticus [taxid 265619]: 3 (0.003%)<br>• other: 264 (0.318%)
Benchmark OTU ID: `CP002771-_Random`<br>OTU taxon: CP002771-_Random [taxid 0]<br>Expected: unknown [taxid 1] (no rank)<br>Number of reads: 62940<br>Number of identified reads: 58972 (93.695%)	• species: 0 (0.0%)<br>• genus: 0 (0.0%)<br>• family: 0 (0.0%)<br>• order: 0 (0.0%)<br>• class: 0 (0.0%)<br>• phylum: 0 (0.0%)<br>• superkingdom: 0 (0.0%)<br>• **root: 57998 (92.148%)**	• Thiorhodococcus mannitoliphagus [taxid 329406]: 5 (0.007%)<br>• Pseudocohnilembus persalinus [taxid 266149]: 5 (0.007%)<br>• Pyramimonas obovata [taxid 1411642]: 3 (0.004%)<br>• Diaphanoeca grandis [taxid 28014]: 3 (0.004%)<br>• Bodo saltans [taxid 75058]: 3 (0.004%)<br>• Eutreptiella gymnastica [taxid 73025]: 2 (0.003%)<br>• Mycolicibacterium tokaiense [taxid 39695]: 2 (0.003%)<br>• Giardia intestinalis [taxid 5741]: 2 (0.003%)<br>• other: 189 (0.3%)
Benchmark OTU ID: `CP002804-_Random`<br>OTU taxon: CP002804-_Random [taxid 0]<br>Expected: unknown [taxid 1] (no rank)<br>Number of reads: 15564<br>Number of identified reads: 14125 (90.754%)	• species: 0 (0.0%)<br>• genus: 0 (0.0%)<br>• family: 0 (0.0%)<br>• order: 0 (0.0%)<br>• class: 0 (0.0%)<br>• phylum: 0 (0.0%)<br>• superkingdom: 0 (0.0%)<br>• **root: 13925 (89.469%)**	• Niabella drilacis [taxid 1285928]: 2 (0.012%)<br>• Pseudocohnilembus persalinus [taxid 266149]: 2 (0.012%)<br>• Lepisosteus oculatus [taxid 7918]: 2 (0.012%)<br>• Alexandrium tamarense [taxid 2926]: 1 (0.006%)<br>• Endocarpon pusillum [taxid 364733]: 1 (0.006%)<br>• Bizionia argentinensis [taxid 456455]: 1 (0.006%)<br>• Durinskia baltica [taxid 400756]: 1 (0.006%)<br>• Leptospira ellisii [taxid 2023197]: 1 (0.006%)<br>• other: 30 (0.192%)

Operational Taxonomic Unit (OTU)	Correct identifications	Wrong or overspecific identifications at species rank
Benchmark OTU ID: `CP002869-_Random`<br>OTU taxon: CP002869-_Random [taxid 0]<br>Expected: unknown [taxid 1] (no rank)<br>Number of reads: 145594<br>Number of identified reads: 120491 (82.758%)	• species: 0 (0.0%)<br>• genus: 0 (0.0%)<br>• family: 0 (0.0%)<br>• order: 0 (0.0%)<br>• class: 0 (0.0%)<br>• phylum: 0 (0.0%)<br>• superkingdom: 0 (0.0%)<br>• **root: 116638 (80.111%)**	• Leptospira bandrabouensis [taxid 2484903]: 4 (0.002%)<br>• Araneus ventricosus [taxid 182803]: 4 (0.002%)<br>• Symbiodinium microadriaticum [taxid 2951]: 3 (0.002%)<br>• Drosophila guanche [taxid 7266]: 3 (0.002%)<br>• Humitalea rosea [taxid 990373]: 3 (0.002%)<br>• Alexandrium monilatum [taxid 311494]: 3 (0.002%)<br>• Actinomadura bangladeshensis [taxid 453573]: 3 (0.002%)<br>• Corethron hystrix [taxid 216773]: 3 (0.002%)<br>• other: 413 (0.283%)
Benchmark OTU ID: `CP002897-_Random`<br>OTU taxon: CP002897-_Random [taxid 0]<br>Expected: unknown [taxid 1] (no rank)<br>Number of reads: 47075<br>Number of identified reads: 31764 (67.475%)	• species: 0 (0.0%)<br>• genus: 0 (0.0%)<br>• family: 0 (0.0%)<br>• order: 0 (0.0%)<br>• class: 0 (0.0%)<br>• phylum: 0 (0.0%)<br>• superkingdom: 0 (0.0%)<br>• **root: 30459 (64.703%)**	• Paroedura picta [taxid 143630]: 3 (0.006%)<br>• Vibrio quintilis [taxid 1117707]: 3 (0.006%)<br>• Achromobacter spanius [taxid 217203]: 3 (0.006%)<br>• Partenskyella glossopodia [taxid 552666]: 3 (0.006%)<br>• Globisporangium ultimum [taxid 2052682]: 2 (0.004%)<br>• Varroa destructor [taxid 109461]: 2 (0.004%)<br>• Durinskia baltica [taxid 400756]: 2 (0.004%)<br>• Herbihabitans rhizosphaerae [taxid 1872711]: 2 (0.004%)<br>• other: 126 (0.267%)
Benchmark OTU ID: `CP002902-_Random`<br>OTU taxon: CP002902-_Random [taxid 0]<br>Expected: unknown [taxid 1] (no rank)<br>Number of reads: 49459<br>Number of identified reads: 37959 (76.748%)	• species: 0 (0.0%)<br>• genus: 0 (0.0%)<br>• family: 0 (0.0%)<br>• order: 0 (0.0%)<br>• class: 0 (0.0%)<br>• phylum: 0 (0.0%)<br>• superkingdom: 0 (0.0%)<br>• **root: 36592 (73.984%)**	• Pandoraea anhela [taxid 2508295]: 3 (0.006%)<br>• Bos mutus [taxid 72004]: 3 (0.006%)<br>• Paenibacillus contaminans [taxid 450362]: 3 (0.006%)<br>• Puccinia triticina [taxid 208348]: 2 (0.004%)<br>• Cryptococcus depauperatus [taxid 5208]: 2 (0.004%)<br>• Neobodo designis [taxid 312471]: 2 (0.004%)<br>• Candidatus Promineofilum breve [taxid 1806508]: 2 (0.004%)<br>• Trichomalopsis sarcophagae [taxid 543379]: 2 (0.004%)<br>• other: 121 (0.244%)
Benchmark OTU ID: `CP002925-_Random`<br>OTU taxon: CP002925-_Random [taxid 0]<br>Expected: unknown [taxid 1] (no rank)<br>Number of reads: 33286<br>Number of identified reads: 30108 (90.452%)	• species: 0 (0.0%)<br>• genus: 0 (0.0%)<br>• family: 0 (0.0%)<br>• order: 0 (0.0%)<br>• class: 0 (0.0%)<br>• phylum: 0 (0.0%)<br>• superkingdom: 0 (0.0%)<br>• **root: 29709 (89.253%)**	• Legionella fallonii [taxid 96230]: 3 (0.009%)<br>• Karenia brevis [taxid 156230]: 2 (0.006%)<br>• Theileria orientalis [taxid 68886]: 2 (0.006%)<br>• Puccinia sorghi [taxid 27349]: 2 (0.006%)<br>• Psilocybe cyanescens [taxid 93625]: 2 (0.006%)<br>• Hebeloma cylindrosporum [taxid 76867]: 2 (0.006%)<br>• Handroanthus impetiginosus [taxid 429701]: 2 (0.006%)<br>• Crustomastix stigmatica [taxid 195967]: 2 (0.006%)<br>• other: 76 (0.228%)

Operational Taxonomic Unit (OTU)	Correct identifications	Wrong or overspecific identifications at species rank
Benchmark OTU ID: `CP003056-_Random`<br>OTU taxon: CP003056-_Random [taxid 0]<br>Expected: unknown [taxid 1] (no rank)<br>Number of reads: 56908<br>Number of identified reads: 53324 (93.702%)	• species: 0 (0.0%)<br>• genus: 0 (0.0%)<br>• family: 0 (0.0%)<br>• order: 0 (0.0%)<br>• class: 0 (0.0%)<br>• phylum: 0 (0.0%)<br>• superkingdom: 0 (0.0%)<br>• **root: 52295 (91.893%)**	• Schizosaccharomyces pombe [taxid 4896]: 4 (0.007%)<br>• Nippostrongylus brasiliensis [taxid 27835]: 4 (0.007%)<br>• Tritrichomonas foetus [taxid 1144522]: 3 (0.005%)<br>• Vitrella brassicaformis [taxid 1169539]: 3 (0.005%)<br>• Sphaerospermopsis torques-reginae [taxid 984207]: 3 (0.005%)<br>• Fasciola hepatica [taxid 6192]: 3 (0.005%)<br>• Chrysochromulina rotalis [taxid 412157]: 2 (0.003%)<br>• Kazachstania africana [taxid 432096]: 2 (0.003%)<br>• other: 180 (0.316%)
Benchmark OTU ID: `SHUFFLED_CP003124-_Random`<br>OTU taxon: SHUFFLED_CP003124-_Random [taxid 0]<br>Expected: unknown [taxid 1] (no rank)<br>Number of reads: 26552<br>Number of identified reads: 23922 (90.094%)	• species: 0 (0.0%)<br>• genus: 0 (0.0%)<br>• family: 0 (0.0%)<br>• order: 0 (0.0%)<br>• class: 0 (0.0%)<br>• phylum: 0 (0.0%)<br>• superkingdom: 0 (0.0%)<br>• **root: 23597 (88.87%)**	• Melghiribacillus thermohalophilus [taxid 1324956]: 3 (0.011%)<br>• Tanacetum cinerariifolium [taxid 118510]: 2 (0.007%)<br>• Diabrotica virgifera [taxid 50389]: 2 (0.007%)<br>• Apolygus lucorum [taxid 248454]: 2 (0.007%)<br>• Rozella allomycis [taxid 281847]: 2 (0.007%)<br>• Proteus vulgaris [taxid 585]: 2 (0.007%)<br>• Idiomarina baltica [taxid 190892]: 2 (0.007%)<br>• Algoriphagus ratkowskyi [taxid 57028]: 1 (0.003%)<br>• other: 61 (0.229%)
Benchmark OTU ID: `CP003132-_Random`<br>OTU taxon: CP003132-_Random [taxid 0]<br>Expected: unknown [taxid 1] (no rank)<br>Number of reads: 37830<br>Number of identified reads: 32623 (86.235%)	• species: 0 (0.0%)<br>• genus: 0 (0.0%)<br>• family: 0 (0.0%)<br>• order: 0 (0.0%)<br>• class: 0 (0.0%)<br>• phylum: 0 (0.0%)<br>• superkingdom: 0 (0.0%)<br>• **root: 32270 (85.302%)**	• Halanaerobium saccharolyticum [taxid 43595]: 3 (0.007%)<br>• Marinomonas spartinae [taxid 1792290]: 3 (0.007%)<br>• Mesoplasma tabanidae [taxid 219745]: 2 (0.005%)<br>• Agrobacterium rubi [taxid 28099]: 2 (0.005%)<br>• Lentinula edodes [taxid 5353]: 2 (0.005%)<br>• Gossypium mustelinum [taxid 34275]: 2 (0.005%)<br>• Brevibacterium aurantiacum [taxid 273384]: 2 (0.005%)<br>• Caenorhabditis japonica [taxid 281687]: 2 (0.005%)<br>• other: 70 (0.185%)
Benchmark OTU ID: `CP003257-_Random`<br>OTU taxon: CP003257-_Random [taxid 0]<br>Expected: unknown [taxid 1] (no rank)<br>Number of reads: 33992<br>Number of identified reads: 24145 (71.031%)	• species: 0 (0.0%)<br>• genus: 0 (0.0%)<br>• family: 0 (0.0%)<br>• order: 0 (0.0%)<br>• class: 0 (0.0%)<br>• phylum: 0 (0.0%)<br>• superkingdom: 0 (0.0%)<br>• **root: 23938 (70.422%)**	• Oryzias latipes [taxid 8090]: 4 (0.011%)<br>• Oscillochloris trichoides [taxid 104176]: 2 (0.005%)<br>• Photobacterium halotolerans [taxid 265726]: 2 (0.005%)<br>• Juglans regia [taxid 51240]: 1 (0.002%)<br>• Methanoregulaceae archaeon [taxid 2485498]: 1 (0.002%)<br>• Mucilaginibacter gracilis [taxid 423350]: 1 (0.002%)<br>• Lingula anatina [taxid 7574]: 1 (0.002%)<br>• Plasmodium chabaudi [taxid 5825]: 1 (0.002%)<br>• other: 38 (0.111%)

Operational Taxonomic Unit (OTU)	Correct identifications	Wrong or overspecific identifications at species rank
Benchmark OTU ID: `CP003295-_Random`<br>OTU taxon: CP003295-_Random [taxid 0]<br>Expected: unknown [taxid 1] (no rank)<br>Number of reads: 29776<br>Number of identified reads: 26488 (88.957%)	• species: 0 (0.0%)<br>• genus: 0 (0.0%)<br>• family: 0 (0.0%)<br>• order: 0 (0.0%)<br>• class: 0 (0.0%)<br>• phylum: 0 (0.0%)<br>• superkingdom: 0 (0.0%)<br>• **root: 26199 (87.986%)**	• Corethron pennatum [taxid 218684]: 2 (0.006%)<br>• Gaeumannomyces tritici [taxid 36779]: 2 (0.006%)<br>• Phialocephala scopiformis [taxid 149040]: 2 (0.006%)<br>• Roseivirga pacifica [taxid 1267423]: 2 (0.006%)<br>• Streptomyces gilvosporeus [taxid 553510]: 2 (0.006%)<br>• Euryarchaeota archaeon [taxid 2026739]: 2 (0.006%)<br>• Alteromonas mediterranea [taxid 314275]: 2 (0.006%)<br>• Andreprevotia lacus [taxid 1121000]: 1 (0.003%)<br>• other: 56 (0.188%)
Benchmark OTU ID:<br>`SHUFFLED_CP003394-_Random`<br>OTU taxon:<br>SHUFFLED_CP003394-_Random [taxid 0]<br>Expected: unknown [taxid 1] (no rank)<br>Number of reads: 14495<br>Number of identified reads: 10286 (70.962%)	• species: 0 (0.0%)<br>• genus: 0 (0.0%)<br>• family: 0 (0.0%)<br>• order: 0 (0.0%)<br>• class: 0 (0.0%)<br>• phylum: 0 (0.0%)<br>• superkingdom: 0 (0.0%)<br>• **root: 10213 (70.458%)**	• Bradyrhizobium betae [taxid 244734]: 4 (0.027%)<br>• Hordeum vulgare [taxid 4513]: 1 (0.006%)<br>• Schistosoma japonicum [taxid 6182]: 1 (0.006%)<br>• Chroogloeocystis siderophila [taxid 329163]: 1 (0.006%)<br>• Mesorhizobium plurifarium [taxid 69974]: 1 (0.006%)<br>• Methanosarcinales archaeon [taxid 2250255]: 1 (0.006%)<br>• Chryseobacterium jeonii [taxid 266749]: 1 (0.006%)<br>• Martelella mediterranea [taxid 293089]: 1 (0.006%)<br>• other: 9 (0.062%)
Benchmark OTU ID:<br>`SHUFFLED_CP003557-_Random`<br>OTU taxon:<br>SHUFFLED_CP003557-_Random [taxid 0]<br>Expected: unknown [taxid 1] (no rank)<br>Number of reads: 52539<br>Number of identified reads: 48312 (91.954%)	• species: 0 (0.0%)<br>• genus: 0 (0.0%)<br>• family: 0 (0.0%)<br>• order: 0 (0.0%)<br>• class: 0 (0.0%)<br>• phylum: 0 (0.0%)<br>• superkingdom: 0 (0.0%)<br>• **root: 47570 (90.542%)**	• Erinaceus europaeus [taxid 9365]: 2 (0.003%)<br>• Heterorhabditis bacteriophora [taxid 37862]: 2 (0.003%)<br>• Paramuricea clavata [taxid 317549]: 2 (0.003%)<br>• Amphimedon queenslandica [taxid 400682]: 2 (0.003%)<br>• archaeon [taxid 1906665]: 2 (0.003%)<br>• Bacteroides cellulosilyticus [taxid 246787]: 2 (0.003%)<br>• Pseudocercospora eumusae [taxid 321146]: 2 (0.003%)<br>• Fomitopsis rosea [taxid 34475]: 2 (0.003%)<br>• other: 123 (0.234%)
Benchmark OTU ID:<br>`SHUFFLED_CP003590-_Random`<br>OTU taxon:<br>SHUFFLED_CP003590-_Random [taxid 0]<br>Expected: unknown [taxid 1] (no rank)<br>Number of reads: 81798<br>Number of identified reads: 76472 (93.488%)	• species: 0 (0.0%)<br>• genus: 0 (0.0%)<br>• family: 0 (0.0%)<br>• order: 0 (0.0%)<br>• class: 0 (0.0%)<br>• phylum: 0 (0.0%)<br>• superkingdom: 0 (0.0%)<br>• **root: 75122 (91.838%)**	• Bergeyella zoohelcum [taxid 1015]: 3 (0.003%)<br>• Hanseniaspora guilliermondii [taxid 56406]: 3 (0.003%)<br>• Kingella oralis [taxid 505]: 3 (0.003%)<br>• Vitis vinifera [taxid 29760]: 3 (0.003%)<br>• Hortaea thailandica [taxid 706561]: 2 (0.002%)<br>• Meleagris gallopavo [taxid 9103]: 2 (0.002%)<br>• Oxyrrhis marina [taxid 2969]: 2 (0.002%)<br>• Raphanus sativus [taxid 3726]: 2 (0.002%)<br>• other: 234 (0.286%)

Operational Taxonomic Unit (OTU)	Correct identifications	Wrong or overspecific identifications at species rank
Benchmark OTU ID: `SHUFFLED_CP003678-_Random`<br>OTU taxon: SHUFFLED_CP003678-_Random [taxid 0]<br>Expected: unknown [taxid 1] (no rank)<br>Number of reads: 81475<br>Number of identified reads: 72677 (89.201%)	• species: 0 (0.0%)<br>• genus: 0 (0.0%)<br>• family: 0 (0.0%)<br>• order: 0 (0.0%)<br>• class: 0 (0.0%)<br>• phylum: 0 (0.0%)<br>• superkingdom: 0 (0.0%)<br>• **root: 70721 (86.8%)**	• Rhizobium oryzae [taxid 464029]: 5 (0.006%)<br>• Pseudovirgaria hyperparasitica [taxid 470096]: 4 (0.004%)<br>• Tetraodon nigroviridis [taxid 99883]: 3 (0.003%)<br>• Pelagodinium beii [taxid 43686]: 3 (0.003%)<br>• Cuerna arida [taxid 1464854]: 3 (0.003%)<br>• Euryarchaeota archaeon [taxid 2026739]: 3 (0.003%)<br>• Oceanospirillum multiglobuliferum [taxid 64969]: 3 (0.003%)<br>• Tanacetum cinerariifolium [taxid 118510]: 3 (0.003%)<br>• other: 240 (0.294%)
Benchmark OTU ID: `SHUFFLED_CP003699-_Random`<br>OTU taxon: SHUFFLED_CP003699-_Random [taxid 0]<br>Expected: unknown [taxid 1] (no rank)<br>Number of reads: 83221<br>Number of identified reads: 59043 (70.947%)	• species: 0 (0.0%)<br>• genus: 0 (0.0%)<br>• family: 0 (0.0%)<br>• order: 0 (0.0%)<br>• class: 0 (0.0%)<br>• phylum: 0 (0.0%)<br>• superkingdom: 0 (0.0%)<br>• **root: 56719 (68.154%)**	• Allomyces macrogynus [taxid 28583]: 4 (0.004%)<br>• Clavispora lusitaniae [taxid 36911]: 3 (0.003%)<br>• Methylocystis heyeri [taxid 391905]: 3 (0.003%)<br>• Ferruginivarius sediminum [taxid 2661937]: 3 (0.003%)<br>• Paludisphaera borealis [taxid 1387353]: 3 (0.003%)<br>• Alexandrium monilatum [taxid 311494]: 3 (0.003%)<br>• Diaminobutyricibacter tongyongensis [taxid 1268043]: 2 (0.002%)<br>• Pseudocohnilembus persalinus [taxid 266149]: 2 (0.002%)<br>• other: 208 (0.249%)
Benchmark OTU ID: `SHUFFLED_CP003790-_Random`<br>OTU taxon: SHUFFLED_CP003790-_Random [taxid 0]<br>Expected: unknown [taxid 1] (no rank)<br>Number of reads: 15611<br>Number of identified reads: 14147 (90.621%)	• species: 0 (0.0%)<br>• genus: 0 (0.0%)<br>• family: 0 (0.0%)<br>• order: 0 (0.0%)<br>• class: 0 (0.0%)<br>• phylum: 0 (0.0%)<br>• superkingdom: 0 (0.0%)<br>• **root: 14006 (89.718%)**	• Pseudidiomarina planktonica [taxid 1323738]: 2 (0.012%)<br>• Azoarcus indigens [taxid 29545]: 2 (0.012%)<br>• [Empedobacter] haloabium [taxid 592317]: 2 (0.012%)<br>• Winkia neuii [taxid 33007]: 2 (0.012%)<br>• Friedmanniomyces endolithicus [taxid 329885]: 2 (0.012%)<br>• Flavobacterium sufflavum [taxid 1921138]: 2 (0.012%)<br>• Deinococcus detaillensis [taxid 2592048]: 1 (0.006%)<br>• Rubritalea profundi [taxid 1658618]: 1 (0.006%)<br>• other: 29 (0.185%)
Benchmark OTU ID: `SHUFFLED_CP003801-_Random`<br>OTU taxon: SHUFFLED_CP003801-_Random [taxid 0]<br>Expected: unknown [taxid 1] (no rank)<br>Number of reads: 19742<br>Number of identified reads: 15464 (78.33%)	• species: 0 (0.0%)<br>• genus: 0 (0.0%)<br>• family: 0 (0.0%)<br>• order: 0 (0.0%)<br>• class: 0 (0.0%)<br>• phylum: 0 (0.0%)<br>• superkingdom: 0 (0.0%)<br>• **root: 15309 (77.545%)**	• Helicobacter felis [taxid 214]: 2 (0.01%)<br>• Gymnoxanthella radiolariae [taxid 1798043]: 2 (0.01%)<br>• Aeromicrobium terrae [taxid 2498846]: 2 (0.01%)<br>• Micavibrio aeruginosavorus [taxid 349221]: 1 (0.005%)<br>• Meira miltonrushii [taxid 1280837]: 1 (0.005%)<br>• Helicobacter pylori [taxid 210]: 1 (0.005%)<br>• Plasmodium cynomolgi [taxid 5827]: 1 (0.005%)<br>• Metarhizium acridum [taxid 92637]: 1 (0.005%)<br>• other: 25 (0.126%)

Operational Taxonomic Unit (OTU)	Correct identifications	Wrong or overspecific identifications at species rank
Benchmark OTU ID:<br>`SHUFFLED_CP003902-_Random`<br>OTU taxon:<br>SHUFFLED_CP003902-_Random [taxid 0]<br>Expected: unknown [taxid 1] (no rank)<br>Number of reads: 15029<br>Number of identified reads: 13825 (91.988%)	• species: 0 (0.0%)<br>• genus: 0 (0.0%)<br>• family: 0 (0.0%)<br>• order: 0 (0.0%)<br>• class: 0 (0.0%)<br>• phylum: 0 (0.0%)<br>• superkingdom: 0 (0.0%)<br>• **root: 13486 (89.733%)**	• Palleronia marisminoris [taxid 315423]: 4 (0.026%)<br>• Colwellia chukchiensis [taxid 641665]: 2 (0.013%)<br>• Fragilariopsis cylindrus [taxid 186039]: 2 (0.013%)<br>• Cryptosporangium phraense [taxid 2593070]: 2 (0.013%)<br>• Candidatus Bathyarchaeota archaeon [taxid 2026714]: 1 (0.006%)<br>• Pocillopora damicornis [taxid 46731]: 1 (0.006%)<br>• Gymnoxanthella radiolariae [taxid 1798043]: 1 (0.006%)<br>• Angiostrongylus cantonensis [taxid 6313]: 1 (0.006%)<br>• other: 34 (0.226%)
Benchmark OTU ID:<br>`SHUFFLED_CP003943-_Random`<br>OTU taxon:<br>SHUFFLED_CP003943-_Random [taxid 0]<br>Expected: unknown [taxid 1] (no rank)<br>Number of reads: 117130<br>Number of identified reads: 108261 (92.428%)	• species: 0 (0.0%)<br>• genus: 0 (0.0%)<br>• family: 0 (0.0%)<br>• order: 0 (0.0%)<br>• class: 0 (0.0%)<br>• phylum: 0 (0.0%)<br>• superkingdom: 0 (0.0%)<br>• **root: 106505 (90.928%)**	• Streptomyces yokosukanensis [taxid 67386]: 4 (0.003%)<br>• Paenibacillus thalictri [taxid 2527873]: 4 (0.003%)<br>• Araneus ventricosus [taxid 182803]: 4 (0.003%)<br>• Prymnesium polylepis [taxid 72548]: 3 (0.002%)<br>• Propithecus coquereli [taxid 379532]: 3 (0.002%)<br>• Clonorchis sinensis [taxid 79923]: 3 (0.002%)<br>• Brachypodium distachyon [taxid 15368]: 2 (0.001%)<br>• Dinoponera quadriceps [taxid 609295]: 2 (0.001%)<br>• other: 314 (0.268%)
Benchmark OTU ID:<br>`SHUFFLED_CP003944-_Random`<br>OTU taxon:<br>SHUFFLED_CP003944-_Random [taxid 0]<br>Expected: unknown [taxid 1] (no rank)<br>Number of reads: 60877<br>Number of identified reads: 56402 (92.649%)	• species: 0 (0.0%)<br>• genus: 0 (0.0%)<br>• family: 0 (0.0%)<br>• order: 0 (0.0%)<br>• class: 0 (0.0%)<br>• phylum: 0 (0.0%)<br>• superkingdom: 0 (0.0%)<br>• **root: 55552 (91.252%)**	• Saliterribacillus persicus [taxid 930114]: 3 (0.004%)<br>• Weissella muntiaci [taxid 2508881]: 3 (0.004%)<br>• Chromera velia [taxid 505693]: 2 (0.003%)<br>• Sphaerulina musiva [taxid 85929]: 2 (0.003%)<br>• Gigaspora rosea [taxid 44941]: 2 (0.003%)<br>• Puccinia coronata [taxid 27344]: 2 (0.003%)<br>• Fragilariopsis kerguelensis [taxid 186038]: 2 (0.003%)<br>• Rhizobium oryzae [taxid 464029]: 2 (0.003%)<br>• other: 163 (0.267%)
Benchmark OTU ID:<br>`SHUFFLED_CP004753-_Random`<br>OTU taxon:<br>SHUFFLED_CP004753-_Random [taxid 0]<br>Expected: unknown [taxid 1] (no rank)<br>Number of reads: 39426<br>Number of identified reads: 36007 (91.328%)	• species: 0 (0.0%)<br>• genus: 0 (0.0%)<br>• family: 0 (0.0%)<br>• order: 0 (0.0%)<br>• class: 0 (0.0%)<br>• phylum: 0 (0.0%)<br>• superkingdom: 0 (0.0%)<br>• **root: 35484 (90.001%)**	• Lacticaseibacillus rhamnosus [taxid 47715]: 3 (0.007%)<br>• Saitozyma podzolica [taxid 1890683]: 3 (0.007%)<br>• Karenia brevis [taxid 156230]: 3 (0.007%)<br>• Alteriqipengyuania halimionae [taxid 1926630]: 3 (0.007%)<br>• Tanacetum cinerariifolium [taxid 118510]: 2 (0.005%)<br>• Didymella exigua [taxid 100019]: 2 (0.005%)<br>• Orchesella cincta [taxid 48709]: 2 (0.005%)<br>• Chryseobacterium carnipullorum [taxid 1124835]: 2 (0.005%)<br>• other: 105 (0.266%)

Operational Taxonomic Unit (OTU)	Correct identifications	Wrong or overspecific identifications at species rank
Benchmark OTU ID: `SHUFFLED_CP005094-_Random`<br>OTU taxon: SHUFFLED_CP005094-_Random [taxid 0]<br>Expected: unknown [taxid 1] (no rank)<br>Number of reads: 88382<br>Number of identified reads: 59861 (67.729%)	• species: 0 (0.0%)<br>• genus: 0 (0.0%)<br>• family: 0 (0.0%)<br>• order: 0 (0.0%)<br>• class: 0 (0.0%)<br>• phylum: 0 (0.0%)<br>• superkingdom: 0 (0.0%)<br>• **root: 56893 (64.371%)**	• bacterium [taxid 1869227]: 4 (0.004%)<br>• Macrostomum lignano [taxid 282301]: 3 (0.003%)<br>• Agaricus bisporus [taxid 5341]: 3 (0.003%)<br>• Diaminobutyricimonas aerilata [taxid 1162967]: 3 (0.003%)<br>• Apocalathium aciculiferum [taxid 268820]: 3 (0.003%)<br>• Brandtodinium nutricula [taxid 1333877]: 3 (0.003%)<br>• Tetraselmis astigmatica [taxid 1074897]: 2 (0.002%)<br>• Photobacterium phosphoreum [taxid 659]: 2 (0.002%)<br>• other: 261 (0.295%)
Benchmark OTU ID: `CR954253-_Random`<br>OTU taxon: CR954253-_Random [taxid 0]<br>Expected: unknown [taxid 1] (no rank)<br>Number of reads: 27633<br>Number of identified reads: 25764 (93.236%)	• species: 0 (0.0%)<br>• genus: 0 (0.0%)<br>• family: 0 (0.0%)<br>• order: 0 (0.0%)<br>• class: 0 (0.0%)<br>• phylum: 0 (0.0%)<br>• superkingdom: 0 (0.0%)<br>• **root: 25163 (91.061%)**	• Gulo gulo [taxid 48420]: 2 (0.007%)<br>• Acrocarpospora corrugata [taxid 35763]: 2 (0.007%)<br>• Ophiocordyceps camponoti-leonardi (nom. inval.) [taxid 2039875]: 2 (0.007%)<br>• Phlebiopsis gigantea [taxid 82310]: 2 (0.007%)<br>• Candidatus Moduliflexus flocculans [taxid 1499966]: 2 (0.007%)<br>• Lichtheimia ramosa [taxid 688394]: 2 (0.007%)<br>• Aureoumbra lagunensis [taxid 44058]: 2 (0.007%)<br>• Domibacillus epiphyticus [taxid 1714355]: 2 (0.007%)<br>• other: 77 (0.278%)
Benchmark OTU ID: `ENA|CT009589|CT009589.1-_Random`<br>OTU taxon: ENA|CT009589|CT009589.1-_Random [taxid 0]<br>Expected: unknown [taxid 1] (no rank)<br>Number of reads: 52695<br>Number of identified reads: 48146 (91.367%)	• species: 0 (0.0%)<br>• genus: 0 (0.0%)<br>• family: 0 (0.0%)<br>• order: 0 (0.0%)<br>• class: 0 (0.0%)<br>• phylum: 0 (0.0%)<br>• superkingdom: 0 (0.0%)<br>• **root: 46900 (89.002%)**	• Clostridium carnis [taxid 1530]: 4 (0.007%)<br>• Halolactibacillus miurensis [taxid 306541]: 4 (0.007%)<br>• Asparagus officinalis [taxid 4686]: 3 (0.005%)<br>• Tanacetum cinerariifolium [taxid 118510]: 3 (0.005%)<br>• Eutreptiella gymnastica [taxid 73025]: 3 (0.005%)<br>• Alkalispirochaeta americana [taxid 159291]: 2 (0.003%)<br>• Karlodinium veneficum [taxid 407301]: 2 (0.003%)<br>• Folsomia candida [taxid 158441]: 2 (0.003%)<br>• other: 167 (0.316%)
Benchmark OTU ID: `CU234118-_Random`<br>OTU taxon: CU234118-_Random [taxid 0]<br>Expected: unknown [taxid 1] (no rank)<br>Number of reads: 124647<br>Number of identified reads: 83323 (66.847%)	• species: 0 (0.0%)<br>• genus: 0 (0.0%)<br>• family: 0 (0.0%)<br>• order: 0 (0.0%)<br>• class: 0 (0.0%)<br>• phylum: 0 (0.0%)<br>• superkingdom: 0 (0.0%)<br>• **root: 79509 (63.787%)**	• Pelagodinium beii [taxid 43686]: 5 (0.004%)<br>• Nakamurella panacisegetis [taxid 1090615]: 5 (0.004%)<br>• Friedmanniomyces simplex [taxid 329884]: 4 (0.003%)<br>• Parasitella parasitica [taxid 35722]: 4 (0.003%)<br>• Oceanotoga teriensis [taxid 515440]: 3 (0.002%)<br>• Candidatus Pacearchaeota archaeon [taxid 2026773]: 3 (0.002%)<br>• Kouleothrix aurantiaca [taxid 186479]: 3 (0.002%)<br>• Methylobacterium currus [taxid 2051553]: 3 (0.002%)<br>• other: 399 (0.32%)

Operational Taxonomic Unit (OTU)	Correct identifications	Wrong or overspecific identifications at species rank
Benchmark OTU ID: `FM211192-_Random`<br>OTU taxon: FM211192-_Random [taxid 0]<br>Expected: unknown [taxid 1] (no rank)<br>Number of reads: 51967<br>Number of identified reads: 45025 (86.641%)	• species: 0 (0.0%)<br>• genus: 0 (0.0%)<br>• family: 0 (0.0%)<br>• order: 0 (0.0%)<br>• class: 0 (0.0%)<br>• phylum: 0 (0.0%)<br>• superkingdom: 0 (0.0%)<br>• **root: 43540 (83.783%)**	• Coptotermes formosanus [taxid 36987]: 3 (0.005%)<br>• Gonium pectorale [taxid 33097]: 3 (0.005%)<br>• Drosophila suzukii [taxid 28584]: 3 (0.005%)<br>• Tanacetum cinerariifolium [taxid 118510]: 2 (0.003%)<br>• Gracilariopsis chorda [taxid 448386]: 2 (0.003%)<br>• Paraphaeosphaeria sporulosa [taxid 1460663]: 2 (0.003%)<br>• Scyphosphaera apsteinii [taxid 418940]: 2 (0.003%)<br>• Chlamydomonas reinhardtii [taxid 3055]: 2 (0.003%)<br>• other: 160 (0.307%)
Benchmark OTU ID: `FN665652-_Random`<br>OTU taxon: FN665652-_Random [taxid 0]<br>Expected: unknown [taxid 1] (no rank)<br>Number of reads: 67444<br>Number of identified reads: 46066 (68.302%)	• species: 0 (0.0%)<br>• genus: 0 (0.0%)<br>• family: 0 (0.0%)<br>• order: 0 (0.0%)<br>• class: 0 (0.0%)<br>• phylum: 0 (0.0%)<br>• superkingdom: 0 (0.0%)<br>• **root: 45653 (67.69%)**	• Candidatus Bathyarchaeota archaeon [taxid 2026714]: 6 (0.008%)<br>• Penicillium roqueforti [taxid 5082]: 2 (0.002%)<br>• Golovinomyces magnicellulatus [taxid 62714]: 2 (0.002%)<br>• Cytobacillus oceanisediminis [taxid 665099]: 2 (0.002%)<br>• Gallibacterium salpingitidis [taxid 505341]: 2 (0.002%)<br>• Pseudomonas phage OBP [taxid 1124849]: 2 (0.002%)<br>• Caloramator mitchellensis [taxid 908809]: 2 (0.002%)<br>• Microcoleus vaginatus [taxid 119532]: 1 (0.001%)<br>• other: 88 (0.13%)
Benchmark OTU ID: `FP929042-_Random`<br>OTU taxon: FP929042-_Random [taxid 0]<br>Expected: unknown [taxid 1] (no rank)<br>Number of reads: 50377<br>Number of identified reads: 45930 (91.172%)	• species: 0 (0.0%)<br>• genus: 0 (0.0%)<br>• family: 0 (0.0%)<br>• order: 0 (0.0%)<br>• class: 0 (0.0%)<br>• phylum: 0 (0.0%)<br>• superkingdom: 0 (0.0%)<br>• **root: 45244 (89.81%)**	• Chaetoceros debilis [taxid 122233]: 3 (0.005%)<br>• Flavobacterium gillisiae [taxid 150146]: 3 (0.005%)<br>• Caulochytrium protostelioides [taxid 1555241]: 3 (0.005%)<br>• Corynebacterium casei [taxid 160386]: 2 (0.003%)<br>• Rhizophagus irregularis [taxid 588596]: 2 (0.003%)<br>• Synedropsis cf. recta [taxid 265551]: 2 (0.003%)<br>• Aeromonas caviae [taxid 648]: 2 (0.003%)<br>• Mytilus coruscus [taxid 42192]: 2 (0.003%)<br>• other: 129 (0.256%)
Benchmark OTU ID: `FQ312041-_Random`<br>OTU taxon: FQ312041-_Random [taxid 0]<br>Expected: unknown [taxid 1] (no rank)<br>Number of reads: 30447<br>Number of identified reads: 27488 (90.281%)	• species: 0 (0.0%)<br>• genus: 0 (0.0%)<br>• family: 0 (0.0%)<br>• order: 0 (0.0%)<br>• class: 0 (0.0%)<br>• phylum: 0 (0.0%)<br>• superkingdom: 0 (0.0%)<br>• **root: 27131 (89.108%)**	• Methylobacter tundripaludum [taxid 173365]: 4 (0.013%)<br>• Pedosphaera parvula [taxid 1032527]: 2 (0.006%)<br>• Salix viminalis [taxid 40686]: 2 (0.006%)<br>• Sedimenticola selenatireducens [taxid 191960]: 2 (0.006%)<br>• Podarcis muralis [taxid 64176]: 2 (0.006%)<br>• Rhodnius prolixus [taxid 13249]: 2 (0.006%)<br>• Pichia kudriavzevii [taxid 4909]: 2 (0.006%)<br>• Aureococcus anophagefferens [taxid 44056]: 2 (0.006%)<br>• other: 63 (0.206%)

Operational Taxonomic Unit (OTU)	Correct identifications	Wrong or overspecific identifications at species rank
Benchmark OTU ID: `SHUFFLED_HF545614-_Random`<br>OTU taxon: SHUFFLED_HF545614-_Random [taxid 0]<br>Expected: unknown [taxid 1] (no rank)<br>Number of reads: 15524<br>Number of identified reads: 14135 (91.052%)	• species: 0 (0.0%)<br>• genus: 0 (0.0%)<br>• family: 0 (0.0%)<br>• order: 0 (0.0%)<br>• class: 0 (0.0%)<br>• phylum: 0 (0.0%)<br>• superkingdom: 0 (0.0%)<br>• **root: 13986 (90.092%)**	• Pseudidiomarina homiensis [taxid 364198]: 2 (0.012%)<br>• Thrips palmi [taxid 161013]: 2 (0.012%)<br>• Scrippsiella trochoidea [taxid 71861]: 2 (0.012%)<br>• Pelagerythrobacter marensis [taxid 543877]: 1 (0.006%)<br>• Hymenobacter mucosus [taxid 1411120]: 1 (0.006%)<br>• Mycobacterium parmense [taxid 185642]: 1 (0.006%)<br>• Mammaliicoccus stepanovicii [taxid 643214]: 1 (0.006%)<br>• Methylacidiphilum kamchatkense [taxid 431057]: 1 (0.006%)<br>• other: 25 (0.161%)
Benchmark OTU ID: `SHUFFLED_HF680312-_Random`<br>OTU taxon: SHUFFLED_HF680312-_Random [taxid 0]<br>Expected: unknown [taxid 1] (no rank)<br>Number of reads: 63294<br>Number of identified reads: 59637 (94.222%)	• species: 0 (0.0%)<br>• genus: 0 (0.0%)<br>• family: 0 (0.0%)<br>• order: 0 (0.0%)<br>• class: 0 (0.0%)<br>• phylum: 0 (0.0%)<br>• superkingdom: 0 (0.0%)<br>• **root: 58469 (92.376%)**	• Galerina marginata [taxid 109633]: 8 (0.012%)<br>• Peniophora sp. CONT [taxid 1314672]: 3 (0.004%)<br>• Azadirachta indica [taxid 124943]: 3 (0.004%)<br>• Camellia sinensis [taxid 4442]: 3 (0.004%)<br>• Natronospirillum operosum [taxid 2759953]: 3 (0.004%)<br>• Nanoarchaeota archaeon [taxid 2026764]: 3 (0.004%)<br>• Botrimarina hoheduenensis [taxid 2528000]: 3 (0.004%)<br>• Labedaea rhizosphaerae [taxid 598644]: 3 (0.004%)<br>• other: 195 (0.308%)

Benchmark dataset, simulated divergence reads

Operational Taxonomic Unit (OTU)	Correct identifications	Wrong or overspecific identifications at species rank
Benchmark OTU ID: `Rose_little_divergence_sequence_i-_Simulated`<br>OTU taxon: Leptospira interrogans serovar Copenhageni str. Fiocruz L1-130 [taxid 267671]<br>Expected: Leptospira interrogans [taxid 173] (species)<br>Number of reads: 45483<br>Number of identified reads: 43860 (96.431%)	• species: 16658 (36.624%)<br>• **genus: 18295 (40.223%)**<br>• family: 5 (0.01%)<br>• order: 0 (0.0%)<br>• class: 10 (0.021%)<br>• phylum: 12 (0.026%)<br>• superkingdom: 1641 (3.607%)<br>• root: 7212 (15.856%)	• Leptospira kirschneri [taxid 29507]: 135 (0.296%)<br>• Leptospira noguchii [taxid 28182]: 83 (0.182%)<br>• Leptospira weilii [taxid 28184]: 45 (0.098%)<br>• Leptospira borgpetersenii [taxid 174]: 30 (0.065%)<br>• Leptospira santarosai [taxid 28183]: 23 (0.05%)<br>• Leptospira alstonii [taxid 28452]: 22 (0.048%)<br>• Leptospira gomenensis [taxid 2484974]: 14 (0.03%)<br>• Leptospira tipperaryensis [taxid 2564040]: 13 (0.028%)<br>• other: 165 (0.362%)
Benchmark OTU ID: `Rose_little_divergence_sequence_g-_Simulated`<br>OTU taxon: Leptospira interrogans serovar Copenhageni str. Fiocruz L1-130 [taxid 267671]<br>Expected: Leptospira interrogans [taxid 173] (species)<br>Number of reads: 45492<br>Number of identified reads: 43849 (96.388%)	• species: 16548 (36.375%)<br>• **genus: 18327 (40.286%)**<br>• family: 9 (0.019%)<br>• order: 0 (0.0%)<br>• class: 13 (0.028%)<br>• phylum: 8 (0.017%)<br>• superkingdom: 1673 (3.677%)<br>• root: 7250 (15.936%)	• Leptospira kirschneri [taxid 29507]: 150 (0.329%)<br>• Leptospira noguchii [taxid 28182]: 91 (0.2%)<br>• Leptospira weilii [taxid 28184]: 37 (0.081%)<br>• Leptospira borgpetersenii [taxid 174]: 25 (0.054%)<br>• Leptospira santarosai [taxid 28183]: 21 (0.046%)<br>• Leptospira alstonii [taxid 28452]: 17 (0.037%)<br>• Leptospira kmetyi [taxid 408139]: 16 (0.035%)<br>• Leptospira adleri [taxid 2023186]: 13 (0.028%)<br>• other: 159 (0.349%)
Benchmark OTU ID: `Rose_little_divergence_sequence_f-_Simulated`<br>OTU taxon: Leptospira interrogans serovar Copenhageni str. Fiocruz L1-130 [taxid 267671]<br>Expected: Leptospira interrogans [taxid 173] (species)<br>Number of reads: 45492<br>Number of identified reads: 43870 (96.434%)	• species: 16748 (36.815%)<br>• **genus: 18245 (40.105%)**<br>• family: 4 (0.008%)<br>• order: 0 (0.0%)<br>• class: 11 (0.024%)<br>• phylum: 9 (0.019%)<br>• superkingdom: 1649 (3.624%)<br>• root: 7182 (15.787%)	• Leptospira kirschneri [taxid 29507]: 139 (0.305%)<br>• Leptospira noguchii [taxid 28182]: 91 (0.2%)<br>• Leptospira weilii [taxid 28184]: 32 (0.07%)<br>• Leptospira borgpetersenii [taxid 174]: 27 (0.059%)<br>• Leptospira gomenensis [taxid 2484974]: 25 (0.054%)<br>• Leptospira santarosai [taxid 28183]: 18 (0.039%)<br>• Leptospira alstonii [taxid 28452]: 16 (0.035%)<br>• Leptospira perolatii [taxid 2023191]: 14 (0.03%)<br>• other: 156 (0.342%)
Benchmark OTU ID: `Rose_little_divergence_sequence_e-_Simulated`<br>OTU taxon: Leptospira interrogans serovar Copenhageni str. Fiocruz L1-130 [taxid 267671]<br>Expected: Leptospira interrogans [taxid 173] (species)<br>Number of reads: 45492<br>Number of identified reads: 43891 (96.48%)	• species: 16651 (36.602%)<br>• **genus: 18158 (39.914%)**<br>• family: 3 (0.006%)<br>• order: 0 (0.0%)<br>• class: 10 (0.021%)<br>• phylum: 13 (0.028%)<br>• superkingdom: 1681 (3.695%)<br>• root: 7352 (16.161%)	• Leptospira kirschneri [taxid 29507]: 143 (0.314%)<br>• Leptospira noguchii [taxid 28182]: 93 (0.204%)<br>• Leptospira weilii [taxid 28184]: 44 (0.096%)<br>• Leptospira borgpetersenii [taxid 174]: 34 (0.074%)<br>• Leptospira santarosai [taxid 28183]: 31 (0.068%)<br>• Leptospira alstonii [taxid 28452]: 20 (0.043%)<br>• Leptospira gomenensis [taxid 2484974]: 15 (0.032%)<br>• Leptospira ellisii [taxid 2023197]: 13 (0.028%)<br>• other: 153 (0.336%)

Operational Taxonomic Unit (OTU)	Correct identifications	Wrong or overspecific identifications at species rank
Benchmark OTU ID: `Rose_little_divergence_sequence_d-_Simulated`<br>OTU taxon: Leptospira interrogans serovar Copenhageni str. Fiocruz L1-130 [taxid 267671]<br>Expected: Leptospira interrogans [taxid 173] (species)<br>Number of reads: 45492<br>Number of identified reads: 43848 (96.386%)	• species: 16518 (36.309%)<br>• **genus: 18211 (40.031%)**<br>• family: 0 (0.0%)<br>• order: 0 (0.0%)<br>• class: 11 (0.024%)<br>• phylum: 8 (0.017%)<br>• superkingdom: 1696 (3.728%)<br>• root: 7375 (16.211%)	• Leptospira kirschneri [taxid 29507]: 134 (0.294%)<br>• Leptospira noguchii [taxid 28182]: 94 (0.206%)<br>• Leptospira weilii [taxid 28184]: 37 (0.081%)<br>• Leptospira santarosai [taxid 28183]: 29 (0.063%)<br>• Leptospira alstonii [taxid 28452]: 24 (0.052%)<br>• Leptospira perolatii [taxid 2023191]: 20 (0.043%)<br>• Leptospira gomenensis [taxid 2484974]: 16 (0.035%)<br>• Leptospira dzianensis [taxid 2484905]: 16 (0.035%)<br>• other: 141 (0.309%)
Benchmark OTU ID: `Rose_little_divergence_sequence_c-_Simulated`<br>OTU taxon: Leptospira interrogans serovar Copenhageni str. Fiocruz L1-130 [taxid 267671]<br>Expected: Leptospira interrogans [taxid 173] (species)<br>Number of reads: 45492<br>Number of identified reads: 43858 (96.408%)	• species: 16422 (36.098%)<br>• **genus: 18488 (40.64%)**<br>• family: 3 (0.006%)<br>• order: 0 (0.0%)<br>• class: 5 (0.01%)<br>• phylum: 9 (0.019%)<br>• superkingdom: 1653 (3.633%)<br>• root: 7252 (15.941%)	• Leptospira kirschneri [taxid 29507]: 162 (0.356%)<br>• Leptospira noguchii [taxid 28182]: 80 (0.175%)<br>• Leptospira weilii [taxid 28184]: 42 (0.092%)<br>• Leptospira borgpetersenii [taxid 174]: 27 (0.059%)<br>• Leptospira alstonii [taxid 28452]: 21 (0.046%)<br>• Leptospira santarosai [taxid 28183]: 20 (0.043%)<br>• Leptospira perolatii [taxid 2023191]: 14 (0.03%)<br>• Leptospira tipperaryensis [taxid 2564040]: 13 (0.028%)<br>• other: 166 (0.364%)
Benchmark OTU ID: `Rose_little_divergence_sequence_b-_Simulated`<br>OTU taxon: Leptospira interrogans serovar Copenhageni str. Fiocruz L1-130 [taxid 267671]<br>Expected: Leptospira interrogans [taxid 173] (species)<br>Number of reads: 45492<br>Number of identified reads: 43822 (96.329%)	• species: 16608 (36.507%)<br>• **genus: 18352 (40.341%)**<br>• family: 4 (0.008%)<br>• order: 0 (0.0%)<br>• class: 9 (0.019%)<br>• phylum: 5 (0.01%)<br>• superkingdom: 1706 (3.75%)<br>• root: 7107 (15.622%)	• Leptospira kirschneri [taxid 29507]: 160 (0.351%)<br>• Leptospira noguchii [taxid 28182]: 92 (0.202%)<br>• Leptospira weilii [taxid 28184]: 38 (0.083%)<br>• Leptospira santarosai [taxid 28183]: 34 (0.074%)<br>• Leptospira alstonii [taxid 28452]: 23 (0.05%)<br>• Leptospira borgpetersenii [taxid 174]: 22 (0.048%)<br>• Leptospira gomenensis [taxid 2484974]: 17 (0.037%)<br>• Leptospira perolatii [taxid 2023191]: 13 (0.028%)<br>• other: 159 (0.349%)
Benchmark OTU ID: `Rose_little_divergence_sequence_a-_Simulated`<br>OTU taxon: Leptospira interrogans serovar Copenhageni str. Fiocruz L1-130 [taxid 267671]<br>Expected: Leptospira interrogans [taxid 173] (species)<br>Number of reads: 45492<br>Number of identified reads: 43917 (96.537%)	• species: 16720 (36.753%)<br>• **genus: 18269 (40.158%)**<br>• family: 5 (0.01%)<br>• order: 0 (0.0%)<br>• class: 11 (0.024%)<br>• phylum: 10 (0.021%)<br>• superkingdom: 1706 (3.75%)<br>• root: 7174 (15.769%)	• Leptospira kirschneri [taxid 29507]: 155 (0.34%)<br>• Leptospira noguchii [taxid 28182]: 84 (0.184%)<br>• Leptospira weilii [taxid 28184]: 44 (0.096%)<br>• Leptospira borgpetersenii [taxid 174]: 31 (0.068%)<br>• Leptospira alstonii [taxid 28452]: 26 (0.057%)<br>• Leptospira santarosai [taxid 28183]: 25 (0.054%)<br>• Leptospira ellisii [taxid 2023197]: 15 (0.032%)<br>• Leptospira mayottensis [taxid 1137606]: 13 (0.028%)<br>• other: 170 (0.373%)

Operational Taxonomic Unit (OTU)	Correct identifications	Wrong or overspecific identifications at species rank
Benchmark OTU ID: `Rose_medium_divergence_sequence_i-_Simulated`<br>OTU taxon: Leptospira interrogans serovar Copenhageni str. Fiocruz L1-130 [taxid 267671]<br>Expected: Leptospira interrogans [taxid 173] (species)<br>Number of reads: 45520<br>Number of identified reads: 42749 (93.912%)	• species: 896 (1.968%)<br>• genus: 2412 (5.298%)<br>• family: 0 (0.0%)<br>• order: 0 (0.0%)<br>• class: 3 (0.006%)<br>• phylum: 5 (0.01%)<br>• superkingdom: 762 (1.673%)<br>• **root: 38532 (84.648%)**	• Leptospira kirschneri [taxid 29507]: 10 (0.021%)<br>• Leptospira weilii [taxid 28184]: 7 (0.015%)<br>• Leptospira noguchii [taxid 28182]: 7 (0.015%)<br>• Bacillus smithii [taxid 1479]: 3 (0.006%)<br>• Helicobacter pylori [taxid 210]: 3 (0.006%)<br>• Folsomia candida [taxid 158441]: 2 (0.004%)<br>• Vagococcus silagei [taxid 2508885]: 2 (0.004%)<br>• Leptospira barantonii [taxid 2023184]: 2 (0.004%)<br>• other: 142 (0.311%)
Benchmark OTU ID: `Rose_medium_divergence_sequence_h-_Simulated`<br>OTU taxon: Leptospira interrogans serovar Copenhageni str. Fiocruz L1-130 [taxid 267671]<br>Expected: Leptospira interrogans [taxid 173] (species)<br>Number of reads: 45511<br>Number of identified reads: 42605 (93.614%)	• species: 815 (1.79%)<br>• genus: 2022 (4.442%)<br>• family: 0 (0.0%)<br>• order: 0 (0.0%)<br>• class: 2 (0.004%)<br>• phylum: 4 (0.008%)<br>• superkingdom: 733 (1.61%)<br>• **root: 38900 (85.473%)**	• Leptospira noguchii [taxid 28182]: 7 (0.015%)<br>• Sulfuritortus calidifontis [taxid 1914471]: 3 (0.006%)<br>• Euryarchaeota archaeon [taxid 2026739]: 3 (0.006%)<br>• Leptospira borgpetersenii [taxid 174]: 3 (0.006%)<br>• Leptospira kirschneri [taxid 29507]: 3 (0.006%)<br>• Physcomitrium patens [taxid 3218]: 3 (0.006%)<br>• Anaeromyces robustus [taxid 1754192]: 2 (0.004%)<br>• Arachis hypogaea [taxid 3818]: 2 (0.004%)<br>• other: 129 (0.283%)
Benchmark OTU ID: `Rose_medium_divergence_sequence_g-_Simulated`<br>OTU taxon: Leptospira interrogans serovar Copenhageni str. Fiocruz L1-130 [taxid 267671]<br>Expected: Leptospira interrogans [taxid 173] (species)<br>Number of reads: 45420<br>Number of identified reads: 42599 (93.789%)	• species: 891 (1.961%)<br>• genus: 2274 (5.006%)<br>• family: 0 (0.0%)<br>• order: 0 (0.0%)<br>• class: 3 (0.006%)<br>• phylum: 3 (0.006%)<br>• superkingdom: 822 (1.809%)<br>• **root: 38464 (84.685%)**	• Leptospira kirschneri [taxid 29507]: 9 (0.019%)<br>• Leptospira noguchii [taxid 28182]: 7 (0.015%)<br>• Leptospira weilii [taxid 28184]: 5 (0.011%)<br>• Hepatitis B virus [taxid 10407]: 2 (0.004%)<br>• Streptomyces klenkii [taxid 1420899]: 2 (0.004%)<br>• Calidifontibacillus azotoformans [taxid 1454]: 2 (0.004%)<br>• Pleurotus ostreatus [taxid 5322]: 2 (0.004%)<br>• Hibiscus syriacus [taxid 106335]: 2 (0.004%)<br>• other: 130 (0.286%)
Benchmark OTU ID: `Rose_medium_divergence_sequence_f-_Simulated`<br>OTU taxon: Leptospira interrogans serovar Copenhageni str. Fiocruz L1-130 [taxid 267671]<br>Expected: Leptospira interrogans [taxid 173] (species)<br>Number of reads: 45507<br>Number of identified reads: 42677 (93.781%)	• species: 827 (1.817%)<br>• genus: 2142 (4.706%)<br>• family: 0 (0.0%)<br>• order: 0 (0.0%)<br>• class: 1 (0.002%)<br>• phylum: 1 (0.002%)<br>• superkingdom: 735 (1.615%)<br>• **root: 38796 (85.252%)**	• Leptospira kirschneri [taxid 29507]: 9 (0.019%)<br>• Leptospira noguchii [taxid 28182]: 8 (0.017%)<br>• Tanacetum cinerariifolium [taxid 118510]: 3 (0.006%)<br>• Leptospira weilii [taxid 28184]: 3 (0.006%)<br>• Phyllostomus discolor [taxid 89673]: 2 (0.004%)<br>• Rhodotorula toruloides [taxid 5286]: 2 (0.004%)<br>• Ramazzottius varieornatus [taxid 947166]: 2 (0.004%)<br>• Ornithodoros turicata [taxid 34597]: 2 (0.004%)<br>• other: 161 (0.353%)

Operational Taxonomic Unit (OTU)	Correct identifications	Wrong or overspecific identifications at species rank
Benchmark OTU ID: `Rose_medium_divergence_sequence_e-_Simulated`<br>OTU taxon: Leptospira interrogans serovar Copenhageni str. Fiocruz L1-130 [taxid 267671]<br>Expected: Leptospira interrogans [taxid 173] (species)<br>Number of reads: 45451<br>Number of identified reads: 42532 (93.577%)	• species: 920 (2.024%)<br>• genus: 2040 (4.488%)<br>• family: 1 (0.002%)<br>• order: 0 (0.0%)<br>• class: 0 (0.0%)<br>• phylum: 3 (0.006%)<br>• superkingdom: 756 (1.663%)<br>• **root: 38668 (85.076%)**	• Leptospira noguchii [taxid 28182]: 15 (0.033%)<br>• Leptospira kirschneri [taxid 29507]: 12 (0.026%)<br>• Leptospira weilii [taxid 28184]: 4 (0.008%)<br>• Leptospira borgpetersenii [taxid 174]: 2 (0.004%)<br>• Ralstonia solanacearum [taxid 305]: 2 (0.004%)<br>• Gonapodya prolifera [taxid 1123529]: 2 (0.004%)<br>• Rariglobus hedericola [taxid 2597822]: 2 (0.004%)<br>• Oidiodendron maius [taxid 78148]: 2 (0.004%)<br>• other: 151 (0.332%)
Benchmark OTU ID: `Rose_medium_divergence_sequence_d-_Simulated`<br>OTU taxon: Leptospira interrogans serovar Copenhageni str. Fiocruz L1-130 [taxid 267671]<br>Expected: Leptospira interrogans [taxid 173] (species)<br>Number of reads: 45468<br>Number of identified reads: 42703 (93.918%)	• species: 905 (1.99%)<br>• genus: 2403 (5.285%)<br>• family: 0 (0.0%)<br>• order: 0 (0.0%)<br>• class: 3 (0.006%)<br>• phylum: 0 (0.0%)<br>• superkingdom: 752 (1.653%)<br>• **root: 38500 (84.674%)**	• Leptospira kirschneri [taxid 29507]: 11 (0.024%)<br>• Leptospira noguchii [taxid 28182]: 11 (0.024%)<br>• Tanacetum cinerariifolium [taxid 118510]: 3 (0.006%)<br>• Vitis vinifera [taxid 29760]: 3 (0.006%)<br>• Bellilinea caldifistulae [taxid 360411]: 2 (0.004%)<br>• Micromonas pusilla [taxid 38833]: 2 (0.004%)<br>• Leptospira alstonii [taxid 28452]: 2 (0.004%)<br>• Leptospira dzoumogneensis [taxid 2484904]: 2 (0.004%)<br>• other: 134 (0.294%)
Benchmark OTU ID: `Rose_medium_divergence_sequence_c-_Simulated`<br>OTU taxon: Leptospira interrogans serovar Copenhageni str. Fiocruz L1-130 [taxid 267671]<br>Expected: Leptospira interrogans [taxid 173] (species)<br>Number of reads: 45476<br>Number of identified reads: 42792 (94.097%)	• species: 42 (0.092%)<br>• genus: 135 (0.296%)<br>• family: 0 (0.0%)<br>• order: 0 (0.0%)<br>• class: 1 (0.002%)<br>• phylum: 1 (0.002%)<br>• superkingdom: 477 (1.048%)<br>• **root: 41983 (92.319%)**	• Vibrio cholerae [taxid 666]: 3 (0.006%)<br>• Teladorsagia circumcincta [taxid 45464]: 3 (0.006%)<br>• Helianthus annuus [taxid 4232]: 3 (0.006%)<br>• Puccinia striiformis [taxid 27350]: 2 (0.004%)<br>• Bipolaris maydis [taxid 5016]: 2 (0.004%)<br>• Dendrobium catenatum [taxid 906689]: 2 (0.004%)<br>• Tetrahymena thermophila [taxid 5911]: 2 (0.004%)<br>• Piedraia hortae [taxid 147573]: 2 (0.004%)<br>• other: 129 (0.283%)
Benchmark OTU ID: `Rose_medium_divergence_sequence_a-_Simulated`<br>OTU taxon: Leptospira interrogans serovar Copenhageni str. Fiocruz L1-130 [taxid 267671]<br>Expected: Leptospira interrogans [taxid 173] (species)<br>Number of reads: 42218<br>Number of identified reads: 39553 (93.687%)	• species: 828 (1.961%)<br>• genus: 1959 (4.64%)<br>• family: 0 (0.0%)<br>• order: 0 (0.0%)<br>• class: 4 (0.009%)<br>• phylum: 0 (0.0%)<br>• superkingdom: 633 (1.499%)<br>• **root: 36010 (85.295%)**	• Leptospira noguchii [taxid 28182]: 9 (0.021%)<br>• Leptospira kirschneri [taxid 29507]: 9 (0.021%)<br>• Leptospira borgpetersenii [taxid 174]: 3 (0.007%)<br>• Reticulomyxa filosa [taxid 46433]: 2 (0.004%)<br>• Leptospira alstonii [taxid 28452]: 2 (0.004%)<br>• Leptospira fainei [taxid 48782]: 2 (0.004%)<br>• Exophiala spinifera [taxid 91928]: 2 (0.004%)<br>• Ampelomyces quisqualis [taxid 50730]: 2 (0.004%)<br>• other: 128 (0.303%)

Operational Taxonomic Unit (OTU)	Correct identifications	Wrong or overspecific identifications at species rank
Benchmark OTU ID: `Rose_mixed_divergence_sequence_i-_Simulated`<br>OTU taxon: Leptospira interrogans serovar Copenhageni str. Fiocruz L1-130 [taxid 267671]<br>Expected: Leptospira interrogans [taxid 173] (species)<br>Number of reads: 45492<br>Number of identified reads: 43863 (96.419%)	• species: 16711 (36.733%)<br>• **genus: 18091 (39.767%)**<br>• family: 3 (0.006%)<br>• order: 0 (0.0%)<br>• class: 9 (0.019%)<br>• phylum: 12 (0.026%)<br>• superkingdom: 1740 (3.824%)<br>• root: 7273 (15.987%)	• Leptospira kirschneri [taxid 29507]: 153 (0.336%)<br>• Leptospira noguchii [taxid 28182]: 69 (0.151%)<br>• Leptospira weilii [taxid 28184]: 38 (0.083%)<br>• Leptospira alstonii [taxid 28452]: 30 (0.065%)<br>• Leptospira borgpetersenii [taxid 174]: 26 (0.057%)<br>• Leptospira santarosai [taxid 28183]: 20 (0.043%)<br>• Leptospira ellisii [taxid 2023197]: 15 (0.032%)<br>• Leptospira alexanderi [taxid 100053]: 15 (0.032%)<br>• other: 169 (0.371%)
Benchmark OTU ID: `Rose_mixed_divergence_sequence_h-_Simulated`<br>OTU taxon: Leptospira interrogans serovar Copenhageni str. Fiocruz L1-130 [taxid 267671]<br>Expected: Leptospira interrogans [taxid 173] (species)<br>Number of reads: 45446<br>Number of identified reads: 43066 (94.763%)	• species: 0 (0.0%)<br>• genus: 3 (0.006%)<br>• family: 0 (0.0%)<br>• order: 0 (0.0%)<br>• class: 0 (0.0%)<br>• phylum: 1 (0.002%)<br>• superkingdom: 527 (1.159%)<br>• **root: 42380 (93.253%)**	• Botrytis paeoniae [taxid 278948]: 3 (0.006%)<br>• [Clostridium] populeti [taxid 37658]: 2 (0.004%)<br>• Zobellella taiwanensis [taxid 347535]: 2 (0.004%)<br>• Phaseolus vulgaris [taxid 3885]: 2 (0.004%)<br>• Flavobacterium terrigena [taxid 402734]: 2 (0.004%)<br>• Triticum turgidum [taxid 4571]: 2 (0.004%)<br>• Absidia glauca [taxid 4829]: 2 (0.004%)<br>• Phaeodactylum tricornutum [taxid 2850]: 2 (0.004%)<br>• other: 136 (0.299%)
Benchmark OTU ID: `Rose_mixed_divergence_sequence_g-_Simulated`<br>OTU taxon: Leptospira interrogans serovar Copenhageni str. Fiocruz L1-130 [taxid 267671]<br>Expected: Leptospira interrogans [taxid 173] (species)<br>Number of reads: 45492<br>Number of identified reads: 43859 (96.41%)	• species: 16700 (36.709%)<br>• **genus: 18309 (40.246%)**<br>• family: 5 (0.01%)<br>• order: 0 (0.0%)<br>• class: 13 (0.028%)<br>• phylum: 11 (0.024%)<br>• superkingdom: 1670 (3.67%)<br>• root: 7126 (15.664%)	• Leptospira kirschneri [taxid 29507]: 155 (0.34%)<br>• Leptospira noguchii [taxid 28182]: 83 (0.182%)<br>• Leptospira weilii [taxid 28184]: 57 (0.125%)<br>• Leptospira borgpetersenii [taxid 174]: 30 (0.065%)<br>• Leptospira alstonii [taxid 28452]: 21 (0.046%)<br>• Leptospira santarosai [taxid 28183]: 21 (0.046%)<br>• Leptospira gomenensis [taxid 2484974]: 13 (0.028%)<br>• Leptospira perolatii [taxid 2023191]: 12 (0.026%)<br>• other: 133 (0.292%)
Benchmark OTU ID: `Rose_mixed_divergence_sequence_f-_Simulated`<br>OTU taxon: Leptospira interrogans serovar Copenhageni str. Fiocruz L1-130 [taxid 267671]<br>Expected: Leptospira interrogans [taxid 173] (species)<br>Number of reads: 45455<br>Number of identified reads: 42932 (94.449%)	• species: 0 (0.0%)<br>• genus: 7 (0.015%)<br>• family: 0 (0.0%)<br>• order: 0 (0.0%)<br>• class: 0 (0.0%)<br>• phylum: 0 (0.0%)<br>• superkingdom: 515 (1.132%)<br>• **root: 42256 (92.962%)**	• Rhizobium laguerreae [taxid 1076926]: 3 (0.006%)<br>• Gymnoxanthella radiolariae [taxid 1798043]: 3 (0.006%)<br>• Afipia massiliensis [taxid 211460]: 3 (0.006%)<br>• Brugia malayi [taxid 6279]: 2 (0.004%)<br>• Breoghania corrubedonensis [taxid 665038]: 2 (0.004%)<br>• Gilliamella apicola [taxid 1196095]: 2 (0.004%)<br>• Rhodopirellula maiorica [taxid 1265734]: 2 (0.004%)<br>• Aureoumbra lagunensis [taxid 44058]: 2 (0.004%)<br>• other: 139 (0.305%)

Operational Taxonomic Unit (OTU)	Correct identifications	Wrong or overspecific identifications at species rank
Benchmark OTU ID: `Rose_mixed_divergence_sequence_e-_Simulated`<br>OTU taxon: Leptospira interrogans serovar Copenhageni str. Fiocruz L1-130 [taxid 267671]<br>Expected: Leptospira interrogans [taxid 173] (species)<br>Number of reads: 45492<br>Number of identified reads: 43898 (96.496%)	• species: 16754 (36.828%)<br>• **genus: 18187 (39.978%)**<br>• family: 3 (0.006%)<br>• order: 0 (0.0%)<br>• class: 14 (0.03%)<br>• phylum: 6 (0.013%)<br>• superkingdom: 1690 (3.714%)<br>• root: 7219 (15.868%)	• Leptospira kirschneri [taxid 29507]: 132 (0.29%)<br>• Leptospira noguchii [taxid 28182]: 86 (0.189%)<br>• Leptospira weilii [taxid 28184]: 48 (0.105%)<br>• Leptospira alstonii [taxid 28452]: 20 (0.043%)<br>• Leptospira santarosai [taxid 28183]: 19 (0.041%)<br>• Leptospira borgpetersenii [taxid 174]: 18 (0.039%)<br>• Leptospira barantonii [taxid 2023184]: 17 (0.037%)<br>• Leptospira mayottensis [taxid 1137606]: 14 (0.03%)<br>• other: 155 (0.34%)
Benchmark OTU ID: `Rose_mixed_divergence_sequence_c-_Simulated`<br>OTU taxon: Leptospira interrogans serovar Copenhageni str. Fiocruz L1-130 [taxid 267671]<br>Expected: Leptospira interrogans [taxid 173] (species)<br>Number of reads: 45435<br>Number of identified reads: 42685 (93.947%)	• species: 22 (0.048%)<br>• genus: 106 (0.233%)<br>• family: 0 (0.0%)<br>• order: 0 (0.0%)<br>• class: 1 (0.002%)<br>• phylum: 1 (0.002%)<br>• superkingdom: 425 (0.935%)<br>• **root: 41982 (92.4%)**	• Gluconobacter cerinus [taxid 38307]: 3 (0.006%)<br>• Talaromyces atroroseus [taxid 1441469]: 2 (0.004%)<br>• Gracilariopsis chorda [taxid 448386]: 2 (0.004%)<br>• Gossypium australe [taxid 47621]: 2 (0.004%)<br>• Skeletonema menzelii [taxid 216823]: 2 (0.004%)<br>• Mucuna pruriens [taxid 157652]: 2 (0.004%)<br>• Sphingobacterium psychroaquaticum [taxid 561061]: 2 (0.004%)<br>• Candidatus Woesearchaeota archaeon [taxid 2026803]: 2 (0.004%)<br>• other: 127 (0.279%)
Benchmark OTU ID: `Rose_mixed_divergence_sequence_b-_Simulated`<br>OTU taxon: Leptospira interrogans serovar Copenhageni str. Fiocruz L1-130 [taxid 267671]<br>Expected: Leptospira interrogans [taxid 173] (species)<br>Number of reads: 45492<br>Number of identified reads: 43882 (96.46%)	• species: 16735 (36.786%)<br>• **genus: 18308 (40.244%)**<br>• family: 2 (0.004%)<br>• order: 0 (0.0%)<br>• class: 14 (0.03%)<br>• phylum: 4 (0.008%)<br>• superkingdom: 1651 (3.629%)<br>• root: 7137 (15.688%)	• Leptospira kirschneri [taxid 29507]: 158 (0.347%)<br>• Leptospira noguchii [taxid 28182]: 92 (0.202%)<br>• Leptospira weilii [taxid 28184]: 32 (0.07%)<br>• Leptospira gomenensis [taxid 2484974]: 22 (0.048%)<br>• Leptospira borgpetersenii [taxid 174]: 22 (0.048%)<br>• Leptospira alstonii [taxid 28452]: 21 (0.046%)<br>• Leptospira santarosai [taxid 28183]: 21 (0.046%)<br>• Leptospira mayottensis [taxid 1137606]: 15 (0.032%)<br>• other: 150 (0.329%)
Benchmark OTU ID: `Rose_mixed_divergence_sequence_a-_Simulated`<br>OTU taxon: Leptospira interrogans serovar Copenhageni str. Fiocruz L1-130 [taxid 267671]<br>Expected: Leptospira interrogans [taxid 173] (species)<br>Number of reads: 42265<br>Number of identified reads: 39759 (94.07%)	• species: 35 (0.082%)<br>• genus: 73 (0.172%)<br>• family: 0 (0.0%)<br>• order: 0 (0.0%)<br>• class: 2 (0.004%)<br>• phylum: 0 (0.0%)<br>• superkingdom: 478 (1.13%)<br>• **root: 39044 (92.379%)**	• Tigriopus californicus [taxid 6832]: 3 (0.007%)<br>• Pseudomonas putida [taxid 303]: 2 (0.004%)<br>• Chaetoceros affinis [taxid 426623]: 2 (0.004%)<br>• Lactobacillus phage LfeInf [taxid 1567484]: 2 (0.004%)<br>• Saccharicrinis carchari [taxid 1168039]: 2 (0.004%)<br>• Lentinus tigrinus [taxid 5365]: 2 (0.004%)<br>• Rhizophagus irregularis [taxid 588596]: 2 (0.004%)<br>• Francisella adeliensis [taxid 2007306]: 2 (0.004%)<br>• other: 114 (0.269%)

Operational Taxonomic Unit (OTU)	Correct identifications	Wrong or overspecific identifications at species rank
Benchmark OTU ID: `Rose_most_divergence_sequence_h-_Simulated`<br>OTU taxon: Leptospira interrogans serovar Copenhageni str. Fiocruz L1-130 [taxid 267671]<br>Expected: Leptospira interrogans [taxid 173] (species)<br>Number of reads: 45464<br>Number of identified reads: 43083 (94.762%)	• species: 0 (0.0%)<br>• genus: 0 (0.0%)<br>• family: 0 (0.0%)<br>• order: 0 (0.0%)<br>• class: 0 (0.0%)<br>• phylum: 1 (0.002%)<br>• superkingdom: 562 (1.236%)<br>• **root: 42385 (93.227%)**	• Delftia acidovorans [taxid 80866]: 2 (0.004%)<br>• Togula jolla [taxid 285029]: 2 (0.004%)<br>• Scrippsiella trochoidea [taxid 71861]: 2 (0.004%)<br>• Alexandrium catenella [taxid 2925]: 2 (0.004%)<br>• Glomus cerebriforme [taxid 658196]: 2 (0.004%)<br>• Mixia osmundae [taxid 34349]: 2 (0.004%)<br>• Bifidobacterium reuteri [taxid 983706]: 2 (0.004%)<br>• Mycobacterium mantenii [taxid 560555]: 2 (0.004%)<br>• other: 129 (0.283%)
Benchmark OTU ID: `Rose_most_divergence_sequence_g-_Simulated`<br>OTU taxon: Leptospira interrogans serovar Copenhageni str. Fiocruz L1-130 [taxid 267671]<br>Expected: Leptospira interrogans [taxid 173] (species)<br>Number of reads: 45349<br>Number of identified reads: 42753 (94.275%)	• species: 3 (0.006%)<br>• genus: 3 (0.006%)<br>• family: 0 (0.0%)<br>• order: 0 (0.0%)<br>• class: 1 (0.002%)<br>• phylum: 1 (0.002%)<br>• superkingdom: 505 (1.113%)<br>• **root: 42083 (92.798%)**	• Pocillopora damicornis [taxid 46731]: 3 (0.006%)<br>• Eutreptiella gymnastica [taxid 73025]: 3 (0.006%)<br>• Salmonella enterica [taxid 28901]: 2 (0.004%)<br>• Paraburkholderia susongensis [taxid 1515439]: 2 (0.004%)<br>• Bacillus endozanthoxylicus [taxid 2036016]: 2 (0.004%)<br>• Bradyrhizobium erythrophlei [taxid 1437360]: 2 (0.004%)<br>• Cucurbita moschata [taxid 3662]: 2 (0.004%)<br>• Halomicronema hongdechloris [taxid 1209493]: 2 (0.004%)<br>• other: 140 (0.308%)
Benchmark OTU ID: `Rose_most_divergence_sequence_f-_Simulated`<br>OTU taxon: Leptospira interrogans serovar Copenhageni str. Fiocruz L1-130 [taxid 267671]<br>Expected: Leptospira interrogans [taxid 173] (species)<br>Number of reads: 45533<br>Number of identified reads: 43015 (94.469%)	• species: 1 (0.002%)<br>• genus: 2 (0.004%)<br>• family: 0 (0.0%)<br>• order: 0 (0.0%)<br>• class: 4 (0.008%)<br>• phylum: 0 (0.0%)<br>• superkingdom: 469 (1.03%)<br>• **root: 42393 (93.103%)**	• Arthrobotrys flagrans [taxid 97331]: 3 (0.006%)<br>• Pichia membranifaciens [taxid 4926]: 3 (0.006%)<br>• Clonorchis sinensis [taxid 79923]: 2 (0.004%)<br>• Xylaria flabelliformis [taxid 2512241]: 2 (0.004%)<br>• Niveispirillum cyanobacteriorum [taxid 1612173]: 2 (0.004%)<br>• Dendrothele bispora [taxid 1314803]: 2 (0.004%)<br>• Melampsora larici-populina [taxid 203908]: 2 (0.004%)<br>• Belliella baltica [taxid 232259]: 2 (0.004%)<br>• other: 135 (0.296%)
Benchmark OTU ID: `Rose_most_divergence_sequence_e-_Simulated`<br>OTU taxon: Leptospira interrogans serovar Copenhageni str. Fiocruz L1-130 [taxid 267671]<br>Expected: Leptospira interrogans [taxid 173] (species)<br>Number of reads: 45472<br>Number of identified reads: 42901 (94.345%)	• species: 0 (0.0%)<br>• genus: 8 (0.017%)<br>• family: 0 (0.0%)<br>• order: 0 (0.0%)<br>• class: 0 (0.0%)<br>• phylum: 2 (0.004%)<br>• superkingdom: 465 (1.022%)<br>• **root: 42268 (92.953%)**	• Uncinocarpus reesii [taxid 33188]: 3 (0.006%)<br>• Esox lucius [taxid 8010]: 3 (0.006%)<br>• Vibrio superstes [taxid 198815]: 2 (0.004%)<br>• Haloplanus rallus [taxid 1816183]: 2 (0.004%)<br>• Mytilus coruscus [taxid 42192]: 2 (0.004%)<br>• Fervidobacterium thailandense [taxid 1008305]: 2 (0.004%)<br>• Candidatus Pacearchaeota archaeon [taxid 2026773]: 2 (0.004%)<br>• Candidatus Bathyarchaeota archaeon [taxid 2026714]: 2 (0.004%)<br>• other: 135 (0.296%)

Operational Taxonomic Unit (OTU)	Correct identifications	Wrong or overspecific identifications at species rank
Benchmark OTU ID: `Rose_most_divergence_sequence_d-_Simulated`<br>OTU taxon: Leptospira interrogans serovar Copenhageni str. Fiocruz L1-130 [taxid 267671]<br>Expected: Leptospira interrogans [taxid 173] (species)<br>Number of reads: 45464<br>Number of identified reads: 42549 (93.588%)	• species: 906 (1.992%)<br>• genus: 2216 (4.874%)<br>• family: 0 (0.0%)<br>• order: 0 (0.0%)<br>• class: 1 (0.002%)<br>• phylum: 2 (0.004%)<br>• superkingdom: 702 (1.544%)<br>• **root: 38585 (84.869%)**	• Leptospira weilii [taxid 28184]: 9 (0.019%)<br>• Leptospira kirschneri [taxid 29507]: 7 (0.015%)<br>• Leptospira noguchii [taxid 28182]: 7 (0.015%)<br>• Tanacetum cinerariifolium [taxid 118510]: 4 (0.008%)<br>• Leptospira tipperaryensis [taxid 2564040]: 3 (0.006%)<br>• Karenia brevis [taxid 156230]: 3 (0.006%)<br>• Enterococcus saccharolyticus [taxid 41997]: 2 (0.004%)<br>• Natronorubrum tibetense [taxid 63128]: 2 (0.004%)<br>• other: 138 (0.303%)
Benchmark OTU ID: `Rose_most_divergence_sequence_c-_Simulated`<br>OTU taxon: Leptospira interrogans serovar Copenhageni str. Fiocruz L1-130 [taxid 267671]<br>Expected: Leptospira interrogans [taxid 173] (species)<br>Number of reads: 45425<br>Number of identified reads: 42956 (94.564%)	• species: 2 (0.004%)<br>• genus: 7 (0.015%)<br>• family: 0 (0.0%)<br>• order: 0 (0.0%)<br>• class: 2 (0.004%)<br>• phylum: 3 (0.006%)<br>• superkingdom: 477 (1.05%)<br>• **root: 42306 (93.133%)**	• Drosophila navojoa [taxid 7232]: 3 (0.006%)<br>• Tanacetum cinerariifolium [taxid 118510]: 3 (0.006%)<br>• Glarea lozoyensis [taxid 101852]: 2 (0.004%)<br>• Chlamydomonas eustigma [taxid 1157962]: 2 (0.004%)<br>• Schistocephalus solidus [taxid 70667]: 2 (0.004%)<br>• Leptospira terpstrae [taxid 293075]: 2 (0.004%)<br>• Arabis nemorensis [taxid 586526]: 2 (0.004%)<br>• Zygosaccharomyces parabailii [taxid 1365886]: 2 (0.004%)<br>• other: 151 (0.332%)
Benchmark OTU ID: `Rose_most_divergence_sequence_b-_Simulated`<br>OTU taxon: Leptospira interrogans serovar Copenhageni str. Fiocruz L1-130 [taxid 267671]<br>Expected: Leptospira interrogans [taxid 173] (species)<br>Number of reads: 45596<br>Number of identified reads: 43042 (94.398%)	• species: 0 (0.0%)<br>• genus: 1 (0.002%)<br>• family: 0 (0.0%)<br>• order: 0 (0.0%)<br>• class: 2 (0.004%)<br>• phylum: 0 (0.0%)<br>• superkingdom: 508 (1.114%)<br>• **root: 42341 (92.861%)**	• Carlito syrichta [taxid 1868482]: 3 (0.006%)<br>• Paramuricea clavata [taxid 317549]: 3 (0.006%)<br>• Dolichomastix tenuilepis [taxid 195969]: 3 (0.006%)<br>• Novosphingobium subterraneum [taxid 48936]: 3 (0.006%)<br>• Methanobrevibacter millerae [taxid 230361]: 3 (0.006%)<br>• Scytonema hofmannii [taxid 34078]: 2 (0.004%)<br>• Gilliamella apicola [taxid 1196095]: 2 (0.004%)<br>• Enterospora canceri [taxid 1081671]: 2 (0.004%)<br>• other: 155 (0.339%)
Benchmark OTU ID: `Rose_most_divergence_sequence_a-_Simulated`<br>OTU taxon: Leptospira interrogans serovar Copenhageni str. Fiocruz L1-130 [taxid 267671]<br>Expected: Leptospira interrogans [taxid 173] (species)<br>Number of reads: 42268<br>Number of identified reads: 39972 (94.567%)	• species: 0 (0.0%)<br>• genus: 3 (0.007%)<br>• family: 0 (0.0%)<br>• order: 0 (0.0%)<br>• class: 1 (0.002%)<br>• phylum: 0 (0.0%)<br>• superkingdom: 431 (1.019%)<br>• **root: 39384 (93.176%)**	• Pyramimonas obovata [taxid 1411642]: 3 (0.007%)<br>• Pseudomonas cremoricolorata [taxid 157783]: 2 (0.004%)<br>• Periconia macrospinosa [taxid 97972]: 2 (0.004%)<br>• Eumeta japonica [taxid 151549]: 2 (0.004%)<br>• Litonotus pictus [taxid 665100]: 2 (0.004%)<br>• Arsenophonus endosymbiont of Bemisia tabaci [taxid 536059]: 2 (0.004%)<br>• Guillardia theta [taxid 55529]: 2 (0.004%)<br>• Nosema apis [taxid 35231]: 2 (0.004%)<br>• other: 122 (0.288%)
